# Modelling the Repair of Carbon-Centered Protein Radicals by Phenolic Antioxidants

**DOI:** 10.3390/antiox13111368

**Published:** 2024-11-08

**Authors:** Max Walton-Raaby, Tyler Floen, Nelaine Mora-Diez

**Affiliations:** 1 Department of Chemistry, Thompson Rivers University, Kamloops, BC V2C 0C8, Canada; max.walton-raaby@uwaterloo.ca (M.W.-R.); tfloen@student.ubc.ca (T.F.); 2 Department of Chemistry, University of Waterloo, Waterloo, ON N2L 3G1, Canada; 3 Faculty of Pharmaceutical Sciences, University of British Columbia, Vancouver, BC V6T 1Z3, Canada

**Keywords:** DFT, M06-2X, SMD, antioxidants, phenols, leucine, repair, rate constants, HAT, SET, thermodynamics, kinetics

## Abstract

Oxidative stress is a biological process that has been linked to many diseases, hence understanding how to prevent and repair it is essential to medicine. The thermodynamics and kinetics of the repair reactions of radically damaged leucine (a lateral chain in a simplified protein environment) by twenty phenolic antioxidants are studied at the M06-2X(SMD)/6-31++G(d,p) level of theory in water and pentyl ethanoate. The two repair mechanisms modelled are formal-hydrogen atom transfer (f-HAT) and single electron transfer (SET). Although all f-HAT reactions are thermodynamically favourable, only one of the phenols produced rate constants in the diffusion limit, exhibiting biological relevance. SET is not suspected to be an important repair pathway for the phenols studied. We show that the Bell–Evans–Polanyi principle, which relates thermodynamics and kinetics properties for a reaction, breaks down when comparing between the solvents, protein repair sites, and the phenolic antioxidants. While thermodynamic data can be used as valuable screening tools, the kinetic calculation of rate constants in solution is crucial for enhancing the biological relevance of theoretical studies.

## 1. Introduction

In biological systems, proteins play an indispensable role in diverse biological processes. Due to their widespread biological presence, proteins are prone to oxidative damage, a form of biological stress caused by the imbalance between the production and elimination of free radicals. Free radicals are labile chemical species, capable of oxidizing proteins at very high rates [1], impairing their functioning. The oxidation of proteins has been linked to many diseases such as Alzheimer’s [2,3,4], diabetes [5,6], and cancer [4,7]; hence, understanding how to mitigate this damage is essential to medicine.

Exogenously sourced and endogenously produced, free radicals play a dual role in health, posing various risks while serving as essential components for maintaining homeostasis, controlling apoptosis, and regulating gene expression [8,9]. Exogenous sources of free radicals include pollution, certain drugs, UV radiation, heavy metals, and transition metals [2,10,11]. Free radicals are also produced endogenously during many biological processes (e.g., oxidative phosphorylation, phagocytosis, and cellular respiration) [2,10,11,12]. Notably, the most biologically prominent free radical, the hydroxyl radical [13], is capable of attacking virtually any biomolecule (e.g., DNA, RNA, protein, lipids, and cholesterol) within its short half-life (10^−9^ s) [1,9,12]. Conversely, species such as the hydroperoxyl and methoxy radicals are less reactive and damage biomolecules more selectively than the hydroxyl radical. Non-radical mechanisms of oxidative damage, such as the mechanism of hydrogen peroxide, can also occur [13]; however, free radicals are generally more biologically concerning due to their lability and tendency to initiate chain reactions.

Antioxidants are a class of molecules capable of quenching free radicals (primary activity), preventing the generation of free radicals (secondary activity), and repairing oxidatively damaged biomolecules (tertiary activity). Enzymatic antioxidants such as superoxide dismutase, catalase, glutathione peroxidase, and non-enzymatic antioxidants such as vitamins A and E, Trolox, and glutathione (GSH) have crucial roles in maintaining the delicate balance between the production and elimination of free radicals, thus contributing to homeostasis [8,9]. More specifically, enzymatic antioxidants are thought to be the primary line of defense against oxidative damage [14], while non-enzymatic ones are the secondary line of defense [11]. Despite the many positive effects of antioxidants, the body cannot differentiate between helpful and deleterious oxidants, hence the excessive consumption of antioxidants can block the positive effects of oxidants in the body [15,16]. Additionally, high concentrations of some phenolic antioxidants can have pro-oxidant action [15,17,18,19]. These conditions are known as antioxidative stress, which has similar effects to oxidative stress [15].

Reported studies primarily focused on the harmful reactions of free radicals with DNA and lipids, overlooking the biological significance of their interactions with proteins. Radical damage has the potential to induce protein aggregation, altered turnover rates, and loss of protein functioning [20]. Not all amino acids are equally affected by free radicals. For example, glycine undergoes the slowest reaction with the hydroxyl radical with a rate constant of 1.7 × 10^7^ M^−1^s^−1^, which is less than the diffusion limit (~1 × 10^8^ M^−1^s^−1^) [21]. Conversely, cysteine and tryptophan exhibit diffusion-controlled reactions, with rate constants of 3.4 × 10^10^ and 1.3 × 10^10^ M^−1^s^−1^, respectively [21]. Leucine is particularly prone to damage due to its long side chain [22] and undergoes diffusion-controlled reactions with the hydroxyl radical with a rate constant of 1.7 × 10^9^ M^−1^s^−1^ [21]. For this reason, we are focusing on the repair of damaged leucine residues, which have also been studied by our group with other antioxidants such as GSH, Trolox, and dihydrolipoic acid [22,23,24].

**Figure 1:**
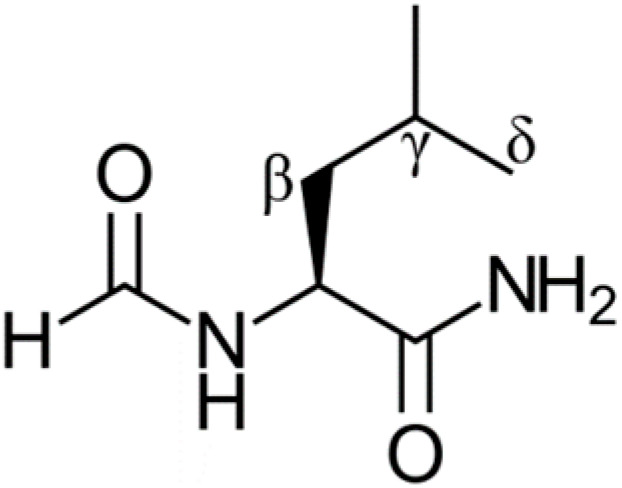
Structure of the N-formyl leucinamide protein model with labelled sites.

N-formyl leucinamide (shown in Figure 1 and referred to as “leucine” in this work) is the tripeptide molecule containing a leucine residue which will be used to represent a protein that has been exposed to oxidative stress [25]. This model has been employed successfully in similar theoretical studies [22,23,24] and correlates well with experimental data [26], and it has also been experimentally tested [27]. It contains four main sites of potential damage, the α, β, γ, and δ. Only the β, γ, and δ sites are investigated in this study since a radical at the α site would not be very reactive, and this would also be the least likely position to be damaged given its internal placement. Hence, the α site is not expected to be of biochemical concern. Furthermore, the repair of the α site with Trolox, GSH, and dihydrolipoic acid (DHLA) has been shown to produce small rate constants [22,23,24]. Our previous studies showed the biochemical effectiveness of DHLA and GSH to repair radically damaged leucine residues in proteins [22,23,24]. It would be of interest to compare their antioxidant repair activity with that of other compounds.

**Figure 2:**
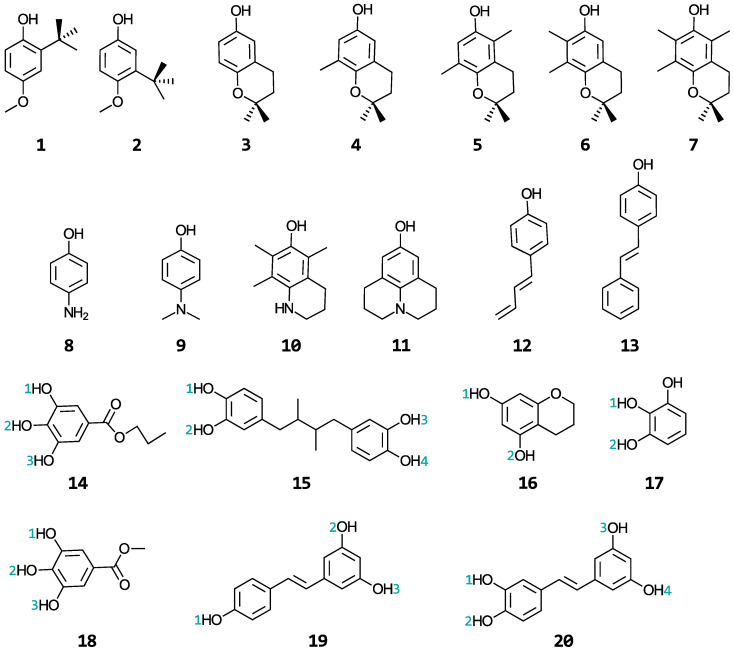
Structures of the phenolic antioxidants studied.

A wide range of phenolic antioxidants are investigated in this study: butylated hydroxyanisoles (BHA) (**1**–**2**), tocopherols (**3**–**7**), aminophenols (**8**–**11**), stilbenes related to resveratrol (**12**–**13** and **19**–**20**), propyl gallate (**14**), nordihydroguaiaretic acid (**15**), and the substructures of epigallocatechin-3-gallate (EGCG) (**16**–**18**). The structures and names of these molecules are shown in Figure 2 and Table S1 of the Supplementary Materials, respectively. BHA is a synthetic, lipid-soluble molecule that is commonly used as a preservative in foods to scavenge hydroperoxyl and alkyl peroxy radicals, preventing the oxidation of fats and oils [28,29]. The tocopherols are a family of four lipid-soluble molecules (α-, β-, γ-, and δ-tocopherol, with the α form being vitamin E) able to scavenge similar radicals as BHA in both biological and industrial contexts [29,30,31]. The aminophenols are synthetic molecules commonly used in cosmetics, dyes, and photographic developers [32,33]. The stilbenes are secondary metabolites produced by numerous types of plants [19,34] and have shown promise in treating cancers, Alzheimer’s, and heart diseases [15,18,35]. Propyl gallate and nordihydroguaiaretic acid, together with BHA, are used as food preservatives. EGCG is a famous antioxidant that is abundant in green tea with the potential for treating Alzheimer’s disease [36]. A relationship between the structural properties of these phenolic antioxidants and their ability to repair oxidatively damaged proteins will be established. These phenols were selected from a publication by Wright et al. [37] in which the authors developed a procedure for calculating their gas-phase bond dissociation enthalpies and ionization potentials and used these values to interpret relative rates for the reactions of these antioxidants with free radicals. As this study only considered two gas-phase properties of the antioxidants to make kinetic conclusions about their antioxidant activity when reacting with free radicals, we were motivated to perform the kinetic study of their repair reactions in a biologically relevant setting to establish comparisons.

In the present work, we aim to study the thermodynamics and kinetics of the formal-hydrogen atom transfer (f-HAT) and single electron transfer (SET) repair reactions at the β, γ, and δ leucine radical sites with phenolic antioxidants. Wright et al. [37] predicted that most of these phenols primarily react through f-HAT, except for molecules **10** and **11**, which may react primarily through SET. We will also evaluate the reliability of the Bell–Evans–Polanyi principle which suggests that the more thermodynamically favourable a reaction, the faster it should be [38,39]. We study the repair reactions of leucine using the same methodology as other studies by our group to develop a relative ranking of antioxidants [22,23,24]. Our methodology does not differentiate between f-HAT and proton-coupled electron transfer reaction; however, this distinction between reactions can be made by including a natural population analysis of atomic charges and a Hirshfeld partition scheme to analyze spin densities along the reaction coordinate [40]. Studying f-HAT and SET reactions will also provide insights into the viability of non-concerted repair mechanisms such as sequential proton loss electron transfer, sequential electron-proton transfer, and sequential proton loss hydrogen atom transfer, all of which include either f-HAT or SET reaction steps [41].

## 2. Computational Methodology

Calculations were performed at the M06-2X(SMD)/6-31++G(d,p) level of theory at 298.15 K using the Gaussian16 package [42]. The M06-2X functional is strongly recommended by its developers for main group thermochemistry and kinetic studies [43]. Geometry optimizations were followed by frequency calculations at the same level of theory. The reactants and products were confirmed to be energy minima, and the transition states (TSs) were confirmed to have only one imaginary frequency in which the correct TS is animated. Doublet systems were tested for spin contamination and all <S^2^> values were very close to 0.75 after annihilation (see Tables S2 and S3), indicating that spin contamination is not a concern in this study [44]. The standard absolute energies, enthalpies, and Gibbs free energies of all optimized species are provided in Tables S4 and S5 along with the Cartesian coordinates for all optimized geometries.

Solvent effects were considered in geometry optimizations and frequency calculations using the SMD continuum solvation method [45], which has been widely used in other thermodynamic and kinetic studies of antioxidant activity [22,23,24,46,47,48]. Water and pentyl ethanoate (PE) were the solvents utilized to simulate hydrophilic and hydrophobic (lipid) microenvironments, respectively. These two microenvironments were chosen since the damaged protein may be exposed to the solvent or buried within the structure of the overall protein.

The M06-2X functional combined with the SMD solvent model was one of the best performing combinations in a benchmark study that compared experimental and calculated (using the same methodology followed in our study) rate constants for a significant collection of radical–molecule reactions in solution [49]. We used the 6-31++G(d,p) basis set to keep consistency with our previous antioxidant studies of DHLA, GSH, and Trolox [22,23,24].

In the f-HAT repair reactions, a hydrogen atom is transferred from the hydroxyl group in the phenolic antioxidant to the damaged site of the leucine radical (Reaction R1). Rate constants were calculated applying conventional transition state theory using Equation (1), where σ represents the reaction path degeneracy (σ = 2 for the f-HAT reactions studied); κ is the tunnelling factor which was calculated for f-HAT reactions assuming a one-dimensional asymmetrical Eckart barrier using the Brown’s numeric integration program [50], k_B_ is the Boltzmann’s constant, T is the absolute temperature, h is Plank’s constant, and R is the ideal gas constant. The standard Gibbs free energies of activation (∆G^≠^) were converted to the 1 M reference state and the solvent cage effects were taken into account according to Okuno [51], which applies the free volume theory [52].

Phenol-OH + Leucine^•^ ⇌ Phenol-O^•^ + Leucine (R1)

(1) $$k=\sigma \kappa \frac{k_{B}T}{h}e^{\frac{-\Delta G^{\neq }}{RT}}$$

Reactions in solution are constrained by how quickly the reactants can diffuse to each other, which is known as the diffusion limit in the solvent considered. If the calculated rate constant exceeds 10^8^ M^−1^s^−1^, the apparent rate constant (k_app_) can be calculated according to Kimball–Collins theory [53] using Equation (2). In Equation (2), k_D_ is the steady-state Smoluchowski rate constant for an irreversible diffusion-controlled bimolecular reaction [54], which can be calculated using Equation (3).

(2) $$k_{app}=\frac{k_{D}k}{k_{D}+k}$$

(3) $$k_{D}=4\pi RD_{AB}N_{A}$$

In Equation (3), R is the distance between the atoms involved in the hydrogen atom transfer (reaction distance), N_A_ is Avogadro’s number, and D_AB_ is the mutual diffusion coefficient of the reactants, which is given as the sum of D_A_ and D_B_, which are calculated using the Stokes–Einstein approach [55,56] with Equation (4). In Equation (4), η is the viscosity of the solvent (8.91 × 10^−4^ Pa s for water and 8.62 × 10^−4^ Pa s for PE) and α is the radius of spherical solute A or B.

(4) $$D_{A\;or\;B}=\frac{k_{B}T}{6\pi \eta \alpha_{A\;or\;B}}$$

In the SET repair reactions, a deprotonated phenol donates an electron to the damaged leucine residue (Reaction R2). Hence, calculating k for the SET reaction requires the pK_a_ value of the phenolic antioxidant. Given the limited availability of experimental aqueous pK_a_ values for molecules **1** to **20**, predicted values were obtained from a recent publication by our group [57]. These values allow the calculation of the molar fraction of the anionic form of the antioxidants at physiological pH (7.4), which needs to be multiplied by the calculated SET rate constants using Equation (1), setting σ and κ to 1. The SET activation barriers ($∆G_{SET}^{\neq }$) were calculated applying Marcus theory [58,59] using Equation (5), where $∆G_{ET}^{0}$ is the Gibbs free energy of reaction, and λ is the nuclear reorganizational energy which defines the nonadiabatic transfer of an electron from reactants to vertical products. λ is calculated using Equation (6).

Phenol-O^−^ + Leucine^•^ ⇌ Phenol-O^•^ + Leucine^− ^ (R2)

(5) $$∆G_{SET}^{\neq }=\frac{\lambda }{4}(1+\frac{∆G_{ET}^{0}}{\lambda })$$

(6) $$\lambda \approx ∆E_{ET}-∆G_{ET}^{0}$$

As carried out in previous studies, if a reaction is exergonic, it is further studied from a kinetic point of view. The methodology described has been widely applied in the kinetic study of reactions in solution, including studies of antioxidant activity [22,23,24,26,46,47,48,60].

## 3. Results and Discussion

### 3.1. Investigating the Formal-Hydrogen Atom Transfer (f-HAT) Repair Reactions

The f-HAT reactions studied involve the transfer of a hydrogen atom from a phenol’s hydroxyl group. It is worth noting that molecules **1**–**13** have one hydroxyl group, whereas molecules **14**–**20** have multiple hydroxyl groups, thus multiple main sites of f-HAT reactivity. It is worth noting that molecules **14**–**20** may be capable of quenching multiple free radicals per antioxidant molecule. The contribution of other sites to f-HAT reactivity is considered negligible in this study. The predicted order of stability of the leucine radical is γ > β > δ, corresponding to tertiary, secondary, and primary radicals, respectively, which should be opposite to the thermodynamic favourabilities of their corresponding repair reactions.

Although many different orientations of the substituent groups were tested when searching for the optimized geometries of the various stationary points in this study, we are unable to guarantee that they are the lowest possible Gibbs energy conformations. Nevertheless, the calculations reported refer to the most thermodynamically favourable conformation identified, with particular attention to the polyphenols. The TSs were built using the optimized geometries of the corresponding reactants while maximizing the possibility of stabilizing interactions such as the formation of intermolecular hydrogen bonding and minimizing steric repulsions.

#### 3.1.1. Thermodynamic Study of f-HAT Reactions

The changes in free energy for the f-HAT reactions are listed in Table 1. Additionally, ∆H° values are provided in Tables S6 and S7 for the calculations in water and PE, respectively. All of the f-HAT repair reactions studied were exergonic in both solvents and were slightly more exergonic in PE than in water in all 13 cases except for the nitrogen-containing molecules (**8**–**11**). Unsurprisingly, the order of the repair site exergonicity was always δ > β > γ, which is a result of many factors, including the radical’s distance from the protein backbone, the primary, secondary, or tertiary (respectively) nature of the repair site, and the degree of hyperconjugation. Considering molecules **1**–**13**, the δ and β repairs have very similar ∆G° values with average differences of 0.86 and 1.15 kcal/mol in water and PE, respectively. However, the ∆G° values for the γ repairs are around 5.0 kcal/mol less negative than the δ repairs in both solvents. These values reflect the Gibbs energy differences between the various radicals to be repaired. The order of exergonicity will be compared to the kinetics results in the next section to test the Bell–Evans–Polanyi principle, which has already been shown to break down in previous antioxidant studies [22,23,24]. The Bell–Evans–Polanyi principle correlates the thermodynamics of a reaction with its kinetics: the more exergonic a reaction, the faster it should be.

When considering the food additive molecules **1** and **2**, **1** reacts more exergonically with leucine in both solvents, which is consistent with the predicted change in bond dissociation energy values, ∆BDE (O-H BDE relative to phenol), by Wright et al. [37] and the preferential use of **1** in industry [61,62]. At equivalent sites, reactions with molecule **1** are 2.1 to 2.2 kcal/mol more exergonic in water and 2.7 kcal/mol more exergonic in PE than with molecule **2**. The tert-butyl group increases the electron density in the phenolic ring and can stabilize the oxygen radical better when in ortho relative to it. Furthermore, the hydroxyl group in ortho-substituted BHA faces much more steric repulsion than in the meta-substituted case [63].

In the family of vitamin E molecules, **3**–**7**, molecule **7**, the most bioactive form, produced the most exergonic repair reactions with leucine (−22.4, −17.5, −23.5 kcal/mol at the β, γ, and δ sites in PE, respectively). In agreement with previous publications which studied BDE and ∆BDE, β-tocopherol (**5**) had slightly more exergonic reactions than γ-tocopherol (**6**) with leucine in both solvents [29,37,64]. Not surprisingly, the least exergonic molecule was the tocol model (**3**) due to the lack of substitution present relative to molecules **4**–**7**. Overall, the order of tocopherol exergonicities is **7** > **5** > **6** > **4** > **3**, which is consistent with the number of ortho methyl groups present, as well as the total number of substituent methyl groups.

For the four aminophenols studied (**8**–**11**), consistent trends in ∆G° between both solvent models and reaction sites were found. Molecule **10** produced the most exergonic repair reactions seen in this study (−29.9, −24.9, and −30.7 kcal/mol at the β, γ, and δ sites in water, respectively). As previously mentioned, these were the only molecules that produced more exergonic reactions in the hydrophilic environment than in the hydrophobic one. The f-HAT reactions of the stilbenes (**12**, **13**) were the least exergonic in both solvents studied when considering the monophenolic antioxidants.

Based on the results of the monophenolic antioxidants, we decided to examine only δ repair in PE for the polyphenolic antioxidants (**14**–**20**). This is because this site and solvent consistently produced the most exergonic reactions and largest repair rate constants (see Section 3.1.2.) for almost all of the monophenolic antioxidants. The other sites and solvent produced rate constants that were sometimes multiple orders lower; therefore, we deemed their contribution to the overall repair rate constants to be negligible.

As seen in Figure 2, molecules **14**, **17**, and **18** are structurally similar, each presenting three adjacent hydroxyl groups. As previously noted [37], when three hydroxyl groups are adjacent, the HAT reaction of the “internal” group is the most thermodynamically favoured. The “internal” oxygen radical is stabilized by hydrogen bonds from both “external” hydroxyl groups, whereas the “external” oxygen radical is only stabilized directly by the “internal” hydroxyl group. Our results follow this trend (Table 1). The stabilization of the “internal” oxygen radicals is also coupled with the electron-withdrawing (and stabilizing) effect of the ester groups in molecules **14** and **18**. Interestingly, despite the lack of additional resonance or electron delocalization relative to molecules **14** and **18**, molecule **17** produced the most exergonic reaction in PE of this study at its “internal” site (−23.9 kcal/mol). This behaviour of molecule **17**, relative to molecules **16** and **18**, is corroborated by the activity of EGCG, which is typically associated with the molecule **17** substructure and has shown similar results in other publications [37,65].

Molecules **19** and **20** are also structurally similar, with **20** having an additional hydroxyl group. In molecule **19**, the **19**^(2)^ and **19**^(3)^ reactions have similar exergonicities (0.2 kcal/mol difference), about 4.5 kcal/mol less negative when compared to the **19**^(1)^ site, which is supported by previous work [65,66]. A similar trend is seen in the **20**^(3)^ and **20**^(4)^ sites (with a difference of 0.7 kcal/mol). The Gibbs energy of the reaction is 9.0–10.0 kcal/mol more negative for the ortho-related hydroxyl groups, **20**^(1)^ and **20**^(2)^.

There are also common substituent effects observed among the polyphenolic antioxidants. The sites **14**^(2)^, **18**^(2)^, **19**^(1)^, and **20**^(2)^ are all para relative to electron withdrawing (by resonance and/or induction) substituents. Unsurprisingly, these hydroxyl sites produce the most exergonic reactions of these molecules.

#### 3.1.2. Kinetic Study of Hydrogen Atom Transfer Reactions

The kinetic study of a reaction should provide the best assessment of reactivity and should give us better tools for the comparison of antioxidant activity between different species when following a given methodology and with experimental results, when available. The experimental evaluation of specific types of antioxidant activity is challenging and most studies analyze the total antioxidant capacity of their samples. However, assigning individual antioxidant activities has been suggested as more important than determining the total antioxidant activity of a system [67], especially when designing synthetic antioxidants based on natural antioxidants. While theory gains insight by studying antioxidants in isolation, it also loses key information such as agonist and antagonist activities of species in the chemical environment.

N-formyl leucinamide has been used experimentally as a protein model, but the kinetics of its repair reactions with the phenols in this study have not been performed experimentally. However, the theoretical calculation of the rate constant of these reactions can allow the reactivity comparison between these phenols, and with previously studied antioxidants, in order to assess their tertiary antioxidant activity.

Following the thermodynamic study of the f-HAT repair reactions, the corresponding TSs were calculated. All the reactions were studied because they are exergonic. The degree of inter-molecular hydrogen bonding in the TS was generally consistent with its stability, but TSs leading to reactions with small Gibbs energies of activation were also found without these additional stabilizing interactions. The δ site repair generally had the lowest Gibbs energy of activation, followed by the β site. The γ site repair faced a large amount of steric repulsion, hence these TSs were built to minimize this and generally showed no inter-molecular hydrogen bonding. Nevertheless, many other factors, such as the presence of electron-withdrawing (EWG)/electron-donating (EDG) groups, local and through-bond effects, conjugation, hyperconjugation, solvent effects, and quantum tunnelling, contribute to the stability of the TS and the rate constant (k) of the reaction.

Table 1 displays the calculated Gibbs energy of activation (∆G^≠^) and k values in water and PE. The ∆G^≠^ values are always larger in water than in PE for equivalent repair positions of molecules **1**–**13** except for **4**(β) and **10**(γ). In general, k values in PE were larger than in water, except for seven of the β repairs (those of molecules **1**, **4**–**6**, **8**, **9**, and **11**) and two of the γ repairs (for molecules **2** and **10**). Hence, a general observation from our calculations is that these antioxidants might be more effective in repairing damaged leucine (and perhaps other aliphatic amino acid residues) in proteins within hydrophobic pockets than in a more solvent-exposed (hydrophilic) environment. Our discussion in this section will mainly focus on the magnitudes of k (instead of ∆G^≠^) since it includes the effects of quantum tunnelling (κ values between 1.0 and 113.3 were calculated as shown in Tables S6 and S7)), reaction path degeneracy, and the reaction temperature.

The food additive antioxidants (**1** and **2**), behave somewhat consistently with the results from the thermodynamic study, as seen in Table 1. Molecule **1** (with more exergonic repair reactions) has a larger rate constant than molecule **2** in all cases except at the γ site in water, with the fastest repair corresponding to the δ site in PE (k = 1.2 × 10^7^ M^−1^s^−1^).

The tocopherol molecules (**3**–**7**) present a similar overall trend to the thermodynamic study; the rate constant increases with the degree of substitution of the phenol, particularly with ortho substitutions. The fastest repair is produced by molecule **7**(δ) in PE with k = 1.7 × 10^7^ M^−1^s^−1^. It should be noted that the overall repair rate constant of molecule **7** is three orders of magnitude larger than the experimental radical lysozyme repair rate constant of 2.6 × 10^4^ M^−1^s^−1^ in sodium dodecyl sulphate (SDS) micelles [68]. Nevertheless, this comparison is indirect, as we are studying radically damaged leucine and the radical lysozyme mentioned in the study seems to be related to the presence of damaged tryptophan residues. Other fast repairs correspond to **3**(β), **6**(δ), and **7**(β) in PE with k values in the (1.0–6.9) × 10^6^ M^−1^s^−1^ range. One surprising observation regarding molecules **3**–**7** is that the β sites had larger rate constants than the δ sites in water, which disagrees with the thermodynamic predictions.

**Figure 3:**
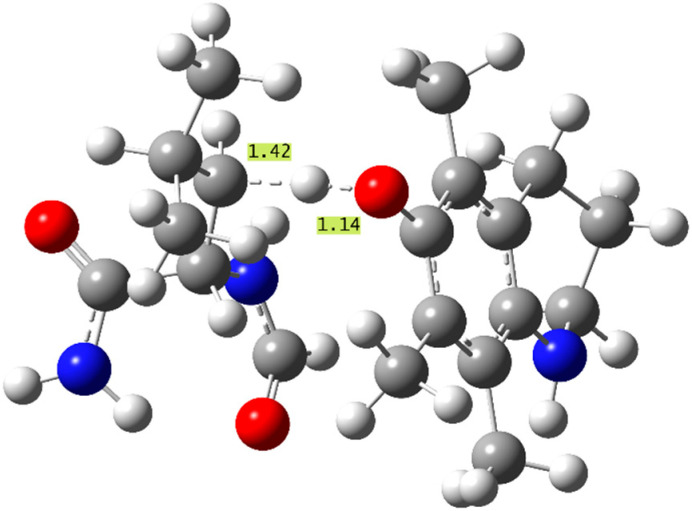
Structure of the f-HAT TS corresponding to the β repair of N-formyl leucinamide with molecule **10**, the fastest repair reaction studied (important bond distances shown in Å).

The aminophenol antioxidants (**8**–**11**) also present similar overall trends to the thermodynamic study. As predicted, the molecule exhibiting the most exergonic f-HAT repairs of all the phenols studied, **10**(β) and **10**(δ), shows the largest k values seen in this study, (1.2–1.4) × 10^8^ M^−1^s^−1^ in PE, which are also the only rate constants in the diffusion limit. These are the only effective f-HAT repair reactions studied, and they are proposed to quench the radical damage and compete with alternate protein radical mechanisms, such as peroxidation [69]. The structure of the TS leading to the fastest repair (β) by molecule **10** is displayed in Figure 3, with important bond distances indicated. This TS did not exhibit additional stabilizing interactions from inter-molecular hydrogen bonding between the reacting units.

Similar to molecule **7**, molecule **10** presents the most substitution among the aminophenols and has the largest rate constant. The other aminophenols produced the largest k values when repairing the δ site in PE. While this site selection corresponds to the thermodynamic prediction, it contradicts the prediction that water would be a more suitable solvent for repair by aminophenols.

Repair by the stilbenes (**12**–**13**) produced the smallest f-HAT rate constants in agreement with these reactions being the least exergonic. One inconsistency was found in molecule **12**, where the largest repair k value occurred in PE at the β site instead of the δ site (the most exergonic).

A few inconsistencies in trends between the thermodynamic and the kinetic study of the repair reactions of molecules **1**–**13** have already been pointed out when discussing the results of the four groups of phenols. In addition to these, we can observe that the thermodynamic study predicted that repairs at the δ site would have the largest rate constants in both PE and water; however, this was not always the case, with the β site producing a larger rate constant in 14 cases. Furthermore, the solvent producing the more thermodynamically favoured reactions did not lead to the fastest repairs in 13 cases.

Based on the thermodynamic results in PE (δ repair), the predicted order of rate constants would have been **10** > **11** > **9** > **8** > **7** > **1** > **5** ≈ **6** > **4** > **3** ≈ **2** > **12** > **13**, compared to the calculated order of **10** > **7** > **9** > **1** > **11** > **8** > **6** > **4** > **5** > **3** > **2** > **12** > **13**. These differences in thermodynamic and kinetic antioxidant activity predictions indicate a breakdown of the Bell–Evans–Polanyi principle. This is expected since thermodynamic calculations ignore structural and stability aspects of the TS and other kinetic features such as quantum tunnelling and reaction path degeneracy.

Because the fastest repair reaction for molecules **1**–**13** was for the δ position in PE, we decided to focus on this specific repair reaction when studying the tertiary antioxidant activity of the polyphenolic antioxidants (**14**–**20**). Overall rate constants that result from adding the site-specific k values for each molecule are displayed in Table 1. The largest overall k value (3.4 × 10^7^ M^−1^s^−1^) is produced by molecule **15**. For these molecules, none of the repair rate constants were in the diffusion limit, indicating that these repair reactions are not biologically relevant. This is corroborated for molecule **14** whose experimental repair rate constant of the lysozyme radical (in SDS micelles, a hydrophobic environment) is (9.9 ± 1.0) × 10^6^ M^−1^s^−1^ [68], in close proximity to our calculated rate constant of 1.12 × 10^7^ M^−1^s^−1^ for the repair of the δ position in leucine.

Many of the general thermodynamic predictions are reflected in the kinetic results. For example, the “internal” hydroxyl groups of **14**, **17**, and **18** (**14**^(2)^**, 17**^(1)^**,** and **18**^(2)^, see Figure 2) had larger rate constants than the “external” hydroxyl groups. As seen in the thermodynamic calculations, this is due to the stabilization of the radical via the “external” hydroxyl groups on either side which was also observed in the TSs. One interesting observation is that **17**^(1)^ produced a rate constant that is two orders larger (2.1 × 10^7^ M^−1^s^−1^) than its other hydroxyl group, while **14**^(2)^ and **18**^(2)^ produced rate constants in the same order as its other hydroxyl groups, (3.2–5.0) × 10^6^ M^−1^s^−1^, in general agreement with Brigati et al. [70] where the BDE of similar molecules were investigated. When an ester group is placed para relative to the hydroxyl group, the strength of the O-H bond increases, hence producing smaller rate constants. The larger rate constants of molecule **17** are also expected since this ring is typically associated with the activity of EGCG and has shown similar results in other publications [37,71], in agreement with our thermodynamic results.

Molecule **19**^(1)^ was predicted to have the largest repair rate constant when considering the thermodynamic study; however, **19**^(3)^ had a slightly larger rate constant which is likely due to the shorter inter-molecular hydrogen bonding that stabilizes its TS. Molecule **20** has its thermodynamic predictions reflected in its kinetic results, as previously described for molecules **14**, **17**, and **18**. As predicted, **20**^(3)^ and **20**^(4)^ had smaller rate constants than **20**^(1)^ and **20**^(2)^, with **20**^(2)^ having the largest repair rate constant (1.3 × 10^6^ M^−1^s^−1^). This is likely due to the stabilization of the radical from the ortho hydroxyl group and the conjugated system in the para position. This indicates that the TS stabilization from the ortho hydroxyl group in **20** is relevant in producing a larger rate constant, and although the para conjugated system may have led to a more exergonic reaction, it did not affect the rate constant to the same extent.

The thermodynamic results reported in Section 3.1.1., led us to believe that para substituents relative to the reacting hydroxyl group correspond to larger rate constants (**14**^(2)^, **18**^(2)^, **19**^(1)^, and **20**^(2)^). However, it is worth noting that **14**^(2)^, **18**^(2)^, and **20**^(2)^ also have ortho hydroxyl groups to stabilize the radical. Given the kinetic results obtained, we propose that the presence of an ortho hydroxyl group is a better predictor of kinetic behaviour than para electron-withdrawing substituents.

Further examining the thermodynamic and kinetic data for molecules **14**–**20**, additional violations of the Bell–Evans–Polanyi principle can be observed. Inspecting each molecule individually, this principle is not followed by molecules **15** and **19** (e.g., the most exergonic site does not produce the largest rate constant). This principle breaks down even further when comparing exergonicities and rate constants between molecules. For instance, the reaction of **20**^(2)^ was the third-most exergonic reaction in this study (ΔG° = −23.0 kcal/mol), yet it produced a rate constant smaller than all of the sites in molecules **14, 15,** and **18**. Other breakdowns of this principle can be found within our data, which indicates that thermodynamic data are insufficient to predict kinetic results, in agreement with previous work [22,24].

**Figure 4:**
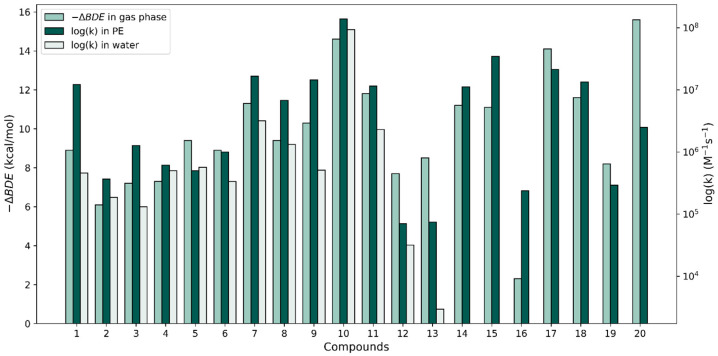
Comparison of −∆BDE values in the gas phase (from [37]) with our largest HAT log(k) values for the 20 compounds in water and PE.

To investigate the correlation between our rate constant calculations and the relative antioxidant activity of the phenolic antioxidants under study with the calculated gas-phase properties by Wright et al. [37], we plotted −ΔBDE versus the logarithm of the largest k value for each compound in both solvents (see Figure 4). With respect to the ΔBDE bars, the relative heights of the rate constants corresponding to both solvents are widely variable. In some cases, as with molecule **10**, the –ΔBDE value matches quite closely with the log(k) values; the two approaches predict this molecule to be very reactive when interacting with a radical via an HAT reaction. However, in other cases, the thermodynamic-kinetic reactivity predictions are very different. The ΔBDE values for molecules **10** and **20** are very similar and the thermodynamic approach predicts these molecules to be the most reactive of all; however, compound **20**’s k value is unimpressive in terms of its reactivity. Another example of contradictions found are the stilbenes (molecules **12** and **13**) which produced the smallest f-HAT rate constant values in both solvents, though their calculated gas-phase ΔBDE values are on par with those of molecules **3–6**, which have k values that are an order or larger than the k values of the stilbenes.

In summary, only molecule **10** produced rate constants in the diffusion limit for the f-HAT repair reaction showing biological significance. Important violations of the Bell–Evans–Polanyi principle have been discussed, which indicate that thermodynamic data are insufficient to properly predict kinetic antioxidant activity. However, thermodynamic data can be used as valuable screening tools [40,60]. Thermodynamic results also indicate the extent of products formed; however, it should be noted that endergonic reactions can still be viable antioxidant pathways if the products are consumed rapidly in subsequent reactions. From our experience, the kinetic calculation of rate constants is a valuable tool for predicting antioxidant activity and greatly increases the biological relevance of theoretical studies. We also discussed some of the contradictions between the calculated rate constants in solution and calculated gas-phase ΔBDE values when attempting to make reactivity predictions for the f-HAT reactions between these phenols and radicals.

### 3.2. Investigating the Single-Electron Transfer Repair Reaction

Multiple forms of SET reactions exist and can considerably contribute to a molecule’s overall antioxidant activity. We consider the case where the antioxidant has been deprotonated before the SET reaction. Since this type of SET reaction is only thermodynamically feasible in protic media, this study was only performed in water.

Table S8 displays the calculated Gibbs energy of reaction (∆G°) and activation (∆G^≠^), and the k values for the SET reactions. As can be observed, these reactions are highly endergonic and are not expected to contribute to the overall antioxidant activity of these molecules. In addition, their pK_a_ values are quite large [57], indicating that only a small molar fraction of the anionic form is available for this reaction at pH 7.4. In the case of the polyphenolic (polyprotic) molecules, the aqueous pK_a_ value for the most stable monoanion was calculated, as this represents the most dominant anion at pH 7.4 (see Table S9). The pK_a_ values for the monoanions were calculated using the ∆G°-pK_a_ correlation equation at the M06-2X(PCM)/6-311++G(d,p) level of theory reported in a recent publication by our group [57].

The calculated SET rate constants are extremely small and this result is in contradiction with the predictions of Wright et al. [37] based on calculated gas-phase ionization potentials and BDE values. They reported that the phenols studied primarily react with radicals through f-HAT, except for molecules **10** and **11**, which may react primarily through SET. While the SET reactions with radically damaged leucine are not predicted to be a viable repair mechanism, this does not reflect the ability of these molecules to engage in SET repair mechanisms with other free radicals (see Ref. [72], for example).

It is important to note that our SET rate constant calculations focus on the anionic species of each phenol as a reactant in aqueous solution, while the gas-phase adiabatic IP values calculated by Wright et al. [37] refer to the formation of the cation from the neutral phenol and account for the geometry changes upon removal of the electron. These IP values might have a better correlation with the rate constant of an SET reaction that focuses on the neutral phenol as a reactant, but this reaction would be of less biochemical relevance than that which we considered [73,74].

**Figure 5:**
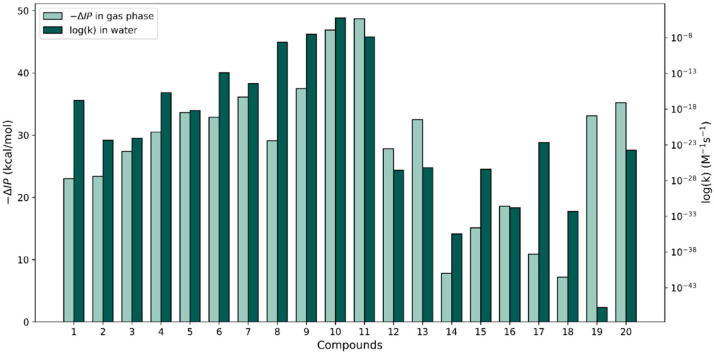
Comparison of −∆IP in the gas phase (from [37]) with our largest SET log(k) values for the 20 compounds in water.

Similar to the way that we proceeded with the f-HAT reactions, the calculated gas-phase –ΔIP values (calculated relative to the value of phenol) by Wright et al. [37] versus the largest log(k) values calculated for the SET reactions in water were plotted for each compound (see Figure 5). A better overall agreement between relative changes in the two properties could be appreciated for the set of molecules relative to our discussion on the −ΔBDE versus the largest f-HAT log(k) values. However, large differences that break trends can be observed for molecules **1**, **8**, **17**, **18**, and especially molecule **19**. Perhaps in the cases where the trends are kept, no significant geometry changes are produced between the neutral and the cation species in the gas phase. This might be the case because our k calculations for the SET process use vertical energy differences (no geometry relaxation is considered). In either type of ionization, the solvent presence should have an effect but we did not explore this further.

## 4. Conclusions

This paper evaluates the tertiary antioxidant activity of a family of phenols. The f-HAT and SET repair of oxidatively damaged N-formyl leucinamide was investigated at its β, γ, and δ sites with twenty phenolic molecules. Thermodynamic and kinetic quantities were calculated at the M06-2X(SMD)/6-31++G(d,p) level of theory in water and pentyl ethanoate to simulate the hydrophilic and hydrophobic microenvironments where a damaged leucine residue may be found.

SET is not an effective repair mechanism in water for the antioxidants studied, and while all the f-HAT repair reactions were exergonic, only one phenol (molecule **10**) produced rate constants in the diffusion limit showing biological significance at the level of dihydrolipoic acid and GSH [22,24]. A general observation from our calculations is that these antioxidants might be more effective at repairing damaged leucine (and perhaps other aliphatic amino acid residues) in proteins within hydrophobic pockets than in a more solvent-exposed (hydrophilic) environment. It is possible that our simplified protein model is not able to capture additional stabilizing intermolecular interactions involving other sites of the protein and the phenolic antioxidants, which would lead to the calculation of larger rate constants. This could be tested by modelling the leucine residue repair within solvent-exposed and hydrophobic sites of a protein which could be compared to previous experimental data [68,75]. Quantum mechanics/molecular mechanics (QM/MM) studies of leucine (in lysozyme) repair reactions with Trolox and GSH are the focus of another study currently in preparation by our group. In general, the conclusions derived from the smaller protein model [22] are validated by the QM/MM study.

While the results of the kinetic study were predicted by the thermodynamic calculation of Gibbs energies in some cases, several important inconsistencies were discussed that indicate the breakdown of the Bell–Evans–Polanyi principle. These breakdowns were found when comparing the solvents, protein repair sites, and the phenolic antioxidants. We also compared the trends between our calculated rate constants in solution and the antioxidant reactivity predictions made by Wright et al. [37] based on their calculated gas-phase thermodynamic quantities (O-H BDE and adiabatic IP values). Important inconsistencies were highlighted in this comparison for the radicals considered in this study. The evaluation of the primary antioxidant activity of this group of phenols when reacting with a simpler but biochemically relevant radical could be performed in a future study following the methodology applied in this work for comparison. Our experience in the evaluation of antioxidant activity indicates that while thermodynamic data can be used as valuable screening tools, the kinetic calculation of rate constants in solution is a more valuable approach and thus greatly increases the biological relevance of theoretical studies.

## Figures and Tables

**Table 1:** Standard Gibbs free energies of reaction (∆G°) and activation (∆G^≠^), and rate constants (k) for the f-HAT repair reactions of the corresponding damaged (dmg) N-formyl leucinamide (Leucine) in water and pentyl ethanoate (PE) at 298.15 K.

	∆G° (kcal/mol)		∆G^≠^ (kcal/mol)		k (M^−1^s^−1^)			∆BDE ^c^
Reactants	Water	PE	Water	PE	Water	PE	Overall (PE)	Gas
Leucine-β-dmg + 1	−19.6	−20.7	10.9	10.4	4.57 × 10^5^	3.04 × 10^5^		−8.9
Leucine-γ-dmg + 1	−14.6	−15.9	14.2	13.7	9.32 × 10^3^	1.59 × 10^4^		
Leucine-δ-dmg + 1	−20.4	−21.9	11.3	8.2	2.29 × 10^5^	1.22 × 10^7^		
Leucine-β-dmg + 2	−17.4	−18.0	15.5	13.0	5.74 × 10^3^	3.00 × 10^4^		−6.1
Leucine-γ-dmg + 2	−12.4	−13.2	14.5	14.5	1.19 × 10^4^	1.01 × 10^4^		
Leucine-δ-dmg + 2	−18.3	−19.2	12.7	11.8	1.86 × 10^5^	3.69 × 10^5^		
Leucine-β-dmg + 3	−17.6	−18.3	14.8	10.5	9.86 × 10^3^	1.27 × 10^6^		−7.2
Leucine-γ-dmg + 3	−12.6	−13.5	15.1	14.1	4.80 × 10^3^	1.64 × 10^4^		
Leucine-δ-dmg + 3	−18.5	−19.5	12.9	11.8	1.31 × 10^5^	4.40 × 10^5^		
Leucine-β-dmg + 4	−18.4	−18.7	11.4	11.6	4.98 × 10^5^	3.25 × 10^5^		−7.3
Leucine-γ-dmg + 4	−13.4	−13.9	15.2	13.6	4.46 × 10^3^	3.80 × 10^4^		
Leucine-δ-dmg + 4	−19.3	−19.9	12.9	11.6	1.28 × 10^5^	6.14 × 10^5^		
Leucine-β-dmg + 5	−20.0	−20.3	10.7	10.6	5.66 × 10^5^	3.53 × 10^5^		−9.4
Leucine-γ-dmg + 5	−15.0	−15.5	13.5	13.2	4.67 × 10^4^	5.63 × 10^4^		
Leucine-δ-dmg + 5	−20.9	−21.4	12.3	11.1	7.85 × 10^4^	4.98 × 10^5^		
Leucine-β-dmg + 6	−19.9	−20.1	11.2	11.0	3.34 × 10^5^	2.75 × 10^5^		−8.9
Leucine-γ-dmg + 6	−14.9	−15.3	13.9	12.8	2.37 × 10^4^	1.21 × 10^5^		
Leucine-δ-dmg + 6	−20.8	−21.3	12.7	11.1	1.36 × 10^5^	1.00 × 10^6^		
Leucine-β-dmg + 7	−22.3	−22.4	9.0	8.5	3.18 × 10^6^	6.86 × 10^6^		−11.3
Leucine-γ-dmg + 7	−17.3	−17.5	13.0	12.3	5.43 × 10^4^	1.57 × 10^5^		
Leucine-δ-dmg + 7	−23.2	−23.5	11.3	8.0	4.60 × 10^5^	1.66 × 10^7^		
Leucine-β-dmg + 8	−23.7	−22.6	11.4	11.1	1.32 × 10^6^	7.63 × 10^5^		−9.4
Leucine-γ-dmg + 8	−18.7	−17.7	13.8	12.9	3.86 × 10^4^	9.97 × 10^4^		
Leucine-δ-dmg + 8	−24.5	−23.7	12.5	9.9	1.84 × 10^5^	6.78 × 10^6^		
Leucine-β-dmg + 9	−25.0	−23.6	12.1	11.5	5.07 × 10^5^	3.08 × 10^5^		−10.3
Leucine-γ-dmg + 9	−20.0	−18.7	13.0	11.9	1.40 × 10^5^	5.16 × 10^5^		
Leucine-δ-dmg + 9	−25.9	−24.7	12.2	9.2	3.69 × 10^5^	1.45 × 10^7^		
Leucine-β-dmg + 10	−29.9	−27.7	6.9	6.6	**9.34 × 10** ^**7** a^	**1.38 × 10** ^**8** a^		−14.6
Leucine-γ-dmg + 10	−24.9	−22.9	9.8	11.3	1.93 × 10^6^	2.34 × 10^5^		
Leucine-δ-dmg + 10	−30.7	−28.9	9.3	6.7	1.92 × 10^6^	**1.23 × 10** ^**8** a^		
Leucine-β-dmg + 11	−27.5	−26.6	10.0	9.6	2.30 × 10^6^	1.21 × 10^6^		−11.8
Leucine-γ-dmg + 11	−22.5	−21.8	12.8	11.8	2.34 × 10^5^	4.14 × 10^5^		
Leucine-δ-dmg + 11	−28.3	−27.8	12.2	8.9	1.85 × 10^5^	1.15 × 10^7^		
Leucine-β-dmg + 12	−15.7	−16.1	14.3	13.0	3.18 × 10^4^	7.01 × 10^4^		−7.7
Leucine-γ-dmg + 12	−10.7	−11.2	16.1	15.3	1.01 × 10^3^	3.04 × 10^3^		
Leucine-δ-dmg + 12	−16.5	−17.2	14.6	13.5	1.34 × 10^4^	4.73 × 10^4^		
Leucine-β-dmg + 13	−14.5	−15.6	15.8	13.0	2.96 × 10^3^	6.87 × 10^4^		−8.5
Leucine-γ-dmg + 13	−9.5	−10.8	16.9	15.0	2.49 × 10^2^	4.85 × 10^3^		
Leucine-δ-dmg + 13	−15.4	−16.8	15.7	13.3	2.26 × 10^3^	7.46 × 10^4^		
Leucine-δ-dmg + 14 ^(1)^		−15.9		8.9		3.94 × 10^6^	1.12 × 10^7^	−11.2
Leucine-δ-dmg + 14 ^(2)^		−21.9		8.9		4.03 × 10^6^		
Leucine-δ-dmg + 14 ^(3)^		−16.3		9.0		3.23 × 10^6^		
Leucine-δ-dmg + 15 ^(1)^		−20.9		8.3		9.47 × 10^6^	3.44 × 10^7^	−11.1
Leucine-δ-dmg + 15 ^(2)^		−22.4		8.1		1.37 ×10^7^		
Leucine-δ-dmg + 15 ^(3)^		−23.6		8.6		6.48 × 10^6^		
Leucine-δ-dmg + 15 ^(4)^		−20.9		8.8		4.74 × 10^6^		
Leucine-δ-dmg + 16 ^(1)^		−14.0		10.9		1.38 × 10^5^	2.39 × 10^5^	−2.3 −1.3
Leucine-δ-dmg + 16 ^(2)^		−13.6		11.3		1.01 × 10^5^		
Leucine-δ-dmg + 17 ^(1)^		−23.9		7.9		2.10 × 10^7^	2.14 × 10^7^	−14.1
Leucine-δ-dmg + 17 ^(2)^		−18.0		11.0		1.98 × 10^5 b^		
Leucine-δ-dmg + 18 ^(1)^		−16.4		8.8		4.69 × 10^6^	1.34 × 10^7^	−11.6
Leucine-δ-dmg + 18 ^(2)^		−21.9		8.7		4.95 × 10^6^		
Leucine-δ-dmg + 18 ^(3)^		−15.9		8.9		3.80 × 10^6^		
Leucine-δ-dmg + 19 ^(1)^		−16.1		13.1		9.36 × 10^4^	2.91 × 10^5^	−8.2
Leucine-δ-dmg + 19 ^(2)^		−11.5		11.8		6.52 × 10^4^		
Leucine-δ-dmg + 19 ^(3)^		−11.7		11.3		1.33 × 10^5^		
Leucine-δ-dmg + 20 ^(1)^		−19.8		9.7		1.05 × 10^6^	2.49 × 10^6^	−15.6
Leucine-δ-dmg + 20 ^(2)^		−23.0		10.2		1.26 × 10^6^		
Leucine-δ-dmg + 20 ^(3)^		−11.6		11.7		3.20 × 10^4^		
Leucine-δ-dmg + 20 ^(4)^		−12.3		11.3		1.52 × 10^5^		

^a^ These are apparent rate constants (k_app_); ^b^ This value represents the rate constant for both degenerate sites combined; ^c^ Calculated bond dissociation enthalpy changes (relative to the value of phenol, in kcal/mol) in the gas phase reported in [37].

## Data Availability

Data available upon request.

## Supplementary Materials

The following supporting information can be downloaded at: https://www.mdpi.com/article/10.3390/antiox13111368/s1. The names of the molecules studied (Table S1); spin contamination values for doublet systems in water and PE (Tables S2 and S3, respectively); the absolute energies, enthalpies, and Gibbs free energies of all stationary points at the M06-2X(SMD)/6-31++G(d,p) level of theory in water and PE (Tables S4 and S5, respectively); the standard enthalpies of reaction and activation, imaginary frequencies, and tunnelling factors in water and PE (Tables S6 and S7, respectively); standard Gibbs free energies of reaction and activation, and rate constants for the SET reactions (Table S8); the standard Gibbs free energies for neutral and anionic forms in water, molar fractions for monoanionic forms, and predicted pK_a_ values for the polyphenolic antioxidant species (Table S9); optimized structures at the M06-2X(SMD)/6-31++G(d,p) level of theory in water and PE.
