# Supplementary material for: Modelling the Repair of Carbon-Centered Protein Radicals by Phenolic Antioxidants
Source: Antioxidants (Basel). 2024 Nov 8;13(11):1368. doi: 10.3390/antiox13111368 (PMC11591136; doi:10.3390/antiox13111368)
Supplement: Supplementary file 1 [file antioxidants-13-01368-s001.zip › antioxidants-3282764-supplementary.pdf]

Modelling the Repair of Carbon-Centered Protein Radicals by Phenolic Antioxidants

Max Walton-Raaby [1,2,†], Tyler Floen [1,†], and Nelaine Mora-Diez [1,*]

[1] Department of Chemistry, Thompson Rivers University, Kamloops, BC V2C 0C8, Canada

[2] Department of Chemistry, University of Waterloo, Waterloo, ON N2L 3G1, Canada

Electronic Supplementary Information

(215 pages)

Contents:

Table S1. Names and numeric labels of the phenolic antioxidants studied with structures shown in Figure 2.

Table S2. <S^2> values for doublet systems before and after spin annihilation in water.

Table S3. <S^2> values for doublet systems before and after spin annihilation in PE.

Table S4. Standard absolute energies, enthalpies, and Gibbs free energies (in atomic units) at 298.15 K for all stationary points in water at the M06-2X(SMD)/6-31++G(d,p) level of theory.

Table S5. Standard absolute energies, enthalpies, and Gibbs free energies (in atomic units) at 298.15 K for all stationary points in PE at the M06-2X(SMD)/6-31++G(d,p) level of theory.

Table S6. Standard enthalpies of reaction (ΔH°) and activation ($\Delta H^{\neq}$) in kcal/mol, imaginary vibrational frequencies ($v^{\neq}$, in cm^{-1}), and tunnelling factors (κ) at 298.15 K in water at the M06-2X(SMD)/6-31++G(d,p) level of theory.

Table S7. Standard enthalpies of reaction (ΔH°) and activation ($\Delta H^{\neq}$) in kcal/mol, imaginary vibrational frequencies ($v^{\neq}$, in cm^{-1}), and tunnelling factors (κ) at 298.15 K in PE at the M06-2X(SMD)/6-31++G(d,p) level of theory.

Table S8. Standard Gibbs free energies of reaction (ΔG°_{SET}) and activation ($\Delta G^{\neq}_{SET}$), and rate constants (k_{SET}) for the SET reactions studied.

Table S9. Standard Gibbs free energies (in atomic units) for neutral and anionic forms at the M06-2X(PCM)/6-311++G(d,p) level of theory in water, molar fractions for monoanionic forms, and predicted pK_a values for the polyphenolic antioxidant species.

Cartesian coordinates of the optimized antioxidant species studied at the M06-2X(SMD)/6-31++G(d,p) level of theory in water and in pentyl ethanoate.

---

[*] Corresponding author e-mail: nmora@tru.ca; † These authors contributed equally to the calculations reported and the preparation of the first draft.

Table S1. Names and numeric labels of the phenolic antioxidants studied with structures shown in Figure 2.

Number	Name
**1**	ortho-butylated hydroxyanisole
**2**	meta-butylated hydroxyanisole
**3**	tocol
**4**	δ-tocopherol
**5**	β-tocopherol
**6**	γ-tocopherol
**7**	α-tocopherol
**8**	4-aminophenol
**9**	N,N-dimethyl-4-aminophenol
**10**	6-hydroxy-5,7,8-trimethyl-1,2,3,4-tetrahydroquinoline
**11**	9-hydroxyjulolidine
**12**	4-butadienylphenol
**13**	4-vinylphenylphenol
**14**	propyl gallate
**15**	nordihydroguaiaretic acid
**16**	epigallocatechin-3-gallate ring 1
**17**	epigallocatechin-3-gallate ring 2
**18**	epigallocatechin-3-gallate ring 3
**19**	trans-resveratrol
**20**	piceatannol

Table S2. $<S^2>$ values for doublet systems before and after spin annihilation in water.

Species	$<S^2>$ before annihilation	$<S^2>$ after annihilation
Leucine-β-dmg	0.7553	0.7500
Leucine-γ-dmg	0.7552	0.7500
Leucine-δ-dmg	0.7549	0.7500
1-dmg	0.7735	0.7503
2-dmg	0.7740	0.7503
3-dmg	0.7739	0.7503
4-dmg	0.7738	0.7503
5-dmg	0.7733	0.7503
6-dmg	0.7734	0.7503
7-dmg	0.7726	0.7503
8-dmg	0.7699	0.7501
9-dmg	0.7711	0.7501
10-dmg	0.7691	0.7501
11-dmg	0.7704	0.7501
12-dmg	0.8046	0.7517
13-dmg	0.7952	0.7512
$14^{(1)}$-dmg	0.7765	0.7505
$14^{(2)}$-dmg	0.7792	0.7506
$14^{(3)}$-dmg	0.7769	0.7505
$15^{(1)}$-dmg	0.7734	0.7503
$15^{(2)}$-dmg	0.7727	0.7503
$15^{(3)}$-dmg	0.7727	0.7503
$15^{(4)}$-dmg	0.7734	0.7503
$16^{(1)}$-dmg	0.7825	0.7506
$16^{(2)}$-dmg	0.7843	0.7507
$17^{(1)}$-dmg	0.7748	0.7504
$17^{(2)}$-dmg	0.7750	0.7504
$18^{(1)}$-dmg	0.7765	0.7505
$18^{(2)}$-dmg	0.7793	0.7506
$18^{(3)}$-dmg	0.7768	0.7505
$19^{(1)}$-dmg	0.7954	0.7512
$19^{(2)}$-dmg	0.7906	0.7510
$19^{(3)}$-dmg	0.7919	0.7511
$20^{(1)}$-dmg	0.7725	0.7503
$20^{(2)}$-dmg	0.7868	0.7508
$20^{(3)}$-dmg	0.7907	0.7510
$20^{(4)}$-dmg	0.7919	0.7511
1-β-TS	0.7624	0.7501
1-γ-TS	0.7627	0.7501
1-δ-TS	0.7616	0.7501

2-β-TS	0.7635	0.7501
2-γ-TS	0.7627	0.7501
2-δ-TS	0.7617	0.7501
3-β-TS	0.7624	0.7501
3-γ-TS	0.7629	0.7501
3-δ-TS	0.7618	0.7501
4-β-TS	0.7628	0.7501
4-γ-TS	0.7629	0.7501
4-δ-TS	0.7620	0.7501
5-β-TS	0.7624	0.7501
5-γ-TS	0.7627	0.7501
5-δ-TS	0.7621	0.7501
6-β-TS	0.7625	0.7501
6-γ-TS	0.7630	0.7501
6-δ-TS	0.7615	0.7501
7-β-TS	0.7620	0.7501
7-γ-TS	0.7624	0.7501
7-δ-TS	0.7614	0.7501
8-β-TS	0.7625	0.7501
8-γ-TS	0.7627	0.7501
8-δ-TS	0.7619	0.7501
9-β-TS	0.7629	0.7501
9-γ-TS	0.7629	0.7501
9-δ-TS	0.7621	0.7501
10-β-TS	0.7622	0.7501
10-γ-TS	0.7625	0.7501
10-δ-TS	0.7615	0.7501
11-β-TS	0.7634	0.7501
11-γ-TS	0.7637	0.7501
11-δ-TS	0.7627	0.7501
12-β-TS	0.7661	0.7502
12-γ-TS	0.7678	0.7502
12-δ-TS	0.7648	0.7502
13-β-TS	0.7653	0.7502
13-γ-TS	0.7670	0.7502
13-δ-TS	0.7644	0.7502

Table S3. <S^2> values for doublet systems before and after spin annihilation in PE.

Species	<S^2> before annihilation	<S^2> after annihilation
Leucine-β-dmg	0.7554	0.7500
Leucine-γ-dmg	0.7552	0.7500
Leucine-δ-dmg	0.7549	0.7500
1-dmg	0.7772	0.7504
2-dmg	0.7792	0.7504
3-dmg	0.7786	0.7504
4-dmg	0.7788	0.7504
5-dmg	0.7777	0.7504
6-dmg	0.7778	0.7504
7-dmg	0.7767	0.7504
8-dmg	0.7745	0.7502
9-dmg	0.7745	0.7502
10-dmg	0.7728	0.7502
11-dmg	0.7740	0.7502
12-dmg	0.8085	0.7521
13-dmg	0.8003	0.7516
$14^{(1)}$-dmg	0.7801	0.7506
$14^{(2)}$-dmg	0.7796	0.7506
$14^{(3)}$-dmg	0.7795	0.7506
$15^{(1)}$-dmg	0.7758	0.7504
$15^{(2)}$-dmg	0.7749	0.7504
$15^{(3)}$-dmg	0.7749	0.7504
$15^{(4)}$-dmg	0.7757	0.7504
$16^{(1)}$-dmg	0.7870	0.7508
$16^{(2)}$-dmg	0.7880	0.7509
$17^{(1)}$-dmg	0.7759	0.7505
$17^{(2)}$-dmg	0.7781	0.7505
$18^{(1)}$-dmg	0.7795	0.7506
$18^{(2)}$-dmg	0.7797	0.7506
$18^{(3)}$-dmg	0.7801	0.7506
$19^{(1)}$-dmg	0.8002	0.7516
$19^{(2)}$-dmg	0.7958	0.7514
$19^{(3)}$-dmg	0.7964	0.7514
$20^{(1)}$-dmg	0.7756	0.7504
$20^{(2)}$-dmg	0.7883	0.7510
$20^{(3)}$-dmg	0.7946	0.7513
$20^{(4)}$-dmg	0.7964	0.7514
1-β-TS	0.7628	0.7501
1-γ-TS	0.7632	0.7501
1-δ-TS	0.7617	0.7501

2-β-TS	0.7634	0.7501
2-γ-TS	0.7635	0.7501
2-δ-TS	0.7620	0.7501
3-β-TS	0.7629	0.7501
3-γ-TS	0.7632	0.7501
3-δ-TS	0.7620	0.7501
4-β-TS	0.7632	0.7501
4-γ-TS	0.7636	0.7501
4-δ-TS	0.7622	0.7501
5-β-TS	0.7629	0.7501
5-γ-TS	0.7632	0.7501
5-δ-TS	0.7618	0.7501
6-β-TS	0.7628	0.7501
6-γ-TS	0.7634	0.7501
6-δ-TS	0.7617	0.7501
7-β-TS	0.7625	0.7501
7-γ-TS	0.7628	0.7501
7-δ-TS	0.7614	0.7501
8-β-TS	0.7629	0.7501
8-γ-TS	0.7632	0.7501
8-δ-TS	0.7622	0.7501
9-β-TS	0.7636	0.7501
9-γ-TS	0.7634	0.7501
9-δ-TS	0.7623	0.7501
10-β-TS	0.7626	0.7501
10-γ-TS	0.7630	0.7501
10-δ-TS	0.7614	0.7501
11-β-TS	0.7639	0.7501
11-γ-TS	0.7644	0.7501
11-δ-TS	0.7631	0.7501
12-β-TS	0.7665	0.7502
12-γ-TS	0.7685	0.7503
12-δ-TS	0.7651	0.7502
13-β-TS	0.7654	0.7502
13-γ-TS	0.7677	0.7502
13-δ-TS	0.7645	0.7502
$14^{(1)}$-δ-TS	0.7612	0.7501
$14^{(2)}$-δ-TS	0.7608	0.7501
$14^{(3)}$-δ-TS	0.7612	0.7501
$15^{(1)}$-δ-TS	0.7613	0.7501
$15^{(2)}$-δ-TS	0.7609	0.7501
$15^{(3)}$-δ-TS	0.7609	0.7501
$15^{(4)}$-δ-TS	0.7613	0.7501
$16^{(1)}$-δ-TS	0.7623	0.7501

16$^{(2)}$-δ-TS	0.7622	0.7501
17$^{(1)}$-δ-TS	0.7601	0.7501
17$^{(2)}$-δ-TS	0.7610	0.7501
18$^{(1)}$-δ-TS	0.7611	0.7501
18$^{(2)}$-δ-TS	0.7608	0.7501
18$^{(3)}$-δ-TS	0.7612	0.7501
19$^{(1)}$-δ-TS	0.7644	0.7502
19$^{(2)}$-δ-TS	0.7626	0.7501
19$^{(3)}$-δ-TS	0.7628	0.7501
20$^{(1)}$-δ-TS	0.7617	0.7501
20$^{(2)}$-δ-TS	0.7626	0.7501
20$^{(3)}$-δ-TS	0.7626	0.7501
20$^{(4)}$-δ-TS	0.7629	0.7501

Table S4. Standard absolute energies, enthalpies, and Gibbs free energies (in atomic units) at 298.15 K for all stationary points in water at the M06-2X(SMD)/6-31++G(d,p) level of theory.

Species	E°	H°	G°
Leucine	-534.75811	-534.75717	-534.80951
Leucine-β-dmg	-534.10520	-534.10425	-534.15756
Leucine-γ-dmg	-534.11227	-534.11133	-534.16554
Leucine-δ-dmg	-534.10391	-534.10296	-534.15618
Leucine-β-anion	-534.21117	-534.21022	-534.26252
Leucine-γ-anion	-534.20393	-534.20299	-534.25450
Leucine-δ-anion	-534.20724	-534.20630	-534.25788
1	-578.76182	-578.76087	-578.81509
1-dmg	-578.14213	-578.14118	-578.19434
1 anion	-578.29746	-578.29652	-578.34972
2	-578.76415	-578.76320	-578.81657
2-dmg	-578.14008	-578.13913	-578.19240
2 anion	-578.29870	-578.29776	-578.35055
3	-577.61365	-577.61271	-577.66177
3-dmg	-576.98953	-576.98858	-577.03786
3 anion	-577.14889	-577.14795	-577.19634
4	-616.88353	-616.88258	-616.93512
4-dmg	-616.26070	-616.25976	-616.31249
4 anion	-616.41807	-616.41712	-616.46916
5	-656.15195	-656.15101	-656.20732
5-dmg	-655.53173	-655.53078	-655.58723
5 anion	-655.68511	-655.68416	-655.73995
6	-656.15004	-656.14909	-656.20594
6-dmg	-655.52945	-655.52851	-655.58570
6 anion	-655.68325	-655.68231	-655.73838
7	-695.41724	-695.41629	-695.47645
7-dmg	-694.79985	-694.79890	-694.86008
7 anion	-694.94980	-694.94885	-695.00868
8	-362.57997	-362.57903	-362.61765
8-dmg	-361.96541	-361.96447	-362.00341
8 anion	-362.11393	-362.11299	-362.15145
9	-441.08942	-441.08848	-441.13360
9-dmg	-440.47630	-440.47535	-440.52152
9 anion	-440.62495	-440.62401	-440.66770
10	-597.00121	-597.00027	-597.05554
10-dmg	-596.39666	-596.39571	-596.45118
10 anion	-596.53262	-596.53167	-596.58639
11	-595.82412	-595.82318	-595.87206
11-dmg	-595.21490	-595.213959	-595.26386
11 anion	-595.35784	-595.35690	-595.40494

12	-461.92433	-461.92338	-461.97108
12-dmg	-461.29796	-461.29702	-461.34411
12 anion	-461.46521	-461.46426	-461.51191
13	-615.47814	-615.47719	-615.53218
13-dmg	-614.85107	-614.85013	-614.90335
13 anion	-615.01853	-615.01759	-615.07089
14(1)-dmg	-763.32211	-763.32116	-763.38161
14(2)-dmg	-763.32961	-763.32866	-763.38831
14(3)-dmg	-763.32173	-763.32079	-763.38135
14(1) anion	-763.50050	-763.49956	-763.55842
14(2) anion	-763.50723	-763.50629	-763.56459
14(3) anion	-763.50058	-763.49964	-763.55888
15(1)-dmg	-998.71827	-998.71732	-998.79196
15(2)-dmg	-998.72019	-998.71924	-998.79402
15(3)-dmg	-998.72011	-998.71917	-998.79391
15(4)-dmg	-998.71842	-998.71748	-998.79291
15(1) anion	-998.88469	-998.88375	-998.95783
15(2) anion	-998.88404	-998.88310	-998.95808
15(3) anion	-998.88419	-998.88324	-998.95730
15(4) anion	-998.88470	-998.88375	-998.95777
16(1)-dmg	-573.64299	-573.64204	-573.68833
16(2)-dmg	-573.64192	-573.64098	-573.68737
16(1) anion	-573.81488	-573.81394	-573.85914
16(2) anion	-573.81587	-573.81492	-573.86035
17(1)-dmg	-457.03772	-457.03677	-457.07725
17(2)-dmg	-457.02918	-457.02823	-457.06872
17(1) anion	-457.20450	-457.20356	-457.24367
17(2) anion	-457.20108	-457.20014	-457.24022
18(1)-dmg	-684.78610	-684.78516	-684.83784
18(2)-dmg	-684.79346	-684.79252	-684.84406
18(3)-dmg	-684.78557	-684.78463	-684.83616
18(1) anion	-684.96472	-684.96378	-685.01515
18(2) anion	-684.97139	-684.97044	-685.02125
18(3) anion	-684.96462	-684.96368	-685.01469
19(1)-dmg	-765.25987	-765.25892	-765.31782
19(2)-dmg	-765.25053	-765.24959	-765.30918
19(3)-dmg	-765.25093	-765.24999	-765.30960
19(1) anion	-765.42825	-765.42730	-765.48615
19(2) anion	-765.42681	-765.42587	-765.48619
19(3) anion	-765.42713	-765.42619	-765.48731
20(1)-dmg	-840.46427	-840.46333	-840.52521
20(2)-dmg	-840.46954	-840.46860	-840.52957
20(3)-dmg	-840.45199	-840.45104	-840.51356
20(4)-dmg	-840.45214	-840.45119	-840.51478

20(1) anion	-840.63336	-840.63242	-840.69326
20(2) anion	-840.63473	-840.63378	-840.69439
20(3) anion	-840.62836	-840.62742	-840.68896
20(4) anion	-840.62952	-840.62858	-840.68764
1-β-TS	-1112.86397	-1112.86302	-1112.94828
1-γ-TS	-1112.86643	-1112.86548	-1112.95096
1-δ-TS	-1112.86239	-1112.86144	-1112.94616
2-β-TS	-1112.85743	-1112.85649	-1112.94230
2-γ-TS	-1112.86517	-1112.86423	-1112.95197
2-δ-TS	-1112.85924	-1112.85829	-1112.94547
3-β-TS	-1111.70782	-1111.70688	-1111.78864
3-γ-TS	-1111.71415	-1111.71321	-1111.79619
3-δ-TS	-1111.70825	-1111.70731	-1111.79025
4-β-TS	-1150.98350	-1150.98255	-1151.06739
4-γ-TS	-1150.98401	-1150.98307	-1151.06944
4-δ-TS	-534.75811	-534.75717	-534.80951
5-β-TS	-534.10520	-534.10425	-534.15756
5-γ-TS	-534.11227	-534.11133	-534.16554
5-δ-TS	-534.10391	-534.10296	-534.15618
6-β-TS	-534.21117	-534.21022	-534.26252
6-γ-TS	-534.20393	-534.20299	-534.25450
6-δ-TS	-534.20724	-534.20630	-534.25788
7-β-TS	-578.76182	-578.76087	-578.81509
7-γ-TS	-578.14213	-578.14118	-578.19434
7-δ-TS	-578.29746	-578.29652	-578.34972
8-β-TS	-896.67764	-896.67670	-896.74993
8-γ-TS	-896.68222	-896.68127	-896.75414
8-δ-TS	-896.67630	-896.67536	-896.74683
9-β-TS	-975.18644	-975.185496	-975.264752
9-γ-TS	-975.19176	-975.19082	-975.27130
9-δ-TS	-975.18555	-975.18460	-975.26329
10-β-TS	-1131.11003	-1131.10909	-1131.19506
10-γ-TS	-1131.11093	-1131.10998	-1131.19844
10-δ-TS	-1131.10384	-1131.10290	-1131.18984
11-β-TS	-1129.92594	-1129.92500	-1130.00660
11-γ-TS	-1129.92667	-1129.92572	-1130.01015
11-δ-TS	-1129.92257	-1129.92163	-1130.00180
12-β-TS	-996.01718	-996.01624	-996.09875
12-γ-TS	-996.02304	-996.02210	-996.10393
12-δ-TS	-996.01675	-996.01581	-996.09696
13-β-TS	-1149.57050	-1149.56955	-1149.65746
13-γ-TS	-1149.57659	-1149.57564	-1149.66369
13-δ-TS	-1149.57037	-1149.56943	-1149.65632

Table S5. Standard absolute energies, enthalpies, and Gibbs free energies (in atomic units) at 298.15 K for all stationary points in PE at the M06-2X(SMD)/6-31++G(d,p) level of theory.

Species	E°	H°	G°
Leucine	-534.74952	-534.74857	-534.80209
Leucine-β-dmg	-534.09622	-534.09528	-534.15014
Leucine-γ-dmg	-534.10367	-534.10272	-534.15782
Leucine-δ-dmg	-534.09525	-534.09430	-534.14827
1	-578.76469	-578.76375	-578.81770
1-dmg	-578.14631	-578.14537	-578.19875
2	-578.76714	-578.76620	-578.81931
2-dmg	-578.14338	-578.14244	-578.19603
3	-577.61528	-577.61434	-577.66344
3-dmg	-576.99229	-576.99135	-577.04069
4	-616.88617	-616.88523	-616.93793
4-dmg	-616.26389	-616.26295	-616.31578
5	-656.15557	-656.15462	-656.21112
5-dmg	-655.53589	-655.53495	-655.59148
6	-656.15394	-656.15300	-656.20986
6-dmg	-655.53379	-655.53285	-655.58995
7	-695.42219	-695.42124	-695.48174
7-dmg	-694.80537	-694.80442	-694.86543
8	-362.57641	-362.57546	-362.61419
8-dmg	-361.95976	-361.95881	-361.99819
9	-441.08816	-441.08721	-441.13275
9-dmg	-440.47368	-440.47273	-440.51834
10	-597.00411	-597.00316	-597.05806
10-dmg	-596.39631	-596.39537	-596.45031
11	-595.82353	-595.82258	-595.87235
11-dmg	-595.21438	-595.21344	-595.26282
12	-461.92719	-461.92625	-461.97335
12-dmg	-461.30087	-461.29993	-461.34698
13	-615.48278	-615.48184	-615.53558
13-dmg	-614.85624	-614.85530	-614.90853
14	-763.94910	-763.94816	-764.00724
$14^{(1)}$-dmg	-763.32079	-763.31985	-763.37872
$14^{(2)}$-dmg	-763.33050	-763.32956	-763.38827
$14^{(3)}$-dmg	-763.32121	-763.32027	-763.37940
15	-999.33944	-999.33850	-999.41394
$15^{(1)}$-dmg	-998.71916	-998.71821	-998.79337
$15^{(2)}$-dmg	-998.72090	-998.71996	-998.79478
$15^{(3)}$-dmg	-998.72058	-998.71963	-998.79777
$15^{(4)}$-dmg	-998.71921	-998.71827	-998.79345
16	-574.27161	-574.27067	-574.31660

16$^{(1)}$-dmg	-573.63971	-573.63877	-573.68501
16$^{(2)}$-dmg	-573.63883	-573.63788	-573.68439
17	-457.64688	-457.64594	-457.68672
17$^{(1)}$-dmg	-457.03655	-457.03561	-457.07575
17$^{(2)}$-dmg	-457.02661	-457.02566	-457.06622
18	-685.41081	-685.40987	-685.46186
18$^{(1)}$-dmg	-684.78299	-684.78205	-684.83412
18$^{(2)}$-dmg	-684.79221	-684.79127	-684.84292
18$^{(3)}$-dmg	-684.78253	-684.78158	-684.83336
19	-765.88143	-765.88049	-765.94031
19$^{(1)}$-dmg	-765.25435	-765.25341	-765.31212
19$^{(2)}$-dmg	-765.24647	-765.24552	-765.30484
19$^{(3)}$-dmg	-765.24718	-765.24624	-765.30515
20	-841.07990	-841.07896	-841.14092
20$^{(1)}$-dmg	-840.45838	-840.45743	-840.51857
20$^{(2)}$-dmg	-840.46356	-840.46262	-840.52370
20$^{(3)}$-dmg	-840.44508	-840.44414	-840.50559
20$^{(4)}$-dmg	-840.44533	-840.44439	-840.50661
1-β-TS	-1112.86005	-1112.85911	-1112.94421
1-γ-TS	-1112.86159	-1112.86064	-1112.94666
1-δ-TS	-1112.85998	-1112.85903	-1112.94583
2-β-TS	-1112.85845	-1112.85750	-1112.94172
2-γ-TS	-1112.86014	-1112.85920	-1112.94697
2-δ-TS	-1112.85566	-1112.85472	-1112.94164
3-β-TS	-1111.70747	-1111.70652	-1111.78976
3-γ-TS	-1111.70895	-1111.70801	-1111.79179
3-δ-TS	-1111.70355	-1111.70261	-1111.78588
4-β-TS	-1150.97709	-1150.97614	-1151.06245
4-γ-TS	-1150.97961	-1150.97866	-1151.06695
4-δ-TS	-1150.97448	-1150.97354	-1151.06068
5-β-TS	-1190.24976	-1190.24881	-1190.33737
5-γ-TS	-1190.25082	-1190.24988	-1190.34086
5-δ-TS	-1190.24684	-1190.24590	-1190.33470
6-β-TS	-1190.24730	-1190.24636	-1190.33532
6-γ-TS	-1190.24883	-1190.24789	-1190.34025
6-δ-TS	-1190.24342	-1190.24248	-1190.33343
7-β-TS	-1229.51810	-1229.51715	-1229.61120
7-γ-TS	-1229.51940	-1229.51846	-1229.61293
7-δ-TS	-1229.51726	-1229.51631	-1229.61017
8-β-TS	-896.66744	-896.66650	-896.73952
8-γ-TS	-896.67140	-896.67045	-896.74439
8-δ-TS	-896.66617	-896.66522	-896.73966
9-β-TS	-975.17991	-975.17896	-975.25751
9-γ-TS	-975.18378	-975.18283	-975.26456

9-δ-TS	-975.17894	-975.17799	-975.25930
10-β-TS	-1131.10413	-1131.10319	-1131.19062
10-γ-TS	-1131.10454	-1131.10359	-1131.19085
10-δ-TS	-1131.10221	-1131.10127	-1131.18861
11-β-TS	-1129.91880	-1129.91785	-1130.00017
11-γ-TS	-1129.92042	-1129.91948	-1130.00423
11-δ-TS	-1129.91565	-1129.91471	-1129.99933
12-β-TS	-996.01572	-996.01477	-996.09570
12-γ-TS	-996.01842	-996.01747	-996.09977
12-δ-TS	-996.01273	-996.01178	-996.09302
13-β-TS	-1149.57150	-1149.57056	-1149.65796
13-γ-TS	-1149.57413	-1149.57319	-1149.66247
13-δ-TS	-1149.56829	-1149.56735	-1149.65566
$14^{(1)}$-δ-TS	-1298.04447	-1298.04353	-1298.13432
$14^{(2)}$-δ-TS	-1298.04423	-1298.04331	-1298.13434
$14^{(3)}$-δ-TS	-1298.04420	-1298.04325	-1298.13413
$15^{(1)}$-δ-TS	-1533.43419	-1533.43325	-1533.54183
$15^{(2)}$-δ-TS	-1533.43480	-1533.43385	-1533.54218
$15^{(3)}$-δ-TS	-1533.43492	-1533.43398	-1533.54148
$15^{(4)}$-δ-TS	-1533.43426	-1533.43332	-1533.54118
$16^{(1)}$-δ-TS	-1108.36532	-1108.36437	-1108.44047
$16^{(2)}$-δ-TS	-1108.36497	-1108.36403	-1108.43987
$17^{(1)}$-δ-TS	-991.74833	-991.74738	-991.82007
$17^{(2)}$-δ-TS	-991.74673	-991.74579	-991.81855
$18^{(1)}$-δ-TS	-1219.50605	-1219.50511	-1219.58909
$18^{(2)}$-δ-TS	-1219.50599	-1219.50505	-1219.58914
$18^{(3)}$-δ-TS	-1219.50627	-1219.50532	-1219.58889
$19^{(1)}$-δ-TS	-1299.96674	-1299.96579	-1300.06058
$19^{(2)}$-δ-TS	-1299.97187	-1299.97092	-1300.06041
$19^{(3)}$-δ-TS	-1299.97428	-1299.97333	-1300.06347
$20^{(1)}$-δ-TS	-1375.17477	-1375.17383	-1375.26674
$20^{(2)}$-δ-TS	-1375.17202	-1375.17108	-1375.26582
$20^{(3)}$-δ-TS	-1375.17281	-1375.17187	-1375.26344
$20^{(4)}$-δ-TS	-1375.17267	-1375.17173	-1375.26415

Table S6. Standard enthalpies of reaction (ΔH°) and activation (ΔH≠) in kcal/mol, imaginary vibrational frequencies (ν≠, in cm^{-1}), and tunnelling factors (κ) at 298.15 K in water at the M06-2X(SMD)/6-31++G(d,p) level of theory.

Transition State	ΔH°	ΔH≠	ν≠	κ
1-β-TS	-20.9	1.3	2278.1	3.3
1-γ-TS	-16.4	4.2	2051.9	18.5
1-δ-TS	-21.7	1.5	2124.8	3.7
2-β-TS	-18.1	6.9	2355.2	113.3
2-γ-TS	-13.7	6.5	1976.9	39.1
2-δ-TS	-18.9	4.9	2139.4	29.3
3-β-TS	-18.1	6.3	2174.2	58.3
3-γ-TS	-13.6	6.8	1981.4	44.3
3-δ-TS	-18.9	5.3	2119.0	32.5
4-β-TS	-18.9	2.7	2236.2	9.7
4-γ-TS	-14.4	6.8	2000.2	46.4
4-δ-TS	-19.7	5.0	2142.4	30.7
5-β-TS	-20.5	1.4	2246.7	3.3
5-γ-TS	-16.1	5.3	2040.0	29.0
5-δ-TS	-21.3	2.3	2128.0	6.9
6-β-TS	-20.3	1.6	2252.8	4.1
6-γ-TS	-15.9	5.3	2027.5	28.3
6-δ-TS	-21.1	4.2	2176.2	21.1
7-β-TS	-22.3	-0.1	2230.6	1.0
7-γ-TS	-17.9	3.8	2072.8	15.3
7-δ-TS	-23.1	2.5	2138.9	7.6
8-β-TS	-24.1	4.1	2346.9	25.6
8-γ-TS	-19.6	5.7	2137.0	40.7
8-δ-TS	-24.9	4.2	2224.1	22.0
9-β-TS	-25.0	4.5	2355.2	32.5
9-γ-TS	-20.5	5.6	2150.1	40.5
9-δ-TS	-25.8	4.3	2284.9	25.6
10-β-TS	-30.4	-2.9	2328.6	1.0
10-γ-TS	-25.9	1.0	2214.8	2.3
10-δ-TS	-31.2	0.2	2216.2	1.0
11-β-TS	-27.4	1.5	2461.3	4.0
11-γ-TS	-23.0	5.5	2234.9	44.4
11-δ-TS	-28.2	2.8	2455.2	12.0
12-β-TS	-16.7	7.2	2164.8	80.7
12-γ-TS	-12.2	7.9	1903.8	49.6
12-δ-TS	-17.5	6.6	2081.2	52.5
13-β-TS	-16.2	7.5	2182.3	96.1
13-γ-TS	-11.8	8.1	1900.0	51.4
13-δ-TS	-17.0	6.7	2091.3	56.5

Table S7. Standard enthalpies of reaction (ΔH°) and activation (ΔH≠) in kcal/mol, imaginary vibrational frequencies (ν≠, in cm^{-1}), and tunnelling factors (κ) at 298.15 K in PE at the M06-2X(SMD)/6-31++G(d,p) level of theory.

Transition State	ΔH°	ΔH≠	ν≠	κ
1-β-TS	-21.9	-0.1	2228.4	1.0
1-γ-TS	-17.2	3.7	2012.6	13.5
1-δ-TS	-22.5	-0.6	2063.0	1.0
2-β-TS	-18.5	2.5	2086.3	7.6
2-γ-TS	-13.9	6.1	1969.8	33.9
2-δ-TS	-19.2	3.6	2073.3	14.2
3-β-TS	-19.0	1.9	2098.4	5.2
3-γ-TS	-14.4	5.7	1935.1	26.9
3-δ-TS	-19.6	3.8	2072.7	15.2
4-β-TS	-19.5	2.7	2107.8	9.0
4-γ-TS	-14.8	5.8	1968.8	30.6
4-δ-TS	-20.1	3.8	2089.9	15.4
5-β-TS	-21.1	0.7	2134.9	1.5
5-γ-TS	-16.4	4.7	2006.0	21.1
5-δ-TS	-21.7	1.9	2149.3	5.1
6-β-TS	-20.8	1.2	2125.9	2.8
6-γ-TS	-16.1	4.9	1996.4	22.7
6-δ-TS	-21.4	3.0	2098.3	10.4
7-β-TS	-22.9	-0.4	2119.4	1.0
7-γ-TS	-18.2	3.5	2036.3	12.5
7-δ-TS	-23.5	-0.5	2058.0	1.0
8-β-TS	-23.0	2.7	2180.1	8.9
8-γ-TS	-18.3	4.9	2025.4	23.1
8-δ-TS	-23.6	2.9	2114.4	9.4
9-β-TS	-24.4	2.2	2204.0	6.6
9-γ-TS	-19.7	4.5	2093.3	21.7
9-δ-TS	-25.0	2.2	2157.6	6.4
10-β-TS	-28.6	-3.0	2163.0	1.0
10-γ-TS	-23.9	1.4	2132.1	3.4
10-δ-TS	-29.2	-2.4	2079.9	1.0
11-β-TS	-27.7	0.0	2195.5	1.0
11-γ-TS	-23.0	3.7	2171.9	15.9
11-δ-TS	-28.3	1.4	2193.7	3.2
12-β-TS	-16.9	4.2	2071.5	19.3
12-γ-TS	-12.3	7.2	1883.7	38.4
12-δ-TS	-17.5	5.5	2037.9	30.9
13-β-TS	-16.8	4.1	2073.5	18.3
13-γ-TS	-12.1	7.1	1881.4	37.4

13-δ-TS	-17.4	5.5	2044.2	31.6
14(1)-δ-TS	-16.29	-0.67	1960.7	1.00
14(2)-δ-TS	-22.38	-0.53	1982.4	1.00
14(3)-δ-TS	-16.55	-0.50	1968.3	1.00
15(1)-δ-TS	-21.33	-0.28	2042.6	1.00
15(2)-δ-TS	-22.42	-0.66	2042.2	1.00
15(3)-δ-TS	-22.22	-0.74	2042.2	1.00
15(4)-δ-TS	-21.36	-0.33	2043.0	1.00
16(1)-δ-TS	-14.04	0.37	2034.2	1.05
16(2)-δ-TS	-13.48	0.59	2036.3	1.44
17(1)-δ-TS	-27.57	-4.48	1994.0	1.00
17(2)-δ-TS	-21.33	-0.65	2003.8	1.00
18(1)-δ-TS	-16.60	-0.59	1963.6	1.00
18(2)-δ-TS	-22.39	-0.55	1980.6	1.00
18(3)-δ-TS	-16.31	-0.72	1958.4	1.00
19(1)-δ-TS	-17.06	5.65	2027.7	32.15
19(2)-δ-TS	-12.11	0.96	1978.3	2.24
19(3)-δ-TS	-12.56	0.92	1983.3	2.14
20(1)-δ-TS	-20.55	-0.36	2051.0	1.00
20(2)-δ-TS	-23.80	1.37	2073.2	3.16
20(3)-δ-TS	-12.21	0.87	1978.26	1.00
20(4)-δ-TS	-12.36	0.96	1984.7	2.24

Table S8. Standard Gibbs free energies of reaction (ΔG°_{SET}) and activation ($\Delta G^{\neq}_{SET}$), and rate constants (k_{SET}) for the SET reactions studied.[a]

Reactants	ΔG°_{SET} (kcal/mol)	$\Delta G^{\neq}_{SET}$ (kcal/mol)	k_{SET} ($M^{-1}s^{-1}$)	ΔIP (kcal/mol) [b]
Leucine-β-dmg + 1	31.6	35.8	$1.69 \cdot 10^{-17}$	
Leucine-γ-dmg + 1	41.7	44.9	$4.15 \cdot 10^{-24}$	-23.0
Leucine-δ-dmg + 1	33.7	36.7	$4.37 \cdot 10^{-18}$	
Leucine-β-dmg + 2	33.4	44.7	$4.92 \cdot 10^{-24}$	
Leucine-γ-dmg + 2	43.4	50.6	$2.48 \cdot 10^{-28}$	-23.4
Leucine-δ-dmg + 2	35.4	43.4	$4.66 \cdot 10^{-23}$	
Leucine-β-dmg + 3	33.6	44.1	$1.57 \cdot 10^{-23}$	
Leucine-γ-dmg + 3	43.6	50.4	$3.65 \cdot 10^{-28}$	-27.4
Leucine-δ-dmg + 3	35.6	43.1	$8.74 \cdot 10^{-23}$	
Leucine-β-dmg + 4	29.8	34.1	$2.05 \cdot 10^{-16}$	
Leucine-γ-dmg + 4	46.4	60.5	$9.42 \cdot 10^{-36}$	-30.5
Leucine-δ-dmg + 4	34.5	41.2	$1.29 \cdot 10^{-21}$	
Leucine-β-dmg + 5	30.0	37.4	$4.50 \cdot 10^{-19}$	
Leucine-γ-dmg + 5	40.0	44.9	$1.41 \cdot 10^{-24}$	-33.6
Leucine-δ-dmg + 5	32.0	37.2	$6.68 \cdot 10^{-19}$	
Leucine-β-dmg + 6	27.3	30.0	$1.22 \cdot 10^{-13}$	
Leucine-γ-dmg + 6	43.9	54.9	$6.38 \cdot 10^{-32}$	-32.9
Leucine-δ-dmg + 6	32.0	36.6	$1.66 \cdot 10^{-18}$	
Leucine-β-dmg + 7	27.4	31.7	$4.11 \cdot 10^{-15}$	
Leucine-γ-dmg + 7	37.4	40.5	$1.53 \cdot 10^{-21}$	-36.1
Leucine-δ-dmg + 7	29.4	32.4	$1.33 \cdot 10^{-15}$	
Leucine-β-dmg + 8	24.4	25.2	$2.45 \cdot 10^{-9}$	
Leucine-γ-dmg + 8	41.0	47.5	$1.18 \cdot 10^{-25}$	-29.1
Leucine-δ-dmg + 8	29.1	31.1	$1.28 \cdot 10^{-13}$	
Leucine-β-dmg + 9	23.2	23.3	$3.14 \cdot 10^{-8}$	
Leucine-γ-dmg + 9	39.8	42.9	$1.25 \cdot 10^{-22}$	-37.5
Leucine-δ-dmg + 9	27.9	28.4	$5.54 \cdot 10^{-12}$	
Leucine-β-dmg + 10	19.0	19.0	$5.75 \cdot 10^{-6}$	
Leucine-γ-dmg + 10	29.0	29.1	$2.39 \cdot 10^{-13}$	-46.9
Leucine-δ-dmg + 10	21.0	21.0	$1.86 \cdot 10^{-7}$	
Leucine-β-dmg + 11	22.7	23.2	$1.36 \cdot 10^{-8}$	
Leucine-γ-dmg + 11	32.7	33.3	$6.03 \cdot 10^{-16}$	-48.7
Leucine-δ-dmg + 11	24.7	25.0	$6.53 \cdot 10^{-10}$	
Leucine-β-dmg + 12	36.8	51.3	$2.79 \cdot 10^{-27}$	
Leucine-γ-dmg + 12	53.4	83.6	$5.08 \cdot 10^{-51}$	-27.8
Leucine-δ-dmg + 12	41.5	60.3	$6.57 \cdot 10^{-34}$	
Leucine-β-dmg + 13	36.6	50.6	$6.58 \cdot 10^{-27}$	
Leucine-γ-dmg + 13	53.2	82.7	$1.80 \cdot 10^{-50}$	-32.5
Leucine-δ-dmg + 13	41.3	59.6	$1.78 \cdot 10^{-33}$	

Leucine-δ-dmg + 14$^{(1)}$	47.1	65.2	$3.39 \cdot 10^{-36}$	
Leucine-δ-dmg + 14$^{(2)}$	46.8	64.7		-7.8
Leucine-δ-dmg + 14$^{(3)}$	47.6	67.5		
Leucine-δ-dmg + 15$^{(1)}$	40.3	52.4		
Leucine-δ-dmg + 15$^{(2)}$	39.1	51.2	$3.72 \cdot 10^{-27}$	-15.1
Leucine-δ-dmg + 15$^{(3)}$	38.7	49.3		
Leucine-δ-dmg + 15$^{(4)}$	39.6	50.2		
Leucine-δ-dmg + 16$^{(1)}$	43.4			-18.6
Leucine-δ-dmg + 16$^{(2)}$	44.3	57.8	$1.70 \cdot 10^{-32}$ [c]	
Leucine-δ-dmg + 17$^{(1)}$	40.6	47.4	$2.25 \cdot 10^{-23}$ [c]	-10.9
Leucine-δ-dmg + 17$^{(2)}$	43.8			
Leucine-δ-dmg + 18$^{(1)}$	47.4			
Leucine-δ-dmg + 18$^{(2)}$	47.4	62.8	$4.80 \cdot 10^{-33}$ [c]	-7.2
Leucine-δ-dmg + 18$^{(3)}$	48.2			
Leucine-δ-dmg + 19$^{(1)}$	41.8	60.7		
Leucine-δ-dmg + 19$^{(2)}$	47.3	74.1		-33.1
Leucine-δ-dmg + 19$^{(3)}$	47.7	77.2	$1.74 \cdot 10^{-46}$	
Leucine-δ-dmg + 20$^{(1)}$	41.6			
Leucine-δ-dmg + 20$^{(2)}$	39.6	48.8	$1.77 \cdot 10^{-24}$ [c]	-35.2
Leucine-δ-dmg + 20$^{(3)}$	46.2			
Leucine-δ-dmg + 20$^{(4)}$	44.7			

[a] Rate constants are multiplied by the anionic molar fractions at pH 7.4 using pK_a predictions from Ref 57; [b] Calculated ionization potential change (relative to the value of phenol) in the gas phase reported in Ref 37; [c] Using the most stable anionic form for pK_a predictions, see Table S9.

Table S9. Standard Gibbs free energies (in atomic units) for neutral and anionic forms at the M06-2X(PCM)/6-311++G(d,p) level of theory in water, molar fractions for monoanionic forms, and predicted pK_a values for the polyphenolic antioxidant species.

Species	G° (neutral)	G° (anionic)	Molar fraction (M)	pK_a [a]
14$^{(1)}$	-764.19445	-763.73993	0.36	7.85
15$^{(2)}$	-999.63805	-999.17623	0.02	9.10
16$^{(2)}$	-574.44984	-573.98499	$6.03 \cdot 10^{-3}$	9.62
17$^{(1)}$	-457.80125	-457.34949	1.06	7.38
18$^{(2)}$	-685.63605	-685.18948	8.21	6.49
19$^{(3)}$	-766.10932	-765.64631	0.01	9.31
20$^{(2)}$	-841.33471	-840.87812	0.16	8.20

[a] Microscopic pK_a values predicted using the correlation equations at the M06-2X(PCM)/6-311++G(d,p) level of theory reported in Ref 57 for the most stable anionic species of each polyphenolic antioxidant.

Cartesian coordinates of the optimized antioxidant species studied at the M06-2X(SMD)/6-31++G(d,p) level of theory in water.

Leucine

Charge=0, Multiplicity=1

N	0.53156000	2.07729200	-0.30267800
H	0.24801400	2.00738900	-1.27068100
H	0.67127800	2.99370400	0.10398200
C	0.72093600	0.98587100	0.44625700
O	1.09558300	1.03414400	1.62522800
C	0.39157800	-0.35190800	-0.22299800
H	0.36021000	-0.20782600	-1.30797600
N	1.42243000	-1.32716100	0.09058300
H	1.17577800	-2.15041100	0.62798600
C	2.70997400	-1.11089500	-0.19835800
O	3.11800200	-0.10016200	-0.78432500
H	3.38643000	-1.91246400	0.12489300
C	-0.95222900	-0.87829500	0.29572100
H	-0.84850500	-1.04995700	1.37485600
C	-2.16265900	0.02486900	0.03839600
H	-1.97685100	1.00307000	0.50287900
C	-3.39267500	-0.58902800	0.70784100
H	-3.61651100	-1.56895100	0.26916900
H	-4.27042200	0.05008500	0.57069600
H	-3.23436300	-0.72681100	1.78228600
C	-2.42343500	0.22964200	-1.45480100
H	-1.60583300	0.75322100	-1.95991100
H	-3.33438000	0.81748500	-1.60616000
H	-2.56070700	-0.73928200	-1.95044800
H	-1.12968900	-1.85355800	-0.17648100

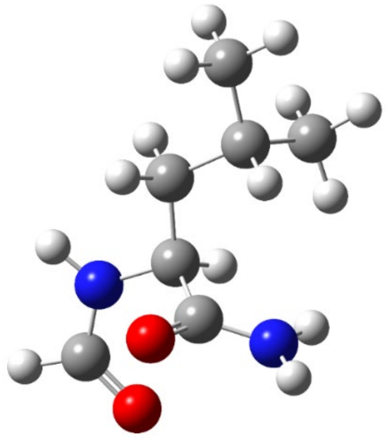

Leucine-β-dmg

Charge=0, Multiplicity=2

N	-0.49386300	2.09303700	0.44595800
H	-0.00214300	1.93581900	1.31592900
H	-0.69924200	3.04288200	0.16231600
C	-0.84352700	1.07447400	-0.34778500
O	-1.45774900	1.22011000	-1.41040800
C	-0.35163300	-0.31154900	0.11700900
H	-0.26542500	-0.29018200	1.20968400
N	-1.30745700	-1.33286200	-0.26537600
H	-1.02665100	-2.05829000	-0.91456300
C	-2.58021900	-1.28005500	0.14237200
O	-3.01912400	-0.39925100	0.89228900
H	-3.21091800	-2.09331600	-0.23872800
C	0.97499300	-0.57628000	-0.52377800
H	0.94421800	-0.96518500	-1.54031100
C	2.24320400	0.05040100	-0.03497300
H	2.21498700	1.12807000	-0.27575400
C	3.44063300	-0.55935600	-0.76781800
H	3.53063900	-1.62481700	-0.52717800
H	4.36850300	-0.06249600	-0.46986200
H	3.33182400	-0.46243200	-1.85244100
C	2.41929200	-0.07961900	1.48300000

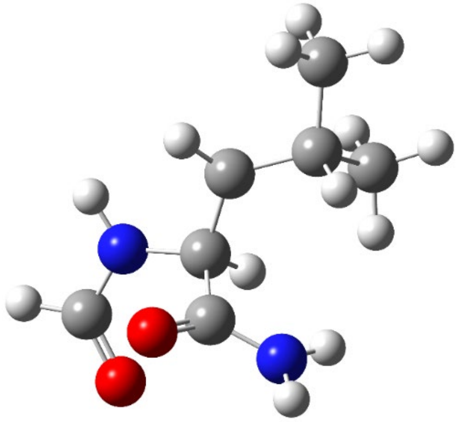

H	1.63723800	0.44387600	2.04077500
H	3.38195400	0.34477900	1.78372500
H	2.40278100	-1.13488800	1.77913000

Leucine-γ-dmg

Charge=0, Multiplicity=2

N	0.00085000	1.68120700	1.00464700
H	0.24604200	1.21647600	1.86880900
H	0.11129800	2.68523700	0.93727200
C	-0.40276100	0.98731600	-0.06460800
O	-0.70907400	1.51895600	-1.14005700
C	-0.40360700	-0.53423100	0.09435700
H	-0.35537300	-0.78140200	1.16080600
N	-1.60953700	-1.10399200	-0.47723900
H	-1.52982000	-1.74735900	-1.25645000
C	-2.82862100	-0.71995600	-0.08316700
O	-3.02584800	0.10901100	0.81394600
H	-3.65187700	-1.20626900	-0.62192200
C	0.82391500	-1.12523200	-0.63546600
H	0.70023900	-0.92924400	-1.70749300
H	0.78114100	-2.21174200	-0.48035300
C	2.13112500	-0.58113800	-0.15142500
C	2.76467500	0.57633600	-0.85270900
H	3.85580700	0.55007300	-0.74789700
H	2.43601300	1.54222100	-0.43240500
H	2.51749400	0.58708300	-1.91896200
C	2.57377000	-0.90599700	1.23799000
H	2.21070900	-1.88842700	1.55831700
H	3.66690400	-0.89498500	1.31834900
H	2.20063000	-0.16849200	1.96912900

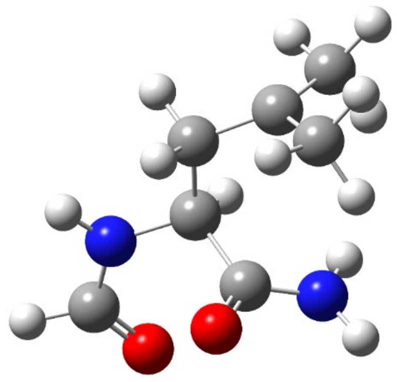

Leucine-δ-dmg

Charge=0, Multiplicity=2

N	-0.39018200	2.06556900	0.27545000
H	-0.01813100	1.98639100	1.21329800
H	-0.48647200	2.98357800	-0.14025300
C	-0.66330400	0.97756000	-0.45299400
O	-1.09310500	1.03109400	-1.61262400
C	-0.36700200	-0.36431000	0.22364200
H	-0.33544400	-0.20800900	1.30738600
N	-1.41553900	-1.31935600	-0.08513000
H	-1.18895300	-2.14656300	-0.62513000
C	-2.69725700	-1.07478200	0.20845100
O	-3.08018900	-0.05549000	0.79642900
H	-3.39258200	-1.86113900	-0.11180600
C	0.97480900	-0.92195200	-0.26578300
H	0.89031500	-1.14277600	-1.33691700
H	1.14832500	-1.87093100	0.25753300
C	2.17058900	0.00748000	-0.02784100
H	2.03060200	0.92368300	-0.61716300
C	3.45294200	-0.67421000	-0.53873400
H	3.64014200	-1.59856900	0.01846800
H	4.31738100	-0.01648100	-0.41134800
H	3.36129200	-0.92315000	-1.60154200
C	2.31003300	0.37456000	1.41411100

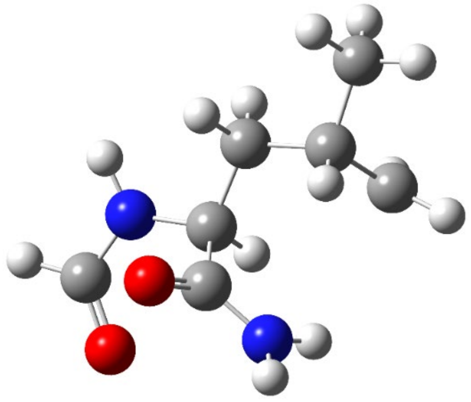

H	2.13657400	-0.37790700	2.17894100
H	2.83849300	1.27747300	1.70073900

Leucine-β-anion

Charge=-1, Multiplicity=1

N	-0.42884900	2.05265200	0.41445900
H	-0.00830700	1.90151700	1.32117500
H	-0.56977600	3.00078100	0.09065700
C	-0.72563600	1.02224800	-0.39177900
O	-1.23960500	1.19434000	-1.50890900
C	-0.35892800	-0.36549500	0.13771700
H	-0.36074000	-0.28988400	1.24111900
N	-1.41630900	-1.30796600	-0.21250100
H	-1.17853100	-2.03442100	-0.87920300
C	-2.69051400	-1.14132200	0.13587200
O	-3.10302800	-0.20856600	0.84596100
H	-3.37074200	-1.91059700	-0.25393100
C	0.95354300	-0.86034500	-0.48329100
C	2.16386100	-0.01328000	-0.07165800
H	2.01293300	1.02273000	-0.41667400
C	3.43215700	-0.53110400	-0.75696000
H	3.60649300	-1.58015200	-0.48215300
H	4.31985700	0.04056600	-0.46020000
H	3.34110100	-0.48555900	-1.84717200
C	2.43196400	0.04701600	1.44560800
H	1.56778600	0.41028200	2.01283700
H	3.28033800	0.70061500	1.68668500
H	2.66771700	-0.95847100	1.81982300
H	1.11035500	-1.86270800	-0.03613700

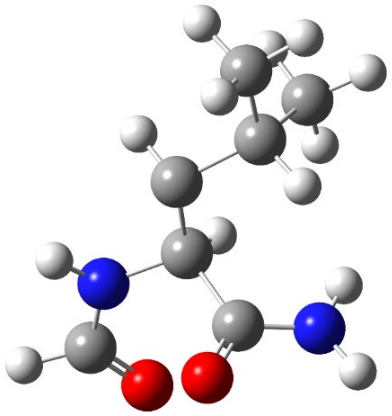

Leucine-γ-anion

Charge=-1, Multiplicity=1

N	0.64971500	2.09626800	-0.32169600
H	0.28964200	2.03414400	-1.26419600
H	0.78639700	3.01063400	0.08986600
C	0.72710200	1.00783100	0.45406200
O	1.09168800	1.05635800	1.63818900
C	0.33917300	-0.31719300	-0.19940100
H	0.30638100	-0.17753600	-1.28522600
N	1.34072600	-1.32894700	0.10840500
H	1.05848400	-2.14352800	0.64153700
C	2.63090700	-1.18587400	-0.20233400
O	3.08981300	-0.20485000	-0.80461200
H	3.26762400	-2.02081800	0.11866800
C	-1.02577500	-0.79382900	0.32035200
H	-0.90083100	-1.01164900	1.39213100
C	-2.20143000	0.13672300	0.09210600
C	-3.42011100	-0.57062400	0.66500300
H	-3.59562800	-1.56535600	0.17807000
H	-4.34501800	0.00403500	0.52288200
H	-3.32853600	-0.77032600	1.74150800
C	-2.42380100	0.27786100	-1.40670900
H	-1.61667200	0.80936500	-1.92953100
H	-3.35049800	0.82275400	-1.63153500
H	-2.51781500	-0.71964900	-1.91055700

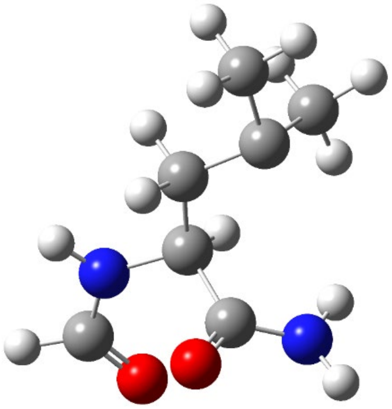

H	-1.19500400	-1.78475000	-0.17768100

Leucine-δ-anion

Charge=-1, Multiplicity=1

N	0.39536600	2.07752200	-0.26376900
H	-0.05295300	2.00084100	-1.16758900
H	0.49613400	2.99187200	0.15858700
C	0.68563500	0.98584900	0.45334900
O	1.16628000	1.04122800	1.59416000
C	0.35785000	-0.35470200	-0.20930000
H	0.30938700	-0.21228900	-1.29400700
N	1.40200800	-1.31999800	0.09119700
H	1.16963800	-2.14508000	0.63176600
C	2.68155500	-1.09814000	-0.22288300
O	3.07459400	-0.08806700	-0.82206400
H	3.36911700	-1.89437400	0.09032000
C	-0.98381600	-0.88378000	0.30842100
H	-0.89662500	-1.01615800	1.39666300
C	-2.19197600	-0.00139800	-0.03062200
H	-2.03237600	0.95627500	0.50882400
C	-3.42902300	-0.63639300	0.60968600
H	-3.60647200	-1.63334100	0.18632500
H	-4.32067900	-0.02982300	0.41545300
H	-3.32059900	-0.73891600	1.69654700
C	-2.39008500	0.17442200	-1.53842300
H	-1.56875100	0.76923300	-1.97573000
H	-3.29801200	0.78365500	-1.69668900
H	-1.13725700	-1.87499600	-0.14059800

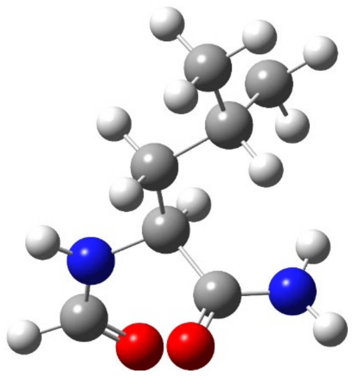

1

Charge=0, Multiplicity=1

C	-1.96160700	1.39812500	-0.20291600
C	-1.91229700	0.01700400	-0.32717100
C	-0.69625900	-0.65940300	-0.25685300
C	0.51849400	0.01022300	-0.06691100
C	0.44874400	1.41483000	0.05342900
C	-0.76972100	2.09152600	-0.01398900
H	-2.91169500	1.92059000	-0.25775400
H	-0.72035700	-1.73861700	-0.36057000
H	-0.77531900	3.17420000	0.08146900
O	1.60950400	2.12750100	0.23799300
H	1.40171500	3.07121600	0.29935700
O	-3.08073300	-0.70266000	-0.54724800
C	-3.69612500	-1.14138200	0.66684100
H	-4.59643800	-1.68925400	0.38615000
H	-3.02027000	-1.80092700	1.22213300
H	-3.96567300	-0.28204800	1.29059000
C	1.84964900	-0.74984600	0.00067700
C	1.64503800	-2.26191700	-0.15511100
H	1.01287000	-2.67231600	0.63940600
H	1.19787200	-2.51614400	-1.12179000
H	2.61995600	-2.75587700	-0.09657300
C	2.77929100	-0.29149900	-1.13748600
H	3.02185000	0.77034100	-1.06808600
H	3.71470900	-0.86054800	-1.09541000

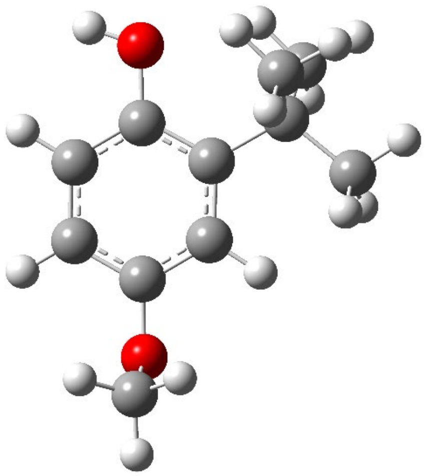

H	2.31236600	-0.47798300	-2.11082100
C	2.52523100	-0.51593900	1.36402700
H	3.46022000	-1.08542300	1.40925900
H	2.75808800	0.53678900	1.53306900
H	1.87730500	-0.86305800	2.17637700

1-dmg

Charge=0, Multiplicity=2

C	1.89360000	1.65894500	-0.00006100
C	1.94192700	0.24165400	0.00003600
C	0.75682200	-0.52674500	0.00009500
C	-0.49054600	0.06604600	0.00005500
C	-0.56355600	1.53092400	0.00004100
C	0.68008900	2.27708700	-0.00003700
H	2.82594000	2.21430600	-0.00014700
H	0.84116900	-1.60515100	0.00015500
H	0.59998100	3.35983400	-0.00009400
O	-1.65887000	2.15820500	0.00009200
O	3.17064700	-0.29031100	-0.00006000
C	3.31682500	-1.71517700	0.00006700
H	2.86489300	-2.14366400	0.89799000
H	2.86428200	-2.14389400	-0.89743800
H	4.38980600	-1.89634200	-0.00031400
C	-1.77083900	-0.76538500	-0.00001200
C	-1.47400800	-2.26956500	-0.00004800
H	-0.91260900	-2.57480400	-0.88955100
H	-0.91273500	-2.57488400	0.88950200
H	-2.42320300	-2.81402600	-0.00013900
C	-2.59562000	-0.45207700	1.26313900
H	-2.89754300	0.59564300	1.30262900
H	-3.49728100	-1.07416400	1.26678600
H	-2.01910200	-0.68478600	2.16507700
C	-2.59544900	-0.45204100	-1.26324800
H	-3.49741900	-1.07368300	-1.26668800
H	-2.89683100	0.59581500	-1.30307600
H	-2.01902600	-0.68534700	-2.16510100

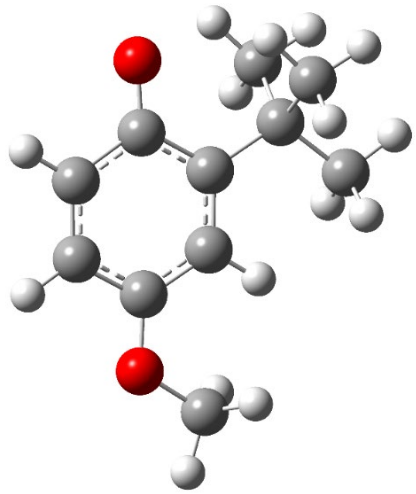

1 anion

Charge=-1, Multiplicity=1

C	-1.96036900	1.40975300	-0.19545500
C	-1.90137000	0.02897700	-0.33047300
C	-0.67059200	-0.62317200	-0.26465200
C	0.53436200	0.05992300	-0.06778900
C	0.49912500	1.49507500	0.07339300
C	-0.77846900	2.11782700	-0.00092500
H	-2.91907500	1.92071600	-0.24575300
H	-0.67984600	-1.70336900	-0.37646900
H	-0.80777800	3.20034700	0.10127200
O	1.56866400	2.22755100	0.26132100
O	-3.06701700	-0.71114500	-0.54998000
C	-3.65257000	-1.18951800	0.66024000
H	-4.54804100	-1.75003800	0.38578500
H	-2.95633700	-1.84853900	1.19156100
H	-3.92868800	-0.35191100	1.31108300
C	1.86369000	-0.70439500	-0.00113700

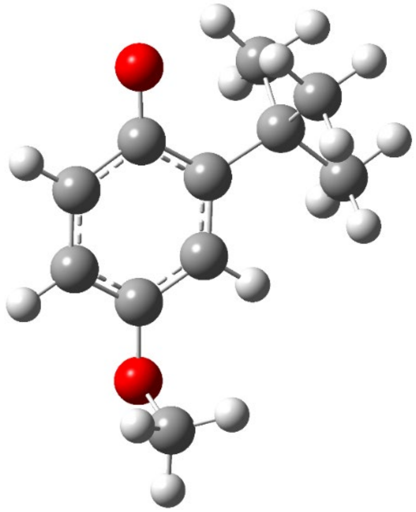

C	1.67756200	-2.21570600	-0.19120600
H	1.03981800	-2.65133200	0.58557900
H	1.24323200	-2.45490900	-1.16807700
H	2.65607000	-2.70408400	-0.13184000
C	2.81397600	-0.22406800	-1.11306600
H	3.03920200	0.83944100	-1.01848000
H	3.75542300	-0.78441800	-1.06640800
H	2.36653800	-0.39800400	-2.09852500
C	2.51984400	-0.49956700	1.37633200
H	3.48032700	-1.02730400	1.41617600
H	2.69620600	0.55711500	1.58470700
H	1.87864200	-0.90573500	2.16710200

2

Charge=0, Multiplicity=1

C	-1.27019900	1.65986400	0.00001500
C	-0.02142100	1.03650700	0.00001600
C	0.08201900	-0.37610200	-0.00000500
C	-1.11340700	-1.10018300	-0.00002700
C	-2.36075900	-0.47325000	-0.00002800
C	-2.44601900	0.90914800	-0.00000700
H	-1.34656900	2.74016800	0.00003300
H	-3.41512200	1.39760700	-0.00000800
O	-3.53593400	-1.19712800	-0.00005000
H	-3.33512100	-2.14376400	-0.00007300
O	1.14661400	1.75588700	0.00003900
C	1.05819300	3.17396400	0.00005600
H	0.54313500	3.53789100	0.89547300
H	2.08630500	3.53538700	0.00006700
H	0.54314700	3.53791200	-0.89536000
H	-1.09588500	-2.18506800	-0.00004400
C	1.43964900	-1.09462500	-0.00000400
C	1.27576000	-2.62026200	-0.00004600
H	0.74416500	-2.97506700	0.88913800
H	0.74418700	-2.97502000	-0.88926200
H	2.26981100	-3.07824700	-0.00004500
C	2.23830400	-0.72374200	-1.26317300
H	2.45352900	0.34458100	-1.31503500
H	3.18982400	-1.26712500	-1.26412700
H	1.68381900	-1.01081400	-2.16346400
C	2.23826500	-0.72380600	1.26320900
H	3.18980000	-1.26716200	1.26415100
H	2.45345900	0.34452000	1.31514600
H	1.68376600	-1.01095200	2.16346700

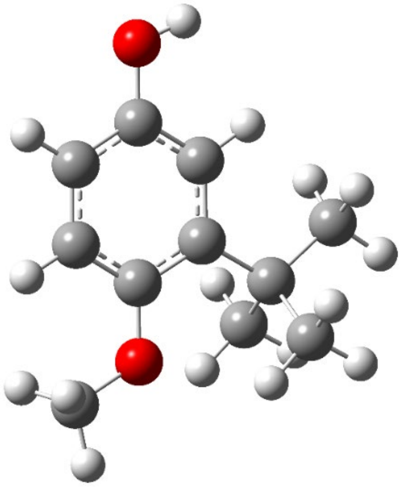

2-dmg

Charge=0, Multiplicity=2

C	1.53307800	1.47893000	0.00001500
C	0.19891800	0.99834400	0.00006600
C	-0.09539400	-0.41418500	0.00007900
C	0.97984800	-1.26505300	0.00003100
C	2.35580200	-0.81567000	0.00001000
C	2.58759200	0.60620400	-0.00003300
H	1.72019700	2.54569300	-0.00004400
H	0.84204800	-2.34021700	0.00001700
H	3.61243300	0.96361900	-0.00010100
O	3.31182900	-1.64477000	0.00005500
O	-0.84299700	1.82806400	0.00012200
C	-0.63240500	3.24451900	-0.00006900
H	-1.62896800	3.68182400	-0.00012400
H	-0.08953600	3.54854900	0.89822100
H	-0.08956100	3.54826800	-0.89848100
C	-1.53982900	-0.92998200	-0.00000200
C	-1.57893600	-2.46322400	0.00042500
H	-1.09801300	-2.88359300	0.88962700
H	-2.62470900	-2.78467400	0.00054800
H	-1.09808300	-2.88409700	-0.88857400
C	-2.27716900	-0.45219300	-1.26495200
H	-2.36264300	0.63449200	-1.31128300
H	-1.75844200	-0.80022000	-2.16475800
H	-3.28744600	-0.87533100	-1.27105100
C	-2.27772400	-0.45143500	1.26436700
H	-2.36357400	0.63525300	1.30982200
H	-3.28786100	-0.87489500	1.27052600
H	-1.75917700	-0.79856600	2.16462300

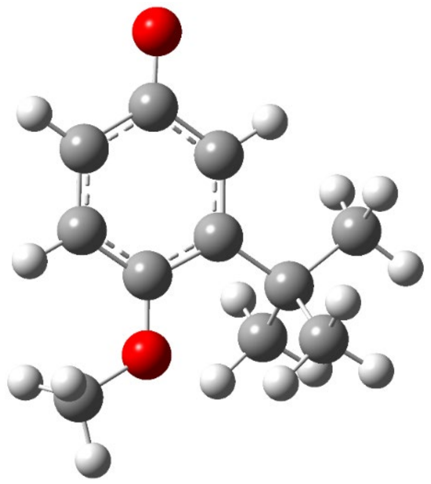

2 anion

Charge=-1, Multiplicity=1

C	-1.40854600	1.54728700	0.00001600
C	-0.11890300	1.01756200	0.00001900
C	0.06949900	-0.38622900	-0.00000600
C	-1.07642900	-1.18471000	-0.00003200
C	-2.40830000	-0.68463900	-0.00003600
C	-2.53436500	0.71940200	-0.00001000
H	-1.55721400	2.62158500	0.00003500
H	-3.52716200	1.16317100	-0.00001100
O	-3.44780200	-1.49911900	-0.00006500
O	1.00853900	1.82227400	0.00004500
C	0.81248900	3.22555400	0.00006900
H	0.26982200	3.55303900	0.89435900
H	1.80895200	3.66861100	0.00008800
H	0.26984000	3.55307200	-0.89421900
H	-0.97620400	-2.26529200	-0.00005200
C	1.47245400	-1.01679500	-0.00000500
C	1.41414000	-2.55041700	-0.00003600
H	0.90690700	-2.94117900	0.88808400
H	0.90692300	-2.94114300	-0.88818300
H	2.43721800	-2.94035700	-0.00003400
C	2.24738300	-0.59638300	-1.26240500
H	2.38953300	0.48410500	-1.31657600

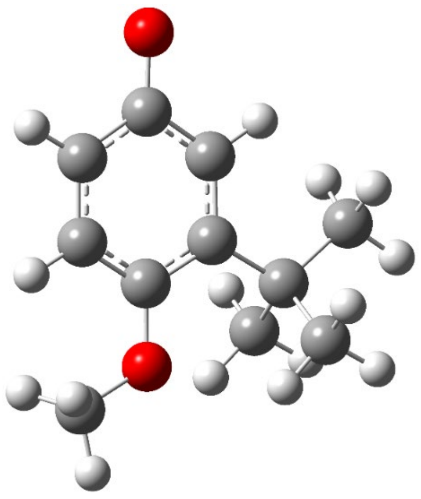

H	3.23404400	-1.07391800	-1.26427100
H	1.71363400	-0.92106600	-2.16274800
C	2.24735900	-0.59643300	1.26242600
H	3.23402100	-1.07396500	1.26429100
H	2.38950400	0.48405300	1.31664500
H	1.71359300	-0.92115500	2.16274600

3

Charge=0, Multiplicity=1

C	0.12277900	-0.63934300	-0.15460800
C	0.47757200	0.71469800	-0.09786800
C	2.81092800	0.05660700	0.03035900
O	4.12784100	0.45921300	0.11950100
H	4.70632000	-0.31696900	0.11234800
C	-0.58329300	1.78775300	-0.13714100
H	-0.70970000	2.21036600	0.86730000
H	-0.25142700	2.60774700	-0.78110200
C	-1.90632600	1.21523600	-0.63756200
H	-1.85303500	1.03655700	-1.71824900
H	-2.72639600	1.91611600	-0.45506500
C	-2.22768800	-0.11608300	0.04281700
C	-3.49842000	-0.72419600	-0.52549000
H	-3.67231300	-1.71514300	-0.09515700
H	-4.35356500	-0.08629300	-0.28476300
H	-3.42620200	-0.81779800	-1.61324100
C	-2.31636600	0.00991200	1.56134500
H	-3.06811800	0.75834000	1.83028900
H	-2.60674300	-0.95033800	1.99782800
H	-1.35965500	0.31094800	1.99827100
O	-1.18117500	-1.06895900	-0.28050700
C	1.10457000	-1.63117100	-0.12069100
H	0.80200000	-2.67285200	-0.16830600
C	2.44968300	-1.28989300	-0.03059900
H	3.21560300	-2.06018200	-0.00636700
C	1.83321600	1.04445400	-0.00285100
H	2.12997000	2.08962500	0.03800000

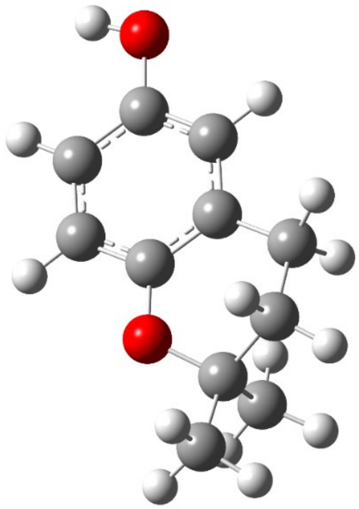

3-dmg

Charge=0, Multiplicity=2

C	-0.18332000	0.62823800	-0.08879200
C	-0.53532900	-0.75352100	-0.05599600
C	-2.91048100	-0.07788100	0.02693000
O	-4.13213100	-0.40100700	0.08550600
C	0.54971100	-1.79912800	-0.10723700
H	0.72451600	-2.19278600	0.90057500
H	0.21295700	-2.63816400	-0.72178500
C	1.83227900	-1.19694400	-0.67194000
H	1.71144800	-0.99405900	-1.74257600
H	2.67109900	-1.88847700	-0.55432200
C	2.18614700	0.11348300	0.02478100
C	3.37338800	0.78983400	-0.63515000
H	3.55986400	1.76552100	-0.17743800
H	4.26289900	0.16766500	-0.50380300
H	3.19338200	0.92446700	-1.70546200
C	2.40155000	-0.04594500	1.52512200

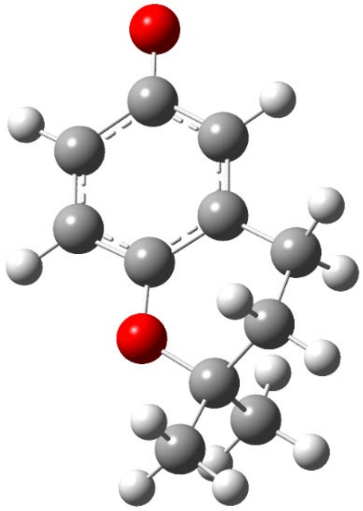

H	3.20265900	-0.76935900	1.70277300
H	2.69040100	0.91297900	1.96404600
H	1.49691900	-0.39841200	2.02851000
O	1.07834400	1.06028300	-0.15293300
C	-1.17881300	1.64310100	-0.08171900
H	-0.85072400	2.67716800	-0.11778800
C	-2.50028600	1.31077000	-0.02219800
H	-3.27426100	2.07184300	-0.00804900
C	-1.86772300	-1.07733000	-0.00286300
H	-2.17360400	-2.12066100	0.00911400

3 anion

Charge=-1, Multiplicity=1

C	-0.16391200	0.63809500	-0.17908500
C	-0.52630500	-0.71115200	-0.11680700
C	-2.91521500	-0.07221100	0.04216000
O	-4.18787900	-0.40051800	0.14859100
C	0.53611900	-1.78659100	-0.14483000
H	0.64797800	-2.21215200	0.86062600
H	0.21502800	-2.60731100	-0.79413000
C	1.87190400	-1.22190700	-0.62241200
H	1.84284100	-1.05376800	-1.70601500
H	2.68685400	-1.92302100	-0.41652700
C	2.18166300	0.11851500	0.04801700
C	3.47370800	0.70877000	-0.49224500
H	3.64311700	1.70463100	-0.07102300
H	4.31798500	0.06895700	-0.21981100
H	3.43293100	0.78881600	-1.58286200
C	2.23359300	0.00911500	1.57057900
H	2.97676500	-0.73730800	1.86797100
H	2.51537300	0.97393000	2.00323400
H	1.26553200	-0.28461800	1.98669000
O	1.15248000	1.06685200	-0.31972400
C	-1.14966500	1.62555600	-0.13550100
H	-0.84748400	2.66891100	-0.18698700
C	-2.49333400	1.28386600	-0.03223100
H	-3.24942000	2.06494000	-0.00167300
C	-1.88254400	-1.04037800	-0.00646500
H	-2.16038000	-2.09274900	0.04248900

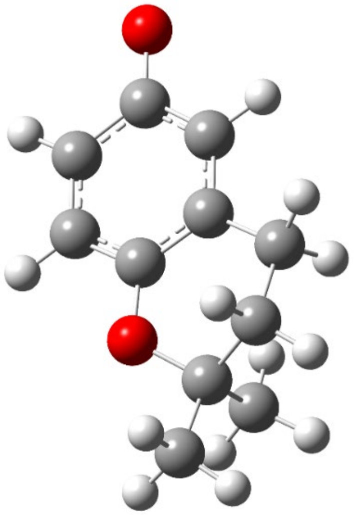

4

Charge=0, Multiplicity=1

C	-0.09628200	0.38644500	-0.12363700
C	-0.38577800	-0.98201000	-0.11037000
C	-2.73990000	-0.44466000	0.04021700
O	-4.04028500	-0.90274200	0.11790400
H	-4.64876700	-0.14993600	0.12341200
C	0.72515200	-2.00165500	-0.18817300
H	0.87640900	-2.45131000	0.80102400
H	0.42839200	-2.81479000	-0.85753900
C	2.01784800	-1.35355100	-0.67402700
H	1.95082200	-1.14051400	-1.74773100
H	2.87024700	-2.02169800	-0.51855500
C	2.27905300	-0.03328000	0.05129100
C	3.51615500	0.65546500	-0.49906900
H	3.64406400	1.63769100	-0.03398100
H	4.40229200	0.05155400	-0.28400500
H	3.43426500	0.78428300	-1.58254400
C	2.38253900	-0.20845800	1.56421500
H	3.16909000	-0.93077700	1.80346200
H	2.63172100	0.74829600	2.03274700
H	1.44281000	-0.56754600	1.99399600
O	1.18685700	0.87841500	-0.23265000
C	-1.11752800	1.34924900	-0.06184300
C	-2.44016700	0.91828000	0.02006300
H	-3.24522300	1.64818000	0.06804900
C	-1.72259200	-1.38658500	-0.02439700
H	-1.96746600	-2.44565200	-0.01742800
C	-0.77273700	2.81288400	-0.08949700
H	-0.11276500	3.07695000	0.74314800
H	-0.24569100	3.07644800	-1.01229400
H	-1.67735600	3.42068500	-0.02243200

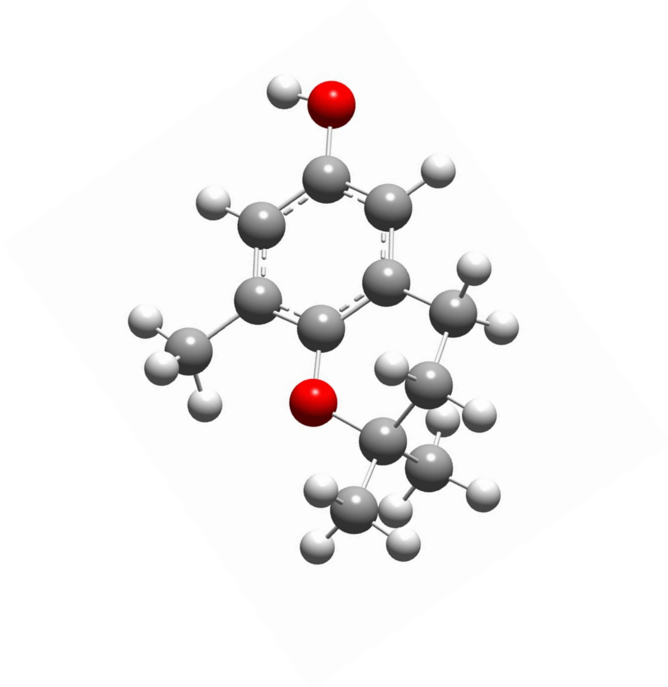

4-dmg

Charge=0, Multiplicity=2

C	-0.15424000	0.36768500	-0.06993400
C	-0.43048400	-1.03034500	-0.06692600
C	-2.83174600	-0.48913400	0.03381500
O	-4.03539700	-0.87719200	0.08610900
C	0.70957200	-2.01499600	-0.14161600
H	0.90687600	-2.42085400	0.85720700
H	0.41620300	-2.85742400	-0.77353300
C	1.95829500	-1.33622100	-0.69376600
H	1.82593400	-1.11685500	-1.75978900
H	2.83247900	-1.98502800	-0.59086200
C	2.24169300	-0.02449300	0.03111000
C	3.38836600	0.73123300	-0.61446400
H	3.52072700	1.70561900	-0.13558400
H	4.31152100	0.15706200	-0.49725200
H	3.19932900	0.87897200	-1.68149900
C	2.46856000	-0.20414300	1.52756500
H	3.30708100	-0.88787100	1.68908700
H	2.70717500	0.75934200	1.98627400
H	1.58472800	-0.61445400	2.02402700

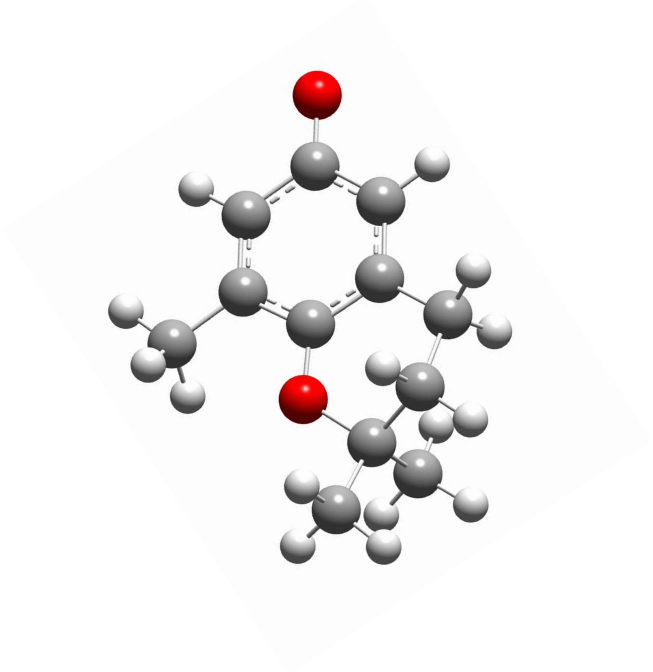

O	1.08371100	0.86297700	-0.12435500
C	-1.19918200	1.34868200	-0.04119600
C	-2.49534200	0.91779300	0.01424600
H	-3.31316700	1.63276800	0.04587500
C	-1.74102700	-1.43306500	-0.01882900
H	-1.98843300	-2.49171100	-0.02942700
C	-0.83120700	2.80439500	-0.06340300
H	-0.19978500	3.05943000	0.79314300
H	-0.26564600	3.04958000	-0.96754700
H	-1.73108800	3.42080900	-0.03376400

4 anion

Charge=-1, Multiplicity=1

C	-0.13507300	0.38375900	-0.14464400
C	-0.42425400	-0.98274800	-0.13000200
C	-2.83642900	-0.48402600	0.05205100
O	-4.09230900	-0.88026700	0.14768300
C	0.69362400	-1.99886600	-0.20081800
H	0.83401600	-2.45433600	0.78785800
H	0.41075800	-2.81142400	-0.87764800
C	1.99525100	-1.35027700	-0.66369800
H	1.95029400	-1.14257700	-1.73991500
H	2.84651300	-2.01575500	-0.48850500
C	2.23771300	-0.02262900	0.05713600
C	3.49337900	0.65663300	-0.46403600
H	3.61232300	1.64244300	-0.00369700
H	4.37197100	0.05219300	-0.22068900
H	3.44214600	0.77788300	-1.55041500
C	2.30532500	-0.18891200	1.57411900
H	3.08637000	-0.90868100	1.83847700
H	2.54208900	0.77115000	2.04288400
H	1.35550800	-0.54609100	1.98251600
O	1.15793800	0.88298600	-0.26725700
C	-1.16650900	1.33389400	-0.07036600
C	-2.48490200	0.89347500	0.02246300
H	-3.28433100	1.63058500	0.07840300
C	-1.75852000	-1.39546500	-0.02931400
H	-1.97727700	-2.46261500	-0.01732100
C	-0.83375500	2.80319900	-0.09684400
H	-0.16817100	3.07440700	0.72963400
H	-0.31921400	3.07941700	-1.02348500
H	-1.74312700	3.40342700	-0.01780000

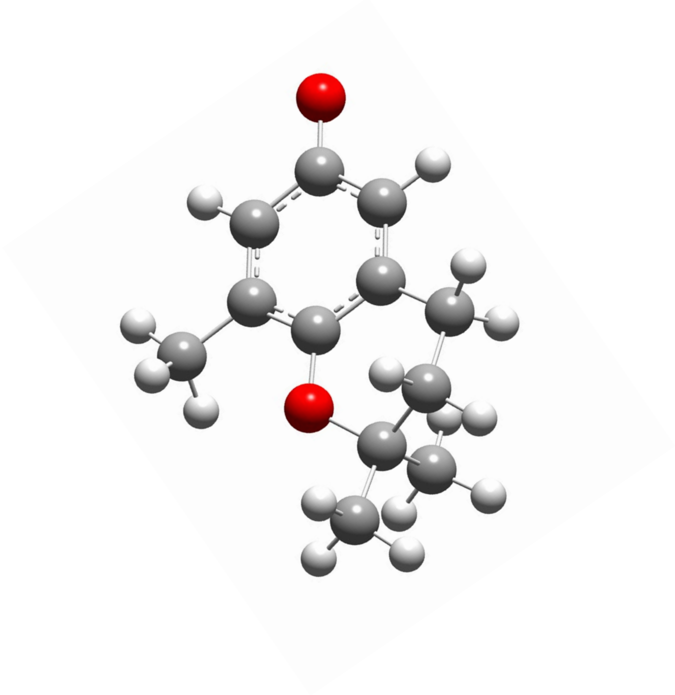

5

Charge=0, Multiplicity=1

C	0.14455400	0.58814000	-0.14468100
C	-0.35256900	-0.71936900	-0.13494400
C	-2.58642700	0.16919400	0.04235900
O	-3.94766300	-0.05605000	0.13729500
H	-4.40848700	0.79253800	0.20151500
C	0.59480400	-1.89180500	-0.23766800
H	0.64913900	-2.40385000	0.73239400
H	0.19729700	-2.62537600	-0.94668100
C	1.98503300	-1.44435300	-0.67923700
H	1.98193700	-1.21522900	-1.75167200
H	2.71832900	-2.23883600	-0.51076700
C	2.42480600	-0.18540700	0.06464200
C	3.76685900	0.31175800	-0.44428200
H	4.02976500	1.25897300	0.03661800
H	4.54486100	-0.42177600	-0.21445100
H	3.73530700	0.46114000	-1.52773400
C	2.45674000	-0.38185500	1.57821700
H	3.12929400	-1.20681500	1.83264000
H	2.82074600	0.52855100	2.06380500
H	1.46447700	-0.61190800	1.97707300
O	1.48974900	0.87717100	-0.24700600
C	-0.70965000	1.69902700	-0.07752100
C	-2.07895000	1.46849700	0.01008300
H	-2.76464500	2.31159700	0.06128000
C	-1.73974100	-0.93797400	-0.02336500
C	-2.28789500	-2.34080700	0.01154600
H	-2.21869600	-2.81740600	-0.97334600
H	-1.72404800	-2.96410700	0.71217200
H	-3.33509600	-2.34851800	0.31490800
C	-0.14578200	3.09320300	-0.10031300
H	0.54895700	3.25122300	0.73099900
H	0.41164900	3.28092400	-1.02388900
H	-0.94817100	3.83042700	-0.02619500

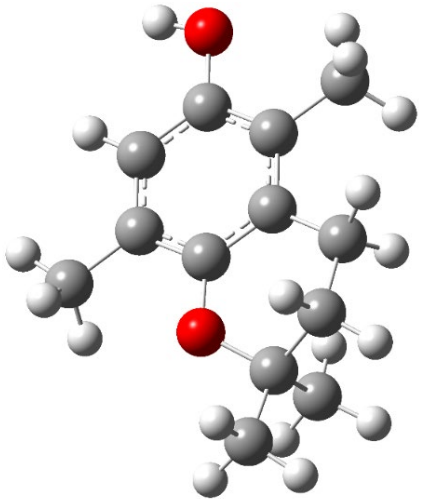

5-dmg

Charge=0, Multiplicity=2

C	0.09888500	0.58196500	-0.09697000
C	-0.40672400	-0.74478000	-0.09958000
C	-2.67663500	0.19081000	0.04927400
O	-3.93058300	0.03421300	0.11917000
C	0.55114100	-1.90503600	-0.22743100
H	0.64321900	-2.41499800	0.73979900
H	0.13801000	-2.63864100	-0.92612800
C	1.91575800	-1.43345200	-0.71763700
H	1.86148300	-1.16871100	-1.78015400
H	2.66187600	-2.22544000	-0.60798900
C	2.38713500	-0.20551300	0.05118400
C	3.67824700	0.35053500	-0.51966300
H	3.95202800	1.27927100	-0.01072900
H	4.48187700	-0.37648800	-0.37395000
H	3.57223900	0.54685200	-1.59039000
C	2.50337300	-0.44891300	1.55154800
H	3.20897300	-1.26467600	1.73445300
H	2.87258900	0.45283500	2.04788100
H	1.53989700	-0.71840200	1.99357400
O	1.40623600	0.86424000	-0.14346600
C	-0.75890300	1.72858100	-0.06067900
C	-2.10575600	1.51796100	0.01037000
H	-2.79464700	2.35770900	0.04653100
C	-1.77130900	-0.94866000	-0.00493000
C	-2.34561500	-2.33317700	0.01474300
H	-2.34127700	-2.76951500	-0.99187500
H	-1.75766000	-2.99385200	0.65791600
H	-3.37613500	-2.31856100	0.37004600
C	-0.15165700	3.10113200	-0.08827700
H	0.51844000	3.24856600	0.76430900
H	0.44188100	3.24802400	-0.99595700
H	-0.93565900	3.85968700	-0.05468800

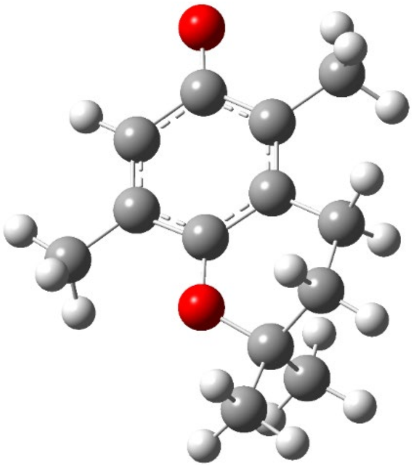

5 anion

Charge=-1, Multiplicity=1

C	0.11331000	0.59344200	-0.15244000
C	-0.39841100	-0.70884100	-0.13836500
C	-2.68561800	0.18157800	0.04878100
O	-3.99520900	0.02232300	0.13436300
C	0.54625400	-1.88756700	-0.23892400
H	0.59584700	-2.40094200	0.73120100
H	0.14927700	-2.62173300	-0.94811500
C	1.94375400	-1.45482800	-0.67346400
H	1.95084700	-1.23313500	-1.74778200
H	2.66973700	-2.25454000	-0.49518800
C	2.38848700	-0.19163900	0.06194700
C	3.74477500	0.27998100	-0.43563000
H	4.01560000	1.22939000	0.03686800
H	4.51045200	-0.46107200	-0.18841400
H	3.72883500	0.41745600	-1.52118300
C	2.40522700	-0.37649200	1.57814900
H	3.06517700	-1.20723500	1.84706100
H	2.77594600	0.53363900	2.05971700

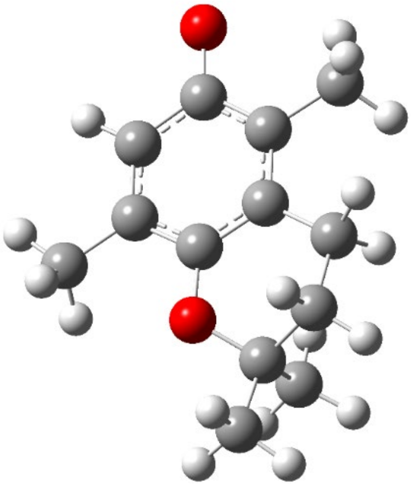

H	1.40629600	-0.59038700	1.96903600
O	1.47200800	0.87309700	-0.27484700
C	-0.73857600	1.70235400	-0.07245400
C	-2.11002400	1.47805100	0.02054900
H	-2.78139200	2.33366200	0.07641300
C	-1.78508000	-0.91960600	-0.01766200
C	-2.32638200	-2.32666000	0.02701800
H	-2.37063500	-2.78288400	-0.97111000
H	-1.70750100	-2.98040200	0.64966800
H	-3.34018700	-2.33208500	0.43142800
C	-0.16913700	3.09736500	-0.09274500
H	0.53714600	3.25179100	0.73008600
H	0.37688300	3.29503500	-1.02166900
H	-0.96819500	3.83725400	-0.00270600

6

Charge=0, Multiplicity=1

C	0.13357500	0.26749300	-0.13192600
C	0.03270600	-1.12710500	-0.10933700
C	-2.37603500	-0.89889400	0.02907300
O	-3.63766700	-1.45886500	0.11078000
H	-3.56191700	-2.42349600	0.09249800
C	1.26542300	-1.99734600	-0.17348600
H	1.46765300	-2.41569900	0.82047700
H	1.07933700	-2.84741100	-0.83703400
C	2.46821400	-1.19415000	-0.65739100
H	2.38589600	-1.00519200	-1.73453900
H	3.39879900	-1.74323100	-0.48457700
C	2.54440100	0.15825200	0.05120200
C	3.68643000	0.99656500	-0.49734800
H	3.68044300	1.99257200	-0.04408700
H	4.64173900	0.51600900	-0.26775300
H	3.59883600	1.10086400	-1.58303900
C	2.65387300	0.01843700	1.56740600
H	3.52730800	-0.58876900	1.82450100
H	2.76799700	1.00610600	2.02410700
H	1.76629000	-0.45744100	1.99444200
O	1.34668500	0.91356500	-0.25932900
C	-1.00823400	1.08530500	-0.06845100
C	-2.28145900	0.49906000	-0.00411700
C	-1.23950300	-1.69578900	-0.02090200
H	-1.34169300	-2.77934300	-0.00135600
C	-0.86745400	2.58561600	-0.08950500
H	-1.59853000	3.05623900	0.57255300
H	0.13011200	2.89293600	0.22558800
H	-1.03607300	2.98362900	-1.09759600
C	-3.52082100	1.35599500	0.03106900
H	-4.41676400	0.76250500	-0.15246800
H	-3.63642300	1.84693500	1.00443500
H	-3.47185800	2.14455500	-0.72547300

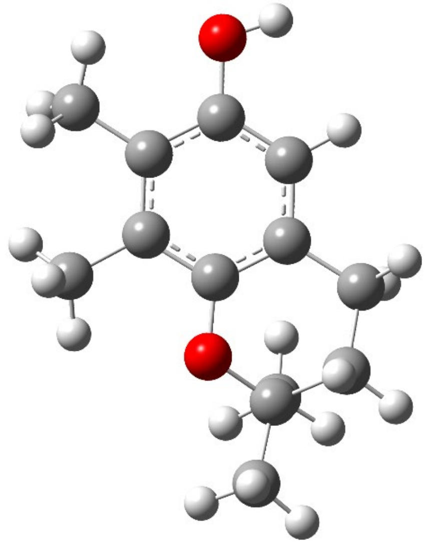

6-dmg

Charge=0, Multiplicity=2

C	0.08003800	0.23386000	-0.08221300
C	0.01306000	-1.18869400	-0.07384700
C	-2.44153600	-0.98704800	0.02706600
O	-3.56587100	-1.56580400	0.08085600
C	1.27974900	-2.00397500	-0.14601200
H	1.52818200	-2.38202700	0.85233400
H	1.10991300	-2.87682000	-0.78206800
C	2.42364900	-1.15456500	-0.68827700
H	2.27156800	-0.95543200	-1.75568100
H	3.38075400	-1.67156600	-0.57622000
C	2.50663300	0.18383900	0.03846300
C	3.54765900	1.09265100	-0.58869100
H	3.53263600	2.07666200	-0.11131100
H	4.54024100	0.65455400	-0.45253500
H	3.35972800	1.21130800	-1.65957100
C	2.73248700	0.03669700	1.53886200
H	3.65511100	-0.52368500	1.71637000
H	2.82740600	1.02412900	1.99905600
H	1.90625500	-0.49217900	2.02234200
O	1.23950600	0.89867200	-0.13983100
C	-1.08805500	1.05703400	-0.05874300
C	-2.33274400	0.47015700	0.00236500
C	-1.22798000	-1.76459600	-0.02284000
H	-1.33093000	-2.84694200	-0.02882400
C	-0.88683800	2.54743900	-0.09272900
H	-0.30083900	2.87701100	0.77130100
H	-0.32959700	2.84019300	-0.98822800
H	-1.83673000	3.08006400	-0.08907000
C	-3.59593700	1.28264700	0.04481600
H	-3.59680300	1.96459300	0.90095600
H	-3.70092500	1.89284200	-0.85824800
H	-4.46616600	0.63168600	0.12187300

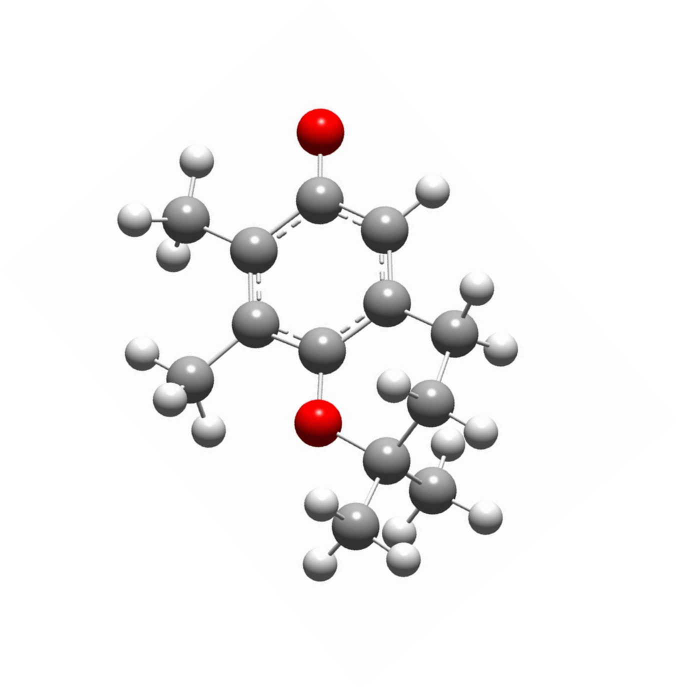

6 anion

Charge=-1, Multiplicity=1

C	0.10094400	0.25528000	-0.15088000
C	0.01547800	-1.13843400	-0.12883700
C	-2.44423000	-0.98440300	0.04163500
O	-3.61991600	-1.57660300	0.15030500
C	1.26417900	-1.99016700	-0.18754400
H	1.46276800	-2.41442400	0.80522000
H	1.10015000	-2.83946600	-0.85869000
C	2.46576100	-1.17172400	-0.65027900
H	2.40054500	-0.98340800	-1.72905300
H	3.40101100	-1.70859600	-0.46200000
C	2.51019900	0.18407600	0.05658900
C	3.65956000	1.03047300	-0.46568800
H	3.63433600	2.02771400	-0.01531600
H	4.61395000	0.55952700	-0.21291200
H	3.59747100	1.13273400	-1.55350100
C	2.59143400	0.04527300	1.57560400
H	3.46633600	-0.55160300	1.85181200
H	2.68494000	1.03436100	2.03452300
H	1.70049100	-0.44040800	1.98388800
O	1.31643800	0.92230800	-0.28655000
C	-1.05833500	1.04894400	-0.08076300
C	-2.31918000	0.43902100	-0.01270800
C	-1.24666500	-1.72981200	-0.02676700
H	-1.31600900	-2.81688200	-0.00081000
C	-0.93955300	2.55526100	-0.08561600
H	0.07167100	2.87407500	0.16940700
H	-1.18130700	2.97380000	-1.07036300
H	-1.63017200	3.00425000	0.63361000
C	-3.57408800	1.27480900	0.01906400
H	-3.85070000	1.56361000	1.04226200
H	-3.46538000	2.19588100	-0.55942100
H	-4.41530500	0.71161400	-0.39155500

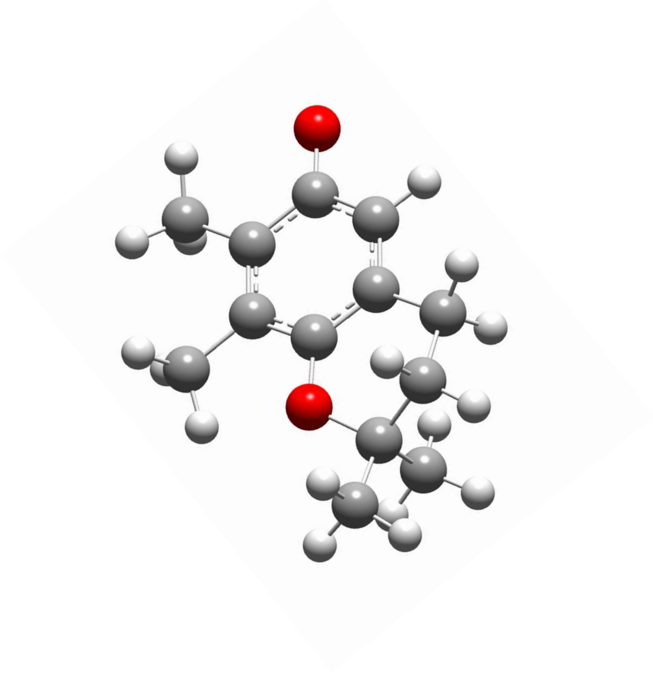

7

Charge=0, Multiplicity=1

C	0.27376900	0.46925500	-0.15545000
C	0.04207800	-0.91091200	-0.13653800
C	-2.31886300	-0.46270600	0.02969300
O	-3.60540100	-0.96799700	0.11118700
H	-4.24665500	-0.24667200	0.17147400
C	1.19266100	-1.88666600	-0.22851000
H	1.33581200	-2.37524100	0.74475700
H	0.94264000	-2.68528500	-0.93463200
C	2.47906700	-1.19372800	-0.66429400
H	2.44587600	-0.98091100	-1.73957700
H	3.34643900	-1.83434300	-0.47775800
C	2.66164000	0.13350200	0.06720100
C	3.89080100	0.87169000	-0.43522700
H	3.96402700	1.85641100	0.03653500
H	4.79130100	0.30147000	-0.18978400
H	3.84375500	1.00167200	-1.52066200
C	2.71460300	-0.03592900	1.58359900
H	3.53135300	-0.71211400	1.85439500

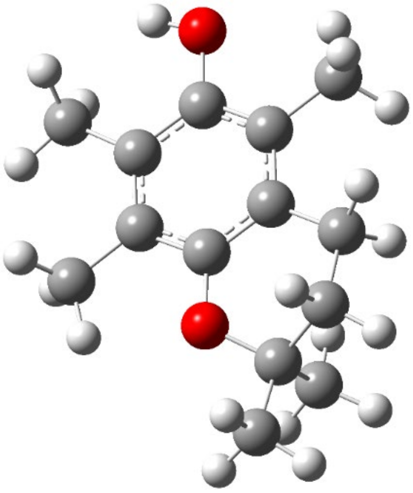

H	2.89081200	0.93325000	2.05990700
H	1.78150900	-0.44874500	1.97778700
O	1.54740500	0.99417100	-0.27045900
C	-0.77934400	1.39765800	-0.09003100
C	-2.09513200	0.92095900	-0.00472000
C	-1.27521500	-1.38639600	-0.02423200
C	-1.54802400	-2.86787400	0.02107000
H	-1.41434600	-3.32537600	-0.96641300
H	-0.85915000	-3.37113900	0.70589100
H	-2.56674400	-3.07364600	0.34986600
C	-0.49531600	2.88025100	-0.11918400
H	0.57565900	3.07594800	-0.13952400
H	-0.94169200	3.35033800	-1.00186100
H	-0.91446400	3.37850800	0.76059800
C	-3.28232300	1.84976800	0.05311300
H	-3.86351600	1.69526300	0.97054600
H	-2.98377900	2.89669300	0.02871400
H	-3.95728200	1.68130100	-0.79502500

7-dmg

Charge=0, Multiplicity=2

C	-0.22016700	-0.44155200	-0.09861900
C	-0.00239700	0.96105400	-0.09358400
C	2.41141900	0.49541100	0.03340700
O	3.59805800	0.93174900	0.07573100
C	-1.17706900	1.90266900	-0.21587700
H	-1.37289900	2.37850100	0.75330600
H	-0.92335000	2.70843500	-0.91096800
C	-2.41602400	1.16466700	-0.70999200
H	-2.30859200	0.92264700	-1.77393000
H	-3.30889700	1.78595800	-0.59667400
C	-2.62420400	-0.13848300	0.05122700
C	-3.77366600	-0.94430500	-0.52540200
H	-3.85031900	-1.91320800	-0.02353100
H	-4.71008400	-0.39980800	-0.37578500
H	-3.62945200	-1.10720600	-1.59734000
C	-2.79201900	0.06740600	1.55253200
H	-3.64920500	0.72140200	1.73784300
H	-2.97124200	-0.89376000	2.04245700
H	-1.90470300	0.52402900	2.00034200
O	-1.44622200	-0.98264700	-0.14779900
C	0.84658300	-1.39137300	-0.06906500
C	2.14356300	-0.93998200	0.00204800
C	1.29282300	1.42673500	-0.00084000
C	1.58963700	2.89680500	0.02870900
H	1.63314200	3.30520500	-0.98897500
H	0.81643700	3.44630000	0.57064200
H	2.55429800	3.08549800	0.50094900
C	0.48383700	-2.85108300	-0.10627900
H	-0.11724700	-3.12253300	0.76778300
H	-0.11879700	-3.07682600	-0.99153000
H	1.37055500	-3.48327900	-0.12415300
C	3.31127500	-1.88606600	0.05134400
H	3.21346200	-2.58746300	0.88561100

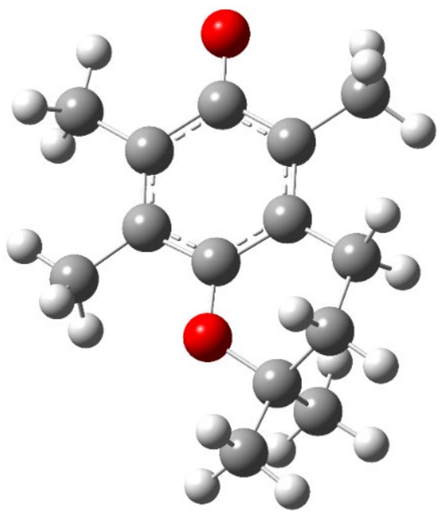

H	3.37515700	-2.47962800	-0.86684200
H	4.24549300	-1.33849500	0.16968600

7 anion

Charge=-1, Multiplicity=1

C	-0.24417500	-0.46027700	-0.16461800
C	-0.00803300	0.92045600	-0.14497800
C	2.40629900	0.48595700	0.02865400
O	3.65053500	0.92516600	0.10416800
C	-1.16991800	1.89029600	-0.24138400
H	-1.31400700	2.38885900	0.72673100
H	-0.93407200	2.68325900	-0.95815300
C	-2.46045900	1.19507000	-0.66289200
H	-2.43941900	0.98273600	-1.73896200
H	-3.32641600	1.83599600	-0.46795600
C	-2.63287000	-0.13518100	0.06562600
C	-3.87413100	-0.86615300	-0.41958000
H	-3.94423400	-1.85294300	0.04879400
H	-4.76934000	-0.29414500	-0.15870600
H	-3.84473200	-0.99248600	-1.50625700
C	-2.66663800	0.03091100	1.58384400
H	-3.48066600	0.70488100	1.86867700
H	-2.83489500	-0.93985400	2.06040900
H	-1.72863600	0.44390900	1.96574000
O	-1.52854900	-0.98914300	-0.29823800
C	0.81030200	-1.38496300	-0.08637900
C	2.12763500	-0.91090000	-0.00517900
C	1.31065000	1.38968100	-0.02070000
C	1.61733600	2.86451200	0.05239200
H	2.05208400	3.23558800	-0.88542500
H	0.73290100	3.46566900	0.26908800
H	2.35838400	3.06150500	0.83401900
C	0.51868400	-2.86929800	-0.10060800
H	-0.55265500	-3.06287400	-0.05655800
H	0.90743500	-3.34498100	-1.00819200
H	0.98757300	-3.37231700	0.75086600
C	3.30793800	-1.84725400	0.05081800
H	3.85298800	-1.74248400	0.99742000
H	3.01918700	-2.89348500	-0.05648800
H	4.02692700	-1.61215700	-0.74258400

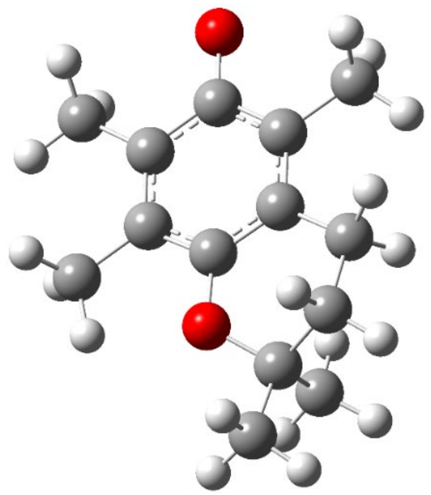

8

Charge=0, Multiplicity=1

C	1.41105500	0.02153100	-0.00201300
C	0.69968400	1.21953000	0.00006800
C	-0.69238600	1.20225800	0.00801200
C	-1.39766000	-0.00859100	0.01019800
C	-0.66827800	-1.20330600	0.00799500
C	0.72584600	-1.19126300	0.00030300
H	1.23993000	2.16140300	-0.00523800
H	-1.24267300	2.13948900	0.00970500
H	-1.19789900	-2.15235000	0.00977300
H	1.28265500	-2.12471800	-0.00489500
O	2.79170400	0.09246300	-0.00990800
H	3.16301900	-0.80118200	-0.01011200
N	-2.80136200	-0.02165700	0.08176300
H	-3.22692300	0.80625800	-0.32065600
H	-3.21177000	-0.85795100	-0.31904700

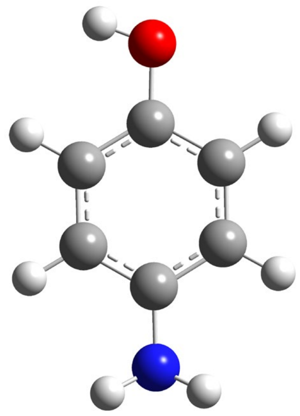

8-dmg

Charge=0, Multiplicity=2

C	-1.50896200	0.00000900	-0.00002400
C	-0.75105600	1.23158700	0.00003400
C	0.61486500	1.23063500	0.00006000
C	1.33698800	-0.00001500	0.00013000
C	0.61488100	-1.23063000	0.00008700
C	-0.75105900	-1.23155100	0.00006000
H	-1.30598700	2.16509700	0.00019400
H	1.17558500	2.16129600	0.00012100
H	1.17556800	-2.16131100	0.00010000
H	-1.30598100	-2.16506600	-0.00008000
O	-2.77859000	-0.00002300	-0.00010200
N	2.67998800	0.00000300	-0.00037400
H	3.19779000	-0.86913700	0.00065000
H	3.19789000	0.86907700	0.00037000

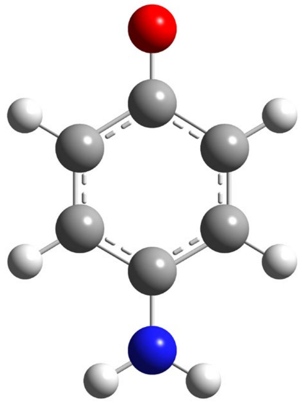

8 anion

Charge=-1, Multiplicity=1

C	-1.52490100	-0.00000600	0.00300900
C	-0.76639700	1.19864200	-0.00075900
C	0.62841200	1.19663700	-0.01041700
C	1.35215400	0.00000000	-0.01198300
C	0.62841900	-1.19663500	-0.01036800
C	-0.76639700	-1.19864700	-0.00077600
H	-1.30039300	2.14662700	0.00521300
H	1.16763300	2.14240800	-0.01318800
H	1.16764200	-2.14240600	-0.01305200
H	-1.30038600	-2.14663700	0.00520700
O	-2.84801500	0.00000200	0.01343900
N	2.77176900	0.00005400	-0.08685200
H	3.16975500	0.82419800	0.35241400
H	3.16973900	-0.82452200	0.35161500

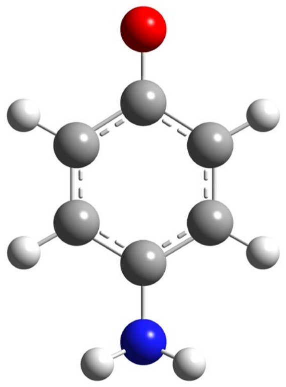

9

Charge=0, Multiplicity=1

C	-2.18606300	0.00601200	0.03408800
C	-1.51895600	-1.20051600	-0.18347300
C	-0.13499100	-1.21948400	-0.29004200
C	0.62804200	-0.04155800	-0.17588000
C	-0.06253700	1.15780700	0.04645700
C	-1.45713400	1.18267800	0.14393900
H	-2.09094900	-2.11862300	-0.27742400
H	0.35640700	-2.16716700	-0.48407400
H	0.46931600	2.09606100	0.14660800
H	-1.97342400	2.12411800	0.31334500
N	2.03964400	-0.09968500	-0.32867200
O	-3.56170800	-0.02751800	0.12891800
H	-3.89882000	0.86815400	0.27501300
C	2.67804300	-1.11079900	0.52007400
H	3.74418900	-1.14500000	0.28708100
H	2.55985600	-0.87155300	1.58875200
H	2.26475700	-2.10158200	0.33611700
C	2.72535700	1.17722500	-0.17607300
H	3.78996300	1.02187900	-0.35937200
H	2.35734300	1.90166000	-0.90537900
H	2.60695200	1.60180900	0.83415300

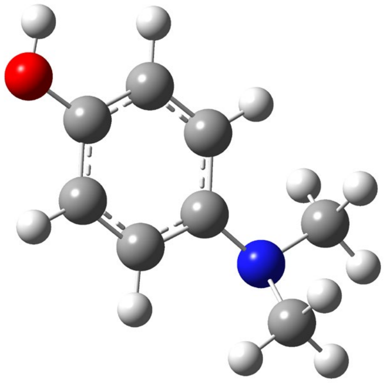

9-dmg

Charge=0, Multiplicity=2

C	-2.29347800	0.00001100	0.00003400
C	-1.53166200	1.22571100	-0.00773100
C	-0.16476900	1.22784300	-0.00739400
C	0.57408600	0.00002600	-0.00006600
C	-0.16474600	-1.22781000	0.00700300
C	-1.53164600	-1.22568500	0.00747300
H	-2.08170200	2.16231400	-0.01502700
H	0.36348000	2.17368800	-0.01567900
H	0.36350500	-2.17365600	0.01496800
H	-2.08167900	-2.16229400	0.01460600
N	1.92340000	0.00000600	0.00030900
O	-3.56463100	-0.00000500	0.00030700
C	2.66569800	1.25832700	0.01382200
H	3.72970100	1.03580500	0.04103500
H	2.45057500	1.84623800	-0.88334900
H	2.40784000	1.85008700	0.89663300
C	2.66553000	-1.25838900	-0.01372100
H	3.72957900	-1.03599700	-0.04025100
H	2.44989800	-1.84684100	0.88297000
H	2.40798000	-1.84954600	-0.89704700

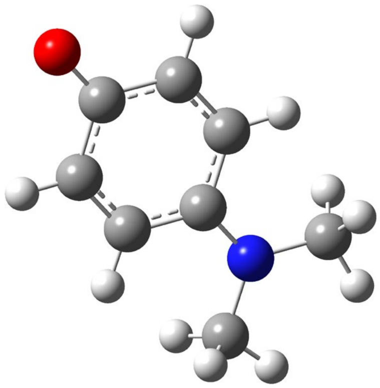

9 anion

Charge=-1, Multiplicity=1

C	-2.29370400	-0.04239800	0.05126000
C	-1.59550900	1.16313300	-0.23849500
C	-0.21238400	1.20440000	-0.36056900
C	0.58020400	0.05626900	-0.19227800
C	-0.08671400	-1.14163900	0.09042900
C	-1.47970900	-1.19092300	0.19910500
H	-2.17433900	2.07417100	-0.37526500

H	0.26763600	2.15012600	-0.60246900
H	0.46984800	-2.06360600	0.22482500
H	-1.96349300	-2.14193200	0.41197200
N	2.00532500	0.16035200	-0.33901400
O	-3.60493800	-0.08243300	0.16771700
C	2.70463000	-1.11657800	-0.29214500
H	3.76530400	-0.94207400	-0.48675700
H	2.61673600	-1.61459800	0.68829500
H	2.32073300	-1.79214700	-1.05998000
C	2.59073000	1.06288800	0.65714300
H	3.65904300	1.17858000	0.45638300
H	2.12755500	2.04874900	0.60766100
H	2.46793000	0.66882500	1.67999200

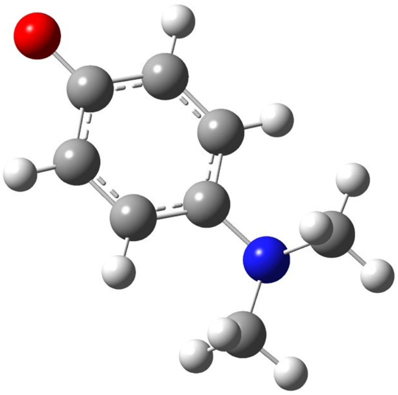

10

Charge=0, Multiplicity=1

C	1.75249600	-0.67374900	-0.04315200
C	0.55093300	-1.39220400	0.02576900
C	-0.67852300	-0.69492700	0.08403000
C	-0.70645900	0.70855300	0.06767500
C	0.50002100	1.43042300	0.08095600
C	1.70212000	0.72852300	0.02412800
C	0.49634700	2.93803900	0.12194200
H	0.33160300	3.36510200	-0.87488700
H	-0.29945500	3.31150900	0.77158600
H	1.44668100	3.32358000	0.49320500
C	0.50462300	-2.89955700	0.03821200
H	-0.17971700	-3.24872300	0.81739200
H	0.13149400	-3.30031400	-0.91329100
H	1.47842600	-3.34785500	0.22845900
C	3.10664000	-1.32919500	-0.18165700
H	3.68358000	-1.27105000	0.74987800
H	3.03294800	-2.37922300	-0.45902300
H	3.69676000	-0.83928100	-0.96457300
O	2.86832900	1.47995900	0.00169300
H	3.63754600	0.90404700	0.10482900
C	-2.02474400	1.45132900	-0.02278600
H	-2.30875500	1.84865900	0.96168100
H	-1.90593000	2.31707500	-0.68219800
C	-3.13529500	0.54322300	-0.54107900
H	-2.96185100	0.30045300	-1.59688700
H	-4.10560400	1.04303800	-0.46574100
C	-3.13711800	-0.73715200	0.27965100
H	-3.92522300	-1.42205000	-0.03980900
H	-3.33327700	-0.48021600	1.32914400
N	-1.86543700	-1.45446800	0.19824200
H	-1.89406000	-2.22298700	-0.46313900

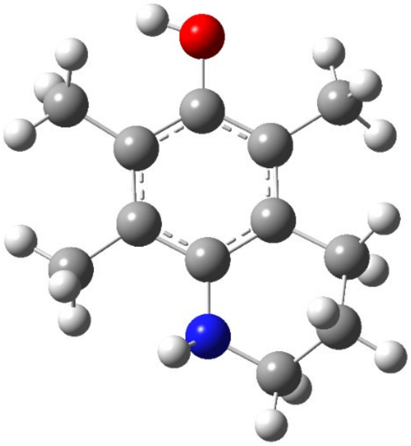

10-dmg

Charge=0, Multiplicity=2

C	-1.82707800	-0.57961000	-0.01978600
C	-0.68521700	-1.34649500	0.00017600
C	0.59958900	-0.70523700	0.05952700
C	0.71467100	0.72320800	0.05749800
C	-0.42496500	1.49322700	0.05857100
C	-1.73729900	0.87392700	-0.00229300
C	-0.35529600	2.99477800	0.10147600
H	0.48287800	3.33793400	0.71223800
H	-0.22582600	3.41475700	-0.90399500
H	-1.27753400	3.40814200	0.51218800
C	-0.72725700	-2.85199000	-0.03572200
H	-0.13759200	-3.24303900	-0.87315200
H	-0.31700500	-3.28511700	0.88468500
H	-1.74470700	-3.22231600	-0.14974700
C	-3.19840600	-1.20091600	-0.06472900
H	-3.35927000	-1.74357200	-1.00270000
H	-3.33792000	-1.91490400	0.75263000
H	-3.96565300	-0.43174100	0.01706100
O	-2.78545100	1.59284000	-0.05172000
C	2.09314500	1.34977500	0.05453600
H	2.07555300	2.26491200	-0.54336400
H	2.36399700	1.64515200	1.07763200
C	3.14779600	0.39129000	-0.49249900
H	4.14842600	0.81169300	-0.36611400
H	2.98409300	0.22497400	-1.56343100
C	3.06090300	-0.93563300	0.23819000
H	3.31143100	-0.79861900	1.29820500
H	3.74800500	-1.67364500	-0.17861500
N	1.71000500	-1.47143800	0.11872600
H	1.60117200	-2.47521300	0.17948500

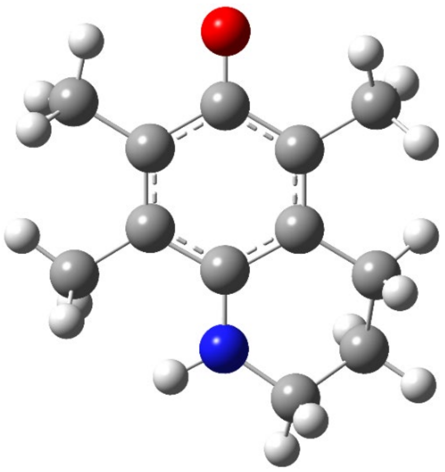

10 anion

Charge=-1, Multiplicity=1

C	-1.80383100	-0.58488700	0.03808600
C	-0.62982100	-1.34670300	-0.03133800
C	0.62913500	-0.71166800	-0.06740300
C	0.70088500	0.69204100	-0.04673200
C	-0.47880600	1.45750900	-0.07229000
C	-1.75748800	0.83993500	-0.01781800
C	-0.42452000	2.96466500	-0.13195700
H	-0.51261300	3.42243100	0.86324500
H	0.50484300	3.32690100	-0.57708300
H	-1.25680400	3.34983000	-0.72755000
C	-0.69806700	-2.85673200	-0.04540900
H	0.15753800	-3.27834100	-0.57685700
H	-0.68693700	-3.27574400	0.96965900
H	-1.60811800	-3.21267600	-0.53331800
C	-3.15208400	-1.25214300	0.16421000
H	-3.58987400	-1.49472000	-0.81387500
H	-3.09484800	-2.18304800	0.73387700
H	-3.85653400	-0.58906400	0.67112900
O	-2.86631600	1.56342500	-0.00611200
C	2.04734800	1.39158700	0.05213600

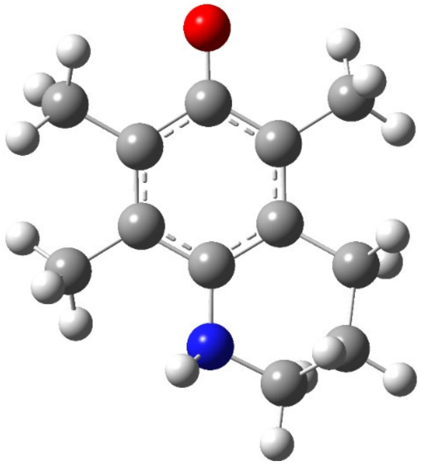

H	2.32317800	1.82497900	-0.91977400
H	1.96705300	2.23264000	0.74884800
C	3.15121200	0.44177700	0.50538500
H	3.02130900	0.18534400	1.56488600
H	4.13283500	0.91364500	0.39435300
C	3.06823700	-0.82787500	-0.32818100
H	3.87543700	-1.52436500	-0.08811200
H	3.17217000	-0.56021700	-1.38877500
N	1.80187100	-1.53368600	-0.14848000
H	1.85559700	-2.15422800	0.65546600

11

Charge=0, Multiplicity=1

C	0.20621500	-2.55468200	0.02271500
H	0.11486900	-2.95892200	1.03900400
H	-0.31963000	-3.25214200	-0.63719200
C	1.68254600	-2.46106700	-0.34822800
H	1.79998200	-2.36352800	-1.43337500
H	2.21122700	-3.36691900	-0.03841800
C	2.29852000	-1.24646700	0.32485900
H	3.36682800	-1.17860900	0.10183800
H	2.19086600	-1.33207600	1.42160300
C	2.33718200	1.18107700	0.32435800
H	2.23304700	1.27019900	1.42116000
H	3.40272800	1.07944500	0.10061800
C	1.75942100	2.41441000	-0.34839300
H	1.87284700	2.31310300	-1.43363100
H	2.31649900	3.30325100	-0.03909400
C	0.28722500	2.55438600	0.02393600
H	-0.21683300	3.26947600	-0.63403500
H	0.20984100	2.95930700	1.04111300
N	1.66706900	-0.02248100	-0.16020900
O	-3.93576600	0.12188500	0.04313500
H	-4.31008900	-0.77053300	0.04455200
C	-1.87771400	-1.16800700	0.01172800
H	-2.43867600	-2.10058900	0.03012400
C	-1.83868700	1.23501600	0.01136500
H	-2.37259500	2.18205100	0.02981700
C	-0.44404000	1.22787700	-0.01686500
C	0.25307900	-0.00064500	-0.04882500
C	-0.48167100	-1.20522400	-0.01675800
C	-2.55612100	0.04478900	0.02049100

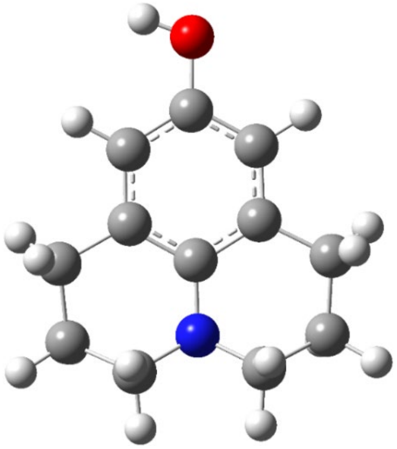

11-dmg

Charge=0, Multiplicity=2

C	0.22679500	-2.54574000	-0.16895400
H	-0.35635400	-3.33626200	0.31038700
H	0.34141600	-2.82082700	-1.22574500
C	1.60702000	-2.40784200	0.45851900
H	2.19645800	-3.31586400	0.30988100
H	1.51847400	-2.23487500	1.53693800
C	2.33429100	-1.24121100	-0.18381000
H	2.53000900	-1.44785200	-1.24514300
H	3.29471100	-1.06210600	0.30384800
C	2.33416600	1.24104100	-0.18487000
H	3.29518900	1.06202400	0.30159600
H	2.52854800	1.44762600	-1.24646000
C	1.60755600	2.40767800	0.45814000
H	2.19697700	3.31565500	0.30913600
H	1.51983200	2.23459800	1.53660700
C	0.22691700	2.54581400	-0.16830200
H	0.34081500	2.82158400	-1.22499200
H	-0.35595100	3.33601500	0.31191400
N	1.55996900	-0.00008400	-0.08079700
O	-3.92256800	0.00014900	0.10484700
C	-1.89361600	-1.22308700	-0.01571200
H	-2.44479800	-2.16050400	0.00148900
C	-1.89348800	1.22319100	-0.01545300
H	-2.44458700	2.16065300	0.00202800
C	-0.52637600	1.24221700	-0.08250900
C	0.20440500	-0.00003000	-0.08632800
C	-0.52651200	-1.24219800	-0.08278100
C	-2.65115300	0.00008800	0.02961300

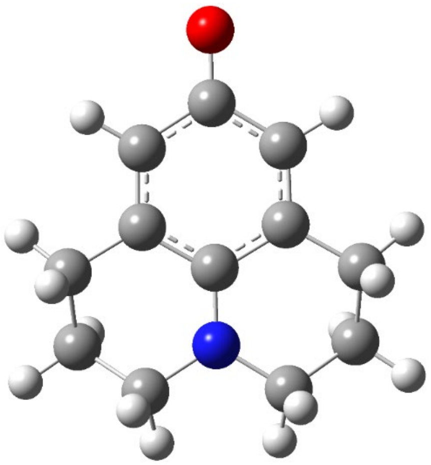

11 anion

Charge=-1, Multiplicity=1

C	0.20539900	-2.55154100	-0.01498400
H	-0.29910700	-3.25715200	0.65357100
H	0.10634000	-2.96203600	-1.02843700
C	1.68674800	-2.43994300	0.33348900
H	2.22428400	-3.33514200	0.00688400
H	1.82082900	-2.35201400	1.41787300
C	2.27527000	-1.20799800	-0.33369200
H	2.13810100	-1.27797300	-1.42974800
H	3.34954800	-1.13592900	-0.13947100
C	2.27533400	1.20795900	-0.33364400
H	3.34959000	1.13585200	-0.13936700
H	2.13824600	1.27796700	-1.42971000
C	1.68682500	2.43988600	0.33341100
H	2.22429600	3.33504300	0.00658700
H	1.82092400	2.35223300	1.41781600
C	0.20552000	2.55144000	-0.01490400
H	0.10644700	2.96205100	-1.02831300
H	-0.29889500	3.25703000	0.65374200
N	1.64505800	-0.00001000	0.18884800
O	-3.97988700	0.00009800	-0.06299600
C	-1.90004400	-1.19492800	-0.00876400
H	-2.43277600	-2.14531500	-0.03454000

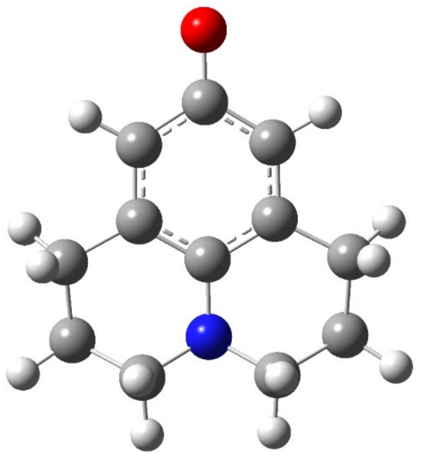

C	-1.89996700	1.19498100	-0.00880600
H	-2.43264900	2.14539600	-0.03458500
C	-0.50456900	1.21100800	0.02892700
C	0.21739100	-0.00001900	0.06371600
C	-0.50462400	-1.21102000	0.02894200
C	-2.65853300	0.00005400	-0.02540400

12

Charge=0, Multiplicity=1

C	-2.32880600	-1.14139400	-0.00003600
C	-2.78164800	0.17718400	-0.00003700
C	-1.86760700	1.23469400	0.00002000
C	-0.50427000	0.96824900	0.00008100
C	-0.02041700	-0.35065300	0.00009100
C	-0.96095900	-1.39287700	0.00000700
H	-3.04815600	-1.95428800	-0.00007800
H	-2.23192700	2.25843100	0.00002000
H	0.18845500	1.80442700	0.00014400
H	-0.61050000	-2.42176600	0.00001100
O	-4.13748100	0.38558800	-0.00007700
H	-4.32987400	1.33504300	0.00012100
C	1.40938900	-0.68330300	0.00016100
H	1.63596100	-1.75020800	0.00032300
C	2.44205700	0.18055900	-0.00009800
H	2.27264000	1.25714100	-0.00029600
C	3.83151400	-0.25970000	-0.00012300
H	3.99926900	-1.33688400	-0.00026200
C	4.87886900	0.57731100	-0.00000200
H	4.73612100	1.65572200	0.00013400
H	5.89912600	0.20726100	0.00011600

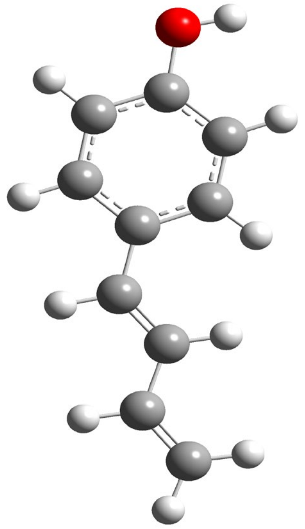

12-dmg

Charge=0, Multiplicity=2

C	-2.38135500	-1.13421000	0.00008600
C	-2.86767700	0.23353700	0.00013000
C	-1.86977200	1.29130100	-0.00012700
C	-0.53833400	1.00588000	-0.00034000
C	-0.07126700	-0.34929600	-0.00024600
C	-1.04392600	-1.39873100	-0.00014500
H	-3.11821900	-1.93168200	0.00021600
H	-2.22752800	2.31647200	-0.00014100
H	0.18010700	1.81920400	-0.00050700
H	-0.68999000	-2.42617300	-0.00017300
O	-4.09752100	0.49368200	0.00038200
C	1.31113600	-0.70203200	-0.00013600
H	1.53413100	-1.76838000	0.00000000
C	2.36853000	0.16707700	-0.00018100
H	2.20883400	1.24378900	-0.00031000
C	3.73690200	-0.28763600	0.00008600
H	3.90442900	-1.36355300	0.00019300
C	4.78184300	0.56142900	0.00032800
H	4.62735600	1.63768900	0.00038600
H	5.80457400	0.19925700	0.00055400

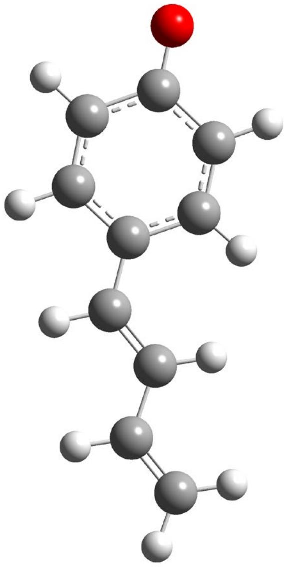

12 anion

Charge=-1, Multiplicity=1

C	2.38783100	1.11655400	0.00006500
C	2.90236300	-0.21221300	0.00004600
C	1.92566300	-1.25585700	-0.00007600
C	0.56717900	-0.98971400	-0.00015300
C	0.07170800	0.33059800	-0.00010100
C	1.02347600	1.36740300	-0.00000600
H	3.09586200	1.94201000	0.00014300
H	2.27943600	-2.28434900	-0.00011800
H	-0.12893800	-1.82557400	-0.00025200
H	0.67254500	2.39820900	0.00002100
O	4.17959200	-0.46459800	0.00013200
C	-1.35018900	0.66124400	-0.00012000
H	-1.57353100	1.73016100	-0.00024000
C	-2.39925800	-0.18940000	0.00003500
H	-2.24412200	-1.26831500	0.00018500
C	-3.77896200	0.27395500	0.00000700
H	-3.92310900	1.35524600	-0.00012500
C	-4.85187200	-0.53297200	0.00013000
H	-4.74174800	-1.61528200	0.00026100
H	-5.86077100	-0.13290900	0.00010500

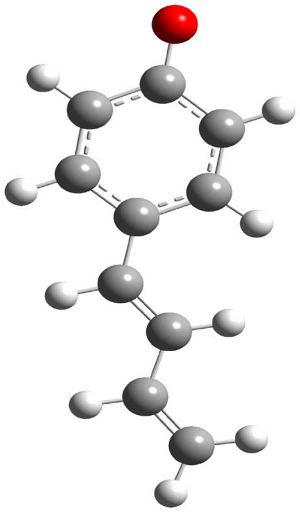

13

Charge=0, Multiplicity=1

C	-2.32851900	1.33264500	0.00003800
C	-1.45720400	0.23368000	-0.00013700
C	-2.02606400	-1.05272200	-0.00029300
C	-3.40241900	-1.23047300	-0.00014300
C	-4.24736200	-0.11658800	0.00011000
C	-3.71130100	1.16972400	0.00008600
H	-1.91399500	2.33751900	0.00010500
H	-1.39019200	-1.93280700	-0.00044200
H	-3.83595700	-2.22571400	-0.00015800
H	-4.37259000	2.03183800	0.00018300
C	-0.00883800	0.48188100	-0.00005400
H	0.26391800	1.53623400	0.00027900
C	0.95923100	-0.45081400	-0.00033200
H	0.68890800	-1.50571500	-0.00063300
C	2.40834200	-0.19465900	-0.00017000
C	2.96682900	1.09579000	-0.00027100
C	3.28118300	-1.29422200	0.00011100
C	4.34686100	1.27217200	-0.00002700
H	2.32361000	1.97056000	-0.00054800
C	4.66390200	-1.11767000	0.00033700
H	2.86434500	-2.29832100	0.00016400
C	5.20369900	0.16774900	0.00027600
H	4.75778500	2.27735400	-0.00010100
H	5.31779800	-1.98455800	0.00055000
H	6.27972800	0.31106600	0.00045200
O	-5.59911900	-0.34856000	0.00025100
H	-6.08044100	0.49206800	0.00096300

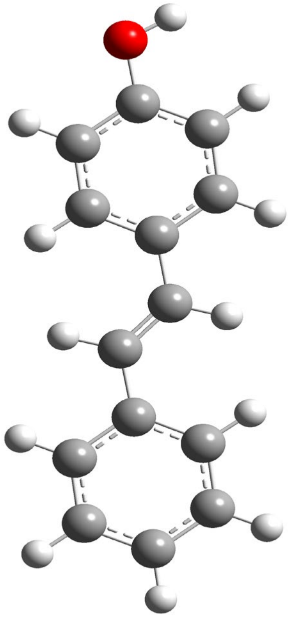

13-dmg

Charge=0, Multiplicity=2

C	2.39037600	1.36177500	-0.00024300
C	1.49664800	0.24533000	-0.00008500
C	2.06083900	-1.07169800	0.00015100
C	3.41002400	-1.25878600	0.00020100
C	4.32859200	-0.13214200	0.00003300
C	3.74432300	1.19601300	-0.00020100
H	1.96240200	2.36079000	-0.00042900
H	1.40743200	-1.93792100	0.00021300
H	3.84078000	-2.25554400	0.00039100
H	4.42062000	2.04545500	-0.00031700
O	5.57431900	-0.30357500	0.00006700
C	0.08946800	0.50454400	-0.00017000
H	-0.18748500	1.55618800	-0.00049500
C	-0.89035500	-0.44504500	0.00010000
H	-0.61560700	-1.49718400	0.00029600
C	-2.32487900	-0.19191000	0.00007400
C	-2.88354000	1.10140700	0.00033300
C	-3.19258600	-1.29943000	-0.00017100
C	-4.26228500	1.27136200	0.00025200
H	-2.24230000	1.97703700	0.00066900
C	-4.57326200	-1.12590200	-0.00026700
H	-2.76938900	-2.30050500	-0.00033700
C	-5.11257000	0.16080100	-0.00006500
H	-4.67954200	2.27346000	0.00045500
H	-5.22725300	-1.99217600	-0.00049500
H	-6.18896900	0.30109200	-0.00013800

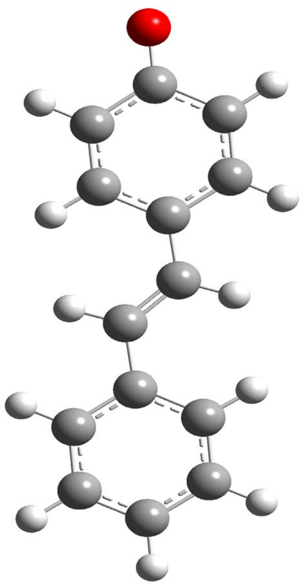

13 anion

Charge=-1, Multiplicity=1

C	-2.38601400	1.32884700	0.00000900
C	-1.50928600	0.22780600	-0.00000100
C	-2.09720400	-1.05396400	-0.00001200
C	-3.47135400	-1.22318000	-0.00001200
C	-4.37181800	-0.11350000	0.00000000
C	-3.76499800	1.17521000	0.00000900
H	-1.96337900	2.33249700	0.00001600
H	-1.46421900	-1.93844100	-0.00002300
H	-3.89633700	-2.22439500	-0.00002100
H	-4.41298700	2.04857400	0.00001700
O	-5.66424100	-0.27458700	0.00000000
C	-0.06648800	0.46317700	0.00000200
H	0.21195000	1.51729200	0.00001000
C	0.91013300	-0.46639100	-0.00000200
H	0.64411900	-1.52264200	-0.00000500
C	2.35607700	-0.20154100	-0.00000100
C	2.91088000	1.09190600	-0.00001300
C	3.23973300	-1.29428400	0.00001200
C	4.29001700	1.27664000	-0.00001000
H	2.26369400	1.96383100	-0.00002400
C	4.62116000	-1.10934500	0.00001400
H	2.83050500	-2.30163900	0.00002000
C	5.15532000	0.17881900	0.00000400
H	4.69370600	2.28495200	-0.00001900

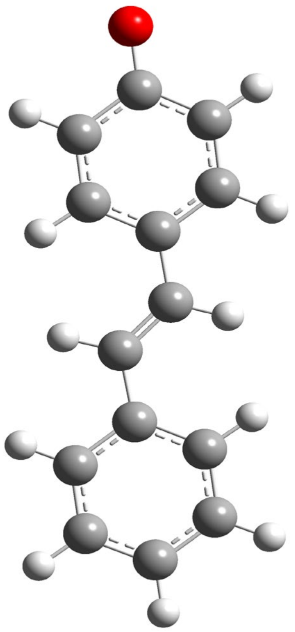

H	5.27948700	-1.97311200	0.00002400
H	6.23044500	0.32857900	0.00000500

14

Charge=0, Multiplicity=1

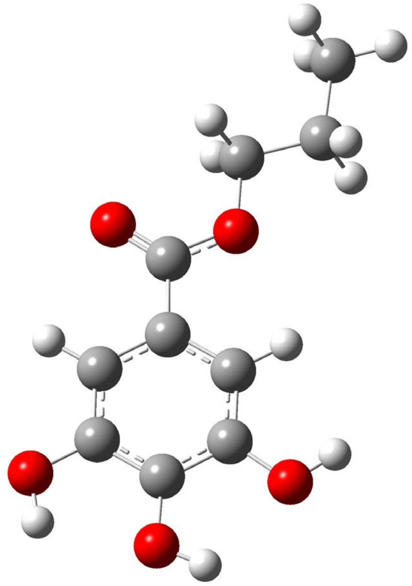

C	1.29098700	-1.40385300	0.00018100
C	2.61180600	-0.97580500	0.00055400
C	2.90946400	0.38870600	0.00003900
C	1.87300700	1.32591100	-0.00039200
C	0.54781500	0.91295800	-0.00073200
C	0.26470800	-0.45695000	-0.00053300
H	1.07203100	-2.46593000	0.00054000
H	-0.24368600	1.65460400	-0.00123600
O	3.61936700	-1.90119000	0.00148500
H	4.47974100	-1.45268200	0.00269000
O	4.22280200	0.75373300	0.00023300
H	4.29923600	1.72170400	-0.00064000
O	2.26936000	2.63577200	-0.00052400
H	1.49702700	3.22083200	0.00013500
C	-1.13664900	-0.94870300	-0.00092300
O	-1.44546400	-2.13056700	-0.00160000
O	-2.03889100	0.03299100	-0.00032700
C	-3.42851300	-0.35995700	-0.00062900
H	-3.61802800	-0.96882800	0.88883400
H	-3.61829200	-0.96644800	-0.89166400
C	-4.26186400	0.90343800	0.00124400
H	-4.00893100	1.50010400	-0.88178800
H	-4.00847700	1.49771700	0.88575100
C	-5.74988600	0.56422000	0.00116300
H	-6.01876100	-0.02141300	0.88610300
H	-6.35536600	1.47394900	0.00235900
H	-6.01914300	-0.01930900	-0.88505300

14(1)-dmg

Charge=0, Multiplicity=2

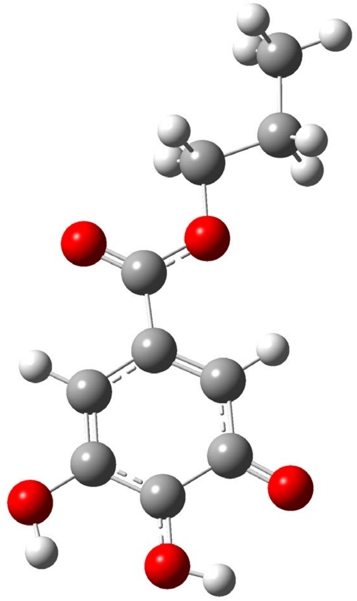

C	1.33166500	-1.33939900	-0.00078400
C	2.65345800	-0.94648500	-0.00621500
C	2.95448700	0.42207000	-0.00253500
C	1.90202600	1.42653000	0.00597400
C	0.53677400	0.96846500	0.01007900
C	0.28059500	-0.37694300	0.00692500
H	1.09199600	-2.39744900	-0.00350300
H	-0.25200900	1.71170000	0.01584400
O	3.64144300	-1.88649800	-0.01564500
H	4.51474600	-1.46350200	-0.02441500
O	4.22373900	0.81132800	-0.00740000
H	4.25480800	1.78872600	-0.00398400
O	2.24006300	2.63845700	0.00887200
C	-1.11704200	-0.90087700	0.01076600
O	-1.38751300	-2.08946600	0.01921600
O	-2.03384800	0.06010500	0.00315000
C	-3.41731300	-0.36064300	0.00500800
H	-3.59467600	-0.96005100	0.90318000
H	-3.59009900	-0.98335100	-0.87804000
C	-4.27404400	0.88645500	-0.01387100

H	-4.02931300	1.47465300	-0.90475900
H	-4.03501400	1.49795400	0.86276100
C	-5.75523100	0.51839900	-0.01385800
H	-6.01592800	-0.05969400	0.87842400
H	-6.37756700	1.41654500	-0.02729500
H	-6.01026600	-0.08237500	-0.89269400

14(2)-dmg

Charge=0, Multiplicity=2

C	1.33093500	-1.39861400	-0.00000900
C	2.64327700	-0.98536800	-0.00003000
C	2.98124000	0.42992500	-0.00001400
C	1.87930200	1.38043900	0.00002300
C	0.56971300	0.95257700	0.00003800
C	0.31270800	-0.42907500	0.00002400
H	1.08709300	-2.45461800	-0.00001900
H	-0.23695800	1.67534000	0.00006400
O	3.65705700	-1.86171500	-0.00006800
H	4.49945200	-1.37074400	-0.00008100
O	4.17295700	0.81660100	-0.00002900
O	2.19076800	2.68276200	0.00004300
H	3.16166600	2.77567000	0.00003300
C	-1.09507600	-0.93220200	0.00004500
O	-1.38166100	-2.11687500	0.00009100
O	-1.99778900	0.04141100	0.00000600
C	-3.38640800	-0.36320000	0.00002400
H	-3.56713500	-0.97277400	0.89066300
H	-3.56712400	-0.97289200	-0.89053600
C	-4.23040500	0.89237800	-0.00006600
H	-3.98314100	1.48979200	-0.88405400
H	-3.98315400	1.48991200	0.88384500
C	-5.71476100	0.53700800	-0.00005300
H	-5.97746800	-0.05030600	0.88559800
H	-6.32958600	1.44037000	-0.00011700
H	-5.97745600	-0.05042400	-0.88562900

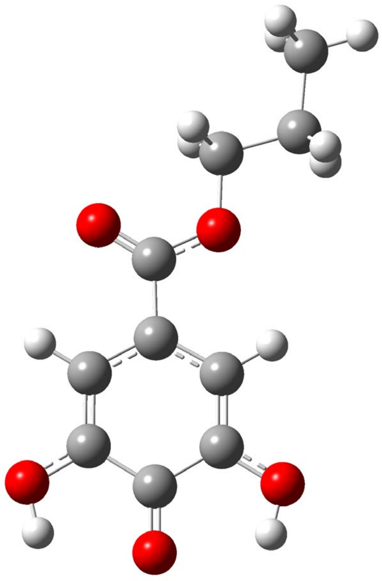

14[(3)]-dmg

Charge=0, Multiplicity=2

C	1.28837700	-1.45174900	-0.00160800
C	2.66788300	-1.04052300	-0.00384000
C	2.94650000	0.38766000	-0.00242800
C	1.90967700	1.32992700	0.00250400
C	0.60181400	0.89011800	0.00364300
C	0.30046400	-0.50366100	0.00160200
H	1.06414600	-2.51274100	-0.00232000
H	-0.19688200	1.62332800	0.00737800
O	3.64273900	-1.83613700	-0.00702700
O	4.20783000	0.80313400	-0.00488500
H	4.79611000	0.02202800	-0.00637100
O	2.17288700	2.66837100	0.00502400
H	3.12965800	2.82807600	0.02676300
C	-1.11794000	-0.97044300	0.00482400
O	-1.43875600	-2.14622100	0.01128500
O	-1.99462400	0.02779900	-0.00009900
C	-3.39364100	-0.33980500	0.00363500
H	-3.59021400	-0.93549400	0.90025900
H	-3.59055400	-0.95343700	-0.88068000
C	-4.20564700	0.93654800	-0.00923800
H	-3.94442800	1.51842800	-0.89955500
H	-3.94357600	1.53704800	0.86837300
C	-5.69815800	0.61703300	-0.00510900
H	-5.97427200	0.04556000	0.88682400
H	-6.29148200	1.53458900	-0.01472800
H	-5.97509000	0.02642400	-0.88422700

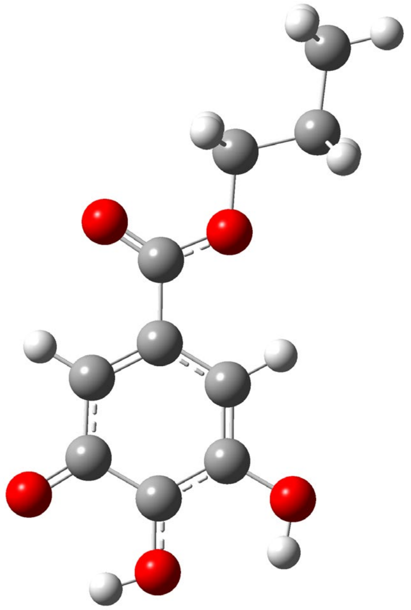

14[(1)] anion

Charge=-1, Multiplicity=1

C	0.56776000	0.94887500	0.00009800
C	1.89896900	1.41140400	0.00005600
C	2.91334900	0.41449900	-0.00001600
C	2.62899100	-0.94262600	-0.00004100
C	1.30665200	-1.38187300	0.00001400
C	0.28720200	-0.42536800	0.00007800
H	-0.23585200	1.67738900	0.00014300
H	1.08726800	-2.44349700	-0.00000900
O	2.26814000	2.66693000	0.00007000
O	4.22175900	0.82805300	-0.00007400
H	4.17457500	1.80381100	-0.00006100
O	3.64519600	-1.87390300	-0.00013000
H	4.49990400	-1.41641100	-0.00030800
C	-1.11054400	-0.92032000	0.00009500
O	-1.42506100	-2.10341700	0.00016800
O	-2.02403300	0.05597500	0.00000400
C	-3.40829900	-0.34864400	-0.00001500
H	-3.59520900	-0.95814400	0.88981600
H	-3.59516400	-0.95819000	-0.88982300
C	-4.25396100	0.90692300	-0.00007100
H	-4.00626100	1.50501800	-0.88366200
H	-4.00632200	1.50505500	0.88351100
C	-5.73888400	0.55434200	-0.00011800
H	-6.00266700	-0.03281200	0.88541300

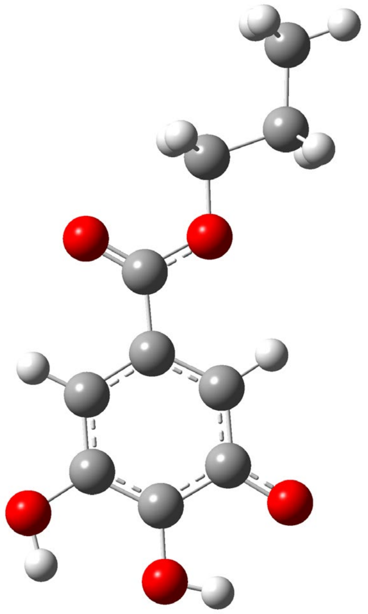

H	-6.35308600	1.45825500	-0.00017400
H	-6.00259500	-0.03287000	-0.88563200

14(2) anion

Charge=-1, Multiplicity=1

C	0.58228600	0.93087000	-0.00004100
C	1.90276400	1.33591700	-0.00006500
C	2.98459400	0.41844100	-0.00004900
C	2.63600500	-0.95678000	-0.00000500
C	1.32484000	-1.38933100	0.00001900
C	0.28426900	-0.44393600	0.00000100
H	-0.20628100	1.67576600	-0.00005600
H	1.10563900	-2.45242100	0.00005100
O	2.22746400	2.67644300	-0.00010900
H	3.20070500	2.71403600	-0.00012200
O	4.22227100	0.81548700	-0.00007200
O	3.67727700	-1.86091100	0.00001300
H	4.49238700	-1.32796300	-0.00000800
C	-1.10038700	-0.93083600	0.00002700
O	-1.42518100	-2.11492100	0.00005000
O	-2.01659600	0.05040600	0.00002500
C	-3.39971900	-0.35284300	0.00004800
H	-3.58953800	-0.96207800	0.88962300
H	-3.58956500	-0.96208600	-0.88951500
C	-4.24531400	0.90307500	0.00005600
H	-3.99749400	1.50130200	-0.88348900
H	-3.99747000	1.50130700	0.88359000
C	-5.73046300	0.55143700	0.00007800
H	-5.99448300	-0.03572400	0.88556100
H	-6.34451600	1.45548100	0.00008500
H	-5.99450900	-0.03572600	-0.88539600

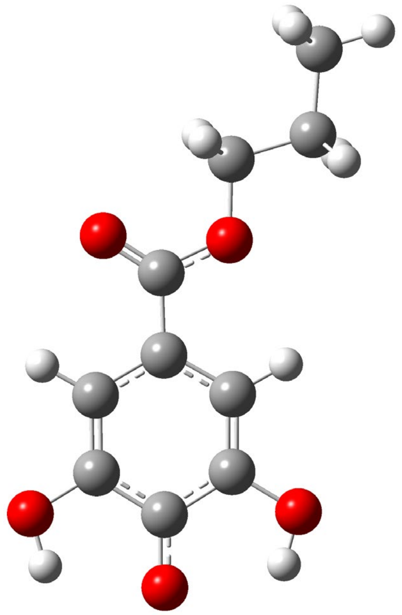

14(3) anion

Charge=-1, Multiplicity=1

C	0.57870700	0.91729900	-0.01602500
C	1.91599800	1.31148400	-0.00897700
C	2.92714600	0.36258900	0.00647200
C	2.66668700	-1.03578600	0.01283300
C	1.31005200	-1.41508600	0.00409000
C	0.29234500	-0.45175900	-0.00945400
H	-0.20212700	1.66826000	-0.02824900
H	1.05978600	-2.47129200	0.00856200
O	2.21536600	2.65736600	-0.01772600
H	3.17784000	2.77325500	-0.02875300
O	4.23545500	0.77669600	0.01460900
H	4.76041000	-0.04667400	0.02251900
O	3.68524300	-1.85675500	0.02637000
C	-1.11045800	-0.93136700	-0.01807200
O	-1.43612800	-2.11094600	-0.03603800
O	-2.01575400	0.05339400	-0.00276200
C	-3.40253800	-0.34308800	-0.00929600
H	-3.59036100	-0.97454400	0.86484700
H	-3.59483800	-0.92867600	-0.91396500
C	-4.24329700	0.91502800	0.02588600
H	-3.99714500	1.53529300	-0.84272200

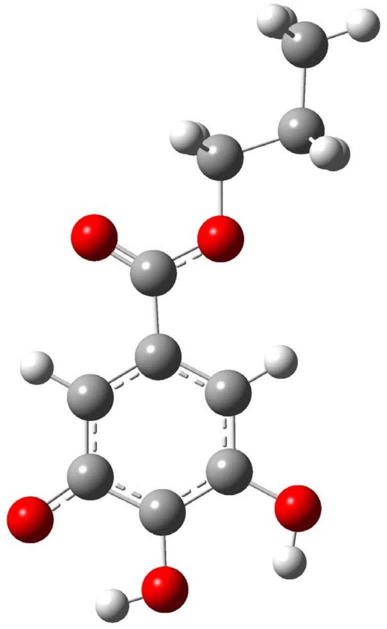

H	-3.99118000	1.48905500	0.92407000
C	-5.72906500	0.56558700	0.02182900
H	-5.99074700	-0.04409900	0.89266100
H	-6.34176200	1.47012200	0.04732300
H	-5.99680000	0.00184300	-0.87764200

15

Charge=0, Multiplicity=1

C	-4.08734500	-0.98040800	-0.41313100
C	-5.41338800	-0.67871500	-0.12109900
C	-5.81598300	0.65162200	0.03184400
C	-4.88313200	1.67112300	-0.11278900
C	-3.55501700	1.36395200	-0.40988200
C	-3.13926600	0.03736300	-0.56460100
H	-3.79840500	-2.02364600	-0.52439200
H	-5.21023800	2.70013600	0.00238400
H	-2.83463900	2.16952900	-0.52812800
O	-7.12494500	0.96106400	0.31834900
H	-7.64527000	0.14353500	0.35493400
O	-6.39680200	-1.62624800	0.03310200
H	-6.03466800	-2.51141400	-0.11908500
C	-1.69554600	-0.28358300	-0.87089700
H	-1.60894600	-1.34204700	-1.14812200
H	-1.38753100	0.30218700	-1.74245400
C	-0.73506000	-0.01397800	0.30731900
H	-0.90122200	1.01802100	0.65449300
C	0.73400000	-0.11027400	-0.16428700
H	0.83372200	-1.04027300	-0.74680000
C	1.71801100	-0.20075400	1.02142300
H	1.48527200	-1.08664200	1.61980000
H	1.57509300	0.67678400	1.66607700
C	3.16274900	-0.28063300	0.58867100
C	3.66290500	-1.45183400	0.01110300
C	4.01683200	0.82001600	0.71502300
C	4.98293400	-1.52435800	-0.43611500
H	3.01722300	-2.32026900	-0.09103300
C	5.33329200	0.75350100	0.27309400
H	3.66241600	1.74829900	1.15609100
C	5.81888700	-0.42162500	-0.30932300
H	5.37390400	-2.43432700	-0.88370200
O	6.14673400	1.85150100	0.41278600
H	7.02156000	1.65056700	0.04549700
O	7.13154500	-0.39577100	-0.72071800
H	7.36789300	-1.23901300	-1.13405600
C	-1.06504900	-0.96813500	1.45709400
H	-0.52855400	-0.71195600	2.37404800
H	-2.13545700	-0.94039500	1.68447200
H	-0.80671000	-2.00021600	1.18671400
C	1.10685300	1.07594100	-1.05829400
H	2.10253200	0.94911800	-1.49331300
H	1.11560500	2.00140600	-0.46830500
H	0.40410300	1.21091200	-1.88418900

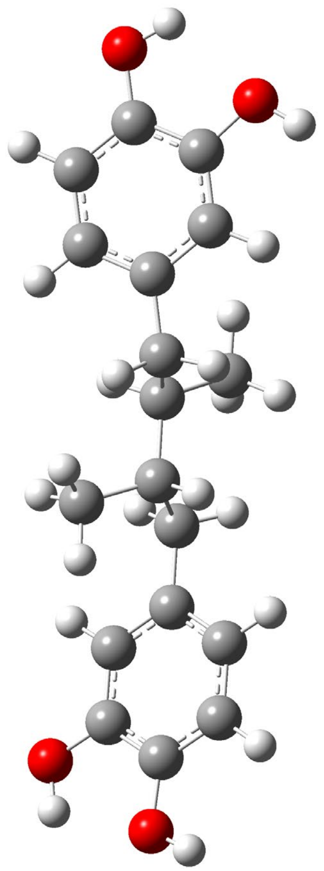

15$^{(1)}$-dmg

Charge=0, Multiplicity=2

C	-4.02934100	-1.00055800	-0.38256100
C	-5.35920100	-0.71109300	-0.09543600
C	-5.78099800	0.61659200	0.02488300
C	-4.86326700	1.64553900	-0.14728600
C	-3.53138100	1.35061300	-0.43979200
C	-3.09669800	0.02672700	-0.56209500
H	-3.72538200	-2.04198800	-0.46778600
H	-5.20516900	2.67221900	-0.05700300
H	-2.82331600	2.16351100	-0.57984900
O	-7.09362800	0.91461100	0.30656000
H	-7.60358400	0.09154400	0.36055900
O	-6.32726500	-1.66943500	0.08569600
H	-5.95230500	-2.55200500	-0.04971500
C	-1.64945000	-0.28191000	-0.86480300
H	-1.54735300	-1.34827300	-1.10366500
H	-1.35544900	0.27654100	-1.75877100
C	-0.68856600	0.04603400	0.29792800
H	-0.86022600	1.09114400	0.59982900
C	0.77968900	-0.06186200	-0.17330300
H	0.88525100	-1.02087300	-0.70475700
C	1.76048700	-0.07446100	1.01939500
H	1.54522700	-0.93452200	1.66160100
H	1.61032100	0.83407900	1.61586500
C	3.20138100	-0.16030100	0.59171200
C	3.66256600	-1.37751800	-0.01397400
C	4.07063800	0.88762000	0.73849200
C	4.96042800	-1.53615300	-0.46239100
H	2.96184600	-2.20201100	-0.11442900
C	5.43462700	0.78858000	0.29258200
H	3.75557800	1.82502900	1.18892500
C	5.84984400	-0.47284700	-0.31918400
H	5.29856100	-2.46228700	-0.91480600
O	6.28806600	1.71129600	0.39087000
O	7.11357300	-0.57199900	-0.73252600
H	7.56130400	0.27525400	-0.53951200
C	-1.00678200	-0.85999000	1.48907300
H	-0.46977000	-0.56035300	2.39245100
H	-2.07645300	-0.83067500	1.71907300
H	-0.74132600	-1.90069500	1.26211700
C	1.14612300	1.07789200	-1.12823500
H	2.13974300	0.93045800	-1.56191500
H	1.15642900	2.03222600	-0.58650200
H	0.43950200	1.16846500	-1.95654700

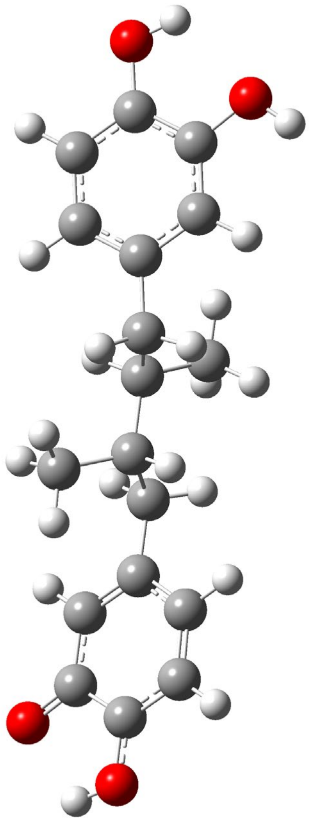

15(2)-dmg

Charge=0, Multiplicity=2

C	-4.02671000	-0.98929300	-0.38181900
C	-5.35342900	-0.68709500	-0.09377500
C	-5.76207900	0.64460700	0.02808000
C	-4.83444300	1.66468000	-0.14383800
C	-3.50567100	1.35695100	-0.43754300
C	-3.08421900	0.02897100	-0.56129700
H	-3.73298400	-2.03353700	-0.46836300
H	-5.16621300	2.69460900	-0.05276800
H	-2.78979600	2.16296800	-0.57769200
O	-7.07164400	0.95484100	0.31059400
H	-7.58891600	0.13635000	0.36488000
O	-6.33087000	-1.63604200	0.08617500
H	-5.96535700	-2.52182800	-0.05416600
C	-1.64051700	-0.29406500	-0.86580700
H	-1.54983700	-1.36051200	-1.10865300
H	-1.34075800	0.26470200	-1.75765700
C	-0.67580100	0.01894500	0.29793000
H	-0.83422100	1.06509200	0.60311600
C	0.78969100	-0.10565400	-0.17646300
H	0.88473000	-1.06369400	-0.71063400
C	1.77150100	-0.13623000	1.01880100
H	1.55225200	-1.00656500	1.64612300
H	1.62483400	0.76547000	1.62560000
C	3.20395000	-0.22716900	0.58326800
C	3.66522400	-1.43826000	-0.02599900
C	4.07441000	0.85082400	0.73081100
C	4.94697000	-1.56907400	-0.47935700
H	2.96727100	-2.26614700	-0.11935500
C	5.38153100	0.74269600	0.27889100
H	3.74057100	1.77735400	1.18854700
C	5.87617300	-0.47630900	-0.35394200
H	5.30350700	-2.48519500	-0.93965200
O	6.23969800	1.76111000	0.40335100
H	7.09323800	1.48293700	0.01851400
O	7.07339900	-0.50809100	-0.75045800
C	-1.00276500	-0.88714700	1.48640500
H	-0.45907400	-0.59775400	2.38923300
H	-2.07118400	-0.84399700	1.71977300
H	-0.75212600	-1.93046800	1.25487500
C	1.17194800	1.03399600	-1.12536600
H	2.16369400	0.87491300	-1.56003400
H	1.19438000	1.98559900	-0.57948900
H	0.46674400	1.13691000	-1.95337600

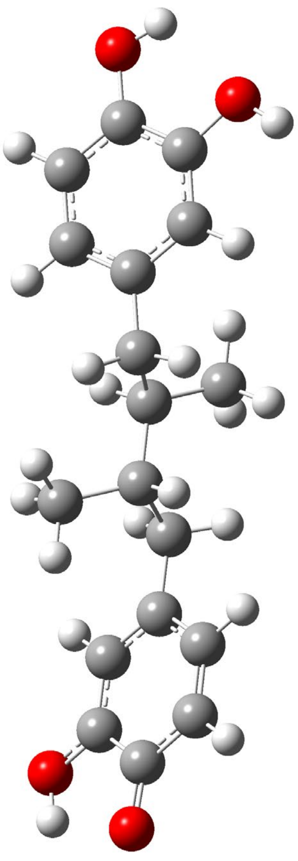

15$^{(3)}$-dmg

Charge=0, Multiplicity=2

C	-4.14075800	-1.01715500	-0.40530600
C	-5.44611400	-0.66337600	-0.09480200
C	-5.84794000	0.73227300	0.05341400
C	-4.82626500	1.72893800	-0.13681800
C	-3.54801900	1.35485200	-0.43925700
C	-3.18113100	-0.02234400	-0.57978000
H	-3.87941400	-2.06649400	-0.50620900
H	-5.11174600	2.77126600	-0.03500400
H	-2.77933000	2.10930500	-0.58626900
O	-7.05119500	0.98752800	0.33343100
O	-6.38861600	-1.59571300	0.08130400
H	-7.22626600	-1.13984600	0.29352200
C	-1.75397700	-0.36223200	-0.89180600
H	-1.67682400	-1.41920700	-1.17125400
H	-1.44555100	0.23537800	-1.75603200
C	-0.79792200	-0.09695200	0.29642600
H	-0.97825100	0.92526300	0.66324900
C	0.67005300	-0.16172400	-0.18339500
H	0.77957400	-1.07163900	-0.79450500
C	1.65463100	-0.27625400	0.99937100
H	1.43791900	-1.18755100	1.56473900
H	1.49442200	0.57430600	1.67515100
C	3.10121000	-0.31230700	0.56705300
C	3.62515000	-1.44961200	-0.05529800
C	3.93472600	0.79692400	0.74441200
C	4.94947600	-1.48100100	-0.49460600
H	2.99553800	-2.32403400	-0.19834800
C	5.25531700	0.77099800	0.31076100
H	3.56145900	1.69946000	1.22183500
C	5.76598300	-0.37114700	-0.31424300
H	5.35888400	-2.36538300	-0.97569300
O	6.04856400	1.87583900	0.50249400
H	6.93097700	1.70296400	0.13919100
O	7.08199500	-0.30770700	-0.71000900
H	7.34077400	-1.13598400	-1.13996800
C	-1.11615100	-1.07954300	1.42526800
H	-0.57785600	-0.83594700	2.34433500
H	-2.18552100	-1.06719600	1.65918900
H	-0.84888000	-2.10262100	1.13191300
C	1.02317400	1.05758500	-1.03935200
H	2.01610700	0.95526900	-1.48668000
H	1.02739800	1.96254400	-0.41849800
H	0.31193900	1.21303400	-1.85467300

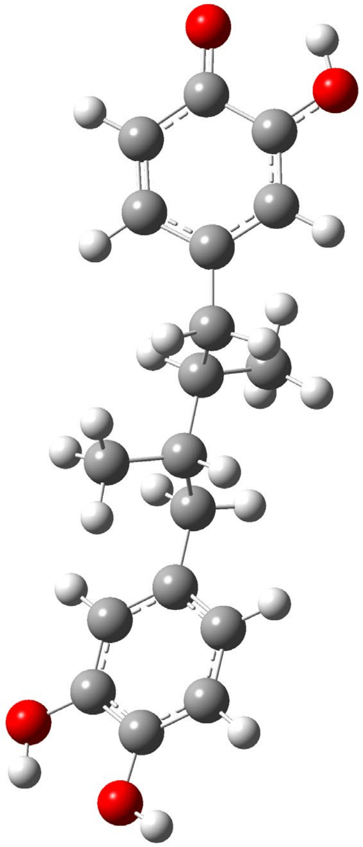

$15^{(4)}$-dmg

Charge=0, Multiplicity=2

C	-4.12402700	-1.05731300	-0.43321500
C	-5.49331400	-0.73412800	-0.13460400
C	-5.82717000	0.67883000	0.03413200
C	-4.85915100	1.67270800	-0.09729600
C	-3.56111400	1.29693000	-0.38973400
C	-3.17732300	-0.07578900	-0.56026800
H	-3.87007200	-2.10691800	-0.55470500
H	-5.13752500	2.71401700	0.02492000
H	-2.79921000	2.06366400	-0.50321900
O	-7.09540700	0.98257700	0.31225100
H	-7.60218400	0.14732600	0.35030700
O	-6.41737700	-1.58296900	-0.01011400
C	-1.73571500	-0.38778200	-0.86168000
H	-1.64087100	-1.44817800	-1.12431000
H	-1.43919900	0.19539600	-1.73958100
C	-0.78439000	-0.08719900	0.31784500
H	-0.97131500	0.94254300	0.66078300
C	0.68552600	-0.15419000	-0.15635700
H	0.80374600	-1.08482000	-0.73408700
C	1.67157100	-0.21807000	1.02925200
H	1.45442700	-1.10283400	1.63497500
H	1.51464200	0.66235300	1.66658400
C	3.11661200	-0.27818000	0.59438900
C	3.63619700	-1.44923000	0.03408800
C	3.95094800	0.83959200	0.70099200
C	4.95617700	-1.50511700	-0.41553000
H	3.00599700	-2.33065400	-0.05227300
C	5.26722900	0.78974700	0.25629600
H	3.58082700	1.76825800	1.12814200
C	5.77239900	-0.38569800	-0.30867600
H	5.36215800	-2.41494700	-0.84977600
O	6.06076500	1.90468900	0.37488600
H	6.93828100	1.71332200	0.00880600
O	7.08308500	-0.34325600	-0.72461300
H	7.33188400	-1.18833700	-1.12683200
C	-1.09438400	-1.04393600	1.47089600
H	-0.56596700	-0.76879100	2.38686300
H	-2.16536900	-1.04227700	1.69716500
H	-0.80970100	-2.07024300	1.20605600
C	1.03106200	1.03550100	-1.05623800
H	2.02953300	0.92874400	-1.49003400
H	1.01823800	1.96395500	-0.47116000
H	0.32634800	1.15004500	-1.88366500

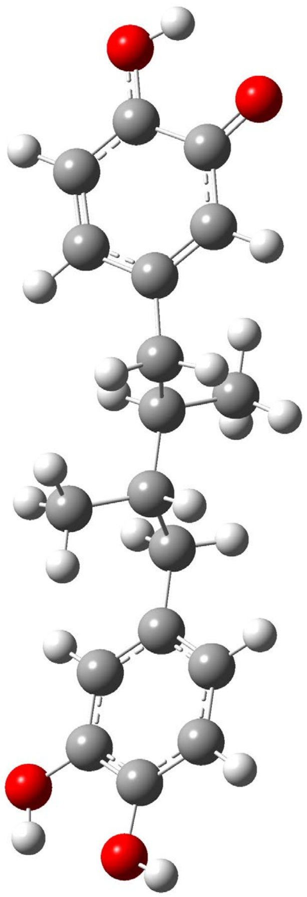

15(1) anion

Charge=-1, Multiplicity=1

C	-4.06358100	-0.98997800	-0.39589400
C	-5.37636800	-0.66313700	-0.07465800
C	-5.75362400	0.67718100	0.05430100
C	-4.81183900	1.68021100	-0.14147500
C	-3.49618600	1.34574600	-0.46390700
C	-3.10481800	0.00921400	-0.59519100
H	-3.79648800	-2.03981100	-0.48838600
H	-5.11817500	2.71838400	-0.04330400
H	-2.76713300	2.13734600	-0.61832500
O	-7.07228600	0.90840000	0.37065600
H	-7.23390600	1.86040300	0.44316100
O	-6.29370400	-1.66845800	0.11356600
H	-7.15486300	-1.27791700	0.32909300
C	-1.67056100	-0.33557700	-0.91888100
H	-1.60650500	-1.38697600	-1.22717900
H	-1.35289400	0.26971400	-1.77334900
C	-0.70251200	-0.11993300	0.26512400
H	-0.87788100	0.89021600	0.66820700
C	0.76553700	-0.17198900	-0.21680000
H	0.87373800	-1.06497800	-0.85337900
C	1.75484000	-0.31638600	0.95872200
H	1.53950500	-1.24522700	1.49647400
H	1.58369200	0.51115800	1.66116000
C	3.20733700	-0.32626000	0.53509600
C	3.73530200	-1.43534800	-0.13045800
C	4.03009700	0.78381300	0.77675200
C	5.07432500	-1.43193600	-0.55285500
H	3.11131100	-2.30536300	-0.31989200
C	5.38055500	0.82631400	0.37235300
H	3.62406300	1.65202200	1.29402200
C	5.86759300	-0.32778600	-0.30582400
H	5.50009300	-2.28894400	-1.06889400
O	6.20052900	1.83492800	0.57407200
O	7.19191200	-0.29751600	-0.70818700
H	7.51696600	0.57200400	-0.40774100
C	-1.01627700	-1.13783700	1.36385100
H	-0.48009800	-0.92390800	2.29169800
H	-2.08630200	-1.13734800	1.59503800
H	-0.74483900	-2.15033700	1.03813900
C	1.11697300	1.06864100	-1.04264500
H	2.11248200	0.98008500	-1.48720700
H	1.11704400	1.95842100	-0.39982900
H	0.40782600	1.24248900	-1.85604600

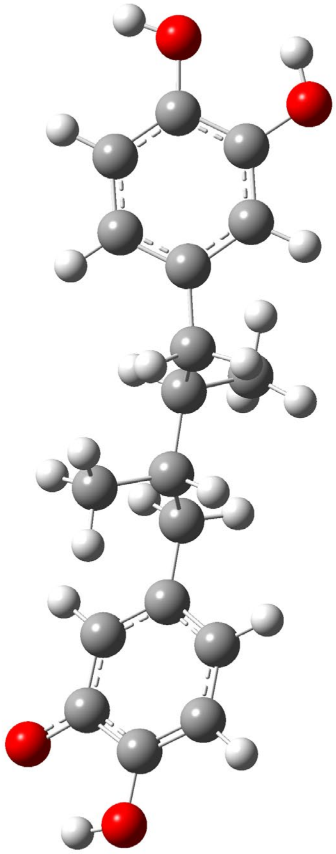

15(2) anion

Charge=-1, Multiplicity=1

C	-4.06426200	-0.98161200	-0.39950200
C	-5.37584000	-0.65127700	-0.07698900
C	-5.74881900	0.68996500	0.05491400
C	-4.80395800	1.69051800	-0.13863500
C	-3.48938300	1.35251800	-0.46178200
C	-3.10230500	0.01506400	-0.59644000
H	-3.80068500	-2.03206800	-0.49505900
H	-5.10706700	2.72943200	-0.03832100
H	-2.75762500	2.14199900	-0.61448500
O	-7.06676900	0.92451400	0.37167200
H	-7.22599500	1.87694200	0.44370000
O	-6.29648100	-1.65394800	0.10918200
H	-7.15622300	-1.26093800	0.32591500
C	-1.66900100	-0.33295700	-0.92031800
H	-1.60986700	-1.37874900	-1.24798000
H	-1.34320900	0.28602800	-1.76195200
C	-0.70416900	-0.14603600	0.27139400
H	-0.88489600	0.85178100	0.70199500
C	0.76433200	-0.17668700	-0.21126100
H	0.87359400	-1.04059600	-0.88695800
C	1.75701600	-0.37302500	0.95398700
H	1.53886400	-1.32264000	1.45310600
H	1.58825100	0.42414300	1.69176500
C	3.20768300	-0.36630500	0.52964700
C	3.76376400	-1.45265800	-0.14806800
C	4.03061800	0.74844700	0.76850300
C	5.09588300	-1.44444000	-0.58248200
H	3.14836500	-2.32992700	-0.34163900
C	5.34695200	0.75265700	0.34158800
H	3.63975900	1.61913300	1.29198400
C	5.93865700	-0.33966500	-0.35391500
H	5.50661400	-2.30618600	-1.10405600
O	6.16205400	1.84311200	0.58143700
H	7.02164200	1.59364300	0.19188200
O	7.20175400	-0.24965200	-0.72046500
C	-1.01623100	-1.19473400	1.34120400
H	-0.48063400	-1.00573000	2.27481900
H	-2.08640200	-1.20218000	1.57185000
H	-0.74323900	-2.19743000	0.98784300
C	1.11188900	1.09995200	-0.98207900
H	2.10220600	1.03028400	-1.44129200
H	1.12124100	1.95853600	-0.29812900
H	0.39387900	1.31395800	-1.77819000

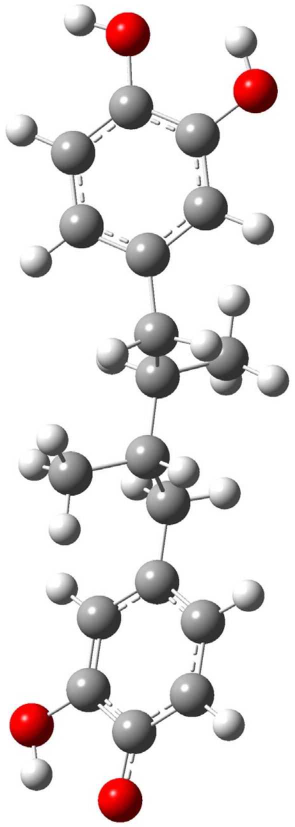

15(3) anion

Charge=-1, Multiplicity=1

C	-4.13039300	-0.96376000	-0.40043800
C	-5.44415000	-0.65661900	-0.09279200
C	-5.91200600	0.67935600	0.05802300
C	-4.94659000	1.68796100	-0.12724500
C	-3.61661500	1.37979400	-0.44102700
C	-3.18358000	0.05982500	-0.58363500
H	-3.83882500	-2.00772600	-0.50116000
H	-5.25930700	2.72508200	-0.02766100
H	-2.90312100	2.19046200	-0.58217200
O	-7.18297100	0.87695500	0.34482300
O	-6.38065000	-1.65843100	0.08245600
H	-7.20848300	-1.18164700	0.28435800
C	-1.73906900	-0.25402600	-0.89904500
H	-1.65675600	-1.29606500	-1.23696700
H	-1.41514500	0.37532000	-1.73435400
C	-0.77580300	-0.06509500	0.29366300
H	-0.94927000	0.93825200	0.71454300
C	0.69422900	-0.11518700	-0.18261200
H	0.79973500	-0.99440400	-0.83867800
C	1.67922200	-0.29452900	0.99194500
H	1.45242100	-1.22762500	1.51631400
H	1.52900200	0.52653700	1.70566300
C	3.12553100	-0.32856600	0.55879200
C	3.64206700	-1.45319900	-0.09215600
C	3.96671000	0.77048700	0.76283500
C	4.96633100	-1.48290100	-0.53208100
H	3.00647200	-2.31948400	-0.25680500
C	5.28668400	0.74689700	0.32743100
H	3.59962100	1.66347700	1.26256600
C	5.79020100	-0.38336500	-0.32446000
H	5.36979400	-2.35819400	-1.03437100
O	6.08699800	1.84211400	0.54461500
H	6.96668100	1.67410200	0.17257600
O	7.10730800	-0.31966100	-0.71731900
H	7.36155700	-1.14047000	-1.16395100
C	-1.09700300	-1.10122300	1.37272100
H	-0.56461700	-0.90708200	2.30732700
H	-2.16821700	-1.09964700	1.59729100
H	-0.82847700	-2.10908200	1.02994600
C	1.05922900	1.14195200	-0.97758100
H	2.05189700	1.05491300	-1.42912000
H	1.07039800	2.01509800	-0.31252000
H	0.34918000	1.34355400	-1.78344500

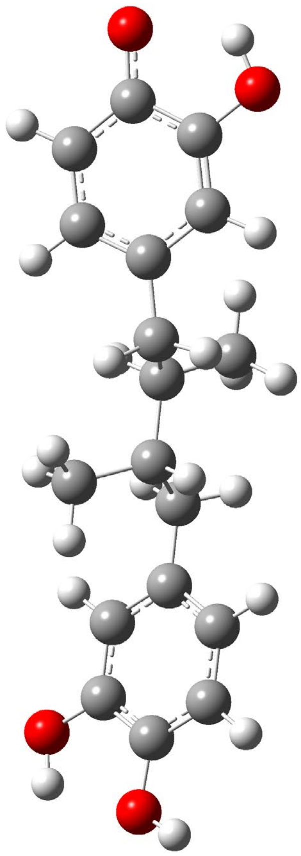

15[(4)] anion

Charge=-1, Multiplicity=1

C	-4.12858400	-1.00705200	-0.40942800
C	-5.48191600	-0.74963600	-0.10836300
C	-5.84372400	0.62066800	0.03071500
C	-4.93173500	1.64747900	-0.12288300
C	-3.59390400	1.34876900	-0.42619800
C	-3.18620100	0.01986300	-0.56978100
H	-3.81726800	-2.04501700	-0.51888300
H	-5.26362600	2.67678200	-0.01170900
H	-2.87575700	2.15531000	-0.55346100
O	-7.17046800	0.88037800	0.32658600
H	-7.58410700	-0.00280800	0.37147900
O	-6.41306100	-1.66591500	0.04773200
C	-1.73925500	-0.30079400	-0.87575200
H	-1.64999500	-1.36113100	-1.14737600
H	-1.42816300	0.27878100	-1.75093700
C	-0.77373400	-0.02647700	0.29743500
H	-0.93809200	1.00738900	0.64005600
C	0.69525800	-0.12472100	-0.17453800
H	0.79463900	-1.05510100	-0.75662400
C	1.67977900	-0.21418400	1.01086200
H	1.45058300	-1.10283500	1.60662300
H	1.53210600	0.66058900	1.65825200
C	3.12567700	-0.28516300	0.58058600
C	3.63122200	-1.44810200	-0.00899700
C	3.97650700	0.81597400	0.72332900
C	4.95335400	-1.51206700	-0.45120900
H	2.98824200	-2.31686400	-0.12439400
C	5.29516500	0.75778800	0.28667900
H	3.61809300	1.73792600	1.17433600
C	5.78629800	-0.40910900	-0.30728300
H	5.34830500	-2.41579400	-0.90785100
O	6.10553300	1.85582900	0.44372600
H	6.98310200	1.66039000	0.08010700
O	7.10109000	-0.37565300	-0.71156900
H	7.34208400	-1.21446700	-1.13121400
C	-1.10120100	-0.97293300	1.45431900
H	-0.56101300	-0.71353200	2.36836400
H	-2.17097200	-0.94100800	1.68325500
H	-0.84670500	-2.00744100	1.18900100
C	1.06832700	1.06071900	-1.06959900
H	2.06254700	0.93248300	-1.50776900
H	1.08045400	1.98614500	-0.47947900
H	0.36279100	1.19697600	-1.89292700

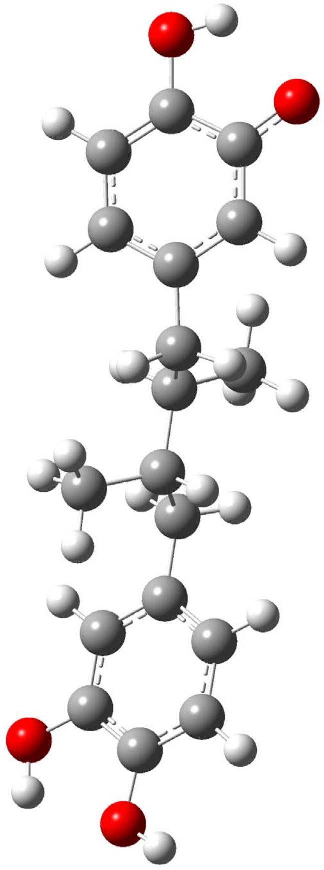

16(1)-dmg

Charge=0, Multiplicity=2

C	-0.26150400	-0.88378600	-0.00938300
C	-0.36663700	0.53411300	0.00407300
C	-1.69431800	1.22304800	-0.03398600
H	-1.87807900	1.67207700	0.95270300
H	-1.64687300	2.05616400	-0.74244600
C	-2.81375200	0.24792800	-0.38876900
H	-2.80927400	0.03475800	-1.46300800
H	-3.78681600	0.67372900	-0.13268200
O	-1.37470400	-1.67218200	-0.02453700
C	0.95997700	-1.51071100	-0.04106800
H	1.02792400	-2.59321800	-0.06366900
C	2.17756500	-0.73329700	-0.03548400
C	0.84077000	1.29757100	0.04510900
C	-2.60909300	-1.04501900	0.37088500
H	-3.38141400	-1.78268100	0.15400600
H	-2.57413300	-0.86493900	1.45266800
C	2.07372500	0.70767900	0.01897500
H	2.98392000	1.29906100	0.03931500
O	0.65981400	2.64545800	0.10108900
H	1.51751000	3.09667400	0.12333400
O	3.30324600	-1.30037400	-0.06434500

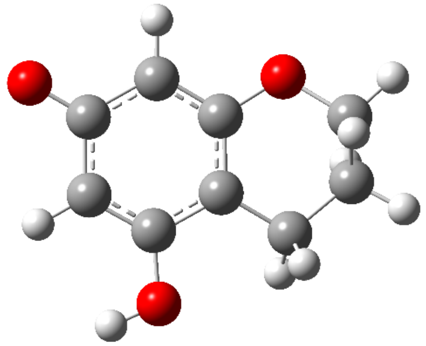

16(2)-dmg

Charge=0, Multiplicity=2

C	-0.26948800	-0.78675800	-0.00098300
C	-0.46356700	0.58061700	0.02248800
C	-1.83561900	1.18604700	-0.00685100
H	-2.07391000	1.59554900	0.98350800
H	-1.84344600	2.03104100	-0.70196400
C	-2.86622700	0.13106300	-0.40520200
H	-2.81306500	-0.06660200	-1.48141500
H	-3.87908300	0.47068800	-0.17474600
O	-1.29581200	-1.68739300	-0.00639500
C	1.02162600	-1.34702800	-0.03818800
H	1.13121300	-2.42729100	-0.06832600
C	2.16605900	-0.51388100	-0.03070500
C	0.71666600	1.43982700	0.05169700
C	-2.58682600	-1.15572300	0.34581200
H	-3.29528800	-1.94592200	0.09721700
H	-2.59985900	-0.98505100	1.42961600
C	2.03534900	0.84795500	0.02215300
H	2.90127900	1.50071500	0.04000800
O	0.57856400	2.68951300	0.09752800
O	3.41659800	-1.05728200	-0.06423800
H	3.36951600	-2.02454200	-0.10039900

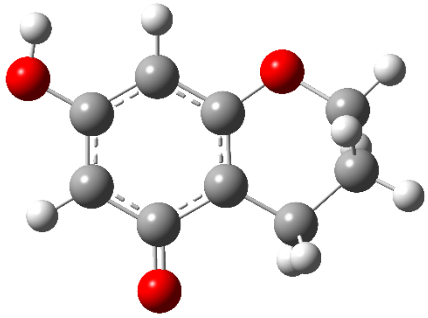

16(1) anion

Charge=-1, Multiplicity=1

C	-0.24501300	-0.84445200	-0.01333600
C	-0.40033500	0.54797400	0.01646300
C	-1.75512600	1.21575300	-0.00397100
H	-1.98755900	1.64101900	0.98174600
H	-1.75062900	2.05305200	-0.70964900
C	-2.83171500	0.20222600	-0.38764000
H	-2.79956400	-0.00274600	-1.46412600
H	-3.82976000	0.57936600	-0.14721700
O	-1.34184000	-1.68586300	-0.03531500
C	0.99960600	-1.47009200	-0.04143800
H	1.04874600	-2.55514500	-0.06156100
C	2.19721800	-0.71002200	-0.03250600
C	0.79176400	1.28732900	0.04034500
C	-2.58621200	-1.09357900	0.36011600
H	-3.34495900	-1.84713300	0.14328000
H	-2.56109700	-0.91439900	1.44390500
C	2.05146700	0.70551100	0.01563800
H	2.93896300	1.33380800	0.03574500
O	0.64449000	2.66029800	0.08711100
H	1.51857900	3.07605800	0.08695500
O	3.37701800	-1.27540600	-0.05568400

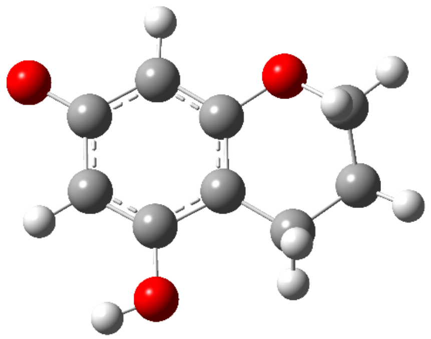

16(2) anion

Charge=-1, Multiplicity=1

C	-0.23724300	-0.79912600	-0.01270500
C	-0.44143400	0.57783900	0.01301400
C	-1.82792200	1.17327200	-0.02146600
H	-2.07909000	1.61066900	0.95492300
H	-1.85125100	2.00116500	-0.73872300
C	-2.86384000	0.11372500	-0.39148100
H	-2.82699100	-0.10093500	-1.46609000
H	-3.87698600	0.44948800	-0.15195900
O	-1.28908400	-1.69681900	-0.02629400
C	1.03785100	-1.38145200	-0.03967400
H	1.15282700	-2.46053700	-0.05561000
C	2.13541100	-0.52571900	-0.03016400
C	0.69988800	1.44585400	0.04936200
C	-2.56011000	-1.16334200	0.36599400
H	-3.28175200	-1.95457500	0.15634300
H	-2.54182400	-0.97451000	1.44833600
C	1.99064800	0.85594800	0.01701000
H	2.87021800	1.49310400	0.03394000
O	0.54411100	2.74094000	0.10670400
O	3.42506800	-1.02576300	-0.05468600
H	3.39460000	-1.99271400	-0.08627400

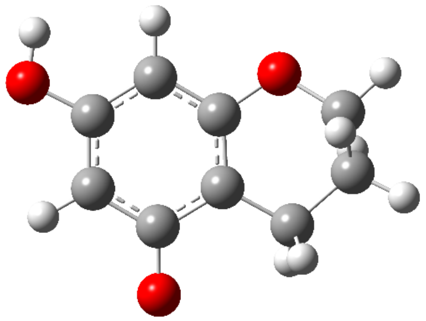

17(1)-dmg

Charge=0, Multiplicity=2

C	0.00000000	2.03838600	0.00000200
C	1.23294300	1.36789700	0.00000200
C	1.24053700	-0.01082200	-0.00000500
C	0.00000000	-0.76617700	-0.00002300
C	-1.24053700	-0.01082200	-0.00000100
C	-1.23294300	1.36789700	0.00000700
H	0.00000000	3.12343900	0.00001100
H	2.16841400	1.91568900	0.00000900
H	-2.16841400	1.91568900	0.00001600
O	2.38819700	-0.70922500	-0.00000100
H	2.17476600	-1.66014000	-0.00001200
O	-2.38819700	-0.70922500	0.00000700
H	-2.17476600	-1.66014000	-0.00000500
O	0.00000000	-2.02563600	0.00000600

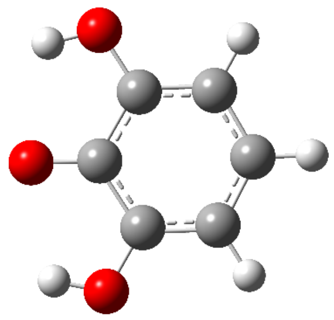

17(2)-dmg

Charge=0, Multiplicity=2

C	-0.37170900	2.03961600	0.00002700
C	0.95698200	1.52227900	0.00013000
C	1.18829100	0.16027800	0.00008400
C	0.09321500	-0.71086500	0.00002500
C	-1.27536300	-0.21176000	0.00005600
C	-1.46285900	1.21734500	-0.00006400
H	-0.50144600	3.11664000	-0.00000300
H	1.80619200	2.19770900	0.00017500
H	-2.47814300	1.59863500	-0.00020300
O	2.46753800	-0.32026800	0.00010100
H	2.45782700	-1.29034200	0.00003600
O	-2.20740100	-1.05885700	-0.00026700
O	0.30343100	-2.02683600	-0.00002400
H	-0.56431700	-2.47631000	-0.00002300

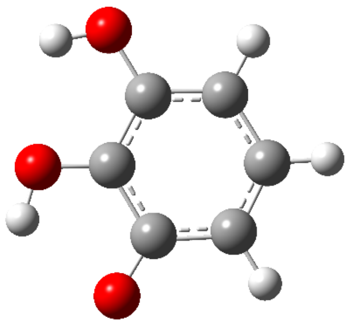

17(1) anion

Charge=-1, Multiplicity=1

C	0.00000000	2.08175500	0.00000800
C	1.21176100	1.38361600	0.00000300
C	1.19577900	-0.00685400	-0.00000100
C	0.00000000	-0.75599800	0.00000000
C	-1.19577900	-0.00685400	0.00000300
C	-1.21176100	1.38361600	0.00000700
H	0.00000000	3.16683100	0.00001200
H	2.16412100	1.90586900	0.00000300
H	-2.16412100	1.90586900	0.00001000
O	2.37996000	-0.72119900	-0.00000600
H	2.11783600	-1.65923900	-0.00000800
O	-2.37996000	-0.72119900	0.00000200
H	-2.11783600	-1.65923900	0.00000000
O	0.00000000	-2.07457400	-0.00001200

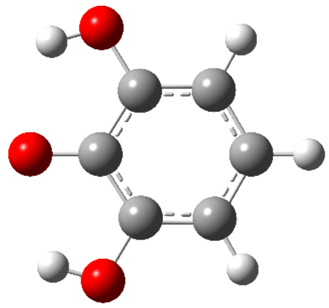

17$^{(2)}$ anion

Charge=-1, Multiplicity=1

C	-0.06586800	2.07614600	0.00001300
C	1.16721800	1.42435100	0.00007600
C	1.17861600	0.02554500	0.00007700
C	-0.00937100	-0.68562000	-0.00001400
C	-1.27841600	-0.04866000	-0.00014100
C	-1.26867400	1.36519200	-0.00008300
H	-0.09029300	3.16244400	0.00004400
H	2.10650500	1.96721900	0.00014900
H	-2.22084400	1.88892300	-0.00011600
O	2.39209000	-0.63537900	0.00015900
H	2.23205600	-1.59111000	0.00012700
O	-2.35512600	-0.79989300	-0.00010800
O	0.03023800	-2.06821200	-0.00000900
H	-0.90608300	-2.34133400	-0.00010200

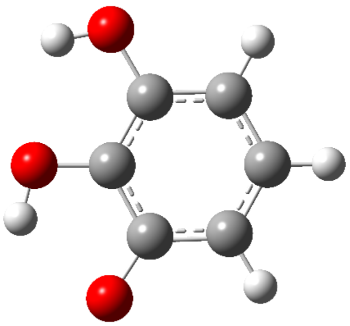

18$^{(1)}$-dmg

Charge=0, Multiplicity=2

C	0.23721700	-1.27307500	-0.00006100
C	1.60757300	-1.12175200	-0.00003800
C	2.14791800	0.17098200	-0.00002200
C	1.29342800	1.34781000	-0.00005000
C	-0.13234800	1.14063700	-0.00000500
C	-0.62444100	-0.13791700	-0.00003200
H	-0.18710300	-2.27141900	-0.00007200
H	-0.77579400	2.01268400	0.00003700
O	1.84362300	2.47956400	0.00007400
O	3.46691000	0.32356700	0.00000800
H	3.67453500	1.27882100	-0.00000300
O	2.41436500	-2.22027200	-0.00003500
H	3.34634200	-1.95140500	-0.00002200
C	-2.09203700	-0.40633900	-0.00003000
O	-2.57025600	-1.52715000	-0.00002000
O	-2.82682100	0.70206400	0.00004600
C	-4.25290000	0.51503500	0.00009600
H	-4.55579100	-0.03027800	0.89541300
H	-4.67535700	1.51735500	0.00014500
H	-4.55586200	-0.03021900	-0.89523400

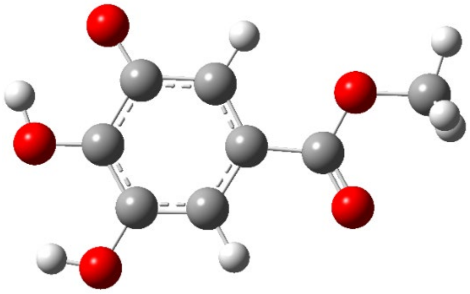

18$^{(2)}$-dmg

Charge=0, Multiplicity=2

C	0.22173200	-1.33645400	-0.00015900
C	1.58836200	-1.17521700	-0.00021600
C	2.18621700	0.15195900	0.00000100
C	1.28208700	1.29221500	0.00022800
C	-0.08428400	1.11565700	0.00028000
C	-0.59618100	-0.19331800	0.00009800
H	-0.21462800	-2.32849300	-0.00031200
H	-0.74114200	1.97669900	0.00046200
O	1.82610800	2.51613800	0.00038600
H	2.79751000	2.43373600	0.00030900
O	3.42972800	0.30432100	-0.00009800
O	2.42060600	-2.22478900	-0.00046700
H	3.33869500	-1.89642700	-0.00046500

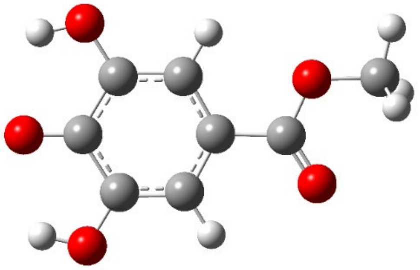

C	-2.07289200	-0.42136700	0.00016400
O	-2.58215800	-1.52820800	-0.00004300
O	-2.77517500	0.70784300	0.00003900
C	-4.20600800	0.56332500	-0.00009100
H	-4.52446600	0.02680500	0.89520100
H	-4.59873500	1.57759400	-0.00009700
H	-4.52430900	0.02685800	-0.89547100

18(3)-dmg

Charge=0, Multiplicity=2

C	-0.16181300	-1.37421400	0.00027800
C	-1.59562700	-1.24177800	0.00017000
C	-2.14842400	0.10393700	-0.00004700
C	-1.31811800	1.23184200	-0.00017400
C	0.05035200	1.05816800	-0.00007800
C	0.61954000	-0.24938400	0.00014700
H	0.26706600	-2.37025900	0.00046100
H	0.68912300	1.93397400	-0.00016600
O	-1.84240100	2.49051900	-0.00040700
H	-2.81148200	2.44876300	-0.00029500
O	-3.46601300	0.26459300	-0.00012200
H	-3.88939900	-0.61660200	-0.00001800
O	-2.39801700	-2.21094900	0.00032900
C	2.10182000	-0.42315100	0.00025500
O	2.65591400	-1.50822300	-0.00001900
O	2.75939200	0.73344800	0.00011300
C	4.19509700	0.64800000	-0.00024200
H	4.53528400	0.12510000	-0.89550500
H	4.54567900	1.67762100	-0.00071800
H	4.53574700	0.12577400	0.89524200

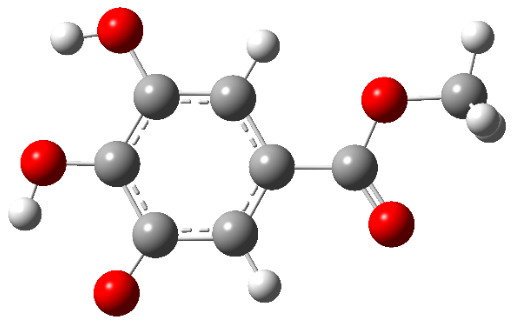

18(1) anion

Charge=-1, Multiplicity=1

C	0.19972400	-1.30698400	-0.00003800
C	1.58031400	-1.12104500	-0.00006800
C	2.11199300	0.15961300	-0.00000200
C	1.30122500	1.32803300	0.00011600
C	-0.09319300	1.12095400	0.00010000
C	-0.62401900	-0.17736200	0.00003200
H	-0.21373900	-2.30909100	-0.00009400
H	-0.74714800	1.98631300	0.00013000
O	1.89783300	2.49218700	0.00002900
O	3.47519000	0.31857400	-0.00003300
H	3.61583400	1.28494200	0.00005200
O	2.40653400	-2.22409200	-0.00016000
H	3.32980000	-1.92904000	-0.00017100
C	-2.08820700	-0.40680500	0.00001700
O	-2.61669800	-1.51054500	-0.00007100
O	-2.80798900	0.72190100	0.00008000
C	-4.23431500	0.56349000	0.00004600
H	-4.55295500	0.02479400	0.89434900
H	-4.63896100	1.57361300	0.00010200
H	-4.55292600	0.02490400	-0.89433400

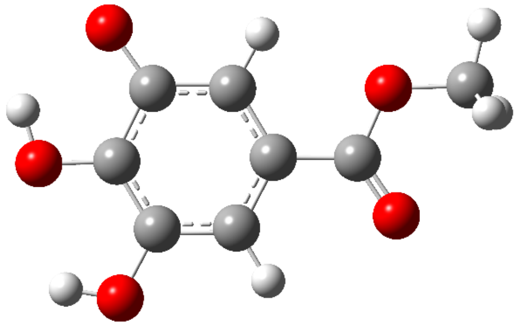

18[(2)] anion

Charge=-1, Multiplicity=1

C	0.21633000	-1.32134800	-0.00021000
C	1.58491800	-1.14121800	-0.00020300
C	2.18417300	0.14517700	0.00002300
C	1.29330500	1.24914200	0.00024800
C	-0.07954200	1.09746100	0.00023600
C	-0.62929600	-0.19764400	0.00000500
H	-0.19864100	-2.32442700	-0.00039700
H	-0.71491700	1.97662600	0.00040500
O	1.86149800	2.50567000	0.00047900
H	2.82497000	2.36362000	0.00043800
O	3.47451100	0.30095300	0.00005100
O	2.44021700	-2.22258800	-0.00042700
H	3.33984700	-1.84891800	-0.00039200
C	-2.07931700	-0.41749300	-0.00005800
O	-2.62212800	-1.51861300	-0.00016200
O	-2.79688300	0.71962900	0.00004200
C	-4.22271500	0.56971000	-0.00001500
H	-4.54717500	0.03362500	0.89394600
H	-4.62180500	1.58231100	0.00020700
H	-4.54714200	0.03403100	-0.89423200

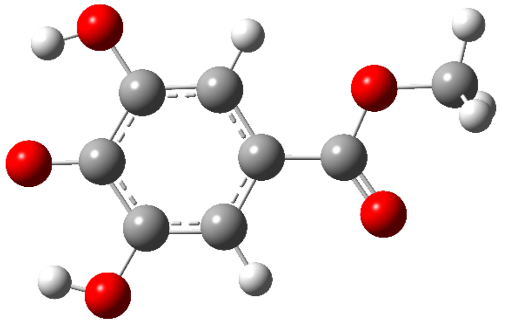

18[(3)] anion

Charge=-1, Multiplicity=1

C	-0.19943300	-1.34574500	0.00024800
C	-1.60266900	-1.22230500	0.00024900
C	-2.11630300	0.10448300	0.00001200
C	-1.29918200	1.22413000	-0.00021500
C	0.08776500	1.08313800	-0.00023600
C	0.62203700	-0.20954200	0.00000200
H	0.24302400	-2.33698200	0.00045600
H	0.71643600	1.96534400	-0.00042200
O	-1.84309300	2.49065100	-0.00042600
H	-2.80991700	2.42108400	-0.00025900
O	-3.47885000	0.26577300	-0.00000800
H	-3.83637000	-0.64346000	0.00015500
O	-2.45796800	-2.21144200	0.00057400
C	2.08929800	-0.41736000	-0.00001100
O	2.63466900	-1.51245100	-0.00000700
O	2.79172700	0.72312000	-0.00001000
C	4.22027400	0.58858000	-0.00012500
H	4.54809000	0.05544500	-0.89446700
H	4.60788100	1.60538800	-0.00030700
H	4.54825300	0.05570800	0.89431600

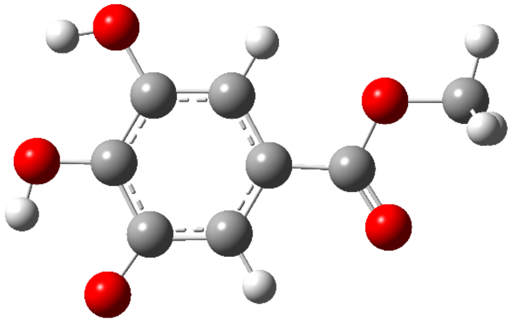

19

Charge=0, Multiplicity=1

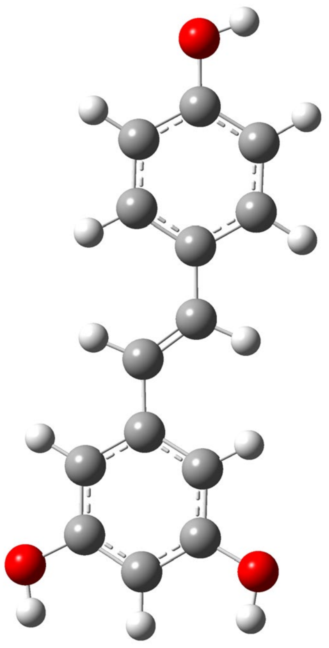

C	-4.94198000	-0.13424600	-0.00627700
C	-4.10219900	-1.22914600	0.21861800
C	-2.72557800	-1.05416100	0.21687000
C	-2.15217700	0.20971800	-0.01164800
C	-3.01827200	1.29041100	-0.23249700
C	-4.40119400	1.13015800	-0.23287800
H	-4.54023700	-2.20596300	0.39855400
H	-2.09318100	-1.91713400	0.40205800
H	-2.59925100	2.27789300	-0.40793500
H	-5.05988400	1.97669000	-0.40643900
O	-6.29425300	-0.36024400	0.01044800
H	-6.77001600	0.47164400	-0.13124000
C	-0.70300900	0.45187800	-0.02776200
H	-0.42309700	1.49891200	-0.13678700
C	0.26025000	-0.48068300	0.06443600
H	-0.01046600	-1.53220700	0.14119300
C	1.70960200	-0.22276600	0.04679200
C	2.25162000	1.06974900	0.12040900
C	2.56760200	-1.32589000	-0.05163100
C	3.63201900	1.23561500	0.08189500
H	1.62327200	1.94877800	0.21553100
C	3.94706600	-1.13187400	-0.09088700
H	2.16893500	-2.33424300	-0.10538100
C	4.49818300	0.14530500	-0.02608500
H	5.57497900	0.29070100	-0.05344600
O	4.11359600	2.51847800	0.15899600
H	5.08165100	2.51337000	0.12984900
O	4.73642700	-2.24833400	-0.19809900
H	5.66952600	-1.99205200	-0.23285300

19(1)-dmg

Charge=0, Multiplicity=2

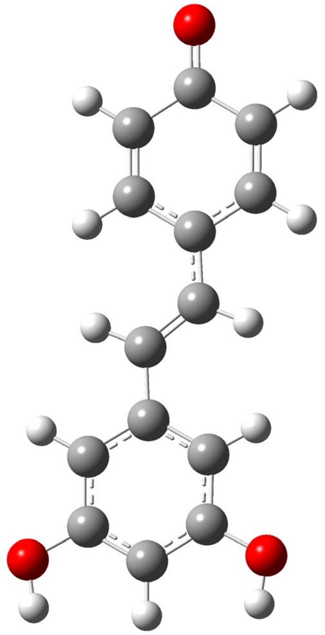

C	5.02012500	-0.15462300	-0.00002500
C	4.10054100	-1.28054800	-0.00052900
C	2.75164400	-1.09148900	-0.00047000
C	2.18990800	0.22633100	0.00008100
C	3.08383400	1.34199700	0.00056900
C	4.43772000	1.17457700	0.00054700
H	4.53022300	-2.27773700	-0.00096300
H	2.09708200	-1.95675200	-0.00087800
H	2.65676900	2.34132500	0.00098700
H	5.11537100	2.02288600	0.00093600
O	6.26540800	-0.32733200	-0.00009000
C	0.78130900	0.48639000	0.00015000
H	0.50381300	1.53793500	0.00047900
C	-0.19562000	-0.46355800	-0.00011200
H	0.07782300	-1.51560000	-0.00034300
C	-1.63298700	-0.21030600	-0.00005600
C	-2.17869200	1.08478600	-0.00024900
C	-2.48249100	-1.32700400	0.00015500
C	-3.56002300	1.23792400	-0.00020500
H	-1.55533000	1.97193500	-0.00049000
C	-3.86303500	-1.14446000	0.00022200

H	-2.07399100	-2.33265100	0.00027000
C	-4.41699100	0.13386000	0.00006200
H	-5.49508200	0.27211200	0.00012400
O	-4.05232600	2.51645000	-0.00043800
H	-5.02090300	2.50265800	-0.00053900
O	-4.64612100	-2.26751500	0.00044000
H	-5.58291900	-2.02219900	0.00028500

19^(2)-dmg

Charge=0, Multiplicity=2

C	4.90427000	-0.15321300	0.00112300
C	4.05770400	-1.19873600	-0.38028200
C	2.68303600	-1.01264300	-0.36354500
C	2.12003000	0.21225200	0.03682500
C	2.99183000	1.24548600	0.40923000
C	4.37298900	1.07327300	0.39738400
H	4.48967400	-2.14363900	-0.69456000
H	2.04328500	-1.83198000	-0.67716900
H	2.57899000	2.20316400	0.71551100
H	5.03778900	1.88164100	0.68893700
O	6.25360700	-0.38707900	-0.04086100
H	6.73698300	0.40882500	0.22635100
C	0.67336700	0.45987800	0.07527500
H	0.39479500	1.49294400	0.28167900
C	-0.29384800	-0.45659400	-0.09778300
H	-0.03907700	-1.50386900	-0.24713300
C	-1.73629700	-0.16619900	-0.07102200
C	-2.26724200	1.10495500	-0.21619200
C	-2.62663700	-1.24837300	0.09841200
C	-3.69837100	1.32907900	-0.16191000
H	-1.64200000	1.97460900	-0.39178700
C	-4.03217700	-1.05391800	0.15104600
H	-2.24291700	-2.25950900	0.19702200
C	-4.57461100	0.19639600	0.02871300
H	-5.64704000	0.36136000	0.06683300
O	-4.16236800	2.49471800	-0.28593900
O	-4.77120400	-2.18370700	0.33017100
H	-5.71502500	-1.96486700	0.36371200

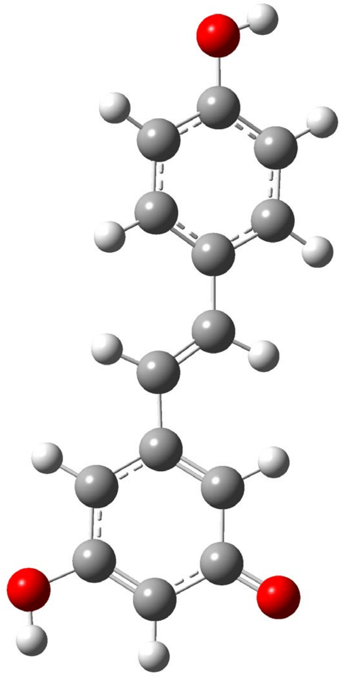

19(3)-dmg

Charge=0, Multiplicity=2

C	4.90019800	-0.09977300	-0.00001800
C	4.08006700	-1.23262900	-0.00008200
C	2.70057600	-1.08495000	-0.00005700
C	2.10576700	0.18950600	0.00003600
C	2.95145200	1.30828900	0.00009700
C	4.33671700	1.17518200	0.00007000
H	4.53562400	-2.21787400	-0.00015400
H	2.08336500	-1.97812700	-0.00012200
H	2.51418100	2.30341900	0.00016400
H	4.97987800	2.05079900	0.00011600
O	6.25525400	-0.30079700	-0.00005000
H	6.71786700	0.55051200	-0.00003200
C	0.65414200	0.40803700	0.00005200
H	0.36355100	1.45711400	0.00007800
C	-0.29428600	-0.54437300	0.00003300
H	-0.01295200	-1.59528500	0.00003600
C	-1.74484000	-0.30784900	0.00001700
C	-2.30177200	0.99620800	-0.00001800
C	-2.60554300	-1.38815900	0.00001700
C	-3.70461100	1.19502500	-0.00004000
H	-1.67292600	1.87992500	-0.00003400
C	-4.04423100	-1.21016200	0.00001600
H	-2.22724000	-2.40621400	0.00003100
C	-4.57237600	0.13413000	-0.00004600
H	-5.64864000	0.27689000	-0.00006400
O	-4.10839300	2.49672100	-0.00006700
H	-5.07637900	2.55098500	-0.00009600
O	-4.81234600	-2.20880500	0.00006800

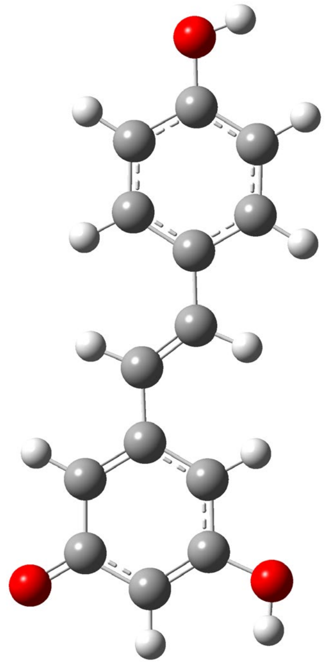

19(1) anion

Charge=-1, Multiplicity=1

C	-5.06663300	-0.13587900	-0.00008500
C	-4.16590800	-1.24580400	0.00038900
C	-2.79225300	-1.07622100	0.00045500
C	-2.20479900	0.20607400	0.00003300
C	-3.08154500	1.30733600	-0.00039900
C	-4.46014800	1.15338200	-0.00045400
H	-4.59086700	-2.24699700	0.00072300
H	-2.15898800	-1.96044200	0.00084600
H	-2.65880400	2.31090100	-0.00070100
H	-5.10863100	2.02632500	-0.00079800
O	-6.35835800	-0.29723600	-0.00015700
C	-0.76287200	0.44070900	0.00000500
H	-0.48254200	1.49431200	-0.00009700
C	0.21209300	-0.49061500	0.00005500
H	-0.05331300	-1.54647500	0.00004200
C	1.65768000	-0.22516000	0.00006700
C	2.19929800	1.07152700	0.00046300
C	2.52604900	-1.32683400	-0.00032800
C	3.57944700	1.23972400	0.00040300
H	1.56965800	1.95470900	0.00082500
C	3.90512800	-1.13014600	-0.00037700
H	2.13336700	-2.33910400	-0.00061700
C	4.45371100	0.15016500	-0.00002900

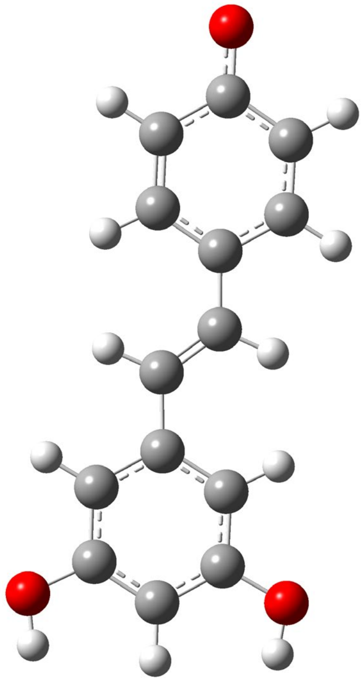

H	5.53019700	0.29892300	-0.00005600
O	4.05539700	2.52830300	0.00079500
H	5.02375400	2.52501700	0.00078300
O	4.69895600	-2.25010400	-0.00080200
H	5.63271500	-1.99443400	-0.00082300

19[(2)] anion

Charge=-1, Multiplicity=1

C	-4.90680100	-0.15830700	-0.00006100
C	-4.04875300	-1.26180600	-0.00009500
C	-2.67441100	-1.06780400	0.00002000
C	-2.11959800	0.22486200	0.00016600
C	-3.00445300	1.31291400	0.00023100
C	-4.38560300	1.13388800	0.00011700
H	-4.47029700	-2.26224500	-0.00021300
H	-2.02868300	-1.94066700	-0.00000500
H	-2.60212300	2.32273400	0.00035700
H	-5.05666800	1.98846600	0.00015700
O	-6.25652600	-0.40649600	-0.00021100
H	-6.74699100	0.42868800	-0.00026900
C	-0.67304400	0.48893300	0.00022400
H	-0.40910000	1.54563400	0.00051900
C	0.30349800	-0.43456500	-0.00008900
H	0.03810500	-1.49099800	-0.00037600
C	1.75503600	-0.17286800	-0.00006700
C	2.29673100	1.11784700	-0.00003400
C	2.60402800	-1.29190800	-0.00007900
C	3.70019000	1.34065300	0.00000400
H	1.65147400	1.99204900	-0.00006300
C	3.98450800	-1.08716700	-0.00003700
H	2.20303000	-2.30073400	-0.00011200
C	4.53618400	0.18622400	0.00000700
H	5.61661700	0.31280500	0.00003000
O	4.21008600	2.54376200	0.00002600
O	4.78222700	-2.21595400	-0.00004500
H	5.71325900	-1.95162000	-0.00002400

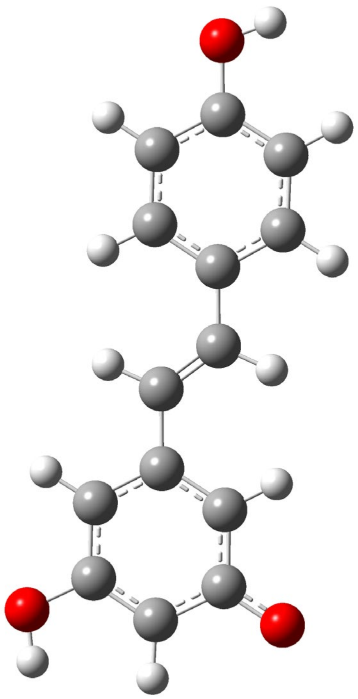

19[(3)] anion

Charge=-1, Multiplicity=1

C	4.90408400	-0.11974200	0.00130900
C	4.06687600	-1.23915900	-0.00161200
C	2.68900200	-1.07089500	-0.00364400
C	2.11033900	0.21112700	-0.00279500
C	2.97456800	1.31568300	-0.00064300
C	4.35867000	1.16242800	0.00137000
H	4.50701200	-2.23155000	-0.00193000
H	2.05943700	-1.95549800	-0.00563400
H	2.55326100	2.31776600	-0.00020000
H	5.01374300	2.02932600	0.00332200
O	6.25833200	-0.34160200	0.00436600
H	6.73261800	0.50298800	0.00892900
C	0.65946500	0.44906200	-0.00318900
H	0.37872600	1.50140800	-0.00366200
C	-0.30288300	-0.48931900	-0.00211100
H	-0.02431200	-1.54225700	-0.00145800
C	-1.75661700	-0.24364800	-0.00118300
C	-2.30039700	1.05428900	-0.00103100
C	-2.60238600	-1.35642200	-0.00005600
C	-3.68648500	1.18689600	0.00034900
H	-1.68123600	1.94452900	-0.00170700
C	-4.02154100	-1.23386000	0.00135400
H	-2.17381300	-2.35632300	-0.00013600
C	-4.54119600	0.08815700	0.00152200
H	-5.61863200	0.23864000	0.00265100
O	-4.19066900	2.47440700	0.00059900
H	-5.15810500	2.44062100	0.00178600
O	-4.80487400	-2.27995900	0.00256000

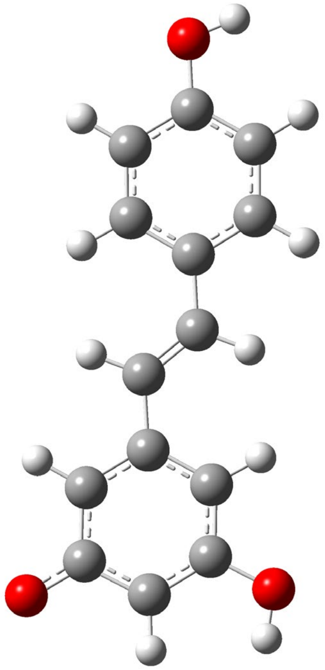

20[(1)]-dmg

Charge=0, Multiplicity=2

C	-4.62361600	-0.52776900	-0.02539900
C	-3.70703000	-1.57819500	-0.10522600
C	-2.35888000	-1.29155500	-0.09300800
C	-1.86667300	0.05798500	0.00080600
C	-2.76648400	1.09661800	0.08069000
C	-4.18084300	0.86131700	0.07022200
H	-4.06791500	-2.59851500	-0.17710100
H	-1.65670500	-2.11497400	-0.15741900
H	-2.42796500	2.12636000	0.15186500
O	-5.06040900	1.76289500	0.13621600
O	-5.93283400	-0.75867600	-0.03612500
H	-6.39193800	0.10291700	0.02290500
C	-0.43217700	0.36038900	0.01132400
H	-0.19744700	1.42125900	0.07226000
C	2.89492100	-1.31022000	0.03019200
C	4.26799300	-1.07200400	0.05675900
C	4.77402900	0.22476900	0.01730100
C	3.87192300	1.28932200	-0.04904000
C	2.49742700	1.07902100	-0.07305200
C	2.00213400	-0.23285800	-0.02988000
H	2.52928200	-2.33184300	0.06176300
H	5.84579200	0.40534500	0.03590400
H	1.83969700	1.93944000	-0.13002000

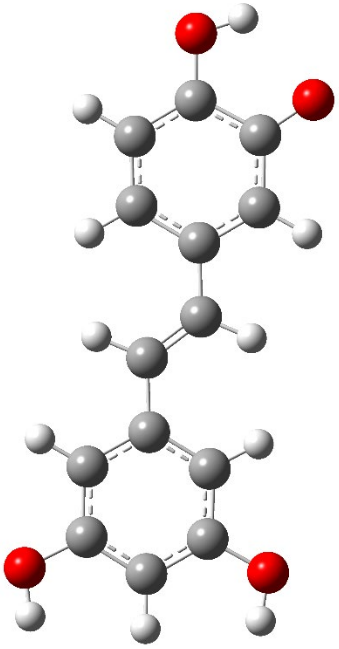

C	0.56360700	-0.54175600	-0.04184000
H	0.33531100	-1.60429300	-0.08818100
O	4.30922700	2.58830500	-0.09500100
H	5.27702000	2.61709400	-0.07252600
O	5.09367700	-2.16400700	0.12414900
H	6.01958400	-1.88131000	0.14754300

20[(2)]-dmg

Charge=0, Multiplicity=2

C	-4.65434200	-0.56748500	-0.03325900
C	-3.68061500	-1.62652000	-0.13968400
C	-2.34575200	-1.35196200	-0.13110900
C	-1.86041800	-0.00474600	-0.01399500
C	-2.78606300	1.05347200	0.08925600
C	-4.13696700	0.79385200	0.08062600
H	-4.05152800	-2.64264700	-0.22896100
H	-1.63916900	-2.17002800	-0.21698500
H	-2.43685000	2.07801100	0.17666100
O	-5.03950900	1.78449700	0.17922500
H	-5.92761300	1.38007100	0.15629800
O	-5.89907300	-0.74564200	-0.03390000
C	-0.45259900	0.31928800	0.00063600
H	-0.22685500	1.38173800	0.06085000
C	2.90090900	-1.30866400	0.04736600
C	4.26904400	-1.04661300	0.07598100
C	4.75123400	0.25912000	0.02088500
C	3.83281100	1.30881100	-0.06295800
C	2.46281500	1.07551500	-0.08985300
C	1.99037900	-0.24547600	-0.03024900
H	2.55115800	-2.33528700	0.09105300
H	5.81975000	0.45855900	0.04166000
H	1.79101800	1.92364800	-0.16138600
C	0.56377700	-0.57626200	-0.04356100
H	0.34666900	-1.64119200	-0.08039700
O	4.24955800	2.61296000	-0.12462000
H	5.21661400	2.65822500	-0.09569100
O	5.11360400	-2.12196500	0.16097600
H	6.03489800	-1.82386200	0.18295000

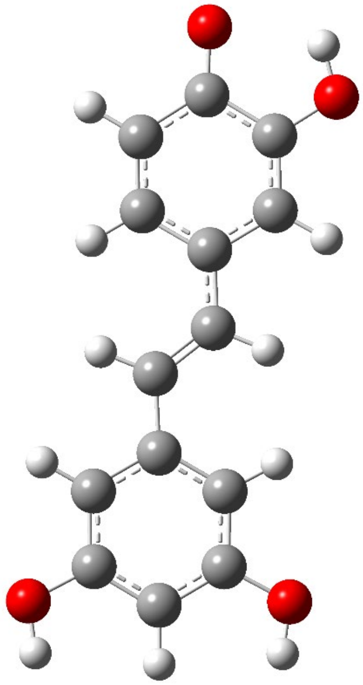

20(3)-dmg

Charge=0, Multiplicity=2

C	-4.54789300	-0.50899000	-0.09637200
C	-3.64808100	-1.51222300	-0.44535000
C	-2.27935700	-1.26172400	-0.43268100
C	-1.78841200	-0.00082500	-0.06295100
C	-2.70670300	1.00460900	0.27802900
C	-4.07139300	0.75668300	0.26526700
H	-4.03271300	-2.48636100	-0.73470200
H	-1.59911100	-2.05514600	-0.72394800
H	-2.35935600	1.99453400	0.56133200
O	-4.93955600	1.76163100	0.60819200
H	-5.85112900	1.43739100	0.54279600
O	-5.90879100	-0.66681800	-0.08611300
H	-6.14845900	-1.56125000	-0.37074000
C	-0.35557500	0.32250000	-0.01784100
H	-0.12926800	1.37822200	0.12812200
C	3.01586900	-1.22614500	0.17746700
C	4.40951000	-0.96222200	0.24683800
C	4.89704800	0.30189900	0.05410800
C	3.97469000	1.37671900	-0.23056500
C	2.55684600	1.08211800	-0.29874700
C	2.08161200	-0.20061900	-0.08080000
H	2.67654700	-2.24590900	0.33363700
H	5.95964500	0.51927900	0.10339500
H	1.89623000	1.90782400	-0.54321300
C	0.65429000	-0.55784100	-0.11494500
H	0.44758700	-1.62297000	-0.19903000
O	4.38692500	2.55216500	-0.42407600
O	5.19382000	-2.04306800	0.51439700
H	6.12615100	-1.78051900	0.55440300

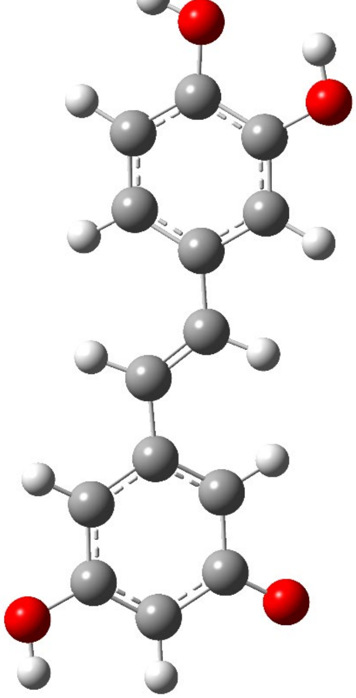

20(4)-dmg

Charge=0, Multiplicity=2

C	4.54855100	-0.48678000	0.00004700
C	3.66843200	-1.56483600	-0.00000600
C	2.29402300	-1.34834300	-0.00005700
C	1.77729500	-0.04403400	-0.00008300
C	2.67646700	1.03443700	-0.00005900
C	4.04663400	0.81972000	0.00001200
H	4.07286700	-2.57333000	0.00001400
H	1.63082100	-2.20681400	-0.00008000
H	2.30923700	2.05713600	-0.00007000
O	4.89762500	1.89524100	0.00004900
H	5.81412500	1.57813800	0.00024900
O	5.91352300	-0.60709100	0.00009500
H	6.17183500	-1.54082400	0.00063800
C	0.33889600	0.25730000	-0.00010000
H	0.10493300	1.32042800	-0.00014300
C	-3.00758300	-1.37180300	0.00003400
C	-4.43568500	-1.12169500	0.00009900
C	-4.89544900	0.24726200	0.00001700
C	-3.97531100	1.26293200	-0.00004100
C	-2.58417100	0.99413400	-0.00004900
C	-2.09388600	-0.33591100	-0.00002200

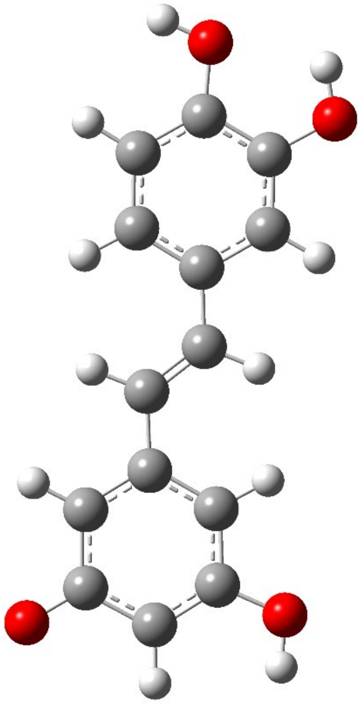

H	-2.68118800	-2.40760600	0.00004800
H	-5.96316900	0.44458500	0.00001400
H	-1.91133300	1.84476000	-0.00009400
C	-0.65687800	-0.64499500	-0.00004200
H	-0.42847100	-1.70874500	0.00000700
O	-4.31410900	2.58290300	-0.00009400
H	-5.27836900	2.68402600	-0.00008300
O	-5.25320200	-2.08056300	0.00007300

20[(1)] anion

Charge=-1, Multiplicity=1

C	-4.61695500	-0.49188500	-0.05627400
C	-3.74439700	-1.54254700	-0.28041700
C	-2.36320800	-1.31663200	-0.28245000
C	-1.86830000	-0.02581200	-0.05146200
C	-2.77434800	1.03021300	0.16782700
C	-4.16853800	0.84084200	0.17545100
H	-4.14518900	-2.53655500	-0.46078700
H	-1.68974000	-2.14605300	-0.47218900
H	-2.39201900	2.03457600	0.34096200
O	-5.06361100	1.78099800	0.37482700
O	-5.98103400	-0.69145400	-0.05432200
H	-6.35725600	0.19323400	0.11706700
C	-0.43096000	0.28707800	-0.02681000
H	-0.20164600	1.35089900	0.03037300
C	2.93364500	-1.30947900	0.11143500
C	4.30006600	-1.03731400	0.14667900
C	4.78023500	0.26502700	0.03406500
C	3.85431400	1.29924200	-0.11901600
C	2.48612300	1.05507300	-0.15881900
C	2.01466800	-0.26156000	-0.03554200
H	2.59190400	-2.33588100	0.20348000
H	5.84706700	0.47223200	0.05987800
H	1.81141100	1.89381800	-0.29157800
C	0.58150400	-0.59758700	-0.05225500
H	0.36481400	-1.66452400	-0.07134500
O	4.26481600	2.60330900	-0.24307400
H	5.23097800	2.65235100	-0.19918400
O	5.15077200	-2.10360500	0.29744500
H	6.06903900	-1.79604100	0.30983900

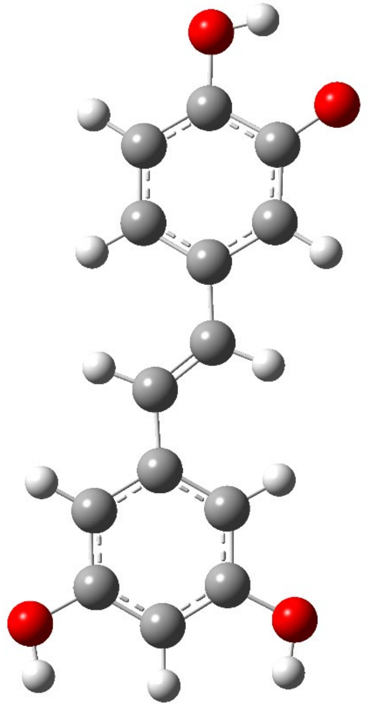

20(2) anion

Charge=-1, Multiplicity=1

C	-4.69181100	-0.53722800	-0.06198400
C	-3.75474600	-1.57798200	-0.25750300
C	-2.38179600	-1.34151900	-0.24604600
C	-1.86324700	-0.05474300	-0.03311200
C	-2.78240100	1.00137900	0.16090900
C	-4.13951300	0.76239500	0.14588000
H	-4.13434600	-2.58264400	-0.42724900
H	-1.70710900	-2.17746500	-0.41073700
H	-2.42183300	2.01486700	0.32423700
O	-5.04181300	1.79009500	0.33628500
H	-5.91768200	1.36296800	0.28438000
O	-5.99089500	-0.67824500	-0.06365600
C	-0.43291300	0.25165200	-0.00243800
H	-0.20429800	1.31416600	0.08357500
C	2.95094600	-1.31031900	0.10061600
C	4.31334800	-1.02016900	0.13692900
C	4.77782200	0.28885500	0.03197000
C	3.83740600	1.31082500	-0.11525100
C	2.47274700	1.04900300	-0.15636300
C	2.01599200	-0.27423200	-0.03942300
H	2.62352200	-2.34191100	0.18724600
H	5.84178000	0.50997700	0.05812500
H	1.78813600	1.87990800	-0.28775800
C	0.58832300	-0.62589300	-0.05889400
H	0.38150300	-1.69381300	-0.10730900
O	4.22958500	2.62157000	-0.23384400
H	5.19508300	2.68374700	-0.19460000
O	5.17835800	-2.07655900	0.28141400
H	6.09243400	-1.75683500	0.29676100

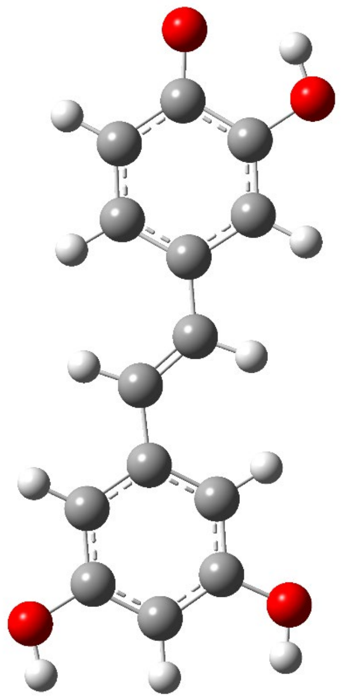

20(3) anion

Charge=-1, Multiplicity=1

C	-4.54535600	-0.51200500	-0.08346300
C	-3.64345600	-1.52175300	-0.40526500
C	-2.27481200	-1.26682700	-0.40469600
C	-1.78350200	0.00500100	-0.07330000
C	-2.70578000	1.01601000	0.24017900
C	-4.07021500	0.76393900	0.23914000
H	-4.02563400	-2.50532600	-0.66492100
H	-1.59387000	-2.06700300	-0.67532000
H	-2.36153100	2.01499600	0.49424400
O	-4.94062500	1.77687500	0.55545300
H	-5.85033900	1.44350100	0.51571800
O	-5.90781800	-0.67332800	-0.06438400
H	-6.14370200	-1.57798900	-0.31762400
C	-0.34948700	0.33308000	-0.03866600
H	-0.12633400	1.39428800	0.06492600
C	2.99762400	-1.27375700	0.16512600
C	4.36231800	-0.98847400	0.22379200
C	4.85057900	0.29913400	0.04820300
C	3.96438700	1.38631400	-0.20312300
C	2.57706900	1.08175700	-0.25961100
C	2.09950800	-0.21965000	-0.06970300
H	2.64558100	-2.29049000	0.30920500
H	5.92149300	0.48555200	0.09470800
H	1.89107200	1.89736700	-0.47197300
C	0.66220500	-0.54947200	-0.09869900
H	0.43939600	-1.61493700	-0.14674000
O	4.41416600	2.60085500	-0.37862500
O	5.21321500	-2.05065000	0.46500300
H	6.12586600	-1.72974700	0.49870900

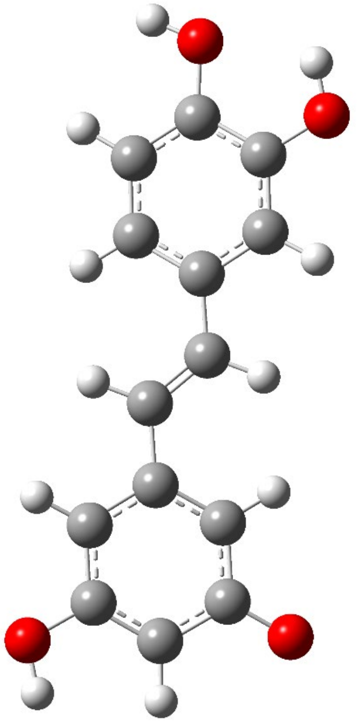

20(4) anion

Charge=-1, Multiplicity=1

C	4.54978900	-0.50110300	0.00011700
C	3.65695900	-1.56818500	0.00035800
C	2.28454900	-1.33591600	0.00033300
C	1.77981900	-0.02646700	0.00004700
C	2.69323900	1.03997500	-0.00019300
C	4.06149800	0.80972100	-0.00015700
H	4.04949400	-2.58149700	0.00057300
H	1.61201100	-2.18716700	0.00053400
H	2.33884300	2.06727900	-0.00040800
O	4.92429600	1.87699500	-0.00041100
H	5.83706300	1.54951000	-0.00023000
O	5.91534900	-0.63749000	0.00009300
H	6.16097900	-1.57448900	0.00075900
C	0.34304900	0.29080900	-0.00001400
H	0.11779000	1.35633200	-0.00011600
C	-3.00886700	-1.34219500	-0.00024800
C	-4.41957900	-1.14341400	-0.00041700
C	-4.86748500	0.20490700	-0.00008900
C	-3.95489900	1.25609800	0.00016100
C	-2.57817200	1.04895100	0.00015800
C	-2.10500600	-0.27600900	-0.00001300

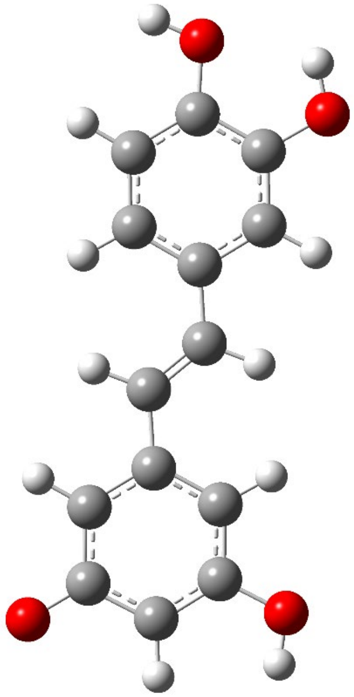

H	-2.63432100	-2.36354200	-0.00033900
H	-5.93521300	0.41303700	-0.00004900
H	-1.91175500	1.90423500	0.00036800
C	-0.66599400	-0.59696400	0.00002900
H	-0.44134900	-1.66277300	0.00007800
O	-4.38655900	2.56985000	0.00043100
H	-5.35421900	2.59258900	0.00041500
O	-5.25967600	-2.14370000	-0.00036500

1-β-TS

Charge=0, Multiplicity=2

C	2.32563200	-0.68356800	-2.23572000
C	3.15725100	-0.74616300	-1.11159600
C	2.90238800	0.05660600	0.00550400
C	1.79618200	0.91350700	0.05879400
C	0.94494900	0.94788400	-1.07353000
C	1.23730800	0.16836600	-2.20824200
H	2.54438300	-1.30047300	-3.10131300
H	3.57554200	0.00577100	0.85008100
H	0.56105500	0.22932200	-3.05658200
O	-0.18607100	1.69084900	-1.09635100
H	-1.12502900	0.96564400	-0.97050100
O	4.20645800	-1.61439500	-1.19362300
C	5.05757000	-1.74416900	-0.05764500
H	5.79731300	-2.49705100	-0.32632200
H	5.55990200	-0.79738900	0.16383400
H	4.49052000	-2.07916000	0.81670300
C	1.53659300	1.78685600	1.29153000
C	2.62896200	1.61917600	2.35491200
H	2.67900000	0.59208800	2.73174700
H	3.61677200	1.89991000	1.97446400
H	2.39751400	2.27377400	3.20087600
C	1.51309200	3.27053800	0.88095000
H	0.71439600	3.48087900	0.16679800
H	1.35467700	3.89188100	1.76934000
H	2.46854500	3.55971000	0.42968600
C	0.19603900	1.40100400	1.93953700
H	0.03604000	2.01105100	2.83584800
H	-0.64284900	1.56705100	1.26121900
H	0.20292200	0.34738400	2.24275500
N	-4.05252700	-1.51546400	1.25445800
H	-3.97158000	-0.70223800	1.85058300
H	-4.88011200	-2.09489800	1.32322900
C	-3.10472900	-1.82193000	0.36283700
O	-3.15592300	-2.81575400	-0.36974100
C	-1.93912800	-0.81828900	0.26201100
H	-1.88906300	-0.25818400	1.20171600
N	-0.68804400	-1.53041000	0.08182400
H	-0.18595000	-1.45826000	-0.79688300
C	-0.13087600	-2.22614400	1.07914200
O	-0.65737000	-2.36868200	2.18960200
H	0.84211800	-2.67063600	0.82864700
C	-2.18045900	0.11249600	-0.91112600
H	-2.04769100	-0.40309000	-1.87084900
C	-3.38190500	1.03360700	-0.87462500

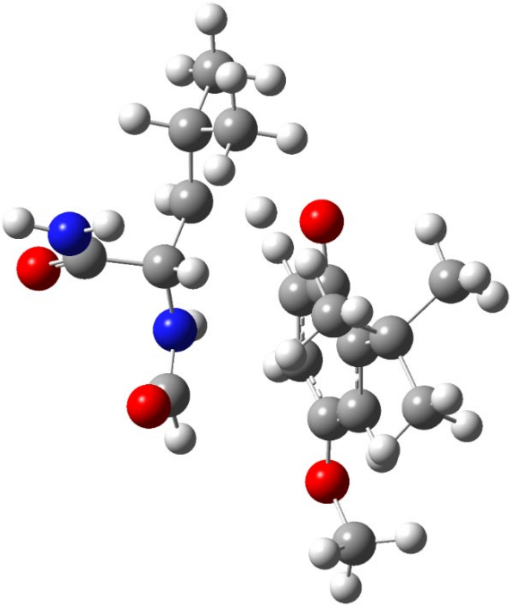

H	-4.27896100	0.39738700	-0.78873100
C	-3.48397800	1.81202000	-2.18703000
H	-2.61402900	2.46637400	-2.31286500
H	-4.38167500	2.43716800	-2.19233800
H	-3.53077300	1.13562300	-3.04605300
C	-3.35877500	1.99263300	0.31940200
H	-3.29903500	1.46951200	1.27920900
H	-4.26663700	2.60307000	0.32632400
H	-2.49677100	2.66616700	0.24569000

1-γ-TS

Charge=0, Multiplicity=2

C	3.34949900	-2.11509400	0.60354500
C	4.15874000	-1.28095200	-0.17564100
C	3.83668700	0.07073300	-0.33591200
C	2.69310700	0.63002700	0.24816900
C	1.86711400	-0.22678300	1.02328600
C	2.22208500	-1.58042500	1.19639000
H	3.62054800	-3.15795700	0.73328500
H	4.49147500	0.69782000	-0.92502400
H	1.57923400	-2.19904700	1.81577600
O	0.74582300	0.21378300	1.62761700
H	-0.24630000	-0.17700600	0.99307100
O	5.25599200	-1.86870300	-0.73706200
C	6.11728800	-1.05658000	-1.52992500
H	6.91302200	-1.71589900	-1.87419500
H	5.58174800	-0.64511100	-2.39108000
H	6.54440300	-0.24399300	-0.93390000
C	2.38627700	2.12474300	0.09548600
C	3.44212400	2.84582100	-0.75207700
H	4.43913100	2.78549000	-0.30321300
H	3.49304200	2.44535800	-1.77016500
H	3.17105700	3.90382000	-0.82288600
C	1.02669200	2.32550100	-0.59456700
H	0.21091300	1.88143300	-0.02122500
H	0.82866500	3.39803400	-0.70187100
H	1.03382900	1.87906800	-1.59494800
C	2.37489200	2.79154800	1.48352700
H	2.18699400	3.86512900	1.37060500
H	1.60077800	2.37176500	2.12872300
H	3.34484300	2.66647500	1.97730200
N	-4.20869700	-1.95348900	-1.11561900
H	-3.73602800	-1.69573200	-1.97172900
H	-4.60189200	-2.88259900	-1.03080900
C	-4.29808000	-1.10175600	-0.08920000
O	-4.87960700	-1.37625000	0.96831700
C	-3.59340400	0.24675400	-0.26934900
H	-3.31757000	0.37213500	-1.32215200
N	-4.48983000	1.32386400	0.11850900
H	-4.23630600	1.91663800	0.90066300
C	-5.68811900	1.49221800	-0.45194100
O	-6.11477300	0.77984300	-1.36864100
H	-6.26970000	2.32669200	-0.03943000
C	-2.33837300	0.33934300	0.61975400
H	-2.65278100	0.25480100	1.66700600

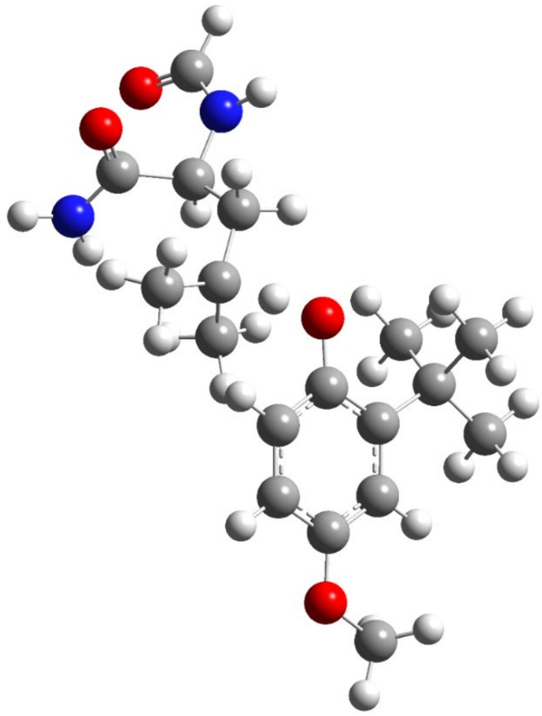

H	-1.93813200	1.34991700	0.46949800
C	-1.25057200	-0.67850900	0.32504000
C	-1.39702700	-2.03324400	0.98346900
H	-0.48699500	-2.62634200	0.84777800
H	-2.21985000	-2.60596200	0.53421200
H	-1.59853200	-1.93563800	2.05482200
C	-0.74970800	-0.72139600	-1.09964000
H	-0.75329000	0.26884400	-1.56295800
H	0.27071500	-1.12479700	-1.14223800
H	-1.37814500	-1.38702400	-1.70948100

1-δ-TS

Charge=0, Multiplicity=2

N	-2.31105700	1.43514300	0.69435400
H	-1.42746700	0.93961800	0.77455100
H	-2.31839000	2.44345100	0.78926200
C	-3.43598300	0.79679200	0.35963700
O	-4.52513200	1.36948000	0.21809900
C	-3.29738800	-0.70960800	0.10764200
H	-2.38424500	-1.07059100	0.59511900
N	-4.43821600	-1.40985900	0.67101000
H	-5.06378700	-1.91393700	0.05320900
C	-4.76082200	-1.30141300	1.96433400
O	-4.09898000	-0.64714500	2.77986100
H	-5.66724000	-1.84962200	2.25180600
C	-3.24826100	-0.98688600	-1.39944400
H	-4.16387500	-0.58357900	-1.84917700
H	-3.26433900	-2.07538600	-1.53895700
C	-2.02870100	-0.41878500	-2.13007600
H	-1.98080800	0.66839200	-1.97863300
C	-2.18452300	-0.66654800	-3.64130800
H	-2.22925800	-1.74111200	-3.84958600
H	-1.34121800	-0.24324100	-4.19366600
H	-3.10678300	-0.20460600	-4.00813700
C	-0.73494900	-1.04462200	-1.67125600
H	-0.76779900	-2.12805100	-1.51618900
H	0.15732300	-0.71771800	-2.21425700
C	3.41002200	-1.90969500	0.53022100
C	4.24278600	-0.82041700	0.27334200
C	3.70513700	0.46441100	0.10885900
C	2.33782300	0.71495800	0.18337600
C	1.49541700	-0.39898400	0.45602900
C	2.04140300	-1.67724400	0.61738300
H	3.80004100	-2.91191300	0.65974700
H	4.40872400	1.26600400	-0.08433700
H	1.35977300	-2.50008100	0.81374500
O	0.14888100	-0.27178000	0.57042400
H	-0.36101600	-0.62223700	-0.44751600
O	5.60114200	-0.90606800	0.16348000
C	6.19307400	-2.19234900	0.32247400
H	7.26495200	-2.04491700	0.19718600
H	5.98974000	-2.59307200	1.32047500
H	5.82450000	-2.88661600	-0.43915600
C	1.77921600	2.13179500	0.01303700
C	2.88277900	3.14854000	-0.30466400

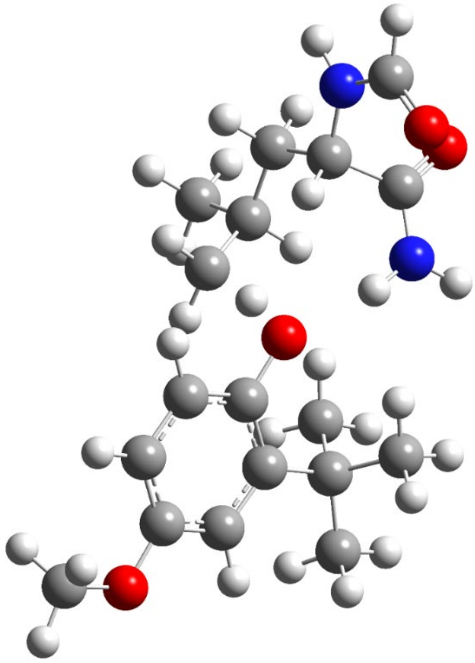

H	3.62685300	3.20932400	0.49584700
H	3.39730900	2.91095000	-1.24184400
H	2.42706600	4.13784500	-0.41257600
C	0.77208700	2.17549300	-1.15046300
H	-0.10082200	1.54768500	-0.96935600
H	0.42504800	3.20549800	-1.28949400
H	1.24599800	1.84766000	-2.08270800
C	1.10645800	2.57582000	1.32512400
H	0.62884400	3.55206800	1.18454000
H	0.35043800	1.86443600	1.66271000
H	1.85460900	2.67332000	2.11944300

2-β-TS

Charge=0, Multiplicity=2

C	-1.99069600	-1.66599200	-1.33063000
C	-2.79747900	-0.90362100	-0.47639100
C	-2.48364200	0.45327100	-0.18338400
C	-1.34548400	0.97446000	-0.78683800
C	-0.51731500	0.21316300	-1.63191800
C	-0.84961900	-1.11505300	-1.90296500
H	-2.24243400	-2.69650800	-1.54794900
H	-0.21190300	-1.70923400	-2.55004800
O	0.58879300	0.77223000	-2.17640300
H	1.55044800	0.66012400	-1.48311700
O	-3.91162900	-1.42047500	0.10574600
C	-4.25371300	-2.77695000	-0.15968500
H	-3.46121100	-3.45314100	0.17549200
H	-5.16299800	-2.96750000	0.40922200
H	-4.44818800	-2.92974700	-1.22584400
H	-1.05009900	2.00165300	-0.60820100
C	-3.33214600	1.29763900	0.77704400
C	-2.76179100	2.71385100	0.92947000
H	-1.74519000	2.70325800	1.33810700
H	-2.74798800	3.25428000	-0.02285000
H	-3.39602000	3.27389700	1.62354600
C	-4.77218500	1.43764200	0.25183000
H	-5.27739900	0.47411100	0.17041000
H	-5.34822800	2.07066900	0.93586400
H	-4.77419800	1.91560900	-0.73396600
C	-3.33116800	0.65425000	2.17636500
H	-3.89359600	1.28936600	2.86971400
H	-3.78605400	-0.33762900	2.17500100
H	-2.30560700	0.56543800	2.55325800
N	0.70233000	-0.71528200	1.09636500
H	0.75581600	0.22313100	1.47465500
H	-0.21277800	-1.14890200	1.03249800
C	1.69881700	-1.19555500	0.34020800
O	1.63762300	-2.26838000	-0.26639100
C	2.93443700	-0.28530600	0.25127200
H	2.99827600	0.27068800	1.19365000
N	4.13515100	-1.08822300	0.10664500
H	4.69500200	-1.00807200	-0.73414400
C	4.47442000	-1.99517300	1.03082400
O	3.82561300	-2.18721400	2.06620400
H	5.38787700	-2.55739000	0.79779800

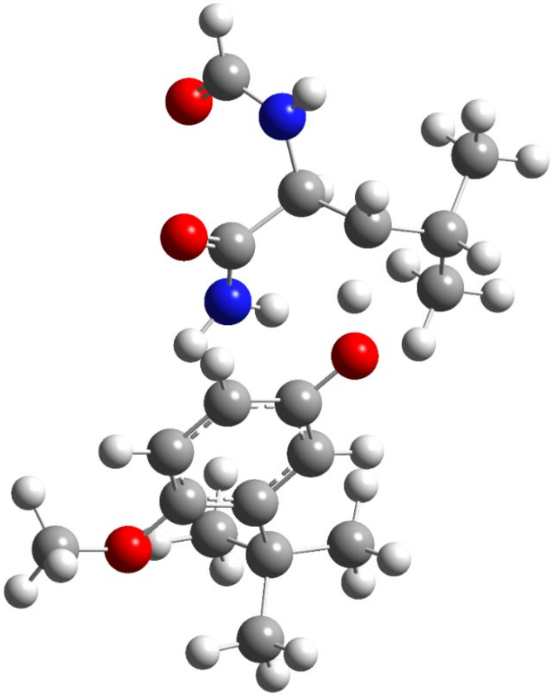

C	2.79445100	0.68067600	-0.91854900
H	3.32451900	0.32185500	-1.80726600
C	3.00575500	2.15641500	-0.63829900
H	2.84345400	2.68288400	-1.58670900
C	4.45728200	2.40406900	-0.19759900
H	4.65662500	1.92110900	0.76595800
H	4.63254800	3.47818600	-0.07787500
H	5.16975900	2.01700700	-0.93258200
C	2.02500400	2.71199100	0.39554800
H	0.98737600	2.54397600	0.09008900
H	2.17532700	3.78924900	0.51513800
H	2.17170600	2.24822800	1.37800100

2-γ-TS

Charge=0, Multiplicity=2

C	-2.13515000	-1.75152300	1.13598000
C	-3.10764000	-1.15804900	0.32147100
C	-3.13179100	0.24819500	0.10738000
C	-2.14984200	0.99402700	0.74991000
C	-1.16768600	0.41068400	1.57384300
C	-1.16270300	-0.97487500	1.75598600
H	-2.12953800	-2.82348500	1.29021000
H	-0.40491700	-1.43275900	2.38484100
O	-0.23966600	1.19025200	2.16881100
H	0.73143500	1.30121800	1.39949800
O	-4.06861300	-1.89520800	-0.29726700
C	-4.06068700	-3.30817700	-0.12458700
H	-4.19868400	-3.57530900	0.92773800
H	-4.90104200	-3.68040000	-0.70952300
H	-3.12941800	-3.74313800	-0.50076500
H	-2.10494100	2.07059800	0.62867000
C	-4.17732200	0.91657600	-0.79689100
C	-3.96899500	2.43501700	-0.86606700
H	-4.06686000	2.90689600	0.11713700
H	-2.98874500	2.69408900	-1.28052200
H	-4.73363400	2.86356300	-1.52132200
C	-4.05921000	0.37662000	-2.23427800
H	-4.25610300	-0.69505600	-2.29087100
H	-4.78154200	0.89293100	-2.87624000
H	-3.05602300	0.56753200	-2.63180300
C	-5.59584700	0.67456200	-0.24968000
H	-6.32304400	1.19126500	-0.88583600
H	-5.85460100	-0.38515600	-0.22620800
H	-5.68764800	1.07729500	0.76502100
N	4.51842900	1.22478600	-1.56460200
H	3.83864700	1.03085200	-2.28793000
H	5.20591700	1.95230600	-1.71739600
C	4.51997600	0.54743600	-0.41220700
O	5.35258000	0.73989800	0.48332000
C	3.38899000	-0.46943600	-0.22457200
H	2.87990400	-0.62698700	-1.18150700
N	3.93697100	-1.73529700	0.23449400
H	3.67316800	-2.08448100	1.14894100
C	4.86873300	-2.39901300	-0.45937300
O	5.30703500	-2.01850300	-1.55144400

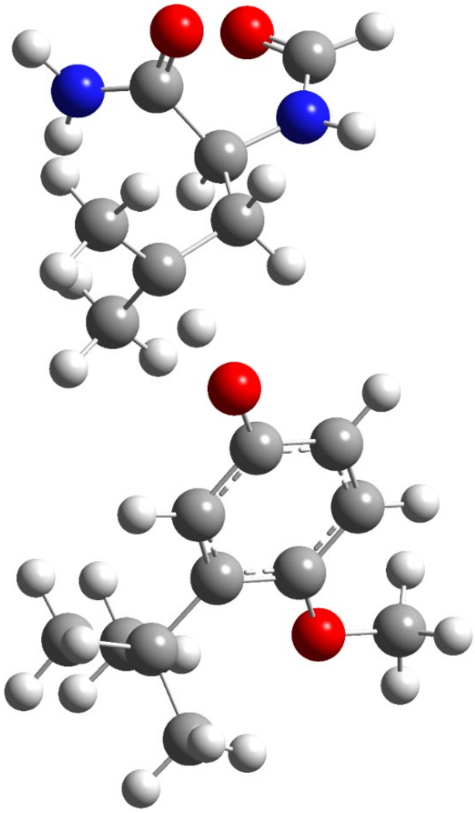

H	5.21521000	-3.32270400	0.02196100
C	2.38625900	0.02331200	0.83548200
H	2.91625800	0.11534600	1.79110000
H	1.63663500	-0.77308400	0.94057900
C	1.67615000	1.32516500	0.51432700
C	2.40105900	2.60927400	0.85519000
H	1.70540100	3.45464100	0.84474400
H	3.18697800	2.82942700	0.11968000
H	2.86848800	2.55070900	1.84264300
C	0.90994500	1.34026800	-0.78767600
H	0.33865700	0.41539600	-0.92855700
H	0.21931100	2.18965200	-0.82515800
H	1.59682100	1.44052700	-1.64117800

2-δ-TS

Charge=0, Multiplicity=2

C	-3.09995800	2.17895200	-0.48459200
C	-3.55403900	0.92503400	-0.05934400
C	-2.64167600	-0.11576600	0.26599200
C	-1.28806800	0.18651500	0.15988200
C	-0.82289500	1.44423300	-0.26103100
C	-1.73773700	2.43736400	-0.60463200
H	-3.80640100	2.96108300	-0.73318800
H	-1.38389800	3.40505800	-0.94621100
O	0.51187700	1.68639500	-0.32542600
H	0.97996500	1.03723300	-1.22693100
O	-4.88094900	0.64581700	0.05671500
C	-5.82119000	1.66165400	-0.27570300
H	-5.72247800	1.95894600	-1.32445600
H	-6.80287500	1.21865300	-0.11158700
H	-5.69934000	2.53547400	0.37203400
H	-0.53868200	-0.56256000	0.39157600
C	-3.10996500	-1.50872000	0.70996400
C	-1.91944400	-2.43434400	0.99571300
H	-1.29516400	-2.58430900	0.10796400
H	-1.28719900	-2.05083700	1.80391300
H	-2.30222300	-3.41173100	1.30518400
C	-3.93463800	-1.41137700	2.00647900
H	-4.84492900	-0.82486700	1.87298200
H	-4.21959300	-2.41837300	2.33078400
H	-3.33975200	-0.95417900	2.80478700
C	-3.94365100	-2.16869300	-0.40374300
H	-4.24230800	-3.17399100	-0.08648500
H	-4.84660500	-1.60114800	-0.63493000
H	-3.34962500	-2.26487200	-1.31929700
N	1.97754500	-0.05069900	1.58246000
H	1.43734500	0.62919000	1.05010300
H	1.56916500	-0.48724300	2.39987700
C	3.16676900	-0.47142100	1.14390900
O	3.84292100	-1.33143000	1.72590100
C	3.64135800	0.14616600	-0.17784900
H	3.10559100	1.08847600	-0.34263500
N	5.06575400	0.42215100	-0.10976400
H	5.69189000	-0.03877400	-0.75987900
C	5.58825400	1.19491500	0.84835300

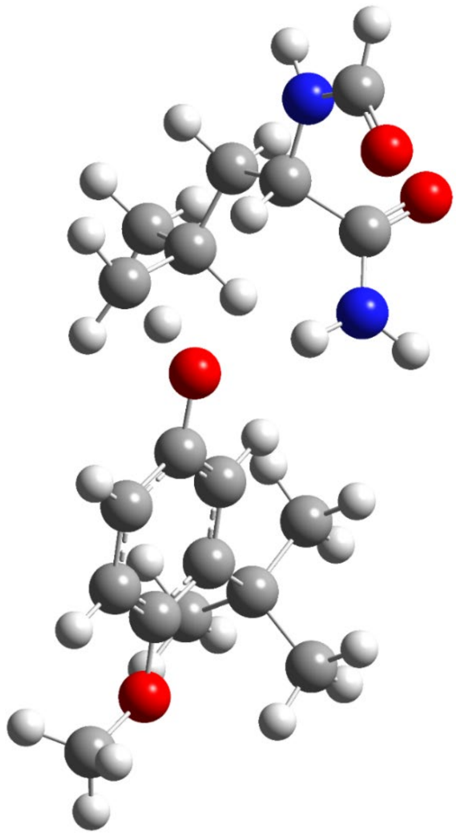

O	4.91025400	1.75580500	1.71836500
H	6.67975600	1.29928100	0.79947400
C	3.38272100	-0.82955300	-1.33261500
H	3.84336100	-1.79147800	-1.07687800
H	3.90081100	-0.44294000	-2.21970600
C	1.90870500	-1.03908000	-1.69016100
H	1.35397800	-1.37107800	-0.80049600
C	1.79374900	-2.15174800	-2.74636400
H	2.32174100	-1.86529700	-3.66252900
H	0.74664000	-2.34233400	-2.99730600
H	2.23483700	-3.08001000	-2.36929000
C	1.27162500	0.21745900	-2.23316900
H	1.90608500	0.81971300	-2.88992200
H	0.25447700	0.09050600	-2.61679800

3-β-TS

Charge=0, Multiplicity=2

C	2.87126700	0.75394100	0.16468700
C	2.53587300	-0.60252100	0.32006900
C	0.34141500	0.08654800	1.13070000
O	-0.88255100	-0.23743700	1.61597900
H	-1.60711900	-0.52989600	0.71052100
C	3.52676500	-1.68123400	-0.04260600
H	3.95937800	-2.10269100	0.87279400
H	3.00566900	-2.49921700	-0.54877100
C	4.62776200	-1.11327700	-0.93297100
H	4.23554500	-0.92144000	-1.93890200
H	5.45638300	-1.82127300	-1.02638800
C	5.16436200	0.20516800	-0.37661100
C	6.18556600	0.82038400	-1.31697400
H	6.49656400	1.80365700	-0.95158700
H	7.06748300	0.17618100	-1.37398300
H	5.76594100	0.93063800	-2.32126500
C	5.73472600	0.05958800	1.03094700
H	6.52774400	-0.69438500	1.03404600
H	6.15631100	1.01315300	1.36194900
H	4.96699800	-0.24456900	1.74844400
O	4.07639300	1.17077400	-0.32616400
C	1.95161500	1.76401600	0.47964500
H	2.24735500	2.79979300	0.34445600
C	0.69096300	1.43616400	0.95068900
H	-0.02985000	2.21027000	1.19953200
C	1.26597500	-0.91349200	0.80330100
H	0.97865800	-1.95551500	0.92473500
N	-5.47114500	-0.13610800	0.33987800
H	-5.71064600	-0.55147700	-0.55070500
H	-6.14863000	-0.15980300	1.09188000
C	-4.26973800	0.41000200	0.55189800
O	-3.94308000	0.93811000	1.62045100
C	-3.27544900	0.31142400	-0.61583100
H	-3.84340000	0.13719900	-1.54101900
N	-2.54183800	1.55628500	-0.75271400
H	-1.54733900	1.57275000	-0.55275100
C	-3.17577000	2.71902800	-0.94755600
O	-4.40045100	2.81043000	-1.09177400

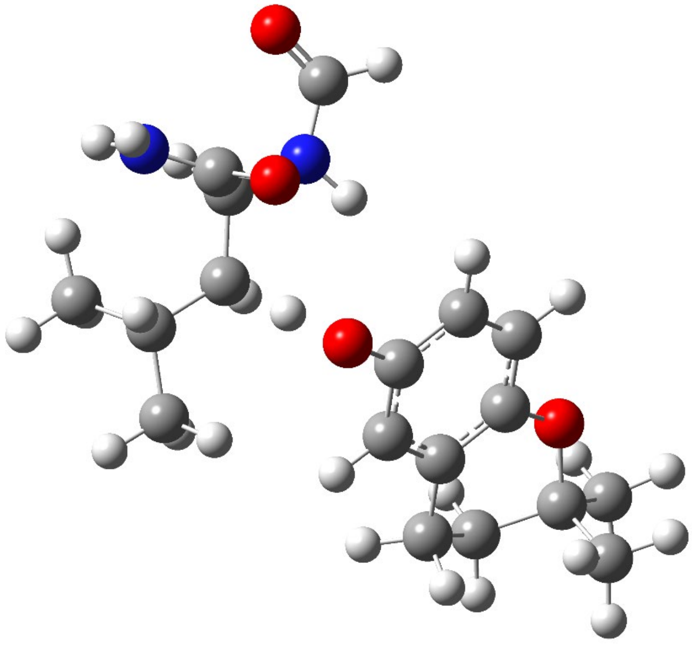

H	-2.51425100	3.59420400	-0.97722900
C	-2.30253100	-0.83256800	-0.40203900
H	-1.47421600	-0.79596400	-1.12321300
C	-2.85253600	-2.22646400	-0.19511300
H	-3.58779800	-2.20551100	0.62000600
C	-1.72895400	-3.19216000	0.18524000
H	-0.95803000	-3.20590500	-0.59454100
H	-2.12151700	-4.20767100	0.29301600
H	-1.25652500	-2.90531900	1.12881600
C	-3.55671200	-2.71794400	-1.47217500
H	-4.40559500	-2.08587700	-1.74864700
H	-3.92726500	-3.73661800	-1.32057000
H	-2.85106300	-2.73058700	-2.31049800

3-γ-TS

Charge=0, Multiplicity=2

C	-3.07930300	0.48447800	-0.52741200
C	-2.97887200	-0.79529800	0.04645600
C	-0.94233500	-1.15941700	-1.25048800
O	0.08565400	-1.96253400	-1.59580800
H	1.04790000	-1.72138000	-0.84174800
C	-4.00360800	-1.26754700	1.04858800
H	-4.67927200	-1.98687700	0.57028100
H	-3.50013800	-1.80169900	1.85984100
C	-4.79550400	-0.08410800	1.59585000
H	-4.17108000	0.50089700	2.28170400
H	-5.67309300	-0.42654100	2.15193100
C	-5.25351300	0.84199400	0.46953200
C	-5.95725700	2.06894200	1.02188400
H	-6.20176400	2.76444100	0.21345200
H	-6.88604300	1.76887400	1.51511300
H	-5.32211200	2.58039900	1.75120100
C	-6.12518100	0.12805000	-0.55929600
H	-6.99428400	-0.31654100	-0.06488800
H	-6.47624900	0.84234700	-1.30971700
H	-5.57580100	-0.66717800	-1.07170100
O	-4.07900800	1.36266000	-0.21511500
C	-2.12043300	0.94128700	-1.44337700
H	-2.23261700	1.93691900	-1.86153300
C	-1.05687900	0.13060300	-1.80237400
H	-0.30873600	0.47298700	-2.51125200
C	-1.90195400	-1.59675500	-0.32531700
H	-1.78996200	-2.58515300	0.11520800
N	4.74950400	-0.37333000	1.92884900
H	4.04880300	0.05154600	2.52138400
H	5.45886800	-0.96120000	2.34862200
C	4.75023500	-0.18156900	0.60600700
O	5.60543800	-0.66602200	-0.14641300
C	3.58946100	0.64520000	0.04295400
H	3.05144500	1.12326900	0.86861500
N	4.10394300	1.67340900	-0.84763600
H	3.83451200	1.65851800	-1.82474700
C	5.00224700	2.57921500	-0.44462700
O	5.44112600	2.64000000	0.71009800
H	5.32191500	3.27584700	-1.23020400

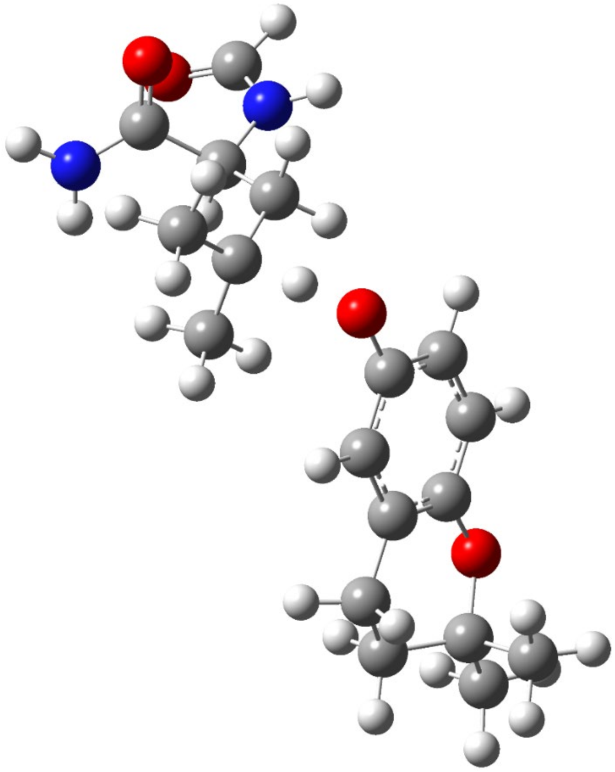

C	2.63210000	-0.24611600	-0.77014000
H	3.18843900	-0.65740300	-1.62097700
H	1.85468900	0.42114000	-1.16608900
C	1.96629500	-1.37226000	-0.00052600
C	2.75417000	-2.65359200	0.16749000
H	2.09274800	-3.47056000	0.47380100
H	3.52013600	-2.54974700	0.94823600
H	3.25494600	-2.93680200	-0.76310600
C	1.16257700	-0.94616300	1.20488000
H	0.54049800	-0.07096300	0.98445100
H	0.51589800	-1.75963600	1.55065900
H	1.82707100	-0.68054700	2.04036700

3-δ-TS water

Charge=0, Multiplicity=2

C	3.33999400	-0.59570500	-0.75762000
C	2.53471500	-0.29053600	0.35308500
C	0.56878800	-0.68608600	-1.03304600
O	-0.78115500	-0.76045100	-1.15745900
H	-1.23520600	0.34285300	-1.27306100
C	3.16172200	0.06490900	1.67896600
H	3.08290500	1.14649900	1.84257100
H	2.60272800	-0.41605800	2.48697500
C	4.62446800	-0.36746600	1.70812800
H	4.69182600	-1.45817500	1.79906500
H	5.14268400	0.07233100	2.56526900
C	5.34894600	0.04453000	0.42697000
C	6.77594700	-0.47446200	0.41465900
H	7.25580300	-0.24847600	-0.54227100
H	7.34871400	0.00615700	1.21272000
H	6.79241800	-1.55670900	0.57392000
C	5.31333300	1.55166500	0.19166600
H	5.74999900	2.07151500	1.04984000
H	5.89252400	1.80248400	-0.70175700
H	4.29102100	1.91544100	0.05218100
O	4.70627300	-0.60681700	-0.70482800
C	2.76769600	-0.93932200	-1.99001100
H	3.42374000	-1.16980800	-2.82369300
C	1.39025500	-0.97491700	-2.13416100
H	0.93428800	-1.23357500	-3.08483200
C	1.15079400	-0.33911300	0.19201000
H	0.50533400	-0.11008100	1.03784100
N	-2.33339000	-0.98137900	1.35834200
H	-1.72649800	-1.02344000	0.54087400
H	-1.96973700	-1.24687700	2.26520200
C	-3.55660200	-0.45165900	1.27928000
O	-4.31005700	-0.32918200	2.25551600
C	-3.97381000	0.05148100	-0.10822200
H	-3.36305300	-0.45143100	-0.86675900
N	-5.37046700	-0.26779000	-0.34787400
H	-6.03632700	0.48495500	-0.47843800
C	-5.82426900	-1.52410200	-0.28492300
O	-5.09851600	-2.50153300	-0.06299500
H	-6.90419900	-1.62405000	-0.45362400
C	-3.79595600	1.57248500	-0.19229100

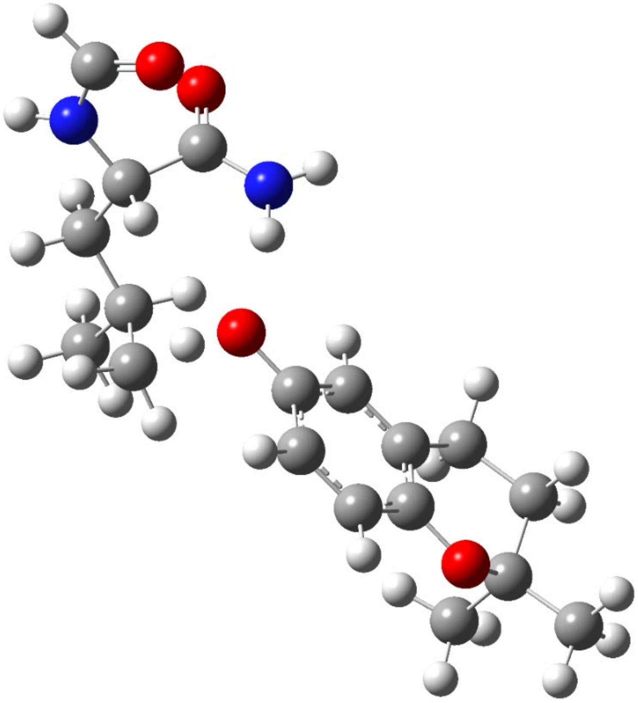

H	-4.36173200	2.02710300	0.63013700
H	-4.25315800	1.91009000	-1.13131000
C	-2.34747100	2.06805800	-0.15525300
H	-1.84987600	1.69665900	0.75134700
C	-2.34223900	3.60582400	-0.08894700
H	-2.82555600	4.02752600	-0.97703800
H	-1.32001800	3.99014700	-0.03802200
H	-2.88689100	3.94883500	0.79655200
C	-1.56051800	1.63463500	-1.36760500
H	-2.09804000	1.68752000	-2.31897700
H	-0.54381200	2.03529000	-1.42597100

4-β-TS

Charge=0, Multiplicity=2

N	3.45442500	2.33499700	1.31036800
H	3.03008600	1.80921300	2.06314700
H	4.08431700	3.09512600	1.53679200
C	3.20249300	2.04563800	0.03070700
O	3.67895900	2.68068800	-0.91740700
C	2.29948100	0.82412700	-0.21809100
H	1.80939000	0.54690300	0.72296800
N	1.26715300	1.15375800	-1.18459500
H	1.34813100	0.78996300	-2.12781500
C	0.09131100	1.67979000	-0.81217600
O	-0.16127000	2.06547800	0.33412900
H	-0.63810000	1.75809900	-1.63093000
C	3.13425300	-0.32325600	-0.74645900
H	3.40183300	-0.18577900	-1.80039700
C	4.26817900	-0.85444500	0.10413400
H	4.98739700	-0.02685000	0.23525600
C	4.98447600	-1.98628400	-0.63415600
H	4.30239300	-2.83031800	-0.78899600
H	5.83964900	-2.34353500	-0.05301700
H	5.34709400	-1.65531700	-1.61215300
C	3.81781500	-1.32051500	1.49158900
H	3.30907800	-0.53423300	2.05788800
H	4.68548500	-1.64271200	2.07493300
H	3.13423700	-2.17193600	1.40179100
C	-2.22455300	-0.70618100	0.46055300
C	-2.02359000	-0.94892200	-0.90814200
C	0.22348200	-1.71670700	-0.39065400
O	1.41714000	-2.20398400	-0.81135500
H	2.23956500	-1.34790500	-0.81587000
C	-3.11625400	-0.65354600	-1.90697400
H	-2.86669500	0.26322100	-2.45630400
H	-3.16133800	-1.45851100	-2.64647800
C	-4.45986000	-0.49790500	-1.20224500
H	-4.82640000	-1.47767900	-0.87325600
H	-5.20702000	-0.07061200	-1.87746800
C	-4.33577000	0.39630200	0.03055900
C	-5.64726300	0.46409900	0.79324500
H	-5.52159000	1.03703500	1.71688600
H	-6.40551500	0.95725700	0.17857200
H	-5.99823500	-0.54138100	1.04370400
C	-3.82727400	1.79500600	-0.30606500

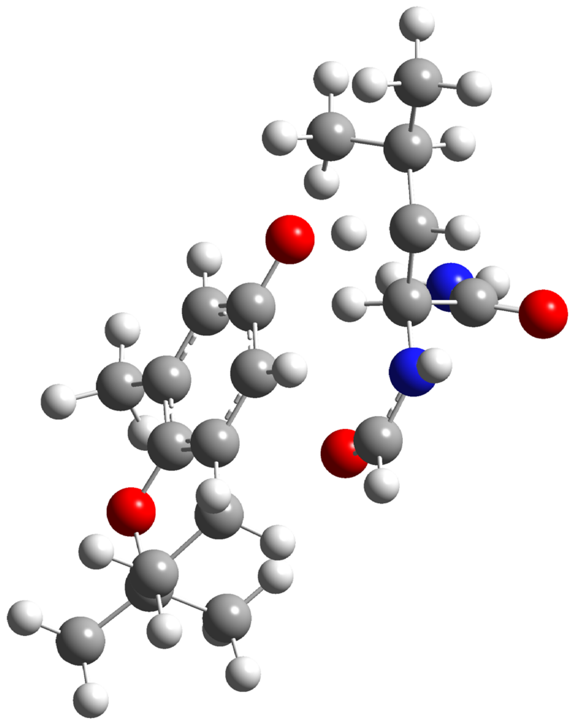

H	-4.50070700	2.27155400	-1.02506300
H	-3.79174100	2.40593600	0.60081100
H	-2.82348100	1.76683500	-0.73992200
O	-3.39715400	-0.21394400	0.95938100
C	-1.22396900	-0.97749600	1.41685200
C	-0.00848200	-1.48227200	0.97633600
H	0.78675900	-1.68671200	1.68937000
C	-0.78748700	-1.44468700	-1.31823200
H	-0.59807200	-1.62133900	-2.37463900
C	-1.47929400	-0.68345600	2.86836400
H	-1.68963400	0.38139000	3.01628800
H	-2.34656700	-1.23790000	3.24050900
H	-0.60893800	-0.95274800	3.47013300

4-γ-TS

Charge=0, Multiplicity=2

N	4.55916800	-1.52938900	1.67744800
H	4.17647600	-0.90642200	2.37615500
H	4.72204900	-2.49931600	1.91798200
C	4.83274600	-1.09999900	0.44160900
O	5.32052200	-1.82605200	-0.43424900
C	4.46357600	0.35528500	0.13623100
H	4.22404900	0.87162300	1.07229200
N	5.58555000	1.02453300	-0.50185800
H	5.47880700	1.37352300	-1.44751100
C	6.78736700	1.10733100	0.07991000
O	7.03440600	0.65618300	1.20469900
H	7.54720600	1.61665600	-0.52672900
C	3.26456800	0.42189800	-0.82882200
H	3.56177800	-0.04893900	-1.77364400
H	3.08693900	1.48669900	-1.02423700
C	1.98011100	-0.21100900	-0.32455800
C	1.83457800	-1.70778500	-0.48726300
H	0.78259800	-1.99885500	-0.38435400
H	2.39173900	-2.25035500	0.28879700
H	2.19649400	-2.04067200	-1.46465400
C	1.43838900	0.34149300	0.97175000
H	1.47450200	1.43605100	0.98931300
H	0.40197000	0.02047700	1.12872600
H	2.02201700	-0.03049700	1.82688600
C	-3.37522300	0.53966900	0.05378600
C	-2.93754000	-0.60908600	-0.62464700
C	-0.93818800	0.60713600	-1.29143300
O	0.23785200	0.64804400	-1.95341200
H	1.13274000	0.23618600	-1.19954700
C	-3.77324500	-1.86647200	-0.61962800
H	-4.26518200	-1.98281900	-1.59293700
H	-3.12080300	-2.73555200	-0.49299300
C	-4.81474900	-1.81173800	0.49312100
H	-4.33125800	-1.94939400	1.46771600
H	-5.55669600	-2.60658400	0.37256800
C	-5.53132300	-0.46238000	0.50835700
C	-6.49619800	-0.36666100	1.67698700
H	-6.93532700	0.63422500	1.72728000
H	-7.30268800	-1.09414600	1.54894900

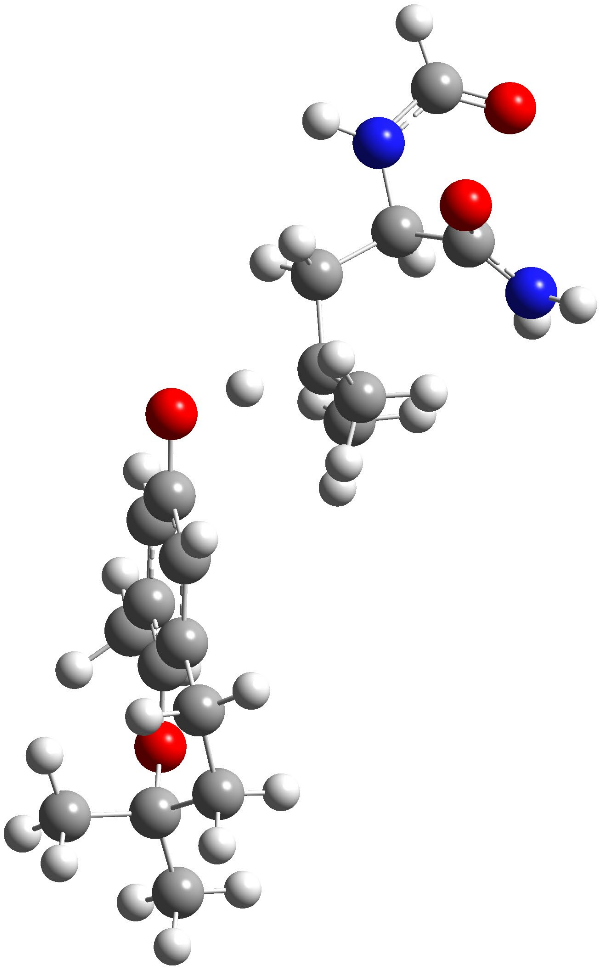

H	-5.98091000	-0.57651800	2.61899900
C	-6.23723200	-0.15815700	-0.80986200
H	-6.94704600	-0.95734900	-1.04422400
H	-6.78537800	0.78505300	-0.72895900
H	-5.52685600	-0.07385500	-1.63748700
O	-4.55109700	0.58536100	0.74836000
C	-2.60491700	1.72279300	0.08518100
C	-1.39129900	1.73807900	-0.58547700
H	-0.77339100	2.63258300	-0.57570600
C	-1.71389000	-0.55888400	-1.29108000
H	-1.34962100	-1.43668800	-1.82023700
C	-3.11031600	2.92284800	0.83699200
H	-4.07711900	3.25428200	0.44459400
H	-3.25632100	2.69145600	1.89704300
H	-2.40054000	3.74867600	0.75669200

4-δ-TS

Charge=0, Multiplicity=2

N	2.54151800	0.02941200	-1.69456500
H	1.92505100	-0.50500900	-1.08357500
H	2.19620800	0.35748600	-2.58815200
C	3.74334000	0.43408800	-1.27646600
O	4.50106900	1.13617800	-1.96108600
C	4.12861600	0.01117700	0.14675400
H	3.54118100	-0.87106800	0.42720600
N	5.53898000	-0.33277400	0.19189600
H	6.16476300	0.21132700	0.77467000
C	6.05695800	-1.27668900	-0.60120000
O	5.38275900	-1.95029700	-1.39057400
H	7.14027600	-1.41506100	-0.49158900
C	3.87168200	1.16246600	1.12663100
H	4.38949200	2.05357400	0.75128500
H	4.33530200	0.89300600	2.08434400
C	2.39822500	1.49027800	1.38350600
H	1.89742600	1.72453000	0.43330900
C	2.30752200	2.74272500	2.27296900
H	2.78722000	2.55887100	3.24057100
H	1.26473000	3.01778500	2.45243400
H	2.81188400	3.58721500	1.79240900
C	1.67046300	0.35546000	2.06196800
H	2.24453200	-0.18991900	2.81662600
H	0.64962900	0.58699800	2.38150400
C	-3.18867700	-0.71267600	0.04604000
C	-2.31733400	0.19406700	-0.57736500
C	-0.44589600	-1.11340200	0.25405900
O	0.89284500	-1.32692800	0.34557400
H	1.35641300	-0.58179900	1.15996700
C	-2.86124900	1.36515200	-1.35963900
H	-2.73617500	2.28448100	-0.77461700
H	-2.27588500	1.49451900	-2.27474700
C	-4.33386700	1.14882400	-1.69257800
H	-4.43408600	0.38896700	-2.47684900
H	-4.78984200	2.07235900	-2.06129700
C	-5.10973100	0.66667900	-0.46774500
C	-6.55237800	0.35139500	-0.82181800

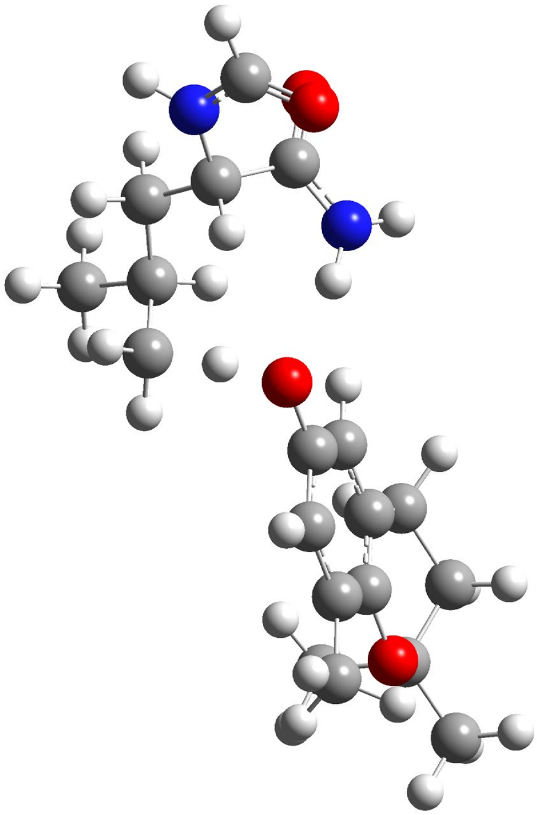

H	-7.07144900	-0.07682700	0.04094900
H	-7.06801500	1.26959100	-1.11700800
H	-6.59761700	-0.35897100	-1.65262200
C	-5.03537100	1.64858000	0.69803100
H	-5.40703200	2.62840300	0.38293600
H	-5.65271300	1.28935200	1.52660100
H	-4.00991400	1.76898400	1.05947500
O	-4.54909600	-0.60118400	-0.02803500
C	-2.70927000	-1.82342700	0.77153500
C	-1.33651500	-2.00241300	0.87655000
H	-0.93726300	-2.84319700	1.43768700
C	-0.94410600	-0.02075700	-0.46138600
H	-0.24900800	0.66769700	-0.93798300
C	-3.68306600	-2.77233300	1.41293000
H	-4.33253200	-2.24901600	2.12195300
H	-4.33294100	-3.23598000	0.66373500
H	-3.14981000	-3.56208400	1.94578800

5-β-TS

Charge=0, Multiplicity=2

N	-3.58676500	2.66189000	-0.35047000
H	-3.22667800	2.48634600	-1.27920600
H	-4.23449500	3.42777500	-0.21043100
C	-3.19914500	1.92604300	0.69495100
O	-3.57448500	2.13900700	1.85356500
C	-2.26877200	0.73883100	0.37645700
H	-1.89574700	0.85511900	-0.64778800
N	-1.13356800	0.75776500	1.28272600
H	-1.03951800	0.02522100	1.97737000
C	-0.09669900	1.58094300	1.07943000
O	-0.07507600	2.44870100	0.19728400
H	0.73570700	1.43790300	1.78214500
C	-3.03980300	-0.55872900	0.51782200
H	-3.12563000	-0.88070100	1.56197800
C	-4.31338100	-0.73374700	-0.28309600
H	-5.00314000	0.07250200	0.02189600
C	-4.96882600	-2.06990300	0.06809200
H	-4.30974800	-2.90102900	-0.20848800
H	-5.91126700	-2.18980000	-0.47434300
H	-5.17728000	-2.14020700	1.13999700
C	-4.09445900	-0.62840300	-1.79593400
H	-3.59616800	0.30061800	-2.09004800
H	-5.05655100	-0.67067800	-2.31505100
H	-3.48241700	-1.46616500	-2.14770400
C	2.31856800	-0.69187800	0.34676600
C	1.67987600	-0.30265100	-0.83950900
C	-0.16909300	-1.75481300	-0.26183600
O	-1.41547300	-2.24280400	-0.48865300
H	-2.19659800	-1.50870600	-0.00290700
C	2.31116500	0.73992800	-1.73058400
H	2.70505200	0.26671300	-2.63968400
H	1.54424400	1.44824300	-2.06112000
C	3.41528700	1.48823000	-0.99139500
H	2.97140200	2.17384300	-0.25826900
H	4.01478000	2.08353800	-1.68641000

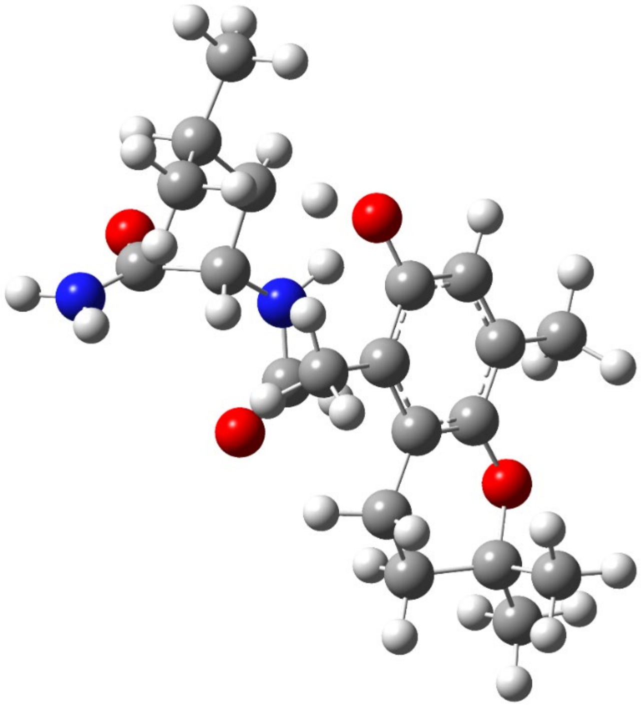

C	4.32810300	0.52504000	-0.23857100
C	5.36597600	1.27351900	0.57904400
H	5.95256800	0.57430800	1.18261100
H	6.04520400	1.80933200	-0.08997300
H	4.88339800	1.99747700	1.24238500
C	4.98985000	-0.49800000	-1.15720000
H	5.56356500	0.01685000	-1.93389800
H	5.67065200	-1.13056900	-0.58005200
H	4.25056300	-1.14098000	-1.64363800
O	3.52862500	-0.19226000	0.73997500
C	1.73632400	-1.61393800	1.24276800
C	0.49150500	-2.12557100	0.92209900
H	-0.00439500	-2.81880600	1.59712600
C	0.42668400	-0.85195900	-1.16125600
C	-0.26529000	-0.44798400	-2.43489600
H	-0.50881200	0.62146800	-2.43378500
H	0.38442500	-0.62412700	-3.29912500
H	-1.18922100	-1.00975000	-2.57493800
C	2.45716900	-1.99502200	2.50548300
H	3.43337300	-2.43848600	2.28446000
H	2.63793300	-1.11874700	3.13684000
H	1.86818500	-2.71687300	3.07507900

5-γ-TS

Charge=0, Multiplicity=2

C	-3.03682200	0.62885800	-0.23498300
C	-2.84274300	-0.74754000	-0.04430100
C	-0.82801800	-0.60015900	-1.38628600
O	0.24429000	-1.16952800	-1.97718000
H	1.17562900	-1.19281100	-1.17568500
C	-3.82056600	-1.54206300	0.78879200
H	-4.42014300	-2.18776100	0.13352200
H	-3.27222300	-2.21258700	1.45807600
C	-4.72492000	-0.62353600	1.60438000
H	-4.16688900	-0.20275600	2.44935300
H	-5.57607600	-1.17841500	2.01005700
C	-5.24081100	0.53786200	0.75986400
C	-6.05251100	1.51030000	1.59737200
H	-6.33882200	2.38124900	1.00013600
H	-6.96230800	1.01877600	1.95341200
H	-5.47444300	1.84732900	2.46293300
C	-6.03401000	0.07184200	-0.45763300
H	-6.87916100	-0.54427800	-0.13590800
H	-6.41970700	0.93763400	-1.00392300
H	-5.41654700	-0.51967700	-1.13993000
O	-4.09998600	1.30827300	0.29471200
C	-2.12823000	1.41730300	-0.97150000
C	-1.02977900	0.78555700	-1.52969900
H	-0.30627300	1.35689700	-2.10645300
C	-1.73098800	-1.37454700	-0.62997800
C	-1.50409900	-2.84845000	-0.43593100
H	-1.30976000	-3.08053700	0.61777300
H	-2.38808500	-3.42329200	-0.73235700
H	-0.65243000	-3.19281900	-1.02350600
C	-2.37089400	2.89205500	-1.13623600

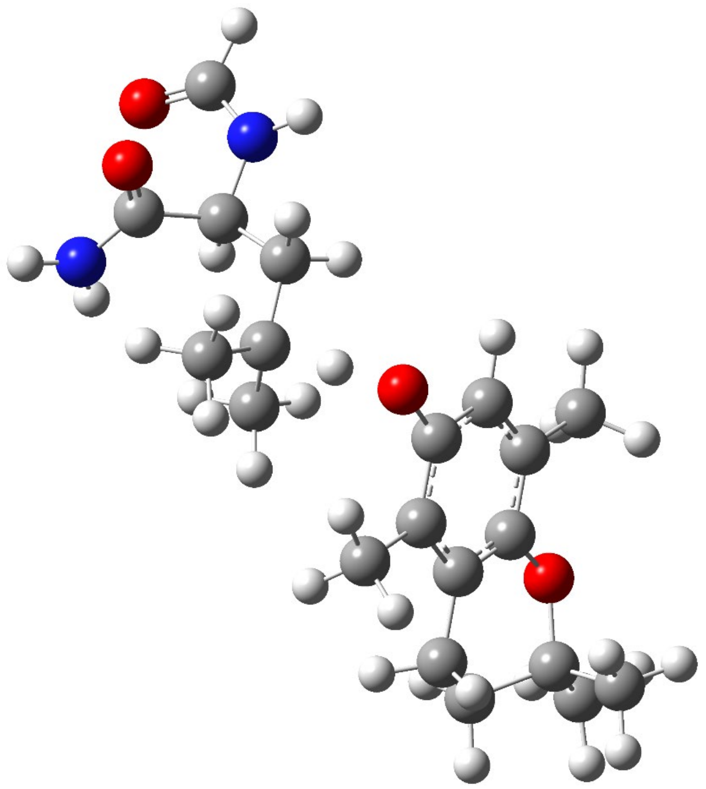

H	-3.32718100	3.08094700	-1.63479200
H	-2.40900900	3.39858700	-0.16640200
H	-1.57408700	3.34323200	-1.73128100
N	4.91048100	-0.89810700	1.84415200
H	4.24192200	-0.51186100	2.49696600
H	5.52118000	-1.64553600	2.14984400
C	4.99218700	-0.44937600	0.58762900
O	5.81269100	-0.88085800	-0.23261500
C	3.96575200	0.61469400	0.18645400
H	3.46595700	0.98873200	1.08630600
N	4.63498300	1.72067600	-0.47908400
H	4.41239600	1.92046500	-1.44781100
C	5.61593200	2.41397200	0.11028300
O	6.00478600	2.20044600	1.26475500
H	6.05184000	3.20090200	-0.51858600
C	2.92913800	0.02868100	-0.79074100
H	3.45507700	-0.27702400	-1.70343900
H	2.25362900	0.85418500	-1.05183500
C	2.10658400	-1.13091900	-0.26024300
C	2.72152900	-2.51125400	-0.33927100
H	1.95253500	-3.27876400	-0.19996700
H	3.47190600	-2.66074500	0.44932100
H	3.20887900	-2.67437800	-1.30528800
C	1.31619500	-0.84667700	0.99418100
H	0.79630800	0.11639000	0.93301300
H	0.57783400	-1.63450600	1.17810200
H	1.97800100	-0.81184300	1.87256500

5-δ-TS

Charge=0, Multiplicity=2

C	-2.51889400	0.45916600	-0.28582000
C	-1.99606500	0.22854500	0.99577500
C	-0.74085600	2.29154400	0.79845400
O	0.11477500	3.21580000	1.30899200
H	1.22193200	2.85180600	1.15114500
C	-2.43438900	-0.98341800	1.78555700
H	-1.61333200	-1.71141500	1.84039700
H	-2.64748700	-0.68890200	2.81819700
C	-3.66981600	-1.62828800	1.16582100
H	-4.55767600	-1.02281700	1.38387000
H	-3.83567400	-2.62606000	1.58267200
C	-3.53722800	-1.73336800	-0.35069100
C	-4.80675200	-2.28250800	-0.97748500
H	-4.73098900	-2.26613500	-2.06889300
H	-4.95885100	-3.31650200	-0.65524100
H	-5.67379800	-1.68907900	-0.67249800
C	-2.31972200	-2.54690400	-0.78125400
H	-2.36431800	-3.54694200	-0.33924300
H	-2.30390400	-2.64539800	-1.87071300
H	-1.38482800	-2.07234200	-0.46772200
O	-3.40447600	-0.39147300	-0.89035500
C	-2.18719400	1.60703600	-1.03346800
C	-1.29556700	2.50798100	-0.47441500
H	-1.01179700	3.40344800	-1.02142600
C	-1.07730700	1.14306400	1.53777500

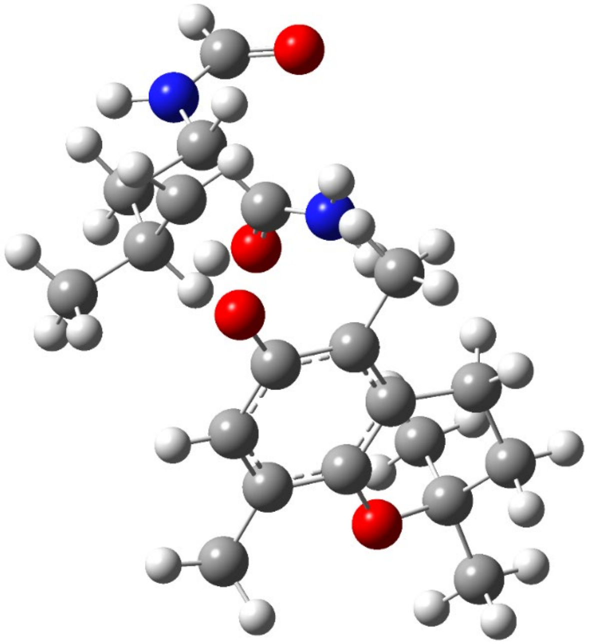

C	-0.47514500	0.91009800	2.89695300
H	-1.14686000	1.26525300	3.68834700
H	-0.29827800	-0.15360400	3.07835800
H	0.47132900	1.44400800	3.00230200
C	-2.79017200	1.82141800	-2.39403600
H	-2.54265100	0.99600000	-3.06929000
H	-3.88235300	1.87566700	-2.34005300
H	-2.41850600	2.75083500	-2.83058700
N	1.35808900	-1.17516100	0.89708500
H	1.65029800	-0.62857300	1.69685800
H	0.42236000	-1.56427800	0.89027100
C	2.12699700	-1.27907800	-0.19215400
O	1.79012800	-1.91518600	-1.19881300
C	3.44964700	-0.50709200	-0.14644600
H	3.68425700	-0.26857500	0.89649500
N	4.52056700	-1.32072600	-0.69394400
H	5.00522800	-1.00871200	-1.52751100
C	4.81513900	-2.52437800	-0.19033600
O	4.23431500	-3.01562300	0.78550600
H	5.62529400	-3.04795400	-0.71358900
C	3.33254000	0.77287700	-0.98155100
H	3.16444200	0.48203100	-2.02650400
H	4.29357400	1.29884300	-0.93121500
C	2.20613100	1.73531500	-0.55653200
H	1.25875900	1.17352900	-0.57789300
C	2.10868800	2.87915000	-1.56598200
H	3.06642200	3.40812600	-1.63288500
H	1.34423900	3.60117400	-1.26346500
H	1.85309200	2.50379600	-2.56159500
C	2.41778700	2.27323300	0.83554700
H	2.51745700	1.54942400	1.64769000
H	3.11715700	3.11005800	0.91480500

6-β-TS

N	4.06596700	-2.38730500	-0.31792600
H	3.99240200	-2.02498900	-1.25939900
H	4.75564300	-3.10189000	-0.11893500
C	3.27885500	-1.93856000	0.66390900
O	3.31848100	-2.37558900	1.81989100
C	2.32377100	-0.78817600	0.29026700
H	2.25163600	-0.73179600	-0.80221400
N	0.99987800	-1.06210000	0.82144700
H	0.65179700	-0.50830000	1.59632400
C	0.14461400	-1.86803400	0.17872700
O	0.44362700	-2.52156000	-0.82852100
H	-0.85412500	-1.91864500	0.63418400
C	2.85779500	0.51216300	0.85912600
H	2.64709100	0.61255500	1.93018300
C	4.24918600	0.96155100	0.46444400
H	4.94981300	0.16819300	0.77752900
C	4.61756200	2.23811500	1.22125300
H	3.93678800	3.05261500	0.94796000
H	5.63635700	2.55069200	0.97392000
H	4.55628900	2.08913200	2.30358400
C	4.40804600	1.17013200	-1.04524100
H	4.12030200	0.28870700	-1.62695800
H	5.45054200	1.40252800	-1.28192100
H	3.78883400	2.01080200	-1.37705900
C	-2.37826200	0.45788800	-0.50337000
C	-2.09237900	1.23810500	0.62799000
C	0.09680700	1.68651400	-0.33341000
O	1.32081000	2.25357400	-0.18454700
H	2.04432700	1.47621500	0.32364800
C	-3.11322400	1.41464700	1.72627900
H	-2.81889700	0.82189800	2.60078000
H	-3.12402000	2.46056700	2.04702500
C	-4.49595900	0.98953300	1.24295400
H	-4.90123800	1.74023600	0.55393100
H	-5.19137400	0.89567300	2.08222700
C	-4.43016400	-0.34361600	0.49965800
C	-5.78634300	-0.72376600	-0.06840800
H	-5.70757900	-1.63794300	-0.66449600
H	-6.49145500	-0.90086500	0.74851400
H	-6.17630900	0.07977500	-0.70026700
C	-3.86522100	-1.46846400	1.36251500
H	-4.48944300	-1.60299900	2.25120700
H	-3.85629200	-2.40379300	0.79481900
H	-2.84371000	-1.25351800	1.68987900
O	-3.57195500	-0.18812800	-0.66400600
C	-1.46267200	0.30944700	-1.56529100
C	-0.21207800	0.93023100	-1.48904300
C	-0.83888500	1.83475600	0.69546000
H	-0.56659200	2.42873000	1.56504100
C	-1.86573200	-0.55868000	-2.72769300
H	-2.10297700	-1.57144300	-2.38349100
H	-2.76610700	-0.16911700	-3.21432300
H	-1.07660900	-0.62845100	-3.47569600
C	0.80442600	0.77546600	-2.59067800

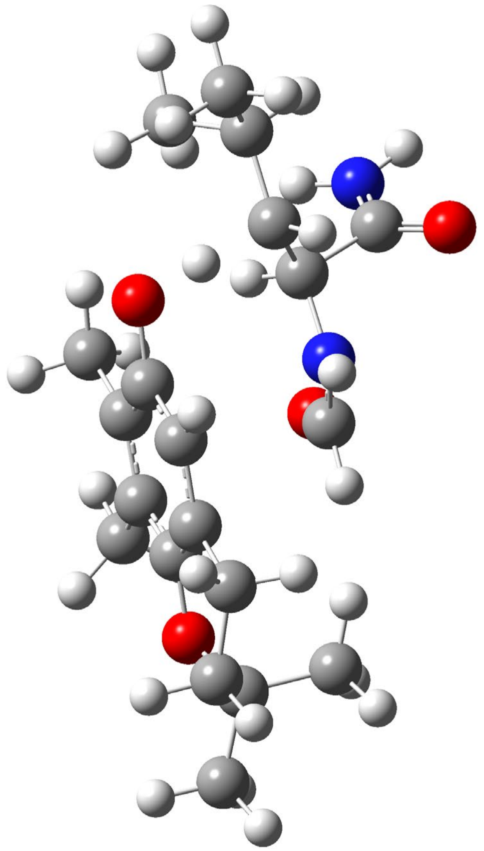

H	1.72642900	1.30289000	-2.34372100
H	1.04658700	-0.27958100	-2.76055800
H	0.42241700	1.17607000	-3.53557500

6-γ-TS

Charge=0, Multiplicity=2

N	-5.04565700	0.74389100	1.04335700
H	-5.52932300	0.19137000	0.34798600
H	-5.52824400	0.99204900	1.89811400
C	-3.79061700	1.15977500	0.84743000
O	-3.17752300	1.86687700	1.65746900
C	-3.11404400	0.66856700	-0.43611400
H	-3.86841000	0.21964600	-1.09138000
N	-2.49035200	1.78734000	-1.12392400
H	-1.48611200	1.78241500	-1.26348400
C	-3.18699100	2.86315100	-1.50717900
O	-4.40589900	2.98442600	-1.33540700
H	-2.58400200	3.63970700	-1.99511600
C	-2.01270700	-0.36036600	-0.11798700
H	-1.24204500	0.13857300	0.48419800
H	-1.56162400	-0.62617600	-1.08357200
C	-2.47572200	-1.62472000	0.57894500
C	-2.61345600	-1.55988400	2.08399100
H	-2.68975800	-2.56804100	2.50417900
H	-3.52092000	-1.01646900	2.38169600
H	-1.75372300	-1.05514800	2.53707900
C	-3.54363000	-2.41605800	-0.14057500
H	-3.33483500	-2.48334900	-1.21334400
H	-3.61780300	-3.42886600	0.26783000
H	-4.52925700	-1.94327700	-0.01770700
C	-1.49309879	-6.73130997	2.14219396
C	-1.79269032	-6.54190103	0.78407923
C	-0.80500185	-4.32200846	0.95351744
O	-0.47594668	-3.16390803	0.34121155
H	-1.41583300	-2.37547000	0.44877700
C	-2.46326491	-7.63015067	-0.01924113
H	-3.51614414	-7.36995353	-0.18274452
H	-1.99912244	-7.69129182	-1.00809114
C	-2.35930503	-8.96824325	0.70486324
H	-1.33368689	-9.35133281	0.64188676
H	-3.02241224	-9.71076649	0.25120848
C	-2.71644875	-8.82022351	2.18302343
C	-2.51820652	-10.12856237	2.92833861
H	-2.68963992	-9.98655720	3.99963246
H	-3.22783801	-10.87417234	2.55877577
H	-1.50249725	-10.50638080	2.77801708
C	-4.12810846	-8.28029415	2.39533037
H	-4.85379629	-8.93027117	1.89698029
H	-4.35847416	-8.25322516	3.46443979
H	-4.23849508	-7.26863392	1.99366032
O	-1.77979851	-7.89552300	2.80080538
C	-0.84781728	-5.74337205	2.91461586
C	-0.51186887	-4.52063644	2.32652466
C	-1.44344686	-5.32417413	0.21173979
H	-1.65572819	-5.13713953	-0.83878014

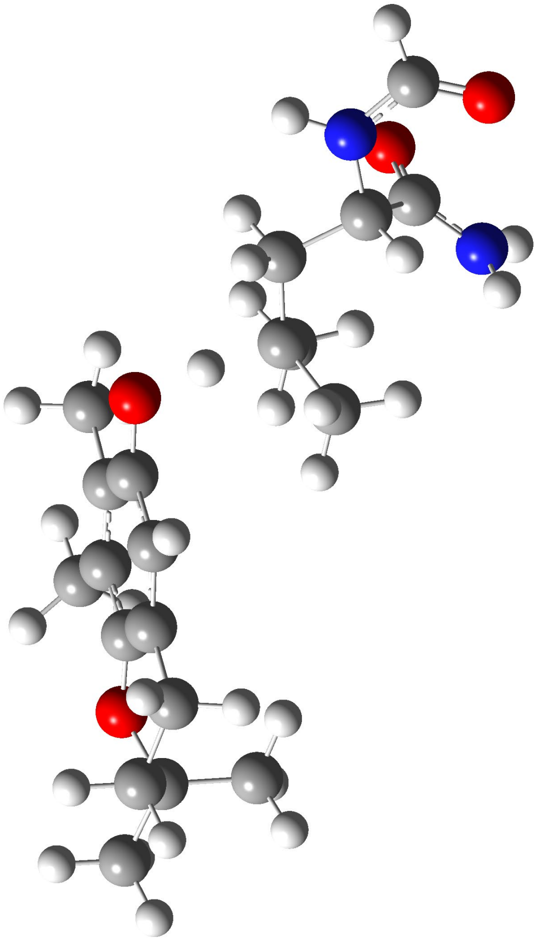

C	-0.55708747	-6.05291319	4.35999764
H	-1.48599724	-6.20242062	4.92122586
H	0.01996029	-6.97869793	4.44880911
H	0.00720884	-5.25374556	4.83965033
C	0.13196277	-3.41576857	3.12353407
H	1.14006126	-3.69531768	3.44859156
H	0.20765394	-2.50475660	2.52846080
H	-0.44884515	-3.19014913	4.02338313

6-δ-TS

Charge=0, Multiplicity=2

N	2.54151800	0.02941200	-1.69456500
H	1.92505100	-0.50500900	-1.08357500
H	2.19620800	0.35748600	-2.58815200
C	3.74334000	0.43408800	-1.27646600
O	4.50106900	1.13617800	-1.96108600
C	4.12861600	0.01117700	0.14675400
H	3.54118100	-0.87106800	0.42720600
N	5.53898000	-0.33277400	0.19189600
H	6.16476300	0.21132700	0.77467000
C	6.05695800	-1.27668900	-0.60120000
O	5.38275900	-1.95029700	-1.39057400
H	7.14027600	-1.41506100	-0.49158900
C	3.87168200	1.16246600	1.12663100
H	4.38949200	2.05357400	0.75128500
H	4.33530200	0.89300600	2.08434400
C	2.39822500	1.49027800	1.38350600
H	1.89742600	1.72453000	0.43330900
C	2.30752200	2.74272500	2.27296900
H	2.78722000	2.55887100	3.24057100
H	1.26473000	3.01778500	2.45243400
H	2.81188400	3.58721500	1.79240900
C	1.67046300	0.35546000	2.06196800
H	2.24453200	-0.18991900	2.81662600
H	0.64962900	0.58699800	2.38150400
C	-3.18867700	-0.71267600	0.04604000
C	-2.31733400	0.19406700	-0.57736500
C	-0.44589600	-1.11340200	0.25405900
O	0.89284500	-1.32692800	0.34557400
H	1.35641300	-0.58179900	1.15996700
C	-2.86124900	1.36515200	-1.35963900
H	-2.73617500	2.28448100	-0.77461700
H	-2.27588500	1.49451900	-2.27474700
C	-4.33386700	1.14882400	-1.69257800
H	-4.43408600	0.38896700	-2.47684900
H	-4.78984200	2.07235900	-2.06129700
C	-5.10973100	0.66667900	-0.46774500
C	-6.55237800	0.35139500	-0.82181800
H	-7.07144900	-0.07682700	0.04094900
H	-7.06801500	1.26959100	-1.11700800
H	-6.59761700	-0.35897100	-1.65262200
C	-5.03537100	1.64858000	0.69803100
H	-5.40703200	2.62840300	0.38293600
H	-5.65271300	1.28935200	1.52660100
H	-4.00991400	1.76898400	1.05947500

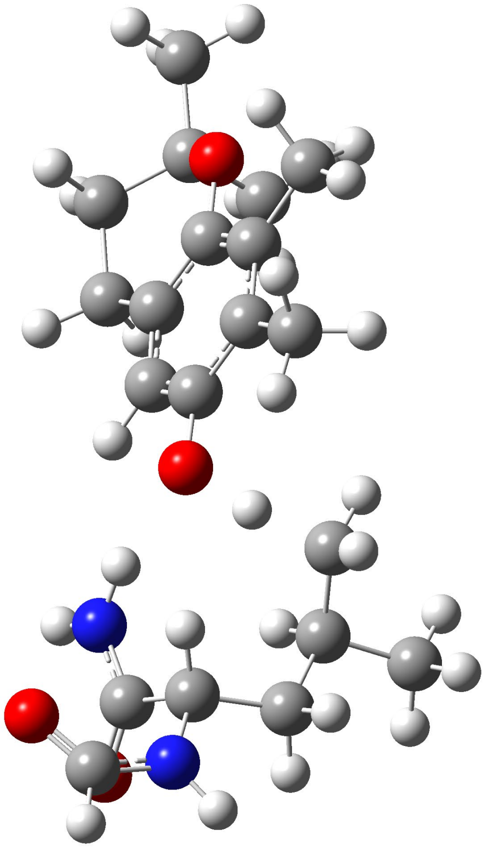

O	-4.54909600	-0.60118400	-0.02803500
C	-2.70927000	-1.82342700	0.77153500
C	-1.33651500	-2.00241300	0.87655000
H	-0.93726300	-2.84319700	1.43768700
C	-0.94410600	-0.02075700	-0.46138600
H	-0.24900800	0.66769700	-0.93798300
C	-3.68306600	-2.77233300	1.41293000
H	-4.33253200	-2.24901600	2.12195300
H	-4.33294100	-3.23598000	0.66373500
H	-3.14981000	-3.56208400	1.94578800

7-β-TS

Charge=0, Multiplicity=2

C	-2.31969200	-0.63104100	-0.12802700
C	-1.71236100	0.02772300	0.94980400
C	0.16695500	-1.48641300	0.72241700
O	1.41137300	-1.86908200	1.10249900
H	2.19145400	-1.30666500	0.44084400
C	-2.36972000	1.23359600	1.57681900
H	-2.76798300	0.97352400	2.56649000
H	-1.61727500	2.01184100	1.74332300
C	-3.47576000	1.77479600	0.67763000
H	-3.03336800	2.28670900	-0.18653300
H	-4.09632300	2.49842700	1.21448900
C	-4.35835500	0.64738500	0.15125000
C	-5.40037600	1.17120700	-0.82144600
H	-5.96475300	0.34218600	-1.25903400
H	-6.09902900	1.82759100	-0.29497300
H	-4.92331100	1.73964000	-1.62540700
C	-5.01210300	-0.15874700	1.27008900
H	-5.60702200	0.50370600	1.90629700
H	-5.67205700	-0.91996300	0.84366000
H	-4.26683400	-0.65910700	1.89519500
O	-3.53187100	-0.25012100	-0.63673400
C	-1.70588200	-1.70877200	-0.79807600
C	-0.44380600	-2.12949700	-0.38122800
C	-0.45925800	-0.41709700	1.39125300
C	0.21503200	0.27604700	2.54522200
H	0.45873200	1.31537700	2.29211900
H	-0.44675800	0.30914500	3.41742400
H	1.13709100	-0.23239000	2.82723300
C	-2.42305800	-2.32935200	-1.96812200
H	-3.45447800	-2.57959100	-1.70317900
H	-2.47142900	-1.63043600	-2.81158000
H	-1.92718100	-3.23750900	-2.31013700
C	0.29950000	-3.20920200	-1.12577200
H	-0.25351400	-4.15414300	-1.11557100
H	0.43999000	-2.92871000	-2.17568200
H	1.28053800	-3.38490300	-0.68491600
N	3.55567400	2.81860000	-0.20421800
H	3.18607000	2.86088600	0.73616900
H	4.20023400	3.53689300	-0.51131700
C	3.17543600	1.86630400	-1.06085800
O	3.56121700	1.81493700	-2.23452400
C	2.24862100	0.77517400	-0.49096000

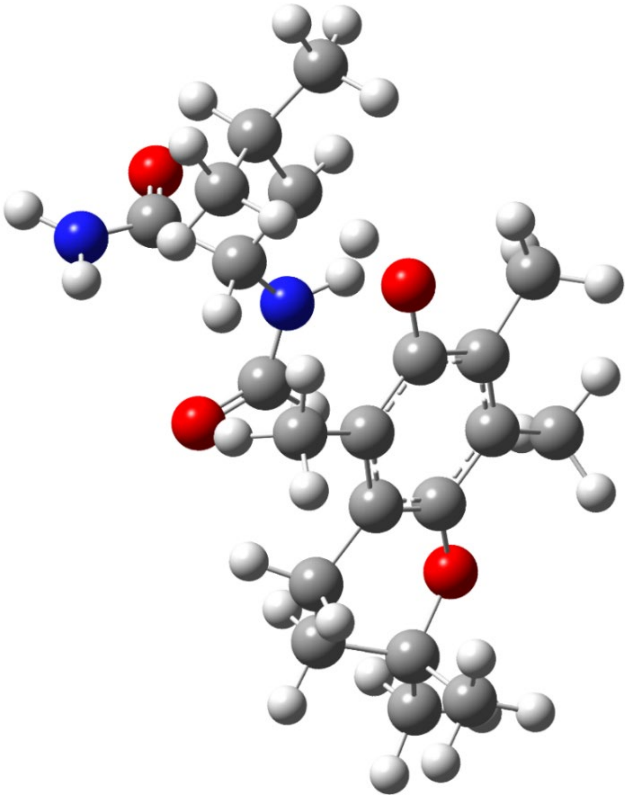

H	1.85122500	1.12133600	0.47119500
N	1.13125100	0.56416000	-1.39545800
H	1.06266200	-0.31169600	-1.90267000
C	0.07522000	1.38815300	-1.40014500
O	0.02328800	2.43828200	-0.74734500
H	-0.74470400	1.06444600	-2.05612500
C	3.03391500	-0.51002200	-0.31215000
H	3.11879600	-1.08054300	-1.24454600
C	4.30864300	-0.47892500	0.50474500
H	4.99638900	0.23090300	0.01293200
C	4.96619100	-1.85960100	0.48683300
H	4.30507000	-2.60095300	0.95083700
H	5.90608500	-1.84597400	1.04650200
H	5.17926200	-2.18405400	-0.53628600
C	4.08946100	-0.01086700	1.94728100
H	3.58716700	0.95948200	2.00783400
H	5.05175700	0.07890200	2.45998300
H	3.48133600	-0.74089900	2.49264300

7-γ-TS

Charge=0, Multiplicity=2

C	-3.37646600	0.41248400	0.14205000
C	-2.79433000	-0.70903700	-0.46444700
C	-0.89023000	0.68365300	-1.04951200
O	0.30287300	0.82108400	-1.66625800
H	1.19766700	0.53684900	-0.88821700
C	-3.50856000	-2.03966100	-0.45812900
H	-3.87872600	-2.26303100	-1.46764000
H	-2.80054700	-2.83692200	-0.21041100
C	-4.65939400	-2.04141200	0.54261000
H	-4.26776600	-2.11679300	1.56414100
H	-5.31891300	-2.89803700	0.37432700
C	-5.47464000	-0.75499300	0.44852400
C	-6.55460300	-0.70857600	1.51526400
H	-7.07497000	0.25365600	1.49008000
H	-7.28447400	-1.50300000	1.33560900
H	-6.11915400	-0.85105200	2.50881100
C	-6.06939900	-0.53733800	-0.93999600
H	-6.70336500	-1.38748300	-1.20983900
H	-6.68007200	0.37039800	-0.94507000
H	-5.29028800	-0.43323800	-1.70085900
O	-4.60142900	0.36509500	0.75316700
C	-2.73432900	1.66569900	0.18664700
C	-1.47762800	1.80360800	-0.40409400
C	-1.54189200	-0.56922300	-1.07461100
C	-0.90655400	-1.75340200	-1.75169400
H	-0.69110200	-2.54973500	-1.03027600
H	-1.58141400	-2.17739300	-2.50346300
H	0.02721700	-1.47476400	-2.24025800
C	-3.44489800	2.80658500	0.86750000
H	-4.42118500	2.98622400	0.40534900
H	-3.63107300	2.57777000	1.92228900
H	-2.86887500	3.72995200	0.81737800
C	-0.75765200	3.12864600	-0.39114100
H	-1.34227500	3.89618700	-0.90949800

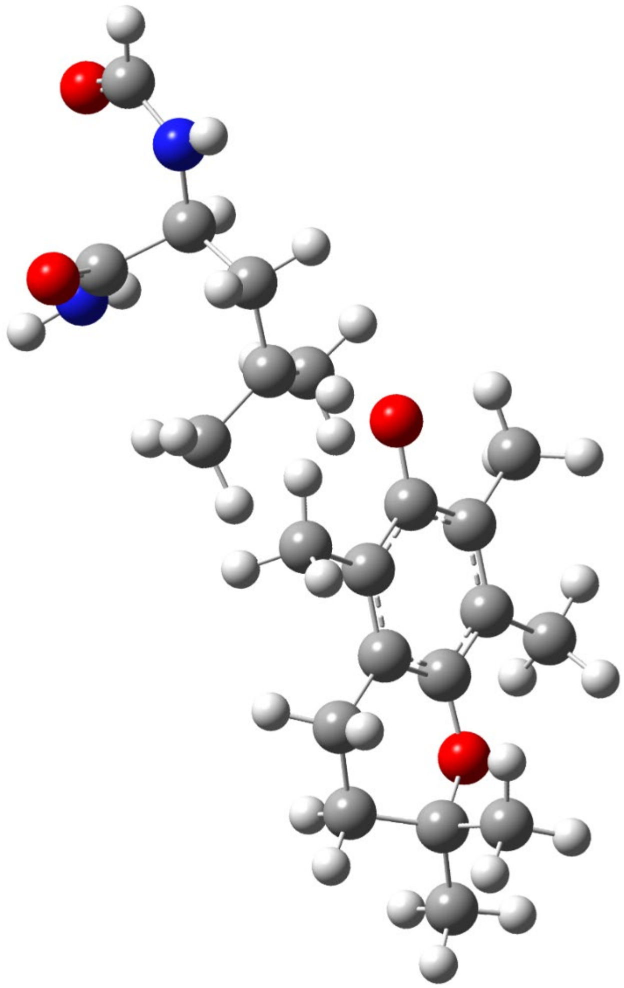

H	-0.59523400	3.48082400	0.63262000
H	0.21190300	3.05027500	-0.88193800
N	4.66752600	-0.82945100	2.17539400
H	4.58625700	0.10292800	2.55835500
H	4.65551500	-1.62248800	2.80476700
C	4.76566500	-1.03346400	0.85814200
O	4.88348000	-2.15843700	0.35528200
C	4.67849200	0.21490200	-0.02585700
H	4.75325100	1.10881300	0.60262200
N	5.76970400	0.21614200	-0.98680300
H	5.55401600	0.17052700	-1.97623700
C	7.05220300	0.17247100	-0.60820900
O	7.41644900	0.16094100	0.57372800
H	7.76657100	0.15085200	-1.44138700
C	3.35482500	0.23170700	-0.81355400
H	3.34350300	-0.64008600	-1.47911600
H	3.38064900	1.13285700	-1.43909400
C	2.09465400	0.24573900	0.03040200
C	1.59729800	-1.08232300	0.55347600
H	0.53334800	-1.01523200	0.81499500
H	2.12825900	-1.37165400	1.47130200
H	1.72686000	-1.87939400	-0.18437800
C	1.95650300	1.39609400	0.99940900
H	2.25849800	2.34508100	0.54484300
H	0.91981700	1.48514100	1.34521300
H	2.58012800	1.23271700	1.89084800

7-δ-TS

Charge=0, Multiplicity=2

C	3.25116100	0.59594900	-0.09267800
C	2.56985000	-0.53601500	-0.56625700
C	0.49429200	0.63491600	-0.12354000
O	-0.86130200	0.66396900	-0.20313300
H	-1.33071100	0.19652800	0.76109100
C	3.34165800	-1.72535900	-1.09273700
H	3.27911200	-2.55655700	-0.37853300
H	2.88160400	-2.08035400	-2.01995100
C	4.79959500	-1.36020700	-1.35101200
H	4.87772100	-0.74776000	-2.25737000
H	5.40289400	-2.26013300	-1.50322600
C	5.38019000	-0.55600600	-0.19211800
C	6.79953800	-0.10552900	-0.49078500
H	7.16968000	0.54374000	0.30844800
H	7.45595900	-0.97743300	-0.56184500
H	6.83793200	0.44048600	-1.43810400
C	5.31469800	-1.30661900	1.13512700
H	5.84789100	-2.25846400	1.05021500
H	5.78516900	-0.71085600	1.92288400
H	4.28230300	-1.51528100	1.43052500
O	4.61917300	0.67324300	-0.06191100
C	2.58308100	1.74598900	0.36900400
C	1.18640900	1.76396500	0.36738300
C	1.16986200	-0.52269100	-0.55105300
C	0.36545100	-1.71815800	-0.98837100
H	0.00673000	-1.60197700	-2.01889000
H	0.95278300	-2.63618200	-0.93983500
H	-0.51427800	-1.84299300	-0.34951400
C	3.41030100	2.91050000	0.84769100
H	4.11136100	3.23398400	0.07168800
H	2.78813000	3.76319300	1.11833000
H	4.00986700	2.63241800	1.72132900
C	0.41618700	2.95338400	0.88064100
H	-0.65216900	2.73919400	0.91183700
H	0.74148700	3.22342100	1.88966800
H	0.56896700	3.83144700	0.24370500
N	-2.91220400	-0.88853600	-1.64909300
H	-2.11643500	-0.36225900	-1.29235700
H	-2.76372300	-1.57337800	-2.38017400
C	-4.11754900	-0.79912000	-1.08092400
O	-5.09480500	-1.46361500	-1.45471500
C	-4.22767500	0.15359200	0.11654600
H	-3.40598300	0.87797100	0.07594300
N	-5.48833900	0.87326200	0.05734600
H	-6.17091000	0.73995100	0.79465200
C	-5.82557000	1.61549900	-1.00251900
O	-5.08717700	1.78358300	-1.98117200
H	-6.81981900	2.07533900	-0.93417500
C	-4.18011300	-0.64203300	1.42701600
H	-4.99574000	-1.37537100	1.40786100
H	-4.38486400	0.05642600	2.24861300
C	-2.85683500	-1.35686900	1.71389300
H	-2.61036900	-2.02617300	0.87830500

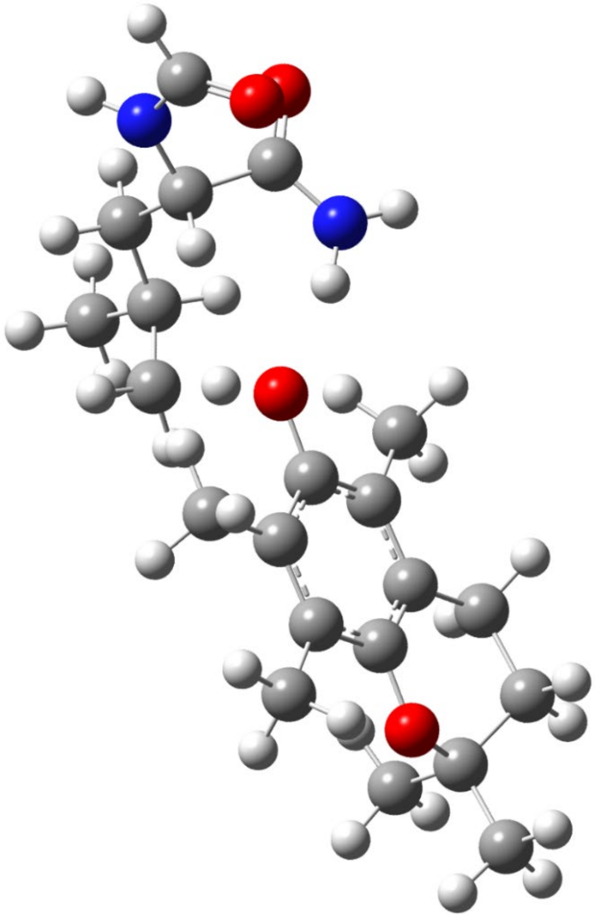

C	-3.01784500	-2.22777000	2.97295200
H	-3.26724200	-1.60431300	3.83856700
H	-2.09357300	-2.76864900	3.19337700
H	-3.82128200	-2.95768400	2.83003600
C	-1.71590500	-0.39470900	1.93374000
H	-1.94608300	0.47317700	2.55946800
H	-0.75439400	-0.85823800	2.17511900

8-β-TS

Charge=0, Multiplicity=2

N	3.03832000	0.03604100	2.00968000
H	2.17555200	0.21078400	2.50690100
H	3.89009900	-0.10324000	2.53909900
C	3.09535500	0.08045100	0.67515600
O	4.14689200	-0.03968300	0.03711300
C	1.74352700	0.22538100	-0.05283800
H	0.99822800	0.60649500	0.65746400
N	1.88013300	1.16920700	-1.14670500
H	1.66478000	0.87231000	-2.09182000
C	2.16766000	2.45472400	-0.91695600
O	2.39509500	2.90850500	0.21143100
H	2.19226500	3.08208700	-1.81709900
C	1.32919200	-1.12585600	-0.59542700
H	1.97653300	-1.45266600	-1.41798200
C	0.97725700	-2.23677200	0.36970200
H	1.91417800	-2.52559600	0.87741500
C	0.47535700	-3.45409000	-0.41021700
H	-0.47590500	-3.22208600	-0.90398900
H	0.31112600	-4.30057700	0.26286300
H	1.19362800	-3.75825700	-1.17789500
C	-0.04029000	-1.83659300	1.44317200
H	0.30442800	-1.01154200	2.07274300
H	-0.23506600	-2.69104100	2.09849100
H	-0.99074400	-1.53942900	0.98678900
C	-1.71807600	-0.05726500	-1.14475800
C	-2.82619100	-0.89359100	-0.93692100
C	-3.86164700	-0.49580600	-0.10568000
C	-3.80829300	0.74377500	0.56108700
C	-2.69078500	1.57562500	0.35759700
C	-1.66325100	1.18228100	-0.48675200
H	-2.85984400	-1.85623000	-1.43943900
H	-4.72277000	-1.14147700	0.04324800
H	-2.64396100	2.53456700	0.86628000
H	-0.80270300	1.82841700	-0.64498900
O	-0.70995700	-0.45318300	-1.96237300
H	0.20060800	-0.82085900	-1.32640000
N	-4.80896200	1.11203500	1.44400500
H	-5.70534200	0.66364200	1.30075000
H	-4.88665600	2.10501900	1.62634300

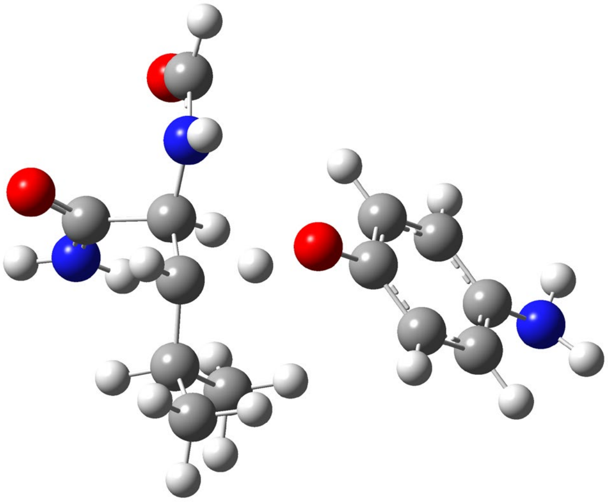

8-γ-TS

Charge=0, Multiplicity=2

N	3.76032600	0.71992100	0.98826400
H	4.11192500	1.18323300	0.16102700
H	4.18534500	0.92430900	1.88417900
C	2.74536300	-0.14715300	0.92211900
O	2.30476900	-0.74892900	1.90994600
C	2.10572900	-0.33037400	-0.45758400
H	2.74678300	0.13142600	-1.21639300
N	1.96246200	-1.74681300	-0.75154700
H	1.03321500	-2.13116900	-0.87929500
C	3.00729300	-2.58237800	-0.75554800
O	4.17194400	-2.22205800	-0.54681900
H	2.74645700	-3.62790600	-0.96436400
C	0.70169300	0.30203800	-0.49503900
H	0.07218100	-0.22341700	0.23532300
H	0.29866600	0.09197100	-1.49497600
C	0.64774600	1.79577400	-0.24222300
C	0.63014100	2.24223700	1.20258300
H	0.29732800	3.28278800	1.27800600
H	1.63302100	2.19140400	1.64930200
H	-0.03726100	1.61543100	1.80320000
C	1.46669200	2.65169300	-1.17994400
H	1.35313400	2.32524100	-2.21868600
H	1.16962300	3.70259400	-1.10628200
H	2.53575200	2.59744200	-0.92605000
C	-2.44367300	1.08548100	-0.51007500
C	-2.69037500	0.03201500	-1.40796100
C	-3.39248100	-1.09206500	-1.00137100
C	-3.85515100	-1.20655900	0.32326600
C	-3.59766600	-0.15640900	1.22486500
C	-2.90085800	0.96995500	0.81446600
H	-2.32279400	0.11454900	-2.42709600
H	-3.58778100	-1.89906400	-1.70235700
H	-3.95127300	-0.23851800	2.24908300
H	-2.69906000	1.77867300	1.51164500
O	-1.76497300	2.18411900	-0.91115900
H	-0.58820100	2.05056000	-0.61284500
N	-4.60261200	-2.30545500	0.71554300
H	-4.46937500	-3.14089600	0.15895700
H	-4.60953800	-2.49279400	1.71073400

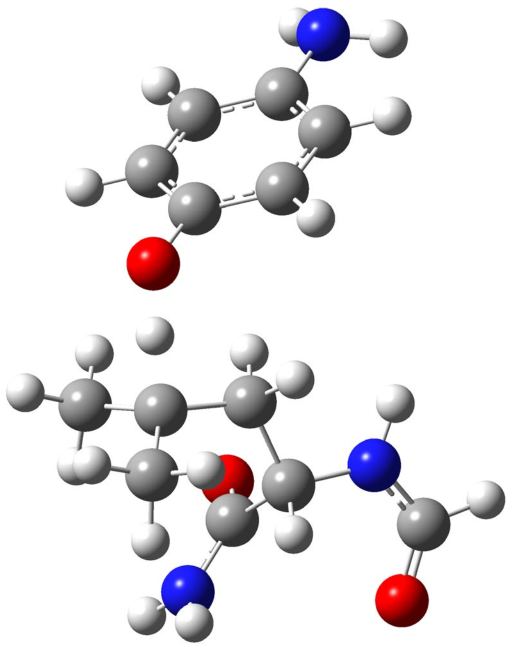

8-δ-TS

Charge=0, Multiplicity=2

N	-1.18643500	-1.05787000	1.37518600
H	-0.45519700	-1.03319200	0.66590100
H	-0.96537000	-1.37793500	2.31000800
C	-2.39924700	-0.55405300	1.13428200
O	-3.30065300	-0.51704100	1.98412600
C	-2.61037300	0.03213200	-0.26730400
H	-1.87709900	-0.40928100	-0.95271200
N	-3.94580600	-0.29067900	-0.73905400
H	-4.59984500	0.46042100	-0.92643200
C	-4.37727000	-1.55379600	-0.81951700
O	-3.67300700	-2.53457000	-0.54831900
H	-5.41730500	-1.65569300	-1.15498500
C	-2.45863200	1.55778100	-0.22682600
H	-3.14304800	1.94914100	0.53579700
H	-2.78875200	1.95084900	-1.19696800
C	-1.03989900	2.06783800	0.04156300
H	-0.66539400	1.64665300	0.98519900
C	-1.07543300	3.59823600	0.20193200
H	-1.44047500	4.06962100	-0.71713700
H	-0.07764200	3.99012800	0.41674500
H	-1.74296500	3.87901900	1.02301800
C	-0.08595100	1.71570300	-1.07227500
H	-0.49067600	1.79318800	-2.08546600
H	0.91956100	2.13618700	-0.97394200
C	2.05876300	-0.54202600	-0.48746700
C	2.43198200	-0.26737000	0.83698500
C	3.77152300	-0.17087300	1.18424200
C	4.77599300	-0.34355700	0.21406100
C	4.39634700	-0.60813200	-1.11474700
C	3.05585900	-0.69643000	-1.46141100
H	1.65831900	-0.13270400	1.58853200
H	4.05642900	0.03782700	2.21179900
H	5.16657200	-0.74072900	-1.86972500
H	2.76311000	-0.89778500	-2.48782100
O	0.74752300	-0.66119800	-0.82628600
H	0.28015400	0.40568400	-0.97975900
N	6.11293000	-0.19303200	0.54955200
H	6.33300100	-0.34529100	1.52624000
H	6.76892600	-0.64529400	-0.07534400

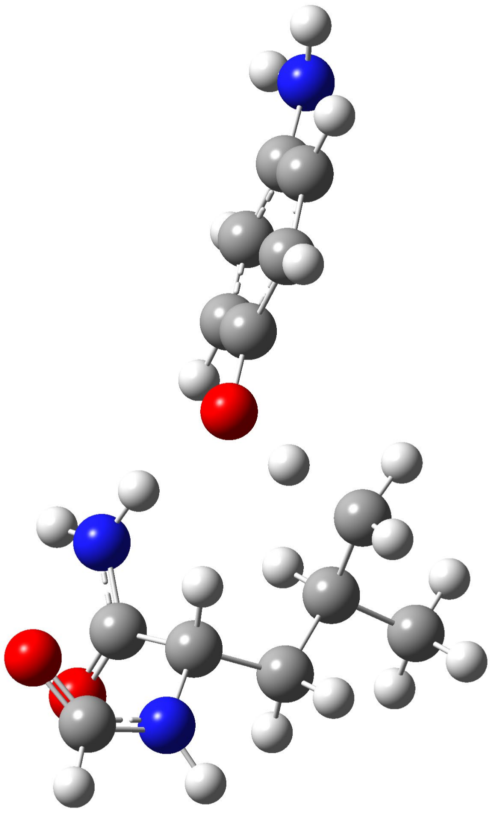

9-β-TS

Charge=0, Multiplicity=2

N	-2.46509900	-2.30384000	0.64630800
H	-1.78799700	-2.49893700	-0.07848100
H	-2.71025600	-3.03658000	1.30049800
C	-3.06577900	-1.11435500	0.73507800
O	-3.93617200	-0.85613200	1.57573100
C	-2.58263700	-0.03158100	-0.24371500
H	-1.97770300	-0.49579100	-1.03296900
N	-3.74862300	0.61038500	-0.84489200
H	-3.88417200	1.60727100	-0.72389100
C	-4.66462600	-0.08710300	-1.52577000
O	-4.59451700	-1.30814200	-1.71125500
H	-5.49506400	0.51799600	-1.91249800
C	-1.76079200	0.99586000	0.51155900
H	-2.35321400	1.54091700	1.25553500
C	-0.75098400	1.82778800	-0.24492600
H	0.06723800	1.15957500	-0.56026800
C	-0.17940400	2.90799100	0.67336400
H	-0.95280600	3.64910600	0.90661400
H	0.65585100	3.42572300	0.19194700
H	0.17705400	2.48411000	1.61736900
C	-1.32535600	2.45854100	-1.52449900
H	-1.66150800	1.70060300	-2.23761100
H	-0.55165700	3.06147200	-2.01057100
H	-2.17064900	3.11478400	-1.28740000
C	1.06263000	-0.35795800	1.31837400
C	2.12865200	0.46373000	1.71672600
C	3.35711800	0.39892600	1.07818700
C	3.57277400	-0.48909700	-0.00388700
C	2.48203900	-1.29331800	-0.41622700
C	1.26177100	-1.23191500	0.23727600
H	1.98128700	1.15352400	2.54285300
H	4.15339100	1.04545000	1.42555400
H	2.58660900	-1.97559100	-1.25062900
H	0.43964100	-1.85891500	-0.09560100
N	4.80787000	-0.58247900	-0.62258900
O	-0.12335200	-0.31177500	1.97168700
H	-0.91213800	0.26161500	1.35378000
C	5.74523500	0.51793300	-0.42623400
H	6.65265700	0.30623300	-0.99093100
H	5.33241600	1.47826200	-0.76713600
H	6.02425100	0.61132300	0.62508100
C	4.85520500	-1.20081500	-1.94307600
H	5.88425400	-1.17901100	-2.30014700
H	4.54287800	-2.24579700	-1.89691600
H	4.21786000	-0.67516600	-2.66863600

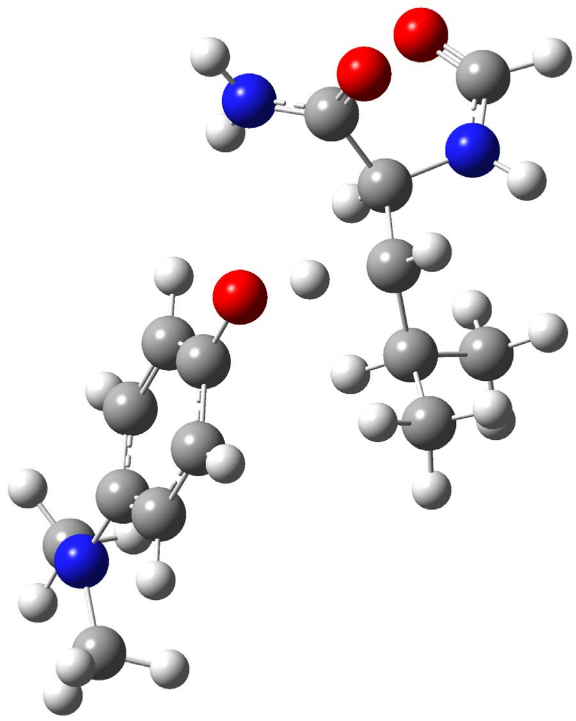

9-γ-TS

Charge=0, Multiplicity=2

N	-3.69480200	1.79204300	1.30323400
H	-3.60536800	1.25602800	2.15611400
H	-3.69778000	2.80301400	1.35687800
C	-3.79677000	1.19315000	0.11263900
O	-3.93073300	1.81641500	-0.94836200
C	-3.69020400	-0.33502300	0.11625500
H	-3.76254700	-0.69977500	1.14672500
N	-4.77251000	-0.90670200	-0.66846500
H	-4.54855400	-1.44783800	-1.49588400
C	-6.05808900	-0.66316300	-0.38984800
O	-6.43088800	0.03238800	0.56252300
H	-6.76639600	-1.13839100	-1.08063400
C	-2.35949600	-0.78766500	-0.51447000
H	-2.34560700	-0.45164400	-1.55819200
H	-2.37880300	-1.88484900	-0.51387700
C	-1.10552600	-0.31185600	0.19345700
C	-0.59483800	1.06643100	-0.15359500
H	0.44296200	1.18518900	0.18126100
H	-1.17955300	1.84926500	0.35030500
H	-0.63792800	1.24997000	-1.23197200
C	-0.97935100	-0.69286500	1.64902100
H	-1.27829300	-1.73227900	1.81882500
H	0.05245700	-0.56193500	1.99408800
H	-1.61250700	-0.05106500	2.27968500
C	1.87973200	-1.14093300	-0.58527000
C	2.45915400	-0.23221900	-1.48598000
C	3.68103800	0.36269000	-1.21272700
C	4.37185600	0.09196900	-0.00670100
C	3.78172200	-0.82861600	0.89260400
C	2.56081600	-1.42193100	0.61041000
H	1.93767000	-0.00662400	-2.41232700
H	4.09742800	1.04503600	-1.94315500
H	4.27724000	-1.08341300	1.82117700
H	2.12136700	-2.12546200	1.31249000
N	5.57122700	0.72250200	0.29365600
O	0.70012300	-1.73719100	-0.86706800
H	-0.20043400	-1.08220400	-0.38189400
C	6.29915700	1.33358700	-0.81325700
H	7.22045900	1.76810200	-0.42643400
H	6.55346500	0.60309200	-1.59503400
H	5.71837100	2.13991800	-1.26474200
C	6.41914000	0.09702300	1.30344200
H	7.32922200	0.68708500	1.40833600
H	5.92315000	0.07978400	2.27546200
H	6.69451300	-0.93227000	1.03120600

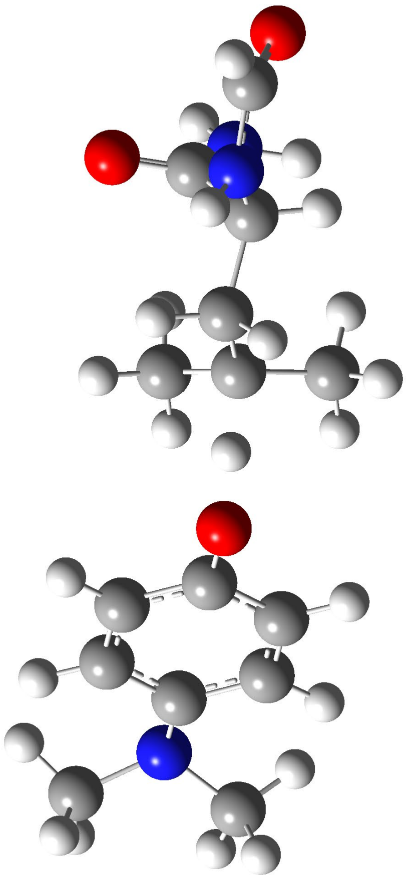

9-δ-TS

Charge=0, Multiplicity=2

N	-1.83287700	-0.93381100	1.44935600
H	-1.12587600	-0.96901200	0.71619700
H	-1.58214700	-1.18107600	2.39868500
C	-3.05470400	-0.45289100	1.20795500
O	-3.92976100	-0.35221500	2.07986800

C	-3.31251700	0.02137600	-0.22784600
H	-2.60862200	-0.48097300	-0.90182900
N	-4.66582900	-0.32742900	-0.62303700
H	-5.32824000	0.41106800	-0.83008800
C	-5.09855700	-1.59249200	-0.60178900
O	-4.38179000	-2.55390900	-0.29622100
H	-6.15137200	-1.71521500	-0.88670800
C	-3.14937700	1.54393400	-0.31716600
H	-3.79694600	2.00223000	0.44029400
H	-3.51944800	1.86112800	-1.30059500
C	-1.71702400	2.06119000	-0.15428000
H	-1.30422000	1.71641200	0.80425900
C	-1.73739700	3.59991600	-0.12033700
H	-2.13865800	3.99678100	-1.05930300
H	-0.72925200	3.99934500	0.01904600
H	-2.36780300	3.95393800	0.70184000
C	-0.81451200	1.60882300	-1.27514600
H	-1.26426000	1.59877200	-2.27208500
H	0.19508300	2.03061700	-1.25884100
C	1.33485800	-0.61632700	-0.55162000
C	1.75107300	-0.16788300	0.70937600
C	3.09961200	-0.05794800	1.01482000
C	4.09314400	-0.40423800	0.06829300
C	3.65832400	-0.82769100	-1.20987500
C	2.30738200	-0.92386600	-1.51123600
H	1.00234500	0.10318900	1.44958200
H	3.37796200	0.29661000	1.99924100
H	4.37652100	-1.08259800	-1.97899700
H	1.99054100	-1.24595600	-2.49909400
N	5.44322200	-0.34809500	0.38950000
O	0.01441800	-0.74593600	-0.84513900
H	-0.44607200	0.30702800	-1.07989300
C	5.82132100	0.48230500	1.52836800
H	6.90491200	0.45005100	1.63845700
H	5.51051700	1.52921400	1.39834900
H	5.38435100	0.10100600	2.45303400
C	6.39061700	-0.31708500	-0.71971900
H	7.39954200	-0.24914100	-0.31371500
H	6.33364200	-1.23454100	-1.30839100
H	6.21948200	0.54020000	-1.38703700

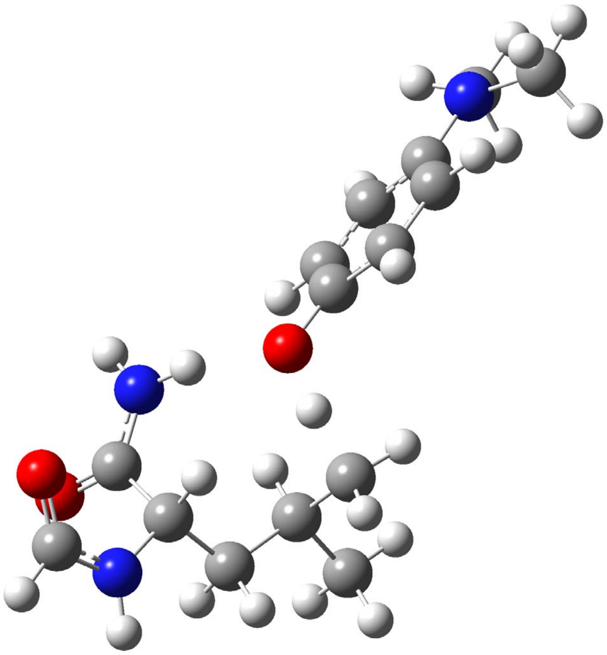

10-β-TS

Charge=0, Multiplicity=2

N	-3.18228400	-2.70353200	-0.42577400
H	-2.83621800	-2.80924800	0.51853600
H	-3.84564400	-3.37852800	-0.78590500
C	-2.76530800	-1.70488100	-1.20920600
O	-3.12999400	-1.56522000	-2.38260000
C	-1.82169900	-0.67792400	-0.55241800
H	-1.43935600	-1.10095800	0.38554500
N	-0.69411700	-0.42730200	-1.43341200
H	-0.58770500	0.48934800	-1.85446000
C	0.32873700	-1.28721000	-1.51295300
O	0.33905600	-2.39219300	-0.95426600
H	1.15959500	-0.94073000	-2.14267700

C	-2.57979500	0.60679900	-0.28688100
H	-2.64695500	1.24605300	-1.17479200
C	-3.85178200	0.55219400	0.53144800
H	-4.55558800	-0.11604000	0.00450500
C	-4.48375900	1.94347600	0.59502100
H	-3.80706300	2.64481900	1.09731100
H	-5.42195400	1.91594500	1.15713600
H	-4.69400700	2.32973000	-0.40704000
C	-3.64054600	-0.00094800	1.94542700
H	-3.15778800	-0.98328800	1.95074900
H	-4.60422400	-0.10016900	2.45393900
H	-3.01753900	0.68467100	2.53015600
C	0.98540000	2.05980200	-0.22817800
C	2.22009000	1.58891500	-0.67223300
C	2.79670600	0.44667900	-0.06048200
C	2.13242300	-0.22471400	0.98949100
C	0.90055600	0.25592500	1.44304200
C	0.33176000	1.39189000	0.83311000
C	0.18180700	-0.45345700	2.56160600
H	-0.14041400	-1.45599900	2.25289300
H	0.84174600	-0.58556600	3.42565200
H	-0.69927800	0.10386500	2.88002900
C	2.95263600	2.23140600	-1.82204200
H	3.92831000	2.62424700	-1.50902600
H	3.14093500	1.50400700	-2.62121800
H	2.39035200	3.05881700	-2.25282300
C	0.30273600	3.22218400	-0.90559300
H	-0.62170600	3.48496800	-0.39183300
H	0.94569700	4.10737200	-0.92740900
H	0.05506400	2.97671500	-1.94534000
O	-0.90047200	1.81209400	1.22286900
H	-1.68538500	1.34202900	0.54213800
C	2.72190100	-1.48170900	1.59714200
H	3.11994100	-1.25429100	2.59573600
H	1.92624400	-2.21994100	1.74634400
C	3.82625800	-2.08277300	0.72995400
H	3.38782700	-2.57626700	-0.14595800
H	4.38472900	-2.83510500	1.29343500
C	4.76022400	-0.98318500	0.25677200
H	5.56889700	-1.38386400	-0.35783600
H	5.21133200	-0.48439400	1.12837600
N	4.00113600	-0.03901800	-0.55479100
H	4.56821400	0.64851300	-1.03452100

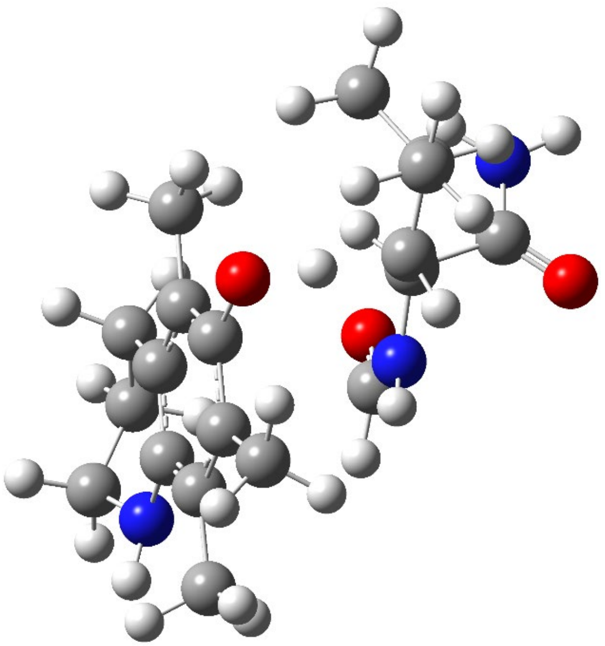

10-γ-TS

Charge=0, Multiplicity=2

N	5.00664300	0.27124000	-0.53323000
H	4.78703900	0.68588700	-1.42894900
H	5.91703900	0.43880000	-0.12320200
C	4.12427000	-0.49590200	0.11469700
O	4.37340900	-1.04381300	1.19639200
C	2.74085200	-0.63022600	-0.53016900
H	2.77670200	-0.23798800	-1.55232600
N	2.34899000	-2.02987700	-0.56949400
H	1.53230900	-2.32688600	-0.04737800

C	3.09267700	-2.95946400	-1.17982400
O	4.14053900	-2.70644800	-1.78643200
H	2.69250600	-3.97861100	-1.10278000
C	1.68773100	0.13201600	0.29703300
H	1.64807700	-0.32201800	1.29485100
H	0.72171300	-0.05551500	-0.19458600
C	1.90399900	1.62545800	0.41725000
C	2.81769200	2.10238900	1.52290600
H	2.67820700	3.17408700	1.70068400
H	3.87586300	1.95521900	1.26219900
H	2.62343800	1.56469600	2.45652500
C	2.00355500	2.37711200	-0.88876100
H	1.23873600	2.04182800	-1.59843300
H	1.88594100	3.45388500	-0.72895800
H	2.98568700	2.22437100	-1.36073500
C	-1.92614800	1.85038500	-0.52632600
C	-2.89305200	1.00435500	-1.07215400
C	-3.22963400	-0.20167500	-0.40884800
C	-2.61592000	-0.54902000	0.81487800
C	-1.64592500	0.29556500	1.36044600
C	-1.31053400	1.49853700	0.69986000
C	-0.94948600	-0.08473600	2.64070000
H	-1.63728400	-0.03590300	3.49339200
H	-0.57461900	-1.11258800	2.59076700
H	-0.10926700	0.57951300	2.84579000
C	-3.60001300	1.33408600	-2.36232700
H	-3.37320800	0.59738700	-3.14378100
H	-4.68800000	1.33247100	-2.22690300
H	-3.31550800	2.31361200	-2.74408500
C	-1.54050700	3.13586500	-1.21620000
H	-1.18703800	2.94893100	-2.23527200
H	-2.39697700	3.81473600	-1.29014500
H	-0.74887800	3.64832000	-0.67044700
O	-0.38924000	2.32278000	1.25089600
H	0.70017500	2.01355900	0.88304300
C	-2.98662700	-1.83451600	1.52876600
H	-2.16731100	-2.55814200	1.41548100
H	-3.07685400	-1.64442700	2.60342200
C	-4.28105300	-2.44718300	0.99735300
H	-5.14579500	-1.88222600	1.36405800
H	-4.38381300	-3.47789400	1.34739300
C	-4.27831300	-2.41002000	-0.52051700
H	-5.18870400	-2.85145300	-0.93098400
H	-3.42074100	-2.98747700	-0.89927900
N	-4.21142900	-1.02039200	-0.96029000
H	-4.33792300	-0.90127700	-1.95767400

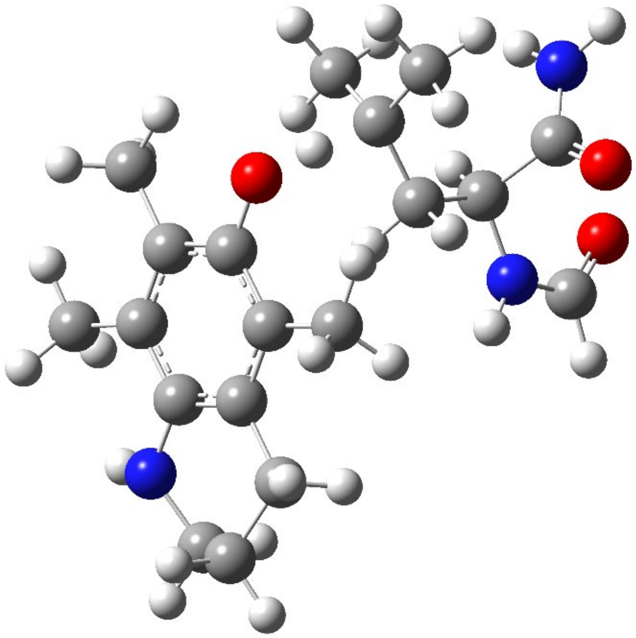

10-δ-TS

Charge=0, Multiplicity=2

N	2.69039700	1.13915000	-1.42900500
H	1.78831500	0.71278500	-1.22459200
H	2.72288700	1.97761000	-1.99600100
C	3.81437800	0.69816400	-0.85834800
O	4.92147000	1.22391300	-1.04343200
C	3.65815200	-0.48925800	0.09967700

H	2.71122300	-0.99871700	-0.11379400
N	4.75076900	-1.42699700	-0.09508700
H	5.38286000	-1.61629700	0.67412000
C	5.00724600	-1.97356600	-1.28800700
O	4.32706600	-1.75692300	-2.29887300
H	5.87737900	-2.64249000	-1.30102200
C	3.68986500	0.00223800	1.55189000
H	4.63442300	0.53751300	1.70848300
H	3.69999100	-0.88149500	2.20287200
C	2.51794800	0.89738900	1.96382200
H	2.46121700	1.76320500	1.28967900
C	2.76790700	1.43375700	3.38495000
H	2.83963400	0.60505600	4.09786400
H	1.95339700	2.09066600	3.70197600
H	3.70393800	2.00091900	3.41873300
C	1.20141200	0.16376400	1.94150200
H	1.21212600	-0.84327700	2.36982800
H	0.32755200	0.75278400	2.23571000
C	-1.54185000	1.24148100	-0.58016700
C	-2.92251200	1.44773400	-0.58417600
C	-3.78715700	0.36455200	-0.26883200
C	-3.27293200	-0.90630800	0.06224300
C	-1.88949400	-1.11730000	0.03274000
C	-1.04113000	-0.05370700	-0.31508900
C	-1.31801000	-2.46733000	0.37605400
H	-1.47650500	-2.69974100	1.43567000
H	-1.80530300	-3.26014700	-0.20104200
H	-0.24664100	-2.50303500	0.17792000
C	-3.55639800	2.77392700	-0.92258100
H	-4.20201200	2.68621800	-1.80556500
H	-4.18450000	3.13683200	-0.10052200
H	-2.82064100	3.54421700	-1.14432300
C	-0.52859400	2.32721600	-0.84457800
H	-0.10918500	2.24269400	-1.85447400
H	-0.94764400	3.32647200	-0.73740000
H	0.30599000	2.23898100	-0.14146200
O	0.30088500	-0.26213900	-0.39617000
H	0.78274800	-0.09890200	0.62956100
C	-4.19786500	-2.04830200	0.43604400
H	-4.22118500	-2.77574300	-0.38705500
H	-3.78865900	-2.58189900	1.30048700
C	-5.61923100	-1.57844800	0.73903200
H	-5.66098900	-1.11075400	1.72945200
H	-6.30636100	-2.42895500	0.74331700
C	-6.05199700	-0.56199600	-0.30234800
H	-7.07073100	-0.21406700	-0.11984300
H	-6.02547500	-1.02656600	-1.30026000
N	-5.16182800	0.59223900	-0.23590800
H	-5.46701300	1.37252200	-0.80348700

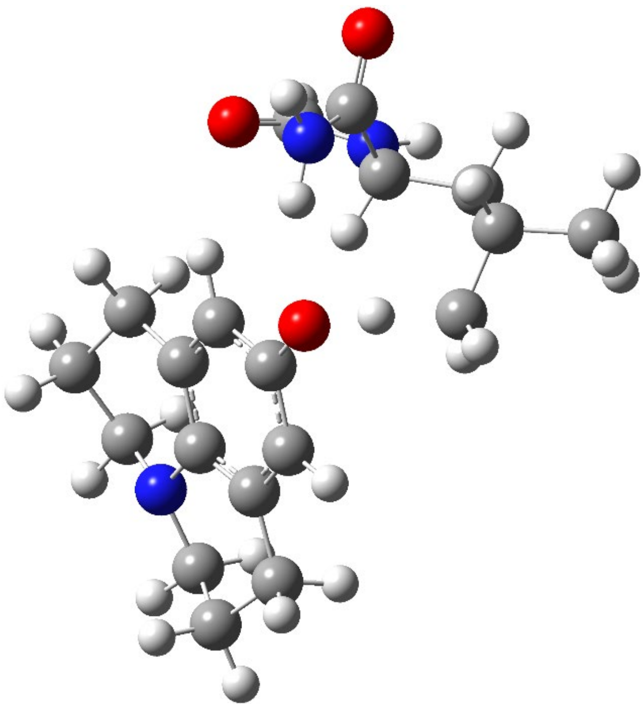

11-β-TS

Charge=0, Multiplicity=2

N	3.52515100	-2.50558200	0.18084800
H	3.25937700	-2.46144200	-0.79389200
H	4.17273100	-3.22343600	0.48190200

C	3.02280100	-1.65408900	1.08021000
O	3.28732400	-1.70836600	2.28651500
C	2.11568200	-0.54024200	0.51970000
H	1.75209400	-0.85341400	-0.46781900
N	0.97311800	-0.35856200	1.39570400
H	0.83762000	0.53421600	1.85662200
C	-0.02903300	-1.24545300	1.41521500
O	0.00313200	-2.32365700	0.80786300
H	-0.88360700	-0.94870900	2.03888900
C	2.90235200	0.74984600	0.41748700
H	3.05888400	1.21887000	1.39535800
C	4.11079000	0.80001900	-0.49245100
H	4.83443300	0.05388900	-0.12022900
C	4.76997300	2.17739200	-0.40116600
H	4.08414500	2.95224700	-0.76304700
H	5.67487600	2.21210900	-1.01497000
H	5.04435100	2.41805800	0.63048100
C	3.78545500	0.46087300	-1.95133300
H	3.31327200	-0.51960800	-2.06592200
H	4.70377700	0.45921200	-2.54606700
H	3.10949500	1.21213100	-2.37392700
C	-1.76535000	-1.27114400	-2.21304400
H	-2.07845500	-0.91597700	-3.20320400
H	-0.83320500	-1.82751300	-2.35803300
C	-2.85218300	-2.17182500	-1.63700700
H	-2.46311900	-2.74191300	-0.78563700
H	-3.19541800	-2.88709900	-2.38939500
C	-4.01970200	-1.32016600	-1.16873100
H	-4.81467100	-1.94487300	-0.75321100
H	-4.44480900	-0.76087000	-2.01957300
C	-4.68330000	0.23846400	0.60697500
H	-5.20365700	0.96374400	-0.04143500
H	-5.40103800	-0.54203400	0.87251500
C	-4.16028500	0.93733400	1.85031600
H	-3.67803300	0.19855800	2.50105800
H	-4.99461500	1.37612800	2.40417600
C	-3.16338100	2.01504000	1.44142000
H	-2.63824200	2.41830900	2.31275900
H	-3.70847100	2.85215500	0.98651500
N	-3.59109500	-0.40161000	-0.11931200
O	1.22239400	2.31252000	-0.72524500
H	2.00200100	1.65037400	-0.19064900
C	-0.30081900	0.61877000	-1.46543600
H	0.43311800	0.28434600	-2.19693600
C	-0.93679600	2.14175700	0.29416100
H	-0.69889300	2.99244600	0.92909600
C	-2.15404400	1.49148800	0.44272300
C	-2.43464400	0.34020200	-0.34392000
C	-1.49581800	-0.07462900	-1.32565600
C	0.00547400	1.71409600	-0.64932400

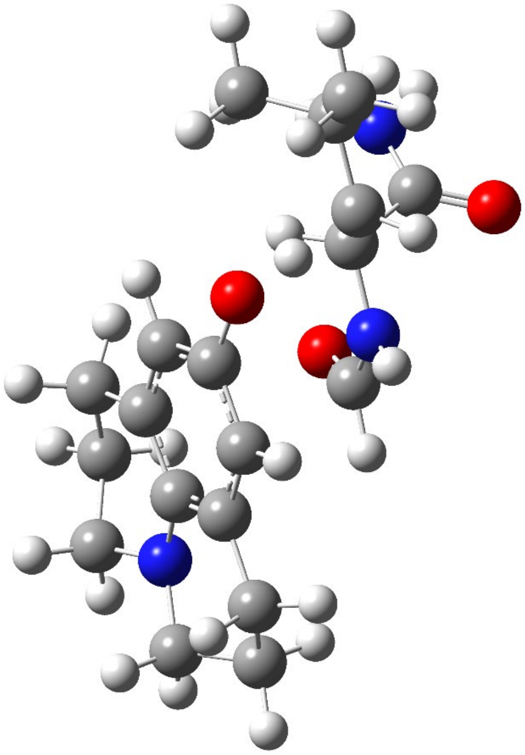

11-γ-TS

Charge=0, Multiplicity=2

N	4.35149000	0.27888700	2.22590500
H	4.31640200	1.28697300	2.15407500

H	4.29464500	-0.15202000	3.14034300
C	4.44503000	-0.48975900	1.13638200
O	4.50659900	-1.72508800	1.18261300
C	4.42606700	0.24705900	-0.20611400
H	4.55812300	1.31967400	-0.02747400
N	5.50824100	-0.23307500	-1.04980600
H	5.28386400	-0.69277100	-1.92499300
C	6.78751200	-0.18409800	-0.66149200
O	7.15660900	0.29803200	0.41609300
H	7.49461700	-0.60850600	-1.38578300
C	3.09965400	-0.00688600	-0.94804300
H	3.02851500	-1.08065400	-1.15956800
H	3.18100700	0.52060900	-1.90698300
C	1.84839500	0.45730100	-0.22866600
C	1.24706800	-0.48108100	0.78991200
H	0.21638200	-0.18612100	1.02259600
H	1.80429900	-0.45380700	1.73752300
H	1.24180500	-1.51458500	0.42865400
C	1.79475600	1.92112900	0.13739600
H	2.16044600	2.55201700	-0.67925300
H	0.76899400	2.22019000	0.38091800
H	2.40895100	2.12896300	1.02639800
C	-3.72796800	2.48431400	0.15748700
H	-3.24909200	2.80363600	1.09212100
H	-3.57664200	3.29279100	-0.56432000
C	-5.21204300	2.25021700	0.41391200
H	-5.75704500	2.17683000	-0.53398100
H	-5.63909600	3.08241300	0.98012800
C	-5.39151600	0.95714100	1.19074800
H	-6.44919500	0.76754400	1.39165200
H	-4.87475500	1.02806700	2.16331100
C	-5.25455800	-1.47790300	0.96760600
H	-4.72633500	-1.66429500	1.91850800
H	-6.32543600	-1.44775700	1.18493900
C	-4.93548100	-2.58142000	-0.02638300
H	-5.49120000	-2.39992800	-0.95331800
H	-5.26246300	-3.54452000	0.37488400
C	-3.43618300	-2.59736200	-0.29950000
H	-3.19840400	-3.24289400	-1.15053700
H	-2.91840000	-3.02074000	0.57081300
N	-4.88157500	-0.17558600	0.42475000
O	0.08387100	0.33010000	-2.04336100
H	0.95875700	0.38274200	-1.21758600
C	-1.80609500	1.33951200	-0.97335600
H	-1.36942300	2.32285400	-1.13595600
C	-1.66594000	-1.05925700	-1.18397700
H	-1.11633700	-1.94214900	-1.50399800
C	-2.89520000	-1.20613100	-0.55587400
C	-3.61061300	-0.05229000	-0.13589100
C	-3.03576600	1.23125100	-0.33690000
C	-1.10823500	0.20651500	-1.41588400

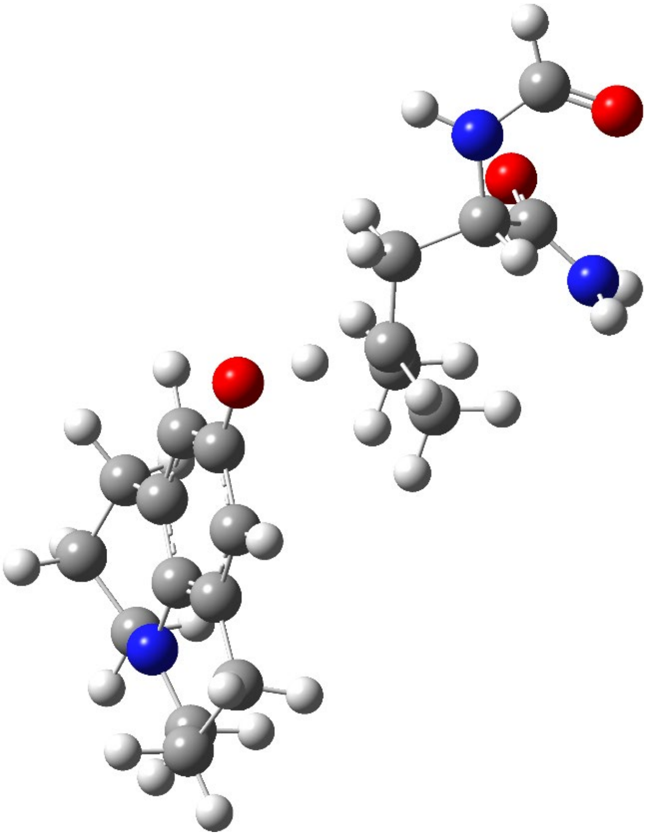

11-δ-TS
Charge=0, Multiplicity=2

N	3.15088300	-0.60497500	-1.81256900

H	2.33616700	-0.01210800	-1.95845200
H	3.73525900	-0.85953000	-2.59907800
C	3.48179800	-1.03028800	-0.59050000
O	4.46298400	-1.75221200	-0.36209300
C	2.59650200	-0.51920900	0.55058000
H	1.64866200	-0.16404200	0.13503300
N	2.31717900	-1.59485500	1.48752300
H	2.57689100	-1.48234700	2.46065600
C	1.78893200	-2.76025300	1.09661500
O	1.48602100	-3.01870900	-0.07452100
H	1.64428900	-3.48269900	1.91043200
C	3.30473100	0.61984100	1.29017100
H	4.26348600	0.24255300	1.66630500
H	2.69057600	0.88742300	2.15989800
C	3.54160000	1.87874500	0.45156900
H	4.13760800	1.62274700	-0.43469200
C	4.35946800	2.88769600	1.27832900
H	3.80461000	3.18508500	2.17491000
H	4.57579200	3.78589100	0.69373300
H	5.30871700	2.44116700	1.59191600
C	2.25614800	2.53852600	0.01662900
H	1.46019100	2.55834400	0.76971600
H	2.38160700	3.49426400	-0.49831900
C	-3.42148300	2.54134800	0.18791400
H	-3.07879800	2.91461300	1.16161200
H	-3.41752000	3.39696000	-0.49442200
C	-4.82610500	1.96957800	0.34053400
H	-5.28828000	1.82277500	-0.64218100
H	-5.45985100	2.65716200	0.90720700
C	-4.75161300	0.63288000	1.05842300
H	-5.74893100	0.20334700	1.18458100
H	-4.31789300	0.76980200	2.06404800
C	-4.05223900	-1.69495400	0.76633500
H	-3.54897900	-1.79723900	1.74322900
H	-5.11175200	-1.91885600	0.91573700
C	-3.43349600	-2.65427600	-0.23637900
H	-3.96374200	-2.56585000	-1.19135100
H	-3.55214800	-3.68224900	0.11677000
C	-1.95790300	-2.31688600	-0.41357900
H	-1.52657900	-2.85710100	-1.26219500
H	-1.40524000	-2.64330800	0.47768500
N	-3.95902500	-0.31948600	0.28709500
O	0.90602500	1.39856600	-1.81943100
H	1.58302400	1.87289900	-0.98974800
C	-1.23044000	1.91198900	-0.85325900
H	-1.01608400	2.97479200	-0.94665800
C	-0.54047000	-0.38079700	-1.13572200
H	0.20495200	-1.10420400	-1.45945200
C	-1.73959900	-0.82955000	-0.59600400
C	-2.71861600	0.11238400	-0.18503300
C	-2.43816000	1.49993400	-0.30355200
C	-0.27231000	0.98558500	-1.28022200

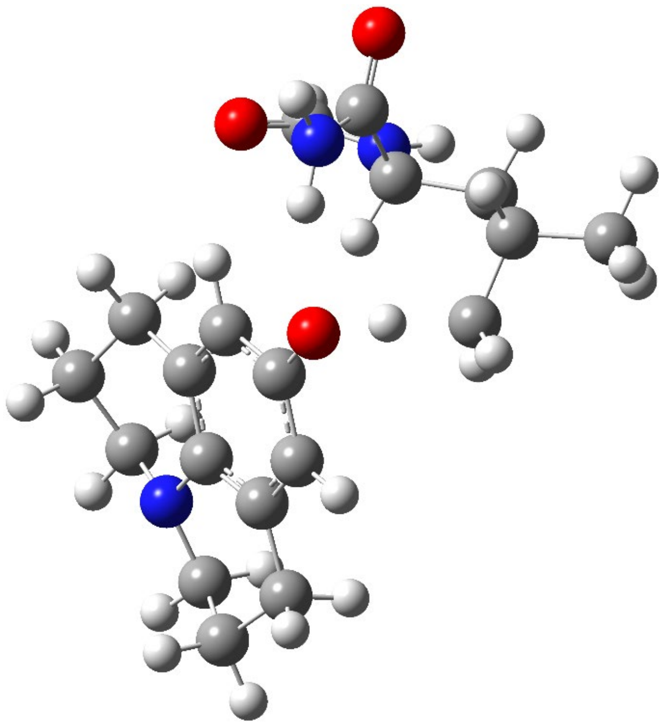

12-β-TS

Charge=0, Multiplicity=2

N	5.07261500	-0.29738100	1.07278200
H	5.40778600	0.09299600	0.20212500
H	5.71076800	-0.37381000	1.85518000
C	3.81265300	-0.72093700	1.20804200
O	3.37500900	-1.23607600	2.24214800
C	2.89094700	-0.47212800	-0.00261900
H	3.51216000	-0.33600500	-0.89504600
N	2.01870300	-1.61516700	-0.19622600
H	1.01479300	-1.49620700	-0.11383400
C	2.51210500	-2.84435100	-0.38730500
O	3.72285700	-3.08913300	-0.44600600
H	1.74886500	-3.62593700	-0.49272600
C	2.05638100	0.76345100	0.26951600
H	1.30817400	0.58519100	1.05307800
C	2.74872700	2.10497800	0.38447700
H	3.43862100	2.04054100	1.24304400
C	1.72457400	3.19969600	0.68800900
H	1.05248400	3.34039800	-0.16595800
H	2.22945900	4.15059200	0.88142100
H	1.11853900	2.94666100	1.56379000
C	3.56109300	2.46271200	-0.86320800
H	4.36896200	1.75207800	-1.05975200
H	4.01069000	3.45289200	-0.74417000
H	2.90849500	2.48913200	-1.74355400
C	-1.25204300	-0.64828400	-1.74088400
C	-0.75051000	0.58930700	-1.30721300
C	-1.54322500	1.40792000	-0.48039700
C	-2.81625600	1.00391100	-0.11534700
C	-3.34209400	-0.22571100	-0.56234700
C	-2.53521000	-1.03659400	-1.38123700
H	-0.62997800	-1.27909600	-2.36915100
H	-1.13936500	2.35643900	-0.13813700
H	-3.41121700	1.65241300	0.52019700
H	-2.92736900	-1.98728600	-1.73245500
O	0.47510400	0.99442300	-1.70039800
H	1.26085600	0.89218100	-0.78061400
C	-4.68429500	-0.69662000	-0.21337200
H	-4.96539700	-1.65879600	-0.64229100
C	-5.58112400	-0.06498100	0.56969500
H	-5.34960900	0.89731300	1.02518100
C	-6.89803300	-0.61701000	0.85493700
H	-7.13383900	-1.57868500	0.39975700
C	-7.80273800	-0.00384200	1.63172300
H	-7.58647900	0.95642000	2.09467200
H	-8.77608500	-0.44440400	1.82256900

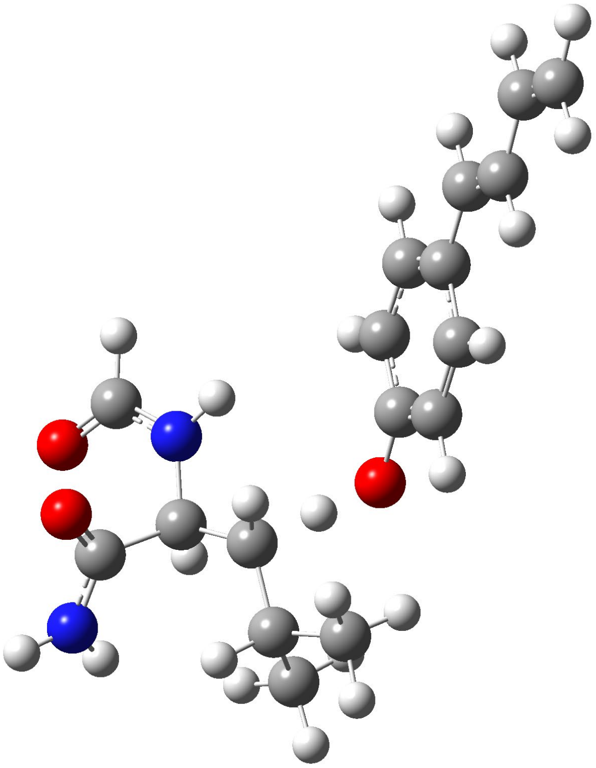

12-γ-TS

Charge=0, Multiplicity=2

N	-3.80024500	-2.35574700	-0.19592300
H	-3.73906800	-2.34014200	-1.20510700
H	-3.71441400	-3.24067700	0.28860000
C	-3.97996500	-1.23228700	0.50537500
O	-4.08362300	-1.21349900	1.73857500
C	-4.00856400	0.06829900	-0.30400600
H	-4.08675600	-0.17161200	-1.36982200
N	-5.15775800	0.86728000	0.08924400
H	-5.00509200	1.77366700	0.51642600
C	-6.40947700	0.40221400	0.00370600
O	-6.69473900	-0.71545900	-0.44227800
H	-7.17465800	1.10245500	0.36274200
C	-2.74302900	0.90488200	-0.04088200
H	-2.72423100	1.17282500	1.02220100
H	-2.86133900	1.82921600	-0.61978400
C	-1.43057800	0.24378700	-0.42442600
C	-0.81408000	-0.69546000	0.58631800
H	0.23665800	-0.89023200	0.33899800
H	-1.32370600	-1.66907100	0.58408300
H	-0.86651700	-0.28467200	1.59956500
C	-1.30850300	-0.20241200	-1.86335500
H	-1.70295200	0.55156700	-2.55166000
H	-0.26119900	-0.39968800	-2.11712500
H	-1.86177000	-1.13816300	-2.02946300
C	1.90510700	1.48104200	1.23838400
C	1.42348800	1.60275600	-0.07775400
C	2.20903000	1.12477800	-1.14697200
C	3.44880700	0.55843700	-0.90435700
C	3.94801400	0.44473700	0.40953800
C	3.15044900	0.91668200	1.46940400
H	1.28829500	1.83708500	2.05840200
H	1.82394000	1.21419900	-2.15872500
H	4.03713400	0.19965800	-1.74328800
H	3.52295100	0.83141400	2.48687200
O	0.22716300	2.16425800	-0.31298200
H	-0.64004400	1.24789500	-0.37203600
C	5.25378300	-0.13904800	0.72135700
H	5.49846300	-0.18944900	1.78257100
C	6.16080600	-0.60141400	-0.16214500
H	5.96741400	-0.56863300	-1.23391000
C	7.43923600	-1.16374800	0.24969300
H	7.63660100	-1.19634800	1.32099900
C	8.35412300	-1.62810800	-0.61371600
H	8.17640200	-1.60478700	-1.68656000
H	9.29762700	-2.04046000	-0.27072900

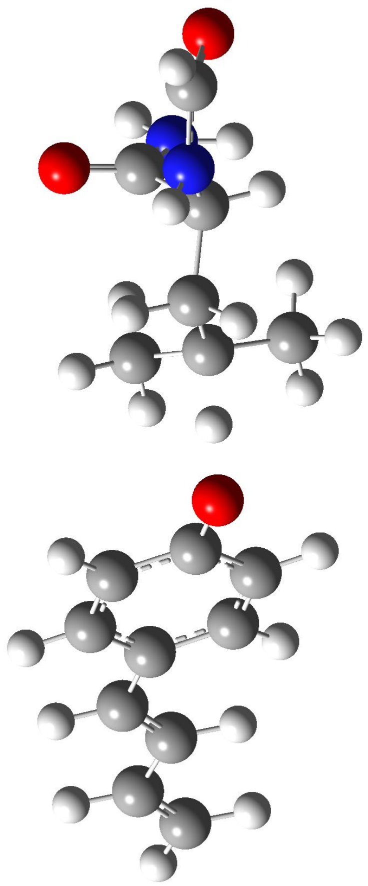

12-δ-TS

Charge=0, Multiplicity=2

N	-2.28924500	-0.88630700	1.53246400
H	-1.51444300	-0.87899900	0.87211500
H	-2.11474300	-1.09692800	2.50732000
C	-3.51734500	-0.51731000	1.15907200
O	-4.48032500	-0.47474900	1.93771400
C	-3.66830300	-0.09386600	-0.30728500
H	-2.84823300	-0.52881200	-0.88948800
N	-4.92738400	-0.58846800	-0.83506300
H	-5.63250800	0.07281300	-1.13931600
C	-5.23077700	-1.89078200	-0.82143600
O	-4.46098700	-2.76666100	-0.40708100
H	-6.22687900	-2.12713700	-1.21726100
C	-3.65593600	1.43581000	-0.41606300
H	-4.45006800	1.82900300	0.23040400
H	-3.91284600	1.69907000	-1.45014400
C	-2.32588800	2.10607400	-0.06076600
H	-2.01951500	1.81200800	0.95210200
C	-2.51320300	3.63412900	-0.06317900
H	-2.81909800	3.97941300	-1.05673600
H	-1.58354200	4.14180900	0.20738300
H	-3.28744100	3.92050400	0.65579900
C	-1.22689400	1.75781800	-1.03391800
H	-1.51567700	1.77394700	-2.08913500
H	-0.26848200	2.25102600	-0.84451600
C	1.37541200	0.02841500	0.91425100
C	0.99110600	-0.40761400	-0.36237000
C	1.97252200	-0.68426700	-1.32935900
C	3.31536300	-0.55869000	-1.01198700
C	3.71889700	-0.13213300	0.26854900
C	2.72325800	0.16267800	1.21798100
H	0.60752800	0.25758900	1.64835700
H	1.65904500	-1.00592400	-2.31792500
H	4.05822200	-0.78675400	-1.76991300
H	3.02123100	0.49946600	2.20735000
O	-0.31526700	-0.57297600	-0.66675500
H	-0.82827000	0.50925200	-0.85952200
C	5.12448200	0.02248400	0.65248200
H	5.29258400	0.39191200	1.66437000
C	6.20060500	-0.24875700	-0.11161800
H	6.08662600	-0.62559500	-1.12762000
C	7.56536600	-0.06236300	0.36162600
H	7.68296400	0.31484500	1.37727700
C	8.64798100	-0.33083900	-0.38241900
H	8.55134100	-0.70789000	-1.39815800
H	9.65136500	-0.18083300	0.00306000

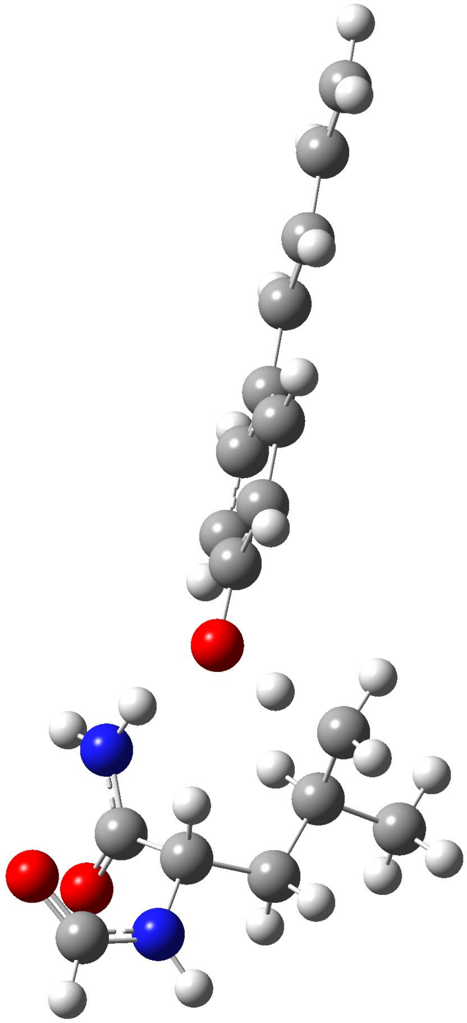

13-β-TS

Charge=0, Multiplicity=2

N	6.32434900	0.47183400	1.41170500
H	6.54025500	1.16418000	0.70657500
H	6.87696200	0.44351200	2.25970700
C	5.33363600	-0.40879800	1.24173800
O	5.05864300	-1.29158700	2.06086600
C	4.48737500	-0.21606600	-0.03289300
H	5.07271200	0.35960800	-0.75833400
N	4.15135900	-1.50762000	-0.60200000
H	3.17770100	-1.74741400	-0.75143300
C	5.09868800	-2.39084100	-0.93734800
O	6.30872900	-2.17969600	-0.79151900
H	4.71303400	-3.32814300	-1.35777700
C	3.21987600	0.52243500	0.34962100
H	2.57597900	-0.08514500	0.99939900
C	3.31869900	1.97078200	0.77648000
H	3.90766800	1.99170200	1.70915300
C	1.92448900	2.51944200	1.08640500
H	1.33296200	2.59559200	0.16723900
H	1.99627600	3.51873900	1.52539400
H	1.38631100	1.87218100	1.78656800
C	4.02091800	2.85191800	-0.25958500
H	5.06144400	2.55945700	-0.42820000
H	4.01854000	3.89382300	0.07392500
H	3.49169600	2.80053500	-1.21846200
C	-1.21813300	-1.25440800	-0.37164000
C	-2.19229200	-0.25286100	-0.53708500
C	-1.79135300	0.98184900	-1.08650000
C	-0.47477600	1.20167400	-1.45474300
C	0.48317600	0.18104700	-1.30817900
C	0.10038700	-1.05061300	-0.75385600
H	-1.51085500	-2.20797200	0.05964700
H	-2.51214700	1.78354600	-1.21322700
H	-0.16229700	2.15686700	-1.86639100
H	0.84412700	-1.83239900	-0.62730600
C	-3.56837900	-0.54073200	-0.12195100
H	-3.70121300	-1.50270800	0.37045600
C	-4.63297200	0.26002300	-0.31317700
H	-4.50594200	1.20640600	-0.83664700
C	-6.01219900	-0.02300800	0.10888000
C	-6.36227100	-1.12173200	0.91337200
C	-7.03083300	0.84810100	-0.30875200
C	-7.68770200	-1.34182700	1.27354400
H	-5.59657300	-1.80498000	1.26821300
C	-8.35861500	0.62734200	0.05238500
H	-6.77227900	1.70540100	-0.92511000
C	-8.69297500	-0.47074300	0.84385600
H	-7.93910500	-2.19484500	1.89656100
H	-9.13023600	1.31287700	-0.28440300
H	-9.72561500	-0.64611000	1.12894300
O	1.75235100	0.39319400	-1.71490600
H	2.48438700	0.50260000	-0.75137300

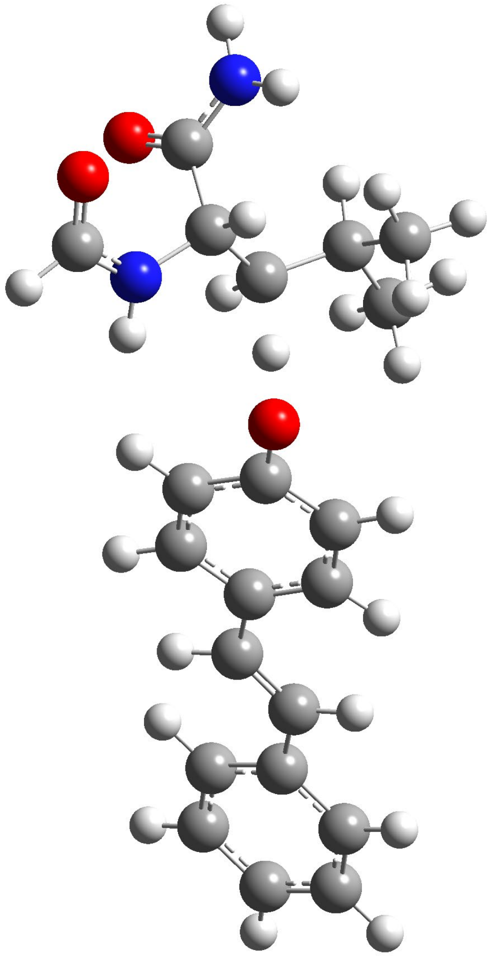

13-γ-TS

Charge=0, Multiplicity=2

N	4.84418800	2.00913400	-1.49650900
H	5.00544900	1.39015200	-2.27995200
H	4.59043400	2.97313300	-1.67417200
C	4.96390100	1.57969300	-0.23654500
O	4.80254000	2.31369500	0.74678600
C	5.27089000	0.08902900	-0.05970600
H	5.54081200	-0.34149500	-1.02999800
N	6.38400100	-0.07811300	0.86084900
H	6.23175700	-0.56791800	1.73499400
C	7.57875600	0.48257800	0.64142000
O	7.84509300	1.15273000	-0.36324300
H	8.31533700	0.29595400	1.43336200
C	4.05684400	-0.65210800	0.53199800
H	3.85022700	-0.23147000	1.52336700
H	4.36905100	-1.69514600	0.66573500
C	2.79555400	-0.61501100	-0.31286300
C	1.92148100	0.61160800	-0.18673600
H	0.92570800	0.41944400	-0.60459600
H	2.34099600	1.45567700	-0.75139900
H	1.81092400	0.91979600	0.85777100
C	2.92517000	-1.16200700	-1.71587800
H	3.49441300	-2.09672700	-1.72951600
H	1.93670700	-1.34302300	-2.15192100
H	3.44011300	-0.44142400	-2.36772800
C	-1.90401400	-1.84653600	-0.90818200
C	-2.60043800	-1.05382400	0.02345000
C	-1.94434500	-0.70128800	1.22115500
C	-0.65070800	-1.12303800	1.47310400
C	0.03303900	-1.92571300	0.53668300
C	-0.60819300	-2.27821800	-0.66433100
H	-2.39744000	-2.12288500	-1.83643500
H	-2.44740800	-0.08293700	1.95788000
H	-0.13996200	-0.84168500	2.38958100
H	-0.07452300	-2.88898800	-1.38679300
C	-3.96570400	-0.63132000	-0.30148500
H	-4.31040700	-0.94052600	-1.28678300
C	-4.78538300	0.07396000	0.50019700
H	-4.44393900	0.36648900	1.49185100
C	-6.15256300	0.50678500	0.17769600
C	-6.76128000	0.28419600	-1.07020400
C	-6.88833800	1.18010800	1.16593900
C	-8.06101400	0.71699900	-1.31150500
H	-6.21942700	-0.22571700	-1.86073000
C	-8.19028900	1.61411300	0.92371800
H	-6.42816800	1.36178400	2.13400800
C	-8.78295800	1.38314400	-0.31694400
H	-8.51426900	0.53658100	-2.28160100
H	-8.73989500	2.13214500	1.70371700
H	-9.79661800	1.71961400	-0.51104200
O	1.28654500	-2.33628800	0.78785900
H	2.08693300	-1.50797600	0.27292600

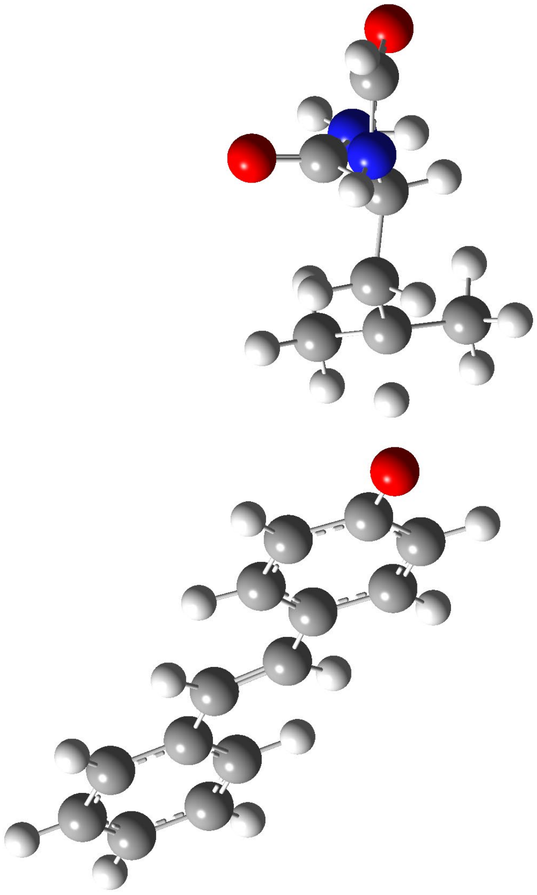

13-δ-TS

Charge=0, Multiplicity=2

N	-3.45886400	-0.78935800	1.55419700
H	-2.76694700	-0.87550600	0.81199800
H	-3.17748100	-0.92070900	2.51785500
C	-4.70181500	-0.37712700	1.29227200
O	-5.56089600	-0.20738800	2.16889000
C	-5.00520900	-0.07213500	-0.17984000
H	-4.28526100	-0.60606300	-0.81093100
N	-6.34403000	-0.52885200	-0.51075500
H	-7.04035500	0.14665500	-0.80393500
C	-6.71682900	-1.80112200	-0.33611400
O	-5.95516200	-2.68598300	0.07421400
H	-7.76425900	-2.00403200	-0.59384900
C	-4.92637500	1.43948800	-0.42864200
H	-5.61253600	1.93440800	0.26948200
H	-5.29656800	1.63030600	-1.44416300
C	-3.53100500	2.05686200	-0.29951500
H	-3.11401800	1.83286300	0.69201300
C	-3.64438800	3.58749000	-0.41830100
H	-4.05148900	3.86508100	-1.39665700
H	-2.66547300	4.06085600	-0.30525600
H	-4.31106600	3.97836600	0.35701300
C	-2.58053100	1.56339700	-1.36292300
H	-3.00763300	1.47140000	-2.36596100
H	-1.59628200	2.04203400	-1.35920100
C	1.41615100	0.24468600	0.90411100
C	2.39352200	-0.18596500	-0.01179000
C	1.96288300	-0.78067400	-1.21416700
C	0.61349600	-0.93836200	-1.48705500
C	-0.34929800	-0.52678300	-0.54952200
C	0.06228000	0.07896300	0.64707900
H	1.73323900	0.71392200	1.83166100
H	2.68817100	-1.11555000	-1.94902600
H	0.28071600	-1.38955700	-2.41689500
H	-0.68800600	0.41453200	1.35803000
C	3.80587500	0.01250300	0.33072900
H	3.97199700	0.52758200	1.27557500
C	4.86088600	-0.38183700	-0.40544200
H	4.69461700	-0.90124700	-1.34802800
C	6.27696300	-0.18242400	-0.06379400
C	6.70611900	0.44719700	1.11794100
C	7.25135000	-0.64416500	-0.96283400
C	8.06212200	0.60724200	1.38323800
H	5.98020600	0.81379600	1.83724400
C	8.61001400	-0.48352400	-0.69693900
H	6.93320200	-1.13392700	-1.87972900
C	9.02140100	0.14369700	0.47822800
H	8.37418200	1.09599600	2.30127100
H	9.34530500	-0.84879200	-1.40738500
H	10.07835800	0.27113300	0.69033600
O	-1.66172200	-0.72604400	-0.80516800
H	-2.18580400	0.33480300	-1.08936200

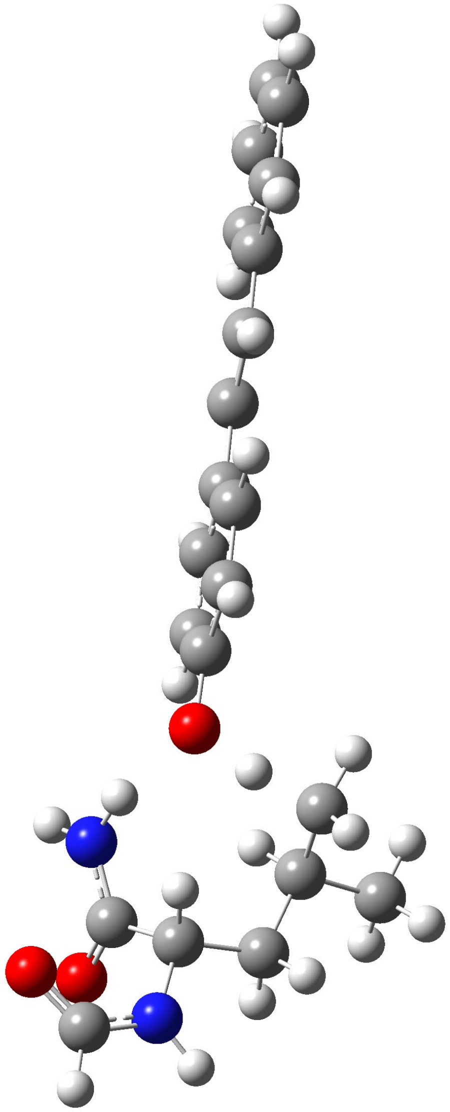

Cartesian coordinates of the optimized antioxidant species studied at the M06-2X(SMD)/6-31++G(d,p) level of theory in pentyl ethanoate.

Leucine

Charge=0, Multiplicity=1

N	-0.59159300	2.06898800	0.50759300
H	-0.65558600	1.84261200	1.48968600
H	-0.77457900	3.02429300	0.22879000
C	-0.64466300	1.09913100	-0.43175600
O	-0.79395300	1.32475500	-1.62608000
C	-0.42262500	-0.33344300	0.07952200
H	-0.46800300	-0.34747800	1.17295600
N	-1.47612400	-1.20101000	-0.41293700
H	-1.31787800	-1.73081300	-1.26107000
C	-2.73377700	-1.11853600	0.06914200
O	-3.06685300	-0.38456600	0.99080200
H	-3.44217300	-1.79282100	-0.43785000
C	0.93253900	-0.84988200	-0.41254100
H	0.90072700	-0.88191200	-1.50943900
C	2.14122500	-0.01761400	0.03123700
H	2.00277900	1.01326100	-0.32385100
C	3.40812700	-0.57501200	-0.61866300
H	3.58967300	-1.60501500	-0.28768700
H	4.28381600	0.02319300	-0.34724700
H	3.32668900	-0.58019000	-1.71058600
C	2.29287300	0.00893700	1.55320100
H	1.44539000	0.49098200	2.05091000
H	3.19570800	0.55756800	1.84061600
H	2.38011000	-1.01142900	1.94724300
H	1.05160000	-1.88110100	-0.05368700

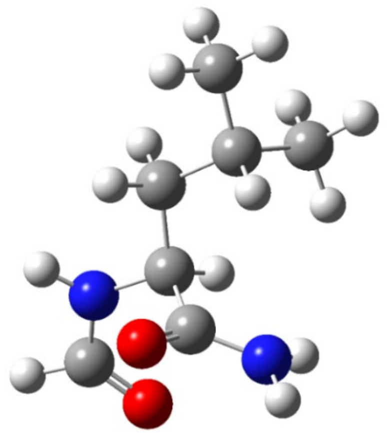

Leucine-β-dmg

Charge=0, Multiplicity=2

N	-0.47836900	2.09198000	0.49097500
H	-0.21781400	1.88217200	1.44429000
H	-0.76102300	3.03947000	0.27702900
C	-0.82023800	1.10528500	-0.36918900
O	-1.35323100	1.30110500	-1.45062400
C	-0.36738700	-0.31167400	0.06746000
H	-0.31164200	-0.33470000	1.16215100
N	-1.33931500	-1.29310000	-0.36326300
H	-1.13553200	-1.87551600	-1.16505500
C	-2.59853100	-1.26443700	0.11935100
O	-2.97134300	-0.49658400	0.99691900
H	-3.26363700	-2.01290800	-0.33922200
C	0.97013700	-0.58590700	-0.54406100
H	0.96167400	-0.93758000	-1.57515600
C	2.23196300	0.03094500	-0.02342700
H	2.22086000	1.10746900	-0.27232000
C	3.44530100	-0.59566000	-0.71587400
H	3.51737500	-1.66133100	-0.46917100
H	4.37050100	-0.10838900	-0.39361900
H	3.37278900	-0.50232500	-1.80413800
C	2.36356700	-0.08867300	1.49980900

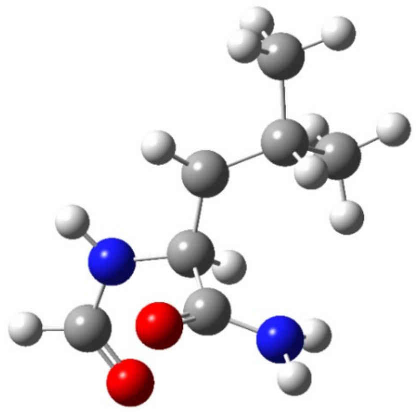

H	1.57522100	0.45334500	2.03094800
H	3.32312000	0.32273900	1.82796800
H	2.31963200	-1.14004800	1.80753200

Leucine-γ-dmg

Charge=0, Multiplicity=2

N	-0.02341300	1.62725100	1.14743800
H	-0.11258800	1.10130400	2.00506400
H	-0.00133300	2.63650300	1.21544100
C	-0.34588200	1.06617100	-0.03982100
O	-0.49317800	1.70650000	-1.07255800
C	-0.42693600	-0.46735400	-0.03823300
H	-0.44664900	-0.83262700	0.99451900
N	-1.63889700	-0.90861400	-0.69800500
H	-1.59384100	-1.19493200	-1.66818800
C	-2.85087000	-0.67873600	-0.15021600
O	-3.01482000	-0.17679400	0.95423900
H	-3.68988900	-1.00121500	-0.78697500
C	0.80211900	-1.04519900	-0.77149400
H	0.75366000	-0.71699600	-1.81717300
H	0.69457400	-2.13870500	-0.75163100
C	2.10629500	-0.63686900	-0.15906000
C	2.83827900	0.55946700	-0.67424800
H	3.91930000	0.45632700	-0.51926500
H	2.53428400	1.48094300	-0.14868300
H	2.65485700	0.72152400	-1.74090500
C	2.44120000	-1.13261100	1.21047700
H	1.99106600	-2.11076700	1.41236500
H	3.52570700	-1.21825800	1.34815800
H	2.08577100	-0.44042800	1.99337400

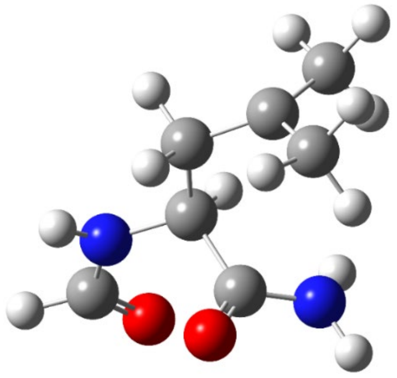

Leucine-δ-dmg

Charge=0, Multiplicity=2

N	-0.37176800	2.07977900	0.40008000
H	-0.19202200	1.90748100	1.37908300
H	-0.49556700	3.03395000	0.08925500
C	-0.61419500	1.06735900	-0.45832400
O	-0.93061100	1.23541200	-1.62974400
C	-0.39778500	-0.34270000	0.11505200
H	-0.42480200	-0.29440300	1.20859100
N	-1.46108900	-1.22563300	-0.31953300
H	-1.31672300	-1.79399700	-1.14484700
C	-2.72286900	-1.07303200	0.13641300
O	-3.04396200	-0.27539500	1.00721600
H	-3.44562900	-1.75444300	-0.33999800
C	0.95042600	-0.90207100	-0.35200100
H	0.91939400	-1.02550100	-1.44215000
H	1.07148600	-1.89906800	0.09189400
C	2.15653600	-0.03114400	0.02336100
H	2.05995200	0.93979400	-0.48099500
C	3.44904400	-0.69196400	-0.49020100
H	3.59376300	-1.66933700	-0.01635800
H	4.32036200	-0.07150600	-0.26091700
H	3.40655900	-0.83732200	-1.57537000
C	2.23552300	0.19985200	1.49808200

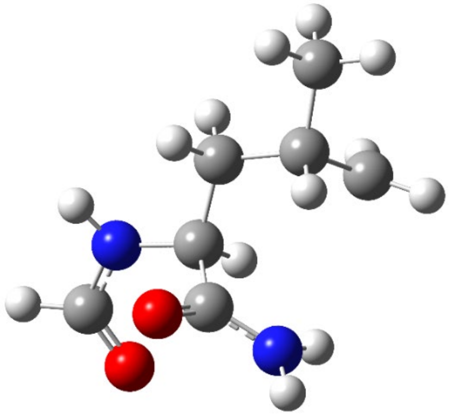

H	2.03490600	-0.62034000	2.18312400
H	2.75482900	1.06774500	1.89079500

1

Charge=0, Multiplicity=1

C	-1.95650500	1.40794600	-0.20174400
C	-1.92055300	0.02547100	-0.32167700
C	-0.70422800	-0.65428500	-0.25259300
C	0.51345500	0.00751200	-0.06388300
C	0.45344500	1.41225600	0.05767000
C	-0.76033100	2.09528700	-0.01389200
H	-2.90291500	1.93641400	-0.26392600
H	-0.73238600	-1.73284200	-0.36292000
H	-0.76553300	3.17956300	0.07482200
O	1.61848100	2.10722700	0.24510700
H	1.42367400	3.05222300	0.30301900
O	-3.08380300	-0.68046500	-0.54076800
C	-3.68534100	-1.15443200	0.65967500
H	-4.58738100	-1.69662000	0.37087300
H	-3.00925400	-1.83092100	1.19674700
H	-3.95564900	-0.31853100	1.31663900
C	1.84483400	-0.75296900	-0.00198300
C	1.64087500	-2.26471200	-0.16276900
H	1.01225600	-2.68019000	0.63235800
H	1.19187300	-2.51718000	-1.12935400
H	2.61524800	-2.76093400	-0.10919500
C	2.77080200	-0.28554100	-1.14010100
H	3.00716700	0.77703300	-1.06036600
H	3.70921100	-0.85092900	-1.10581600
H	2.30242500	-0.46536400	-2.11420700
C	2.52378700	-0.51847500	1.36000300
H	3.46271400	-1.08233800	1.40394400
H	2.74909700	0.53655500	1.52516400
H	1.88058700	-0.86838200	2.17526900

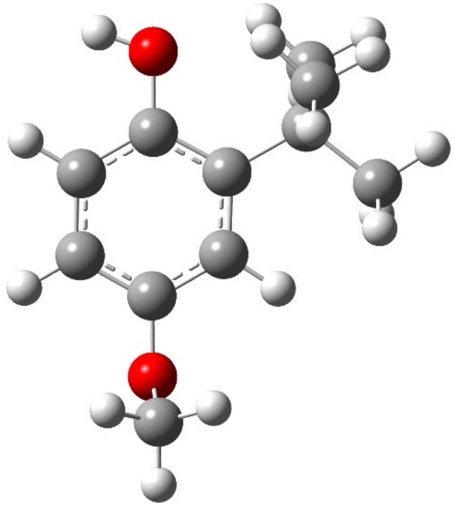

1-dmg

Charge=0, Multiplicity=2

C	1.89099400	1.65726400	-0.00022300
C	1.94498800	0.24098000	0.00024800
C	0.76096900	-0.52762800	0.00018200
C	-0.48933000	0.06224700	0.00001300
C	-0.57139200	1.52893700	0.00002200
C	0.67591600	2.27415800	-0.00044800
H	2.82322400	2.21335200	-0.00046400
H	0.84373000	-1.60631700	0.00034500
H	0.59194300	3.35662400	-0.00083300
O	-1.66210600	2.14685600	0.00050500
O	3.17527200	-0.28593300	0.00050700
C	3.32429800	-1.70428400	-0.00028500
H	2.87592100	-2.14195500	0.89670200
H	2.87650200	-2.14105400	-0.89797600
H	4.39788300	-1.88688500	-0.00005500
C	-1.77012900	-0.76876000	-0.00005000
C	-1.47871700	-2.27433200	-0.00061000
H	-0.92021600	-2.58396400	-0.89107200

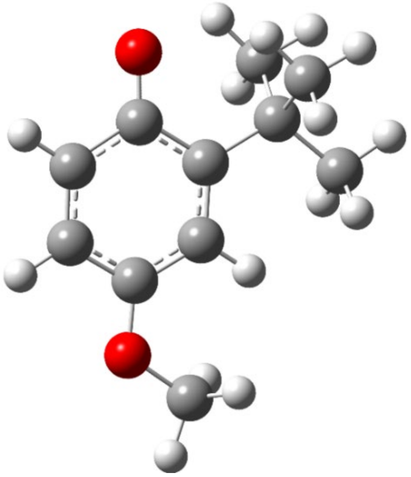

H	-0.91961000	-2.58450200	0.88930900
H	-2.42837700	-2.81855900	-0.00041700
C	-2.59534700	-0.44699200	1.26101500
H	-2.88865300	0.60358000	1.28608000
H	-3.50085800	-1.06422900	1.26949400
H	-2.02223000	-0.67482300	2.16694100
C	-2.59578500	-0.44604700	-1.26067600
H	-3.50108200	-1.06358700	-1.26958800
H	-2.88948400	0.60444100	-1.28474300
H	-2.02281100	-0.67276800	-2.16694000

2

Charge=0, Multiplicity=1

C	-1.26493800	1.66149600	0.00002000
C	-0.01794500	1.03470000	0.00002000
C	0.07738400	-0.37769800	-0.00000500
C	-1.12132600	-1.09487200	-0.00002900
C	-2.36945100	-0.46673700	-0.00003000
C	-2.44421400	0.91690400	-0.00000500
H	-1.33629200	2.74238000	0.00004000
H	-3.41147600	1.40882400	-0.00000600
O	-3.53949500	-1.17611600	-0.00005300
H	-3.34880000	-2.12345600	-0.00006700
O	1.15285800	1.74184600	0.00004600
C	1.08150700	3.15547100	0.00005400
H	0.57237500	3.53040000	0.89573600
H	2.11375000	3.50696200	0.00006000
H	0.57238100	3.53040900	-0.89562700
H	-1.09900100	-2.18005600	-0.00005000
C	1.43416600	-1.09833900	-0.00000500
C	1.27053800	-2.62435700	-0.00004900
H	0.74056700	-2.98099000	0.88985600
H	0.74058600	-2.98094000	-0.88998600
H	2.26352800	-3.08520000	-0.00005200
C	2.23285700	-0.72354700	-1.26245100
H	2.44274400	0.34622200	-1.30664200
H	3.18642900	-1.26416500	-1.26685300
H	1.67944200	-1.00723400	-2.16473500
C	2.23281900	-0.72361500	1.26248400
H	3.18639400	-1.26423000	1.26688300
H	2.44270200	0.34615200	1.30674000
H	1.67938000	-1.00735400	2.16473700

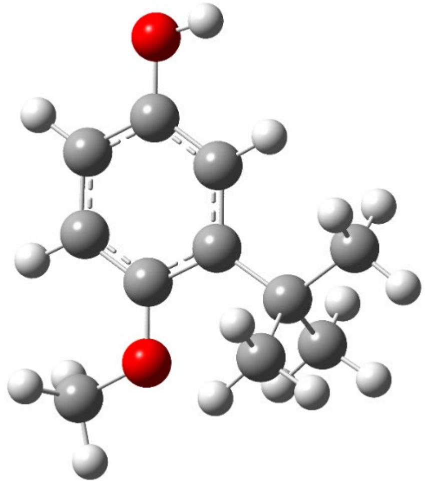

2-dmg

Charge=0, Multiplicity=2

C	1.52162500	1.48621500	0.00062300
C	0.19341100	0.99757900	0.00051500
C	-0.09054600	-0.41487300	0.00016300
C	0.99080900	-1.25937900	-0.00018500
C	2.37011800	-0.80646000	-0.00014600
C	2.58516500	0.62090600	0.00030100
H	1.70338200	2.55415400	0.00109500
H	0.86068800	-2.33542300	-0.00055700
H	3.60755500	0.98556100	0.00041100
O	3.32420600	-1.62035600	-0.00047500
O	-0.86002600	1.82277400	0.00064400
C	-0.65779900	3.23474600	-0.00098700
H	-0.11803800	3.54861100	-0.89936200
H	-1.65569900	3.67128600	-0.00149600
H	-0.11811900	3.55087100	0.89664800
C	-1.53374800	-0.93885900	0.00004000
C	-1.56600900	-2.47274400	-0.00085400
H	-1.08391300	-2.89331400	0.88782600
H	-2.60956600	-2.80251800	-0.00099800
H	-1.08397500	-2.89226600	-0.89006500
C	-2.27452100	-0.46196300	-1.26353500
H	-2.36280400	0.62478500	-1.30448300
H	-1.75526900	-0.80391100	-2.16566400
H	-3.28421500	-0.88739900	-1.27361300
C	-2.27402300	-0.46326100	1.26439500
H	-2.36181600	0.62350900	1.30663200
H	-3.28389100	-0.88828100	1.27432500
H	-1.75464800	-0.80644700	2.16596900

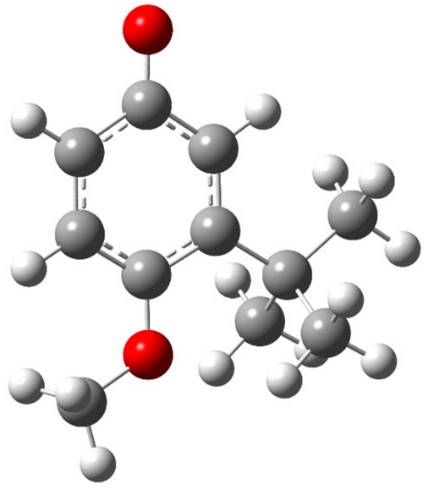

3

Charge=0, Multiplicity=1

C	0.11606600	-0.63861700	-0.15540000
C	0.47907700	0.71448600	-0.09981800
C	2.81395200	0.05507100	0.03019900
O	4.11837700	0.45596100	0.12401800
H	4.69796700	-0.31712900	0.12950900
C	-0.58030100	1.79005400	-0.14199200
H	-0.70535300	2.21998700	0.86003000
H	-0.24987300	2.60685000	-0.79161000
C	-1.90527900	1.21644300	-0.63836000
H	-1.85275200	1.03709100	-1.71913500
H	-2.72531700	1.91880600	-0.45771500
C	-2.22167400	-0.11728200	0.04343700
C	-3.49222800	-0.72929600	-0.52363000
H	-3.66024700	-1.72137800	-0.09379500
H	-4.35301800	-0.09691900	-0.28595800
H	-3.41831700	-0.82785600	-1.61081500
C	-2.31403000	0.01456500	1.56289500
H	-3.06707900	0.76170400	1.83416900
H	-2.60053700	-0.94545400	2.00255500
H	-1.35745900	0.31567400	2.00028400
O	-1.17894800	-1.06424400	-0.27534900
C	1.09931600	-1.62949500	-0.12384000

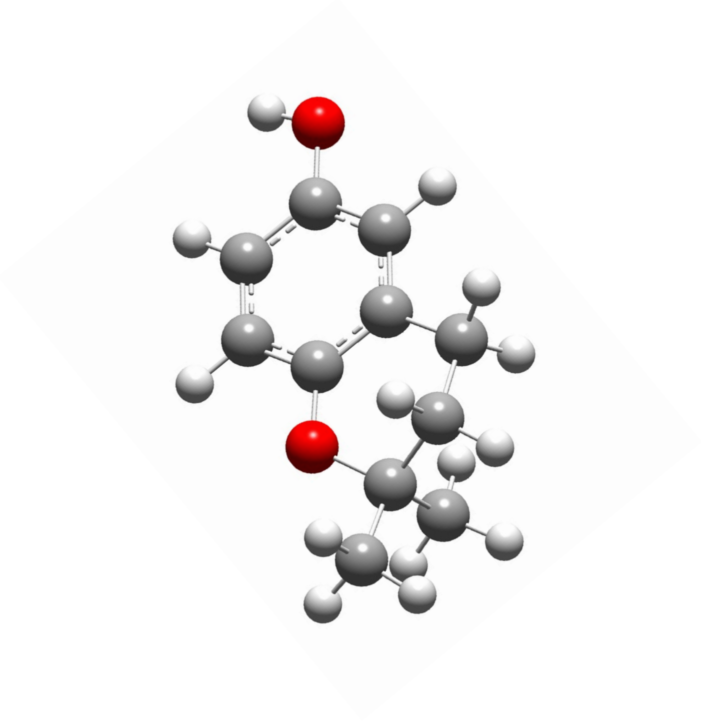

H	0.79375400	-2.67021900	-0.17207200
C	2.44467400	-1.28993600	-0.03437000
H	3.20296700	-2.06875500	-0.01133600
C	1.83453100	1.04197700	-0.00432000
H	2.13520600	2.08603900	0.03771900

3-dmg

Charge=0, Multiplicity=2

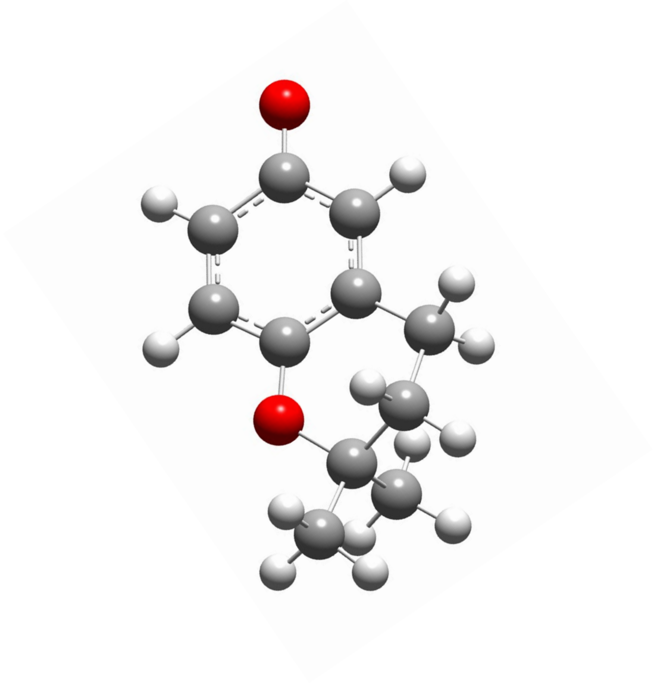

C	0.17921400	-0.62688400	-0.09774000
C	0.53431300	0.75149200	-0.06054400
C	2.91820000	0.07779100	0.02957800
O	4.12868900	0.39796200	0.09122200
C	-0.54922000	1.80146500	-0.11380500
H	-0.71824100	2.20721800	0.89072800
H	-0.21560200	2.63528200	-0.73808200
C	-1.83812100	1.20036300	-0.66712200
H	-1.72805900	1.00357300	-1.74045300
H	-2.67619400	1.89244400	-0.54143200
C	-2.18108300	-0.11670700	0.02694900
C	-3.38206700	-0.78554100	-0.61880300
H	-3.56078300	-1.76620300	-0.16851100
H	-4.27224000	-0.16740700	-0.47028000
H	-3.22000500	-0.91519100	-1.69277500
C	-2.37970300	0.04038700	1.53151400
H	-3.17500100	0.76613000	1.72778200
H	-2.66501400	-0.91879100	1.97258500
H	-1.46703500	0.38512200	2.02619400
O	-1.08380700	-1.05860700	-0.17117100
C	1.17428200	-1.64071600	-0.08865400
H	0.84414400	-2.67444700	-0.12815000
C	2.49737700	-1.31133000	-0.02422400
H	3.27023000	-2.07374200	-0.00948200
C	1.86818400	1.07520800	-0.00107900
H	2.17648200	2.11800600	0.01505100

4

Charge=0, Multiplicity=1

C	-0.09137600	0.38515400	-0.12603400
C	-0.38979100	-0.98199500	-0.11386400
C	-2.74458900	-0.44006300	0.03971900
O	-4.03325300	-0.89420600	0.12232100
H	-4.64177500	-0.14424200	0.14983800
C	0.71918200	-2.00470500	-0.19266300
H	0.86751100	-2.46094400	0.79449900
H	0.42404900	-2.81470900	-0.86734000
C	2.01493100	-1.35689300	-0.67315400
H	1.95017300	-1.14410700	-1.74719300
H	2.86650100	-2.02719700	-0.51792100
C	2.27192300	-0.03397500	0.05255600
C	3.51068600	0.65575000	-0.49545900
H	3.63206900	1.64036900	-0.03381600
H	4.40120100	0.05792700	-0.27871400
H	3.43004500	0.78576100	-1.57886800
C	2.37627800	-0.21300800	1.56663300
H	3.16171200	-0.93578100	1.81078200
H	2.62337900	0.74320300	2.03755600
H	1.43488800	-0.56912400	1.99547400
O	1.18523700	0.87301100	-0.23037400
C	-1.11145500	1.34916500	-0.06475200
C	-2.43491000	0.92172000	0.01706500
H	-3.23252100	1.66076700	0.06647500
C	-1.72713200	-1.38248500	-0.02710500
H	-1.97758400	-2.44025800	-0.01871700
C	-0.75721500	2.81037500	-0.09136600
H	-0.08869400	3.06591700	0.73698800
H	-0.23019200	3.07020400	-1.01520900
H	-1.65583800	3.42752600	-0.01886400

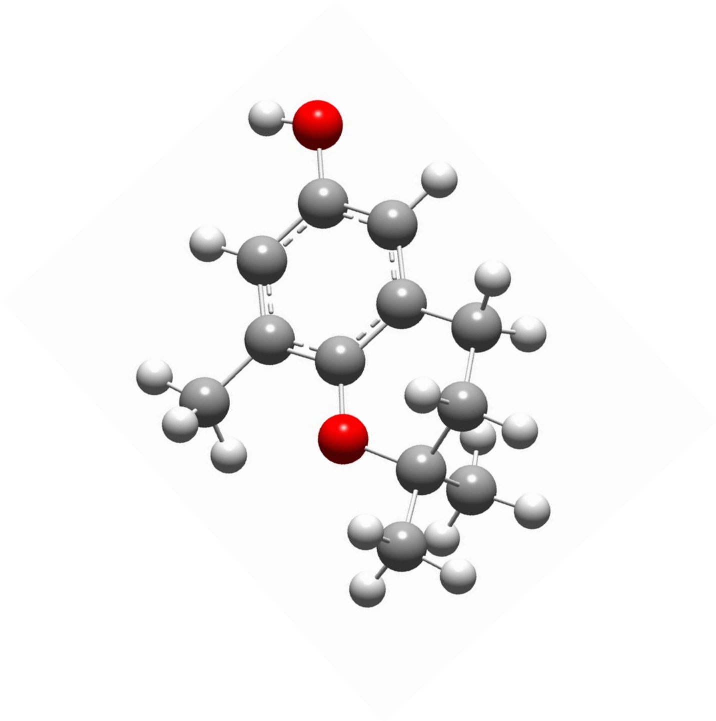

4-dmg

Charge=0, Multiplicity=2

C	-0.15121500	0.36697600	-0.07999900
C	-0.43080800	-1.02766600	-0.07510500
C	-2.83995900	-0.48817700	0.03779700
O	-4.03283500	-0.87016200	0.09650100
C	0.70751500	-2.01699400	-0.15511200
H	0.89874000	-2.43936800	0.83857300
H	0.41666200	-2.85111300	-0.80003900
C	1.96360500	-1.33821800	-0.69212000
H	1.84371200	-1.12061400	-1.76036700
H	2.83653300	-1.98872700	-0.58231000
C	2.23515800	-0.02302300	0.03527900
C	3.40018400	0.72533100	-0.58877900
H	3.52276400	1.70362000	-0.11508500
H	4.32261800	0.15393100	-0.45008000
H	3.23569000	0.87101800	-1.66034200
C	2.43808800	-0.20596900	1.53654700
H	3.26448300	-0.89989400	1.71907800
H	2.67929300	0.75479300	2.00008700
H	1.54049000	-0.60206400	2.02034300
O	1.08994400	0.86253300	-0.14351200

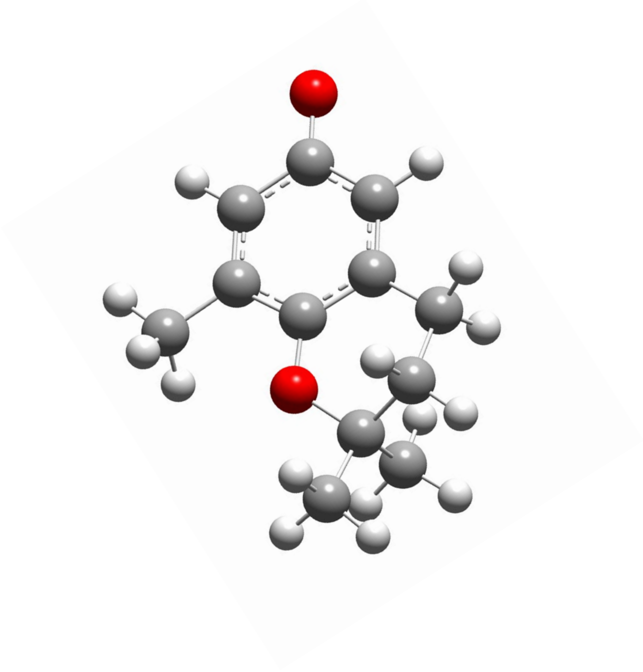

C	-1.19357600	1.34788300	-0.04674600
C	-2.49137600	0.92030000	0.01456600
H	-3.30846700	1.63616900	0.04928700
C	-1.74301500	-1.43045200	-0.01958600
H	-1.99318400	-2.48869300	-0.02744600
C	-0.82075200	2.80338800	-0.07076900
H	-0.18374600	3.05817900	0.78200600
H	-0.25754300	3.04909300	-0.97648100
H	-1.71800400	3.42442400	-0.03697200

5

Charge=0, Multiplicity=1

C	0.15035900	0.58546000	-0.13581700
C	-0.35684000	-0.71908100	-0.12626300
C	-2.58913700	0.17819100	0.04100000
O	-3.94052600	-0.04707800	0.12616800
H	-4.40343300	0.79846500	0.18681900
C	0.58672200	-1.89509400	-0.22988400
H	0.64800900	-2.40648200	0.74066200
H	0.18496800	-2.63050600	-0.93512200
C	1.97492900	-1.44953500	-0.68152600
H	1.96152800	-1.21934200	-1.75382400
H	2.70838700	-2.24673200	-0.52279500
C	2.42060800	-0.18920600	0.06019900
C	3.75880400	0.30798500	-0.46171200
H	4.02345100	1.25683100	0.01481000
H	4.54359000	-0.42227900	-0.24208700
H	3.71502700	0.46131500	-1.54419800
C	2.47114100	-0.39344400	1.57392200
H	3.14099700	-1.22307100	1.82281700
H	2.84395400	0.51366300	2.05912200
H	1.48131500	-0.61554000	1.98355500
O	1.48824500	0.87048500	-0.23524900
C	-0.70141500	1.69874100	-0.07156700
C	-2.07191600	1.47439000	0.01154000
H	-2.74943900	2.32552000	0.05875800
C	-1.74451600	-0.93101800	-0.02028400
C	-2.30809800	-2.32793800	0.01269300
H	-2.25645700	-2.80157300	-0.97524200
H	-1.74957700	-2.96319400	0.70732700
H	-3.35361600	-2.31754800	0.32255400
C	-0.12567400	3.08779200	-0.09597100
H	0.57345000	3.23764500	0.73306100
H	0.43521000	3.26638800	-1.01921600
H	-0.91891300	3.83572700	-0.02234900

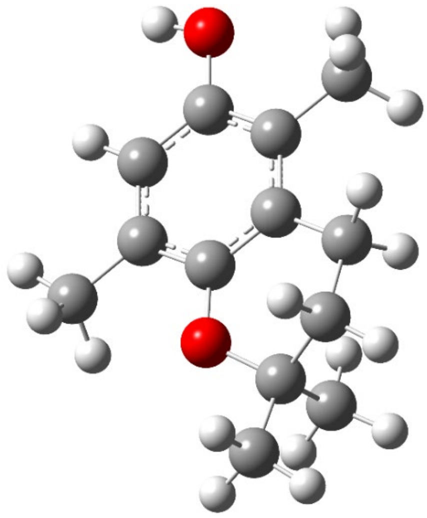

5-dmg

Charge=0, Multiplicity=2

C	0.10243100	0.57996200	-0.10113100
C	-0.40808400	-0.74304700	-0.10169700
C	-2.68407100	0.19581100	0.05204200
O	-3.92647600	0.04115100	0.12973800
C	0.54705500	-1.90768600	-0.22338400
H	0.63645900	-2.41764100	0.74473700
H	0.13551700	-2.64377700	-0.92111800
C	1.91592300	-1.44212700	-0.70983800
H	1.86669200	-1.18642800	-1.77521600
H	2.66031000	-2.23579300	-0.59410900
C	2.38309200	-0.20564300	0.05216600
C	3.68225700	0.33998600	-0.51430000
H	3.95339500	1.27493000	-0.01530800
H	4.48691200	-0.38419100	-0.35695800
H	3.58558400	0.52875900	-1.58741700
C	2.49397300	-0.44446400	1.55546800
H	3.19110000	-1.26517300	1.75141300
H	2.86732500	0.45652500	2.05052800
H	1.52608300	-0.70057300	1.99618400
O	1.41243800	0.85982100	-0.15506000
C	-0.75207400	1.72688800	-0.06324600
C	-2.10049700	1.52149700	0.01097400
H	-2.78785500	2.36247800	0.04950100
C	-1.77456400	-0.94443600	-0.00953300
C	-2.35899100	-2.32484900	0.00607000
H	-2.30137300	-2.78656700	-0.98787500
H	-1.81989000	-2.97843500	0.69923900
H	-3.40925400	-2.28230600	0.29616400
C	-0.13834300	3.09815300	-0.09384200
H	0.53634900	3.24492200	0.75536300
H	0.45417100	3.24329200	-1.00261900
H	-0.91785800	3.86194500	-0.05841300

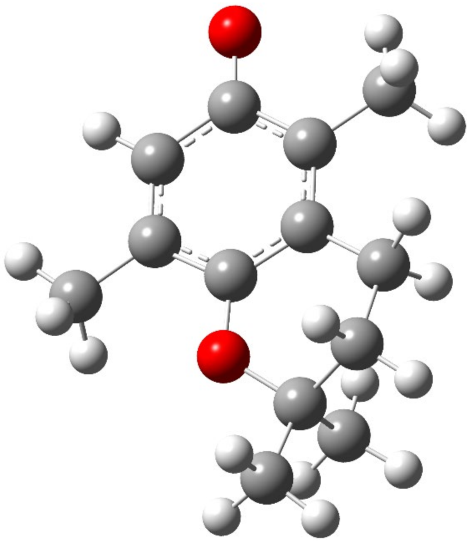

6

Charge=0, Multiplicity=1

C	0.13836000	0.26540500	-0.13357200
C	0.03192800	-1.12846300	-0.11345000
C	-2.37843700	-0.89908500	0.02926700
O	-3.63453600	-1.44490100	0.11494500
H	-3.56643900	-2.40841500	0.10615600
C	1.26452800	-1.99965900	-0.18114500
H	1.46737200	-2.42651600	0.80968400
H	1.08146100	-2.84539100	-0.85208000
C	2.46774400	-1.19240500	-0.65969900
H	2.38483800	-1.00007800	-1.73631200
H	3.39925000	-1.74228100	-0.49069300
C	2.53798800	0.15974000	0.05366500
C	3.67920000	1.00312700	-0.49160100
H	3.66668300	1.99852000	-0.03723100
H	4.63922500	0.52929800	-0.26517200
H	3.58944500	1.11380600	-1.57644100
C	2.65129100	0.01152900	1.57048600
H	3.52467900	-0.59613900	1.82913300

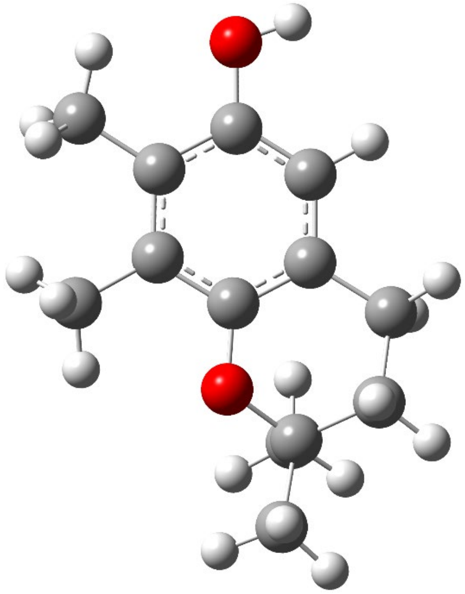

H	2.76289800	0.99711700	2.03240800
H	1.76258200	-0.46374400	1.99606600
O	1.34446500	0.90993200	-0.25143900
C	-1.00531700	1.08280700	-0.07245000
C	-2.27751000	0.49921600	-0.00455300
C	-1.24189300	-1.69555200	-0.02509200
H	-1.33908500	-2.78049900	-0.00733300
C	-0.85907200	2.58222200	-0.09752700
H	-1.55672700	3.05716600	0.59794900
H	0.15582300	2.87787200	0.16929700
H	-1.07041100	2.98260700	-1.09694600
C	-3.51867500	1.35290000	0.03635000
H	-4.41121100	0.75060500	-0.13600000
H	-3.63015400	1.84622200	1.00948600
H	-3.48046700	2.13891700	-0.72409300

6-dmg

Charge=0, Multiplicity=2

C	0.08344300	0.23335500	-0.09249900
C	0.01256500	-1.18642600	-0.08327000
C	-2.44797400	-0.98690900	0.03041100
O	-3.56465900	-1.55516400	0.09301100
C	1.27858000	-2.00588400	-0.16077400
H	1.52314300	-2.40103100	0.83243100
H	1.11079400	-2.86999600	-0.81015800
C	2.42944900	-1.15464500	-0.68736700
H	2.28956900	-0.95409700	-1.75656500
H	3.38551800	-1.67349400	-0.56924300
C	2.49987200	0.18477500	0.04322700
C	3.55804200	1.09100300	-0.56161200
H	3.53289400	2.07609700	-0.08675900
H	4.54964500	0.65560100	-0.40722000
H	3.39230800	1.21362500	-1.63589200
C	2.70343000	0.02869700	1.54772700
H	3.61797700	-0.53955600	1.74412300
H	2.79684100	1.01299400	2.01536500
H	1.86428500	-0.49350400	2.01648000
O	1.24534800	0.89896200	-0.15636400
C	-1.08237100	1.05764900	-0.06548300
C	-2.32855400	0.47351800	0.00092200
C	-1.23000400	-1.76282400	-0.02508200
H	-1.33658600	-2.84494200	-0.02830300
C	-0.87615300	2.54852100	-0.10155400
H	-0.28936200	2.87930700	0.76165200
H	-0.31572500	2.84016000	-0.99544000
H	-1.82489700	3.08435000	-0.09968500
C	-3.59571000	1.27967000	0.04719200
H	-3.60564900	1.95711900	0.90759300
H	-3.70982100	1.89218600	-0.85381200
H	-4.45412400	0.61180600	0.12122400

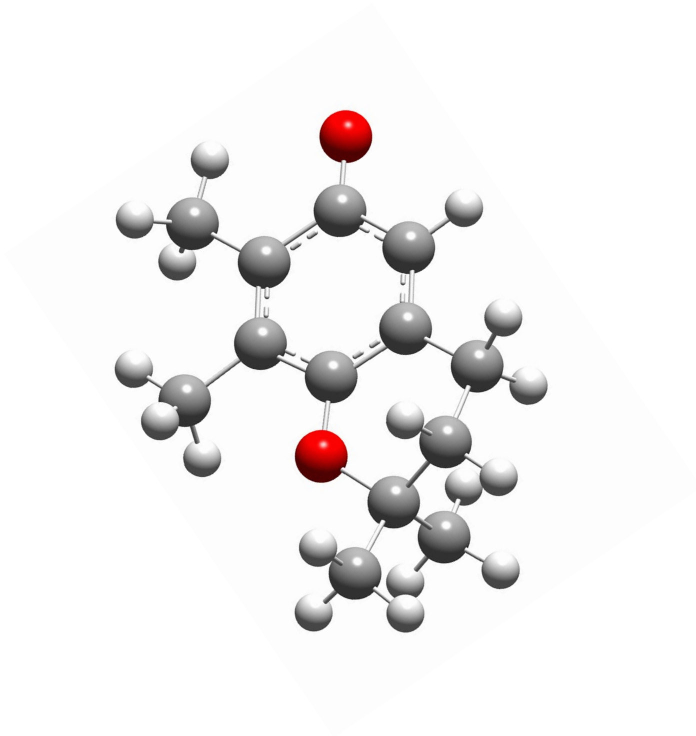

7

Charge=0, Multiplicity=1

C	0.27821400	0.46695500	-0.15013900
C	0.03797800	-0.91244700	-0.13517800
C	-2.32245700	-0.45808100	0.03117300
O	-3.59862700	-0.95904600	0.11754900
H	-4.23223500	-0.23853900	0.22037300
C	1.18660000	-1.89067600	-0.23160800
H	1.33455600	-2.38270900	0.73974600
H	0.93455600	-2.68808100	-0.93899700
C	2.47195800	-1.19658800	-0.67074100
H	2.43126400	-0.97952800	-1.74503900
H	3.34032200	-1.83982200	-0.49439200
C	2.65633700	0.12999400	0.06517300
C	3.88352400	0.87050500	-0.44157300
H	3.95538800	1.85509500	0.03059400
H	4.78948900	0.30439900	-0.20434700
H	3.82852100	1.00669800	-1.52582500
C	2.72159000	-0.04973100	1.58154800
H	3.53578000	-0.73174800	1.84781300
H	2.90410000	0.91590400	2.06277000
H	1.78753800	-0.45645200	1.98001700
O	1.54540100	0.98851600	-0.25866500
C	-0.77371100	1.39713200	-0.08597700
C	-2.09102500	0.92467400	-0.00972000
C	-1.28009500	-1.38342200	-0.02375500
C	-1.56692900	-2.86226300	0.02057600
H	-1.44831500	-3.32021500	-0.96915600
H	-0.88152700	-3.37721400	0.70076900
H	-2.58772600	-3.05103300	0.35391400
C	-0.47434000	2.87662100	-0.10620100
H	0.59983500	3.05181800	-0.06121900
H	-0.85419800	3.34786200	-1.01986900
H	-0.93955200	3.38660000	0.74336700
C	-3.27600400	1.85948600	0.03525800
H	-3.80844900	1.79532100	0.99354800
H	-2.97873000	2.89950600	-0.09535700
H	-3.99465100	1.63343200	-0.76280200

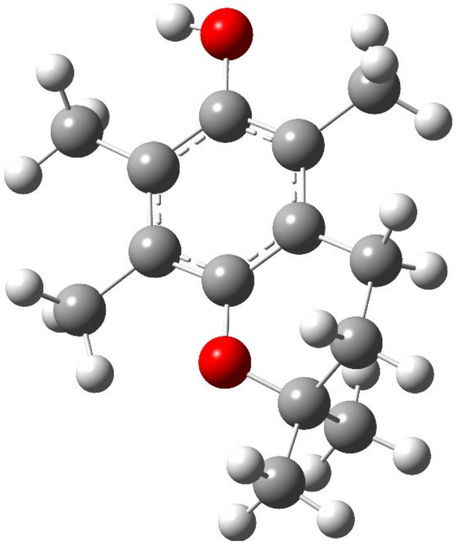

7-dmg

Charge=0, Multiplicity=2

C	-0.22391700	-0.44140400	-0.10668300
C	-0.00096400	0.95825700	-0.10159500
C	2.41786000	0.49405000	0.03983300
O	3.59602400	0.91982900	0.11125200
C	-1.17327100	1.90463800	-0.21607300
H	-1.36222300	2.38284700	0.75406200
H	-0.92288900	2.71178100	-0.91174400
C	-2.41944900	1.17110800	-0.70118300
H	-2.32252400	0.93617000	-1.76807900
H	-3.31082000	1.79403100	-0.57893800
C	-2.61868300	-0.13904700	0.05472800
C	-3.78069000	-0.93666800	-0.51140200
H	-3.85125200	-1.91052200	-0.01821100
H	-4.71676700	-0.39502100	-0.34662000
H	-3.65196900	-1.09531500	-1.58608800
C	-2.77215400	0.06396600	1.55965500
H	-3.62175600	0.72335500	1.76294300
H	-2.95112600	-0.89746600	2.04953400
H	-1.87568900	0.50996600	2.00026500
O	-1.45230200	-0.98143800	-0.16153400
C	0.84066800	-1.39152400	-0.07597900
C	2.13901500	-0.94269400	-0.00412000
C	1.29587000	1.42480300	-0.01098000
C	1.59226900	2.89515200	0.01174300
H	1.46940300	3.33533900	-0.98623700
H	0.91516200	3.42760200	0.68692000
H	2.62170700	3.06555500	0.32740100
C	0.47327900	-2.85108000	-0.11078600
H	-0.14745400	-3.11481100	0.75184400
H	-0.11407500	-3.08297400	-1.00494300
H	1.35842500	-3.48665200	-0.10544500
C	3.31187300	-1.88232100	0.04189100
H	3.25699800	-2.54454000	0.91266200
H	3.34746600	-2.51707000	-0.85004700
H	4.23936700	-1.31280500	0.09867700

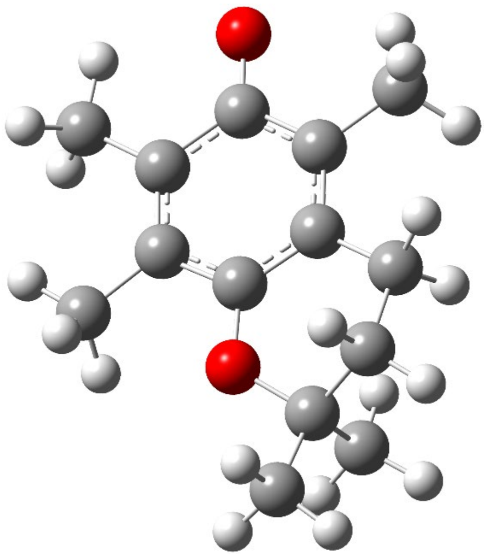

8

Charge=0, Multiplicity=1

C	1.41653300	-0.01925500	-0.00255000
C	0.72555100	1.19076200	0.00010200
C	-0.66862300	1.20224500	0.00748600
C	-1.39732600	0.00804700	0.00866200
C	-0.68842000	-1.20131300	0.00773600
C	0.70243500	-1.21684300	0.00014000
H	1.27285300	2.13074900	-0.00337000
H	-1.19705700	2.15208500	0.01378900
H	-1.23575900	-2.14049300	0.01428300
H	1.24479900	-2.15738900	-0.00272000
O	2.78419400	-0.09286700	-0.01029200
H	3.15932600	0.79751200	-0.00519400
N	-2.79978700	0.01888600	0.07845500
H	-3.22386800	0.85575800	-0.30420300
H	-3.23623900	-0.80934800	-0.30888700

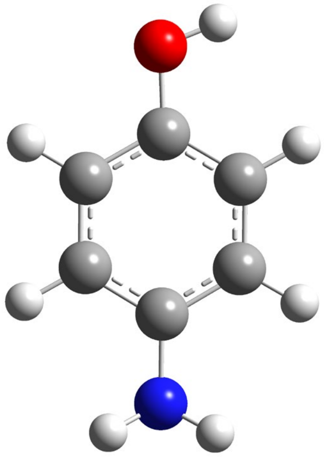

8-dmg

Charge=0, Multiplicity=2

C	-1.52061000	0.00000100	-0.00002400
C	-0.75250300	1.23147600	0.00001800
C	0.61438700	1.22819900	0.00007400
C	1.33656600	0.00000900	0.00000900
C	0.61439300	-1.22818600	0.00007400
C	-0.75250100	-1.23147300	0.00002700
H	-1.30745700	2.16499800	0.00006300
H	1.17236600	2.16104400	0.00014800
H	1.17238000	-2.16102800	0.00013600
H	-1.30744900	-2.16499900	0.00007700
O	-2.77506900	-0.00000900	-0.00007200
N	2.68793600	-0.00000500	-0.00016100
H	3.20833800	-0.86556800	0.00006300
H	3.20843400	0.86550100	0.00014000

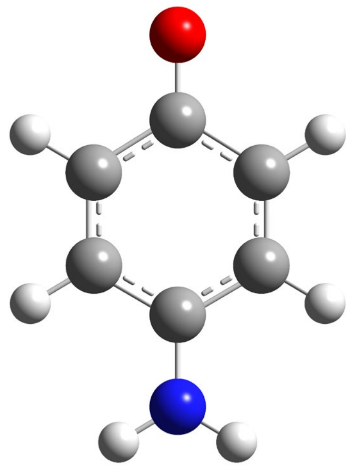

9

Charge=0, Multiplicity=1

C	-2.19461400	-0.04252300	-0.03664500
C	-1.53849300	1.16732300	0.20026800
C	-0.15285800	1.20398300	0.29956200
C	0.62388300	0.04149100	0.15311100
C	-0.05454200	-1.16315800	-0.08743400
C	-1.44653400	-1.20576100	-0.17357000
H	-2.11186300	2.08377100	0.32180000
H	0.32819800	2.15193700	0.51804600
H	0.49329800	-2.08978200	-0.21176800
H	-1.95641300	-2.14674100	-0.35641700
N	2.03080600	0.11392500	0.29058800
O	-3.55497900	-0.13710700	-0.13794800
H	-3.95430200	0.73380700	-0.01212700
C	2.68563100	1.12279000	-0.53745900
H	3.73240500	1.20552700	-0.23397000
H	2.65000000	0.85764500	-1.60761400
H	2.22242900	2.10077200	-0.40811300
C	2.74273600	-1.15040800	0.24029400
H	3.79403300	-0.96571400	0.47366200
H	2.34486100	-1.84075600	0.98823600
H	2.69028800	-1.63350800	-0.75101800

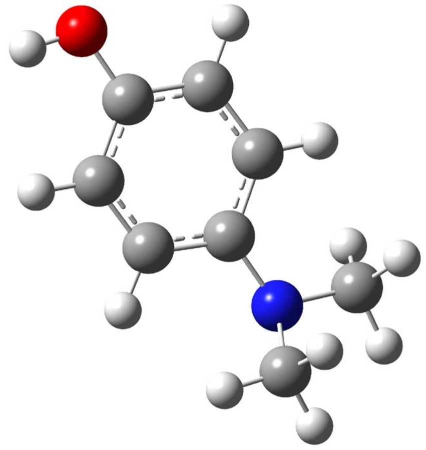

9-dmg

Charge=0, Multiplicity=2

C	-2.30563400	0.00000100	-0.00001900
C	-1.53322400	1.22609100	-0.01589900
C	-0.16579900	1.22598900	-0.01539500
C	0.57359400	0.00002200	0.00011400
C	-0.16579000	-1.22595300	0.01548400
C	-1.53321400	-1.22608100	0.01584600
H	-2.08397000	2.16226600	-0.03101800
H	0.36236900	2.17207000	-0.03327400
H	0.36240400	-2.17202300	0.03332600
H	-2.08394900	-2.16226500	0.03080000
N	1.92854400	0.00000000	0.00026700
O	-3.56056500	-0.00000100	-0.00002600
C	2.66732600	1.25529200	0.02910600
H	3.73223200	1.03619300	0.07929100
H	2.47431200	1.84705700	-0.87242900
H	2.39422200	1.84841300	0.90793100
C	2.66720400	-1.25534000	-0.02930200
H	3.73214700	-1.03630400	-0.07895900
H	2.47385200	-1.84775300	0.87172400
H	2.39431600	-1.84777700	-0.90866800

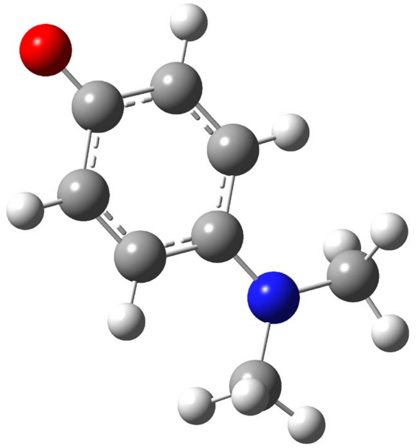

10

Charge=0, Multiplicity=1

C	1.75808300	-0.65210200	-0.04463200
C	0.56564000	-1.38439400	0.02148500
C	-0.67095000	-0.70389900	0.05576900
C	-0.71287400	0.70012800	0.04615700
C	0.48429700	1.43487100	0.07002800
C	1.69778500	0.74877600	0.01996800
C	0.46958800	2.94191100	0.11721500
H	0.25755200	3.37199300	-0.86968800
H	-0.29787900	3.30903600	0.80477000
H	1.43600200	3.32774200	0.44370900
C	0.56061400	-2.89264000	0.05994500
H	-0.25517100	-3.24597500	0.69659600
H	0.40888800	-3.33019400	-0.93601000
H	1.49252100	-3.29557000	0.45891200
C	3.10923500	-1.31617300	-0.17418800
H	3.64080200	-1.36883500	0.78519100
H	3.02518300	-2.33267100	-0.55835600
H	3.74890300	-0.77547800	-0.88140800
O	2.84379900	1.50930600	0.00735800
H	3.61449400	0.94877300	0.15754700
C	-2.03789600	1.43431500	-0.02493300
H	-2.29250200	1.84521700	0.96247700
H	-1.93988100	2.29297500	-0.69766100
C	-3.16703400	0.52275800	-0.49769100
H	-3.04327700	0.28962200	-1.56289500
H	-4.13547700	1.01782400	-0.37355400
C	-3.11745700	-0.76961900	0.30762900
H	-3.92375800	-1.45336200	0.03062700
H	-3.24245300	-0.52992700	1.37277200
N	-1.85394600	-1.47680900	0.13570900
H	-1.90089700	-2.19154100	-0.58236500

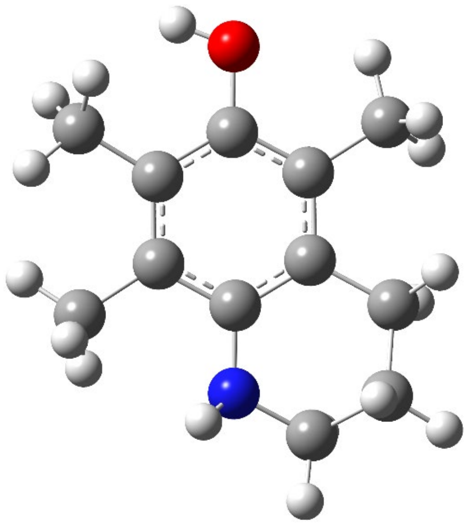

10-dmg

Charge=0, Multiplicity=2

C	-1.82826700	-0.58156600	0.02185700
C	-0.68462600	-1.34636700	0.00691500
C	0.59768800	-0.70560000	-0.04947900
C	0.71243600	0.71962300	-0.04561000
C	-0.42573500	1.49321100	-0.04343300
C	-1.74720700	0.87827700	-0.00733500
C	-0.35118100	2.99398400	-0.07164500
H	-0.01990100	3.39393600	0.89514600
H	0.35661800	3.34631000	-0.82882100
H	-1.33645100	3.40931000	-0.28495700
C	-0.72400600	-2.85325100	0.04484800
H	-0.29827100	-3.29012600	-0.86751500
H	-0.15291800	-3.24314500	0.89657500
H	-1.74311300	-3.22611900	0.13792500
C	-3.20256600	-1.19408200	0.06701900
H	-3.36828900	-1.87188200	-0.77723200
H	-3.35412100	-1.77145800	0.98595700
H	-3.95431200	-0.40569500	0.03041900
O	-2.78665400	1.58473700	0.00549300
C	2.08969500	1.35092400	-0.05139300
H	2.34599300	1.66316400	-1.07378900
H	2.07640100	2.26037400	0.55650200
C	3.15923600	0.39291000	0.46929000
H	3.01768800	0.22063200	1.54267100
H	4.15677600	0.81680300	0.32554000
C	3.05647900	-0.93472300	-0.26328300
H	3.76530300	-1.66640300	0.13090800
H	3.27352200	-0.79066000	-1.33154500
N	1.71588700	-1.47549600	-0.09382900
H	1.60541900	-2.47449100	-0.19143800

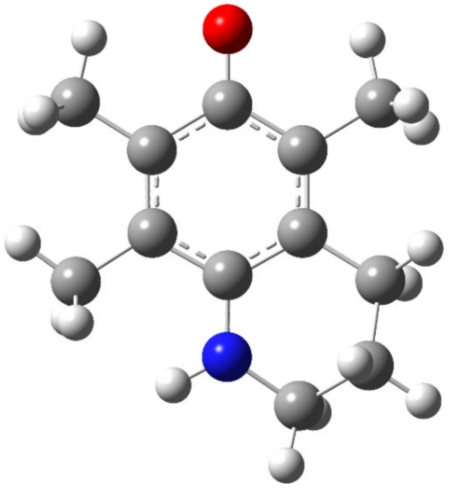

11

Charge=0, Multiplicity=1

C	0.25807600	-2.46644800	-0.41823900
H	-0.38669200	-3.32831100	-0.22223100
H	0.60193900	-2.54116500	-1.45999000
C	1.47363900	-2.45432300	0.50901800
H	2.04888600	-3.38139900	0.42980600
H	1.12657800	-2.36649700	1.54477400
C	2.38011300	-1.27901200	0.16074300
H	3.01435200	-1.53760900	-0.69866600
H	3.05347500	-1.07548800	1.00686600
C	2.42048100	1.15484800	0.04581400
H	2.65720500	1.28309800	1.11789900
H	3.37410900	1.03096200	-0.48148700
C	1.68578800	2.38560700	-0.46171600
H	1.52274900	2.29250400	-1.54209200
H	2.30274700	3.27273500	-0.29085600
C	0.34523700	2.50694300	0.25832300
H	-0.24406700	3.33690700	-0.14448700
H	0.52468500	2.72968300	1.31882200
N	1.64157500	-0.05504200	-0.16034000
O	-3.93205400	0.14567900	0.08439100

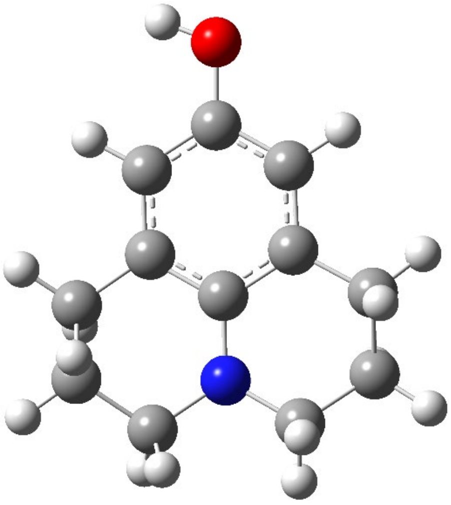

H	-4.31693100	-0.73112500	-0.04268800
C	-1.89427000	-1.14958800	-0.15296100
H	-2.45528200	-2.07699200	-0.25959700
C	-1.83063400	1.23333500	0.15628900
H	-2.35787700	2.17563700	0.28332200
C	-0.43578600	1.21930400	0.12763900
C	0.24718700	-0.00168400	-0.05568700
C	-0.50258400	-1.18453800	-0.21695100
C	-2.56398900	0.05837700	0.02903800

11-dmg

Charge=0, Multiplicity=2

C	0.25491100	-2.51088700	0.31656000
H	0.49273000	-2.66191900	1.37845000
H	-0.35700900	-3.35903300	-0.00224600
C	1.55330800	-2.42799100	-0.47876400
H	1.32955900	-2.32301900	-1.54671600
H	2.15648900	-3.33067900	-0.34948300
C	2.35499100	-1.22944800	-0.00327500
H	3.21805600	-1.05465000	-0.65504400
H	2.73809100	-1.40254100	1.01283400
C	2.35509000	1.22942300	0.00335900
H	3.21799500	1.05457200	0.65533500
H	2.73843600	1.40236600	-1.01267300
C	1.55325000	2.42810000	0.47877000
H	2.15637400	3.33083200	0.34954900
H	1.32935600	2.32309500	1.54668400
C	0.25483200	2.51092400	-0.31668500
H	0.49265300	2.66187200	-1.37857900
H	-0.35713800	3.35905700	0.00209200
N	1.56119400	0.00002100	0.00002400
O	-3.91920400	-0.00004800	0.00010100
C	-1.89327900	-1.21840100	0.13441000
H	-2.44616500	-2.14942600	0.23695600
C	-1.89334600	1.21835100	-0.13448500
H	-2.44625800	2.14935300	-0.23709100
C	-0.52524900	1.23149800	-0.14926100
C	0.20171900	0.00001700	-0.00001800
C	-0.52521000	-1.23149500	0.14918400
C	-2.66400000	-0.00003000	0.00002900

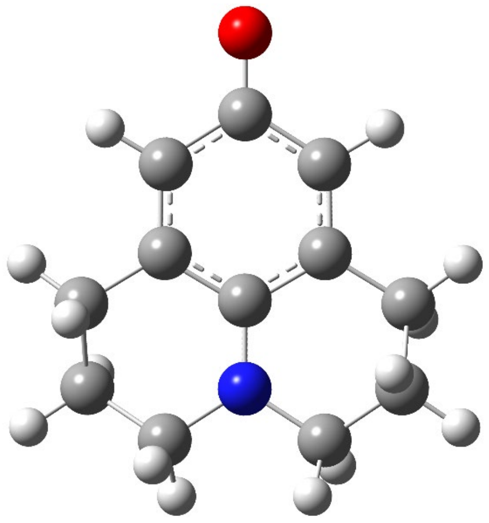

12

Charge=0, Multiplicity=1

C	-2.32913000	-1.13841400	-0.03753100
C	-2.78557300	0.17952800	-0.01685100
C	-1.86931300	1.23454200	0.03587100
C	-0.50636100	0.96727500	0.06735000
C	-0.02123900	-0.34971500	0.03935100
C	-0.96289900	-1.38992500	-0.00811400
H	-3.04942500	-1.94938800	-0.07587300
H	-2.22478300	2.26200300	0.05952400
H	0.18578800	1.80247800	0.11807200
H	-0.61342600	-2.41919800	-0.02527900
O	-4.13053900	0.38178800	-0.04687900
H	-4.32477100	1.32880500	-0.02206800
C	1.40841000	-0.68188100	0.05849900
H	1.63455000	-1.74633100	0.13466100
C	2.44203100	0.17644700	-0.01967100
H	2.27078000	1.24875800	-0.11218700
C	3.83245900	-0.25831400	0.00998700
H	4.00379100	-1.33133300	0.09925900
C	4.87893900	0.57568300	-0.06380500
H	4.73806000	1.65075400	-0.15292700
H	5.89979900	0.20779300	-0.03866200

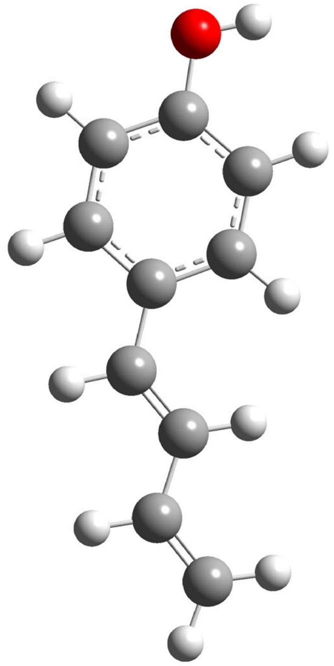

12-dmg

Charge=0, Multiplicity=2

C	1.87085900	-1.29181100	0.00012200
C	2.87860500	-0.23603600	0.00000700
C	2.38481700	1.13471000	-0.00000500
C	1.04671000	1.39882200	0.00002400
C	0.07438200	0.35124200	0.00004200
C	0.54013400	-1.00301200	0.00004900
H	2.22833500	-2.31717300	0.00018100
H	3.12272400	1.93125800	-0.00003300
H	0.69391200	2.42713700	0.00003800
H	-0.18112100	-1.81416000	0.00008200
O	4.09602300	-0.49548800	-0.00009000
C	-1.31458700	0.70176200	0.00005300
H	-1.53713600	1.76859300	0.00040300
C	-2.36950400	-0.16347700	-0.00024900
H	-2.20644100	-1.23991000	-0.00066400
C	-3.74333000	0.28394000	-0.00008600
H	-3.91451700	1.35962800	-0.00008100
C	-4.78861600	-0.56183100	0.00010900
H	-4.63978000	-1.63904600	0.00008500
H	-5.81098900	-0.19827900	0.00031000

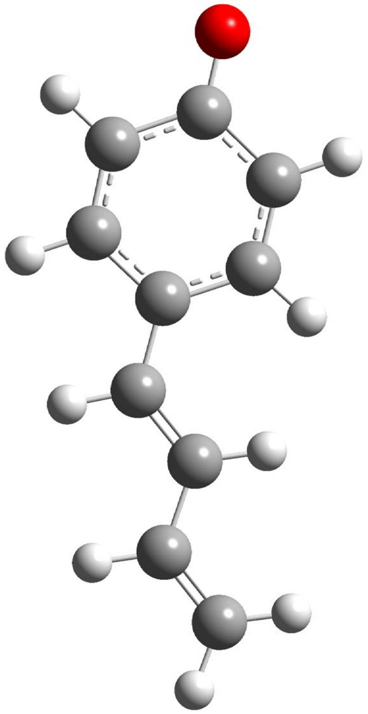

13

Charge=0, Multiplicity=1

C	-2.33266000	1.33424200	0.00029100
C	-1.45870600	0.23852000	0.00006300
C	-2.02745500	-1.04792800	-0.00018300
C	-3.40201700	-1.22895100	-0.00022000
C	-4.25217600	-0.11831200	-0.00002000
C	-3.71522300	1.16835200	0.00024900
H	-1.92190400	2.34088700	0.00048700
H	-1.39009500	-1.92696100	-0.00031000
H	-3.83496200	-2.22430000	-0.00039100
H	-4.37258600	2.03471100	0.00044500
C	-0.01041200	0.48580500	0.00008500
H	0.26409800	1.53975900	0.00039600
C	0.95901100	-0.44474900	-0.00020300
H	0.68633100	-1.49900200	-0.00041500
C	2.40861500	-0.19289900	-0.00010700
C	2.97114000	1.09520300	-0.00039900
C	3.27959100	-1.29346600	0.00021300
C	4.35092900	1.26877100	-0.00024400
H	2.32983700	1.97144100	-0.00076800
C	4.66223400	-1.12050500	0.00034000
H	2.86185200	-2.29740500	0.00038100
C	5.20510500	0.16299300	0.00013300
H	4.76414400	2.27322800	-0.00046700
H	5.31398900	-1.98925900	0.00058700
H	6.28163000	0.30375100	0.00022400
O	-5.59163400	-0.35618600	-0.00004600
H	-6.07710900	0.48017800	0.00020500

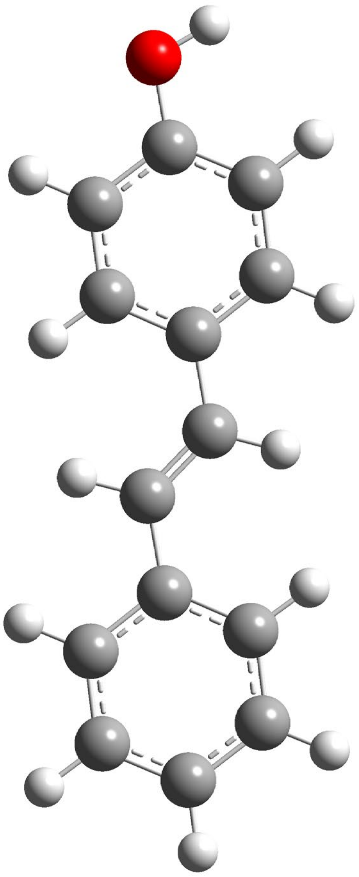

13-dmg

Charge=0, Multiplicity=2

C	2.39306000	1.35979500	-0.00001000
C	1.50155900	0.24341800	-0.00001500
C	2.06770300	-1.07157700	-0.00003100
C	3.41700800	-1.25903800	-0.00002900
C	4.34289200	-0.13165400	0.00000900
C	3.74793500	1.19735100	-0.00000800
H	1.96441000	2.35908000	-0.00001200
H	1.41342700	-1.93769400	-0.00005000
H	3.84962200	-2.25508300	-0.00004000
H	4.42349300	2.04744700	-0.00000300
O	5.57690800	-0.29956200	0.00005700
C	0.08872600	0.49847400	-0.00001100
H	-0.18927100	1.55017900	-0.00001100
C	-0.89198800	-0.44485500	-0.00000600
H	-0.61702600	-1.49722600	0.00000100
C	-2.32980000	-0.19267900	0.00000400
C	-2.88949000	1.09911000	-0.00000100
C	-3.19954200	-1.29740400	0.00001200
C	-4.26790200	1.27105100	0.00000300
H	-2.24808900	1.97487300	-0.00000200
C	-4.58023000	-1.12329100	0.00001200
H	-2.77994600	-2.30032700	0.00001700
C	-5.11962900	0.16254100	0.00000800
H	-4.68348600	2.27413200	0.00000100
H	-5.23416000	-1.98995900	0.00001500
H	-6.19605500	0.30361100	0.00000800

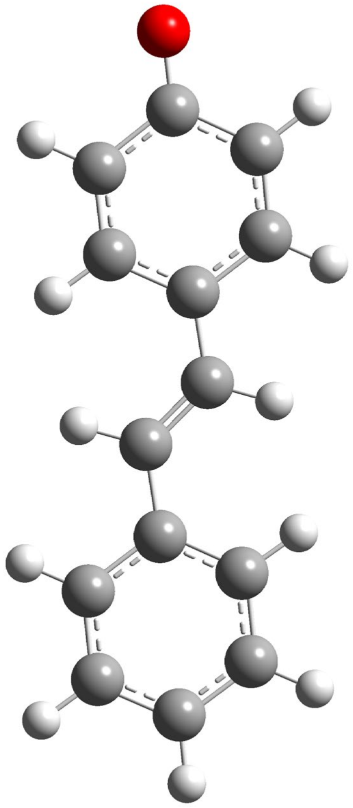

14

Charge=0, Multiplicity=1

C	0.54750700	-0.93511100	0.00007000
C	1.87499600	-1.34978300	0.00000800
C	2.90229900	-0.40526100	-0.00002800
C	2.60159300	0.95778300	-0.00004100
C	1.28236500	1.38546900	0.00002600
C	0.26030000	0.43122000	0.00009500
H	-0.24193400	-1.67744300	0.00009500
H	1.03739400	2.44319900	0.00001700
O	2.17038700	-2.67409600	-0.00002700
H	3.13258900	-2.78764100	-0.00001300
O	4.18054200	-0.86645200	-0.00005900
H	4.79690000	-0.11833700	0.00002400
O	3.69064300	1.77955800	-0.00009200
H	3.41965000	2.70807600	0.00010000
C	-1.14237400	0.93288300	0.00008600
O	-1.44081900	2.11013000	-0.00004700
O	-2.04641600	-0.05156900	0.00019100
C	-3.42788400	0.35168300	0.00004000
H	-3.61580200	0.96559600	-0.88730600
H	-3.61598500	0.96571600	0.88726300
C	-4.27752700	-0.90193000	0.00005300
H	-4.03003400	-1.50168300	0.88286100
H	-4.02977100	-1.50189600	-0.88253700
C	-5.76303600	-0.54973100	-0.00021000

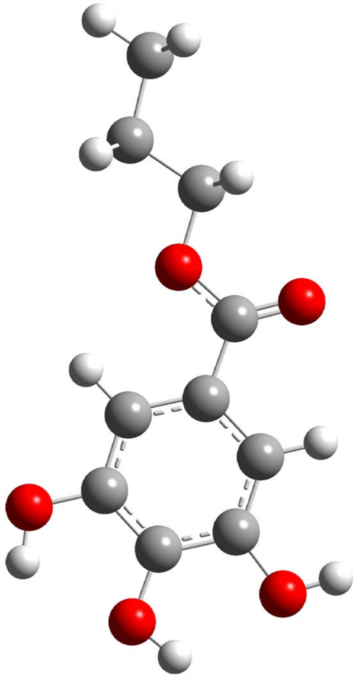

H	-6.02959100	0.03689100	-0.88568800
H	-6.37773500	-1.45365200	-0.00009200
H	-6.02982200	0.03726700	0.88495000

14$^{(1)}$-dmg

Charge=0, Multiplicity=2

C	0.59638700	0.88647300	-0.00002500
C	1.90595900	1.33284000	-0.00003700
C	2.93618900	0.39072700	-0.00002900
C	2.67090200	-1.03875700	-0.00000700
C	1.29139100	-1.45522700	0.00000700
C	0.30056000	-0.50766400	-0.00000300
H	-0.20681400	1.61472700	-0.00003400
H	1.06153300	-2.51511800	0.00002700
O	2.17141000	2.66192200	-0.00005300
H	3.12919000	2.80780300	-0.00008500
O	4.20074800	0.80436100	-0.00003900
H	4.76924200	0.00829800	-0.00005600
O	3.65772900	-1.81175000	-0.00000500
C	-1.11927100	-0.98237200	0.00002300
O	-1.43683100	-2.15162300	0.00009700
O	-1.99416400	0.02439300	-0.00004800
C	-3.38901100	-0.34068000	-0.00001100
H	-3.59064500	-0.94918600	0.88773400
H	-3.59068500	-0.94919300	-0.88774200
C	-4.20263800	0.93614000	0.00000100
H	-3.93908300	1.52861100	-0.88303800
H	-3.93896300	1.52866700	0.88296600
C	-5.69706500	0.62398200	0.00011300
H	-5.97938600	0.04495900	0.88559000
H	-6.28647700	1.54449100	0.00003000
H	-5.97947500	0.04474600	-0.88519800

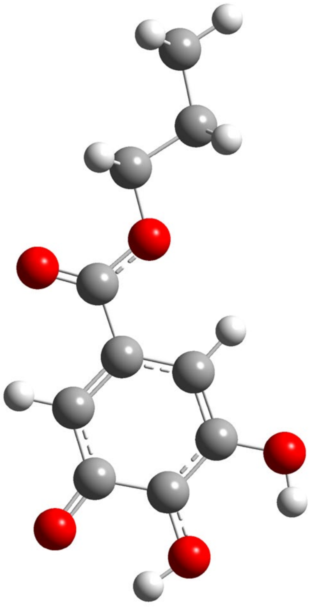

14$^{(2)}$-dmg

Charge=0, Multiplicity=2

C	0.56852900	0.95338900	-0.00002700
C	1.87854400	1.38076900	-0.00005700
C	2.97663200	0.42731200	-0.00005400
C	2.64164900	-0.98783800	-0.00001800
C	1.32868600	-1.40109100	0.00001300
C	0.31191900	-0.42941700	0.00000700
H	-0.24146300	1.67241400	-0.00003100
H	1.07472100	-2.45487100	0.00004100
O	2.20359700	2.67443700	-0.00008900
H	3.17646100	2.73949200	-0.00010600
O	4.16751700	0.81163700	-0.00008200
O	3.66146500	-1.84793900	-0.00001600
H	4.48884100	-1.33231700	-0.00004200
C	-1.09724900	-0.93816200	0.00004000
O	-1.38094600	-2.11687000	0.00007000
O	-1.99851300	0.04309500	0.00003000
C	-3.38269500	-0.36081700	0.00005700
H	-3.56714700	-0.97466100	0.88792300
H	-3.56717100	-0.97469900	-0.88777700
C	-4.23082000	0.89329700	0.00004200

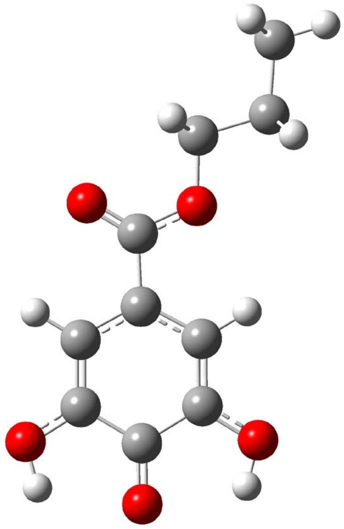

H	-3.98311800	1.49259700	-0.88294300
H	-3.98309800	1.49263200	0.88299800
C	-5.71631500	0.54092500	0.00006600
H	-5.98309000	-0.04550300	0.88548900
H	-6.33007100	1.44537500	0.00005600
H	-5.98311000	-0.04553600	-0.88532800

14[(3)]-dmg

Charge=0, Multiplicity=2

C	0.54151900	0.97651400	-0.00005800
C	1.91155900	1.42703400	-0.00008700
C	2.95142300	0.41090200	-0.00004200
C	2.65113500	-0.95311200	0.00002000
C	1.32331100	-1.33792900	0.00004300
C	0.27922900	-0.36912200	0.00000500
H	-0.24613200	1.72101700	-0.00008800
H	1.06904400	-2.39269700	0.00009100
O	2.27309700	2.62722900	-0.00014600
O	4.22344600	0.80061300	-0.00006200
H	4.22997700	1.77865300	-0.00010700
O	3.63324700	-1.88642200	0.00005600
H	4.50044000	-1.45453200	0.00003400
C	-1.12061200	-0.89785000	0.00003800
O	-1.38714300	-2.08077900	0.00008400
O	-2.03639900	0.06970200	0.00001200
C	-3.41428600	-0.35452800	0.00004200
H	-3.59065700	-0.97069300	0.88791000
H	-3.59068100	-0.97073400	-0.88779300
C	-4.28082400	0.88702900	0.00002400
H	-4.04189000	1.48999700	-0.88287800
H	-4.04186900	1.49003500	0.88289500
C	-5.76104900	0.51310000	0.00005000
H	-6.01929200	-0.07721700	0.88544700
H	-6.38803500	1.40845600	0.00004100
H	-6.01931400	-0.07725200	-0.88531600

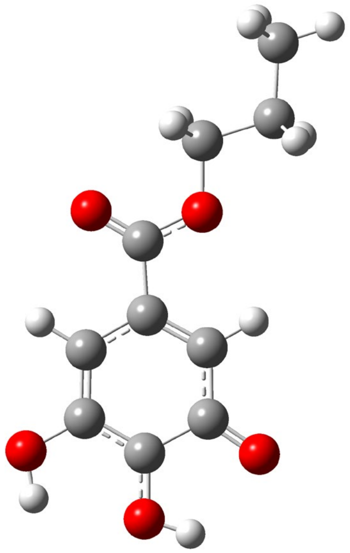

15

Charge=0, Multiplicity=1

C	-4.09277600	-0.96982600	-0.40112800
C	-5.40994800	-0.65773000	-0.08632900
C	-5.81147300	0.67595200	0.04957600
C	-4.87903700	1.68896800	-0.13617300
C	-3.55795400	1.37170800	-0.45314200
C	-3.14515700	0.04313200	-0.58916400
H	-3.80187500	-2.01455600	-0.50013100
H	-5.20158200	2.72070500	-0.03450900
H	-2.83865800	2.17368800	-0.60112500
O	-7.10230800	0.98105300	0.35608700
H	-7.60519000	0.15810400	0.44364500
O	-6.39663700	-1.58522900	0.11210900
H	-6.05217100	-2.48080800	-0.00503100
C	-1.70492200	-0.28445200	-0.90639900
H	-1.62847700	-1.33384900	-1.22040800
H	-1.39030700	0.32415800	-1.75976600
C	-0.73963600	-0.06457500	0.27974100
H	-0.91156800	0.94959300	0.67389900
C	0.72981600	-0.12926900	-0.19962200
H	0.83405500	-1.02554100	-0.83231800
C	1.71733300	-0.27813900	0.97782200
H	1.48970500	-1.19542500	1.52939400
H	1.56706200	0.56024100	1.67186800
C	3.16531800	-0.32590800	0.54911400
C	3.68336100	-1.46199200	-0.07865300
C	4.01162100	0.77253800	0.73931100
C	5.00886600	-1.50656100	-0.51257100
H	3.04509500	-2.32905400	-0.22984100
C	5.33269300	0.73110600	0.31041900
H	3.63850200	1.67249800	1.22557400
C	5.83980300	-0.40988300	-0.32183200
H	5.41301900	-2.39008500	-0.99724400
O	6.22523300	1.75752700	0.46117200
H	5.80663900	2.51302800	0.89530100
O	7.13446000	-0.44975000	-0.74094500
H	7.55853700	0.39355900	-0.52470300
C	-1.06383000	-1.06768800	1.38914700
H	-0.52480900	-0.85094500	2.31487900
H	-2.13282600	-1.04806800	1.62319200
H	-0.80703100	-2.08855100	1.07633100
C	1.09518100	1.10306300	-1.03239700
H	2.09415100	1.00720000	-1.46746100
H	1.09025400	2.00058400	-0.39997000
H	0.39593000	1.27205100	-1.85530100

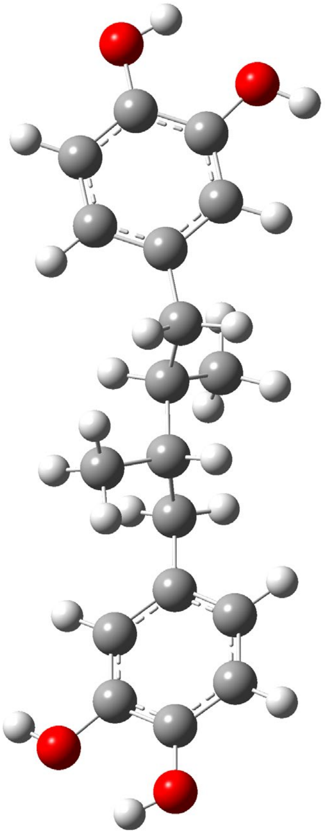

15^(1)-dmg

Charge=0, Multiplicity=2

C	-4.04241300	-1.00592800	-0.35499900
C	-5.36044000	-0.69092400	-0.04519100
C	-5.75715400	0.64944000	0.02811600
C	-4.83618600	1.66018800	-0.21275500
C	-3.51485600	1.33472500	-0.52337900
C	-3.10181300	0.00137500	-0.59655600
H	-3.75875200	-2.05411500	-0.40494100
H	-5.15356400	2.69895000	-0.16177800
H	-2.80066100	2.13194000	-0.71443700
O	-7.07502400	0.85262800	0.34086900
H	-7.27865000	1.79715300	0.36524300
O	-6.25794300	-1.68652100	0.18543200
H	-7.12032800	-1.29104700	0.38057300
C	-1.66140600	-0.33361400	-0.90594600
H	-1.58366500	-1.39162500	-1.18835300
H	-1.34820900	0.25065300	-1.77670500
C	-0.69850500	-0.07673300	0.27464300
H	-0.88588200	0.94106700	0.65165800
C	0.77104000	-0.12291000	-0.20643100
H	0.88675900	-1.02443700	-0.82924000
C	1.75540500	-0.23750200	0.97786100
H	1.55942300	-1.16357800	1.52867300
H	1.58020300	0.59723900	1.66857300
C	3.20257400	-0.23748400	0.55564600
C	3.70323600	-1.35992100	-0.18345700
C	4.04195200	0.80725000	0.83973900
C	5.01150700	-1.43336600	-0.63074200
H	3.02729400	-2.18518600	-0.39223000
C	5.41547100	0.79602300	0.40613200
H	3.69923800	1.67587200	1.39550500
C	5.86975400	-0.37369900	-0.34787200
H	5.37535600	-2.29177800	-1.18587600
O	6.24872400	1.70734000	0.62151900
O	7.13844100	-0.37142500	-0.74115500
H	7.52357500	0.47008500	-0.42193400
C	-1.00270400	-1.06681600	1.40101500
H	-0.46634800	-0.82528600	2.32228800
H	-2.07143100	-1.06274300	1.63627900
H	-0.73004800	-2.08819900	1.10410800
C	1.11771200	1.10752100	-1.05004100
H	2.10912700	1.01684400	-1.50366600
H	1.12122500	2.00706700	-0.42112700
H	0.40269100	1.26919600	-1.86063400

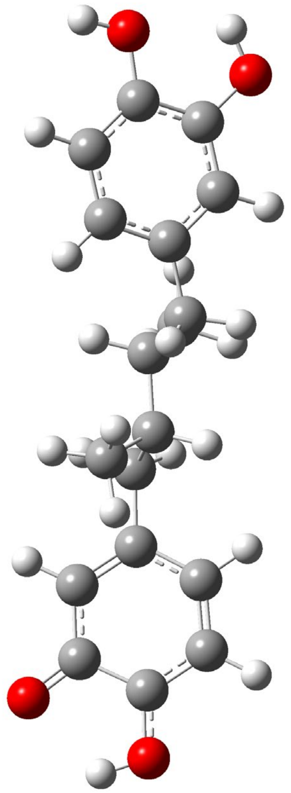

15(2)-dmg

Charge=0, Multiplicity=2

C	-4.03120300	-0.99809800	-0.35496900
C	-5.34889500	-0.68440400	-0.04265200
C	-5.74613100	0.65569700	0.03386900
C	-4.82602400	1.66735300	-0.20633100
C	-3.50490000	1.34318800	-0.51941300
C	-3.09147100	0.01021100	-0.59588500
H	-3.74710600	-2.04603100	-0.40769400
H	-5.14394700	2.70582600	-0.15306800
H	-2.79148700	2.14113700	-0.71027700
O	-7.06361500	0.85757100	0.34873400
H	-7.26859000	1.80183400	0.37204500
O	-6.24556100	-1.68085900	0.18705500
H	-7.10831500	-1.28635600	0.38256000
C	-1.65163000	-0.32429600	-0.90823100
H	-1.57506900	-1.38073800	-1.19657500
H	-1.33830800	0.26467700	-1.77575100
C	-0.68723100	-0.07537100	0.27298900
H	-0.86725900	0.94318900	0.65137100
C	0.78054200	-0.13215600	-0.21140000
H	0.89012100	-1.03380200	-0.83423700
C	1.76714800	-0.25700900	0.97322800
H	1.56188200	-1.18350800	1.52032900
H	1.60261600	0.57940400	1.66403800
C	3.20585500	-0.28016900	0.54096000
C	3.69469000	-1.41452500	-0.18113700
C	4.05668200	0.79064900	0.81070800
C	4.98559700	-1.48347900	-0.62567200
H	3.01171600	-2.23917900	-0.36963600
C	5.37217300	0.74565200	0.37166300
H	3.70264800	1.66030100	1.35680900
C	5.89856000	-0.39751000	-0.37253000
H	5.36138600	-2.34391400	-1.17061200
O	6.22173700	1.74331700	0.60422100
H	7.07313000	1.48037400	0.20076200
O	7.09862800	-0.36141700	-0.73634700
C	-0.99609800	-1.06529400	1.39803600
H	-0.45637300	-0.82871500	2.31871600
H	-2.06417900	-1.05419900	1.63558900
H	-0.73108300	-2.08800000	1.09889300
C	1.13833400	1.09652400	-1.05294300
H	2.13096700	0.99898300	-1.50314300
H	1.14389500	1.99627800	-0.42444800
H	0.42786400	1.26178300	-1.86671300

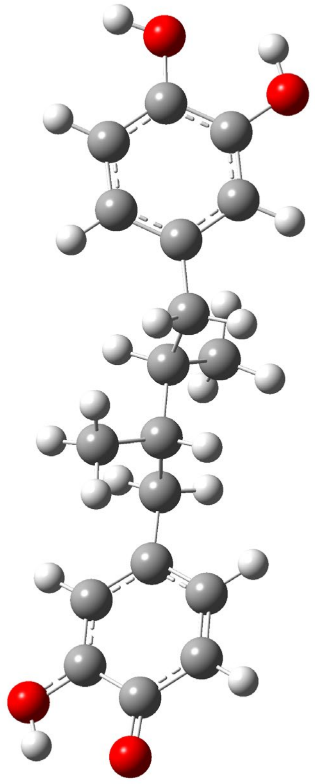

15(3)-dmg

Charge=0, Multiplicity=2

C	-4.12758300	-1.00013100	-0.48681100
C	-5.44216700	-0.70687700	-0.15260900
C	-5.88287700	0.66117900	0.11564500
C	-4.88622000	1.69677000	0.01182700
C	-3.59833100	1.38077000	-0.31982400
C	-3.19450500	0.03186700	-0.57352100
H	-3.83834700	-2.03024800	-0.67421900
H	-5.19791500	2.71931800	0.20046200
H	-2.85119900	2.16629000	-0.40358800
O	-7.08769800	0.84425300	0.41329600
O	-6.36836900	-1.65773800	-0.05728100
H	-7.20156000	-1.20770900	0.18776600
C	-1.75758200	-0.24511700	-0.91377600
H	-1.65265600	-1.28220500	-1.25483000
H	-1.47956300	0.40506400	-1.74970200
C	-0.79096100	-0.01507400	0.27295600
H	-0.94835500	1.00766300	0.64952400
C	0.67792700	-0.11057500	-0.20193600
H	0.78870400	-1.06107600	-0.74833400
C	1.65727500	-0.14567700	0.99196000
H	1.42075500	-1.00126600	1.63127800
H	1.50938400	0.76007600	1.59567200
C	3.10766800	-0.24875800	0.58092200
C	3.61906500	-1.45671900	0.09934400
C	3.95703000	0.86137800	0.64421300
C	4.94824400	-1.55802000	-0.31591900
H	2.97802900	-2.33333300	0.05046400
C	5.28200600	0.76904900	0.23466900
H	3.59449600	1.81746000	1.01304700
C	5.77798300	-0.44713000	-0.25041000
H	5.34273200	-2.50131700	-0.68578800
O	6.09059700	1.86012400	0.30681000
H	6.97316900	1.61682300	-0.00951300
O	7.09439000	-0.43261000	-0.62815900
H	7.37068800	-1.30571700	-0.93722400
C	-1.12612500	-0.99854200	1.39729700
H	-0.59064400	-0.76594100	2.32067800
H	-2.19541100	-0.97529700	1.62961600
H	-0.87020700	-2.02494500	1.10321600
C	1.04321600	1.04075500	-1.14286700
H	2.07164600	0.94441400	-1.50190100
H	0.96248900	2.00119900	-0.61718000
H	0.39803000	1.08362800	-2.02372900

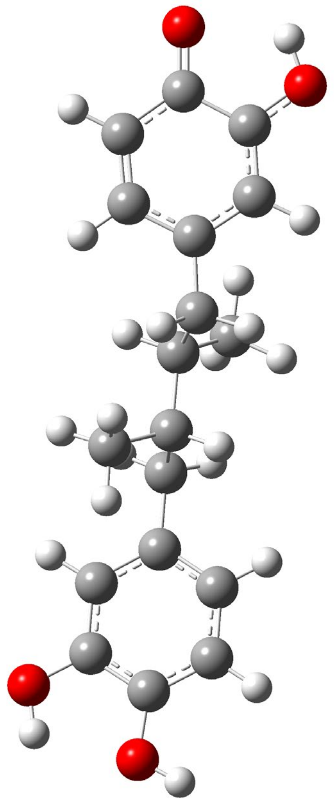

15[(4)]-dmg

Charge=0, Multiplicity=2

C	-4.15513700	-1.04644900	-0.41103800
C	-5.50993200	-0.66850900	-0.09875700
C	-5.79157200	0.76177600	0.03393600
C	-4.79396400	1.71810800	-0.13703600
C	-3.51272700	1.28712200	-0.43504400
C	-3.17769900	-0.10015100	-0.57483400
H	-3.94044300	-2.10710400	-0.51041500
H	-5.03210800	2.77210800	-0.03925900
H	-2.72597000	2.02421000	-0.57655100
O	-7.04420600	1.09748100	0.32104200
H	-7.54530500	0.25924000	0.38777500
O	-6.46251600	-1.46630500	0.06796800
C	-1.74734800	-0.46377100	-0.88023500
H	-1.69213800	-1.51911000	-1.17371200
H	-1.42003900	0.12668300	-1.74241600
C	-0.79162300	-0.23598900	0.31360500
H	-1.00412300	0.75628900	0.74255400
C	0.67679100	-0.21765700	-0.17200100
H	0.81374000	-1.07550100	-0.85007400
C	1.67468700	-0.38462500	0.99370300
H	1.48480700	-1.33508800	1.50165900
H	1.49779700	0.41465900	1.72619300
C	3.12021600	-0.35550600	0.55486500
C	3.68302000	-1.45573000	-0.09736100
C	3.91210100	0.77962000	0.76001500
C	5.00634100	-1.42583500	-0.54217700
H	3.08745800	-2.35063100	-0.25900100
C	5.23018600	0.81781600	0.32126000
H	3.50924800	1.65369100	1.26509000
C	5.77784800	-0.29037600	-0.33616800
H	5.44168100	-2.28647400	-1.04452600
O	5.98183500	1.93132700	0.53271100
H	6.86896900	1.78281300	0.17343900
O	7.08251900	-0.15072100	-0.72915600
H	7.39738900	-0.95625800	-1.16059200
C	-1.07326100	-1.28951100	1.38712800
H	-0.55561500	-1.07316800	2.32503900
H	-2.14435100	-1.33654200	1.60879800
H	-0.75942600	-2.28410400	1.04492600
C	0.98248500	1.07137800	-0.94040400
H	1.97371400	1.03661900	-1.40111100
H	0.96249900	1.93064900	-0.25754200
H	0.25868100	1.26250600	-1.73752200

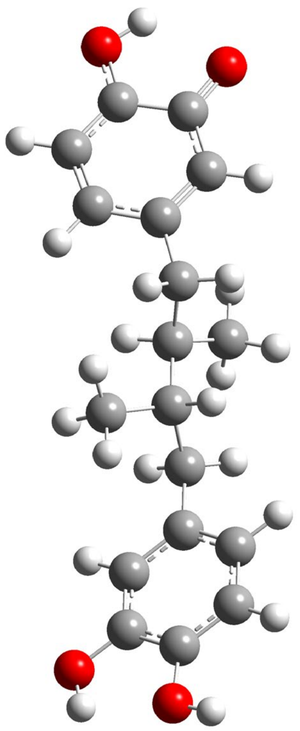

16

Charge=0, Multiplicity=1

C	-0.27924500	-0.84404800	-0.00090800
C	-0.44300900	0.54748500	0.02811600
C	-1.80847800	1.19156300	0.00195100
H	-2.04976800	1.60276500	0.99086900
H	-1.80208800	2.03772500	-0.69200600
C	-2.86162300	0.16252500	-0.40452400
H	-2.80754700	-0.03559700	-1.48137300
H	-3.86967400	0.52346300	-0.18035300
O	-1.34085000	-1.69499500	-0.01381000
C	0.98245500	-1.44421900	-0.03746500
H	1.07371000	-2.52406200	-0.06110600
C	2.11017800	-0.63445400	-0.03172200
C	0.72318200	1.32145700	0.04581400
C	-2.60736100	-1.13265400	0.34300700
H	-3.34079200	-1.90315200	0.09938600
H	-2.62127000	-0.96075300	1.42868200
C	1.99741900	0.75752500	0.01579000
H	2.88338500	1.38827000	0.02687300
O	0.54467400	2.67224900	0.08828700
H	1.40154100	3.11925500	0.07197200
O	3.32370300	-1.25281400	-0.06389900
H	4.03117600	-0.59451000	-0.04791500

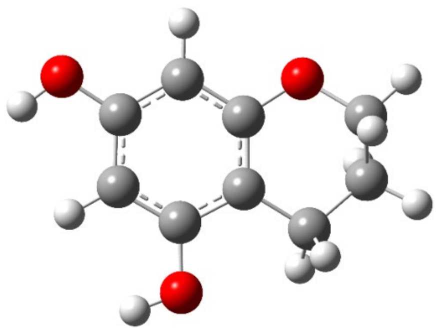

16^(1)-dmg

Charge=0, Multiplicity=2

C	-0.26295200	-0.88214900	-0.00960700
C	-0.36714300	0.53234800	0.00817200
C	-1.69807600	1.22273800	-0.02496900
H	-1.88709800	1.66931100	0.96170900
H	-1.65322100	2.05979700	-0.72908400
C	-2.81328100	0.24413200	-0.38663800
H	-2.80651500	0.03714800	-1.46255400
H	-3.79051800	0.66308600	-0.13207600
O	-1.36764000	-1.67179400	-0.02681900
C	0.96385100	-1.50693500	-0.04169400
H	1.03275800	-2.58918600	-0.06650900
C	2.18512500	-0.73187200	-0.03438100
C	0.83616200	1.29847800	0.04513400
C	-2.60046200	-1.05503500	0.36431300
H	-3.37133500	-1.79373000	0.14139900
H	-2.57837100	-0.88054500	1.44899800
C	2.07187300	0.71234900	0.02035000
H	2.98609700	1.29843900	0.03988200
O	0.64760000	2.64299400	0.09605900
H	1.50306700	3.09534800	0.10254700
O	3.30435800	-1.29170000	-0.06278900

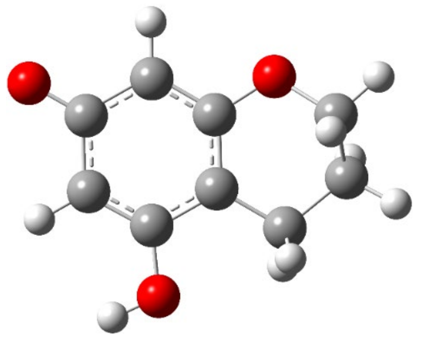

16[(2)]-dmg

Charge=0, Multiplicity=2

C	-0.27215500	-0.78762400	0.00191700
C	-0.46286900	0.58085400	0.02884200
C	-1.83551200	1.18706700	0.00212400
H	-2.07734900	1.59153300	0.99396400
H	-1.83908200	2.03997300	-0.68309700
C	-2.86394800	0.13336200	-0.40653400
H	-2.80739400	-0.05705700	-1.48436200
H	-3.87969700	0.46890000	-0.17944500
O	-1.29196400	-1.68472600	-0.00522700
C	1.02127300	-1.34775800	-0.03729400
H	1.11908900	-2.42988100	-0.06702200
C	2.16478700	-0.51876200	-0.03034700
C	0.71568400	1.44495300	0.05214700
C	-2.58221000	-1.15935700	0.33695900
H	-3.28885900	-1.94947400	0.07984400
H	-2.61067000	-0.99482100	1.42271000
C	2.03393600	0.84560700	0.02253500
H	2.90117500	1.49637900	0.03774400
O	0.57986100	2.68742300	0.09024400
O	3.41241700	-1.05464200	-0.06544200
H	3.36635900	-2.02005300	-0.09904800

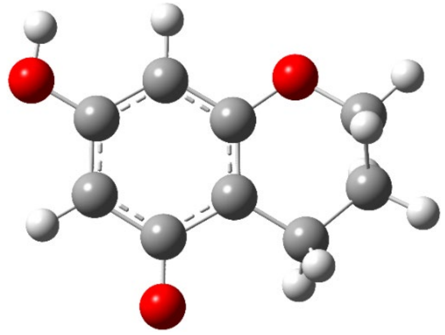

17

Charge=0, Multiplicity=1

C	-0.14287700	2.07204900	0.00006600
C	-1.30229200	1.29958300	0.00011400
C	-1.21465000	-0.09358600	0.00002000
C	0.03689800	-0.71390400	0.00008100
C	1.18824000	0.07640500	-0.00007200
C	1.11212600	1.46650400	-0.00006200
H	-0.21854600	3.15430600	0.00011200
H	-2.28161800	1.77033300	0.00018900
H	2.02290400	2.05860600	-0.00012500
O	-2.30172700	-0.91053900	-0.00023700
H	-3.10673700	-0.37534000	-0.00007800
O	2.35887600	-0.63060300	-0.00003500
H	3.11818000	-0.03245200	-0.00036200
O	0.11214400	-2.07056400	0.00023400
H	1.04680300	-2.32410600	-0.00031300

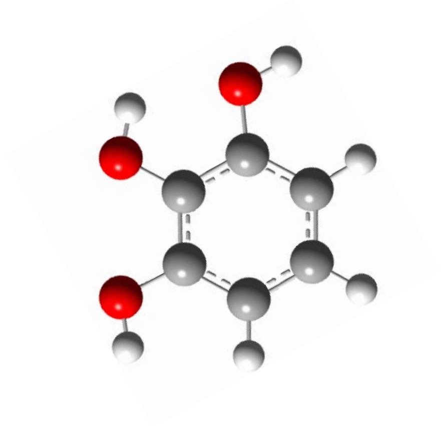

17⁽¹⁾-dmg

Charge=0, Multiplicity=2

C	0.00000000	-2.03866400	-0.00001000
C	1.23478100	-1.37057500	-0.00000400
C	1.24206300	0.00912800	0.00000000
C	0.00000000	0.75988700	-0.00000700
C	-1.24206300	0.00912800	-0.00000400
C	-1.23478100	-1.37057500	-0.00000800
H	0.00000000	-3.12411700	-0.00001100
H	2.16926500	-1.92010600	-0.00000200
H	-2.16926500	-1.92010600	-0.00000900
O	2.37345600	0.71990100	0.00000700
H	2.12879800	1.66376100	0.00000400
O	-2.37345600	0.71990100	-0.00000100
H	-2.12879800	1.66376100	-0.00000300
O	0.00000000	2.01605400	0.00002000

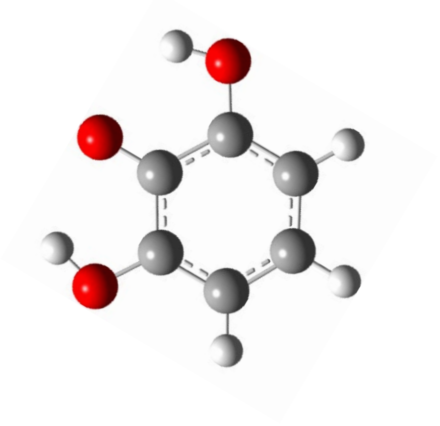

17⁽²⁾-dmg

Charge=0, Multiplicity=2

C	0.35024400	2.04277100	-0.00001600
C	-0.97251700	1.51387300	-0.00008100
C	-1.19279400	0.14659100	-0.00007200
C	-0.08753900	-0.70582600	0.00001500
C	1.27787700	-0.20057300	0.00012100
C	1.45151400	1.23229300	0.00006600
H	0.46927700	3.12130800	-0.00004300
H	-1.82866000	2.18064300	-0.00015000
H	2.46278700	1.62405800	0.00009900
O	-2.45699000	-0.34587200	-0.00014100
H	-2.43218600	-1.31422400	-0.00017100
O	2.20336400	-1.04618100	0.00012400
O	-0.27643300	-2.02598200	0.00001500
H	0.60854200	-2.44229100	0.00008900

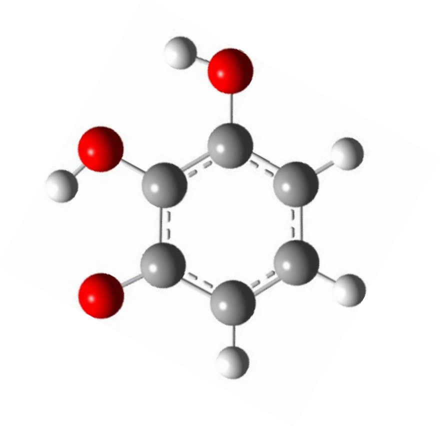

18

Charge=0, Multiplicity=1

C	0.18148200	-1.31340400	-0.00001400
C	1.55699500	-1.13590400	-0.00003700
C	2.10349000	0.14849200	-0.00008700
C	1.26840400	1.26641100	0.00002600
C	-0.11263300	1.10342200	0.00005700
C	-0.64684400	-0.18654500	0.00008800
H	-0.25364100	-2.30816000	-0.00016900
H	-0.75183800	1.97824800	0.00038300
O	1.80315900	2.51333200	0.00011000
H	2.76979200	2.44724400	0.00025900
O	3.44458300	0.36562800	-0.00015900
H	3.91223800	-0.48342400	0.00043700
O	2.47697100	-2.14341800	-0.00016700
H	2.04080200	-3.00679500	0.00135200
C	-2.11759200	-0.41747800	0.00001400
O	-2.63484600	-1.51577500	0.00012800
O	-2.82152100	0.72013000	-0.00020500
C	-4.24579900	0.57002300	-0.00003200
H	-4.57067100	0.03015300	0.89193200

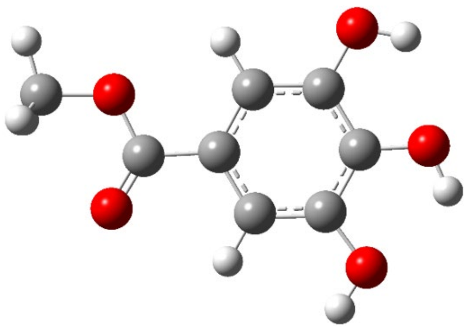

H	-4.64745500	1.58207100	0.00070300
H	-4.57100700	0.03138100	-0.89263900

18[(1)]-dmg

Charge=0, Multiplicity=2

C	0.23260600	-1.27297200	-0.00010900
C	1.60729400	-1.12619100	0.00004100
C	2.14180200	0.16438800	0.00006900
C	1.29646400	1.34715200	-0.00005800
C	-0.13104000	1.14326000	-0.00017300
C	-0.62495400	-0.13590100	-0.00021200
H	-0.20123700	-2.26742400	-0.00014000
H	-0.77697700	2.01336000	-0.00023700
O	1.86277800	2.46549000	0.00004100
O	3.46237400	0.32467500	0.00020700
H	3.64104500	1.28636500	0.00019300
O	2.41046400	-2.21684100	0.00014800
H	3.34006900	-1.94411000	0.00024800
C	-2.09533900	-0.40852100	-0.00038800
O	-2.57118800	-1.52325100	-0.00017200
O	-2.82567900	0.70712800	0.00002800
C	-4.24779600	0.52004100	0.00029900
H	-4.55618700	-0.02762500	0.89312600
H	-4.67435600	1.52155200	0.00059700
H	-4.55657100	-0.02726100	-0.89261900

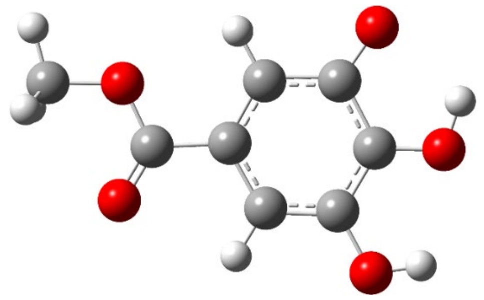

18[(2)]-dmg

Charge=0, Multiplicity=2

C	-0.21730100	-1.33720900	0.00034800
C	-1.58451100	-1.17770500	0.00003000
C	-2.17997200	0.14895600	-0.00020000
C	-1.28221600	1.29216900	-0.00012200
C	0.08505300	1.11870000	0.00021200
C	0.59768900	-0.19072600	0.00045100
H	0.22955400	-2.32464700	0.00051800
H	0.74549900	1.97697200	0.00030300
O	-1.84594100	2.50069500	-0.00037500
H	-2.81351200	2.38060800	-0.00057300
O	-3.42194700	0.30169200	-0.00058000
O	-2.42373300	-2.21477800	-0.00007400
H	-3.33370000	-1.86449500	-0.00028900
C	2.07671800	-0.42263000	0.00081600
O	2.58288400	-1.52370200	0.00006700
O	2.77548000	0.71336100	0.00030000
C	4.20234000	0.56553000	-0.00037700
H	4.52468300	0.02582900	-0.89310300
H	4.60114400	1.57830600	-0.00111400
H	4.52560100	0.02678200	0.89260000

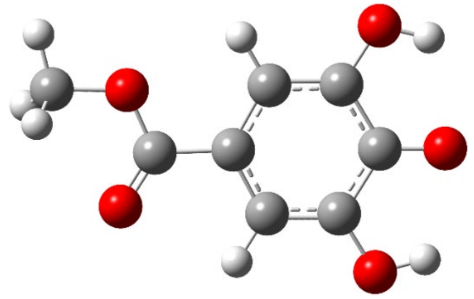

18^(3)-dmg

Charge=0, Multiplicity=2

C	0.16103500	-1.37469100	-0.00007900
C	1.59591100	-1.24179500	-0.00006800
C	2.14139600	0.10600000	0.00000400
C	1.32030500	1.23497500	-0.00001200
C	-0.05192400	1.05881100	0.00003500
C	-0.62002800	-0.24803000	-0.00003700
H	-0.27495900	-2.36769100	-0.00020100
H	-0.69405300	1.93213100	0.00018500
O	1.84650600	2.48393100	0.00011600
H	2.81409600	2.43499500	-0.00016800
O	3.46269500	0.25845400	0.00005300
H	3.86051500	-0.63506500	0.00000700
O	2.40849200	-2.19611000	-0.00004000
C	-2.10528700	-0.42749400	-0.00004400
O	-2.65544600	-1.50642600	0.00028700
O	-2.76008000	0.73601300	-0.00037600
C	-4.19161800	0.64510900	0.00008500
H	-4.53551400	0.11756900	0.89194700
H	-4.54991500	1.67290300	0.00211500
H	-4.53624900	0.12094400	-0.89350800

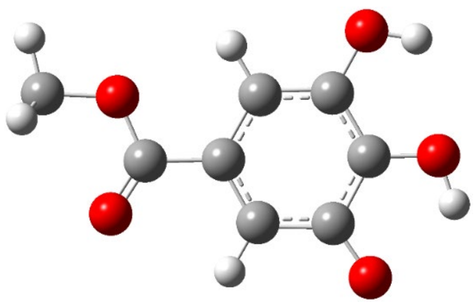

19

Charge=0, Multiplicity=1

C	-4.94665500	-0.13862800	0.00002600
C	-4.09699900	-1.24976200	-0.00034600
C	-2.72255100	-1.06912600	-0.00036600
C	-2.15384800	0.21735600	-0.00000900
C	-3.02698900	1.31363900	0.00036700
C	-4.40952100	1.14804700	0.00038700
H	-4.53032700	-2.24493500	-0.00062400
H	-2.08533600	-1.94822300	-0.00065900
H	-2.61559600	2.31999300	0.00064600
H	-5.06680100	2.01444400	0.00068600
O	-6.28595600	-0.37611200	0.00000700
H	-6.77135700	0.46031200	0.00031600
C	-0.70559400	0.46350800	0.00000300
H	-0.42879800	1.51682100	0.00016500
C	0.26166800	-0.46874600	-0.00013800
H	-0.00966200	-1.52279700	-0.00025700
C	1.71152500	-0.21474800	-0.00009600
C	2.25744300	1.07765300	-0.00021200
C	2.56665900	-1.32398700	0.00007400
C	3.63830400	1.23966600	-0.00012400
H	1.63375500	1.96450600	-0.00037200
C	3.94819300	-1.14074700	0.00016100
H	2.16751600	-2.33341700	0.00015200
C	4.50008700	0.13893200	0.00007600
H	5.57845200	0.28002200	0.00016400
O	4.11750200	2.51432900	-0.00024900
H	5.08406600	2.50785200	-0.00020100
O	4.72510300	-2.25806100	0.00032600
H	5.66057000	-2.01415500	0.00047200

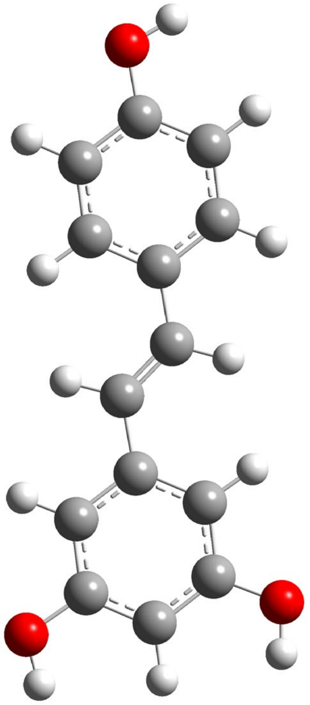

19(1)-dmg

Charge=0, Multiplicity=2

C	-5.03300900	-0.15287400	-0.00003600
C	-4.10776900	-1.28066300	-0.00007300
C	-2.75836800	-1.09310100	-0.00005000
C	-2.19301900	0.22193500	0.00001000
C	-3.08331600	1.33870600	0.00004800
C	-4.43845900	1.17627300	0.00002800
H	-4.54050600	-2.27663400	-0.00012000
H	-2.10393500	-1.95902800	-0.00007700
H	-2.65424400	2.33774000	0.00009400
H	-5.11413300	2.02624900	0.00005700
O	-6.26708700	-0.32067900	-0.00005900
C	-0.77862800	0.47631900	0.00003300
H	-0.49885400	1.52753100	0.00008400
C	0.19889100	-0.46815100	-0.00000700
H	-0.07448800	-1.52044300	-0.00005800
C	1.63893300	-0.21403000	0.00000900
C	2.18160400	1.08113600	0.00009000
C	2.49175000	-1.32705700	-0.00006400
C	3.56217600	1.24216600	0.00009100
H	1.55759800	1.96747700	0.00015000
C	3.87263200	-1.14333600	-0.00006400
H	2.09035300	-2.33546500	-0.00012500
C	4.42089400	0.13844300	0.00001000
H	5.49922800	0.27959400	0.00000000
O	4.04216100	2.51439500	0.00017300
H	5.00896800	2.50985300	0.00016800
O	4.64971200	-2.25815900	-0.00013800
H	5.58584900	-2.01592500	-0.00012600

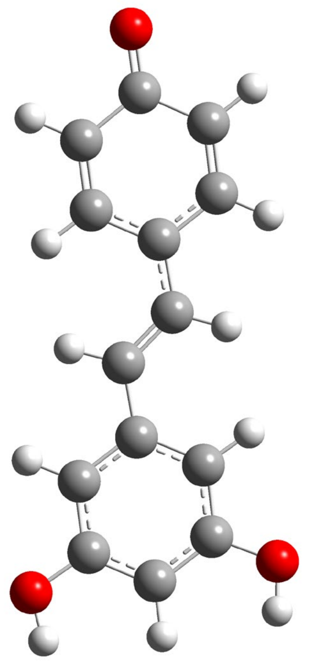

19(2)-dmg

Charge=0, Multiplicity=2

C	-4.91365300	-0.16009700	0.00002400
C	-4.05484100	-1.26468600	-0.00036900
C	-2.68234900	-1.07252600	-0.00039400
C	-2.12596000	0.21922400	-0.00001900
C	-3.00730800	1.30890200	0.00036500
C	-4.38796400	1.13158900	0.00038600
H	-4.48026500	-2.26317700	-0.00065600
H	-2.03692900	-1.94570600	-0.00070500
H	-2.60417800	2.31854600	0.00064900
H	-5.05269900	1.99219300	0.00068500
O	-6.24973500	-0.40924100	0.00002200
H	-6.74345300	0.42245800	0.00036600
C	-0.68107100	0.47820100	-0.00000200
H	-0.41379800	1.53414900	0.00014900
C	0.29611700	-0.44337700	-0.00013200
H	0.04201500	-1.50141700	-0.00025100
C	1.74039200	-0.15765700	-0.00009400
C	2.27656700	1.12330000	-0.00022600
C	2.62937700	-1.25040900	0.00008900
C	3.70893200	1.34275300	-0.00013400
H	1.65391800	2.01168700	-0.00039800
C	4.03657100	-1.06839600	0.00016700

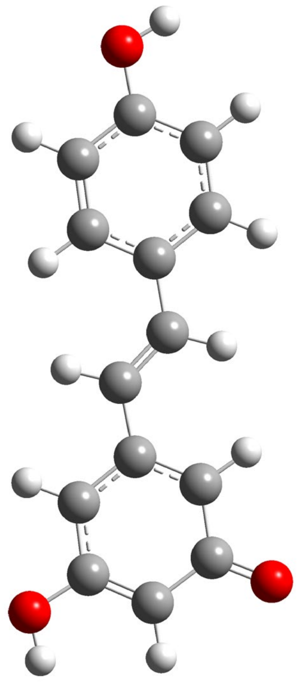

H	2.24619000	-2.26682800	0.00018200
C	4.58205000	0.18773100	0.00007000
H	5.65568600	0.35294400	0.00013900
O	4.17156700	2.50732000	-0.00023700
O	4.76808200	-2.21069100	0.00033700
H	5.71304000	-2.00125500	0.00048000

19(3)-dmg

Charge=0, Multiplicity=2

C	-4.90600200	-0.10333500	-0.00001300
C	-4.07825000	-1.23137300	0.00082600
C	-2.70071200	-1.07783600	0.00080100
C	-2.10833800	0.19776000	-0.00004400
C	-2.95893600	1.31148000	-0.00089000
C	-4.34391000	1.17295600	-0.00090200
H	-4.53121200	-2.21768900	0.00149900
H	-2.08065200	-1.96911400	0.00147200
H	-2.52782300	2.30954300	-0.00155500
H	-4.98333700	2.05224100	-0.00160600
O	-6.24877700	-0.31423500	0.00011500
H	-6.71850300	0.53120600	-0.00058800
C	-0.65677100	0.41780500	-0.00008400
H	-0.36545900	1.46696600	-0.00040500
C	0.29438500	-0.53098200	0.00019100
H	0.01063200	-1.58129700	0.00041500
C	1.74668600	-0.29899100	0.00012900
C	2.30879600	1.00004700	0.00043100
C	2.60335200	-1.38505000	-0.00023900
C	3.71068900	1.19437400	0.00031400
H	1.68296900	1.88609000	0.00077500
C	4.04499300	-1.21986800	-0.00039800
H	2.22159500	-2.40186900	-0.00044900
C	4.57359400	0.12790500	-0.00011800
H	5.65230000	0.25695200	-0.00025800
O	4.11717500	2.48944100	0.00067000
H	5.08383000	2.53573800	0.00059600
O	4.80387600	-2.21497100	-0.00077400

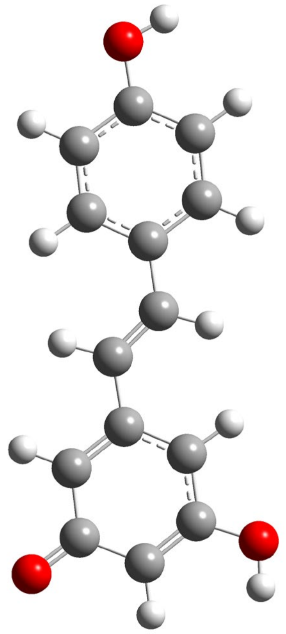

20

Charge=0, Multiplicity=1

C	-4.58576200	-0.50803100	-0.04787000
C	-3.69519700	-1.56287500	-0.21340400
C	-2.32311200	-1.32642500	-0.20944100
C	-1.82216500	-0.02874400	-0.03191900
C	-2.73510400	1.02541100	0.13126300
C	-4.10289300	0.79476000	0.12440900
H	-4.07892800	-2.57088900	-0.35087400
H	-1.64880800	-2.16422500	-0.35175400
H	-2.38199300	2.04391800	0.26725600
O	-4.96529700	1.83269500	0.28516500
H	-5.87261500	1.49487500	0.25689500
O	-5.94654400	-0.63164800	-0.04262300
H	-6.20859200	-1.55377700	-0.16843800
C	-0.38674000	0.28958300	-0.01100000
H	-0.16236800	1.35405500	0.03865300
C	2.97613500	-1.31062000	0.09113800
C	4.34406500	-1.04440200	0.11589900
C	4.82033200	0.26231300	0.02615100
C	3.89731100	1.30478800	-0.09778700
C	2.52919300	1.05933000	-0.12733400
C	2.05935800	-0.25833000	-0.02547900
H	2.63580000	-2.33855800	0.16624000
H	5.88816900	0.46712700	0.04736500
H	1.85597100	1.90208800	-0.23684000
C	0.62551400	-0.59261400	-0.03676600
H	0.40911200	-1.65940300	-0.05629500
O	4.30023500	2.60130500	-0.19966800
H	5.26518900	2.65396500	-0.17837500
O	5.18528300	-2.10816200	0.23191900
H	6.10404600	-1.80757100	0.24066100

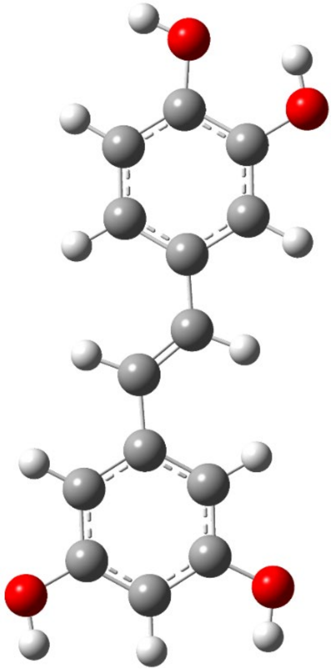

20[(1)]-dmg

Charge=0, Multiplicity=2

C	-4.62646300	-0.53003300	-0.02166200
C	-3.71421300	-1.58231400	-0.09650500
C	-2.36364200	-1.29520700	-0.08750400
C	-1.86705500	0.05136800	-0.00287700
C	-2.76386700	1.09326400	0.07158300
C	-4.18135700	0.86083000	0.06435400
H	-4.07400400	-2.60378000	-0.16040100
H	-1.66209200	-2.11992500	-0.14577800
H	-2.42490000	2.12329100	0.13672500
O	-5.06302600	1.75015600	0.12763900
O	-5.93582000	-0.73696000	-0.02570500
H	-6.35490700	0.14630500	0.03374600
C	-0.43013900	0.34981500	0.00657900
H	-0.19394600	1.41030100	0.06938100
C	2.90310800	-1.31018700	0.02560800
C	4.27589500	-1.07031000	0.05622900
C	4.77434100	0.23081400	0.02648400
C	3.87072500	1.29519100	-0.04309400
C	2.49775200	1.07660200	-0.07390400
C	2.00700600	-0.23621100	-0.03356100

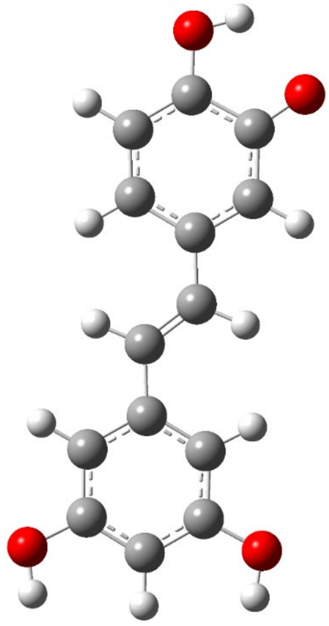

H	2.54411200	-2.33406800	0.05329000
H	5.84590900	0.41555000	0.05577600
H	1.83901500	1.93557300	-0.13430600
C	0.56842500	-0.54758700	-0.04826500
H	0.34136800	-1.61047200	-0.09753200
O	4.29663900	2.58660400	-0.08578700
H	5.26249200	2.62227300	-0.06554200
O	5.09628100	-2.15340400	0.11650600
H	6.02126100	-1.87241300	0.13263000

20(2)-dmg

Charge=0, Multiplicity=2

C	-4.65932800	-0.56929700	-0.02432200
C	-3.68276800	-1.63072100	-0.10161500
C	-2.34864600	-1.35142500	-0.09426700
C	-1.86289200	-0.00243200	-0.00773000
C	-2.78700400	1.05830100	0.06919600
C	-4.13915900	0.79729800	0.06026500
H	-4.05172400	-2.64941900	-0.16672500
H	-1.63991500	-2.17009200	-0.15536300
H	-2.43860800	2.08485400	0.13485700
O	-5.04850600	1.77145400	0.13088500
H	-5.92251000	1.33480600	0.10978300
O	-5.89821300	-0.73215200	-0.02512500
C	-0.45144400	0.31854400	0.00270900
H	-0.22401800	1.38127400	0.05528800
C	2.90464800	-1.30707300	0.03167600
C	4.27382400	-1.04982800	0.05823300
C	4.75465400	0.25819800	0.02409500
C	3.83851600	1.31235500	-0.04536000
C	2.46862200	1.07717800	-0.07246200
C	1.99488800	-0.24298700	-0.02792900
H	2.55773500	-2.33492200	0.06234900
H	5.82399500	0.45617700	0.04984700
H	1.79902400	1.92763300	-0.13198400
C	0.56660900	-0.57238700	-0.03973200
H	0.35003900	-1.63755800	-0.07585200
O	4.24878800	2.60805300	-0.09199200
H	5.21422300	2.65626300	-0.07418400
O	5.10781600	-2.12156600	0.11914000
H	6.02955100	-1.82968600	0.13217300

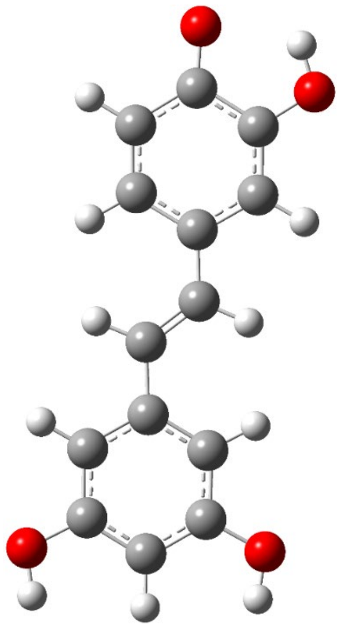

20(3)-dmg

Charge=0, Multiplicity=2

C	-4.54210000	-0.52611800	-0.04245000
C	-3.64140700	-1.57513500	-0.19037200
C	-2.27172700	-1.32580600	-0.18767000
C	-1.78378000	-0.02089700	-0.02888000
C	-2.70645900	1.02726900	0.11695600
C	-4.07192400	0.78377400	0.11128600
H	-4.01524400	-2.58868200	-0.31335300
H	-1.58915000	-2.15926600	-0.31523800
H	-2.36316100	2.05094300	0.23843000
O	-4.94398200	1.81577900	0.25506800
H	-5.84827100	1.46959400	0.23055000
O	-5.90127400	-0.66201900	-0.03765100
H	-6.15515400	-1.58819700	-0.15022800
C	-0.35181000	0.31127800	-0.01026000
H	-0.13933100	1.37856100	0.03028700
C	3.02612700	-1.25640900	0.08548800
C	4.39319900	-0.97942900	0.10358700
C	4.86720100	0.32683900	0.01531300
C	3.92223100	1.35654400	-0.09704000
C	2.54910300	1.11154600	-0.12081200
C	2.09945800	-0.21492800	-0.02217200
H	2.69579000	-2.28739700	0.15914600
H	5.92825100	0.55252400	0.02800400
H	1.86609200	1.94622900	-0.22085600
C	0.66872300	-0.56139500	-0.02941400
H	0.46416400	-1.63043400	-0.04244000
O	4.36251200	2.61371600	-0.18869400
O	5.23986300	-2.03668200	0.21114500
H	6.15805900	-1.73303700	0.21538600

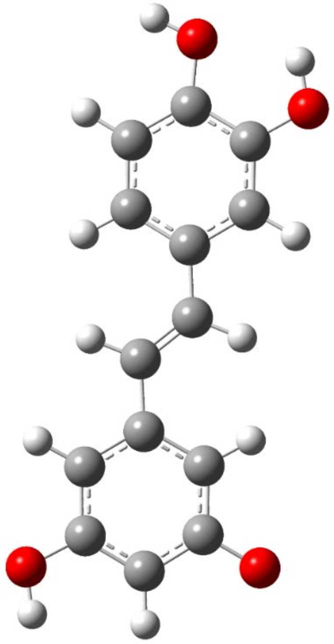

20(4)-dmg

Charge=0, Multiplicity=2

C	4.54800400	-0.48867900	0.00001000
C	3.66996900	-1.56708700	0.00014300
C	2.29529100	-1.34848900	0.00017900
C	1.77980600	-0.04456100	0.00003800
C	2.67936900	1.03338800	-0.00009300
C	4.04987900	0.82006100	-0.00009400
H	4.06506200	-2.57995300	0.00021000
H	1.63072200	-2.20584500	0.00029200
H	2.31467800	2.05687900	-0.00017800
O	4.89958800	1.87999300	-0.00023500
H	5.81142900	1.55319000	-0.00001000
O	5.90936200	-0.59385700	-0.00009200
H	6.18356600	-1.52114300	0.00100100
C	0.34082000	0.25623100	0.00003500
H	0.10646000	1.31950300	-0.00003200
C	-3.01067300	-1.36937400	-0.00009700
C	-4.44143700	-1.12595400	-0.00010400
C	-4.89618900	0.24840300	-0.00003500
C	-3.97601800	1.26595900	0.00006800
C	-2.58676700	0.99565000	0.00010300
C	-2.09632300	-0.33165400	0.00001900

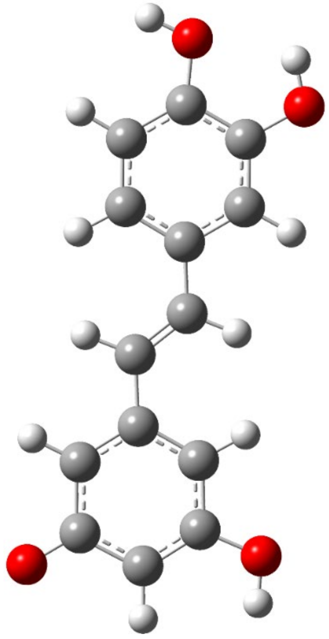

H	-2.68553700	-2.40561700	-0.00017100
H	-5.96635300	0.43646100	-0.00005600
H	-1.91376600	1.84636900	0.00020200
C	-0.65833100	-0.64110800	0.00005900
H	-0.42989000	-1.70503400	0.00006600
O	-4.30990600	2.58120700	0.00016500
H	-5.27250200	2.68148600	0.00015300
O	-5.25257700	-2.07897100	-0.00019500

1-β-TS

Charge=0, Multiplicity=2

C	2.33143100	-0.73427000	-2.21688800
C	3.18277600	-0.77491000	-1.10512000
C	2.93091900	0.04262500	0.00324800
C	1.81862900	0.89162300	0.05826100
C	0.95285700	0.90842400	-1.06316900
C	1.23867100	0.11182600	-2.18848600
H	2.54765200	-1.35919800	-3.07748400
H	3.61294900	0.01037700	0.84167500
H	0.55920900	0.16591300	-3.03521800
O	-0.16526400	1.66029100	-1.08473500
H	-1.10818600	0.96090800	-0.97230600
O	4.23236600	-1.62852200	-1.19179600
C	5.11371600	-1.72334400	-0.08282600
H	5.86089500	-2.46733600	-0.35802100
H	5.60929100	-0.76580500	0.11208800
H	4.58332900	-2.05532400	0.81682600
C	1.56233100	1.78024900	1.28142900
C	2.65868800	1.62932800	2.34376200
H	2.71376100	0.60735400	2.73455400
H	3.64481300	1.91083600	1.95861200
H	2.42997900	2.29203600	3.18444400
C	1.52852500	3.25784300	0.84904300
H	0.72493600	3.44552600	0.13398200
H	1.36891400	3.89409700	1.72723700
H	2.47959400	3.54777000	0.38854300
C	0.22313700	1.40055300	1.93715800
H	0.06676700	2.01320400	2.83280000
H	-0.61304500	1.57443300	1.25741600
H	0.22072400	0.34768700	2.24221700
N	-4.10722800	-1.49416200	1.23576400
H	-3.93665600	-0.81534000	1.96409100
H	-4.89092700	-2.12381900	1.34865300
C	-3.14613600	-1.80852000	0.33945700
O	-3.21228700	-2.76151600	-0.42258800
C	-1.97041000	-0.80524100	0.27117600
H	-1.93693800	-0.24142300	1.20957500
N	-0.71805200	-1.51588100	0.12819700
H	-0.27458700	-1.57528400	-0.78067900
C	-0.19395100	-2.22063800	1.14986500
O	-0.69861000	-2.28643800	2.26446200
H	0.74879200	-2.72896400	0.89103600
C	-2.18470700	0.11731500	-0.91136900
H	-2.05303100	-0.41244100	-1.86396900
C	-3.38198000	1.04616200	-0.90536600

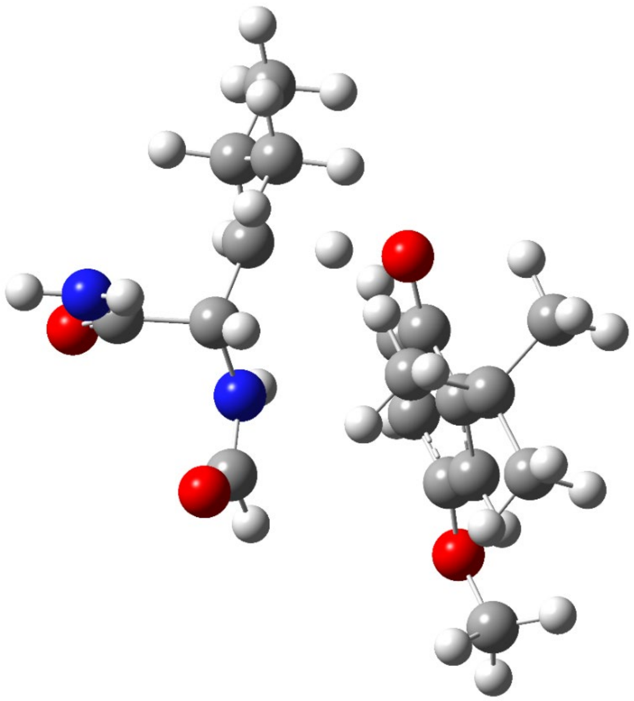

H	-4.28514200	0.41454300	-0.84907200
C	-3.44017800	1.83682000	-2.21402900
H	-2.56373200	2.48751800	-2.30950800
H	-4.33487600	2.46608900	-2.24462600
H	-3.46470100	1.16802300	-3.08045600
C	-3.39048300	1.99446900	0.29703700
H	-3.38885500	1.46039300	1.25283000
H	-4.28421700	2.62557500	0.27318300
H	-2.51240200	2.65055100	0.27201800

1-γ-TS

Charge=0, Multiplicity=2

C	3.31724200	2.05225300	0.91223600
C	4.15889000	1.30480500	0.07955300
C	3.84123100	-0.02232200	-0.23214300
C	2.67610000	-0.63534000	0.24503000
C	1.81974600	0.13443900	1.07621400
C	2.16995300	1.46118900	1.40381400
H	3.58446600	3.07446700	1.16048000
H	4.51618300	-0.58877900	-0.85886200
H	1.50419400	2.01017800	2.06374800
O	0.68720500	-0.36912500	1.58843500
H	-0.27321100	0.08399700	0.98945900
O	5.26770700	1.94365200	-0.37147400
C	6.15621700	1.22863000	-1.21536500
H	6.96187500	1.92151100	-1.45763100
H	6.57157000	0.35357100	-0.70287100
H	5.65769300	0.91306600	-2.13881000
C	2.36710700	-2.10223100	-0.08013800
C	3.45308600	-2.74055200	-0.95605400
H	3.54919800	-2.23672800	-1.92412000
H	4.43131100	-2.73942200	-0.46316000
H	3.18246500	-3.78316300	-1.15142800
C	2.28649900	-2.91166100	1.22775600
H	1.48745300	-2.54362800	1.87415300
H	2.09252100	-3.96567000	0.99714700
H	3.23340500	-2.85348300	1.77624300
C	1.03501000	-2.20984300	-0.84200300
H	0.83304000	-3.26027800	-1.08216700
H	0.20205000	-1.82932900	-0.24779300
H	1.08072200	-1.64996000	-1.78262000
N	-4.24019800	1.75508800	-1.52893200
H	-4.64256200	2.30573000	-0.78370300
H	-4.38233100	2.06844000	-2.48076300
C	-3.85994500	0.47616800	-1.31587200
O	-3.56999400	-0.30281500	-2.21512100
C	-3.77344500	0.04740000	0.15669400
H	-4.05847900	0.87786300	0.80895100
N	-4.71924100	-1.02756700	0.39880900
H	-4.43237700	-1.98108500	0.21279300
C	-6.02211800	-0.78391000	0.65807900
O	-6.50660200	0.33483500	0.76974500
H	-6.61877600	-1.70200100	0.77789800
C	-2.35296800	-0.42927600	0.49701400
H	-2.11074300	-1.28010500	-0.15150700

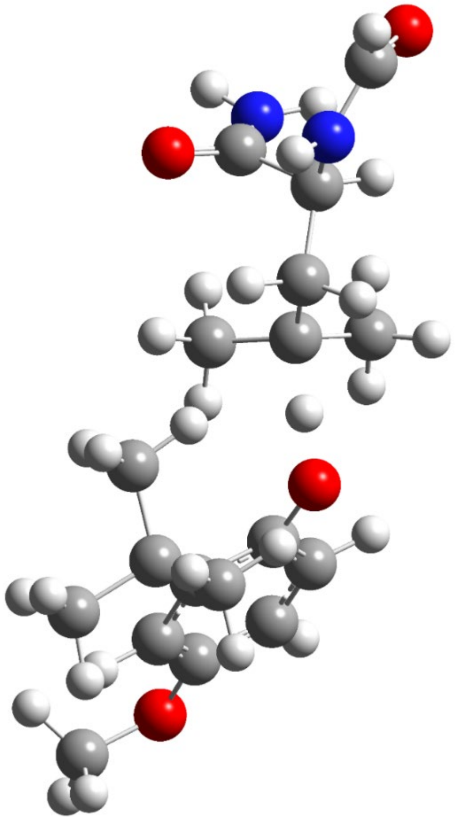

H	-2.38024600	-0.78789400	1.53380200
C	-1.28688800	0.64523200	0.35916000
C	-0.74859100	0.91865600	-1.02702900
H	0.25991200	1.35076800	-0.97024200
H	-1.37719600	1.65301700	-1.55199400
H	-0.70741000	0.01325300	-1.63661300
C	-1.52285900	1.89241100	1.18195400
H	-1.78851200	1.65332400	2.21729000
H	-0.63308100	2.53030100	1.18736700
H	-2.33919600	2.49072400	0.74972500

1-δ-TS

Charge=0, Multiplicity=2

N	1.94363700	1.40042000	-1.87706300
H	1.15588800	0.86646300	-1.51683700
H	1.79453900	2.08482100	-2.60688600
C	3.20322600	1.11886800	-1.50321800
O	4.19767500	1.66222300	-1.97950700
C	3.35042100	0.08607200	-0.38398500
H	2.51640700	-0.62431000	-0.40105400
N	4.57296300	-0.64798800	-0.65165800
H	5.28990600	-0.17552900	-1.19108200
C	4.80184400	-1.87506500	-0.14841600
O	4.00953600	-2.50078400	0.54686400
H	5.78709800	-2.27983100	-0.43096400
C	3.43150100	0.77989600	0.99122400
H	4.20027300	1.56006000	0.93065900
H	3.78552600	0.02694500	1.70648000
C	2.12305400	1.37438400	1.52166400
H	1.73002200	2.10687200	0.80153200
C	2.39671800	2.12364600	2.83838500
H	2.78382000	1.43498000	3.59762700
H	1.48256200	2.58219700	3.22628600
H	3.13854300	2.91374800	2.68120400
C	1.07977000	0.31097500	1.76805800
H	1.45145400	-0.63133700	2.18157800
H	0.15698900	0.65657500	2.24521800
C	-2.79740900	2.15885900	-0.37238200
C	-3.88241600	1.29383100	-0.18615500
C	-3.67215400	-0.08488100	-0.07022200
C	-2.38906000	-0.64446900	-0.12942700
C	-1.30487300	0.24005100	-0.33455600
C	-1.52545700	1.62451800	-0.44538400
H	-2.97401600	3.22610600	-0.45763500
H	-4.52520800	-0.73355600	0.07284400
H	-0.66414300	2.27292700	-0.58826600
O	-0.03257100	-0.21330200	-0.44075700
H	0.53572700	-0.04694800	0.57839200
O	-5.10392300	1.87885600	-0.12850100
C	-6.23893400	1.04514100	0.04854500
H	-7.10077700	1.71212900	0.05927900
H	-6.33974200	0.33356100	-0.77866000
H	-6.18764500	0.50225200	0.99900100
C	-2.17724700	-2.15881000	-0.00491900
C	-3.49699300	-2.90199000	0.24103600

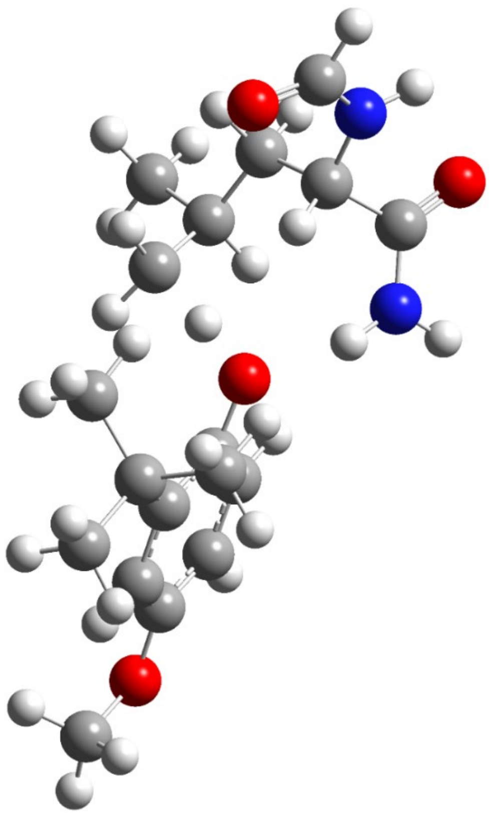

H	-3.97709800	-2.58725400	1.17430100
H	-4.20698700	-2.76337800	-0.58134000
H	-3.28944400	-3.97369900	0.31959400
C	-1.56814200	-2.70307000	-1.31031400
H	-0.59894600	-2.24589300	-1.51921600
H	-1.42786900	-3.78689400	-1.22711000
H	-2.23593100	-2.51090000	-2.15756600
C	-1.23978700	-2.47270100	1.17613300
H	-1.19123200	-3.55730900	1.32462600
H	-0.22440500	-2.11648600	0.99487100
H	-1.61161400	-2.02081500	2.10298200

2-β-TS

Charge=0, Multiplicity=2

C	2.17832900	2.07577200	-0.44534000
C	2.69770400	0.79781600	-0.19997600
C	2.00733600	-0.37000800	-0.62492300
C	0.80586700	-0.17247500	-1.29651700
C	0.26697900	1.10558400	-1.54357000
C	0.96431800	2.23287300	-1.10319500
H	2.71476000	2.95743300	-0.11622700
H	0.54736700	3.21990700	-1.27599100
O	-0.89547800	1.23047100	-2.20895000
H	-1.81353900	1.11510700	-1.47399400
O	3.87631800	0.61455300	0.44972600
C	4.60238600	1.75426300	0.88130700
H	4.02579600	2.34294900	1.60361300
H	5.50027300	1.36846900	1.36430400
H	4.88860700	2.38661300	0.03372800
H	0.23217800	-1.01873600	-1.65789900
C	2.55137600	-1.78019500	-0.35422800
C	1.60961400	-2.86021700	-0.90367300
H	0.61838100	-2.81488800	-0.43817700
H	1.48928600	-2.78657300	-1.98992300
H	2.03400600	-3.84471200	-0.68227600
C	3.91825300	-1.96633700	-1.03859300
H	4.66502500	-1.27094600	-0.65168300
H	4.27739100	-2.98784200	-0.86859400
H	3.82941900	-1.81486500	-2.12017000
C	2.68056300	-2.01592100	1.16228600
H	3.04862400	-3.03200000	1.34517600
H	3.37214600	-1.31158300	1.62746000
H	1.70310000	-1.92279900	1.64926500
N	-0.24647700	0.92472300	2.02997700
H	-0.10768400	-0.07297100	2.13961700
H	0.33158800	1.56974700	2.55144600
C	-1.23898000	1.40227400	1.25113100
O	-1.49337500	2.59298800	1.11925700
C	-2.07921900	0.29947500	0.57538600
H	-1.40256900	-0.51221600	0.27864600
N	-2.97532400	-0.27773500	1.57984800
H	-3.87887300	0.15258000	1.73672000
C	-2.59625300	-1.29890200	2.37443500
O	-1.48940400	-1.82806200	2.33833000
H	-3.38499300	-1.62763800	3.06808300

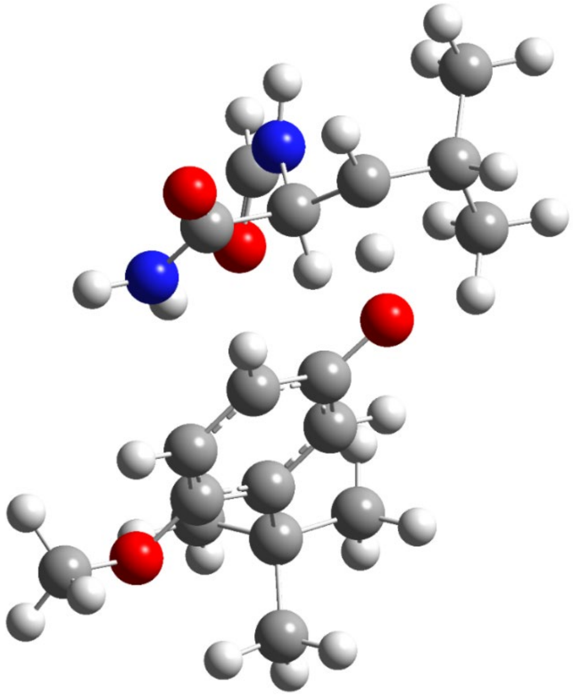

C	-2.82721900	0.81999300	-0.62525400
H	-3.26214200	1.81072200	-0.46686000
C	-3.68453200	-0.13222200	-1.44071600
H	-3.76202600	0.31124200	-2.44345200
C	-5.12073900	-0.23652600	-0.89724300
H	-5.14822900	-0.78781300	0.04824000
H	-5.75110900	-0.77509500	-1.61291100
H	-5.56139400	0.75292500	-0.73762200
C	-3.05743000	-1.51929900	-1.58599900
H	-2.04544600	-1.45737200	-2.00010200
H	-3.65843800	-2.13914300	-2.25862300
H	-3.00350800	-2.03471700	-0.61938000

2-γ-TS

Charge=0, Multiplicity=2

C	-2.20823500	-1.95072700	-0.75208700
C	-3.22284100	-1.19011400	-0.15832200
C	-3.22096000	0.22953300	-0.24274800
C	-2.16811300	0.81214100	-0.93864400
C	-1.13819900	0.06182100	-1.54194500
C	-1.16813400	-1.33206700	-1.43726100
H	-2.22241000	-3.03160700	-0.68197900
H	-0.37849800	-1.91977900	-1.89635700
O	-0.14979400	0.68570300	-2.19753800
H	0.76724000	0.90357100	-1.41908500
O	-4.24983800	-1.76552600	0.51965100
C	-4.27868500	-3.17811200	0.64497400
H	-3.39789900	-3.54647100	1.18248900
H	-5.17564000	-3.40651000	1.22097300
H	-4.34420500	-3.66240300	-0.33552000
H	-2.10153200	1.88963400	-1.04064700
C	-4.31992600	1.08100000	0.41002800
C	-4.08164100	2.57921700	0.17905000
H	-3.13375900	2.91519500	0.61373600
H	-4.08705500	2.83665400	-0.88546300
H	-4.88636700	3.14298200	0.66173900
C	-5.69370300	0.73534200	-0.19388200
H	-5.96582000	-0.30634300	-0.01660500
H	-6.46274700	1.37402700	0.25562400
H	-5.69386100	0.91595000	-1.27466900
C	-4.33545900	0.84966400	1.93265600
H	-5.09454200	1.49249800	2.39304200
H	-4.56421600	-0.18686400	2.18521300
H	-3.36393400	1.10776300	2.36937300
N	4.27435100	0.94407100	2.10355800
H	4.86757800	1.62427700	1.65027100
H	4.24803600	0.93377700	3.11507500
C	3.79601300	-0.12345700	1.42845800
O	3.23733000	-1.07020100	1.96837700
C	3.96193000	-0.07283700	-0.09674000
H	4.50106200	0.83255200	-0.38956600
N	4.76884300	-1.20232400	-0.52390800
H	4.31691000	-2.09866200	-0.65923400
C	6.11912100	-1.15703800	-0.52212500
O	6.77874300	-0.16669900	-0.23501500

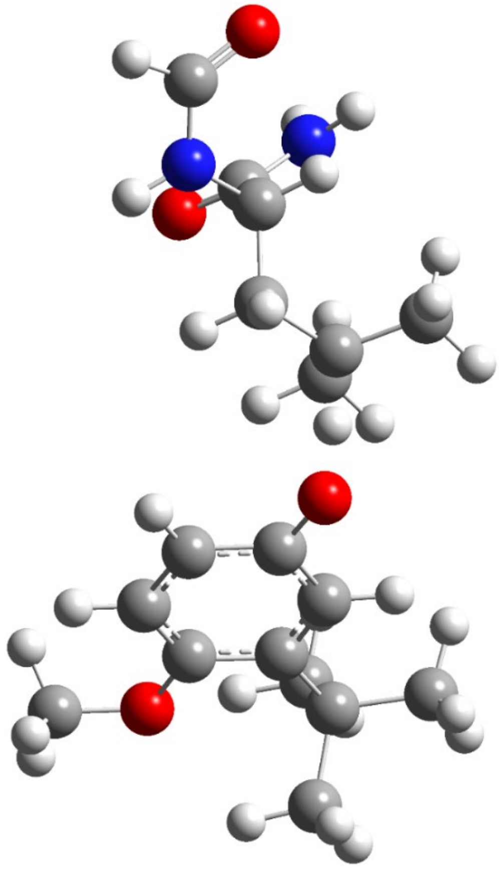

H	6.58314300	-2.10895100	-0.82525000
C	2.59419200	-0.12279600	-0.79608500
H	2.08685700	-1.04987600	-0.50082600
H	2.79635300	-0.17386600	-1.87313500
C	1.69453900	1.06801100	-0.50857800
C	0.89940600	1.03035400	0.77673300
H	0.11622200	1.79743900	0.76724700
H	1.54115900	1.23764900	1.64542800
H	0.42812600	0.05488500	0.93662900
C	2.28326900	2.42095100	-0.84149400
H	2.79915800	2.41203600	-1.80734400
H	1.50335100	3.18872900	-0.87236300
H	3.00795900	2.72795900	-0.07206800

2-δ-TS

Charge=0, Multiplicity=2

N	1.90218000	0.28262300	-1.57668300
H	1.42874800	-0.52556200	-1.18496700
H	1.51540100	0.73969400	-2.39182600
C	3.12384800	0.64881200	-1.14327200
O	3.77754400	1.56177400	-1.63523700
C	3.64460800	-0.12361600	0.08363700
H	3.09681800	-1.06778700	0.18180100
N	5.04873000	-0.43851000	-0.09748200
H	5.73986200	0.18814900	0.29608000
C	5.45279500	-1.31075100	-1.04647600
O	4.69124900	-1.98070600	-1.72993000
H	6.54879400	-1.38051100	-1.13697300
C	3.48958400	0.72978100	1.34775600
H	3.98984500	1.69046400	1.17083800
H	4.02537100	0.22529600	2.16275400
C	2.04577900	0.97945400	1.79551700
H	1.47468300	1.41584000	0.96293800
C	2.03350100	1.99872700	2.94851600
H	2.59031100	1.61186300	3.80946500
H	1.01070400	2.21278100	3.27150800
H	2.49829300	2.93844300	2.63240100
C	1.36432100	-0.28789600	2.25442900
H	1.98326700	-0.96749700	2.84816200
H	0.36163800	-0.15155900	2.67137900
C	-3.04853300	-2.17512600	0.49098100
C	-3.53825500	-0.92896500	0.08298000
C	-2.65393700	0.13248000	-0.25225100
C	-1.29267300	-0.14006700	-0.16964600
C	-0.79000000	-1.39085700	0.23212700
C	-1.67928200	-2.40553300	0.58126800
H	-3.73296900	-2.97468600	0.74646400
H	-1.29587200	-3.36870200	0.90326600
O	0.54431600	-1.60883300	0.26429900
H	1.01408800	-1.03623100	1.18749200
O	-4.86873200	-0.67423500	-0.00833800
C	-5.78480400	-1.71245500	0.30238900
H	-5.68399900	-2.03200200	1.34547800
H	-6.77695500	-1.28793400	0.14870500
H	-5.64987300	-2.57199300	-0.36339600

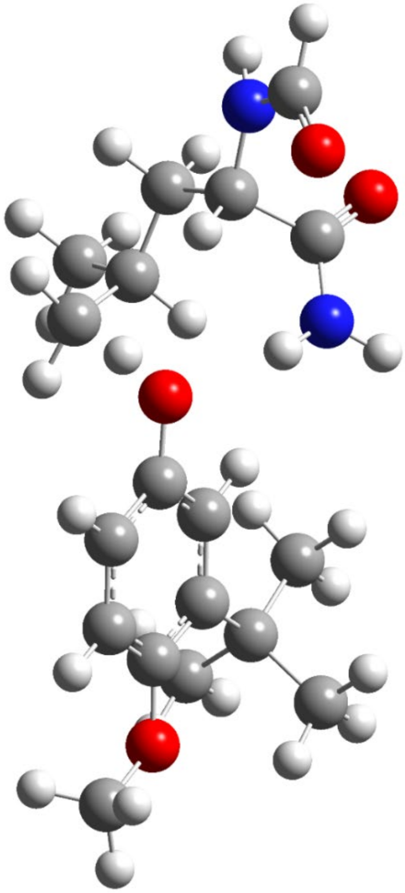

H	-0.56524200	0.62637800	-0.41434800
C	-3.16664200	1.51226900	-0.68918200
C	-2.00777800	2.47367700	-0.98739700
H	-1.37677500	2.64337100	-0.10776400
H	-1.37401900	2.11299300	-1.80519100
H	-2.42053100	3.44092300	-1.29091300
C	-4.00316400	1.38676600	-1.97618100
H	-4.88761600	0.76539000	-1.82753000
H	-4.33082400	2.38198100	-2.29772000
H	-3.40356500	0.95120400	-2.78325500
C	-4.01194900	2.14419900	0.43241600
H	-4.34459900	3.14143300	0.12251200
H	-4.89390100	1.54471900	0.66381500
H	-3.41698500	2.25536700	1.34596300

3-β-TS

Charge=0, Multiplicity=2

N	3.43109400	2.52559800	0.87009300
H	2.94834300	2.28566600	1.72398200
H	4.00483600	3.35912500	0.86629700
C	3.06005700	1.98953500	-0.31256000
O	3.45460100	2.39568000	-1.39582700
C	2.14975200	0.74146100	-0.21495200
H	1.70735800	0.69929700	0.78821200
N	1.06555400	0.85448500	-1.16663000
H	1.13006600	0.38418500	-2.06034600
C	0.00331100	1.64665500	-0.91839400
O	-0.12705300	2.31581000	0.10074000
H	-0.74755900	1.63716100	-1.72487700
C	2.98042000	-0.49537700	-0.48774400
H	3.19096900	-0.62592500	-1.55578800
C	4.17577900	-0.77367800	0.40235800
H	4.87643200	0.07129300	0.28620400
C	4.88909800	-2.04448200	-0.06335900
H	4.23093900	-2.91469800	0.04071600
H	5.78619100	-2.22571900	0.53643800
H	5.19087100	-1.96822700	-1.11290000
C	3.80096300	-0.88067600	1.88394200
H	3.33176000	0.03171800	2.26451800
H	4.69571300	-1.06606800	2.48608500
H	3.10501800	-1.71203100	2.04092200
C	-2.40522700	-0.83710200	-0.56102800
C	-1.75372300	-0.59201100	0.66043200
C	0.12964200	-1.92986300	-0.12764400
O	1.38003300	-2.39480200	0.05853000
H	2.14058000	-1.54986500	-0.22667000
C	-2.37647500	0.32104900	1.68725800
H	-2.78893700	-0.26913400	2.51528300
H	-1.59932100	0.96778400	2.10609100
C	-3.46720700	1.16718600	1.03796800
H	-3.00429900	1.92700800	0.39629500
H	-4.06278000	1.68690800	1.79509900
C	-4.39273400	0.30916800	0.17396200
C	-5.41771500	1.16661400	-0.54936300
H	-6.01103700	0.55355200	-1.23442300

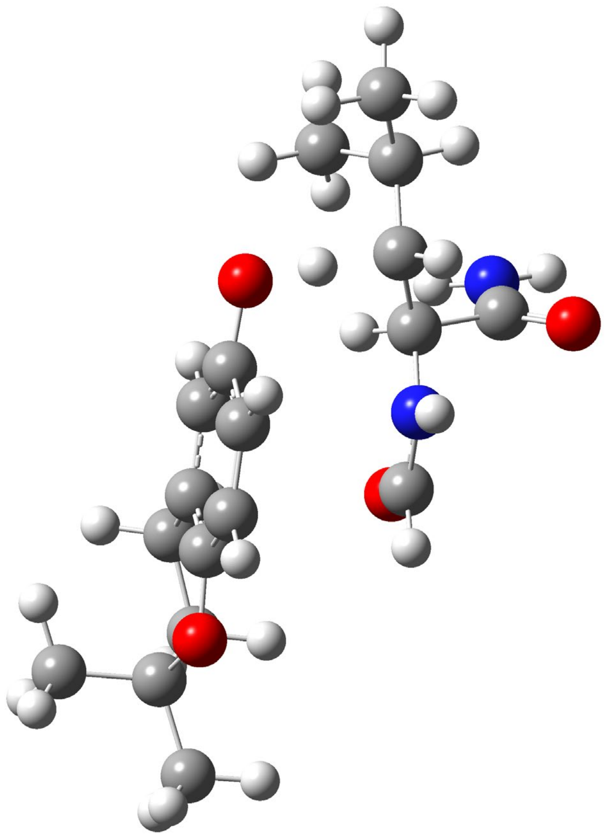

H	-6.09331200	1.63196300	0.17460200
H	-4.92218400	1.95508500	-1.12361100
C	-5.07480700	-0.80036600	0.97177100
H	-5.63519200	-0.37286400	1.80954100
H	-5.77040100	-1.34840000	0.32940800
H	-4.34780500	-1.51343400	1.37174200
O	-3.61391100	-0.30718100	0.88165000
C	-1.80129700	-1.63349100	-1.54788200
H	-2.33985000	-1.80420600	2.47520300
C	-0.54234000	-2.16886300	-1.33972900
H	-0.05419200	-2.76657700	2.10406400
C	-0.49559800	-1.15716200	0.85865100
H	0.04051000	-0.96181300	1.78574700

3-γ-TS

Charge=0, Multiplicity=2

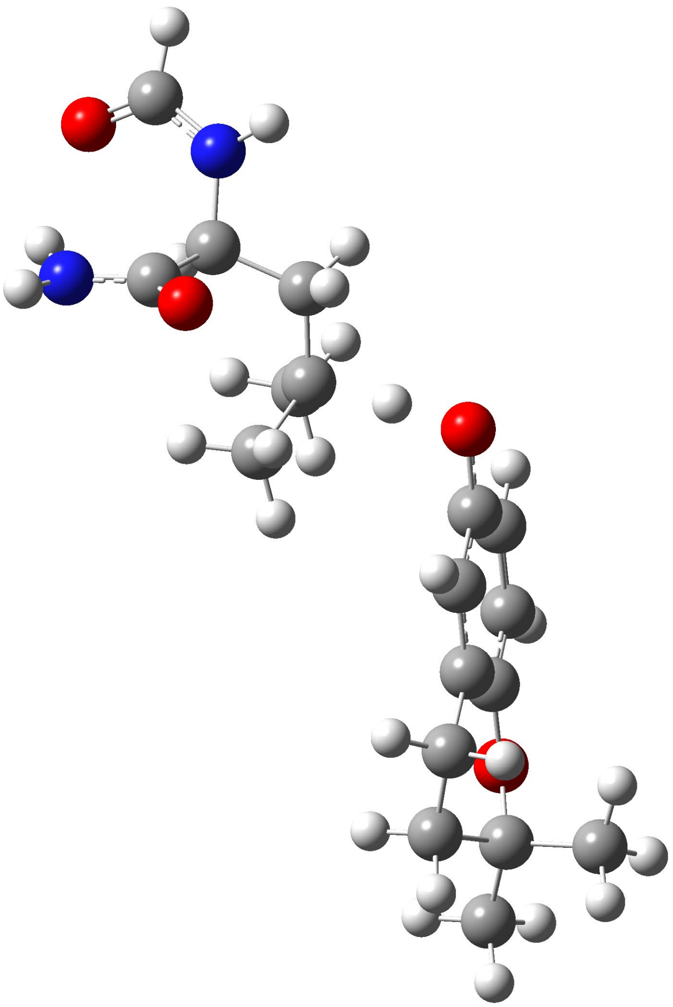

N	-5.21671100	2.00606400	0.91541300
H	-5.68222400	1.41644800	1.59633000
H	-5.50553300	2.97251200	0.83581300
C	-4.56916800	1.43654000	-0.12652700
O	-4.18418300	2.05543700	-1.10950300
C	-4.38560900	-0.08769500	0.01185200
H	-4.30670000	-0.34138400	1.07450900
N	-5.60306000	-0.72513300	-0.47932300
H	-5.64996800	-1.00891000	-1.45101200
C	-6.69035100	-0.89185800	0.29878200
O	-6.76662300	-0.51982400	1.46637700
H	-7.51828200	-1.40727600	-0.21071100
C	-3.16172900	-0.59944700	-0.75588700
H	-3.13757100	-0.10508500	-1.73396800
H	-3.29368900	-1.67584800	-0.91511600
C	-1.85378900	-0.36931600	-0.01705200
C	-1.43321700	1.06737100	0.18525300
H	-0.39858600	1.11746800	0.54400700
H	-2.05922900	1.55319000	0.95092800
H	-1.52400400	1.65075100	-0.73509600
C	-1.62608200	-1.25397100	1.18724300
H	-1.84991300	-2.30289400	0.96645600
H	-0.58759200	-1.18383700	1.53061500
H	-2.26149000	-0.94297400	2.03017400
C	3.58969100	-0.72456700	0.21313300
C	3.05377300	0.32499300	-0.55426800
C	1.09060700	-1.06173800	-1.00609500
O	-0.09244300	-1.22668700	-1.61437200
H	-0.97889800	-0.82051800	-0.88425900
C	3.81361300	1.62097100	-0.70791300
H	4.26039800	1.67064300	-1.70879800
H	3.11909200	2.46384300	-0.63458600
C	4.89891400	1.72870700	0.35982000
H	4.44344300	1.93683200	1.33569400
H	5.58884800	2.54783600	0.13396500
C	5.68939900	0.42390500	0.47720400
C	6.69970900	0.49781500	1.60980500
H	7.19527400	-0.46871800	1.74096400
H	7.46044700	1.25105000	1.38392900

H	6.20574300	0.76789100	2.54791500
C	6.36768300	0.03333000	-0.83439200
H	7.02677400	0.83904200	-1.17358100
H	6.96665200	-0.87052300	-0.68941400
H	5.63593000	-0.16749500	-1.62255800
O	4.78493600	-0.64200000	0.85582500
C	2.87947200	-1.92352900	0.37871800
H	3.32512000	-2.70930000	0.98107200
C	1.64171100	-2.09147800	-0.21743100
H	1.08345300	-3.01540000	-0.09612000
C	1.80850600	0.13602500	-1.15077200
H	1.36860300	0.93438400	-1.74526000

3-δ-TS

Charge=0, Multiplicity=2

N	-2.62544500	-0.81575800	1.62113100
H	-1.89387300	-0.95001000	0.92980000
H	-2.44997800	-1.05886800	2.58693400
C	-3.87781600	-0.48419800	1.25274800
O	-4.81734600	-0.39054500	2.03467400
C	-4.05665300	-0.15529800	-0.24166500
H	-3.23345800	-0.59857000	-0.81334400
N	-5.29779500	-0.73076200	-0.72301800
H	-6.12202500	-0.14403600	-0.76347200
C	-5.47025100	-2.06937100	-0.77391900
O	-4.58476300	-2.88182700	-0.54644700
H	-6.49101700	-2.36781700	-1.06267400
C	-4.10617700	1.36333000	-0.44672600
H	-4.89984500	1.76572900	0.19568800
H	-4.39881600	1.55322700	-1.48810300
C	-2.79646100	2.10679900	-0.16552800
H	-2.44878400	1.86627500	0.84911100
C	-3.04581300	3.62480500	-0.22221900
H	-3.39803100	3.91981100	-1.21715800
H	-2.12957500	4.18123000	-0.00539500
H	-3.80559800	3.91405600	0.51141100
C	-1.71464500	1.75199600	-1.15699400
H	-2.03621600	1.68241800	-2.20072400
H	-0.77385800	2.29779500	-1.03587000
C	3.23113600	0.17325800	0.50450800
C	2.95283700	-0.33700900	-0.77597500
C	0.57503900	-0.25523400	-0.24398800
O	-0.70426700	-0.49947900	-0.60809500
H	-1.23645000	0.50989500	-0.90275400
C	4.07865300	-0.64453900	-1.73376600
H	4.23394400	-1.72954700	-1.78284100
H	3.80046000	-0.32317400	-2.74236700
C	5.36074800	0.05142700	-1.28510900
H	5.28884000	1.12878400	-1.47802500
H	6.22468300	-0.32813300	-1.83952100
C	5.59993900	-0.14524000	0.21315000
C	6.81900100	0.63296800	0.67878800
H	6.92647100	0.55712400	1.76488200
H	7.72138900	0.22829100	0.21083800
H	6.72714500	1.68920400	0.40883800

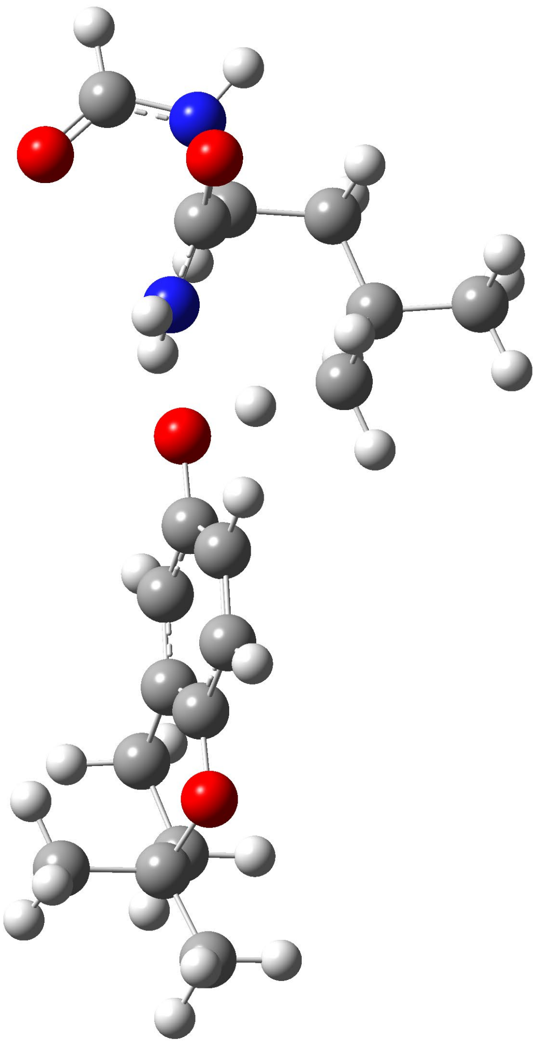

C	5.71398700	-1.61890100	0.59749400
H	6.51592300	-2.09744400	0.02623500
H	5.94287100	-1.70881000	1.66341900
H	4.78305200	-2.15995400	0.40340700
O	4.48982700	0.42957000	0.94611400
C	2.19005900	0.48064100	1.39321600
H	2.44409900	0.88107900	2.36972700
C	0.87195800	0.26869900	1.02622600
H	0.06001600	0.50418700	1.70955500
C	1.61935700	-0.53277100	-1.13261100
H	1.37430600	-0.91702800	-2.12013800

4-β-TS

Charge=0, Multiplicity=2

N	3.72877900	2.23418000	1.16933600
H	3.26857200	1.83541700	1.97530000
H	4.32624000	3.03734500	1.31664600
C	3.26938900	1.99368400	-0.07890800
O	3.62518300	2.62019800	-1.06571500
C	2.31813600	0.77920700	-0.20150800
H	1.89431000	0.55587900	0.78639400
N	1.22634800	1.09334600	-1.09604600
H	1.20774500	0.69107000	-2.02429400
C	0.24628500	1.94232300	-0.73043900
O	0.21176700	2.52387500	0.34677300
H	-0.53059300	2.06979800	-1.50235000
C	3.09359600	-0.40631800	-0.73424000
H	3.32499900	-0.29800800	-1.80060200
C	4.25034700	-0.94911000	0.07983200
H	5.00325200	-0.14539600	0.15459900
C	4.89355500	-2.13035700	-0.64967100
H	4.18202500	-2.95906200	-0.74012700
H	5.76780900	-2.49359600	-0.10088900
H	5.21724200	-1.84662000	-1.65622400
C	3.84534100	-1.35308200	1.50045800
H	3.41795800	-0.52067000	2.06866600
H	4.71911900	-1.71317900	2.05230200
H	3.10727900	-2.16223500	1.46828700
C	-2.27032400	-0.67051000	0.47958300
C	-2.07302800	-1.00336600	0.87138400
C	0.17352000	-1.75485400	-0.31511100
O	1.35609400	-2.26801700	-0.70407600
H	2.17153900	-1.42984700	-0.74602900
C	-3.17595300	-0.77823600	1.87938800
H	-2.93922900	0.09946300	-2.49484500
H	-3.22699300	-1.63206300	2.56230100
C	-4.51419000	-0.57931100	1.17367800
H	-4.87415900	-1.53758500	0.78004700
H	-5.26983000	-0.20082400	1.86922300
C	-4.37917600	0.39215800	0.00043400
C	-5.68530500	0.50673200	0.76850800
H	-5.55036900	1.13268400	1.65556400
H	-6.45287800	0.96154400	0.13516700
H	-6.03222200	-0.48104000	1.08627400
C	-3.87954200	1.76861300	-0.43339500

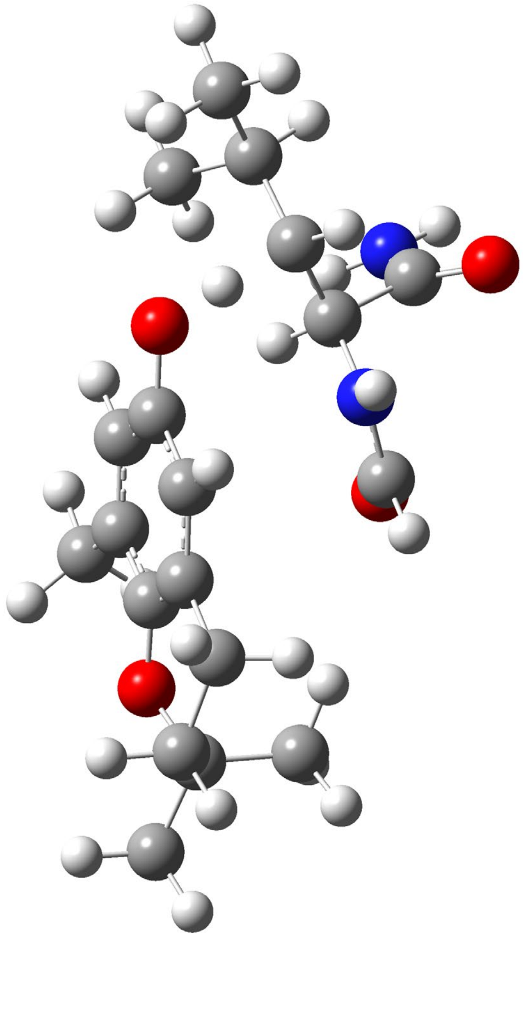

H	-4.54704500	2.19329900	-1.19016700
H	-3.84933800	2.44296800	0.42731700
H	-2.87033500	1.71474500	-0.85249000
O	-3.43429000	-0.15125700	0.95279900
C	-1.26748200	-0.89230000	1.44748200
C	-0.05986000	-1.43637400	1.03559000
H	0.73209900	-1.61414000	1.75961900
C	-0.84204000	-1.53439000	1.25236500
H	-0.65879700	-1.78929900	2.29408400
C	-1.52653600	-0.51885500	2.87997400
H	-1.74087500	0.55137500	2.96762100
H	-2.39528100	-1.05301200	3.27769500
H	-0.65855900	-0.75401700	3.50030700

4-γ-TS

Charge=0, Multiplicity=2

N	4.73110200	-1.34568200	1.87989400
H	4.86170800	-0.51612800	2.44156600
H	4.90616300	-2.24322700	2.31360700
C	4.73017000	-1.26675200	0.53085700
O	4.80803400	-2.24207300	-0.20520200
C	4.57797500	0.14831100	-0.04614000
H	4.52864800	0.87965500	0.76620500
N	5.74825400	0.46918300	-0.84393500
H	5.75342800	0.21901100	-1.82551800
C	6.90515700	0.86898700	-0.27357800
O	7.05123600	1.05887400	0.92713000
H	7.71650000	1.02688500	-1.00161800
C	3.31858900	0.24468700	-0.92117300
H	3.40805900	-0.48169300	-1.73795000
H	3.31647400	1.25085900	-1.35903700
C	2.01682900	0.01794000	-0.17071800
C	1.59343100	-1.41324800	0.06927400
H	0.53432400	-1.45277300	0.35199700
H	2.15987200	-1.86398700	0.89702400
H	1.74357800	-2.03347000	-0.81938700
C	1.77197000	0.93999700	1.00101300
H	2.02568300	1.97842700	0.76145300
H	0.72183700	0.90148400	1.31309400
H	2.37323800	0.63333600	1.87060300
C	-3.41712900	0.52483100	0.05943500
C	-2.92120700	-0.58154500	0.64917000
C	-0.92443300	0.70899700	-1.18731800
O	0.25963700	0.80915400	-1.80712500
H	1.14109800	0.42896400	-1.05882800
C	-3.72364700	-1.85931700	0.72466700
H	-4.18010400	-1.95096200	1.71823800
H	-3.05470600	-2.71804800	0.60921100
C	-4.80269100	-1.87530600	0.35399400
H	-4.34601600	-2.04167900	1.33732600
H	-5.51842700	-2.68479000	0.17918800
C	-5.55229500	-0.54270200	0.40051900
C	-6.55489700	-0.51798100	1.54226100
H	-7.01756700	0.47042200	1.62029400
H	-7.34184600	-1.25737800	1.36567600

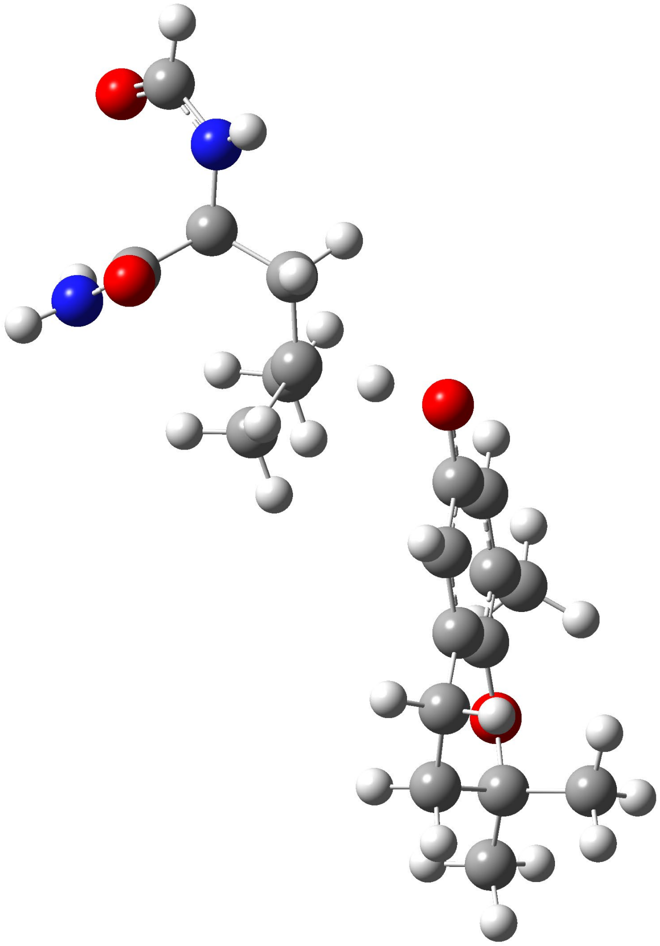

H	-6.06224800	-0.74961400	2.49130300
C	-6.22990500	-0.20874600	0.92715100
H	-6.91275500	-1.01423700	1.21617400
H	-6.80374100	0.71753300	-0.82953800
H	-5.49848400	-0.07242900	1.72920200
O	-4.61309300	0.51503700	0.70848400
C	-2.67809400	1.72338900	0.16800400
C	-1.44079000	1.79659500	-0.45363700
H	-0.84967700	2.70713500	-0.38976800
C	-1.67487400	-0.47273400	1.26427500
H	-1.26567600	-1.31693100	1.81535900
C	-3.24961300	2.87676500	0.94548000
H	-4.21013500	3.19432900	0.52711300
H	-3.43462800	2.59690800	1.98761800
H	-2.56386400	3.72695200	0.92968500

4-δ-TS

Charge=0, Multiplicity=2

N	2.51652700	0.10272700	-1.77398300
H	1.90830400	-0.47912700	-1.20560100
H	2.23313900	0.35599600	-2.71098600
C	3.76410600	0.40685200	-1.36868300
O	4.56645400	1.04138300	-2.04477800
C	4.11841000	-0.04574400	0.06041900
H	3.42756900	-0.83599500	0.37460700
N	5.46109000	-0.59351700	0.08055200
H	6.22862700	0.00974500	0.34875500
C	5.75380600	-1.74287400	-0.56571300
O	4.91888900	-2.45686900	-1.10339900
H	6.82680900	-1.99325600	-0.54742400
C	4.05541000	1.14668900	1.02153800
H	4.71588600	1.93142700	0.63104900
H	4.46394000	0.82349100	1.98829100
C	2.65537400	1.72541500	1.25143400
H	2.20178300	1.98253200	0.28377800
C	2.76452000	3.02344900	2.07175800
H	3.21686400	2.82330300	3.04967500
H	1.77876400	3.46778000	2.23620000
H	3.38845100	3.75498700	1.54753600
C	1.75169700	0.76091000	1.98062500
H	2.20958500	0.22930300	2.82049800
H	0.75108100	1.14028200	2.20970700
C	-3.18941300	-0.65911100	0.16568000
C	-2.37240800	0.18249000	-0.60603800
C	-0.42229400	-0.90786700	0.35259900
O	0.92083100	-1.05505500	0.42748500
H	1.37249600	-0.25330800	1.15462700
C	-2.98954800	1.19404700	-1.54272500
H	-2.90503200	2.19900200	-1.11036600
H	-2.42864800	1.21118200	-2.48242700
C	-4.45389100	0.85361700	-1.80337300
H	-4.52234300	-0.02228000	2.45986500
H	-4.96507900	1.68324700	-2.30168000
C	-5.18242800	0.52430600	-0.49942400
C	-6.61106600	0.08150000	-0.76764700

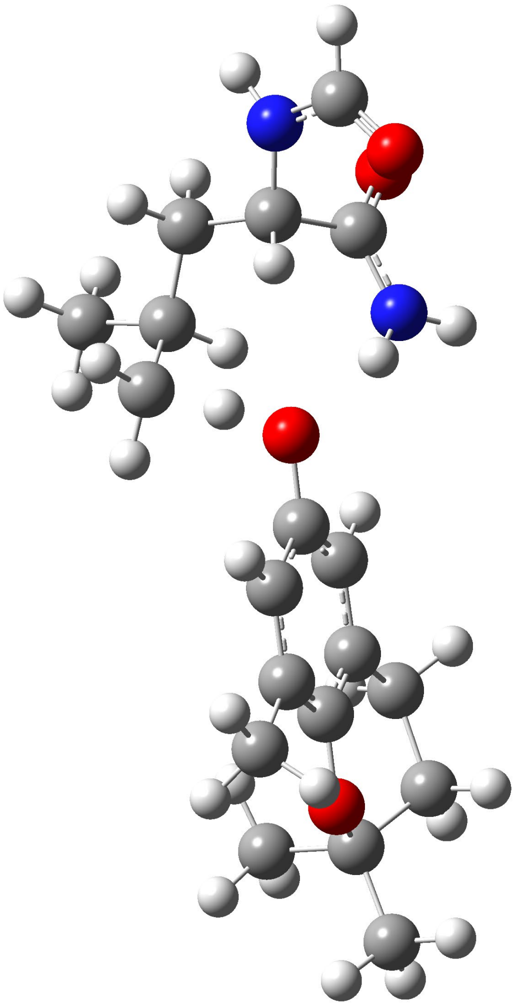

H	-7.09145400	-0.23656000	0.16246600
H	-7.18644300	0.91053000	-1.19042300
H	-6.62800600	-0.75387100	1.47386200
C	-5.14885500	1.68185800	0.49674900
H	-5.57688400	2.58229800	0.04439800
H	-5.73371400	1.42538000	1.38494000
H	-4.12736700	1.90872400	0.81643500
O	-4.54780800	-0.62175300	0.11737400
C	-2.64050200	-1.63127600	1.02993800
C	-1.25990400	-1.73530700	1.11856300
H	-0.80672800	-2.47222800	1.77638000
C	-0.98837400	0.04754500	-0.49646800
H	-0.33565300	0.68818200	-1.08706000
C	-3.55844400	-2.51821100	1.82419300
H	-4.22423200	-1.92512700	2.45919300
H	-4.19666700	-3.11448900	1.16408200
H	-2.98246600	-3.19699300	2.45709800

5-β-TS

Charge=0, Multiplicity=2

C	2.43451400	-0.67828700	0.34360100
C	1.79067900	-0.15573700	-0.78831300
C	-0.07750100	-1.63982300	0.36007500
O	-1.30582200	-2.11033700	0.66008400
H	-2.11187000	-1.46035800	0.14131700
C	2.43141800	0.96497700	-1.57145900
H	2.78778400	0.59014200	-2.54064700
H	1.67507300	1.72669200	-1.78778600
C	3.57803700	1.59408100	-0.78555700
H	3.17431300	2.22485800	0.01641100
H	4.18810700	2.23340800	-1.43160500
C	4.46420800	0.52797200	-0.14452900
C	5.53892200	1.15593900	0.72722200
H	6.10707500	0.38029400	1.24953800
H	6.23001400	1.73676500	0.10898800
H	5.08982300	1.82153300	1.47047100
C	5.08152000	-0.41504600	-1.17559000
H	5.66756500	0.15447400	-1.90416100
H	5.74319300	-1.13007100	-0.67768200
H	4.31507600	-0.97878700	-1.71561300
O	3.65779500	-0.25536400	0.76611500
C	1.83595200	-1.67308600	1.14644800
C	0.58192600	-2.13191100	0.78031000
H	0.08218500	-2.89309000	1.37491300
C	0.52797100	-0.64920100	-1.15499400
C	-0.18349800	-0.08456100	2.35339500
H	-0.41069900	0.97794100	-2.20033200
H	0.43983500	-0.15563900	-3.25190000
H	-1.11528000	-0.62181600	2.53667300
C	2.56527600	-2.19872900	2.35182800
H	3.52346000	-2.64530500	2.06695800
H	2.78933900	-1.39559300	3.06147200
H	1.96595600	-2.95687200	2.86139600
N	-4.10000300	2.52811300	-0.06315000
H	-3.72975200	2.56747200	-1.00193800

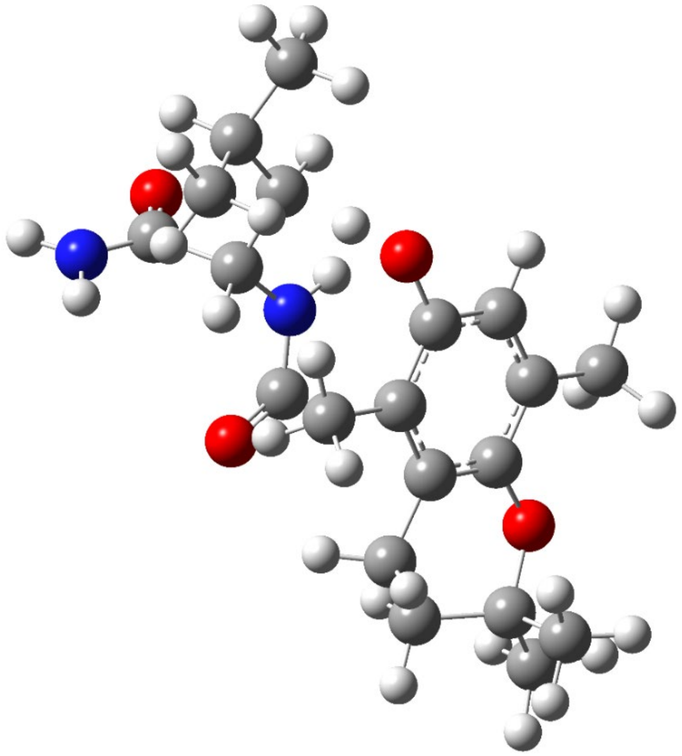

H	-4.77929600	3.22539900	0.21337700
C	-3.51154200	1.77363100	0.88917700
O	-3.80579400	1.82534200	2.07536000
C	-2.46583100	0.75779800	0.37538200
H	-2.18887600	1.00923900	-0.65386600
N	-1.26426900	0.84791600	1.18001300
H	-1.18418600	0.28039400	2.01432400
C	-0.27214700	1.71337700	0.88855700
O	-0.28788500	2.49161600	-0.05886100
H	0.57213400	1.65525500	1.59425300
C	-3.05890900	-0.63437400	0.44834100
H	-3.07954900	-1.02277100	1.47351500
C	-4.32440200	-0.92075700	0.33433700
H	-5.10653900	-0.23857100	0.04276100
C	-4.78801000	-2.35575500	0.07707000
H	-4.04111100	-3.07121500	0.43963100
H	-5.72963700	-2.55703000	0.59706700
H	-4.94362300	-2.53669600	0.99131500
C	-4.16102400	-0.67025800	1.83695200
H	-3.85783700	0.35782000	-2.06050400
H	-5.10732400	-0.85437200	2.35497500
H	-3.40714500	-1.34723800	2.25384400

5-γ-TS

Charge=0, Multiplicity=2

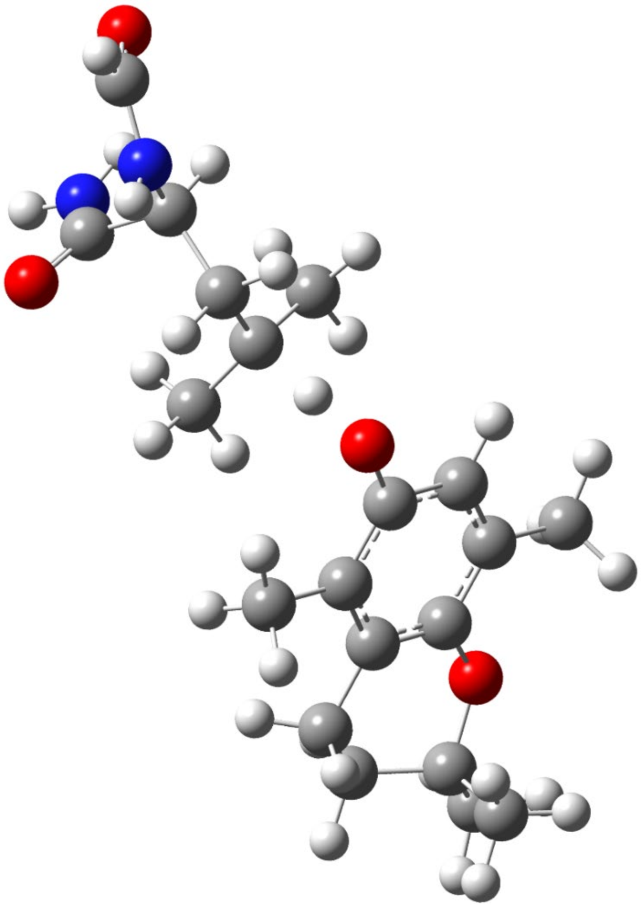

C	-3.38884100	0.59634500	0.05347700
C	-2.86221300	-0.61917500	0.40995900
C	-0.88842900	0.57276700	-1.17467000
O	0.29765200	0.60244200	-1.80064800
H	1.18360200	0.41971500	-1.00195700
C	-3.64819900	-1.89744300	0.23159800
H	-4.04019300	-2.22906300	1.20278100
H	-2.98273100	-2.69506500	0.11481900
C	-4.78865300	-1.70819900	0.76466800
H	-4.38960800	-1.67264400	1.78569100
H	-5.49243900	-2.54504100	0.71316900
C	-5.53495000	-0.40012000	0.50844400
C	-6.59994600	-0.15808100	1.56505900
H	-7.06525300	0.82137500	1.41896800
H	-7.37655200	-0.92580900	1.49633800
H	-6.16162900	-0.19122600	2.56697500
C	-6.13524700	-0.33609800	0.89481800
H	-6.81854800	-1.17686600	1.05226100
H	-6.69466900	0.59602400	-1.01798700
H	-5.36051200	-0.37249200	1.66640600
O	-4.60490100	0.69693900	0.65883200
C	-2.67339400	1.80708900	-0.05952900
C	-1.43078900	1.77095300	-0.66829100
H	-0.85199600	2.68420900	-0.78533200
C	-1.60531300	-0.63506200	1.03566700
C	-1.02863900	-1.92504200	1.55120500
H	-0.83009400	-2.62722000	0.73277500
H	-1.72392100	-2.42089400	2.23798500
H	-0.09265800	-1.74087100	2.08018200
C	-3.27497100	3.08201500	0.46364300

H	-4.22750900	3.29921600	-0.03049000
H	-3.48292300	3.01077400	1.53618700
H	-2.59624700	3.92157000	0.29584000
N	4.77152200	-0.50588600	2.26875000
H	4.99436900	0.45989800	2.46387100
H	4.88024900	-1.17863700	3.01688800
C	4.71102000	-0.95295200	0.99509200
O	4.66501900	-2.13900300	0.69425100
C	4.65353300	0.13943300	-0.08289400
H	4.74218800	1.12578400	0.38217100
N	5.77722700	-0.01424900	-0.98904800
H	5.66988900	-0.60648000	-1.80365700
C	7.01463000	0.41628500	-0.66253900
O	7.28405700	1.01803800	0.36901500
H	7.77099300	0.18651600	-1.42953700
C	3.34178800	0.04656600	-0.87833100
H	3.30226400	-0.93526300	-1.36612600
H	3.39590600	0.81282200	-1.66217400
C	2.08710600	0.25803200	-0.04923900
C	1.55193500	-0.92861000	0.71908000
H	0.49547400	-0.77166200	0.97398500
H	2.08986000	-1.06288100	1.66895800
H	1.64179900	-1.85599300	0.14638600
C	2.01181000	1.56710300	0.70170200
H	2.32390900	2.41160400	0.07775500
H	0.99075500	1.75304000	1.05407900
H	2.65694100	1.54434500	1.59351100

5-δ-TS

Charge=0, Multiplicity=2

C	2.60091900	0.46166900	-0.33054700
C	2.51176400	-0.93112700	-0.48252500
C	0.18097600	-0.67078800	-1.08597000
O	-1.00912200	-1.18908200	-1.47561900
H	-1.55509200	-1.69344200	-0.58099500
C	3.73017800	-1.79339300	-0.24816200
H	3.61751100	-2.34651800	0.69440000
H	3.80590400	-2.54992000	-1.03590500
C	5.00255700	-0.95133300	-0.22202700
H	5.26480900	-0.64319300	-1.24138200
H	5.84254000	-1.52952200	0.17593900
C	4.81370600	0.31214200	0.61528900
C	6.04105900	1.20559800	0.54677000
H	5.85789000	2.14468900	1.07747200
H	6.89584200	0.70442800	1.01093000
H	6.29082400	1.43492700	-0.49341100
C	4.44656600	0.00154300	2.06530000
H	5.21849100	-0.62431800	2.52469200
H	4.36552300	0.93137600	2.63604300
H	3.48990500	-0.52479100	2.13463700
O	3.74917000	1.09742800	0.03217400
C	1.49796600	1.30655600	-0.56958600
C	0.29691800	0.72155100	-0.93681000
H	-0.57312900	1.35118000	-1.11479000
C	1.28703500	-1.50869400	-0.85640500

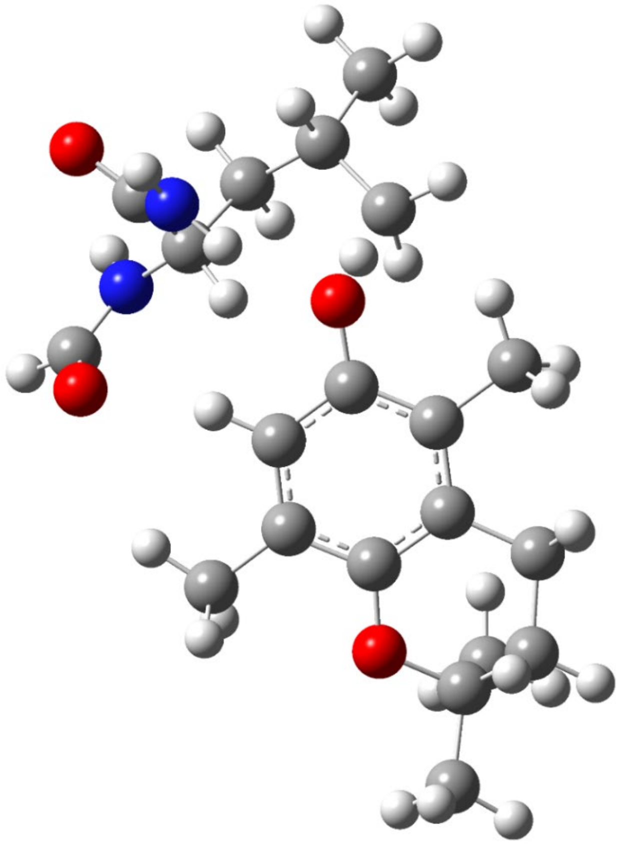

C	1.16125100	-3.00090000	-1.00464300
H	1.86662000	-3.38454800	-1.75020400
H	1.38052300	-3.51349800	-0.06071000
H	0.15563200	-3.27714600	-1.32314800
C	1.63819900	2.79522500	-0.41339200
H	1.99164000	3.05257500	0.59036200
H	2.37003200	3.19947700	-1.12080600
H	0.67606500	3.28395300	-0.58311000
N	-3.52844700	0.24391500	-1.88575300
H	-2.57981500	-0.11977100	-1.91452000
H	-4.06931600	0.28814800	-2.73914700
C	-4.06066400	0.74530400	-0.75568400
O	-5.18333100	1.23466000	-0.68732000
C	-3.18269100	0.61670000	0.50279800
H	-2.13948900	0.45337600	0.21309800
N	-3.24362000	1.84660800	1.27021800
H	-3.91598000	1.91388400	2.02446200
C	-2.70567200	2.99110000	0.80028000
O	-2.03313600	3.06426600	-0.22070200
H	-2.91191900	3.86612900	1.43682300
C	-3.67827600	-0.53958400	1.37637800
H	-4.73041500	-0.35135600	1.62539600
H	-3.10803800	-0.52128300	2.31466400
C	-3.54826800	-1.93080600	0.74918400
H	-4.08898600	-1.95182300	-0.20735600
C	-4.20843700	-2.96998400	1.67358800
H	-3.70104600	-2.99809700	2.64457800
H	-4.16216500	-3.97077900	1.23496800
H	-5.26065600	-2.71769500	1.84243300
C	-2.11008200	-2.32883300	0.51400400
H	-1.39925600	-2.02809000	1.29207900
H	-1.96864900	-3.36186700	0.18594400

6-β-TS

Charge=0, Multiplicity=2

N	4.26569800	2.33774600	-0.06166900
H	4.09403000	2.26946000	0.93133100
H	4.92434600	3.03424800	-0.38576500
C	3.43128100	1.76369400	-0.95398200
O	3.47793300	1.96948700	-2.15894000
C	2.43000000	0.74679500	-0.36009900
H	2.42958900	0.83586700	0.73178500
N	1.09103200	1.05660600	-0.81986800
H	0.73483700	0.60832400	-1.65451900
C	0.31644300	1.95759500	-0.18172900
O	0.66994500	2.59235600	0.80557800
H	-0.68102600	2.07437500	-0.63538700
C	2.83730600	-0.64905600	-0.78330300
H	2.56981600	-0.86335500	-1.82491900
C	4.21833000	-1.13923700	-0.39763300
H	4.95092600	-0.45938400	-0.86704500
C	4.45375200	-2.54354500	-0.95630400
H	3.74777500	-3.25569100	-0.51392200
H	5.46770100	-2.88566200	-0.72741700
H	4.32427800	-2.56486600	-2.04312700
C	4.45077600	-1.11555300	1.11631000
H	4.32033500	-0.11552900	1.54219600
H	5.46923800	-1.44243600	1.34745400
H	3.75252200	-1.79499700	1.61786800
C	-2.45690100	-0.35345100	0.53552000
C	-2.14164300	-1.28025100	0.47428600
C	0.04221900	-1.58108400	0.56020200
O	1.26052700	-2.15925800	0.52223000
H	1.98621600	-1.49390100	-0.09411400
C	-3.14443700	-1.61915900	1.55331900
H	-2.84051300	-1.15578800	2.50058800
H	-3.14476800	-2.70047700	1.72318900
C	-4.53921000	-1.14231700	1.16022800
H	-4.95413200	-1.79665600	0.38402200
H	-5.21881300	-1.17093100	2.01784100
C	-4.49074700	0.27929800	-0.59900300
C	-5.86013400	0.72431100	-0.11281500
H	-5.79385600	1.70742900	0.36277700
H	-6.55371800	0.79168900	-0.95631300
H	-6.25935900	0.01040300	0.61372600
C	-3.91792900	1.28282700	-1.59872800
H	-4.51529100	1.28558000	-2.51616400
H	-3.93463200	2.28836800	-1.16769200
H	-2.88411400	1.04228400	-1.86490800
O	-3.65911300	0.28638700	0.58378600
C	-1.54958900	-0.05760500	1.57440400
C	-0.29331000	-0.67169600	1.58816800
C	-0.88461300	-1.87399700	0.44619000
H	-0.60294100	-2.58772300	1.21766200
C	-1.90423700	0.95094400	2.63498700
H	-1.25066500	1.82845700	2.55387800
H	-2.93836800	1.28044600	2.54184200
H	-1.76308200	0.53174300	3.63687900

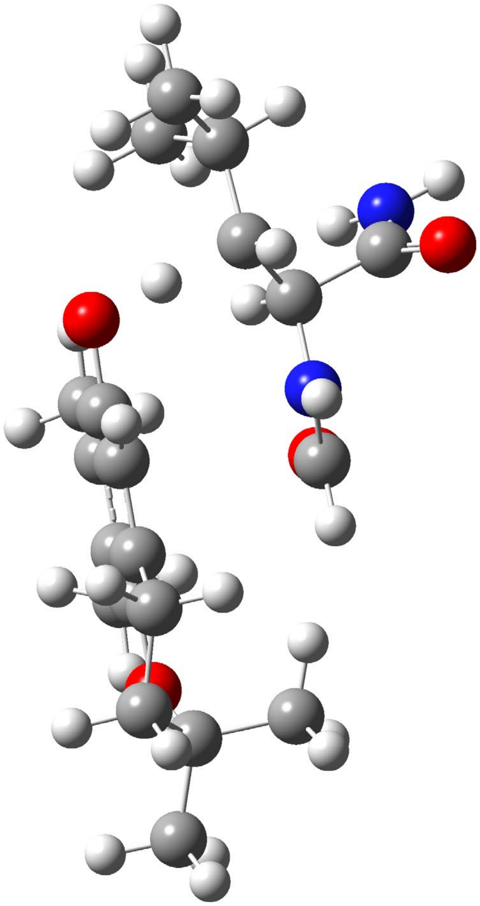

C	0.70361000	-0.32499000	2.65907800
H	1.64499800	-0.85297700	2.49796100
H	0.89569500	0.75479800	2.66838700
H	0.32574000	-0.59646700	3.65172600

6-γ-TS

Charge=0, Multiplicity=2

N	4.77441900	-1.72097400	1.68667000
H	4.75841800	-1.00065400	2.39439800
H	5.01754000	-2.66140500	1.97038600
C	4.83252900	-1.40160800	0.37545300
O	5.06003500	-2.21616400	-0.51009900
C	4.55028500	0.07038600	0.04120100
H	4.38764400	0.63976800	0.96137100
N	5.71036400	0.64268000	-0.61901600
H	5.79299000	0.54992800	-1.62433700
C	6.78032700	1.08314400	0.07749400
O	6.84726600	1.10426600	1.29972300
H	7.59780200	1.44436300	-0.56645700
C	3.32567200	0.19258500	-0.87956100
H	3.52614500	-0.37149500	-1.79849600
H	3.23864000	1.25397000	-1.14497500
C	2.02286400	-0.27907300	-0.25720900
C	1.74037700	-1.76430600	-0.27253600
H	0.68331500	-1.95017600	-0.04710000
H	2.32639500	-2.29184300	0.49398200
H	1.97581200	-2.21000400	-1.24351200
C	1.62714600	0.41213900	1.02690900
H	1.84445900	1.48506700	0.99745600
H	0.55674300	0.27928400	1.22845100
H	2.16588600	-0.02116100	1.88365900
C	-3.37471900	0.40476500	0.08680500
C	-2.91411700	-0.72358000	-0.61308300
C	-0.90989800	0.53254500	-1.20982400
O	0.26190500	0.57419300	-1.86110500
H	1.14267600	0.17779700	-1.13328500
C	-3.73436600	-1.99164800	-0.65628100
H	-4.21119600	-2.08962300	-1.63972500
H	-3.07495400	-2.85806200	-0.54291200
C	-4.79418900	-1.98050400	0.44087300
H	-4.32421800	-2.14584500	1.41808300
H	-5.52727100	-2.77844200	0.28546500
C	-5.51645000	-0.63314800	0.48709500
C	-6.50060100	-0.57944000	1.64396600
H	-6.94474200	0.41767500	1.71952200
H	-7.30302800	-1.30659800	1.48715700
H	-5.99706200	-0.81050300	2.58742900
C	-6.20878900	-0.29700900	-0.83254400
H	-6.91147500	-1.09146000	-1.10412200
H	-6.76326400	0.64090200	-0.73350000
H	-5.48795200	-0.18102500	-1.64725100
O	-4.55181600	0.40626200	0.77400300
C	-2.62071000	1.59553100	0.14895800
C	-1.37758800	1.65877600	-0.48684400
C	-1.68266000	-0.63699000	-1.25179800

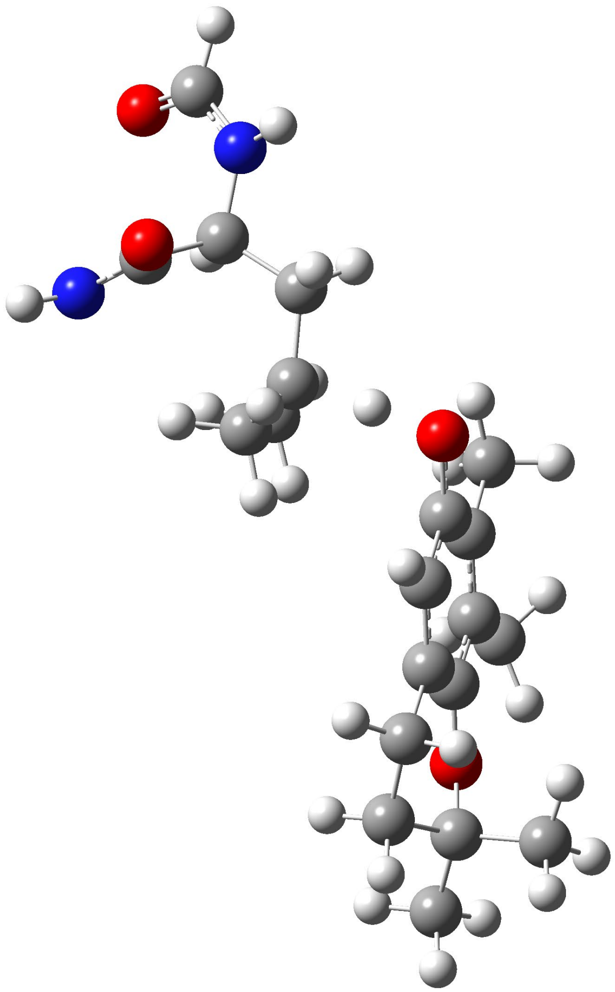

H	-1.29440100	-1.49119300	-1.80270600
C	-3.15523600	2.77417400	0.92076200
H	-2.87573400	3.71688900	0.44397000
H	-4.24241500	2.73074200	0.99625800
H	-2.75460800	2.79094900	1.94219300
C	-0.53526700	2.90343600	-0.40798500
H	0.43661900	2.73865800	-0.87485000
H	-1.01880200	3.74155100	-0.92356900
H	-0.38020400	3.21049200	0.63215800

6-δ-TS

Charge=0, Multiplicity=2

N	-2.59760700	-1.87051500	0.45708100
H	-1.93872300	-1.15401900	0.74821500
H	-2.33827800	-2.84540300	0.52577200
C	-3.86142100	-1.55924800	0.11154100
O	-4.71472300	-2.39073200	0.17852100
C	-4.16851300	-0.05191200	0.03575300
H	-3.43231200	0.50136100	-0.62946700
N	-5.48024800	0.21066600	-0.59529400
H	-6.27487000	0.24592300	0.03129400
C	-5.72247800	0.05003600	-1.91455600
O	-4.85944000	-0.19908900	2.74450100
H	-6.78255500	0.18660700	-2.18303200
C	-4.15301100	0.41533300	1.42468700
H	-4.87020200	-0.19906900	1.98410000
H	-4.51713400	1.45103300	1.45095500
C	-2.78691500	0.35795200	2.11615300
H	-2.37748400	-0.65842200	2.03473700
C	-2.95698700	0.67226100	3.61363200
H	-3.36720500	1.67916100	3.75106100
H	-1.99810600	0.61974700	4.13700300
H	-3.64208600	-0.04450500	4.07858100
C	-1.80343200	1.33584300	1.52054000
H	-2.20017800	2.33461700	1.31200600
H	-0.81854800	1.35722300	1.99740200
C	3.20585500	0.34225200	-0.36773600
C	2.45098200	-0.84031400	-0.29101100
C	0.43904800	0.49407700	-0.57914900
O	-0.90803700	0.53612900	-0.70924100
H	-1.38937300	0.92206100	0.27958400
C	3.12954800	-2.17954600	-0.12287400
H	3.00558200	-2.52996000	0.90961500
H	2.64107800	-2.91977400	-0.76431000
C	4.61195400	-2.07408300	-0.46614000
H	4.73941900	-1.98318500	-1.55162900
H	5.15346000	-2.96810700	-0.14115600
C	5.23614600	-0.84010300	0.18648800
C	6.68647800	-0.67254700	-0.23537100
H	7.09381800	0.26075400	0.16486600
H	7.28567100	-1.50507100	0.14548000
H	6.76903000	-0.65094400	-1.32607700
C	5.11078600	-0.85843500	1.70882400
H	5.56821500	-1.76634300	2.11507800
H	5.62128400	0.01035600	2.13489200

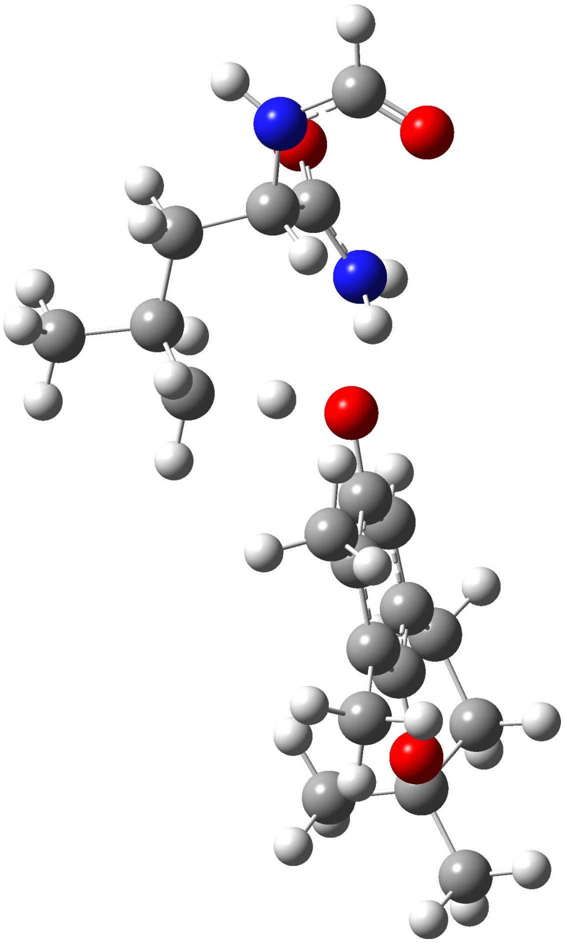

H	4.06452100	-0.82769000	2.02706400
O	4.56713900	0.34215700	-0.31210400
C	2.59290300	1.60105000	-0.54075400
C	1.20211300	1.67917700	-0.66161700
C	1.06687300	-0.73856900	-0.38013000
H	0.45434800	-1.63615300	-0.32030400
C	3.44205200	2.84377800	-0.61051000
H	2.95967700	3.67832800	-0.09480800
H	4.41997200	2.67397900	-0.15930300
H	3.60576500	3.15092100	-1.65087900
C	0.51430500	3.00294800	-0.86259000
H	-0.52012200	2.85573900	-1.17643700
H	0.50872800	3.59170900	0.06330900
H	1.02436700	3.60065000	-1.62395000

7-β-TS

Charge=0, Multiplicity=2

C	-2.43494400	-0.59891200	-0.18077800
C	-1.83324000	0.12274400	0.85996200
C	0.06475300	-1.38083100	0.71588600
O	1.29491100	-1.74355100	1.13811000
H	2.09210000	-1.23310400	0.48673600
C	-2.51162500	1.34439700	1.43197200
H	-2.88700900	1.12892900	2.44171100
H	-1.77486700	2.14788700	1.53681600
C	-3.64931000	1.80776700	0.52705100
H	-3.23779900	2.30317400	-0.36142300
H	-4.28717200	2.53286300	1.04252700
C	-4.49834800	0.62817300	0.05704800
C	-5.56458300	1.07448700	-0.92950500
H	-6.10375700	0.20816400	-1.32460200
H	-6.28241400	1.73434900	-0.43299100
H	-5.11126300	1.61566900	-1.76544300
C	-5.12113200	-0.14202500	1.22008100
H	-5.73807200	0.52663900	1.82908300
H	-5.75388000	-0.94796500	0.83643000
H	-4.35609800	-0.58616000	1.86376400
O	-3.65661500	-0.27791200	0.69223800
C	-1.80163500	-1.69649700	0.79945700
C	-0.54104300	-2.08975500	0.35023400
C	-0.57315100	-0.28029600	1.31888800
C	0.11326900	0.49798400	2.40824600
H	0.35467300	1.51014500	2.05881400
H	-0.53420700	0.60434200	3.28571600
H	1.03511500	0.00291100	2.71611800
C	-2.52604400	-2.39171700	1.92274900
H	-2.80462300	-1.67982500	2.70633700
H	-1.91915900	-3.17715800	2.37346600
H	-3.45854500	-2.84126400	1.56421500
C	0.19757400	-3.23821100	-0.99098900
H	1.12674600	-3.44137700	-0.45799000
H	-0.40483300	-4.15224900	0.98459600
H	0.44244100	-3.02013700	-2.03737300
N	4.19976000	2.57758400	-0.36344800
H	3.91383100	2.74789900	0.58990500

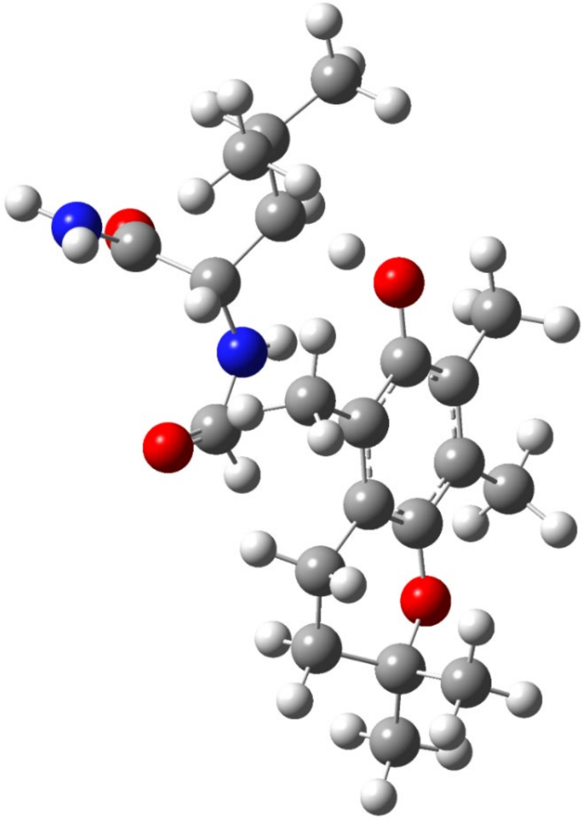

H	4.90000700	3.18325800	-0.77124300
C	3.51896100	1.72853800	-1.16150600
O	3.73657000	1.60010300	-2.35847600
C	2.47152000	0.84585200	-0.44504700
H	2.22737100	1.28605000	0.52837900
N	1.24956500	0.80838500	-1.22098900
H	1.15031900	0.11381700	-1.95062900
C	0.26877100	1.72027700	-1.06112700
O	0.30930300	2.64908400	-0.26208400
H	-0.59101300	1.54907100	-1.72861200
C	3.04143800	-0.54867200	-0.27847300
H	3.02635100	-1.11771900	-1.21594000
C	4.31744500	-0.72471500	0.51887700
H	5.10697700	-0.13786300	0.01766000
C	4.74283700	-2.19428900	0.50597900
H	3.98608200	-2.81701000	0.99707800
H	5.68893400	-2.32833000	1.03938700
H	4.87417000	-2.56003200	-0.51749600
C	4.18882700	-0.21667700	1.95891400
H	3.90757700	0.84027100	2.00911100
H	5.14137300	-0.32967800	2.48576400
H	3.42996200	-0.79486600	2.49769400

7-γ-TS

Charge=0, Multiplicity=2

C	-3.36392400	0.47545900	0.14568300
C	-2.85867100	-0.70005700	0.43382800
C	-0.87205500	0.53873500	-1.09319600
O	0.31389000	0.58096500	-1.72153800
H	1.19350700	0.29814200	-0.95556400
C	-3.65713700	-1.98223400	0.37524100
H	-4.06198300	-2.21098600	1.37074300
H	-2.99764300	-2.81774300	0.11811300
C	-4.78771400	-1.88405000	0.64445900
H	-4.38197000	-1.95806400	1.66076400
H	-5.50285000	-2.70233500	0.51233600
C	-5.51551100	-0.54644800	0.52863100
C	-6.56957300	-0.39840700	1.61343700
H	-7.02116500	0.59740900	1.57240500
H	-7.35828000	-1.14391900	1.47390300
H	-6.12421500	-0.53992800	2.60273200
C	-6.12537900	-0.33133600	0.85550200
H	-6.81837100	-1.14486800	1.09329800
H	-6.67616400	0.61379500	-0.87691700
H	-5.35687900	-0.29478200	1.63331400
O	-4.56832700	0.51414200	0.78562500
C	-2.64147500	1.68499500	0.12283700
C	-1.38683000	1.71286600	-0.48757800
C	-1.61046400	-0.66596100	1.06660000
C	-1.06491200	-1.90475400	1.72523400
H	-0.86999200	-2.69614100	0.99194100
H	-1.78152200	-2.30908700	2.44915300
H	-0.13351800	-1.68418700	2.24721400
C	-3.21702500	2.92327300	0.76033600
H	-4.29243100	2.82412500	0.90873500

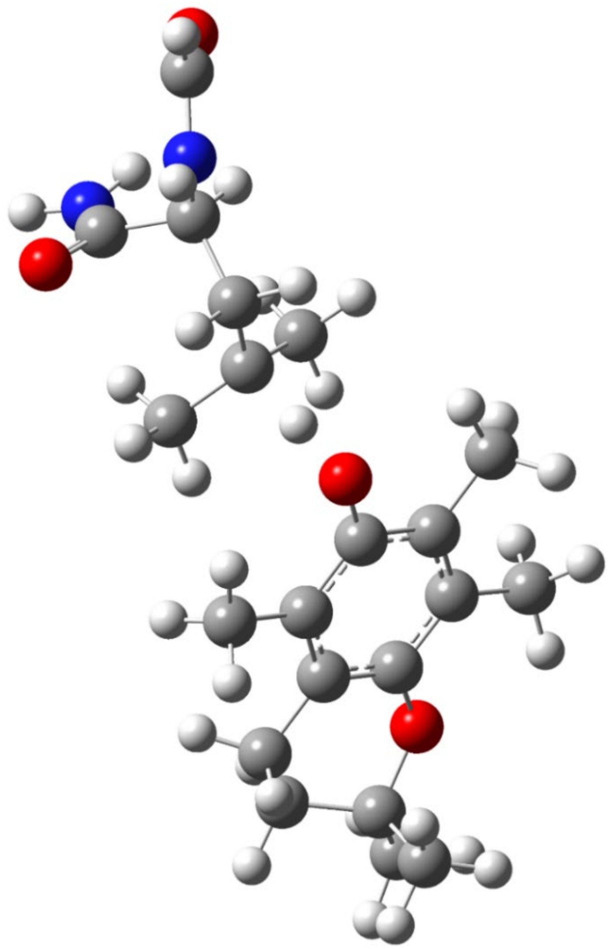

H	-2.76148900	3.11095600	1.74076100
H	-3.03053000	3.80601800	0.14225900
C	-0.59080200	2.99034900	-0.52688500
H	-1.10781700	3.75485500	-1.11895300
H	-0.45196900	3.40290800	0.47871200
H	0.38817700	2.82184500	-0.97566400
N	4.83585600	-1.17034000	2.10462500
H	4.96326500	-0.27610800	2.55652500
H	4.98856200	-2.00736800	2.65233900
C	4.80192400	-1.26882800	0.75797600
O	4.84413100	-2.33435000	0.15580100
C	4.66659500	0.06045300	0.00144900
H	4.66626200	0.89605100	0.70750000
N	5.81668100	0.23637700	-0.86749600
H	5.79948600	-0.18561100	-1.78823800
C	6.98362600	0.74225200	-0.41298700
O	7.16272100	1.15159500	0.72673600
H	7.77186600	0.76903500	-1.18213100
C	3.38026500	0.07681500	-0.83973800
H	3.42397900	-0.75346400	-1.55515100
H	3.38953600	1.01569900	-1.40798300
C	2.10008200	-0.00949600	-0.02838900
C	1.64925800	-1.38007800	0.42227500
H	0.58870400	-1.35529600	0.70380400
H	2.20568500	-1.70928800	1.31212000
H	1.78895200	-2.13144300	-0.36006700
C	1.91175400	1.07004000	1.01198800
H	2.22961100	2.05194000	0.64616300
H	0.86023300	1.13525500	1.31780700
H	2.49169500	0.83999000	1.91931900

7-δ-TS

Charge=0, Multiplicity=2

C	3.22750500	-0.44587100	-0.00122300
C	2.63640100	0.82582900	0.07082000
C	0.48181000	-0.22429400	-0.25275000
O	-0.85688500	-0.12226400	0.43471500
H	-1.39933700	-0.01615500	0.58103800
C	3.48791300	2.05245400	0.30405200
H	3.54407100	2.64555600	-0.61891100
H	3.00943300	2.69633800	1.04957000
C	4.88828700	1.67008600	0.77243300
H	4.85774800	1.35141600	1.82142600
H	5.56822100	2.52533000	0.70513400
C	5.44310500	0.51045300	-0.05207200
C	6.79247900	0.05473100	0.47805300
H	7.13519100	-0.83141200	-0.06464600
H	7.53324600	0.84965600	0.34927800
H	6.72366800	-0.19166900	1.54181500
C	5.52646200	0.83896100	-1.54164700
H	6.14957900	1.72530100	-1.69875700
H	5.97164200	-0.00087000	-2.08343100
H	4.53777800	1.03316200	-1.96775100
O	4.57323500	-0.63103600	0.12028600
C	2.46160800	-1.61870900	-0.16906200
C	1.07364200	-1.50554300	-0.25747600
C	1.24795600	0.94003300	-0.07357800
C	0.57897200	2.28716400	-0.01731000
H	0.56520700	2.67716200	1.00810000
H	1.11070800	3.01919600	-0.63328100
H	-0.45250600	2.22568900	-0.36700300
C	3.14794600	-2.95979800	-0.24333200
H	3.08963200	-3.49293800	0.71349800
H	2.68668000	-3.59543100	-1.00385600
H	4.20319600	-2.84003300	-0.48920200
C	0.18712000	-2.71805200	-0.37684500
H	-0.76556500	-2.55157300	0.13401600
H	-0.03836800	-2.94785100	1.42634900
H	0.65539200	-3.60042900	0.06305500
N	-3.08798500	-1.67673100	1.46304000
H	-2.18742400	-1.26338400	1.23253100
H	-3.12078100	-2.56014800	1.95492500
C	-4.23975300	-1.02335800	1.23262300
O	-5.34346400	-1.43682300	1.58239800
C	-4.12143600	0.28189200	-0.44427400
H	-3.13632100	0.73612400	-0.59259100
N	-5.11897900	1.19027300	-0.97921600
H	-5.94322300	0.78097100	-1.40511900
C	-5.01941600	2.52570700	-0.84502200
O	-4.08484700	3.10261800	-0.30023200
H	-5.87090600	3.06803200	-1.28680000
C	-4.37579900	0.03271800	1.05619700
H	-5.33739600	-0.48661900	1.15130400
H	-4.48661500	1.01703600	1.52835700
C	-3.28680500	-0.74740000	1.79918900
H	-3.13375600	-1.72044300	1.31119700

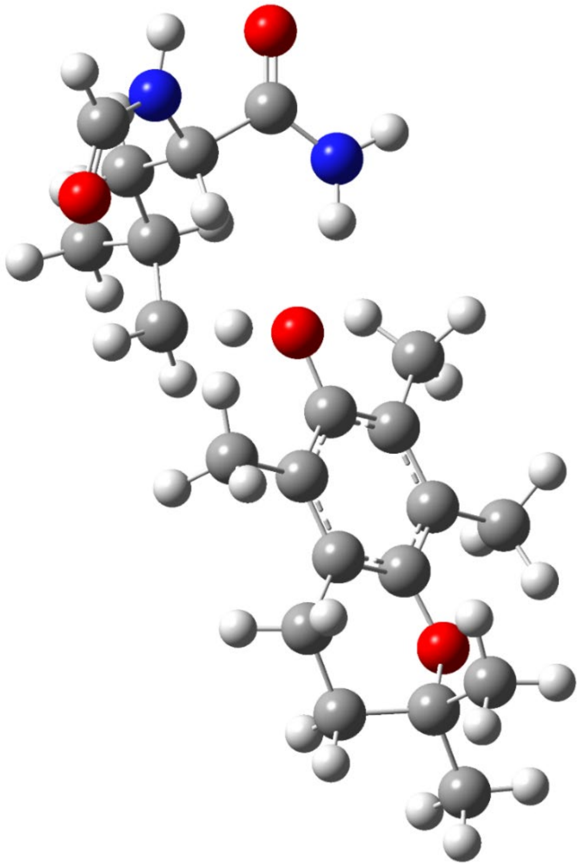

C	-3.74745400	-1.02186200	3.24265200
H	-3.91560700	-0.08002900	3.77675600
H	-2.99636100	-1.59705900	3.79182700
H	-4.68405300	-1.58952300	3.24350100
C	-1.97641700	0.00058600	1.83531900
H	-2.04818900	1.07433900	2.03580100
H	-1.16529500	-0.48888700	2.38272300

8-β-TS

Charge=0, Multiplicity=2

N	-2.96203300	-0.18198600	2.02609000
H	-2.11267900	-0.48229600	2.48292700
H	-3.81436400	-0.18344700	2.57162800
C	-3.06693800	-0.21264300	0.67902500
O	-4.12634800	-0.11612500	0.07820000
C	-1.72004500	-0.27620500	-0.08291900
H	-0.93797800	-0.62803700	0.60211400
N	-1.82358800	-1.21703200	-1.17750600
H	-1.91661500	-0.87361900	-2.12505400
C	-1.96645100	-2.53601800	-0.94176000
O	-1.95819700	-3.03014200	0.17938300
H	-2.07785000	-3.13782300	-1.85723900
C	-1.39011400	1.10280000	-0.60931400
H	-2.05149700	1.39513100	-1.43457900
C	-1.14510400	2.22412800	0.37887000
H	-2.10853000	2.42851800	0.87812900
C	-0.72781700	3.49359000	-0.36715400
H	0.24232900	3.34752200	-0.85646200
H	-0.63627400	4.33628700	0.32488100
H	-1.45879700	3.76185800	-1.13676300
C	-0.11886200	1.87750600	1.46237700
H	-0.41911300	1.01621200	2.06607300
H	0.00426300	2.72655700	2.14210500
H	0.85894300	1.65568300	1.02101900
C	1.70714600	0.19633300	-1.13606600
C	1.67647100	-1.09331400	-0.57807900
C	2.69488300	-1.52789500	0.25741200
C	3.78719500	-0.69057000	0.54997700
C	3.82131000	0.59843200	-0.01560000
C	2.79284900	1.03614900	-0.83553900
H	0.83853400	-1.74533000	-0.81156400
H	2.65776700	-2.52505200	0.68844800
H	4.66142100	1.25258000	0.20324500
H	2.81329500	2.03496000	-1.26256000
O	0.72205200	0.62613200	-1.94688900
H	-0.22692600	0.88977900	-1.33008300
N	4.78096100	-1.10538100	1.42183000
H	4.88061400	-2.10510200	1.54072400
H	5.66886600	-0.62627200	1.34718200

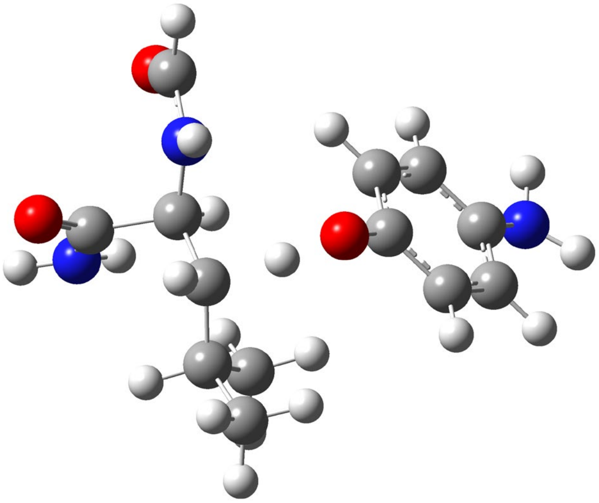

8-γ-TS

Charge=0, Multiplicity=2

N	4.41878400	0.01177000	-0.35934400
H	4.56672500	0.58976600	0.46052700
H	5.20629500	-0.18153900	-0.96456400

C	3.16939100	-0.14032100	-0.85415600
O	2.92601400	-0.62806500	-1.94969200
C	2.06145300	0.39510000	0.07497400
H	2.38017100	0.27299900	1.11566800
N	1.94542600	1.83160800	-0.15655500
H	1.27563300	2.16569500	-0.83961000
C	2.73891400	2.72487000	0.46571200
O	3.62812700	2.42378000	1.25723600
H	2.51244100	3.76952500	0.20430800
C	0.71723000	-0.30325000	-0.16074200
H	0.56640400	-0.40556300	-1.24224900
H	-0.07049300	0.34973900	0.23653000
C	0.61885600	-1.65781000	0.51632100
C	1.51252800	-2.75292400	-0.01819400
H	1.21555600	-3.72531700	0.38872000
H	2.55886900	-2.58326200	0.28227000
H	1.48687000	-2.79933200	-1.10996500
C	0.51454900	-1.61056500	2.02409100
H	-0.19838000	-0.84701200	2.35404900
H	0.19757600	-2.57922200	2.42445200
H	1.48846700	-1.37635700	2.48010900
C	-2.46305000	-1.19332700	-0.16097300
C	-3.05295000	-0.72408200	1.02716200
C	-3.82767600	0.42638700	1.03036700
C	-4.03868600	1.15113800	-0.15704300
C	-3.44787500	0.68533800	-1.34635400
C	-2.66847800	-0.46164400	-1.34534600
H	-2.89519100	-1.28586700	1.94394600
H	-4.28683600	0.77284900	1.95268500
H	-3.61249500	1.23316000	-2.27085900
H	-2.20917500	-0.81876500	-2.26306500
O	-1.72547500	-2.31338800	-0.16767400
H	-0.59389300	-2.05114300	0.14730700
N	-4.86372900	2.26594600	-0.16685700
H	-4.99259500	2.73256800	0.72136300
H	-4.73386700	2.90974600	-0.93621800

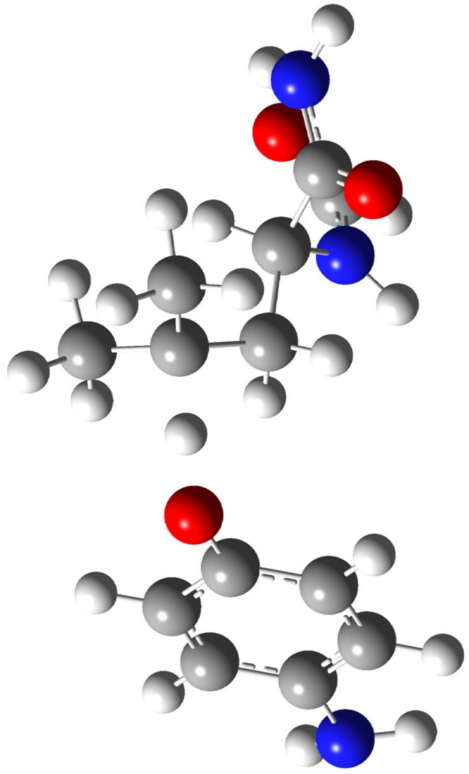

8-δ-TS

Charge=0, Multiplicity=2

N	-1.15583700	-0.91737400	1.54130200
H	-0.46293700	-1.07372300	0.81533100
H	-0.96792700	-1.22786100	2.48522500
C	-2.38672900	-0.46082400	1.24135500
O	-3.28596100	-0.32857700	2.06442800
C	-2.59097500	-0.03909800	-0.22605800
H	-1.82313700	-0.50833200	-0.85129500
N	-3.88670700	-0.49645600	-0.69027000
H	-4.66770500	0.14718300	-0.65783600
C	-4.15732800	-1.81394500	-0.81528500

O	-3.32601500	-2.69968000	-0.67324900
H	-5.20670300	-2.02094000	-1.08049400
C	-2.53483400	1.48809900	-0.34753600
H	-3.27974200	1.90941400	0.33964400
H	-2.83928500	1.75715300	-1.36783400
C	-1.16643300	2.11656400	-0.06438300
H	-0.81315600	1.79376600	0.92524600
C	-1.30369300	3.64967100	-0.02967900
H	-1.66133900	4.02518000	-0.99513600
H	-0.34266000	4.12487400	0.18660900
H	-2.01838500	3.95170300	0.74318700
C	-0.13929300	1.73962600	-1.10363700
H	-0.48716100	1.75430800	-2.14099100
H	0.84699000	2.19600500	-0.97497800
C	2.02648100	-0.50428800	-0.42660600
C	3.00045600	-0.72756200	-1.41103000
C	4.34929500	-0.64738000	-1.09918100
C	4.76492700	-0.32077100	0.20494000
C	3.78560900	-0.08196000	1.18656200
C	2.43730400	-0.17025500	0.87338900
H	2.67895500	-0.97582000	-2.41833100
H	5.09805000	-0.83620500	-1.86395700
H	4.09601700	0.17024900	2.19721800
H	1.68383300	0.02007500	1.63389900
O	0.71247800	-0.61805200	-0.72979800
H	0.25907400	0.42882900	-0.93694200
N	6.11110200	-0.28920100	0.52945600
H	6.75324200	-0.15707100	-0.24095100
H	6.36132800	0.24562400	1.35081200

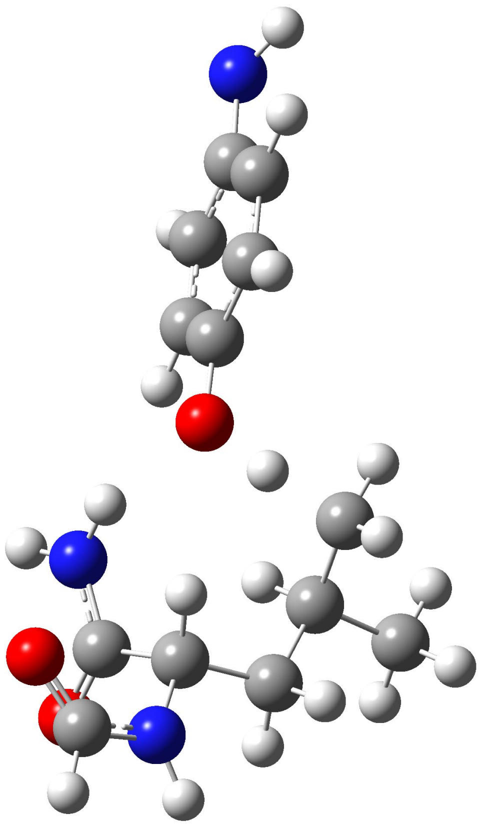

9-β-TS

Charge=0, Multiplicity=2

N	0.30667000	-1.64889000	1.12852600
H	0.32251100	-2.15738900	0.25266800
H	1.02449000	-1.82015500	1.81915200
C	-0.68761600	-0.78196500	1.40642300
O	-0.79445100	-0.17841700	2.46688600
C	-1.73997400	-0.65968800	0.28399900
H	-1.23057400	-0.71356500	-0.68640100
N	-2.59709200	-1.84860600	0.35919800
H	-3.42994200	-1.81579000	0.93507700
C	-2.29147000	-2.99513000	-0.27750600
O	-1.27991200	-3.16375200	-0.95297400
H	-3.04691600	-3.78412800	-0.14136500
C	-2.51854100	0.62512800	0.39007300
H	-2.91685400	0.81674500	1.39178200
C	-3.40641000	1.06187300	-0.75645100
H	-2.80786200	1.03230500	-1.67864500
C	-3.87570500	2.50111400	-0.52452700
H	-4.49407400	2.55756500	0.37961600
H	-4.47957700	2.85191100	-1.36734000
H	-3.02925600	3.18300600	-0.40167300
C	-4.62670400	0.14525100	-0.95877200
H	-4.33848600	-0.85880000	-1.27834300
H	-5.28404400	0.56953900	-1.72567800

H	-5.20523600	0.06238100	-0.03038100
C	0.54572800	1.67838800	-0.05224400
C	0.93372200	1.05164300	-1.24793000
C	2.16098500	0.41701600	-1.36128300
C	3.06416700	0.38115900	-0.26991500
C	2.67557300	1.03891700	0.92515800
C	1.44153200	1.65784200	1.02958200
H	0.25389700	1.07633400	-2.09619700
H	2.42144700	-0.04726700	-2.30461900
H	3.34095000	1.05957600	1.77990300
H	1.14567000	2.13781300	1.95755200
N	4.27091700	-0.27340800	-0.36368400
O	-0.63968600	2.30788100	0.04678600
H	-1.48756900	1.57788500	0.30695100
C	5.26009600	-0.09025200	0.68340000
H	6.14150500	-0.68491700	0.44412200
H	5.56418000	0.96153100	0.78706000
H	4.87624400	-0.43553300	1.64835500
C	4.72410800	-0.74256800	-1.66083300
H	5.68463900	-1.24283100	-1.53914000
H	4.02085500	-1.46829900	-2.08109000
H	4.84597600	0.08034300	-2.38028200

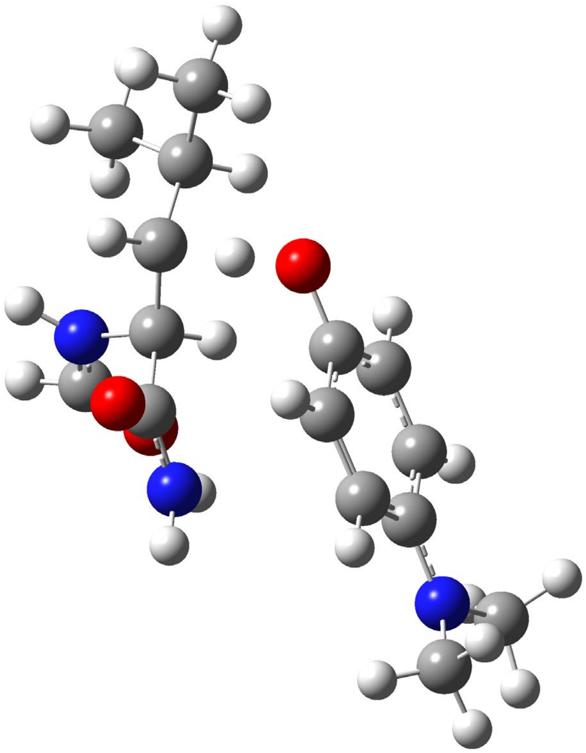

9-γ-TS

Charge=0, Multiplicity=2

N	3.95019700	1.39032300	-1.74734000
H	3.96943800	0.62646000	-2.40847600
H	4.25218000	2.30082800	-2.07028400
C	3.95108600	1.14534200	-0.41794400
O	4.17737900	2.00133100	0.42761800
C	3.60267500	-0.29438000	-0.01129600
H	3.41528200	-0.90070200	-0.90276000
N	4.73721600	-0.88328400	0.67880700
H	4.80336500	-0.78571100	1.68484400
C	5.80588200	-1.35416400	0.00153900
O	5.88642100	-1.38305500	-1.22012600
H	6.60661300	-1.73056700	0.65752400
C	2.37186100	-0.31700800	0.90786200
H	2.59272400	0.28618300	1.79668000
H	2.23804400	-1.35831500	1.22776600
C	1.09508100	0.17735500	0.25206200
C	0.87455000	1.67099000	0.19188600
H	-0.17527400	1.89031000	-0.03757000
H	1.47667000	2.13368900	-0.60419300
H	1.13665700	2.15688500	1.13639200
C	0.67084800	-0.56425300	-0.99285800
H	0.78315000	-1.64822000	-0.87910300
H	-0.37563900	-0.34755700	-1.23823100
H	1.27148700	-0.24971800	-1.86070000
C	-1.88220500	-0.41758300	1.26439600
C	-2.58904800	0.79679100	1.31832000
C	-3.82575900	0.93753900	0.70829000

C	-4.42384600	-0.14262100	0.01310600
C	-3.69694100	-1.35722700	-0.05754700
C	-2.45965300	-1.48459400	0.55341600
H	-2.14737000	1.63253000	1.85474200
H	-4.33381000	1.89180000	0.77753900
H	-4.10290600	-2.20763800	-0.59184600
H	-1.91492400	-2.42301000	0.49104600
N	-5.66759900	-0.01928200	-0.56424100
O	-0.69812500	-0.55790100	1.87837900
H	0.17908200	-0.21170400	1.15192900
C	-6.14624700	-1.06649100	-1.44772200
H	-7.14416000	-0.80293600	-1.79768300
H	-5.49338900	-1.20434300	-2.32228200
H	-6.22031500	-2.02122700	-0.91764600
C	-6.27254900	1.29588800	-0.66943500
H	-7.24845600	1.19776800	-1.14458200
H	-6.42642000	1.73596200	0.32085000
H	-5.66053000	1.98843500	-1.26596700

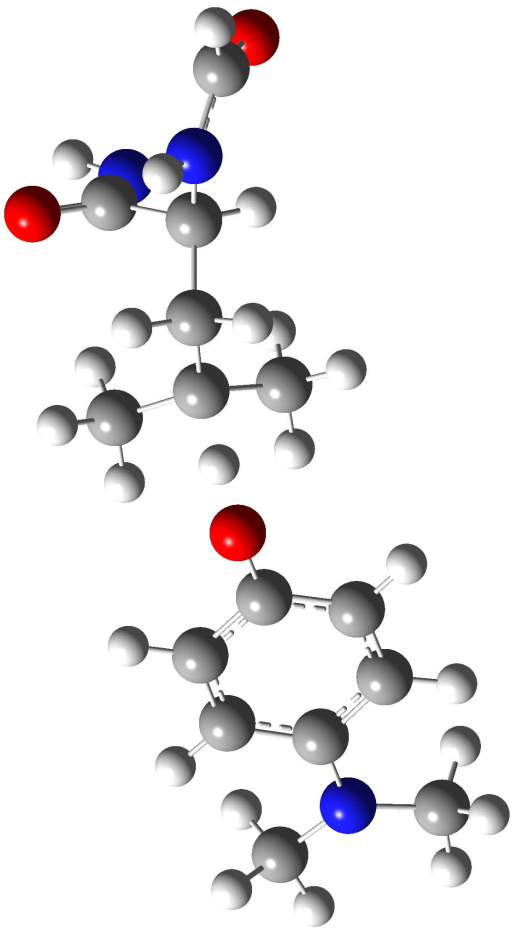

9-δ-TS

Charge=0, Multiplicity=2

N	-1.81438100	-0.77912600	1.62164700
H	-1.12354500	-0.96903700	0.90150300
H	-1.60451300	-0.99661000	2.58668800
C	-3.05922400	-0.37799200	1.30198500
O	-3.95212100	-0.19901400	2.12343800
C	-3.29047700	-0.08661400	-0.19266900
H	-2.51171700	-0.57901300	-0.78545800
N	-4.57212400	-0.62718100	-0.60390000
H	-5.37758900	-0.01375800	-0.61386400
C	-4.78952400	-1.95990500	-0.63452400
O	-3.92181900	-2.79994000	-0.44078200
H	-5.83187700	-2.22665400	-0.87291600
C	-3.28811500	1.42646600	-0.43884000
H	-4.04348000	1.87725900	0.21750300
H	-3.60606500	1.60113700	-1.47540500
C	-1.93999500	2.12030300	-0.21626400
H	-1.56914000	1.88283700	0.79101500
C	-2.12975400	3.64650200	-0.29047700
H	-2.50910000	3.93907000	-1.27611500
H	-1.18340200	4.16790700	-0.12082500
H	-2.84682700	3.97990600	0.46723600
C	-0.90805100	1.70224000	-1.23421100

H	-1.26029300	1.63423400	-2.26786400
H	0.06830500	2.18745900	-1.14187600
C	1.31597000	-0.48904400	-0.47136500
C	1.78807200	-0.03907400	0.76992100
C	3.14749100	0.05987300	1.02424100
C	4.10064700	-0.29720000	0.03814500
C	3.61099900	-0.72309300	-1.22178400
C	2.24931700	-0.81000900	-1.46609300
H	1.07101200	0.23898400	1.53902000
H	3.47039100	0.41322000	1.99591100
H	4.29921300	-0.98730800	-2.01529200
H	1.88772800	-1.14022300	-2.43579200
N	5.44913800	-0.23574200	0.29521100
O	-0.01088000	-0.61648000	-0.70821000
H	-0.46993800	0.39917400	-0.97757600
C	6.39226300	-0.42661700	-0.79102500
H	7.40520900	-0.35824300	-0.39503900
H	6.27504600	-1.41770300	-1.24148000
H	6.27405500	0.32967500	-1.58054000
C	5.91418400	0.38916100	1.51948100
H	7.00234800	0.34163800	1.54840100
H	5.60914000	1.44337000	1.58825100
H	5.53234900	-0.13948500	2.39911900

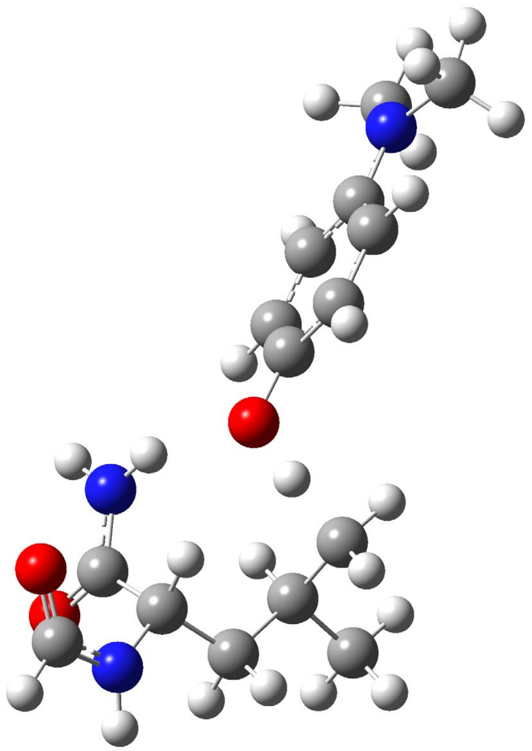

10-β-TS

Charge=0, Multiplicity=2

N	-3.31704100	-2.66744800	-1.00744000
H	-2.57220000	-3.08681900	-0.46057300
H	-3.91922400	-3.26702800	-1.55671100
C	-3.26534700	-1.35056500	-1.30804100
O	-4.00095200	-0.80538700	-2.11952200
C	-2.16349400	-0.58093900	-0.53482400
H	-2.10415500	-1.00639900	0.47597200
N	-0.87948600	-0.84393200	-1.17919800
H	-0.48337600	-0.13395500	-1.78394200
C	-0.12772700	-1.91924100	-0.87471400
O	-0.49250600	-2.82926000	-0.13341400
H	0.85567200	-1.91975900	-1.36942100
C	-2.43391300	0.90021000	-0.46550100
H	-2.14997900	1.43924300	-1.37647600
C	-3.73690000	1.37025700	0.14300200
H	-4.52799400	1.13790200	-0.58753500
C	-3.70699700	2.88699300	0.34857800
H	-2.95880900	3.16055500	1.10114000
H	-4.68173900	3.24918900	0.68993700
H	-3.45906200	3.40783100	-0.58261700
C	-4.06734200	0.64721200	1.45104900
H	-4.20456500	-0.43005700	1.30595500
H	-4.99558200	1.04126500	1.87628500
H	-3.26774600	0.79662500	2.18671200
C	0.79928500	-0.14859100	1.63783300
C	2.00029200	-0.79403300	1.34309400
C	2.87156600	-0.24712600	0.36917200
C	2.55919700	0.96845100	-0.27913600

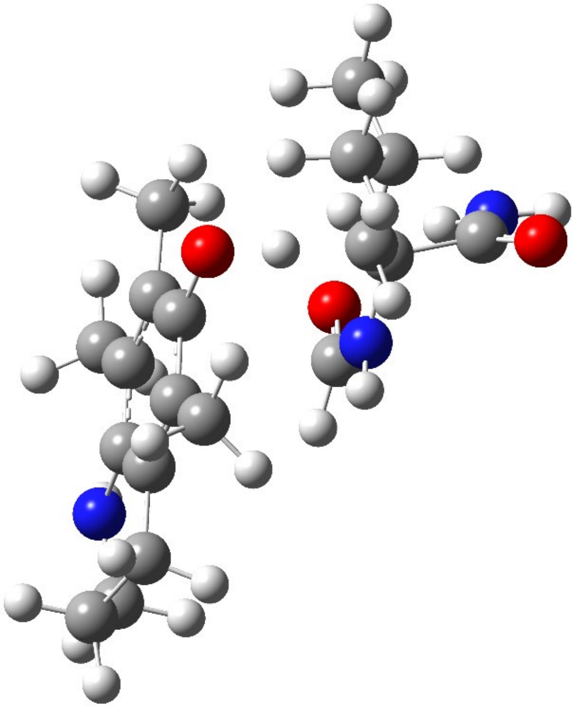

C	1.35626600	1.61506600	0.01965300
C	0.48042500	1.05881800	0.97432700
C	0.98053900	2.88615600	-0.69707100
H	1.66878500	3.70145700	-0.44436100
H	1.02664800	2.75763700	-1.78484100
H	-0.02635300	3.20463600	-0.42491000
C	2.38871200	-2.09113000	2.00415100
H	2.38536200	-2.92225200	1.28538000
H	3.39671700	-2.02969700	2.43210400
H	1.70419100	-2.36069500	2.80758600
C	-0.18149800	-0.73821000	2.61967900
H	-0.47890300	-1.74689400	2.31202500
H	0.25347000	-0.81215700	3.62261500
H	-1.07433000	-0.11561100	2.68691800
O	-0.69344900	1.67450800	1.23681900
H	-1.47087000	1.36864300	0.46454200
C	3.51468800	1.56742500	-1.29372300
H	3.10803300	1.43184200	-2.30625500
H	3.58300300	2.64947100	-1.13853100
C	4.90844800	0.94534600	-1.21943800
H	5.43519800	1.30728200	-0.32891700
H	5.49838100	1.22720200	-2.09630700
C	4.78877000	-0.56780600	-1.13075800
H	5.77224800	-1.04145700	-1.07908300
H	4.27430900	-0.94806700	-2.02909300
N	4.05923500	-0.90854300	0.08109000
H	4.07936100	-1.88913700	0.32617900

10-γ-TS

Charge=0, Multiplicity=2

N	-5.02333300	0.27394100	0.84234000
H	-4.74453700	0.28149500	1.81321100
H	-5.99402600	0.46502900	0.62865200
C	-4.26979100	-0.35798300	-0.08483700
O	-4.65986300	-0.60607900	-1.21854800
C	-2.83766500	-0.70400500	0.34999900
H	-2.70316400	-0.47421300	1.41151500
N	-2.60765200	-2.12691500	0.17797300
H	-2.21100700	-2.45654400	-0.69371100
C	-3.12327900	-3.03282400	1.03548200
O	-3.75179600	-2.74195200	2.04509700
H	-2.91150400	-4.07512700	0.74873800
C	-1.81889400	0.07708700	-0.49713000
H	-1.96000200	-0.20723900	-1.54719200
H	-0.82297200	-0.26914000	-0.18306400
C	-1.89158700	1.58499400	-0.35951600
C	-2.89183200	2.31254400	-1.22815000
H	-2.66768500	3.38466000	-1.25716900
H	-3.91459500	2.20695100	-0.83648500
H	-2.88634400	1.92841600	-2.25289300
C	-1.76376100	2.10908900	1.05140800
H	-0.93488100	1.62765300	1.58436600
H	-1.59811900	3.19121600	1.05516400
H	-2.68461400	1.92229000	1.62589300
C	1.95579400	1.88136000	0.40408000

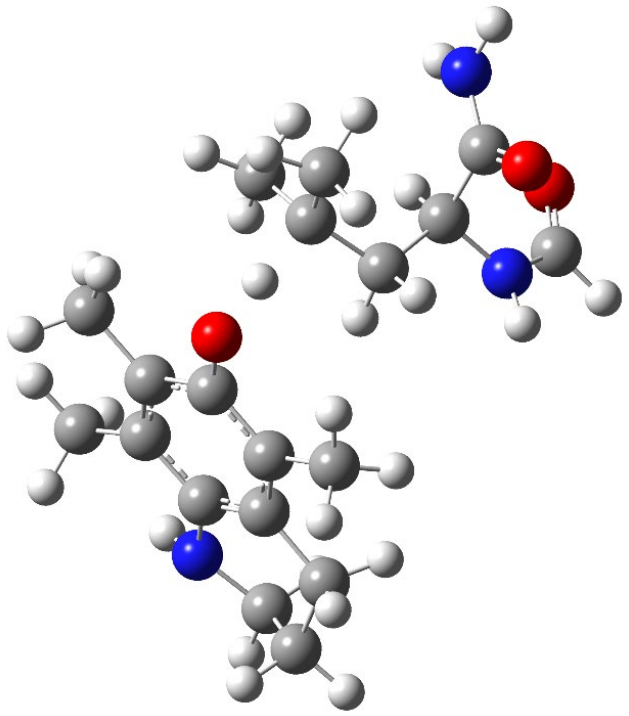

C	2.93808100	1.08059900	0.98749400
C	3.25759200	-0.17355600	0.41117200
C	2.62673900	-0.60406200	-0.77629400
C	1.64230700	0.19719100	-1.35967300
C	1.29916700	1.43645100	-0.77123000
C	0.94270200	-0.25570000	-2.61442100
H	1.63768000	-0.28721400	-3.46222500
H	0.53256800	-1.26579700	-2.49965600
H	0.13159200	0.42536600	-2.87298600
C	3.68805000	1.51027900	2.22312900
H	3.47154600	0.85409600	3.07704800
H	4.77147000	1.47542600	2.05504900
H	3.43286600	2.52585400	2.52395200
C	1.59455200	3.22532900	0.98676700
H	0.81280500	3.69761600	0.39230600
H	1.23890700	3.13631100	2.01908500
H	2.46195000	3.89457200	0.99749000
O	0.36081600	2.20784400	-1.34833900
H	-0.70487900	1.94817600	-0.92049100
C	3.00764600	-1.92769200	-1.41173300
H	2.21022500	-2.66281700	-1.23068400
H	3.06942500	-1.80913100	-2.49869100
C	4.33078500	-2.47186800	-0.87525400
H	5.16818600	-1.89322400	-1.28216600
H	4.46478400	-3.51437100	-1.17808800
C	4.35307800	-2.35851900	0.64073000
H	5.28926000	-2.74286400	1.05342900
H	3.52490300	-2.94949800	1.06512000
N	4.24233700	-0.95388500	1.00577600
H	4.40934900	-0.76071700	1.98404200

10-δ-TS

Charge=0, Multiplicity=2

N	2.64070400	1.54573500	-1.65112500
H	1.72280600	1.18550800	-1.40147600
H	2.71140900	2.36413400	-2.24153800
C	3.76812800	0.89386500	-1.31935400
O	4.89047800	1.24055800	-1.68276000
C	3.59836400	-0.31607400	-0.39907900
H	2.60060500	-0.75306100	-0.51246700
N	4.57450000	-1.30404000	-0.82192700
H	5.41646100	-0.96341400	-1.27281600
C	4.44083100	-2.61434300	-0.54505700
O	3.48626900	-3.10784900	0.04475000
H	5.28443200	-3.22051400	-0.91316900
C	3.84425800	0.08277000	1.06994200
H	4.82293700	0.57538900	1.12343000
H	3.91372800	-0.84885600	1.64556300
C	2.77569800	0.97497000	1.70953100
H	2.66042800	1.89366700	1.11626200
C	3.23497900	1.39199200	3.11911500
H	3.36713000	0.50957300	3.75513400
H	2.49937100	2.04811100	3.59341800
H	4.19011700	1.92571000	3.06841800
C	1.44080300	0.27973900	1.80804800

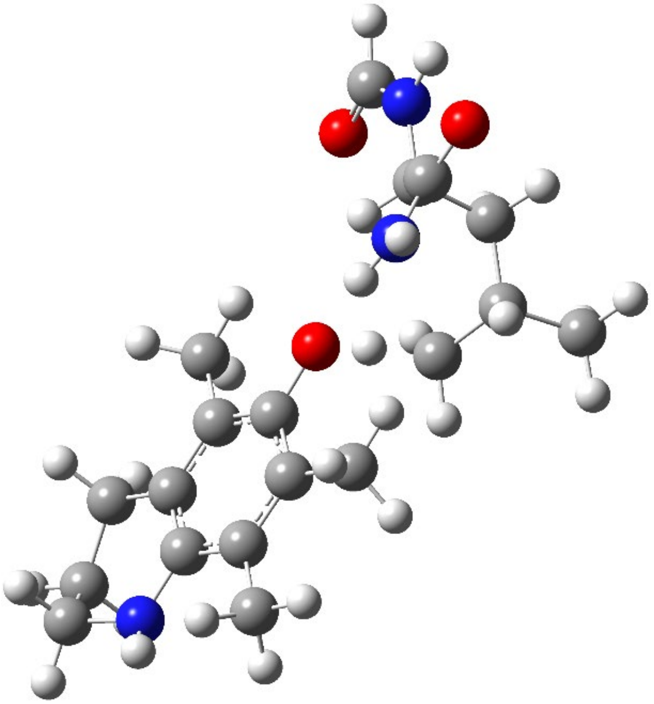

H	1.46866200	-0.77559100	2.09665100
H	0.64175700	0.84570300	2.29561400
C	-1.68778100	1.51007600	-0.30973600
C	-3.08074800	1.52383600	-0.21914900
C	-3.78901600	0.30173200	-0.11492000
C	-3.10253500	-0.93131600	-0.07055500
C	-1.70986600	-0.94550700	-0.18446300
C	-1.01291800	0.26974400	-0.32543200
C	-0.95367700	-2.24780000	-0.15377000
H	-0.95448200	-2.68005500	0.85450200
H	-1.40954900	-2.98662300	-0.82068400
H	0.08420600	-2.10492400	-0.45587700
C	-3.86993300	2.80831200	-0.22824000
H	-4.51769600	2.87387500	-1.11297600
H	-4.51773700	2.87968000	0.65420400
H	-3.22476500	3.68622200	-0.23803400
C	-0.89578000	2.79189400	-0.39143600
H	-1.10838400	3.33684700	-1.31818600
H	-1.13748300	3.45880900	0.44212700
H	0.17585200	2.59118500	-0.35764700
O	0.33531700	0.24207600	-0.48111000
H	0.85669100	0.21064400	0.52601800
C	-3.86524900	-2.23170200	0.09337600
H	-3.87827000	-2.76998000	-0.86486200
H	-3.33566500	-2.88074700	0.79896000
C	-5.29970800	-2.00979800	0.57087000
H	-5.30578700	-1.73403500	1.63184600
H	-5.88334300	-2.92819500	0.45996600
C	-5.93591100	-0.88327800	-0.22775300
H	-6.96536600	-0.70282200	0.09180500
H	-5.95215900	-1.15409200	-1.29597000
N	-5.17377200	0.33663700	-0.00287900
H	-5.61208100	1.17658400	-0.35536400

11-β-TS

Charge=0, Multiplicity=2

C	-1.85949300	1.23928900	2.14494200
H	-1.32586400	1.16847500	3.09788900
H	-1.50216900	2.14587800	1.63657700
C	-3.36666700	1.33840600	2.36112700
H	-3.62641200	2.23277200	2.93458100
H	-3.71880100	0.46487300	2.92267100
C	-4.06739100	1.39350600	1.01308000
H	-3.85666000	2.35520900	0.51903500
H	-5.15381200	1.32794500	1.14813000
C	-4.58859500	0.01043600	-0.94926800
H	-5.44298400	-0.56925000	-0.56631600
H	-4.98357600	0.96749900	-1.31160300
C	-3.91998700	-0.74279400	-2.08879600
H	-3.21321300	-0.08066300	-2.60353400
H	-4.68239500	-1.04354600	-2.81298800
C	-3.17585000	-1.95405400	-1.53220900
H	-2.67531800	-2.51424400	-2.32794800
H	-3.90050200	-2.63555300	-1.06544500
N	-3.66488400	0.29653900	0.14181000

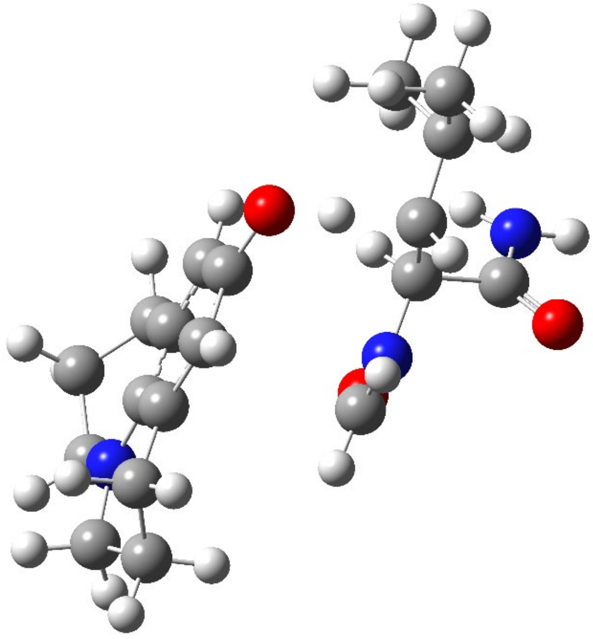

O	1.20578000	-2.30461700	0.67570700
H	1.97338200	-1.65571000	0.17278200
C	-0.34484000	-0.64689900	1.43342600
H	0.37047500	-0.32791100	2.18973000
C	-0.93490700	-2.12711800	-0.37508200
H	-0.67777500	-2.95826500	-1.02854300
C	-2.16487400	-1.49926400	-0.51022400
C	-2.47505600	-0.37630700	0.30481700
C	-1.54040200	0.03989600	1.29024400
C	-0.00627500	-1.71627100	0.59190900
N	3.61792900	2.49729200	-0.04226100
H	3.18612300	2.55111300	0.86931400
H	4.21935600	3.26023500	-0.32485100
C	3.11394900	1.68670000	-0.99998700
O	3.43461500	1.74921000	-2.17758500
C	2.16206600	0.58088700	-0.48368400
H	1.80200000	0.86423100	0.51427600
N	1.01276900	0.47632400	-1.35585800
H	0.90808500	-0.33068400	-1.95710200
C	0.03894700	1.40602500	-1.32996900
O	0.07712900	2.41648800	-0.63620500
H	-0.80185300	1.17963500	-2.00520000
C	2.91435400	-0.72948100	-0.43087900
H	3.05940400	-1.16882500	-1.42430700
C	4.12742300	-0.83482600	0.46877000
H	4.88097600	-0.11752800	0.09779300
C	4.72996900	-2.23822400	0.37323800
H	4.02287300	-2.98647500	0.74940700
H	5.64506200	-2.30765700	0.96945700
H	4.97796500	-2.49349700	-0.66207300
C	3.81993900	-0.48192000	1.92791300
H	3.44499200	0.54033800	2.04015800
H	4.72639900	-0.56760700	2.53529300
H	3.06950000	-1.16845500	2.33543300

11-γ-TS

Charge=0, Multiplicity=2

N	4.55314300	-0.26417300	2.26158600
H	4.65069300	0.73368200	2.38409600
H	4.71710600	-0.85834200	3.06422300
C	4.59230800	-0.81423600	1.02792300
O	4.70239700	-2.01701000	0.82656300
C	4.43714900	0.17137400	-0.13932500
H	4.40126600	1.19713400	0.24057900
N	5.59258500	0.07217700	-1.01274300
H	5.56884700	-0.58972000	-1.77908400
C	6.76962400	0.65332400	-0.69773400
O	6.94826600	1.35492700	0.28976100
H	7.56584700	0.44884000	-1.43080100
C	3.16227200	-0.13821200	-0.94223700
H	3.24358400	-1.15846700	-1.33685300
H	3.15448200	0.55461800	-1.79383100
C	1.87327000	0.00898200	-0.15688500
C	1.44013700	-1.15392700	0.70295800
H	0.38079000	-1.05470500	0.97121300

H	2.00443000	-1.19049800	1.64704700
H	1.58158800	-2.11046800	0.19030900
C	1.62796900	1.36671600	0.45528500
H	1.88258600	2.17671400	-0.23715300
H	0.57650000	1.47656100	0.74607300
H	2.22524800	1.50189400	1.37096400
C	-3.67922400	2.57427700	-0.19441000
H	-2.93397700	3.37021000	-0.10128200
H	-4.33664300	2.84499900	-1.03227600
C	-4.51769700	2.44893200	1.07505900
H	-5.05517600	3.37718800	1.28898000
H	-3.86365300	2.23517900	1.92879000
C	-5.52331600	1.32068900	0.90731700
H	-6.29935800	1.61379700	0.18238300
H	-6.03017000	1.12149600	1.85990300
C	-5.68222900	-1.12054500	0.69944500
H	-5.63000300	-1.40423900	1.76252500
H	-6.72972700	-0.87680400	0.48142600
C	-5.22312100	-2.28031500	-0.17040300
H	-5.46193200	-2.07131500	-1.21986100
H	-5.76583400	-3.18327300	0.12353800
C	-3.71562500	-2.46444300	-0.01626100
H	-3.35019800	-3.28768000	-0.63741100
H	-3.49186500	-2.72377600	1.02801000
N	-4.89046000	0.08204400	0.47432400
O	0.09478200	-0.03691600	-1.96244400
H	0.94863600	-0.02821900	-1.16458500
C	-1.73662400	1.21294100	-1.06574200
H	-1.22734200	2.13859700	-1.32688100
C	-1.76722400	-1.19754000	-1.00961900
H	-1.28213600	-2.14677600	-1.22825900
C	-3.00957600	-1.18587800	-0.39362100
C	-3.65001300	0.05335200	-0.12425500
C	-2.99297100	1.26144800	-0.47981500
C	-1.10411300	-0.00776600	-1.35684800

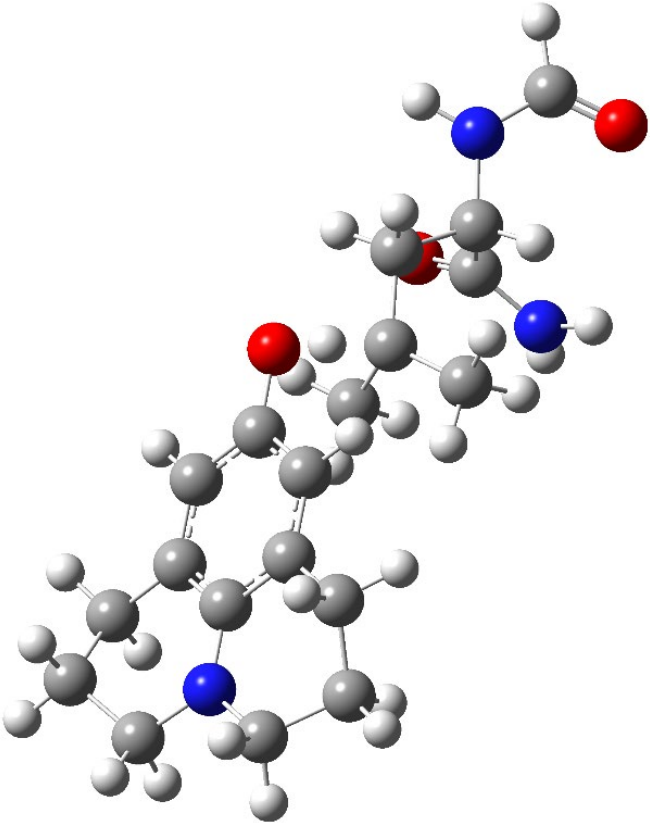

11-δ-TS

Charge=0, Multiplicity=2

N	2.48162500	-0.33441000	1.70999000
H	1.81916400	0.28446400	1.24989500
H	2.23269400	-0.76691400	2.58950700
C	3.73051500	-0.49830500	1.23545200
O	4.58486300	-1.18856800	1.78166200
C	4.02044500	0.20328200	-0.10508300
H	3.28939700	1.00409500	-0.26370000
N	5.33973300	0.80474900	-0.06646800
H	6.12265100	0.27927900	-0.43537800
C	5.60772200	1.85249100	0.74264300
O	4.76306500	2.44524700	1.39915000
H	6.67080900	2.14306000	0.74186800
C	3.97355100	-0.81109200	-1.25401100
H	4.66368800	-1.62962800	-1.01235400
H	4.35518100	-0.31504100	-2.15641700
C	2.58690700	-1.38488700	-1.56391300
H	2.15829400	-1.81855100	-0.64861900

C	2.72167500	-2.52268700	-2.59256500
H	3.15327500	-2.14610800	-3.52688200
H	1.74627900	-2.96195200	-2.82029100
H	3.37354200	-3.31370600	-2.20640700
C	1.64620900	-0.33626600	-2.10196100
H	2.07813900	0.38048500	-2.80638500
H	0.65565200	-0.69478900	-2.39767700
C	-2.82601400	-1.98141800	1.42264200
H	-2.02726200	-2.72926100	1.41013600
H	-3.03319300	-1.74837100	2.47624700
C	-4.09837200	-2.51855900	0.77274100
H	-4.46980000	-3.40185200	1.29992400
H	-3.88855000	-2.80915900	-0.26344100
C	-5.17021800	-1.44033700	0.79394200
H	-5.52141600	-1.27923900	1.82544300
H	-6.03814000	-1.75860800	0.20309300
C	-5.74076600	0.74324400	-0.17518800
H	-6.16703000	0.41446400	-1.13603700
H	-6.54442400	0.68984600	0.57005000
C	-5.23133600	2.17096800	-0.29396500
H	-4.99385300	2.56219600	0.70224400
H	-6.02041300	2.79663000	-0.72056500
C	-3.97701500	2.19143800	-1.16376500
H	-3.57835300	3.20545200	-1.26239900
H	-4.23665200	1.84627800	-2.17429000
N	-4.69214000	-0.18181300	0.23632600
O	0.70650400	1.05364300	-0.18121500
H	1.18260200	0.49698500	-1.03979500
C	-1.02811000	-0.42530300	0.56661400
H	-0.27961700	-1.09372000	0.98923800
C	-1.57848600	1.58049100	-0.65327000
H	-1.25435800	2.48135900	-1.16935600
C	-2.93126300	1.28647600	-0.56190300
C	-3.35292500	0.11826000	0.12756800
C	-2.37393700	-0.73791100	0.69839600
C	-0.60996200	0.74012400	-0.08993700

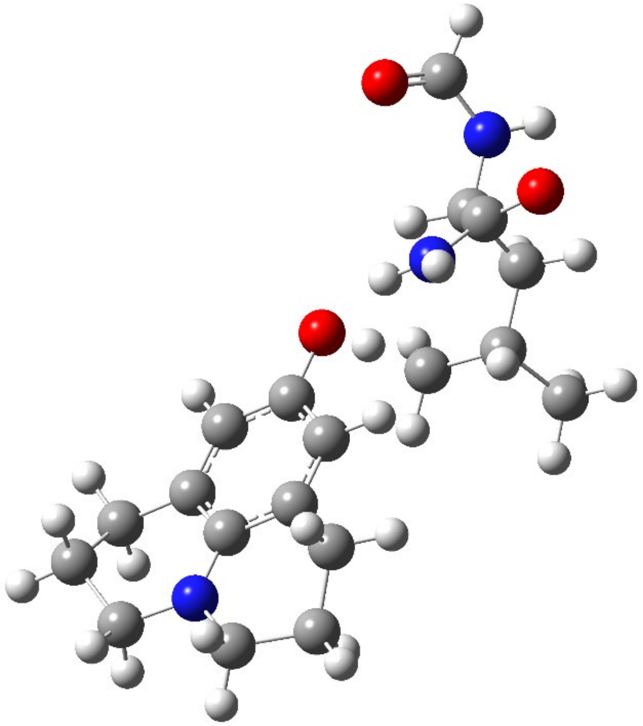

12-β-TS

Charge=0, Multiplicity=2

N	3.40501400	-2.40139700	-0.99254100
H	2.95735100	-2.10710100	-1.84903300
H	3.99633700	-3.22206800	-1.02756100
C	2.92472300	-2.00422100	0.20606700
O	3.25199100	-2.50444200	1.27177500
C	1.97748500	-0.78122800	0.15844700
H	1.64549000	-0.63174200	-0.87617400
N	0.80303400	-1.03062600	0.96567500
H	0.72930000	-0.62180500	1.88852000
C	-0.16339700	-1.86849800	0.53668700
O	-0.11377000	-2.47327000	-0.52725500
H	-1.01001600	-1.95850600	1.23590200
C	2.71979600	0.43524500	0.66764700
H	2.84153600	0.41638800	1.75710600
C	3.96564700	0.88568900	-0.06918300
H	4.69995300	0.06469500	0.00411100
C	4.56285400	2.11567300	0.61726100
H	3.86917500	2.96228400	0.55963900
H	5.49798200	2.41159300	0.13233600
H	4.77423800	1.91808100	1.67299100
C	3.71157500	1.16712100	-1.55301400
H	3.34429100	0.28627700	-2.08842600
H	4.63918300	1.48357500	-2.03962800
H	2.97613300	1.97129400	-1.66692600
C	-0.45067900	1.24316300	-1.20072800
C	-0.14093900	1.80659800	0.04819000
C	-1.11796100	1.80896700	1.06263700
C	-2.36630700	1.25725000	0.83314700
C	-2.68190600	0.67024700	-0.40946000
C	-1.69915700	0.67707800	-1.41502600
H	0.30459700	1.24678100	-1.98218900
H	-0.86653700	2.24638500	2.02458800
H	-3.10199300	1.26709200	1.63183100
H	-1.92630000	0.22361900	-2.37645700
O	1.07071400	2.32954600	0.28505900
H	1.85663400	1.45744500	0.49609200
C	-3.97722300	0.04953500	-0.69482900
H	-4.04668300	-0.45777100	-1.65744800
C	-5.06698800	0.05511200	0.09738400
H	-5.05383600	0.56218400	1.06169700
C	-6.31572500	-0.59454400	-0.27510300
H	-6.33306600	-1.10263600	-1.23913100
C	-7.41137100	-0.59364200	0.49685800
H	-7.41942100	-0.09393200	1.46305000
H	-8.32472800	-1.08991900	0.18465600

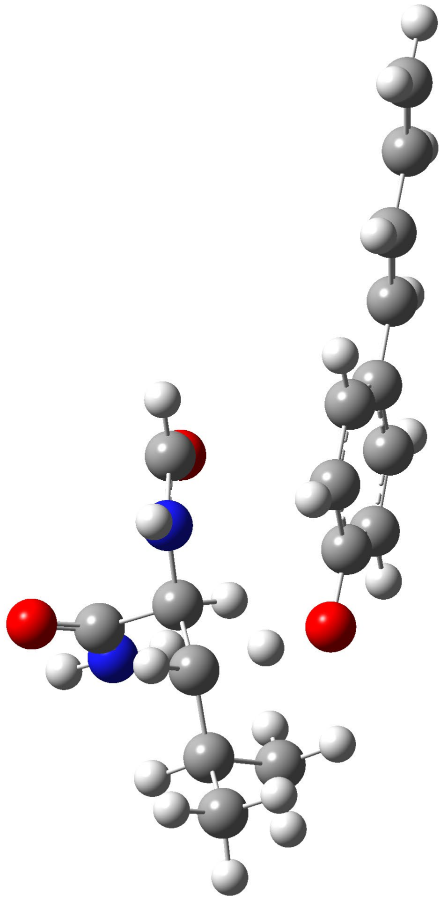

12-γ-TS

Charge=0, Multiplicity=2

N	-4.01355100	-2.40397800	-0.03049300
H	-4.28680200	-2.40365400	-1.00336200
H	-4.08119500	-3.27734600	0.47687300
C	-3.98268700	-1.24949800	0.67170800
O	-3.90145700	-1.20002500	1.89225600
C	-4.01072100	0.03556300	-0.16960400
H	-4.07892000	-0.21388100	-1.23259000
N	-5.19651200	0.80314100	0.16825600
H	-5.14355000	1.48239700	0.91773400
C	-6.41017300	0.47560700	-0.32514600
O	-6.60364200	-0.42927000	-1.12698700
H	-7.22216900	1.11613800	0.05359800
C	-2.75902700	0.88579900	0.09431400
H	-2.73035400	1.14038400	1.16053800
H	-2.88428800	1.81553200	-0.47466700
C	-1.45338000	0.21962800	-0.30950300
C	-0.84774600	-0.76358800	0.66561900
H	0.20340200	-0.95438300	0.41557800
H	-1.36498700	-1.73281200	0.62037300
H	-0.90330300	-0.40030700	1.69607400
C	-1.36060600	-0.20863600	-1.75690200
H	-1.73971400	0.56358700	-2.43431800
H	-0.32337200	-0.43388200	-2.02863900
H	-1.94137100	-1.12793600	-1.92559500
C	1.93471900	1.38856100	1.30622000
C	1.41808500	1.56225300	0.00781400
C	2.19450600	1.14664400	-1.09559300
C	3.44706200	0.58937500	-0.90446700
C	3.97581100	0.42161400	0.39156500
C	3.19220700	0.83320400	1.48589700
H	1.33153300	1.70084600	2.15379200
H	1.78950100	1.28154600	-2.09463300
H	4.02315200	0.28012900	-1.77151000
H	3.58663100	0.70942900	2.49134400
O	0.21982300	2.11586800	-0.17725500
H	-0.63901200	1.21965000	-0.24367600
C	5.29545800	-0.15688700	0.65065200
H	5.55640200	-0.26174300	1.70434900
C	6.19828800	-0.55597100	-0.26651900
H	5.98844200	-0.46514600	-1.33169500
C	7.49128400	-1.12053800	0.09251900
H	7.70702400	-1.20900500	1.15723200
C	8.40042100	-1.52451500	-0.80576200
H	8.21018400	-1.44729000	-1.87401700
H	9.35442700	-1.94065700	-0.49810700

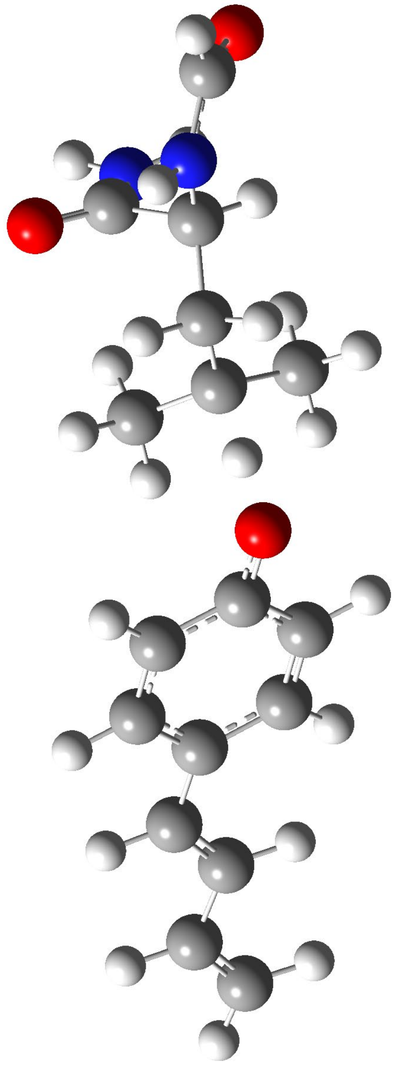

12-δ-TS

Charge=0, Multiplicity=2

N	-2.21284400	-0.84200500	1.58914800
H	-1.50720900	-1.01051600	0.87958000
H	-2.02649800	-1.10480300	2.54777600
C	-3.46186200	-0.46683700	1.25076600
O	-4.37878000	-0.34423600	2.05491000
C	-3.66302500	-0.12434800	-0.23756300
H	-2.86185100	-0.58241000	-0.82843900
N	-4.92538400	-0.66940300	-0.69817000
H	-5.73775100	-0.06532600	-0.71809000
C	-5.12505100	-2.00402100	-0.75755100
O	-4.25121900	-2.83498000	-0.55287500
H	-6.15632700	-2.28038300	-1.03001100
C	-3.68187500	1.39627400	-0.43106200
H	-4.46181300	1.81057200	0.22042900
H	-3.97706200	1.60112400	-1.46879400
C	-2.35464200	2.10798800	-0.15021400
H	-2.00749500	1.85178100	0.86048000
C	-2.56912000	3.63197700	-0.19490200
H	-2.91996000	3.94218700	-1.18559300
H	-1.63967100	4.16618700	0.02140300
H	-3.31847900	3.93178800	0.54504800
C	-1.28460100	1.74152400	-1.15042100
H	-1.61033900	1.70728800	-2.19473500
H	-0.32992100	2.26085900	-1.02095600
C	1.33188900	0.10567100	0.86720400
C	0.95536400	-0.38897900	-0.39126500
C	1.94827300	-0.72442200	-1.32826400
C	3.28771400	-0.59604300	-1.00000800
C	3.68163700	-0.10847900	0.26134800
C	2.67694800	0.24244800	1.18079000
H	0.56010500	0.37867000	1.58254500
H	1.64208400	-1.09680700	-2.30111900
H	4.03788400	-0.87310600	-1.73415700
H	2.96497200	0.62515400	2.15659200
O	-0.34330800	-0.56217800	-0.70334700
H	-0.85384300	0.49581400	-0.93810700
C	5.08367300	0.05382600	0.65445200
H	5.24138300	0.45861200	1.65452500
C	6.16868500	-0.24392100	-0.08592600
H	6.06363100	-0.65440600	-1.08976900
C	7.52916500	-0.04335400	0.39272400
H	7.63838200	0.36391200	1.39774700
C	8.61977400	-0.33085700	-0.33118300
H	8.53728200	-0.73805000	-1.33649900
H	9.61886100	-0.16782900	0.06024100

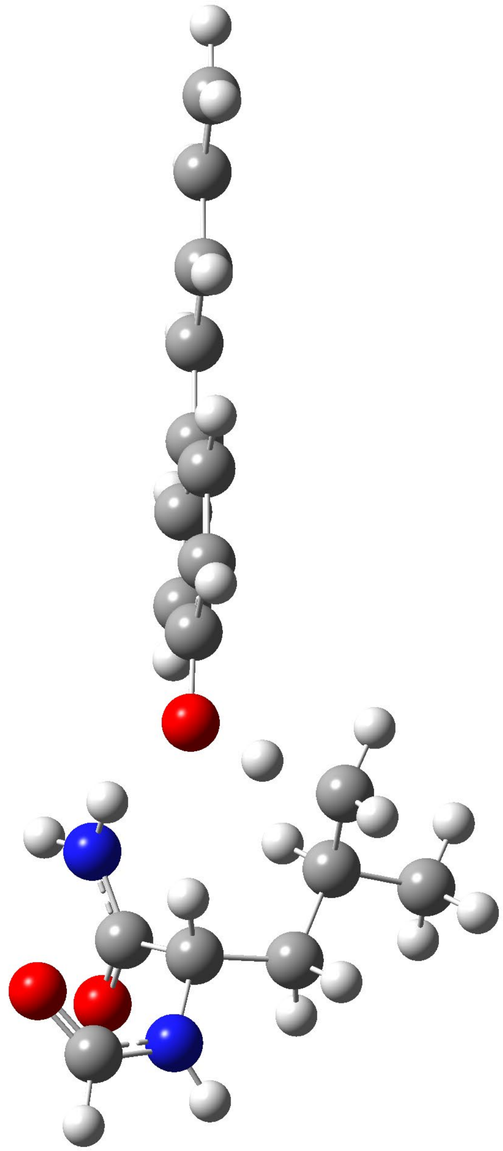

13-β-TS

Charge=0, Multiplicity=2

N	3.72990400	2.96075200	0.62822300
H	3.31346900	2.71939100	1.51626200
H	4.12369900	3.88763700	0.52862200
C	3.42645300	2.26750800	-0.49086600
O	3.69993800	2.64586400	-1.62005400

C	2.78113000	0.88046700	-0.25306100
H	2.38424500	0.84028200	0.76905600
N	1.67376100	0.68853000	-1.16363500
H	1.80043900	0.13309600	-2.00004600
C	0.49693700	1.31659300	-0.96277600
O	0.28418600	2.07783700	-0.02672500
H	-0.26374200	1.07878000	-1.72314800
C	3.83544700	-0.18626400	-0.46308200
H	4.06594500	-0.33336800	-1.52508300
C	5.06412800	-0.17226400	0.42490900
H	5.59440000	0.77611700	0.23085600
C	5.99863800	-1.32207400	0.04372400
H	5.51716000	-2.28817700	0.23332800
H	6.91930800	-1.28209200	0.63359600
H	6.27073000	-1.27795200	-1.01568900
C	4.71940700	-0.22952200	1.91606900
H	4.10743900	0.61885300	2.23718000
H	5.63554900	-0.21833200	2.51437600
H	4.17457900	-1.15215500	2.14537200
C	-0.77369700	-2.26738400	-1.05783600
C	-1.42603900	-1.53508300	-0.04834200
C	-0.67889400	-1.14318800	1.07874400
C	0.67306900	-1.42962400	1.17031300
C	1.32681000	-2.11181900	0.12735700
C	0.58335000	-2.54393100	-0.98349800
H	-1.34458800	-2.59826000	-1.92198400
H	-1.16409600	-0.61203900	1.89194900
H	1.25146300	-1.12274600	2.03785800
H	1.09313300	-3.08213000	-1.77735100
C	-2.84409200	-1.20144600	-0.21685700
H	-3.36859200	-1.77939300	-0.97668000
C	-3.49635800	-0.22976800	0.44638500
H	-2.94538200	0.39466600	1.14869300
C	-4.91517400	0.12076400	0.28810200
C	-5.83835400	-0.70639700	-0.37349100
C	-5.37373800	1.33333100	0.82544500
C	-7.16785200	-0.32033200	-0.50723600
H	-5.52024300	-1.66487000	-0.77284100
C	-6.70526800	1.72032800	0.69144100
H	-4.67168100	1.97932500	1.34699000
C	-7.60783000	0.89554700	0.02175400
H	-7.86777100	-0.97452200	-1.01872000
H	-7.03714500	2.66532300	1.11103600
H	-8.64723600	1.19171200	-0.08199100
O	2.64800400	-2.33740900	0.18397100
H	3.22779000	-1.34709700	-0.14449000

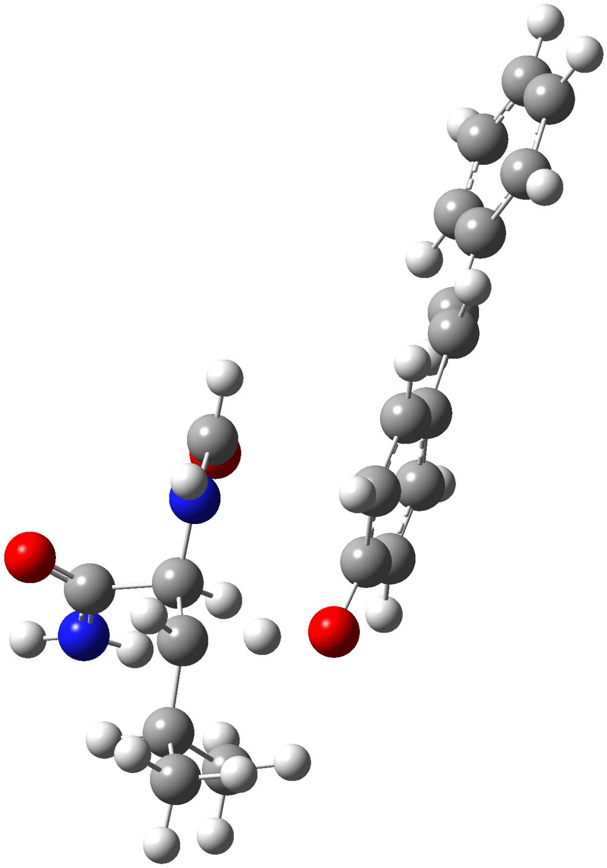

13-γ-TS

Charge=0, Multiplicity=2

N	5.07796700	2.22961500	-1.29512100
H	5.42055200	1.71655300	-2.09494800
H	4.99232500	3.23478800	-1.37776300
C	5.07440300	1.67458600	-0.06357800
O	4.86631300	2.30707500	0.96406200
C	5.30751600	0.15728000	-0.01996700

H	5.50365000	-0.22389800	-1.02659500
N	6.48357700	-0.12509700	0.78322900
H	6.37529700	-0.22432500	1.78549000
C	7.73207300	-0.02747300	0.27707300
O	7.98447100	0.23301400	-0.89196400
H	8.51810100	-0.22335500	1.02327700
C	4.09338700	-0.55938600	0.59254400
H	3.94525400	-0.17957800	1.61061700
H	4.35897100	-1.62162500	0.66164900
C	2.80557800	-0.41271100	-0.20142200
C	2.00959700	0.85680900	-0.00310000
H	0.98866500	0.73297700	-0.38536000
H	2.45589800	1.69371400	-0.55902800
H	1.95714700	1.14259400	1.05172200
C	2.86636500	-0.88718300	-1.63580000
H	3.38789000	-1.84581900	-1.72504700
H	1.85814700	-0.99780200	-2.05045100
H	3.39033100	-0.15212900	-2.26519700
C	-1.91286700	-1.79135300	-0.84982900
C	-2.64255200	-0.98165400	0.04020200
C	-2.00311300	-0.55179200	1.22121400
C	-0.69796000	-0.91776500	1.49811300
C	0.02284500	-1.73971100	0.60464800
C	-0.60468600	-2.16630600	-0.58122700
H	-2.39153100	-2.13175700	-1.76478300
H	-2.53102800	0.08149500	1.92764300
H	-0.20391000	-0.58219500	2.40550300
H	-0.04904300	-2.79817200	-1.26820800
C	-4.02224000	-0.62804700	-0.30506700
H	-4.35282300	-0.99641000	-1.27495100
C	-4.87648500	0.07643200	0.45941300
H	-4.55158700	0.42421500	1.43873100
C	-6.26033700	0.43407500	0.11678100
C	-6.84267500	0.16466200	-1.13392400
C	-7.04297200	1.08017700	1.08645100
C	-8.16070600	0.52245200	-1.39498400
H	-6.26405000	-0.32207900	-1.91299400
C	-8.36335400	1.43927500	0.82518800
H	-6.60594200	1.29949400	2.05758400
C	-8.92898500	1.16032400	-0.41762900
H	-8.59197200	0.30614800	-2.36798100
H	-8.94876000	1.93685200	1.59261500
H	-9.95735900	1.43848800	-0.62693300
O	1.27215000	-2.10950000	0.88596200
H	2.06451500	-1.30064600	0.37356200

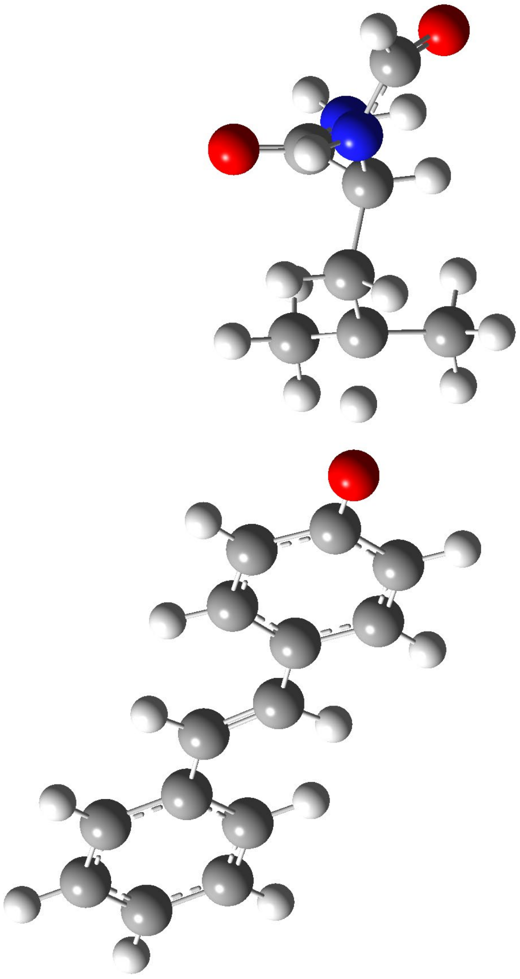

13-δ-TS

Charge=0, Multiplicity=2

N	-3.38275000	-0.63660000	1.68157400
H	-2.74362000	-0.91985600	0.94609500
H	-3.11915900	-0.77050600	2.64873600
C	-4.64775900	-0.27024200	1.39767100
O	-5.48998200	-0.00969600	2.24936600
C	-4.97040600	-0.12780500	-0.10168600
H	-4.22875700	-0.67952200	-0.68989200
N	-6.27325100	-0.70152900	-0.37822600
H	-7.08151300	-0.09163300	-0.37801900
C	-6.48401800	-2.03249700	-0.28244200
O	-5.59967000	-2.85273400	-0.07995800
H	-7.53850300	-2.31879400	-0.42536300
C	-4.98805100	1.35330100	-0.49699500
H	-5.70971300	1.86715100	0.15086800
H	-5.36201300	1.42386500	-1.52698800
C	-3.63447000	2.06630100	-0.41594000
H	-3.21104900	1.93558700	0.58977300
C	-3.83328700	3.57603600	-0.64305200
H	-4.25884400	3.76290700	-1.63539000
H	-2.88309600	4.11271100	-0.57177400
H	-4.51715300	3.98602700	0.10739000
C	-2.65193200	1.54956600	-1.43950000
H	-3.06085400	1.38772900	-2.44175900
H	-1.68450900	2.06119800	-1.45435900
C	1.39155700	0.35546500	0.87621000
C	2.36343400	-0.13761500	-0.01276200
C	1.92282200	-0.79835100	-1.17613200
C	0.57196300	-0.95969200	-1.43695700
C	-0.38697100	-0.48398800	-0.52560200
C	0.03576900	0.18686500	0.63243100
H	1.71380200	0.87528800	1.77489200
H	2.64343500	-1.18399700	-1.89054200
H	0.23119400	-1.46551000	-2.33527200
H	-0.70858400	0.56984400	1.32615000
C	3.77828500	0.06471400	0.31622400
H	3.95145700	0.63583000	1.22696200
C	4.83054200	-0.38628600	-0.38961800
H	4.65732800	-0.95975200	-1.29905300
C	6.24894800	-0.18447900	-0.05992400
C	6.68664400	0.48497400	1.09587900
C	7.21813100	-0.68585000	-0.94241900
C	8.04376200	0.64729600	1.35085800
H	5.96553100	0.87965800	1.80513000
C	8.57816100	-0.52312800	-0.68767800
H	6.89556300	-1.20891300	-1.83940100
C	8.99710500	0.14550500	0.46097000
H	8.36157800	1.16696700	2.24996200
H	9.30885000	-0.91948800	-1.38639000
H	10.05565800	0.27452100	0.66488700
O	-1.69632700	-0.69125800	-0.76418200
H	-2.21856600	0.33455300	-1.09908900

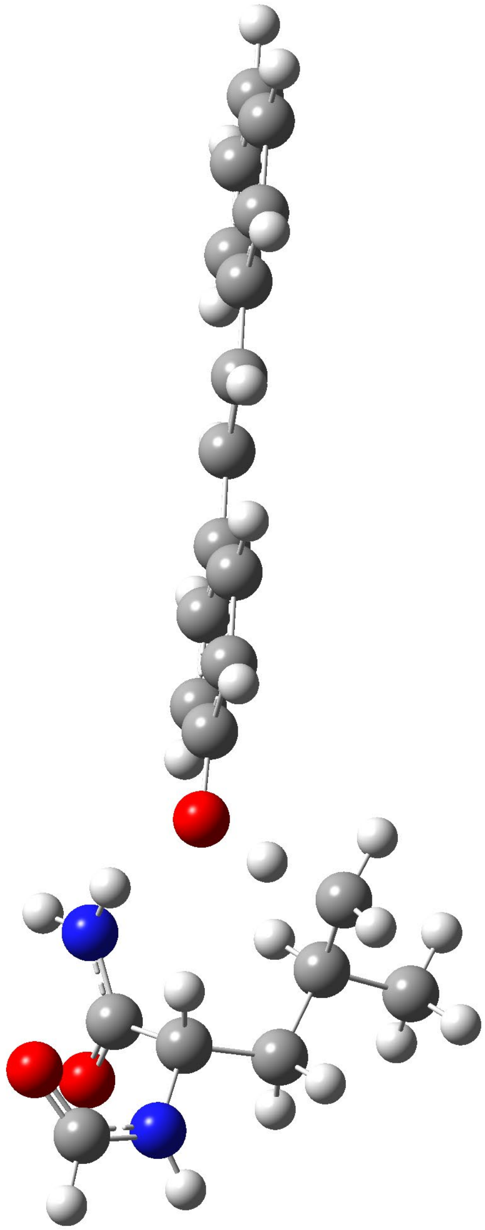

14(1)-δ-TS

Charge=0, Multiplicity=2

C	2.73869900	-1.36267100	-0.21391500
C	1.60469300	-2.14317500	-0.36628900
C	0.33040600	-1.60015900	-0.12915200
C	0.21191500	-0.25759600	0.27302800
C	1.35863600	0.53090300	0.41904900
C	2.61204300	-0.02145200	0.18216000
H	3.71244200	-1.80128300	-0.40031900
H	1.25871100	1.56744200	0.72363400
O	1.71806100	-3.43872300	-0.75205000
H	0.83302700	-3.83386900	-0.77924000
O	-0.70537800	-2.43341800	-0.32029800
H	-1.57899400	-2.09337800	-0.03338200
O	-1.01898000	0.23152900	0.51679400
H	-1.48004800	0.68419200	-0.48767600
C	3.80392200	0.85406900	0.35583200
O	3.74812000	2.02142100	0.68554800
O	4.95339800	0.21722400	0.10754700
C	6.15684300	0.99329400	0.24953700
H	6.21186300	1.37424100	1.27490700
H	6.10652800	1.84951900	-0.43160000
C	7.33093600	0.09290600	-0.07360800
H	7.21545800	-0.29411900	-1.09204000
H	7.32039700	-0.76662400	0.60566100
C	8.64854300	0.85295000	0.05641300
H	8.78661100	1.23262500	1.07425100
H	9.49702900	0.20441200	-0.17688100
H	8.68096700	1.70691700	-0.62839400
N	-3.27223800	1.75466100	1.94465800
H	-2.34589600	1.40316500	1.73035200
H	-3.37257400	2.47719600	2.64594300
C	-4.37701700	1.19345700	1.41603000
O	-5.52287500	1.51142800	1.71861200
C	-4.11920600	0.12982600	0.35187600
H	-3.10159400	-0.24454200	0.44344400
N	-4.98581400	-1.00260400	0.61358400
H	-5.95181000	-0.83195100	0.86983900
C	-4.53312500	-2.26060600	0.52096900
O	-3.37841400	-2.56893900	0.21916100
H	-5.29307200	-3.02296300	0.74189500
C	-4.35038600	0.69557400	-1.05897500
H	-5.35830900	1.12608000	-1.09961600
H	-4.32462100	-0.14852100	-1.76050300
C	-3.31598700	1.73872600	-1.49625500
H	-3.26905600	2.54513200	-0.75212500
C	-3.74248800	2.36495900	-2.83701800
H	-3.81881400	1.59546500	-3.61308900
H	-3.02043500	3.11634500	-3.16879300
H	-4.71942300	2.84832300	-2.73338400
C	-1.94031400	1.13976200	-1.66437900
H	-1.90076100	0.22701700	-2.26894300
H	-1.14141100	1.84599900	-1.90596100

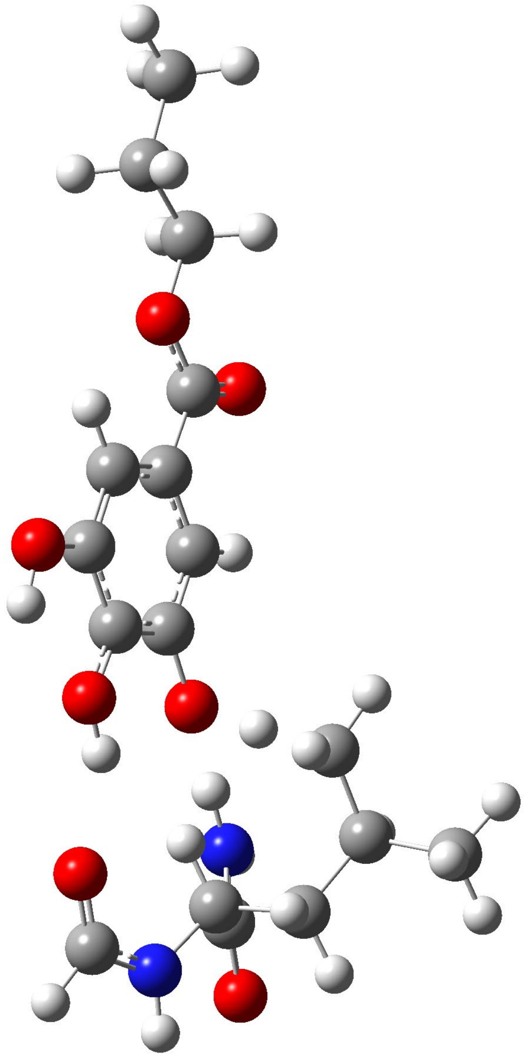

14[(2)]-δ-TS

Charge=0, Multiplicity=2

C	-2.22372400	0.42794400	0.10973100
C	-0.83388500	0.50089500	0.20679300
C	-0.10079800	-0.66506800	0.49987700
C	-0.76962900	-1.89200100	0.68416500
C	-2.15068400	-1.96572900	0.58846800
C	-2.86287700	-0.79755900	0.29981300
H	-2.78556500	1.32743900	-0.11133900
H	-2.66511400	-2.90865900	0.73373200
O	-0.23932600	1.68689800	0.00341900
H	0.71930700	1.69918100	0.19245900
O	1.24163300	-0.63268300	0.61672600
H	1.74566500	-0.79992700	-0.42963100
O	-0.04787800	-3.00426100	0.95025600
H	0.88549400	-2.75156900	1.02804200
C	-4.35043600	-0.91248400	0.20049800
O	-4.95824700	-1.95337700	0.33870500
O	-4.94363900	0.25587900	-0.05738000
C	-6.37869800	0.23236000	-0.16836800
H	-6.79786800	-0.15195000	0.76761600
H	-6.65782400	-0.45289300	-0.97590100
C	-6.84525000	1.64539100	-0.44955500
H	-6.37001500	2.00262600	-1.36975900
H	-6.51448400	2.29985900	0.36438600
C	-8.36498200	1.69729700	-0.58518200
H	-8.85518900	1.35412000	0.33196800
H	-8.70347000	2.71751000	-0.78392500
H	-8.70998900	1.06303600	-1.40852400
N	4.06484900	-0.99291500	1.80615000
H	3.05355700	-0.98193500	1.75825000
H	4.51591400	-1.58951200	2.48757800
C	4.80334300	-0.08915500	1.13029800
O	6.01773400	0.03069600	1.24839900
C	4.02328800	0.78245300	0.14837300
H	2.95678300	0.70700400	0.35396500
N	4.37249700	2.16959700	0.38592900
H	5.34845200	2.42045600	0.49612700
C	3.42873000	3.12272500	0.44374700
O	2.22180200	2.91852800	0.31427600
H	3.82899100	4.13090700	0.62233900
C	4.31201900	0.36976500	-1.30221500
H	5.39704600	0.38833800	-1.45980900
H	3.86909700	1.13071600	-1.95785100
C	3.74951100	-1.00448200	-1.68161400
H	4.11240400	-1.75974100	-0.97071100
C	4.24953400	-1.40540800	-3.08272900
H	3.92189500	-0.67433900	-3.82982900
H	3.86675800	-2.38787300	-3.37261800
H	5.34366800	-1.44403000	-3.09396100
C	2.24118500	-1.01714800	-1.69372800
H	1.76497500	-0.18474700	-2.22314800
H	1.77159100	-1.98299000	-1.89945600

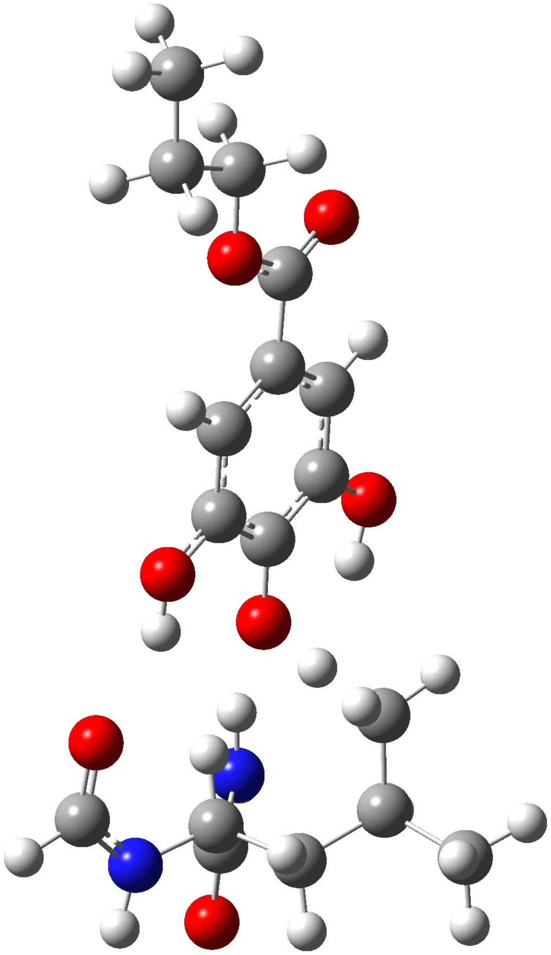

14(3)-δ-TS

Charge=0, Multiplicity=2

C	-1.67194700	0.28908000	0.17673000
C	-0.36253500	0.78523600	0.19859500
C	-0.12617700	2.15886000	0.01363300
C	-1.21252900	3.02961200	-0.18060900
C	-2.50618200	2.53762600	-0.19621100
C	-2.73503900	1.16505600	-0.01399500
H	-1.83138800	-0.77409000	0.31716700
H	-3.33974700	3.21568600	-0.34523800
O	0.69397800	-0.02444600	0.40368800
H	1.12320600	-0.40321000	-0.64297000
O	1.09089700	2.72710100	-0.00333700
H	1.83119000	2.14423600	0.26744400
O	-0.98442300	4.35468000	-0.35662100
H	-0.03132600	4.51864100	-0.28750800
C	-4.14631100	0.69125900	-0.03769600
O	-5.10332500	1.41630800	-0.21642400
O	-4.25362100	-0.62773700	0.16102100
C	-5.58868000	-1.16469800	0.16053800
H	-6.05655100	-0.94721300	-0.80554400
H	-6.17007200	-0.66483500	0.94257900
C	-5.48959000	-2.65555800	0.40792300
H	-4.98467200	-2.82643100	1.36514200
H	-4.86989900	-3.10793000	-0.37428900
C	-6.87509700	-3.29659300	0.42006800
H	-7.38719900	-3.14813600	-0.53641000
H	-6.80525600	-4.37274900	0.59894400
H	-7.50227300	-2.86579900	1.20760100
N	2.38153600	-2.27784800	1.63081800
H	1.58998000	-1.67155600	1.44817300
H	2.24445700	-3.10679700	2.19443000
C	3.63110100	-1.92959900	1.26618000
O	4.63657200	-2.57034300	1.55565100
C	3.72874700	-0.67226900	0.40693400
H	2.82307400	-0.07943200	0.51738600
N	4.80823700	0.15141500	0.91547900
H	5.67325700	-0.29341400	1.20200800
C	4.67783800	1.48215000	1.00838900
O	3.66625500	2.10958400	0.68793600
H	5.56835400	1.98955000	1.40484800
C	3.94796800	-1.03252700	-1.07174200
H	4.82664200	-1.68486600	-1.14384500
H	4.18287200	-0.10271000	-1.60618800
C	2.74190400	-1.70091700	-1.74089200
H	2.44106300	-2.58271600	-1.15938900
C	3.12876000	-2.18162400	-3.15192600
H	3.44973800	-1.33648700	-3.77073600
H	2.28446600	-2.66881300	-3.64779100
H	3.95407400	-2.89871300	-3.09288100
C	1.56540500	-0.76236400	-1.86169200
H	1.79361700	0.21702700	-2.29616800
H	0.64751700	-1.20178000	-2.26152200

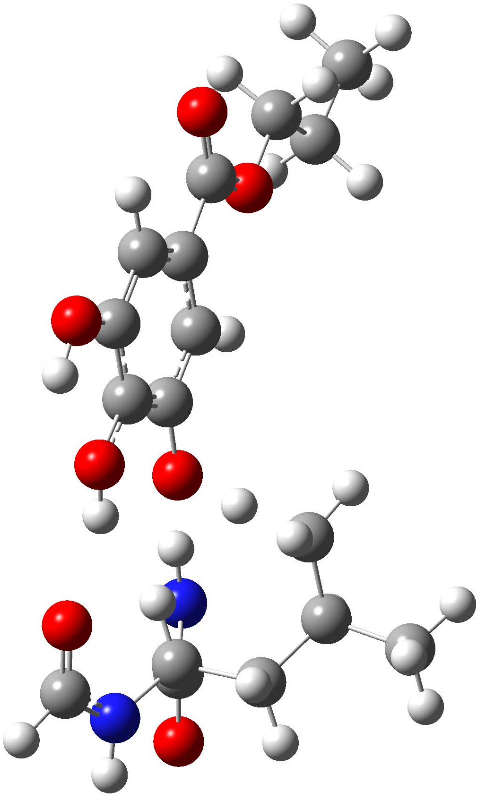

15(1)-δ-TS

Charge=0, Multiplicity=2

C	7.42454500	-0.59415800	0.11286600
C	8.61892700	0.08960300	-0.08141400
C	8.71185200	1.45507800	0.20994900
C	7.59660400	2.12301700	0.70015200
C	6.40000300	1.43294200	0.89515800
C	6.29479500	0.06957200	0.60501100
H	7.37469000	-1.65635500	-0.12224400
H	7.68020300	3.18108700	0.92898300
H	5.53558800	1.96576800	1.28421200
O	9.88222300	2.12412200	0.02058400
H	10.54494600	1.50455900	-0.31822600
O	9.76780300	-0.48343900	-0.55569900
H	9.63304600	-1.42454800	-0.73060700
C	4.98297100	-0.65516400	0.79633100
H	5.15237500	-1.73915000	0.75297500
H	4.60738100	-0.43443000	1.80035200
C	3.91354300	-0.29095700	-0.25760800
H	3.85123700	0.80743200	-0.31201800
C	2.52271400	-0.80841400	0.17912900
H	2.64258400	-1.85614400	0.49967500
C	1.50769600	-0.79265500	-0.98423400
H	1.87486300	-1.42764700	-1.79662800
H	1.44019800	0.23031800	-1.37911500
C	0.13077300	-1.27001900	-0.58911200
C	-0.10495300	-2.63348000	-0.34120300
C	-0.93163500	-0.38793600	-0.42622700
C	-1.35180400	-3.09827200	0.05913200
H	0.70780500	-3.34514900	-0.46891100
C	-2.19581100	-0.83541500	-0.01682000
H	-0.80321000	0.67752700	-0.60491500
C	-2.41598400	-2.20710000	0.22395400
H	-1.52510500	-4.15403900	0.24364600
O	-3.22036200	0.02646300	0.15958400
H	-3.80831500	0.16064300	-0.84361000
O	-3.61398400	-2.69490600	0.59428600
H	-4.28054800	-2.00392400	0.77213300
C	4.35717800	-0.81111900	-1.62687900
H	3.72559000	-0.43921600	-2.43763000
H	5.38266500	-0.49487400	-1.84243500
H	4.33234000	-1.90874700	-1.65127100
C	1.97615300	0.00513100	1.35604800
H	1.05152900	-0.42941300	1.74659900
H	1.75278600	1.03060700	1.03383900
H	2.68502600	0.06374000	2.18588800
N	-4.64306300	2.55729900	1.07846400
H	-3.90916800	1.87570800	0.91973100
H	-4.39444700	3.48132700	1.40688700
C	-5.94479500	2.21884000	0.99739800
O	-6.87012100	2.97495800	1.27730100
C	-6.21789900	0.80794200	0.48276300
H	-5.32147200	0.19870900	0.58231800
N	-7.21407400	0.18964200	1.33535100
H	-8.01975300	0.73269400	1.62401400

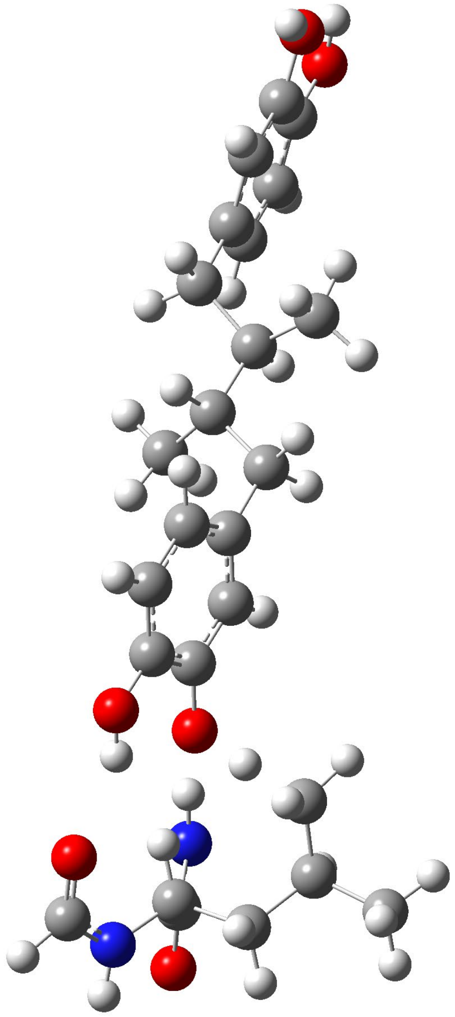

C	-7.08304700	-1.08289400	1.74361200
O	-6.14023300	-1.81684100	1.44845000
H	-7.90889600	-1.42855100	2.38220900
C	-6.67513800	0.83720000	-0.98468900
H	-7.53560000	1.51217100	-1.06817100
H	-7.02180700	-0.17237000	-1.24153500
C	-5.58145600	1.25393800	-1.97449600
H	-5.16709800	2.22628100	-1.67492500
C	-6.18657600	1.41848700	-3.38102700
H	-6.62516800	0.47438100	-3.72253100
H	-5.42511400	1.72384800	-4.10430800
H	-6.97393100	2.17941600	-3.36812500
C	-4.46491900	0.24073900	-2.04459400
H	-4.78253000	-0.80045500	-2.16626500
H	-3.61774700	0.50791100	-2.68195200

15[(2)]-δ-TS

Charge=0, Multiplicity=2

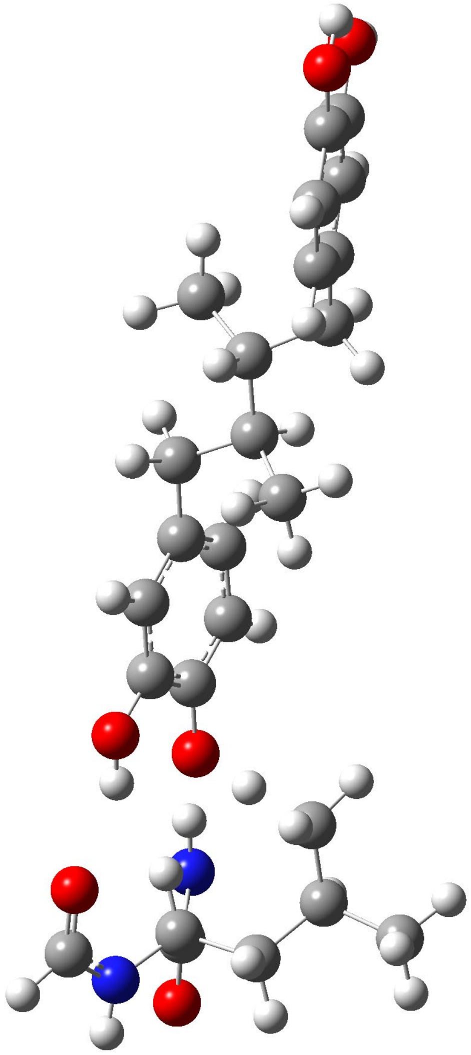

C	7.43350400	0.70618100	0.78064600
C	8.75751400	0.48729700	0.41938500
C	9.09392400	0.20159300	-0.90850300
C	8.08940200	0.14065900	-1.86638100
C	6.76160900	0.36165700	-1.49993900
C	6.41369700	0.64578700	-0.17624200
H	7.19434600	0.92567800	1.82011200
H	8.36091700	-0.07632600	-2.89496300
H	5.98492200	0.31544200	-2.25963800
O	10.39124700	-0.01057800	-1.26203000
H	10.94732100	0.07307300	-0.47363800
O	9.81257000	0.52956600	1.29040400
H	9.50826100	0.72871600	2.18602400
C	4.96829500	0.85694400	0.20898500
H	4.91783100	1.31593500	1.20521800
H	4.52252400	1.57140600	-0.49014400
C	4.13415900	-0.44338400	0.23546800
H	4.30444100	-0.97652200	-0.71312900
C	2.62498400	-0.11205200	0.31245100
H	2.49328000	0.65785600	1.08977200
C	1.78223800	-1.33822400	0.72592200
H	2.10767100	-1.68866000	1.71053300
H	1.96713300	-2.15224500	0.01259700
C	0.30220700	-1.04965800	0.78937100
C	-0.21192500	-0.24086300	1.81877200
C	-0.56769800	-1.55044100	-0.17737200
C	-1.56514800	0.05841800	1.86550600
H	0.45669100	0.14748700	2.58271800
C	-1.93445100	-1.25967900	-0.14082800
H	-0.19668700	-2.17742800	-0.98382900
C	-2.44069900	-0.44864700	0.89629500
H	-1.97913600	0.68039400	2.65448300
O	-2.71885700	-1.76163900	-1.11070200
H	-3.67134800	-1.59891100	-0.96999200
O	-3.76585900	-0.18854600	0.93490200
H	-4.00299800	0.77554500	0.32647200
C	4.62482400	-1.33548100	1.37771000

H	4.18242200	-2.33433800	1.34372700
H	5.71119300	-1.45910600	1.32680800
H	4.38300900	-0.88820900	2.35104200
C	2.11416000	0.44209800	-1.02111700
H	1.08612400	0.80617800	-0.93550300
H	2.12570300	-0.34565900	-1.78528400
H	2.72522800	1.27084100	-1.38826200
N	-6.53831900	0.15079600	2.14994500
H	-5.54330700	-0.02649000	2.06625800
H	-6.94309600	0.26092400	3.07047500
C	-7.35099800	0.09890100	1.07653700
O	-8.57278700	0.20067200	1.13274500
C	-6.64163100	-0.05955500	-0.26540000
H	-5.63415700	-0.44016500	-0.10728000
N	-7.33326500	-1.07436300	-1.03546900
H	-8.34666200	-1.07755700	-1.04645800
C	-6.65263300	-2.00382000	-1.72534000
O	-5.42503300	-2.08155900	-1.76669600
H	-7.29690700	-2.71211600	-2.26612900
C	-6.58651700	1.27888400	-1.01975400
H	-7.60571600	1.67604900	-1.10014100
H	-6.23353000	1.06843800	-2.03778700
C	-5.66399900	2.32226100	-0.37949700
H	-5.95509700	2.47485300	0.66883500
C	-5.81882100	3.67085700	-1.10681200
H	-5.55147000	3.56896800	-2.16441700
H	-5.17385000	4.43433500	-0.66254700
H	-6.85565600	4.01774400	-1.04538600
C	-4.21424100	1.90604800	-0.43499200
H	-3.86290300	1.55572800	-1.41144900
H	-3.50622800	2.57509900	0.06136600

15(3)-δ-TS

Charge=0, Multiplicity=2

C	-0.57979900	-0.88200800	-0.94448200
C	-1.92294200	-0.78210100	-0.56995400
C	-2.36067800	0.36047700	0.13146800
C	-1.44751900	1.38096200	0.42833200
C	-0.11776700	1.26754800	0.05252000
C	0.33043700	0.12961600	-0.64225100
H	-0.25960800	-1.77204800	-1.48003800
H	-1.81197100	2.25618200	0.95929200
H	0.58373500	2.06394100	0.28834100
O	-3.65740100	0.44244100	0.50133700
H	-4.28029400	0.95606600	-0.33819200
O	-2.75478200	-1.78489700	-0.90292800
H	-3.65266200	-1.68993400	-0.53080200
C	1.78422900	0.00961900	-1.02885300
H	1.90429300	-0.79813500	-1.76189200
H	2.08723800	0.93806000	-1.52339600
C	2.72004400	-0.27936900	0.16724300
H	2.49313000	0.44881900	0.96195400
C	4.19639200	-0.06734900	-0.24417500
H	4.34773100	-0.57380900	-1.21097600
C	5.17812100	-0.69893600	0.76573400
H	4.98501800	-1.77366800	0.83689700
H	4.98723400	-0.27059600	1.75931900
C	6.62995900	-0.49723400	0.39937900
C	7.19502200	-1.19386900	-0.67237600
C	7.43238800	0.41130500	1.09897800
C	8.52388400	-0.99030000	-1.04603300
H	6.59116500	-1.90969000	-1.22462000
C	8.75743400	0.61352100	0.73228000
H	7.02159000	0.97011900	1.93851200
C	9.31143700	-0.08457400	-0.34670900
H	8.96449600	-1.53285100	-1.87690900
O	9.60995800	1.47667700	1.36566200
H	9.16255100	1.92348300	2.09681200
O	10.60927500	0.11554300	-0.70595000
H	11.00087600	0.77036100	-0.10958100
C	2.42956100	-1.68130900	0.70791400
H	2.94495000	-1.87587400	1.65189400
H	1.35785100	-1.80902100	0.88953900
H	2.73945500	-2.44771300	-0.01451200
C	4.51325800	1.42118800	-0.41566600
H	5.51763900	1.57012400	-0.82248700
H	4.46681400	1.93087300	0.55569100
H	3.81146700	1.92176200	-1.08796400
N	-6.03460300	1.01587700	2.31331200
H	-5.07870100	0.93560700	1.98398900
H	-6.21817000	1.51563500	3.17345600
C	-7.04119500	0.35163400	1.71203600
O	-8.19496100	0.32728900	2.12964100
C	-6.66401300	-0.36011600	0.41560400
H	-5.58501900	-0.49500800	0.36994300
N	-7.22907700	-1.69487100	0.43821500
H	-8.17936500	-1.82214700	0.76707400

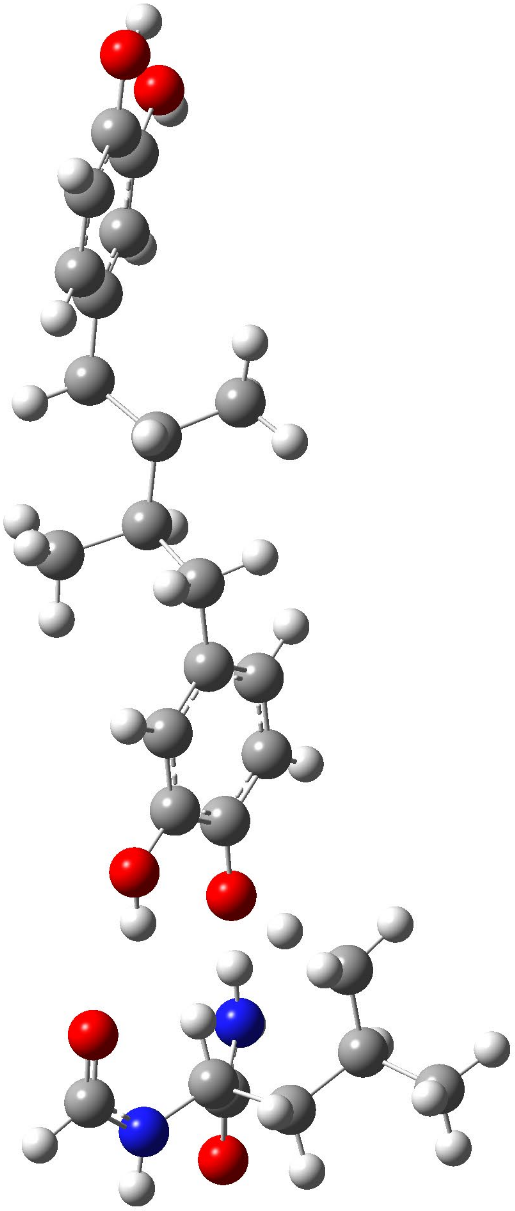

C	-6.51558900	-2.75448200	0.02428800
O	-5.35833600	-2.69664700	-0.39079900
H	-7.06173700	-3.70680500	0.08757300
C	-7.14734200	0.43363700	-0.80908500
H	-8.22350700	0.61731200	-0.70349200
H	-7.00667000	-0.20527600	-1.69079200
C	-6.40732400	1.75755700	-1.03166600
H	-6.46835600	2.36927500	-0.12124200
C	-7.08218500	2.54618800	-2.16933000
H	-7.05831200	1.97048700	-3.10117400
H	-6.57502100	3.49961900	-2.34259700
H	-8.12792300	2.75305600	-1.91884800
C	-4.95635900	1.54277100	-1.38692200
H	-4.77005500	0.80799200	-2.17747800
H	-4.35086300	2.44725100	-1.48933700

15(4)-δ-TS

Charge=0, Multiplicity=2

C	-1.00298400	-0.57732500	0.85407600
C	-2.27068300	-1.02060400	0.45091600
C	-2.41233300	-2.26943900	-0.18749900
C	-1.27355200	-3.05468100	-0.39003500
C	-0.02735500	-2.59966500	0.02251300
C	0.13329400	-1.35288400	0.65239900
H	-0.93430000	0.39571300	1.33578900
H	-1.38757900	-4.02037200	-0.87280500
H	0.84606000	-3.22703300	-0.14289500
O	-3.60016000	-2.73392400	-0.61580100
H	-4.35689600	-2.17304100	-0.35862300
O	-3.37405700	-0.27125200	0.66294900
H	-3.55173500	0.44088200	-0.24836800
C	1.50657700	-0.87502100	1.05861600
H	1.40929300	-0.02709600	1.74909800
H	2.00649800	-1.67696000	1.61080900
C	2.38425600	-0.42981500	-0.13287700
H	2.35649200	-1.22708600	-0.89282600
C	3.85745600	-0.26890800	0.31089000
H	3.86084100	0.31043900	1.24832900
C	4.69192800	0.51836800	-0.72217500
H	4.25797000	1.51372900	-0.85794000
H	4.62870300	0.00428100	-1.69112600
C	6.14202400	0.67916900	-0.32989100
C	6.50812300	1.55785300	0.69325100
C	7.14641300	-0.06762900	-0.95633800
C	7.83939800	1.68952800	1.09030700
H	5.74432300	2.15251100	1.18824000
C	8.47357900	0.06247700	-0.56525900
H	6.89246500	-0.76039600	-1.75705900
C	8.82852100	0.94173100	0.46422800
H	8.12520300	2.37415600	1.88303600
O	9.51474400	-0.62597300	-1.12662400
H	9.19838000	-1.21272400	-1.82667800
O	10.12890400	1.06918100	0.84654600
H	10.67317500	0.48049300	0.30339300
C	1.79058700	0.83808700	-0.75128000

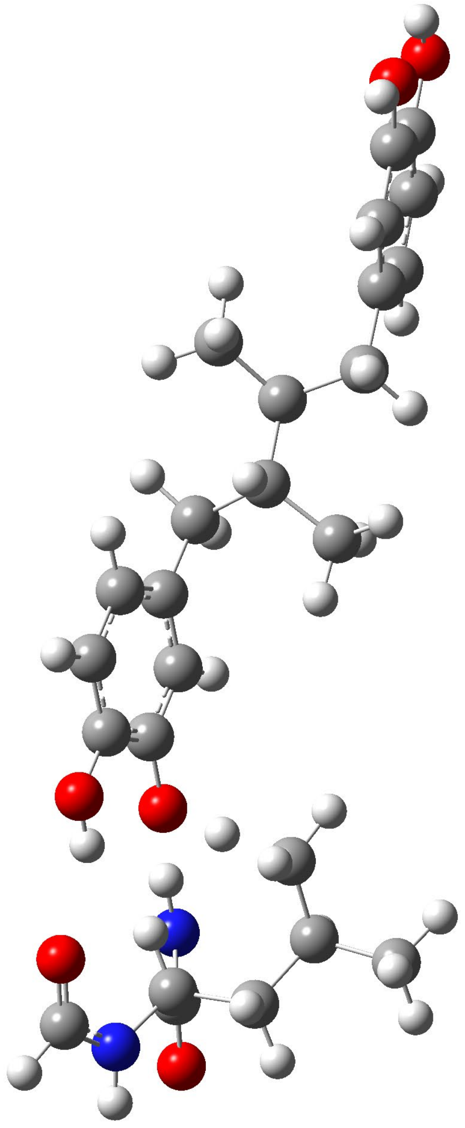

H	2.26990900	1.10210100	-1.69734000
H	0.72330900	0.70210300	-0.95357900
H	1.89600200	1.68950200	-0.06597000
C	4.50623700	-1.63102100	0.57428000
H	5.50640900	-1.51959200	1.00280700
H	4.60539400	-2.18925400	-0.36594900
H	3.92159300	-2.24403700	1.26514800
N	-5.30807800	1.52778700	2.17065100
H	-4.49921900	0.95353400	1.95996100
H	-5.30072500	2.10256000	3.00322700
C	-6.43666900	1.44415400	1.43929400
O	-7.47529700	2.04112600	1.70594400
C	-6.33862100	0.56622700	0.19448100
H	-5.48515900	-0.10357700	0.28172200
N	-7.50806700	-0.28843600	0.13743300
H	-8.41822000	0.10750300	0.34251200
C	-7.40092300	-1.58744900	-0.18537900
O	-6.34151000	-2.15599800	-0.44844400
H	-8.36266100	-2.12027500	-0.19594200
C	-6.20677600	1.42482600	-1.07391000
H	-7.03865900	2.13914700	-1.09962900
H	-6.31975900	0.75600700	-1.93720600
C	-4.87134000	2.16800600	-1.19145000
H	-4.70874500	2.77406800	-0.28965800
C	-4.91192900	3.12654800	-2.39600600
H	-5.08454200	2.56972300	-3.32364800
H	-3.97051900	3.67438100	-2.49550500
H	-5.72206900	3.85318300	-2.27457300
C	-3.70970500	1.22024000	-1.36606100
H	-3.84425800	0.44756200	-2.13075700
H	-2.71808300	1.68046100	-1.39169700

16$^{(1)}$-δ-TS

Charge=0, Multiplicity=2

C	2.97539100	0.94343400	0.27456100
C	2.98089900	-0.44764800	0.07743800
C	4.15707600	-1.15361500	-0.54875500
H	4.69126300	-1.73079200	0.21746900
H	3.79334200	-1.88021100	-1.28172800
C	5.10266400	-0.14223200	-1.19360500
H	4.68727200	0.22212400	-2.14041300
H	6.07444400	-0.59661600	-1.40708700
O	4.04664800	1.72099600	-0.04503000
C	1.85546300	1.61360900	0.77145900
H	1.86855000	2.69225900	0.88638300
C	0.72783400	0.86961000	1.12732000
C	1.82720400	-1.16196200	0.44344000
C	5.28503900	1.03568000	-0.25560500
H	5.96309800	1.78853700	-0.66103300
H	5.67021300	0.69897500	0.71723900
C	0.72031000	-0.52502500	0.99311000
H	-0.14386700	-1.10511300	1.29689600
O	1.83038300	-2.50164600	0.22723300
H	0.90874500	-2.82071500	0.21613000
O	-0.37642400	1.48386600	1.60972800

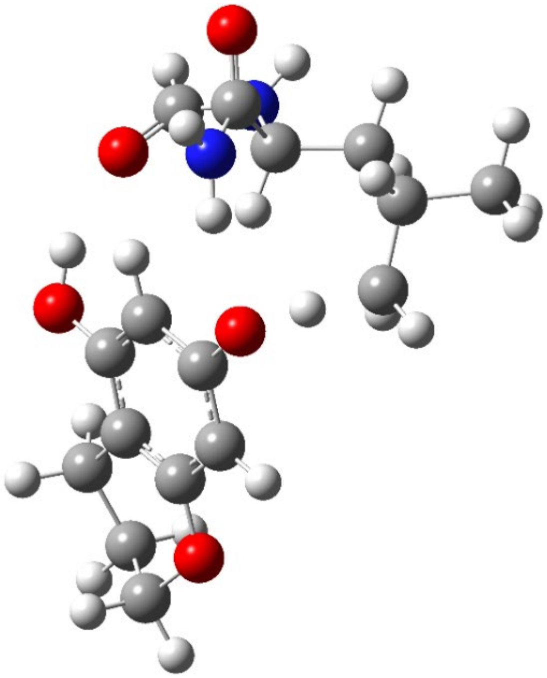

H	-1.01264300	1.94475400	0.69700800
N	-2.76270800	-0.26007600	1.94802900
H	-1.90656300	0.28828800	1.95219400
H	-3.25881000	-0.39904400	2.81860000
C	-3.25551500	-0.82728700	0.82963300
O	-4.27163700	-1.51203700	0.80522700
C	-2.48589000	-0.51660600	-0.46737300
H	-1.44323600	-0.27440400	-0.23901700
N	-2.48597400	-1.68705400	-1.32776100
H	-3.20601300	-1.76670700	-2.03595400
C	-1.74814300	-2.77529200	-1.06413100
O	-0.95006300	-2.85977300	-0.12999400
H	-1.90794200	-3.60074700	-1.77280300
C	-3.14209300	0.65116900	-1.21335100
H	-4.19500100	0.39806800	-1.39213600
H	-2.65277600	0.73599000	-2.19265000
C	-3.05970500	2.01012700	-0.50945700
H	-3.53584000	1.94076400	0.47815300
C	-3.84651300	3.05083600	-1.32644000
H	-3.40106400	3.17770000	-2.31958200
H	-3.84675700	4.02266300	-0.82516900
H	-4.88563700	2.73003600	-1.45392300
C	-1.63651500	2.49700300	-0.34503200
H	-0.94996800	2.23971100	-1.15942000
H	-1.54632600	3.54231700	-0.03760500

16(2)-δ-TS

Charge=0, Multiplicity=2

C	3.14523400	-0.68942900	-0.21675200
C	2.40574100	0.32572400	0.40314100
C	2.96208600	1.71593400	0.58405800
H	2.49737500	2.40497100	-0.13481700
H	2.70274100	2.08571200	1.58076100
C	4.47550700	1.69970600	0.37588900
H	4.97410400	1.24603000	1.24018900
H	4.86667000	2.71433200	0.25722000
O	4.39959200	-0.48138300	-0.70526000
C	2.63788900	-1.98470800	-0.37414900
H	3.22847700	-2.73568600	-0.88799600
C	1.36402000	-2.28826000	0.10236400
C	1.12569700	-0.01916500	0.87918100
C	4.79883200	0.88348900	-0.86183300
H	5.87051900	0.84664700	-1.06447200
H	4.28715100	1.29955900	-1.74155900
C	0.61193400	-1.31334400	0.75613600
H	-0.36993400	-1.55352200	1.14840700
O	0.36352900	0.92128200	1.48141700
H	-0.11380100	1.64899600	0.65011800
O	0.87577700	-3.53812800	-0.09807900
H	-0.09643300	-3.50779700	-0.03285800
N	-2.45605600	0.14028700	2.01638400
H	-1.46160200	0.34267100	1.95895600
H	-2.90524700	0.14267600	2.92282700
C	-3.19630600	-0.15454900	0.92985600
O	-4.39095300	-0.42445900	0.97240000

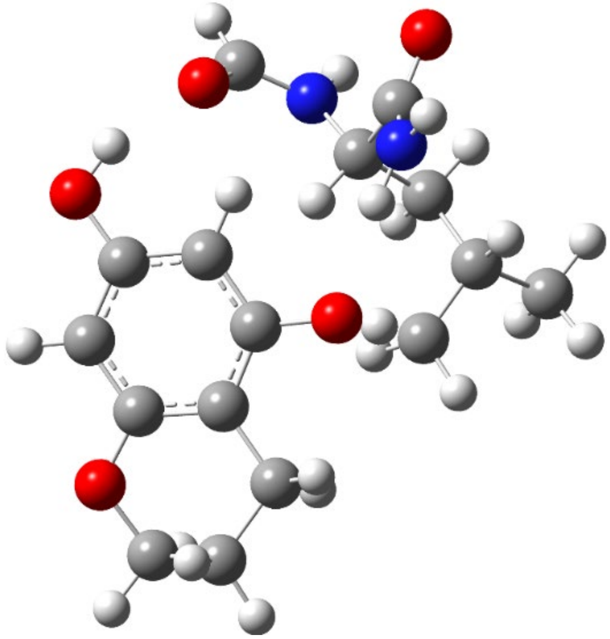

C	-2.45463900	-0.07667400	-0.41744700
H	-1.38178200	-0.23285500	-0.26744400
N	-2.93237200	-1.12704200	-1.29975600
H	-3.67196500	-0.90860800	-1.95683900
C	-2.63090900	-2.42011500	-1.10820700
O	-1.86767500	-2.82946800	-0.23312900
H	-3.12233900	-3.09930800	-1.81977700
C	-2.69742800	1.28286900	-1.08330500
H	-3.78066900	1.42682100	-1.18508300
H	-2.27560500	1.23752700	-2.09608900
C	-2.09408800	2.49027200	-0.35710600
H	-2.48713700	2.53655400	0.66760000
C	-2.53019000	3.78040500	-1.07534400
H	-2.14740300	3.79799400	-2.10200800
H	-2.15388800	4.66516300	-0.55445600
H	-3.62254200	3.84400100	-1.11463800
C	-0.58161000	2.45228700	-0.31105500
H	-0.09362500	2.03182200	-1.19778000
H	-0.11684700	3.38419100	0.02201300

17$^{(1)}$-δ-TS

Charge=0, Multiplicity=2

C	4.78500900	0.35315900	0.22569900
C	3.86722300	1.33476500	0.59157400
C	2.50889500	1.14586800	0.32610000
C	2.09071600	-0.03687500	-0.31276000
C	3.03716000	-1.01700200	-0.66897000
C	4.38839700	-0.82682500	-0.40691200
H	5.83837500	0.51110900	0.43618900
H	4.18202700	2.25071500	1.07978300
H	5.10332000	-1.58985800	-0.69411400
O	1.63922100	2.09885000	0.70308800
H	0.72583800	1.95937100	0.38713300
O	2.61515500	-2.15625000	-1.26731900
H	1.65851100	-2.08661800	-1.41177200
O	0.78665500	-0.25435300	-0.59782200
H	0.26109000	-0.76725000	0.30103600
N	-1.76577100	-0.88629200	-2.17883900
H	-0.79191600	-0.67488000	-1.99762000
H	-2.01044800	-1.38523100	-3.02443400
C	-2.74647600	-0.35215500	-1.42277700
O	-3.94236300	-0.47007300	-1.66695300
C	-2.27263900	0.40252900	-0.18203800
H	-1.20536300	0.60439300	-0.25740900
N	-2.91775500	1.70093900	-0.15710400
H	-3.91517700	1.75955400	-0.32797000
C	-2.22105200	2.81978100	0.10142200
O	-1.01164300	2.85328600	0.32628500
H	-2.83424300	3.73232700	0.09550700
C	-2.56939000	-0.39932400	1.09333600
H	-3.63887400	-0.64091700	1.11155100
H	-2.36239700	0.25322600	1.95151400
C	-1.73915000	-1.68041300	1.22783700
H	-1.86707800	-2.29664400	0.32693900
C	-2.23969800	-2.50424400	2.42944700

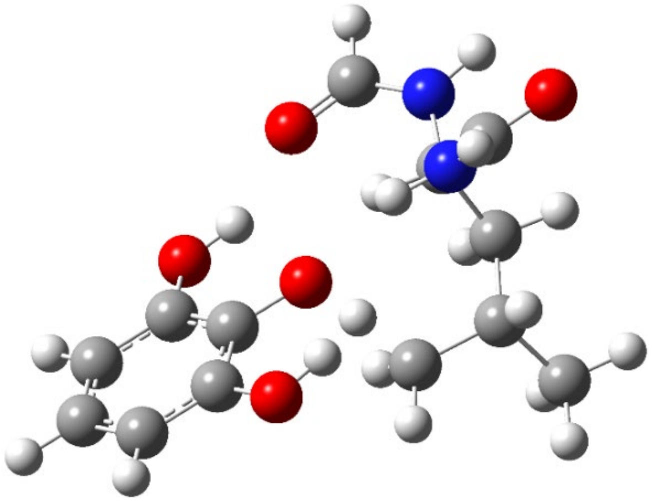

H	-2.14430300	-1.92742900	3.35589400
H	-1.66608600	-3.42873400	2.54031300
H	-3.29397100	-2.76673200	2.29338600
C	-0.27252500	-1.38806900	1.42470800
H	-0.03751200	-0.62939700	2.17895500
H	0.38785600	-2.25836600	1.46850100

17[(2)]-δ-TS

Charge=0, Multiplicity=2

C	-4.20282500	-1.11942700	1.04005000
C	-4.41406600	-0.00886700	0.21823700
C	-3.33845600	0.59030600	-0.43724200
C	-2.03792800	0.10260900	-0.25423200
C	-1.83449400	-1.00204300	0.59778400
C	-2.92617500	-1.63410000	1.21690400
H	-5.04870900	-1.58256300	1.53783200
H	-5.41617400	0.38657700	0.07233700
H	-2.73786200	-2.49349400	1.85171800
O	-3.47318400	1.66082200	-1.26450000
H	-4.39937000	1.93659400	-1.29248100
O	-0.58686400	-1.42102500	0.86391300
H	0.03974300	-1.65640700	-0.17589200
O	-0.98664200	0.59953300	-0.95101100
H	-0.72973200	1.51591200	-0.68602900
N	2.04826500	-1.00795800	2.13313600
H	1.06915300	-1.21050400	1.96239200
H	2.54073100	-1.50700500	2.86215200
C	2.69322900	-0.05276400	1.44136600
O	3.86138700	0.27172600	1.64046600
C	1.88426300	0.61135800	0.32740900
H	0.81601800	0.49488200	0.51228700
N	2.20856000	2.02999600	0.36373900
H	3.16543700	2.26871100	0.60483600
C	1.34880500	3.02031300	0.10684600
O	0.16026200	2.88756800	-0.20139000
H	1.79816400	4.01881900	0.20112700
C	2.25996000	0.01704200	-1.04491500
H	3.30673300	0.28532100	-1.23462100
H	1.64659700	0.52294300	-1.80123100
C	2.10755600	-1.50288400	-1.19827600
H	2.60866300	-2.00470300	-0.36013600
C	2.81399100	-1.95055100	-2.49224300
H	2.37165500	-1.45310800	-3.36255500
H	2.72639600	-3.03150800	-2.63537500
H	3.87787200	-1.69436300	-2.45255000
C	0.67148300	-1.97309300	-1.26767500
H	0.06208000	-1.46708600	-2.02125900
H	0.55309000	-3.05965200	-1.28458300

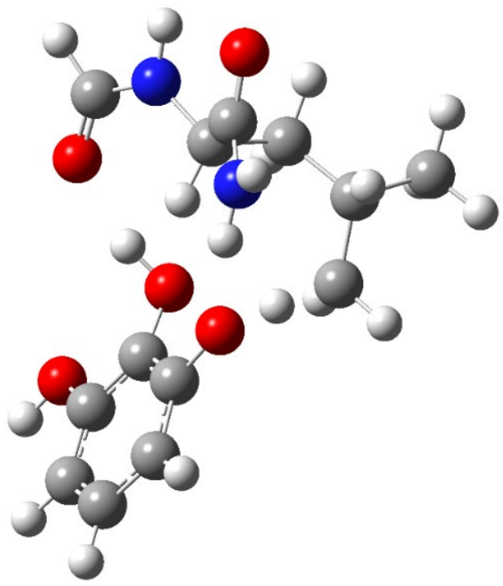

18[(1)]-δ-TS

Charge=0, Multiplicity=2

N	-2.43103100	-1.83210700	-1.85144600
H	-1.51256900	-1.45911300	-1.63947400
H	-2.51083000	-2.59251600	-2.51414700
C	-3.55122900	-1.24988200	-1.38052500
O	-4.68786200	-1.59297100	-1.69008900
C	-3.32324300	-0.12629000	-0.37298300
H	-2.30645500	0.25037400	-0.46500900
N	-4.19219500	0.98241100	-0.71571600
H	-5.14925000	0.78942400	-0.98894100
C	-3.75177900	2.24754000	-0.67593100
O	-2.60759100	2.58023600	-0.36035500
H	-4.51110400	2.99123500	-0.95502900
C	-3.57897400	-0.61393800	1.06286300
H	-4.58179200	-1.05585500	1.10631500
H	-3.57982600	0.26916400	1.71515800
C	-2.54096600	-1.61478800	1.58276400
H	-2.46562100	-2.46155700	0.88736400
C	-2.99000500	-2.16990400	2.94731500
H	-3.09369400	-1.35883200	3.67641900
H	-2.26606500	-2.89104200	3.33712200
H	-3.95808200	-2.67202000	2.85001000
C	-1.17814300	-0.98621400	1.74737300
H	-1.16543200	-0.03705400	2.29420200
H	-0.37657300	-1.66428800	2.05258900
C	3.51077000	1.46442700	0.22034200
C	2.36923500	2.24150300	0.31051000
C	1.10397700	1.67004000	0.08850600
C	1.00571300	0.30501800	-0.23459600
C	2.16197300	-0.48104400	-0.31704900
C	3.40549700	0.10111200	-0.09584300
H	4.48373600	1.91277100	0.39130100
H	2.06390200	-1.53290000	-0.56101700
O	-0.21604400	-0.21206800	-0.46783300
H	-0.69392600	-0.59997900	0.55413900
O	0.05727400	2.50152900	0.21542800
H	-0.80931200	2.13620400	-0.06148800
O	2.46309200	3.55853200	0.61903800
H	1.57356900	3.94438800	0.61218100
C	4.66523000	-0.68634600	-0.17891700
O	5.77204000	-0.22357100	0.00339200
O	4.45386500	-1.97454200	-0.47816500
C	5.62843500	-2.78796800	-0.57702300
H	6.17035200	-2.79045900	0.37110400
H	5.27297600	-3.78943100	-0.81465200
H	6.28143800	-2.41739800	-1.37019000

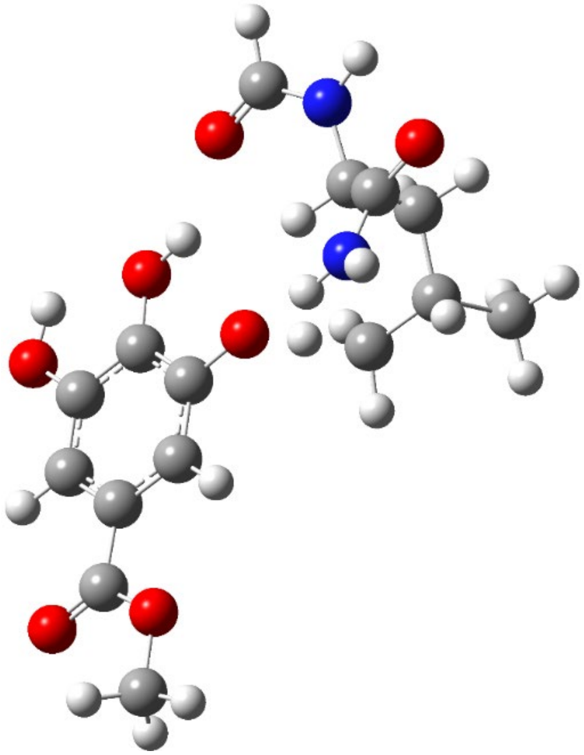

18(2)-δ-TS

Charge=0, Multiplicity=2

N	-3.09405200	1.01482100	2.08679800
H	-2.10703700	0.82599800	1.96252200
H	-3.39194200	1.55665500	2.88793500
C	-4.02275900	0.39033500	1.33373800
O	-5.23087500	0.47542000	1.52452700
C	-3.47144500	-0.42549000	0.16639300
H	-2.40192100	-0.58193300	0.29670100
N	-4.06932000	-1.74596000	0.20369200
H	-5.06824500	-1.83193500	0.35307900
C	-3.32670700	-2.84978100	0.02188500
O	-2.11163000	-2.84879300	-0.17473500
H	-3.90511700	-3.78354000	0.06459500
C	-3.74254400	0.27835800	-1.17154200
H	-4.81585100	0.49173900	-1.24121400
H	-3.49266800	-0.42747900	-1.97420700
C	-2.93611000	1.56610200	-1.37141200
H	-3.10249900	2.24105000	-0.52029200
C	-3.41604100	2.29216000	-2.64270800
H	-3.28032100	1.65394200	-3.52259400
H	-2.85895300	3.21965100	-2.80171000
H	-4.47921200	2.53872700	-2.55568200
C	-1.45879100	1.29411800	-1.50825100
H	-1.18740000	0.48719700	-2.19742000
H	-0.81620400	2.17385400	-1.60083700
C	2.71707400	-1.21939800	-0.30524100
C	1.34557900	-1.05521200	-0.11591200
C	0.86612400	0.15600900	0.42040700
C	1.76776500	1.18659200	0.75241600
C	3.13302000	1.02339100	0.56730400
C	3.59037000	-0.18713400	0.03538700
H	3.09642400	-2.14902900	-0.71418500
H	3.81510700	1.82227200	0.83077800
O	1.28898100	2.34931500	1.25080000
H	0.32836800	2.26383600	1.35633100
O	-0.45075500	0.35172900	0.63114000
H	-0.95785600	0.78133500	-0.33687200
O	0.52383300	-2.05633800	-0.46617800
H	-0.40971100	-1.92615200	-0.20841600
C	5.04825500	-0.42337300	-0.19273500
O	5.50653400	-1.44362800	-0.66088400
O	5.80411000	0.61655500	0.17529600
C	7.21466100	0.45821000	-0.01822400
H	7.43670600	0.29646400	-1.07519100
H	7.66434100	1.38773900	0.32716100
H	7.58530000	-0.38582600	0.56732700

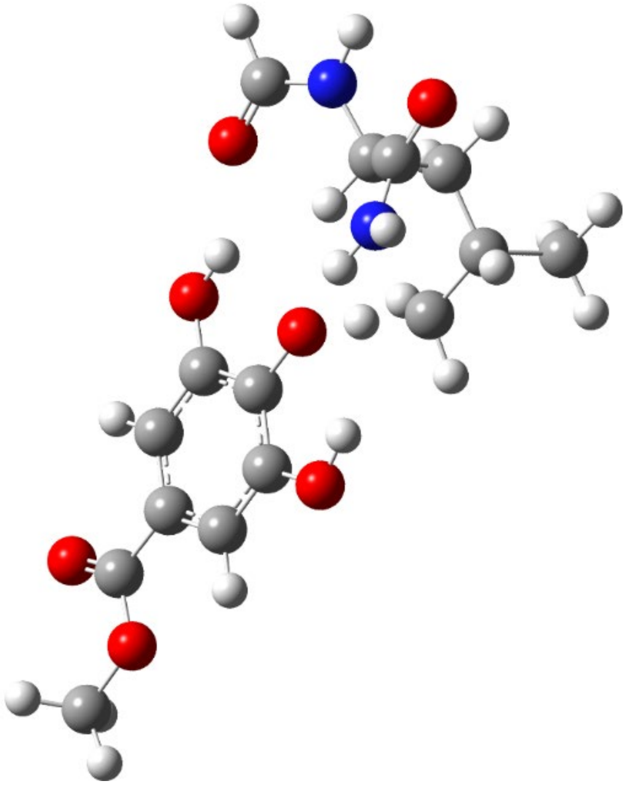

18(3)-δ-TS

Charge=0, Multiplicity=2

C	-2.05195300	-0.65665200	0.41278900
C	-0.93999200	0.18000400	0.26701900
C	-1.11537400	1.51643700	-0.13496900
C	-2.41155400	2.00520700	-0.37160800
C	-3.51130100	1.17711500	-0.21962300
C	-3.32765700	-0.15769600	0.17604700
H	-1.90748700	-1.68816300	0.71657400
H	-4.50274400	1.57408400	-0.40561500
O	-2.57991800	3.29501200	-0.75638400
H	-1.71231600	3.72703600	-0.78498500
O	-0.11556700	2.39241400	-0.32573300
H	0.77139800	2.08908400	-0.03834500
O	0.31046200	-0.25597800	0.51130800
H	0.79130700	-0.69114700	-0.49212500
C	-4.48045300	-1.08248500	0.35026800
O	-4.37870400	-2.24555100	0.68242500
O	-5.65714300	-0.49594400	0.09942300
C	-6.81161400	-1.33062200	0.24562900
H	-6.88718800	-1.69866800	1.27106500
H	-7.66406100	-0.69714800	0.00536600
H	-6.75870900	-2.17611400	-0.44364100
N	2.61399800	-1.68762200	1.94380100
H	1.67537400	-1.37239900	1.72673000
H	2.74014700	-2.40476200	2.64640200
C	3.69776900	-1.08275100	1.41971900
O	4.85421800	-1.35505600	1.72650600
C	3.40150000	-0.03013100	0.35470200
H	2.36912200	0.30219300	0.44190100
N	4.21921800	1.13718900	0.62063600
H	5.18930700	1.00687000	0.88473700
C	3.71485000	2.37523300	0.52613800
O	2.54987200	2.63517000	0.21804600
H	4.44087900	3.16866200	0.75141700
C	3.66127400	-0.58585900	-1.05520500
H	4.68590900	-0.97521000	-1.09201900
H	3.60381700	0.25635900	-1.75716900
C	2.67176100	-1.67022700	-1.49617600
H	2.65652600	-2.47904600	-0.75342600
C	3.12633000	-2.27632600	-2.83697500
H	3.16995100	-1.50371000	-3.61246200
H	2.43767600	-3.05799000	-3.16981700
H	4.12320900	-2.71697700	-2.73281000
C	1.27281900	-1.12861800	-1.66695900
H	1.19685300	-0.21880200	-2.27249100
H	0.50432500	-1.86743300	-1.90982800

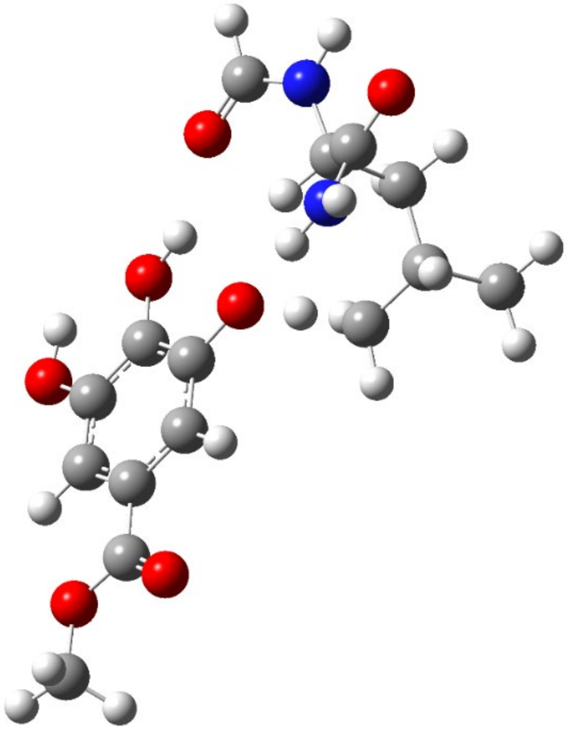

19^(1)-δ-TS

Charge=0, Multiplicity=2

N	-4.11024200	-0.09182700	1.81569400
H	-3.46834600	-0.58769600	1.20564700
H	-3.85223300	0.07315100	2.77953200
C	-5.37112100	0.17783100	1.42526800
O	-6.21552400	0.68967200	2.15175000
C	-5.68666000	-0.14254700	-0.04797000
H	-4.95252600	-0.85906400	-0.43285300
N	-6.99671400	-0.75663200	-0.14488200
H	-7.79805200	-0.16302000	-0.32050100
C	-7.22218900	-1.99898500	0.33568700
O	-6.34654600	-2.73559800	0.76748400
H	-8.28047600	-2.30151200	0.28310500
C	-5.68048200	1.14438100	-0.88096100
H	-6.38612600	1.84880200	-0.42251000
H	-6.06264100	0.90006200	-1.88096100
C	-4.31355300	1.81939100	-1.03140400
H	-3.88579500	2.00900300	-0.03688600
C	-4.48715000	3.18046400	-1.72964500
H	-4.91415300	3.04751200	-2.73004100
H	-3.52753200	3.69460500	-1.83309600
H	-5.16019700	3.82125900	-1.15054300
C	-3.34788100	0.98291800	-1.83620500
H	-3.76873600	0.50768500	-2.72784300
H	-2.37445400	1.44728800	-2.02279700
C	-1.08742100	-0.64878700	-0.32940900
C	-0.14327700	-1.36819600	-1.08247900
C	1.21066900	-1.12646500	-0.91727700
C	1.66709100	-0.14925100	-0.01174500
C	0.70983200	0.58151700	0.71427600
C	-0.64861200	0.33899000	0.56597100
H	-0.49771300	-2.11654800	-1.78477100
H	1.92166000	-1.69330500	-1.51024200
H	1.04623800	1.34715400	1.40861700
H	-1.38305200	0.90656000	1.13194700
O	-2.39976800	-0.91902700	-0.46849500
H	-2.92333800	-0.05882800	-1.12003100
C	3.08711700	0.14879600	0.20278300
H	3.27767100	1.01567200	0.83351400
C	4.12528300	-0.55173000	-0.28600300
H	3.93820800	-1.44183200	-0.88421000
C	5.54839400	-0.24474400	-0.07350700
C	5.98165200	0.92666600	0.56443700
C	6.49194600	-1.17097100	-0.53403000
C	7.34289500	1.14825900	0.74054700
H	5.28494300	1.67688500	0.92106400
C	7.85233600	-0.93095000	-0.34779500
H	6.17909000	-2.08205100	-1.03424800
C	8.29345100	0.22668900	0.29118600
H	9.35588500	0.41339900	0.43345400
O	7.71049700	2.30084000	1.36402000
H	8.67350100	2.35335100	1.43012400
O	8.71914600	-1.87007900	-0.81382100
H	9.63050800	-1.60544100	-0.62899600

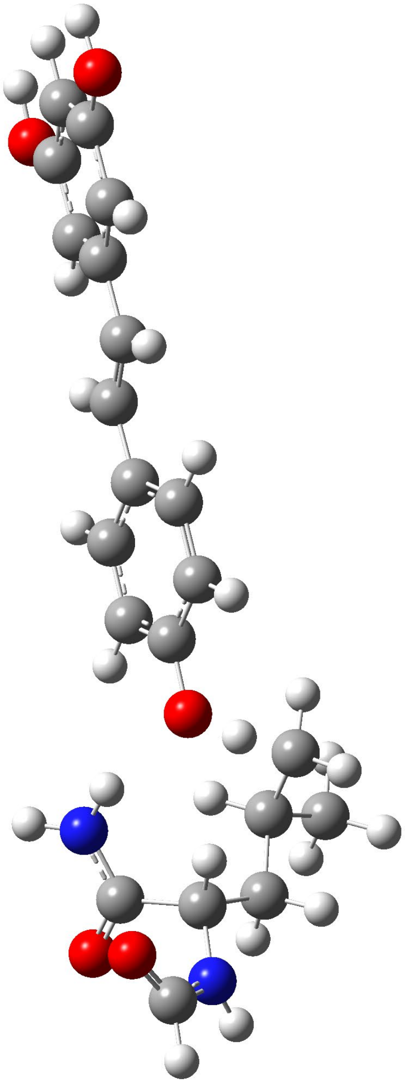

19(2)-δ-TS

Charge=0, Multiplicity=2

C	7.79715500	-0.48062700	0.35485900
C	7.07850100	0.52332300	1.01231700
C	5.72159900	0.67291200	0.77021400
C	5.04309700	-0.16529500	-0.13248100
C	5.78490700	-1.16612100	-0.77432300
C	7.14767400	-1.32915700	-0.54073100
H	7.59705900	1.17219000	1.71103400
H	5.18348100	1.45479000	1.29753500
H	5.28549100	-1.83111200	-1.47450400
H	7.70427300	-2.11104800	-1.05168900
O	9.12450700	-0.58434100	0.63227300
H	9.51462100	-1.30688500	0.12139600
C	3.60942800	-0.04249500	-0.42819300
H	3.21457000	-0.81677100	-1.08527000
C	2.78340500	0.92074200	0.01312100
H	3.17597500	1.72589500	0.63187100
C	1.34692700	1.02188500	-0.29290200
C	0.61417100	-0.03740800	-0.84806800
C	0.68993600	2.22448400	0.00968500
C	-0.74178200	0.13997900	-1.14029700
H	1.06602600	-1.00451900	-1.04409700
C	-0.66981800	2.38864200	-0.27215000
H	1.23169400	3.04298900	0.47488100
C	-1.38199300	1.36026800	-0.88223500
H	-2.42833800	1.49605900	-1.13314500
O	-1.46738100	-0.87234900	-1.66749400
H	-1.79187300	-1.63980500	-0.78338800
O	-1.26348500	3.55905700	0.06505800
H	-2.22901500	3.42562400	0.10115900
N	-4.40675600	-0.42758000	-1.87134000
H	-3.39419200	-0.50316100	-1.91774300
H	-4.94144700	-0.49197300	-2.72783900
C	-5.06837500	-0.24727800	-0.71149400
O	-6.28679200	-0.14228600	-0.63244100
C	-4.19463200	-0.23181900	0.55618300
H	-3.17069300	0.06130200	0.30488800
N	-4.71218300	0.74833500	1.49506100
H	-5.37871200	0.44259300	2.19418600
C	-4.54578600	2.06830500	1.32871000
O	-3.88396200	2.57293200	0.42080000
H	-5.04922600	2.67754500	2.09314000
C	-4.19388600	-1.61218100	1.22401000
H	-5.23391800	-1.89593000	1.42964100
H	-3.68237500	-1.51244400	2.19031400
C	-3.51281000	-2.72849100	0.42468900
H	-4.00729600	-2.83679700	-0.55033900
C	-3.68011600	-4.06195500	1.17541200
H	-3.18214200	-4.01970800	2.15057200
H	-3.24713200	-4.88873000	0.60564800
H	-4.74108100	-4.27661800	1.33995800
C	-2.03588100	-2.47709300	0.21103100
H	-1.51625200	-1.97890500	1.03738900

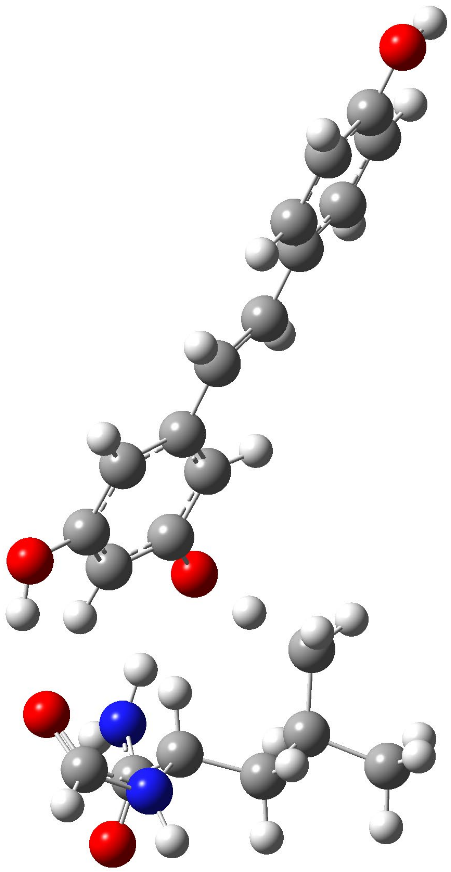

H	-1.48033400	-3.33768100	-0.17132000

19(3)-δ-TS

Charge=0, Multiplicity=2

C	-7.88341200	-0.59478200	-0.36309200
C	-6.86869400	-1.55657700	-0.39881300
C	-5.54528200	-1.17014300	-0.24701700
C	-5.19529900	0.17726400	-0.04998200
C	-6.22940300	1.12275900	-0.03067600
C	-7.56206200	0.75006100	-0.18108600
H	-7.13338800	-2.59755100	-0.55568700
H	-4.77182000	-1.93082800	-0.29699800
H	-5.98611800	2.17280900	0.11062400
H	-8.34834800	1.50063300	-0.15722100
O	-9.16320200	-1.02811600	-0.52057000
H	-9.77391300	-0.27920100	-0.48332600
C	-3.81027600	0.63346100	0.12827500
H	-3.67642800	1.71321900	0.07011200
C	-2.74341600	-0.14423700	0.37709600
H	-2.87672500	-1.21774000	0.50296500
C	-1.35985600	0.33027500	0.54366600
C	-0.95259300	1.61320900	0.13927700
C	-0.41818500	-0.54190100	1.10336400
C	0.36622600	2.02816600	0.32773000
H	-1.64029700	2.29141600	-0.35530900
C	0.90084400	-0.11302100	1.30138300
H	-0.69800300	-1.55002500	1.39625200
C	1.29035000	1.18129100	0.94033500
H	2.30738600	1.51850400	1.10946000
O	0.72593500	3.25987900	-0.10981000
H	1.69478600	3.28676400	-0.21869400
O	1.82092000	-0.94361600	1.84095200
H	2.21209300	-1.71975500	0.99236400
N	4.64602000	0.02494400	1.76226600
H	3.67330500	-0.23127100	1.90795000
H	5.24739700	0.13948100	2.56757900
C	5.16247500	0.24048500	0.53635900
O	6.32441700	0.57428800	0.33657400
C	4.20434000	-0.01813400	-0.64102200
H	3.16653100	0.10207900	-0.31469100
N	4.44828400	0.95257000	-1.69354300
H	5.07758400	0.70261800	-2.44714600
C	4.07990900	2.23722500	-1.58670400
O	3.44314600	2.69530800	-0.63700600
H	4.38835600	2.85964200	-2.43906000
C	4.41693300	-1.42852200	-1.20361600
H	5.47425700	-1.53314000	-1.47863500
H	3.82722500	-1.51062400	-2.12616700
C	4.02105000	-2.57626200	-0.26842100
H	4.59213600	-2.50116100	0.66698700
C	4.39214500	-3.91698100	-0.92744800
H	3.83480000	-4.05492100	-1.86080200
H	4.16249200	-4.75577100	-0.26458200
H	5.46203200	-3.94495600	-1.15846200
C	2.54155300	-2.58627300	0.04911700

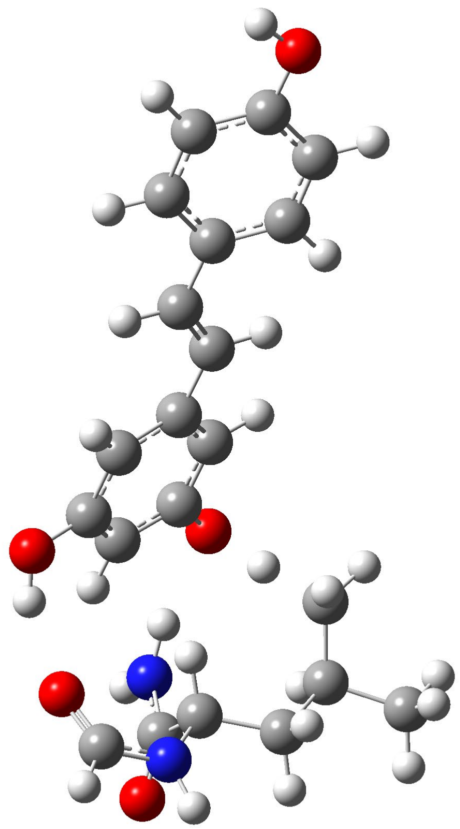

H	1.88053600	-2.27378500	-0.76720400
H	2.19019700	-3.49514800	0.54515200

20(1)-δ-TS

Charge=0, Multiplicity=2

C	-1.05237100	-2.16416600	-0.24169300
C	0.10775500	-2.89270300	-0.53233900
C	1.35458500	-2.29506500	-0.44889500
C	1.49911300	-0.94453200	-0.06720600
C	0.33832300	-0.22114200	0.21183300
C	-0.92782200	-0.81132400	0.13773700
H	0.00585200	-3.93261200	-0.82630600
H	2.23111500	-2.88960200	-0.68663800
H	0.39596000	0.82464000	0.50437700
O	-2.04545900	-0.11309600	0.42746400
H	-2.46258800	0.39859900	-0.54456700
O	-2.23911700	-2.78013000	-0.34837300
H	-2.99394900	-2.24329200	-0.03661500
C	2.79938000	-0.27076200	0.04473300
H	2.73845900	0.78584000	0.30221300
C	6.46184500	-0.93647000	-0.01184100
C	7.71289400	-0.33476900	0.11075800
C	7.83355200	1.04863800	0.23271200
C	6.67130200	1.82535600	0.22243500
C	5.41435400	1.24391600	0.09827500
C	5.30321400	-0.14991000	-0.01120500
H	6.40041500	-2.01652300	-0.10086200
H	8.81200200	1.51434200	0.32962700
H	4.54487200	1.89126800	0.07841500
C	4.00602700	-0.83639900	-0.12516700
H	4.07490100	-1.89912300	-0.35014000
O	6.72233100	3.18201900	0.32738900
H	7.64008200	3.47708900	0.39873200
O	8.80064300	-1.15244400	0.10731700
H	9.61010200	-0.63181600	0.19876900
N	-3.86331400	1.79084100	1.95511500
H	-3.04909600	1.24266800	1.70081700
H	-3.76961300	2.51811600	2.65213700
C	-5.08740100	1.49088600	1.47830200
O	-6.11811400	2.06070400	1.82290200
C	-5.11904600	0.39093700	0.42014700
H	-4.19414400	-0.18141500	0.45683800
N	-6.17537300	-0.54486400	0.75278900
H	-7.07697000	-0.18763200	1.04781700
C	-5.97877200	-1.86996200	0.66238200
O	-4.92310600	-2.39648600	0.31067800
H	-6.86042100	-2.46730000	0.93604000
C	-5.31626500	0.98558800	-0.98322100
H	-6.21747700	1.61061800	-0.97312100
H	-5.50118100	0.15089300	-1.67206500
C	-4.11964700	1.79657200	-1.49293100
H	-3.87072100	2.58032900	-0.76444600
C	-4.48670200	2.49094200	-2.81758400
H	-4.75502300	1.74902800	-3.57777900
H	-3.64942900	3.08331500	-3.19737900

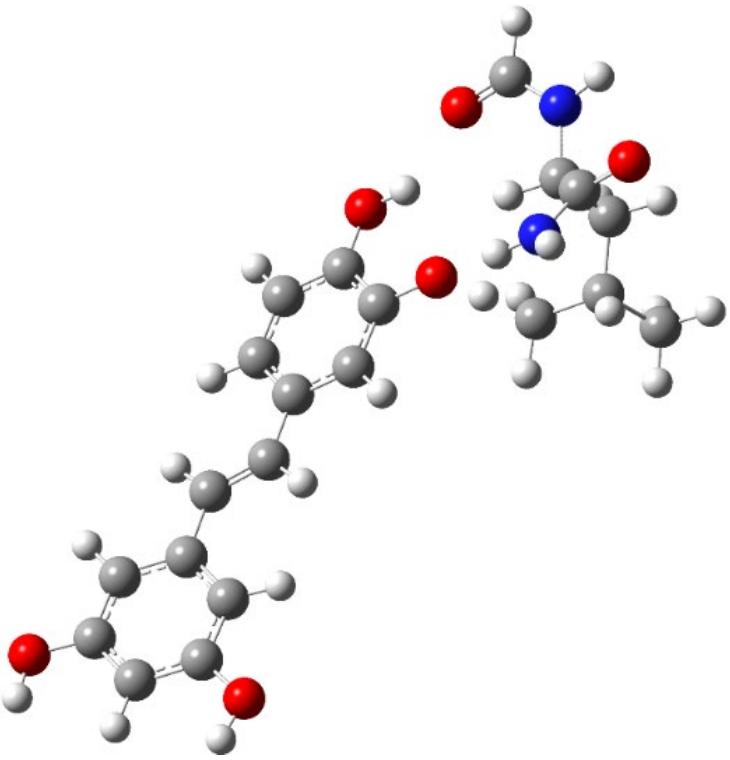

H	-5.34214200	3.15846600	-2.67033000
C	-2.90442700	0.93130400	-1.72311600
H	-3.08229300	0.01798200	-2.30109800
H	-1.99468400	1.45815400	-2.02346800

20[(2)]-δ-TS

Charge=0, Multiplicity=2

C	1.06205900	0.84705600	0.27484600
C	0.37989900	-0.33707300	0.58373900
C	-1.00381100	-0.38047000	0.54003600
C	-1.74325500	0.76525000	0.18011100
C	-1.05869800	1.95078300	-0.12228200
C	0.33020500	1.99437600	-0.08822500
H	0.95838900	-1.21519300	0.86047200
H	-1.51345600	-1.30235100	0.79841700
H	-1.60595000	2.84814600	-0.39692300
O	0.97874600	3.13726200	-0.39912800
H	1.93025500	2.98591600	-0.27545300
O	2.40700900	0.94957200	0.32058400
H	2.88601300	0.48919300	-0.65446500
C	-3.20996700	0.77748200	0.10664600
H	-3.64899600	1.76198500	-0.04576100
C	-6.13923500	-1.51867200	0.00878300
C	-7.52958100	-1.55885300	-0.08222700
C	-8.28136600	-0.38482300	-0.07064900
C	-7.61302300	0.83838100	0.03899300
C	-6.22692100	0.89898700	0.13060100
C	-5.48011400	-0.28739700	0.10805600
H	-5.58069100	-2.44925800	-0.00443500
H	-9.36689100	-0.41775500	-0.14042500
H	-5.75677500	1.87081400	0.22874300
C	-4.01062100	-0.29904200	0.18495700
H	-3.57075500	-1.28888600	0.29349100
O	-8.29037000	2.01835000	0.06689800
H	-9.24203400	1.86001100	0.00524100
O	-8.11053300	-2.78500100	-0.18235600
H	-9.07168100	-2.69607900	-0.23810800
N	3.88122600	-0.99377300	1.99082200
H	3.27007300	-0.29536000	1.57828200
H	3.56227300	-1.52723200	2.78905500
C	5.16424500	-1.10904700	1.60453000
O	5.97753400	-1.85407300	2.14553200
C	5.57057400	-0.27735000	0.38648500
H	4.95412300	0.62501800	0.31074800
N	6.94474800	0.13649800	0.59910900
H	7.52275200	-0.44852800	1.19232600
C	7.47868200	1.20068900	-0.02919200
O	6.86584600	1.93177800	-0.79894800
H	8.53889700	1.36376300	0.22258500
C	5.45571600	-1.11119900	-0.90572100
H	5.99917000	-2.05104400	-0.74950300
H	5.98258000	-0.55515200	-1.69115700
C	4.03275300	-1.40593000	-1.38928500
H	3.47142300	-1.92470300	-0.59876600
C	4.08861000	-2.34798900	-2.60700300

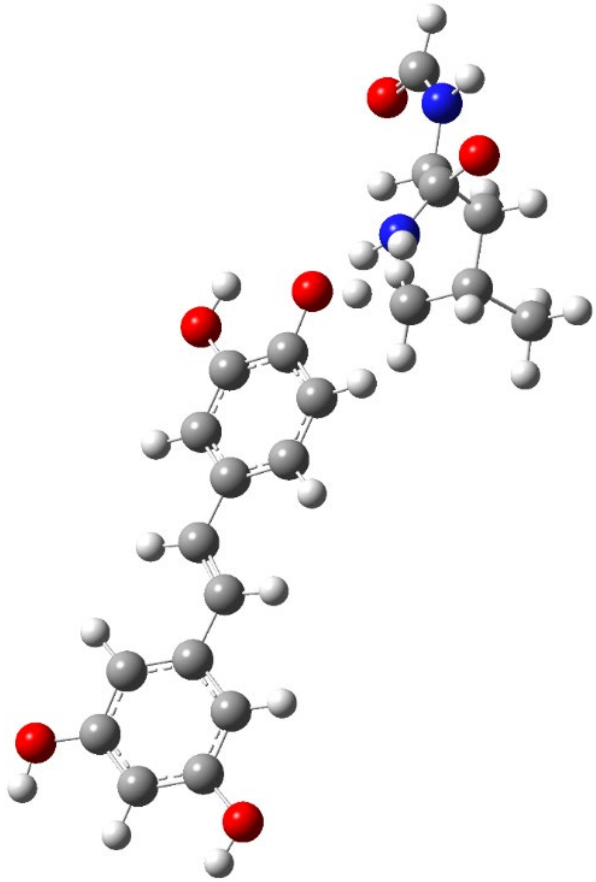

H	4.63279800	-1.87561700	-3.43214600
H	3.08298600	-2.59735300	-2.95715300
H	4.60280300	-3.27807000	-2.34326100
C	3.29472100	-0.15069300	-1.78332400
H	3.88640400	0.61106900	-2.29948800
H	2.30337300	-0.30230900	-2.22175000

20(3)-δ-TS

Charge=0, Multiplicity=2

C	7.49772300	0.11238900	-0.51949100
C	6.73684100	-0.87118500	-1.14131500
C	5.37140700	-0.96384100	-0.88560100
C	4.74857900	-0.07556800	0.00274000
C	5.52969400	0.91629000	0.61691100
C	6.89009000	1.01350600	0.36340500
H	7.21471200	-1.56178400	-1.83203200
H	4.79668800	-1.73307400	-1.39034900
H	5.07906500	1.62372200	1.30768100
O	7.62462300	1.98109100	0.97239200
H	8.54714200	1.90158000	0.68804300
O	8.83981300	0.29076200	-0.70302400
H	9.19208900	-0.36231600	-1.32290600
C	3.31479500	-0.12980300	0.32524000
H	2.95807700	0.69296200	0.94354800
C	0.31841600	-2.31995300	0.03791400
C	-1.04186400	-2.42680000	0.34379200
C	-1.71154000	-1.34948300	0.91627000
C	-1.02875400	-0.14055200	1.11182500
C	0.32797300	-0.02083000	0.79481000
C	1.01769900	-1.12723800	0.27813600
H	0.82790300	-3.17532000	-0.39661900
H	-2.75767700	-1.44023700	1.18757600
H	0.81342500	0.93830900	0.94362700
C	2.45239400	-1.08861800	-0.04992500
H	2.81091600	-1.93955500	-0.62683400
O	-1.71338900	0.91643900	1.60443700
H	-2.02755100	1.65706000	0.69298800
O	-1.67771400	-3.59097800	0.06717900
H	-2.63886600	-3.42772800	0.03777300
N	-4.65865900	0.57367400	1.88155200
H	-3.64362000	0.62065200	1.90836400
H	-5.17631000	0.68852000	2.74315700
C	-5.34526300	0.36557600	0.74106900
O	-6.56742000	0.29403900	0.68606100
C	-4.49573000	0.27348400	-0.53982500
H	-3.47556700	-0.03817600	-0.29574000
N	-5.05875500	-0.72696900	-1.42994300
H	-5.72772400	-0.42830600	-2.12981900
C	-4.93168300	-2.04389600	-1.21250000
O	-4.27066500	-2.53299900	-0.29557100
H	-5.46757500	-2.66689300	-1.94306800
C	-4.46975900	1.62641100	-1.26086800
H	-5.50562300	1.93207300	-1.45623300
H	-3.98187500	1.47545200	-2.23277900
C	-3.73941600	2.75067800	-0.51847000

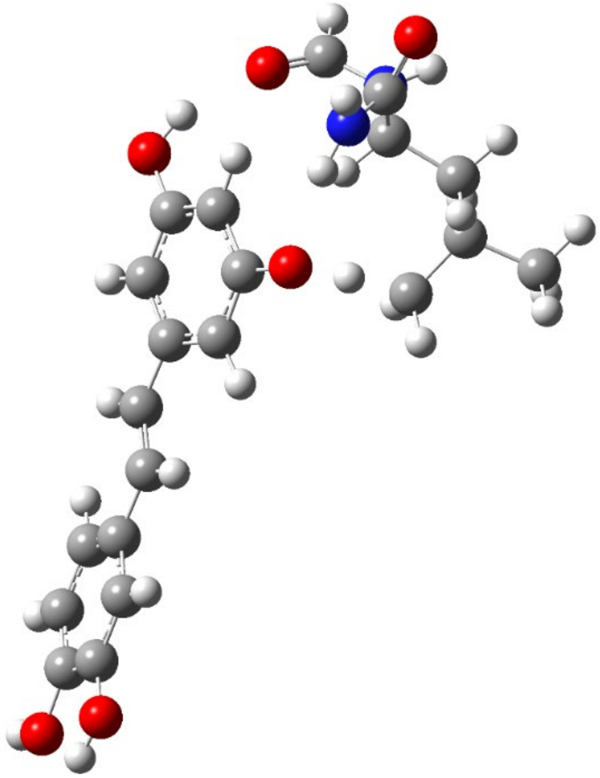

H	-4.20836500	2.90817200	0.46247400
C	-3.88572000	4.06044500	-1.31370100
H	-3.41380100	3.96761400	-2.29831800
H	-3.41427000	4.89378500	-0.78557100
H	-4.94368700	4.30114200	-1.46060100
C	-2.26601700	2.46251300	-0.32747200
H	-1.78025700	1.91820700	-1.14526000
H	-1.67653100	3.31921600	0.01016800

20[(4)]-δ-TS

Charge=0, Multiplicity=2

C	-7.54823900	-0.83838700	-0.29485900
C	-6.53245100	-1.78427500	-0.21561400
C	-5.20701300	-1.37693300	-0.08857800
C	-4.88035300	-0.01464000	-0.03141900
C	-5.91672800	0.92798000	-0.12391200
C	-7.23837400	0.52658900	-0.25266100
H	-6.78052100	-2.84188000	-0.26111800
H	-4.43004800	-2.13263900	-0.04292200
H	-5.69857300	1.99181900	-0.08908200
O	-8.22361600	1.45864600	-0.33640300
H	-9.07703100	1.00800200	-0.41884300
O	-8.87652900	-1.13200600	-0.42257100
H	-9.01645700	-2.08828100	-0.44989700
C	-3.50386600	0.47965700	0.12290700
H	-3.39225300	1.55570700	-0.00349700
C	-0.08462900	-0.62412500	1.10744900
C	1.22874200	-0.17444400	1.29695000
C	1.58823700	1.13410500	0.95804900
C	0.64040000	1.97483200	0.37329600
C	-0.67234000	1.53836400	0.19219000
C	-1.04969300	0.24001200	0.57587600
H	-0.34171700	-1.64276200	1.38430100
H	2.59942600	1.48958300	1.12439100
H	-1.37959500	2.21621200	-0.27433400
C	-2.42475800	-0.26205500	0.42180400
H	-2.53881600	-1.33035400	0.59846800
O	0.97017200	3.22268700	-0.04055700
H	1.93815400	3.27634700	-0.14696500
O	2.17119800	-0.99894800	1.80675700
H	2.57874800	-1.73501200	0.93154900
N	4.97815700	0.01323800	1.76584200
H	4.00669400	-0.25475400	1.89818000
H	5.57508600	0.10442600	2.57741200
C	5.49898700	0.27374500	0.55080000
O	6.65975400	0.62166400	0.36831500
C	4.54880200	0.05091300	-0.64012000
H	3.50793500	0.14515300	-0.31496200
N	4.78511300	1.06669900	-1.65126400
H	5.43958700	0.86340800	-2.39746300
C	4.37635600	2.33571600	-1.50929300
O	3.70495200	2.74288300	-0.56032200
H	4.68553500	2.99582000	-2.33254000
C	4.78045000	-1.33404200	-1.25641600
H	5.83919000	-1.41479800	-1.53394300

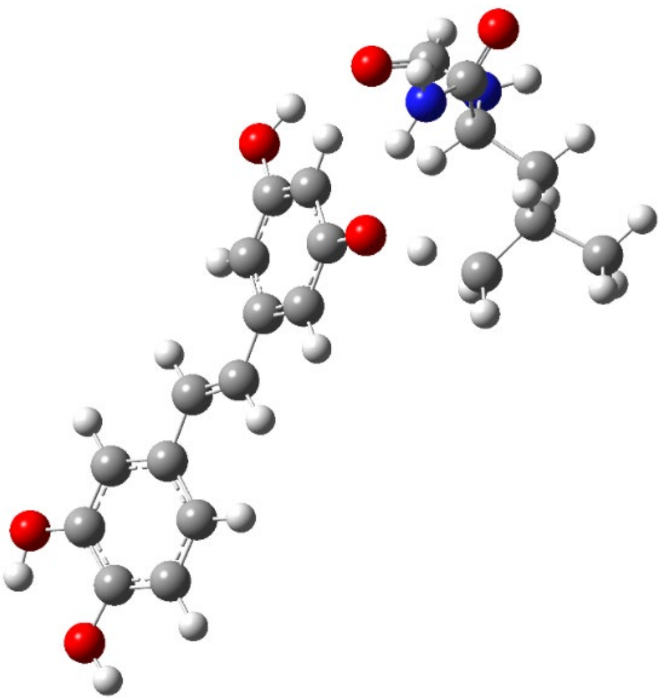

H	4.19204900	-1.38900900	-2.18175400
C	4.39892700	-2.52033500	-0.36400000
H	4.97003600	-2.47315300	0.57329600
C	4.78549400	-3.83122300	-1.07231100
H	4.22974800	-3.94075300	-2.01037300
H	4.56551100	-4.69669100	-0.44117700
H	5.85564700	-3.83823100	-1.30366100
C	2.91991600	-2.56008200	-0.04584300
H	2.25405600	-2.22324000	-0.84847500
H	2.58006500	-3.49212200	0.41384400
